# Identification of olfactory receptor genes in the Japanese grenadier anchovy *Coilia nasus*

**DOI:** 10.1007/s13258-017-0517-8

**Published:** 2017-02-23

**Authors:** Guoli Zhu, Liangjiang Wang, Wenqiao Tang, Xiaomei Wang, Cong Wang

**Affiliations:** 1 College of Fisheries and Life Science, Shanghai Ocean University, Shanghai, China; 2 Department of Genetics and Biochemistry, Clemson University, Clemson, SC USA

**Keywords:** Olfaction, Spawning migration, *Coilia nasus*, Olfactory receptor, Japanese grenadier anchovy

## Abstract

Olfaction is essential for fish to detect odorant elements in the environment and plays a critical role in navigating, locating food and detecting predators. Olfactory function is produced by the olfactory transduction pathway and is activated by olfactory receptors (ORs) through the binding of odorant elements. Recently, four types of olfactory receptors have been identified in vertebrate olfactory epithelium, including main odorant receptors (MORs), vomeronasal type receptors (VRs), trace-amine associated receptors (TAARs) and formyl peptide receptors (FPRs). It has been hypothesized that migratory fish, which have the ability to perform spawning migration, use olfactory cues to return to natal rivers. Therefore, obtaining OR genes from migratory fish will provide a resource for the study of molecular mechanisms that underlie fish spawning migration behaviors. Previous studies of OR genes have mainly focused on genomic data, however little information has been gained at the transcript level. In this study, we identified the OR genes of an economically important commercial fish *Coilia nasus* through searching for olfactory epithelium transcriptomes. A total of 142 candidate MOR, 52 V2R/OlfC, 32 TAAR and two FPR putative genes were identified. In addition, through genomic analysis we identified several MOR genes containing introns, which is unusual for vertebrate MOR genes. The transcriptome-scale mining strategy proved to be fruitful in identifying large sets of OR genes from species whose genome information is unavailable. Our findings lay the foundation for further research into the possible molecular mechanisms underlying the spawning migration behavior in *C. nasus*.

**Electronic supplementary material:**

The online version of this article (doi:10.1007/s13258-017-0517-8) contains supplementary material, which is available to authorized users.

## Introduction

The accurate perception and discrimination of diverse odorant molecules from the surrounding environment is essential for the survival of vertebrates. Vertebrates distinguish these numerous chemical cues through the olfactory system via olfactory receptors, which are G protein-coupled receptors with seven-transmembrane domains that are encoded by olfactory receptor (OR) genes (Mombaerts 2004; Kaupp 2010; Niimura and Nei 2005; Hino et al. 2009).

Four groups of ORs, including vomeronasal type receptors (Saraiva and Korsching 2007; Ryba and Tirindelli 1997); main odorant receptors (MORs) (Buck and Axel 1991; Alioto and Ngai 2005), trace amine-associated receptors (TAARs) (Liberles and Buck 2006) and formyl peptide receptor-like proteins (Riviere et al. 2009), have been identified in the mammalian olfactory organ. Due to lack of a vomeronasal system, all the types of ORs are expressed in the olfactory epithelium in nasal cavity in fish (Naito et al. 1998; Pfister and Rodriguez 2005; Cao et al. 1998; Asano-Miyoshi et al. 2000). Correspondingly, V1Rs in fish are also named as ORAs (ORs related to class A GPCRs) (Saraiva and Korsching 2007; Johnstone et al. 2008), while V2Rs in fish are also named as OlfCs (ORs related to class C GPCRs) (Alioto and Ngai 2006).

The Japanese grenadier anchovy *Coilia nasus*, an ocean-river anadromous fish with spawning migration behavior, lives in the Yangtze River, coastal waters of China and Korean peninsula, and also in the Ariake Bay in Japan (Whitehead et al. 1998; Zhu et al. 2014; Du et al. 2014; Shen et al. 2015; Duan et al. 2015; Zhou et al. 2015). At spawning time every spring, mature *C. nasus* individuals migrate over long distances from ocean to freshwater lakes to spawn (Whitehead et al. 1998; Zhu et al. 2014). To better understand the spawning migration of *C. nasus* to enable its conservation, it is necessary to study the potential migration genes that govern *C. nasus*.

Anadromous fish return to natal streams to spawn via olfactory cues (Wisby and Hasler 1954; Nordeng 1971, 1977). Two olfactory hypotheses for salmon imprinting and homing have been proposed, including an imprinting hypothesis in coho salmon (*Oncorhynchus kisutch*) and a pheromone hypothesis in Arctic char (*Salvelinus alpinus*) and Atlantic salmon (*Salmo salar*) (Wisby and Hasler 1954; Nordeng 1971, 1977). Studies in salmonids and American eels have concluded, through altering olfactory organs, that functional olfactory ability is essential to accurate spawning migration (McBride et al. 1964; Tarrant 1966; Jahn 1967; Doving et al. 1985; Yano and Nakamura 1992; Barbin et al. 1998).

If odorants are involved in fish homing migration, then olfactory receptors should play a critical role in the dissipation of information from water. Therefore, before we study the relationship between olfaction and spawning migration behavior, it is essential to identify the OR genes expressed in the olfactory epithelium from *C. nasus*. Recently, the most recently identified fish OR genes have primarily come from genome databases, and whether they are actually expressed in fish olfactory epithelia is unknown (Alioto and Ngai 2005; Chen et al. 2010; Hashiguchi and Nishida 2006; Nikaido et al. 2013; Saraiva and Korsching 2007; Hashiguchi et al. 2008; Zhou et al. 2011). Therefore, the study of OR genes at the expression level is also necessary.

Transcriptional profiling gained from high-throughput sequencing can provides insights into gene expression patterns, but also generates sequences which can be mined for gene families for use in evolution analysis. To make OR gene identification possible in *C. nasus*, whose genome information is unavailable, we researched transcriptomes corresponding to olfactory epithelium in male *C. nasus* that were produced by high-throughput sequencing technology (Zhu et al. 2014). This approach has been proven to be effective at identifying large sets of ORs (Liu et al. 2012; Bengtsson et al. 2012; Julien and Leal 2011; Emmanuelle et al. 2012). In this work, we attempted to identify the olfactory genes including OlfCs/V2Rs, MORs, TAARs and FPRs in *C. nasus*.

## Materials and methods

### De novo transcriptome

Unigenes were obtained from our previous study on de novo transcriptomes of *C. nasus* olfactory epithelium, which were uploaded to the NCBI Sequence Read Archive (SRA) sequence database with an accession number of SRP035517 (Zhu et al. 2014).

### Gene identification and functional annotation

Unigenes assembled in olfactory epithelium transcriptomes were aligned to protein databases, including NR (ftp://ftp.ncbi.nih.gov/blast/db/), NT (ftp://ftp.ncbi.nih.gov/blast/db/), Swiss-Prot (ftp://ftp.uniprot.org/pub/databases/uniprot/previous_releases/), KEGG (http://www.genome.jp/), gene ontology (http://www.geneontology.org/), and COG (http://www.ncbi.nlm.nih.gov/COG/), through BLASTx with an E-value <0.00001. The putative coding regions of the unigenes were predicted according to proteins with the highest ranks, and the amino acid sequences were translated from the coding region sequences with a standard codon table. The predicted coding regions and corresponding amino sequences of unigenes that failed to be matched to the above protein databases were based on nucleotide sequence (5′–3′) direction and obtained using ESTScan v3.0.2 (http://www.ch.embnet.org/software/ESTScan2.html). The secondary structures of all of the identified proteins in *C. nasus* were predicted with TMHMM2.0 (http://www.cbs.dtu.dk/services/TMHMM/). The presence of signal peptides in the protein sequences was predicted using the online software SignalP 4.1 (http://www.cbs.dtu.dk/services/SignalP/).

### Phylogenetic analysis of OR genes

The phylogenetic reconstruction that was implemented for the analysis of MOR was performed based on the amino acid sequences of candidate OR genes and corresponding data sets that were collected from other species. Amino acid sequences were aligned using Clustal X (Thompson et al. 1997). Unrooted trees were constructed by a neighbor-joining method with Poisson correction of distances, as implemented in MEGA5 software (Tamura et al. 2011). A phylogenetic analysis of OlfC genes was not performed because most of the identified OlfC sequences were so shorter that they did not have a consensus sequence.

To investigate the evolution of TAARs, a phylogenetic tree that was created by aligning already known TAAR amino acid sequences from 13 other vertebrate species, including Atlantic salmon (*Salmo salar*), medaka (*Oryzias latipes*), stickleback (*Gasterosteus aculeatus*), fugu (*Takifugu rubripes*), tetraodon (*Tetraodon nigroviridis*), lizard (*Anolis carolinensis*), frog (*Xenopus tropicalis*), zebrafish (*Danio rerio*), alligator (*Alligator mississippiensis*), chicken (*Gallus gallus*), mouse (*Mus musculus*), human (*Homo sapiens*) and Japanese Grenadier Anchovy (*C. nasus*), using MEGA5 software (Tessarolo et al. 2014; Tamura et al. 2011). The human rhodopsin receptor and several biogenic amine receptors from zebrafish, human and chicken were used as out-groups following a previous study (Tessarolo et al. 2014).

### Sample collection and DNA extraction

The anadromous fish were captured in May 2014 by the fisherman in the Yangtze River in Jingjiang, Jiangsu Province with the special fishing license No. SuChuanBu 2014 ZX-M025 for *C. nasus* in the Yangtze River and the fishing license No. SuChuanBu (2011) JMF217 permitted by Jiangsu Provincial Oceanic and Fishery Bureau permitted by Chinese Ministry of Agriculture.

The sample collection work was performed according to the method in our previous work (Zhu et al. 2014). Immediately captured live fishes were buried into the medical ice bags. Three minutes later, the captured fish loss of consciousness were rapidly dissected on ice. The needed tissues in this study were collected and then stored in RNAlater (Ambion, USA). All the efforts were made in order to minimize the suffering of fish. Then the fishes died under the statement of loss of consciousness.

Total RNA from the female olfactory sac and male olfactory sac, liver, heart, gill, muscle, ovary, testis, eye, and stomach were extracted respectively. Then, the cDNA was prepared through reverse transcription. In addition, the genomic DNA of *C. nasus* was also extracted from the muscle tissue for further research in this study.

### Validation of identified OR genes and genomic analysis

In order to validate the sequences of identified OR genes, PCR amplifications were performed with approximately 50 ng of genomic DNA in 25 µl reaction volumes using DNA Taq plus polymerase (Tiangen, Shanghai). All of the primers were designed according to the OR sequences obtained by transcriptome sequencing and are listed in Supplementary Text 1. PCR reactions were performed as follows: initial denaturation for 5 min at 95 °C, 30 cycles of denaturation for 45 s at 95 °C, annealing for 45 s at the corresponding temperature, extension for 1 min at 72 °C, and a 10 min additional extension procedure at 72 °C, followed by a hold at 4 °C. PCR products were subjected to electrophoresis, and then target DNA bands were isolated and sequenced. BIOEDIT software was used to analyze the obtained sequences (Hall 1999).

### Expression analysis by semi-quantitative reverse transcription PCR

In order to study the expression of candidate ORs identified in transcriptome data, semi-quantitative reverse transcription PCR reactions were performed with cDNAs prepared from the female olfactory sac, male olfactory sac, liver, heart, gill, muscle, ovary, testis, eye, and stomach as described above. An GAPDH (glyceraldehyde-3-phosphate dehydrogenase) gene fragment from *C. nasus* was used as control for its constitutive and stable expression in most cells and tissues (Zhu et al. 2016; Wang et al. 2016). Primer sequences designed are shown in Supplementary Text 2. PCR reactions were performed under the following conditions: initial denaturation for 5 min at 95 °C, 30 cycles of denaturation for 45 s at 95 °C, annealing for 45 s at the corresponding temperature, extension for 30 min at 72 °C, and a 10 min additional extension procedure at 72 °C, followed by a hold at 4 °C. Then, PCR amplification products were analyzed in 1% agarose gel.

## Results and discussion

### Data mining of OR genes in transcriptome data

#### Identification of V2R/OlfC genes in *C. nasus*

We identified an estimated repertoire of V2R/OlfC gene candidates in *C. nasus* from transcriptome data. Within *C. nasus* olfactory epithelium transcriptomes, 52 different transcripts encoding OlfC gene sequences were identified. Information on unigene reference, gene length, BLASTx best hit, CDS length and so on of the putative OlfCs is listed in Supplementary Text 5. The nucleotide lengths of the OlfC genes ranged from 227 to 3463 bp. The nucleotide and protein sequences of the putative OlfC sequences are listed in Supplementary Text 3 and Supplementary Text 4. Among the OlfC sequences, 13 transcripts were identified as having 7 transmembrane regions. Two OlfCs were detected as having 9 transmembrane regions, and two had 8 transmembrane regions. From our analysis, 20 of the OlfC sequences were detected to have signal peptide sequences.

With respect to the annotation results, most (43) of the OlfC genes were annotated as identified OlfC genes from teleost fish. These data suggest good results for the annotation of OlfC genes. However, five sequences (CL14841.Contig2_All, Unigene24070_All, Unigene28241_All, Unigene65091_All, and Unigene77993_All) were blasted to V2R genes from *Mus musculus* (Supplementary Text 5). The reason for this result may be that the five genes are orthologous to related V2R genes in *Mus musculus*. It should be noted that 37 OlfC sequences identified in *C. nasus* were matched to the corresponding OlfC genes from *S. salar*, which has the same spawning migration behavior as *C. nasus*.

This study is the first to describe the global transcripts of OlfC genes in the olfactory epithelium of a migratory fish species with spawning migration behavior. Until now, OlfC genes from several fish have only been extensively retrieved when genomic data were available (Hashiguchi and Nishida 2006, 2009; Johnstone et al. 2012; Nikaido et al. 2013; Hashiguchi et al. 2008; Shi and Zhang 2007). Thus, the number of functional OlfC genes that have been identified have already exhibited differences from 11 genes in *Tetraodon nigroviridis* and 53 genes in *Tanakia lanceolata* (Table 1). Therefore, we may predict that the OlfC gene repertoire of *C. nasus* contains more than 52 members. The number of identified OlfC genes in *C. nasus* is relatively larger than what has been found in other teleost fish (Table 1).

**Table 1:** The identified V2R/OlfC genes from some aquatic animal

Species	OlfC functional (pseudogene) gene number	Article
*Coilia nasus*	13 (39)	This study
*Danio rerio*	45 (9)	Hashiguchi and Nishida (2006)
*Tanakia lanceolata*	54 (2)	Hashiguchi and Nishida (2009)
*Salmo salar*	29 (26)	Johnstone et al. (2012)
*Haplochromis chilotes*	61	Nikaido et al. (2013)
*Oryzias latipes*	17 (19)	Hashiguchi and Nishida (2006)
*Gasterosteus aculeatus*	23	Hashiguchi et al. (2008)
*Tetraodon nigroviridis*	11 (11)	Hashiguchi and Nishida (2006)
*Takifugu rubripes*	27 (12)	Hashiguchi and Nishida (2006)
*Xenopus laevis*	249 (448)	Shi and Zhang (2007)

Pseudogenes are shown in brackets. It should be noted that the V2R/OlfC genes in *Coilia nasus* predicted to encode seven-transmembrane proteins were defined as functional genes, while the remaining genes were defined as pseudogenes

OlfC subfamily gene expansions have been identified in other teleost fish (Table 1) (Johnstone et al. 2009). These differences in gene numbers may reflect species-specific evolutionary requirements for olfaction (Johnstone et al. 2009). In our data, many transcripts were identified to encode the same OlfC genes, such as the subfamily 17. The production of these sequences was potentially either from selective slicing or expanded copies of OlfC genes.

OlfCs in mammals are involved in chemical communication via the detection of peptides that are released by individuals. In mice, a peptide pheromone that is excreted from extraorbital lacrimal glands in male individuals was predicted to be discriminated by OlfCs. Female three-spined stickleback (*Gasterosteus aculeatus*) assess the degree of major histocompatibility complex (MHC) diversity in their potential mates by detecting peptides for MHC ligands. OlfCs may be used as receptors to detect and bind the small peptides that serve as ligands for MHC. OlfCs are also predicted to act as amino acid-detecting receptors, and three receptors, OlfC 5.24, OlfCc1 and OlfC ZO6, which were identified in zebrafish and goldfish have been proven to be activated by amino acids (Speca et al. 1999; DeMaria et al. 2013; Luu et al. 2004). Eight amino acids, a signature motif of other amino acid-sensing ligand-binding receptors, are conserved in the OlfC gene products of zebrafish (Alioto and Ngai 2006).

It should be noted that amino acids, which are known as the most common odorant elements in natural waters, play important roles in many vital movements in fish (Hara 1994). A previous study has suggested that amino acids derived from streams and rivers function as possible home-stream olfactory elements for the masu salmon *Oncorhynchus masou* (Shoji et al. 2000). Furthermore, the amino acid l-kynurenine has been proven to function as a sex pheromone in female masu salmon to advise their readiness for mating to males (Yambe et al. 2006). In rose bitterling (*Rhodeus ocellatus*), amino acids were reported to induce sperm ejaculation (Kawabata 1993). A single dissolved amino acid in a natal stream can be imprinted by one-year-old lacustrine sockeye salmon (*Oncorhynchus nerka*) before and during parr-smolt transformation (PST) (Yamamoto et al. 2010). Moreover, several OlfC genes, OlfC 2.2, 3.1, 4.9, 13.1, 15.1, 16.1 and 17.1, are differentially expressed in juvenile anadromous salmon compared to returning adults in both populations of anadromous Atlantic salmon have been identified, while no statistical differences in the expression levels of these genes have been detected in the non-anadromous population (Johnstone et al. 2011). This increasing evidence collectively suggests that the odorant elements that are used by anadromous fish during spawning migration may be derived from the amino acid composition of their spawning ground water. In the result, the *C. nasus* possesses many similar transcripts of these OlfC subfamilies have been identified.

Therefore, the OlfC genes identified in this study should be considered in future research of the molecular mechanisms underlying spawning migration behavior in *C. nasus*.

#### Identification of MOR genes in *C. nasus*

Bioinformatic analysis led to the identification of a total of 142 different sequences encoding candidate MORs. Information on unigene reference, gene length, BLASTx best hit, CDS length and so on of the putative MOR genes is listed in Supplementary Text 6. The nucleotide and protein sequences of all of the putative MORs are listed in Supplementary Text 3 and Supplementary Text 4. A total of 49 sequences were identified as having 7 transmembrane regions, and two sequences had 8 transmembrane regions. In addition, a signal peptide was detected in 59 sequences.

Most (134) of the putative MOR genes were successfully annotated as already known fish MOR genes. These results suggest a better annotation result for the putative MOR genes. However, some of the putative MOR genes that were identified in this study were annotated as mammal MOR genes.

**Fig. 1:**
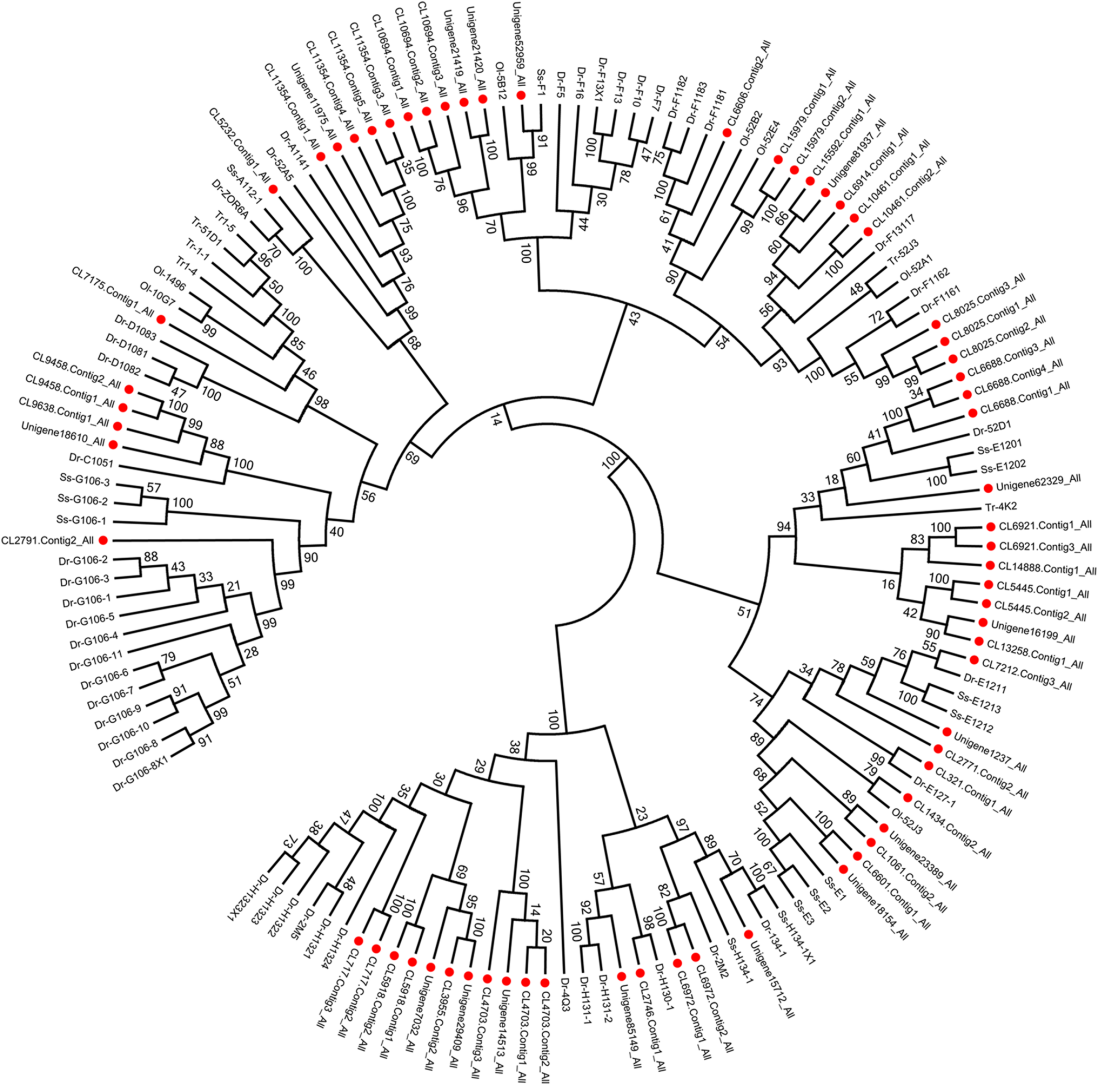
Phylogenetic tree of 138 candidate MOR amino acid sequences from six species of teleost fishes including *Latimeria chalumnae* (Lc), *Oryzias latipes* (Ol), *Salmo salar* (Ss), *Danio rerio* (Dr), *Takifugu rubripes* (Tr) and *Coilia nasus*. All the MORs are shown by species latin name abbreviations and gene names, except for the MORs from *Coilia nasu*s are shown by corresponding Unigene names. The clades containing *Coilia nasus* MORs are indicated by *red solid circle*

A MOR gene tree was created with the MOR genes that were identified from *C. nasus* and relevant MOR genes from 6 other species (frog, zebrafish, Atlantic salmon, medaka, stickleback, fugu and pufferfish). The phylogenetic tree shows the relationships of MORs between different species. The annotation of the MORs in *C. nasus* was confirmed through sequence similarity analysis (Fig. 1): they all clustered with corresponding MORs identified from teleost fish.

MORs, which can detect a wide range of chemical compounds, are recognized as the most important olfactory receptors in detecting environmental odorant elements in vertebrates (Buck and Axel 1991; Quignon et al. 2005). The first MORs were identified in the rat genome (Buck and Axel 1991). In mammalian genomes, MORs constitute a large multigene family that contains tens to thousands of members (Niimura 2012). To date, a large number of MOR genes have been identified from various species (Irie-Kushiyama et al. 2004; Alioto and Ngai 2005; Quignon et al. 2005; Hashiguchi et al. 2008; Kolmakov et al. 2008; Niimura 2009; Chen et al. 2010; Zhou et al. 2011; Johnstone et al. 2012) (Table 2). Information on MOR gene numbers in fish species suggest that they have an OR gene repertoire size that is about five- to ten-fold smaller than that of mammalian species (Alioto and Ngai 2005; Ngai et al. 1993; Barth et al. 1997), however, in teleost fish, the MOR genes are much more diverse than in mammals (Niimura and Nei 2005).

**Table 2:** The identified MOR genes from some aquatic animals

Species	MOR functional gene (Pseudogene)	References
*Coilia nasus*	49 (93)	This study
*Branchiostoma floridae*	31	Niimura (2009)
*Petromyzon marinus*	32 (26)	Niimura (2009)
*Callorhinchus milii*	1	Niimura (2009)
*Carassius auratis*	41	Kolmakov et al. (2008)
*Misgurnus anguillicaudatus*	24 (2)	Irie-Kushiyama et al. (2004)
*Danio rerio*	154 (21)	Niimura (2009)
*Salmo salar*	24 (24)	Johnstone et al. (2012)
*Oryzias latipes*	68 (24)	Niimura (2009)
*Gasterosteus aculeatus*	102 (55)	Niimura (2009)
*Larimichthys crocea*	111	Zhou et al. (2011)
*Tetraodon nigroviridis*	43 (10)	Niimura (2009)
*Takifugu rubripes*	47 (39)	Niimura (2009)
*Xenopus tropicalis*	824	Niimura (2009)

Pseudogenes are shown in brackets. It should be noted that the V2R/OlfC genes in *Coilia nasus* predicted to encode seven-transmembrane proteins were defined as functional genes, while the remaining genes were defined as pseudogenes

In the olfactory imprinting hypothesis, it is proposed that specific odorant cues from natal stream waters are imprinted by anadromous fish through their olfactory systems during downstream migration and that adult salmon memorize these odorant factors to distinguish their natal streams during homing migration (Wisby and Hasler 1954; Dittman and Quinn 1996). Then, the detection of the thousands of olfactory cues that exist in water may require the involvement of a considerable number of MORs.

#### Identification of TAAR genes in *C. nasus*

Contigs from olfactory epithelium transcriptomes of *C. nasus* were queried. In total, we identified 32 TAAR candidates in the transcriptome data. Information on unigene reference, gene length, BLASTx best hit, CDS length and so on of the putative TAARs is listed in Supplementary Text 7. The nucleotide and protein sequences of all of the putative TAAR genes are listed in Supplementary Text 3 and Supplementary Text 4. All of the TAAR genes of *C. nasus* are labeled according to the identity of the contigs from which they were identified rather than being named based on a scheme proposed by Lindemann (Lindemann et al. 2005). The lengths of all of the putative TAAR genes ranged from 205 to 1900 bp. 12 sequences were predicted to include seven-transmembrane domains as well as N-terminal domains existing in extracellular space and C-terminal domains existing in cytosol. From our analysis, 12 of the TAAR genes were detected to have signal peptide sequences.

In addition, 26 of all of the putative TAAR genes in *C. nasus* were successfully annotated as already known teleost fish TAAR genes. The remaining 6 TAAR genes in *C. nasus* were identified via BLASTx as TAAR genes from mammals. This is consistent with findings that have shown that Class I and Class III TAAR genes are present in teleost fish and that Class I and Class II genes are present in both tetrapods and teleosts (Hussain et al. 2009). The 6 TAAR genes discussed above may be the most highly related to mammalian TAAR genes from an evolutionary point of view and may belong to Class I.

Through analysis of the amino acids that are predicted to be encoded by the TAAR genes, we found that 19 TAAR proteins corresponding to the identified TAAR genes possessed a TAAR fingerprint motif, NSXXNPXX(Y/H)XXX(Y/F)XWF, which suggests that putative TAAR genes were successfully identified in this work (Lindemann et al. 2005) (Supplementary Text 4). Fifteen sequences did not contain this motif because the fragments were too short.

The above is the smallest possible estimate of TAAR gene number in *C. nasus* because in this study TAAR sequences were identified from transcriptome data that was sequenced from *C. nasus* olfactory epithelia during a specific phase. Despite this, until now, *C. nasus* has possessed the largest published TAAR repertoire compared to TAAR genes that have been identified in other teleost fish, except for *D. rerio* and *Gasterosteus aculeatus* (Tessarolo et al. 2014; Hussain et al. 2009) (Table 3). If the prediction that TAAR genes in *C. nasus* play a role in spawning migration is correct, its large TAAR gene repertoire would be helpful and necessary to the detection and discrimination of odorant elements in water.

**Table 3:** The identified TAAR genes from some aquatic animals

Species	TAAR functional gene numbers (I type, II type, III type)	References
*Coilia nasus*	32	This study
*Lampetra japonicum*	0 (0, 0, 0)	Tessarolo et al. (2014)
*Latimeria chalumnae*	18 (1, 17, 0)	Tessarolo et al. (2014)
*Callorhinchus milii*	2 (1, 1, 0)	Tessarolo et al. (2014)
*Danio rerio*	112(7, 18, 87)	Hussain et al. (2009)
*Salmo salar*	27 (5, 0, 22)	Tessarolo et al. (2014)
*Oryzias latipes*	25 (6, 0, 19)	Hussain et al. (2009)
*Gasterosteus aculeatus*	48 (4, 0, 44)	Hussain et al. (2009)
*Takifugu rubripes*	18 (7, 0, 11)	Hussain et al. (2009)
*Tetraodon nigroviridis*	18 (9, 0, 9)	Hussain et al. (2009)
*Xenopus laevis*	3 (1, 2, 0)	Hussain et al. (2009)
*Alligator mississippiensis*	8 (1, 7, 0)	Tessarolo et al. (2014)

It should be noted that the TAAR functional gene numbers in the TAAR class I, II and III in *Coilia nasus* were not counted due to several TAAR transcripts were too short to be predicted their accurate belonging TAAR class types

TAARs, as olfactory receptors, can recognize trace amine substances and related compounds (Liberles and Buck 2006; Hussain et al. 2009; Liberles 2009). From the first identified TAAR gene in mammals, a TAAR gene repertoire in aquatic animals has been identified (Table 3). Amazingly, the TAAR gene repertoire in fish is much larger than in mammals, although the opposite holds true for the remaining olfactory receptor families, including MORs, V1Rs/ORAs and OlfCs/V2Rs. From an evolutionary standpoint, the TAAR gene family is not present in lamprey, a jawless vertebrate, and therefore this family is younger than other olfactory receptor families (Hussain et al. 2009). The TAAR gene family has been divided into three classes: Class I–III (Hussain et al. 2009). Class I and Class II TAARs include both tetrapod and teleost genes, and the expansion event that led to Class III has been found strictly in fish genomes (Hussain et al. 2009; Tessarolo et al. 2014). Therefore, TAARs may perform species-specific functions in teleost fish.

A phylogenetic analysis of 17 longer TAARs identified in this work was performed with MEGA5 software using the corresponding amino acid sequences (Tamura et al. 2011). We found that the putative TAARs in *C. nasus* could be separated into three clades. Three TAARs belonged to Class I, and three TAARs belonged to Class III. Interestingly, one TAAR belonged to Class II, which has been lost in other teleost fish, except for zebrafish. It should be noted that our statistical results are not comprehensive, as the phylogenetic analysis contained only 17 sequences, and they were not whole sequences.

**Fig. 2:**
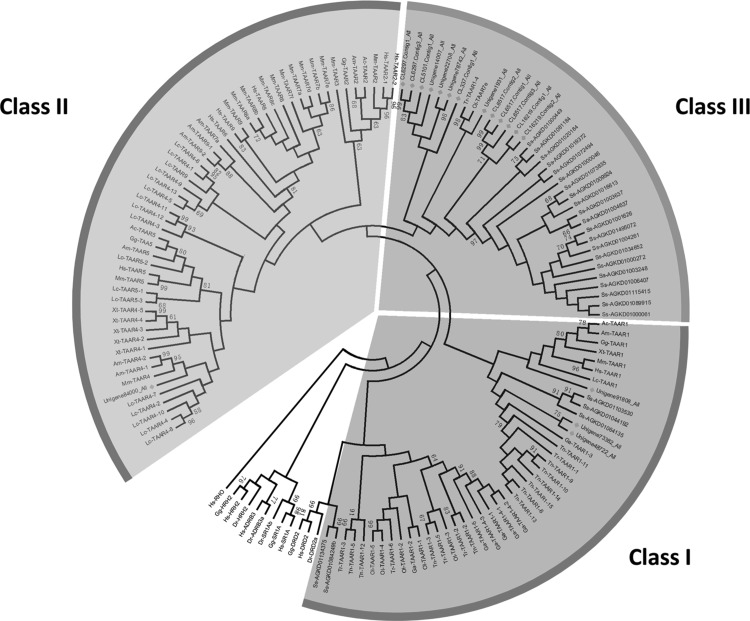
Phylogenetic analysis of 133 candidate functional TAAR amino acid sequences identified fourteen species including *Latimeria chalumnae* (LC), *Oryzias latipes* (Ol), *Salmo salar* (Ss), *Danio rerio* (Dr), *Takifugu rubripes* (Tr), *Tetraodon nigroviridis* (Tn), *Gasterosteus aculeatus* (Ga), *Alligator mississippiensis* (Am), *Xenopus tropicalis* (Xt), *Homo sapiens* (Hs), *Mus musculus* (Mm), *Gallus gallus* (Gg), *Anolis carolinensis* (Ac) and *Coilia nasus*. Rhodopsin receptor from *Homo sapiens* (Hs-RHO); histamine H2 receptors from *Gallus gallus* (Gg-HRH2), Homo sapiens (Hs-HRH2) and *Danio rerio* (Dr-HRH2); adrenergic receptor beta 3 from *Homo sapiens* (Hs-ADRB3); adrenergic receptor beta 3a from *Danio rerio* (Dr-ADRB3a); serotonin receptor 1Ab from *Danio rerio* (Dr-SR1AB); serotonin receptor 1 A from *Gallus gallus* (Gg-SR1A) and *Homo sapiens* (Hs-SR1A); dopamine receptor D2a from *Danio rerio* (Dr-DRD2a); and dopamine receptor D2 from *Gallus gallus* (Gg-DRD2) and *Homo sapiens* (Hs-DRD2) were utilized as outgroups. All the TAARs are indicated by species latin name abbreviations and gene names, except for the TAARs from *Salmo salar* are indicated by species latin name abbreviations and Genebank accession numbers from ASalBase/NCBI and the TAARs from *Coilia nasus* are indicated by corresponding Unigene names. The clades including TAARs from *Coilia nasus* are indicated by *green solid diamond*

Within each class, there are distinct species-specific expansions, which are shown in Fig. 2. Therefore, TAARs may perform species-specific functions, which is unique to teleost fishes. Many of the contigs were annotated as trace amine-associated receptor 7 g and trace amine-associated receptor 14. An expansion event that affected specific TAARs in *C. nasus* may therefore be suggested. Thus, Class III TAARs seem to have gained a novel set of ligands and appear to have eventually evolved into a new olfactory receptor gene family. It is interesting to examine whether they were involved in spawning migration in *C. nasus*.

Furthermore, the fact that *D. rerio, S. salar* and *C. nasus* all exhibit the expansion of TAAR genes suggests that Class III TAARs may have a more important role in *C. nasus*. Furthermore, based on the created phylogenetic tree, we found that five sequences were closer to TAARs from *S. salar*. Both *C. nasus* and *S. salar* have the ability to perform spawning migration. Therefore, these sequences should be paid attention to in future research. The identification of TAARs in *C. nasus* will facilitate further functional studies and help to identify possible relationships with spawning migration behaviors.

#### Identification of FPR genes in *C. nasus*

From transcriptome data, we identified two putative FPR genes with lengths of 388 and 333 bp. Information on unigene reference, gene length, BLASTx best hit, CDS length and so on of the putative FPR genes is listed in Supplementary Text 8. The nucleotide and protein sequences of all of the putative FPR genes are listed in Supplementary Text 3 and Supplementary Text 4. Neither of the genes was full-length, and they were not detected to possess a signal peptide sequence.

Formyl peptide receptors (FPRs) are found in all mammals; humans and mice encode three and seven FPR genes, respectively (Migeotte et al. 2006). Formyl peptide receptor-like proteins were proven to be a novel family of vomeronasal chemosensors in 2009 and through olfactory function can identify pathogenic states (Riviere et al. 2009). To the best of our knowledge, few reports on FPR genes in teleost fish have been published.

### Identification of olfactory genes with more than one coding exon

The products of CL13258.Contig2_All, Unigene52959_All, CL3955.Contig1_All, Unigene34891_All, Unigene87980_All, CL10694.Contig2_All, Unigene16290_All, Unigene18154_All, Unigene68575_All, Unigene68576_All, Unigene95217_All, CL16219.Contig1_All, Unigene101623_All, and Unigene3763_All, which are all annotated to be MOR genes, were highly similar to the DNA fragments that were identified from transcriptome data (Supplementary Text 1). These results suggest that the sequences in the transcriptome database were adequate and reliable.

According to published articles, MOR genes are usually intronless in their coding sequences (Glusman et al. 2000; Nef et al. 1992). In Unigene59895_All, CL10694.Contig4_All, CL5232.Contig2_All and CL12962.Contig1_All, which are annotated to be MOR genes, sequences longer than the corresponding fragments in the transcriptome databases were produced. Through sequence alignments, we found that each of these genes had more than one coding exon. Previous studies have reported the identification of olfactory genes with more than one coding exon (Azzouzi et al. 2014). However, these results were predicted only by sequence analysis. In our work, we report such results based on transcriptional analysis, and our findings here compliment this previous study. Through analysis, all four of the sequences were found to possess the standard “GT/AG” splice site. The unusual results implied that a unique olfactory receptor gene repertoire as well as the spliced OR genes were present in *C. nasus*.

In addition, with respect to CL321.Contig2_All, which is annotated to be MOR genes, three sequences with different lengths were produced. One sequence possessed the same length as the sequence fragment of CL321.Contig2_All, while the other two sequences were longer than the target fragment of CL321.Contig2_All. This suggests that there may be several copies of genes encoded by CL321.Contig2_All in the genome of *C. nasus*. Therefore, an expansion event of this gene may have occurred in *C. nasus*.

### Tissue- and sex-specific expression of putative *C. nasus* OR genes

**Fig. 3:**
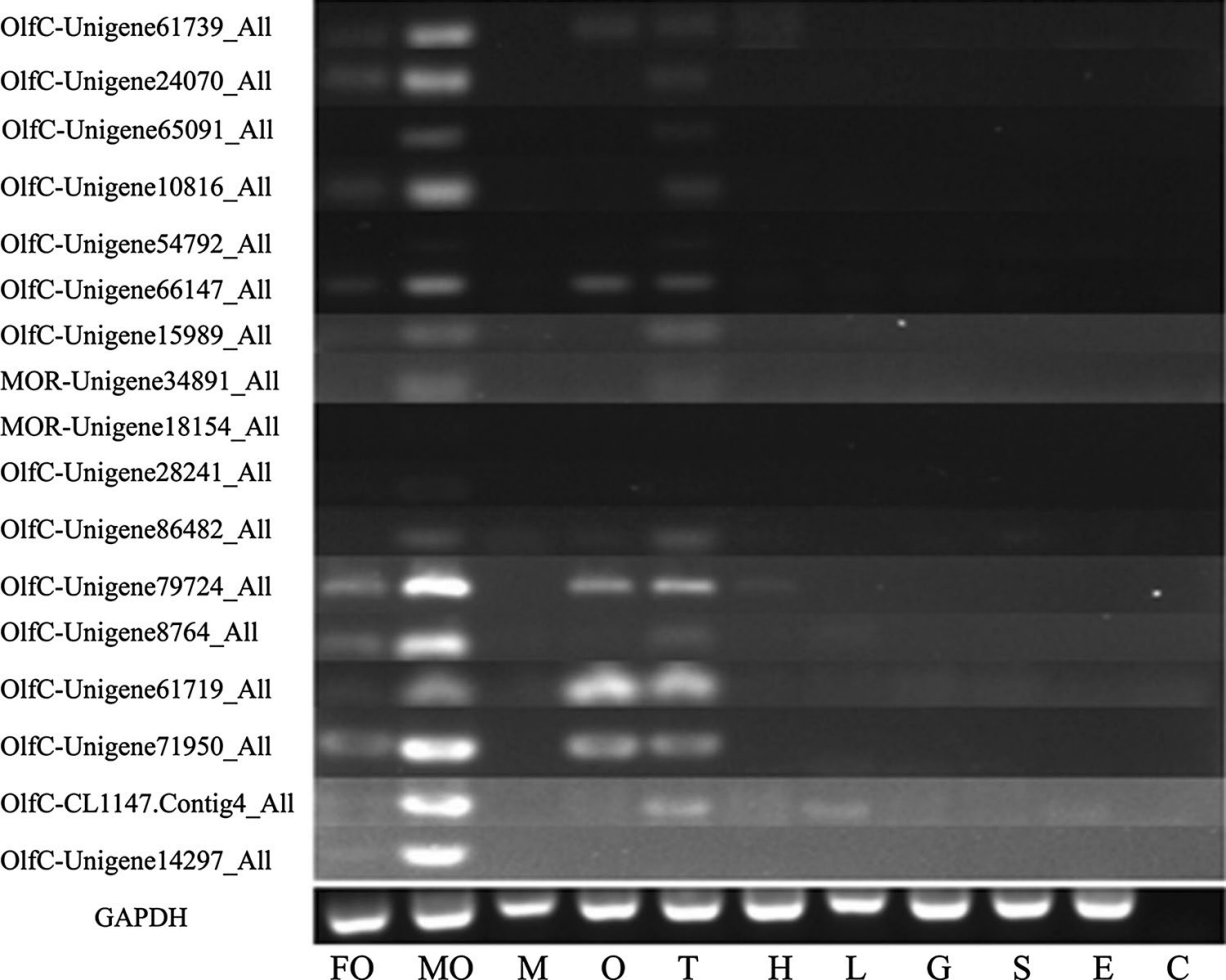
Tissue distribution analysis of several OR genes in anadromous *Coilia nasus. FO* female olfactory sac, *MO* male olfactory sac, *M* muscle, *O* ovary, *T* testis, *H* heart, *L* liver, *G* gill, *S* stomach, *E* eye, *C* control

The expression patterns of 14 OlfC genes and two MOR genes (Unigene34891_All and Unigene18154_All) in the above mentioned ten tissues were analyzed using the semi-quantitative reverse transcription PCR (Fig. 3). The expression of all the analyzed OlfC and MOR genes were detected in olfactory sacs of *C. nasus*, with some of them were found to be expressed exclusively in the male olfactory sacs. This result is consistent with the function of olfactory receptor genes.

In addition, fourteen OR genes studied were also detected in the gonad of *C. nasus*. The accurate functional link between the olfactory sensing for spawning sits and the regulation of sexual maturation is essential to the successful spawning migration of *C. nasus*. These OR genes may be also play a part in the interaction between the detection of odour cues from spawning grounds and the regulation signals from gonad development.

It should be noted that all the analyzed OlfC genes in *C. nasus* were expressed at a higher level in the male olfactory sacs than in the female olfactory sacs. The male-biased expression differences in *C. nasus* suggest that these OlfC genes may be potential receptors of water-soluble chemical cues involved in male-specific behaviors. Anadromous fish may use water-soluble amino acids as the possible home-stream olfactory elements during their spawning migration (Hara 1994; Shoji et al. 2000; Yambe et al. 2006; Kawabata 1993; Yamamoto et al. 2010; Johnstone et al. 2011), while OlfCs are also predicted to act as amino acid-detecting receptors (Speca et al. 1999; DeMaria et al. 2013; Luu et al. 2004; Alioto and Ngai 2006). Therefore, we propose that the spawning migration of *C. nasus* may be a male-specific character. During the spawning migration of anadromous *C. nasus*, the orientation may be performed by the male migrants with higher expression levels of OlfC genes independently. Then the female *C. nasus* migrants follow behind the male migrants to swim to their spawning grounds. It is of great interest to test this hypothesis in our future research.

## Conclusion

In this study, a comprehensive analysis of olfactory receptor genes of *C. nasus* was performed. Totally, we identified 142 candidate ORs, 52 OlfCs, 32 TAARs and 2 FPRs, all of which were expressed in the olfactory epithelium of *C. nasus*. This is the first comprehensive study of olfactory receptors in *C. nasus*, an important commercial fish possesses spawning migration behavior in China. This study demonstrates that high-throughput sequencing data provide a good resource for the recovery of expressed olfactory genes from different sub-families. Our results lay the foundation for the further research of the possible relationships between olfactory reception and spawning migration of *C. nasus*.

## Electronic supplementary material

Below is the link to the electronic supplementary material.

Supplementary Text 1. Primer used in the validation of OR sequences and Sequences obtained. (DOCX 20 KB)

Supplementary Text 2. Primers used in RT-PCR. (DOCX 16 KB)

Nucleotide sequences of identified OR genes in *Coilia nasus*. (DOCX 125 KB)

Amino acid sequences of identified OR genes in *Coilia nasus*. (DOCX 49 KB)

Putative identified OlfC genes in *Coilia nasus*. (DOCX 20 KB)

Putative identified MOR genes in *Coilia nasus*. (DOCX 35 KB)

Putative identified TAAR genes in *Coilia nasus*. (DOCX 18 KB)

Putative identified FPR genes in *Coilia nasus*. (DOCX 14 KB)
