# Supplementary material for: Identification of olfactory receptor genes in the Japanese grenadier anchovy Coilia nasus
Source: Genes Genomics. 2017 Feb 23;39(5):521–32. doi: 10.1007/s13258-017-0517-8 (PMC5387026; doi:10.1007/s13258-017-0517-8)
Supplement: Supplementary file 1 — Supplementary Text 1. Primer used in the validation of OR sequences and Sequences obtained. (DOCX 20 KB) [file 13258_2017_517_MOESM1_ESM.docx]

**Appendix. Primers used for internal PCR.**

| **OR** | **Forward Primer** | **Reverse primer** | **Tm(℃)** |
| --- | --- | --- | --- |
| **Unigene95217_All** | GTACATTGGCTGATGAAGTCTGG | ACGCGAACTACACTGTAGTTTGT | 55.2 |
| **Unigene68576_All** | ACGAGCGGCGTGTTGAGGAAGGT | CCCATCAATAGCACCTCTGTGGG | 65.0 |
| **Unigene68575_All** | GTGCCCAGCAGGATGCGCAC | TGGAGCGCTACGTGGCCATC | 65.0 |
| **Unigene59895_All** | GTGAAGCTGAGCCATATGTG | AAAGACTGGCTGCATGTATG | 56.9 |
| **Unigene18154_All** | TCATAATAATTCTAGCTGCTCCA | TTGACATCATTAATTGCTGTTATC | 54.0 |
| **CL10694.Contig2_All** | TTGACTAAAACTGACTCCAAACA | ATACAGTGCAATAATGACCACAA | 55.0 |
| **Unigene87980_All** | TACAGAAAAGAAAATGGACCTTA | TTCTCACCCCAATCTATCTAACT | 54.0 |
| **CL10694.Contig4_All** | TGACTGGCAAGTTACAAAATAGCAA | TCCTACCCGTTTCTGGGATAGTTTA | 56.9 |
| **Unigene34891_All** | GTTGTTGGAATGAAACTGGC | GTTGATTGGGAGCACAGCTG | 59.2 |
| **CL5232.Contig2_All** | TTGTACCACAAACACTTTATTG | GAGACAGACAAGAACCTTTAGAG | 55.6 |
| **CL3955.Contig1_All** | GGCCACATAGCGCTCAAGTGAC | TCATGGATCCGTTTTGTCCCTG | 60.0 |
| **CL321.Contig2_All** | CAAGTGGTATTGTAAATGGTC | CTGATATTGCACTCAGTCGAT | 55.6 |
| **CL12962.Contig1_All** | TATGATGCCATACAGAAACGAGT | CTCTGTCTCACTGGCACATAGTT | 58.2 |
| **Unigene52959_All** | AAAATGTTAATCTTGGTAAGCAG | AATTTACCACCTATGCTGAATGT | 54.6 |
| **CL13258.Contig2_All** | TCTCAAGACACGATAAACTAACA | ACAGATTTGTCTCCATATTTAGG | 54.6 |
| **CL16219.Contig1_All** | TACTGGTAAACAGTGAAGGGAACATAT | GCACAAGATTTATTTTCCATGACATAT | 60 |
| **Unigene101623_All** | AATGGCCTGGAGGATTTTGAC | GCAGCGTATTCCTTCTGGTCA | 60 |
| **Unigene3763_All** | TAGAGTTAGCCAGAAGAAGGG | ATGCCATTGTGATCTTGTACT | 53 |

**> Product of Internal PCR of Unigene34891_All**

GTTGATTGGGAGCACAGCTGTTTGGCCGTACGTCATGGTCATCCTGTTGACAAACCTGAGTATTATATCATACGAGGGCTGTTTCGTTCAGTTTTTTCTCCTAGGAAGCTATGGCAGTTGTAATTTTACAATTTTAACAGCAATGGCTTATGACAGGCTTGTCTCCATATTTAAGCCTTTGCAGTACCAAACCATCATGACCCCACAAAGAGTAAAACTGCTGTTGTTTATGGCCAGTTTCATTCCAACAAC

**>Product of Internal PCR of CL10694.Contig4_All**

TCCTACCCGTTTCTGGGATAGTTTATGAAGGTAGGCTGTGTGCATGTGTTTTCCACTCCTAATACAATCCATATTACATTACAAATGTCACAAACTCACATTTATATATAAAATGTTATTTTACAAACTATAGAAAACACAGTGTTTTATGAATCTGATTCTTTTTTCACACATAGACATCAAACAAGGATGGAAAACATTTCATCCGTCAGTAACATCCTTGTACTGGAAGAGTTGGGGCACCCGGAGTCATCCACATACGCTGTGTTCTTCACACTGCTCTTTGTCTACATTGCACTGCTAATAACTAACTTTGGTGTACTTATTATCATCATTGCAGAGAAGAGCTTGCACCAACCCATGTACTTGCTATTTTGTAACTTGCCAGTCA

**>Product of Internal PCR of Unigene95217_All**

ACGCGAACTACACTGTAGTTTGTATGTGTGCTATATATATTACATTTAATCTGCATTTTGTCATTAGTTTTAGTGTGGTTGTGCATTGCCACATGTGAAGATTTTTTTCTGTGGTCATACATTTCAGACTCTCTTTCATACAGATTATCATTTCATCATCCATGTTCATATTCTTTTTCAGGTAAGAGGGAAAAATGAATCACTCGTTTGATATCACTGTCATTTTTACTGCTTATCAATCTATTGGGGTCCAGAAGTATGCCTATTTCACCATTATTGCAGCATTATACATTTCCTCTGTATTTACCAACGTGTTCTTAATGCTTATTATCATCTGGGAGTCCAGACTTCATCAGCCAATGTAC

**>Product of Internal PCR of Unigene68576_All**

CCCATCAATAGCACCTCTGTGGGCCTCTGTGCATTATGCAAAACCTGACTAGCGCTGTCAACGCCACGTCAGACCGGAACCTGGCGGTCATCGTGAAAGTGTGCCTGGTCATCCCCATCTTCAGCGTCTTCCTCTACTTCATCGTCCTTATGCTGCACACGTTCGCCTCGCACCGCCACTTCCTGGAGAGCCCGCGCTACATCCTGTTCACCTACATGCTGGCCAACGACACGCTGCAGCTGCTCACCTCCGTGCTGCTCTTCCTGTTCGTCATGGCGCAGGTCAACTTCGCGCTGGTCTTCTGTGCACCGCTGCTCTTCTTCTCCACAGCCACCTTCCTCAACACGCCGCTCGT

**>Product of Internal PCR of Unigene68575_All**

TGGAGCGCTACGTGGCCATCTTCTACCCGCTGCAGCGCCCCGTGGCCTGGCGCGCCGACCGCATCTGGATCGTCATCGTCTGCATGTGGGTGCTGAGCTGCGTGCAACCCACGGCCGAGTTCATCATGAGCAAGCCGCGCGGCGGCGCCCAAATGGACGTCCTCACCACGCCCGTGCAGTGCAAGAGCGCCGTGCTGCACGTGGCGCCTGCAATCACGCTCTTCAAGGTGGTCCTGAACGGGCTCTTCTTCGCCCTGGTGGCCGCCGTCATCCTCTTCACGTACGTGCGCATCCTGCTGGGCAC

**>Product of Internal PCR of Unigene59895_All**

AAAGACTGGCTGCATGTATGAGCTTTTTTTTAATGCTGATAGTCAGATCATATCTAAACACAGGTGAGGCACGTTAAAAAGTATTTAATGCAAACATGTTCTGCAGTTAAGAAAGAAGACTGCTGTCTGAATTTAAATGTGTTTTGTTTCAGGTTATCAGACTGCAAGGATGAGAAACATTTCATACATCAGTGATATTCTTACCATGGAAGGATTAGAGATCGCTGAATCATCCACATATCCAGTGTTTTTCACATTACTCATTGTGTATATTGCACTGCTAATATCCAATATTGGTGTTCTTGCGGTCATCATTGCAGAGAGAAAACTACACCAGCCTATGTACTTGCTGTTCTGCAATTTGTCTGTGAATGACATTTTAGGCAATACAATCTTAATGCCCCGGGTACTGTTAGACATTGTTTCAAATGAGAAGCTGATTTCTTATAGCGCATGTGTCACACAAGCATTTTTCAGCCACACATATGGCTCAGCTTCAC

**>Product of Internal PCR of Unigene18154_All**

TTGACATCATTAATTGCTGTTATCCTTACTGTTAGAATACCTATTTGTGGATCGCACATTCATAAACTATTTTGTGATAATCCTTCAATTCTAAAGCTGGGATGTTTTCAAACCACAGTGAACAAAATATGGGGTATGATACTAATTACACTTCAGCTTATGCAATTTGTTTTCATTTTAATTTCCTATTGTCATATTGTCCAGGTTTGTGTATCATCCAGTGAGGGAAGAGCAAAGTTTACTAAAACGTGTGTGCCTCATATTTTGGTTGTTGTCATCTTCATTGCCACAACACTGTTTGATGTGTTATATAGTTGGAATGGGTCTCTACATTTTCCAATTATTGTGCGTAGTGCATTGGCTACACAGTTTTTGATTCTTCCACCACTTTTCAATCCAATAATCTATGGGTTTCAGCTTCCACAGATCCGAAAAGTCTTGTGCAGACAAAGTTGTAATCACAAAATTATGTGCAATCGTTGAGTTAACCCTAATGGATTTTCATCATCTTTGATTTGACCAAAATGTTGGAGCAGCTAGAATTATTATGA

**>Product of Internal PCR of CL10694.Contig2_All + nr**

ATACAGTGCAATAATGACCACAAAGGCTGTTTTGACTTTGTCGTTTTTTGCATGGGCTGTTTCAGTTGTATTAGTGGGTGTGTTACTTAGCCTCACAATAAGGCTATCGCGATGTAGGTCACACATTATGCATGCTTTCTGTGACAATGCATCCTTGTTCAAGCTGTCTTGTGAGGATGTGTCCATTAATAATATATATGGGCTGTTTTACACTGTGGTGTTGTTCTCCTCATCAATGGGCACTGTTGCCATCACATATATCAGAATTGCTGTCATATGCTGGACTAGGAATAATGCAGAGCTCAACAGCAAAGCCATTCAAACCTGTGCAAGCCATTTAGTGTTGTACCTGATAATGCTACTGACTGGATATGTTGTGATTATCATGCATCGGTTCCCAGAACACCGTTTTTTAAGAAAACTAATGGCTGTTCTTTTTCATGTTGTACCTGCTCATTTAAATCCAGTCATTTATGCTTTCCAGACTAAACATTTAAGGGTGAAAATATTGCAAATATTTGGTAGAAAAATTACACACAGAAATTTCTAGATATGTTTGGAGTCAGTTTTAGTCAA

**>Product of Internal PCR of Unigene87980_All**

TTCTCACCCCAATCTATCTAACTCTTATGTCCCTGGAGCGCTACGTAGCCATATGCTTTCCCCTCCGACATGCTCAGATAGCTAACAAGAGGAGAACCATGGTGGCAGTAATGGCCGTCTGGTCTCTAGGGCTAGTAATATGGACAACGGACCTAACTGTTGCCATGGTGTTTCAGGCTGGCTCCTCCAAAAGGAGCTGTTCTGATTACATACTTTCCCAAATGGTAGTTTCCTATCAGGTAAGCACAGGTTCAATAGCCCTTGTGTTCACATTGGCATGTGTTGTGATAATCTATGTATACATAGCCATTGTGGTAACTGCCAGGTCAACTACTACCAGTGACAAGTCCTCTGCGAGTAAGGCCCACAAAACAGTACTCCTGCACATGGTGCAGCTTTGCTTGTGTCTCTCATCGCTTCTAATGGGAGCGGTTCGCAGGACACTAGTTATGAGCGGGCTGGACCGAGTGCGTTATGATGAAGTGTCGTACGTTTTGTTTTTAATGTTGAACATCTTGCCTCGGTGCCTCAGTCCCATCATATATGGTCTAAGAGACAAGACTTTCTGTGCCTATTTTAAGGTCCATTTTCTTTTCTGTA

**>Product of Internal PCR of CL13258.Contig2_All**

ACAGATTTGTCTCCATATTTAGGCCCCTGCAGTACCACACCATCATAACACCATGGAAAGTAAAGCAGTTGATGATAGTTGCAAACGTGATCCCGACAGCTTTAATACTTGTTCAAATATTCATGACTTCCCAACTGCCTCAGTGTAGGTACAATGTTCATAGGACATATTGTGATAATTTAGCTGTTGTTAGTTTATCGTGTCTTGAGA

**>Product of Internal PCR of Unigene52959_All**

AATTTACCACCTATGCTGAATGTGTCACACAGGCCTTTTGTAGTCACACATTTGGATCAGCTTCACACATGATACTAATCATAATGGCCTTTGACAGGTATATTGCTATATGTAACCCATTAAGATATGCGTCAATAATGACAACAAAGACCATTGTAAAGTTGTCTGTGTCTGCCTGGGGCATTTCACTTGTGCTGGTGGCTATTTTATTGGGTCTCACAATCAGGTTGTCTCGCTGTAGGTCTGTTATTCTCAATGCGTACTGTGACAATGCATCTTTGTTTAAGTTATCTTGTGATGATGTGTCAATTAACAACCTCTATGGTCTAGTTTTCACCGCAGTACTTTTCGGCTCATCAATAGGAAGCATCCTTGTGACATACTTGAGAATAGCAATAATATGCTGGACTAAAAAAAGCAAAGAGCTAAACAACAGAGCTTTGCAAACGTGTGCAAGTCACTTGTTAGTGTACATGATTATGTTGTGGACAGGGTTCCTTACTATAATTCTCCATCGTTTCCCAGATTACCCATATTTGAGGAAACTGGCATACATACTGTTTCATGTTGTTCCTGCTAATTTGAATCCAATTATATATGGCATGCAAACAAAAACTCTACGGAAGAAAATTCTGCAGACTTTTTTAAGAAAGGTGTCGTCTACATAGGCACTCCATTTTTTCAAAACTCTCCATCTGGTTAAAATTATTGAATAACAGATTTTCTTCTAAAACCTGTGCTCACTAACTGACATAATTTGTGCCCATAGAGACTAAATCATATATTTAAATGACACCAAACATTGTTTAGAAGATTTTCTAATCCTAACTGCTTACCAAGATTAACATTTT

**>Product of Internal PCR of CL12962.Contig1_All with intron in red**

CTCTGTCTCACTGGCACATAGTTGAGACATGCATGAAGTCCTAGGAATCAGACTTAGACATGACACAGACTGGTGTTAACTCTCTCAACACAAGTTAGTGTGTTTTTAGTGGGCTGAAGGGATCCTGAATTACCATAGTTTAGAGTGCGATGTGATAGCTCAGCTTAGAGTGCTCATTTATTGTGTATGCTGATGTAAAGTTTGGATCTCCTCAGGCTGGAGTGTCTGTCAACCACCAGCACATTGTCTTTAATTATCTTTATTTGTACTGTATATTATTTAATGTTAGCTTGATGATGTACATCCTTATGCTTACTGGGAATCATTCCATTGTGATGACTTATTGTGATTAACCTGAGGTTACGCATGTATTGCCTAATCTGTTATTTTTATTGATTTACTATCTTATTTGCCTTGATGTTTAACTTGATATTTGCGTTGTATTAATTATACTGATACTTGTATTTGTATTAGTCTTAATATTTTATGGTCATGGCCGAAACAAACTCGTCCACTGAGCAGGTGCTCTTCGTCCACCAGCAGATGTACCAGGTGCAGCTGAATGAGGGCCCGCTGACTAAACTGGTGGTGGCCATCATCATGTCGCTGCTGTTTATTTGGGTCAACTGCCTGCTGCTCTTCACGCTGAGCCGCAAGCACATCTTCCGGGAGACGCCGCGCTACGTGCTCTTCTCCCACATGCTGTGCAACGACTCCATCCAGCTGCTCTTCACCTCGCTGCTCTACATCTTTTCCATGTCCTACTCGCAAGTCCACCGCGGCGCCTGCTACCTCCTCCTCTTCGTCTCCAGCTCCACCTTCCTCAACGCGCCGCTCAACCTGGCCCTCATGTCCCTCGAGCGCTACGCCGCCATCTGCTTCCCGCTGCACCACGGCAACGTGGCCACACCCACAGCCACGGCCGTTGGCATCCTTACCATATGGTTCCTGGGGGGCGTAAACGTAGTGATTGACATCTTGTACGCACTCACCAGAGATGGCACGGCCCTCGGCAATCATATGTTCTGTACACGCGAGCGCATCTTCATGGCCGTGTGGCAGGCTGACGCGTTCCAGGGATTTAACGGCTTCTACTTTGTGATCGTGACCCTGATCATCGTCTTCACCTACATCAGCGTGATGGTGGCTGCTCGCTCGGCCACTAGCAATAAAGAGTCAGCAAAGAAAGCCCACCGGACGCTCCTGCTGCACCTCATCCAGCTGGGCCTGTGCCTGAACTCGTTTCTGTATGGCACATA

**>Product of Internal PCR of CL321.Contig2_All**

**>CL321-1**

CTGATATTGCACTCAGTCGGTATGAACCTGGCCAGATTTTAAATGCCATAGTTTTGATAATGTCTCTTGAATTTATTGTGATTCCTCCGTTACTAAATCCTCTGATATATGGCCTAAACTTACCAGATATCCGAAGGAAAATTCTCAGTATAATGATTTCATTAAAAGTTGCTTTGAGTTGTTAGACATGCATTTACCAGGAACATATTGCCAGAAAGCATACTAAACCTATGAAATATATTTCTTATATTATGATGTATTTTTATGTATATAACCAGGGCTGTACTGGAGGGTAAACACTAGTAAACTGAGTTACACTACAGCCATTTACAATACCACTTG

**>CL321-2**

CTGATATTGCACTCAGTCGATTTGAACCTGGACAAATTATAAATGCGATAGCTTTGATCATTTCCCTTGAATTTATTGTGATACCTCCATTACTAAATCCTCTGATATATGGCCTAAACTTACCAGATATCCGAAGGAAAATTCTCAGTATGATGATTTCAATAAAAGTTGGTTTGAGTCCTTAAACAAATGTATTTACCAAGAACATATTTCCAGAAAGCATACTAAACCTATGAAATATATTTCTTATTTTATGATGTATTTTTATGTATATAACCAGGGCTGTACTGGAGGGTAAATGTTAGTAAACGGAGTTTACCCACCTCTCTAATGTCTACTTACAATATGTGCACTACGGACCATTTACAATACCACTTG

**>CL321-3** CTGATATTGCACTCAGTCGGTATGAACCTGGCCAGATTTTAAATGCAATAGTTTTGATCATTTCTCTTGAATTTATTGTGATTCCTCCAATACTAAATCCTCTGATATATGGCCTAAATTTACCAGGTATCCGAAGGAAAATTGGCAGTATGACGATTTCATTAAAAGTTGCTTTGAGTTCTTAGAAATGTATTTACCAGAAACATATTACCAGAAAGCATACTAAACCTATGAAATACATTTCTTATACTGTGATGTATTTTTATGTATATAACCAGGGCTGTACTGGAGGGTAAACGTTAGTAAACTGAGTTTACCCACCTCTAAAATTTCAGAGATAGAGTTTATAAATCTCTGAGGAAAGTTTATCCACATATTAATAGAGTGCATTTATATCATAATGAAGTTTATGCACCAAATACAACTTGTAATATTTCAAAGTTACACTACATAATTTATTTTTTACAATCTGTACACTAGGGGCCATTTACAATACCACTTG

**>Product of Internal PCR of CL3955.Contig1_All**

TCATGGATCCGTTTTGTCCCTGTGATTAATACATTCATATAAGATATGTGCTATGTAATGATTTTATATAGATCTTTACTTATGTTTGTATAGGCCTAATGGCCATGAATAATGAGTCCATTGAGGGCACCCTACTGGTGCATCAGCAGTTTTTTAAGGTGGAAATGACAGAGGGGCCAATGTCTAAACTTATTGTGGCCATTCTCATGGCCCTGGTTTTCATCTTTATTAACAGCATCATGTTTCACACTCTTCTAAGTAAGCCTGTGTTTAGAGAGCTGCCTCGCTATATCCTCTTTGCCCACATGCTCTGCAATGACTCAGTCCAGTTGCTGGTATGTATGACATTGTATCTTATGACTGTAAACTTACAACAGATTCCTAAAGCTCTGTGTGCATTATTTTTACTTTTTGCAACAACAACCTTTAACAACGCACCTTTGAATCTGGCTGTGATGTCACTTGAGCGCTATGTGGCC

**>Product of Internal PCR of CL5232.Contig2_All with intron in red**

GAGACAGACAAGAACCTTTAGGTATTGTGTAGCAGGTTCAGATGTGTAATGTCTGCATTTCAACTCGAATAGTCTGTTTTTGACATTAATATGTATTGTATGTGTAATGGTGTATTGCTTTGCTTGTTGTGATTGAATGTCATGTTTTTCTTTTAGAGGAAATGTCAGTGTCACTGATGTACTTATTGTTGTGAAACCTGTAGGTAGGACCGTCAGGACTGTAAATGTGCCATGATGGGGGAAGCCGAAGGAGCCAATATTTCACATGCTGTGTTCATTTTCATTGGTTTCCCAGAGACCTATGATCACAGGGACTGGTACGCAGTGCCTGTCCTCCTGAGCTACCTGCTTCTCCTGGCAGGCAACTCCCTCCTGCTCCACGTTATCCACAGCACGGCCAGCCTTCACAGCCCCATGTACGTCCTGGTCTCCGCTCTGGCCATTGTCAACATCGTGGTTCCCACGGCCATCATCCCCAAGATGCTGCTGGCGGTCCTGTTCGACCTCAGGGAGATCACCCTGGCGGGGTGCCTGGTGCAGATGTTCGTGACGCACTTCTTCTCGTCTGTGGAGTCCACCATCCTCCTGGTCATGGCCCTTGACCGGTATGTGGCCATCTGCCATCCGCTGCGCTATGTGGAGATCGTCAACAGCGCCCTGTTCGTAAAGCTGCTCGTCTTCACCCTCGTCCGCAGCGGGTCGATCATGCTGACGCTGGTTGGCCTGGTGGCGCCCCTCAGGTTTTGTGGATCGAATGTTATCAGCCACTGCTACTGCGACCACATGGCCCTCGTTAGCCTTGCGTGCAACAGCACGGACAAAAACAGCGCCATGGGGGTGGCCGTTATAGTGTGCTTTGTCGGCATCGACATATCGCTCATATTCTTCTCCTATGTCAACATTCTGTACGTTGTCCTAAGGGCAGCTGCTGGGGAGGACAGGTGGAAAGCCTTTCACACCTGCGGTACTCACCTGATGGTCATGATGAGTTTTTACCTGGTGGGCAGTGTGACATTCCTCTCCCACAACCTTAACCTACCCCTCCCTGTGGATGTGAACACCTGCTTAGGCTTGCTCTACATCATCTTCCCTGCCAGTGTCAACCCTGTCATCTACGGGGTTCGGACTAAAGAAATAAGGCACGCCATACTGAAAATTTTCAAGGTCCAGGCCAATAAAGTGTTTGTGGTACAA

**>Product of Internal PCR of CL16219.Contig1_All**

GCACAAGATTTATTTTCCATGACATATTAGGACTAAATGGCTTTTACAGCAATGGGGGATCACACAGTAGTTAACTACTGTTTTCAAACGCTAAACAACTCCTGCACAAAGGATATCAGAGCACAAGGGGAGTACATAATTCTGTACATACTCTTGTTCCTGATATCGGCATCAACTGTGTTTCTGAATCTTTTAGTGGTCATTGCTGTTTCTCACTTCAAGCAGCTTCATACTCCAACCAACCTGCTCCTTCTCTCTCTGGCTGTGGCAGACTTGCTTGTGGGCTTCCTGGTAATGCCTGTGGAGGGCATGAGATTAATTGAAAGGTGTTGGTATTTTGGAGACACATTCTGCTACATATTTCCACTAATTTTGTTTGTTGTTATTTCAGCCTCTTTGGGAAATCTGGTTTTTATATCTGTTGATCGTTACATTGCTGTTTCCAATGCTTTGAACTATTATTCATACATAACACTTAACAAGGCAGTGCTTTGCATATTTTTTAGCTGGTTTGGTTCTTTTGTTTATTCGGTGCTGATACTGTCCAATCATCTTCTTCAACCTGAGCCACACAGGACTTGCCATGGAGAGTGTCTGCTTGTAATCAATTTTTCATGGATTATTGCTGACCTTTTTGTTTCATTTACAGTGCCATGTTCCATTGTAATATGTCTATACCTTAAAATATTTAGTATGGCTAAACACCAGACATTAGCAATCAACTCTGTTAGAAATCCTGGCATGACCACAAATGAAAATGTCAAAATACGTAAATCTGTCAACAAAGCAGCAAGAACATTAGGAATTCTTGTGGCTGTTTATTTGCTCTGTTGGATTCCTTACTATGTAAGCATTCTTGCTAACGGTAGCATTTCATCATCATCTTTTATAGTGACTTTCCTCAGCTGGACTATGTACATGAACTCTTGCATGAACCCACTGATTTATGCACTGTTTTATCCATGGTTTAGAGTGTCAATTAAGCGCATTTTAACACTGGGCATACTCGACCCAACTTCACCATATTACAGTGTCTATCCAGATGATGTATAACTTTGAAAAGAATCACAGTTATACTTTGGATGTATTTATATGTTCCCTTCACTGTTTACCAGTA

**>Product of Internal PCR of Unigene101623_All**

AATGGCCTGGAGGATTTTGACATACGATTGGCTCTCAGCTTCTGCATAATAATAGCATAACAAACGACTATAACTACCAAAGGGACAGCAAGTCCTACTACAAAACCAGTCACAATCAACGTTTGCAGCGACACTTTAGCAACGATGTCATTATAAACACAGATGGTCCTGCCAGGTTGCTCCAGTGTGGTAGAAAAGACAAGGGTGGGAAGAGATATCAGTGCTGAAAGAGTCCAGGCGACAGCAATGACGACAGAGGACCCTCTCAAAGTACGGTGGTTCTGAGACCACACAGGAGCGATGACACATAAGCAGCGGTCTATACTGACGATGACCAGAAGGAATACGCTGC

**>Product of Internal PCR of Unigene3763_All**

TAGAGTTAGCCAGAAGAAGGGTCACTGTGTTGTCTACCACATGACCGTGTGATTCATCCAGTGTATTTGTCAGGAGCATGGAGACATTGTAAGGTATTAAACATATTAGAAAAACTGAAACAAGAATCCCAAGCACTCTGGCAGCTTTTCCCTCAGATGCCATAGAGTCAGCATTATTCTGGTCTTCCAGTCTTTTCCTGTTTTTGAATGTTGCTCTGATTACAACTGCATGCCTTCTAGCAATTAGGAATACTTTGAAGTACAAGATCACAATGGCAT
