# Supplementary material for: Identification of olfactory receptor genes in the Japanese grenadier anchovy Coilia nasus
Source: Genes Genomics. 2017 Feb 23;39(5):521–32. doi: 10.1007/s13258-017-0517-8 (PMC5387026; doi:10.1007/s13258-017-0517-8)
Supplement: Supplementary file 2 — Supplementary Text 2. Primers used in RT-PCR. (DOCX 16 KB) [file 13258_2017_517_MOESM2_ESM.docx]

Table. Primers used in semi-quantitative analysis.

| Gene Name | Sequence |  | Tm (ºC) |
| --- | --- | --- | --- |
| CL1147.Contig4 | 5’-CTGGTTTACACAACCCGTACCA -3’ | 5’-GTTATCAAGACACCAAAGAGGGAGA -3 | 60 |
| Unigene71950 | 5’- TCTGTTTCCCCTCCATGTTGT -3’ | 5’- TTGGGCTTTGGTTTATCTCCTC -3’ | 63 |
| Unigene79724 | 5’- GGCACAGAGGGCAGAAAGA -3’ | 5’- TCGGTGTAGGACAGGAACTCAA -3’ | 68 |
| Unigene8764 | 5’- TCTCAGCGACAAAGACAGGTTC -3’ | 5’- CTACTTCTAACGGCTCCAATCCA -3’ | 68 |
| Unigene14297 | 5' CAGTGTCTGCTCTTATCGGTTTAG 3' | 5' GCACTTGAAACATATCACTAGGCA 3' | 64 |
| Unigene61719 | 5' CACTAATGACTTTGGCGAGGAA 3' | 5' CAGTTTGTTGCGAGTGGTTTC 3' | 63 |
| Unigene24070 | 5' TCTGGCTCGTAGATTGCCTGAT 3' | 5' GCAAAAGAGCACCCCAAAACTA 3' | 60 |
| Unigene65091 | 5' GCTCAGCAAACAAAACATAACG 3' | 5' GCAAGCATACAAAGTCTCCCAC 3' | 60 |
| Unigene10816 | 5' GTAATGGGAAAAACATCCACTGAC 3' | 5' CAGGCTGTGGTTTTAGAAATGTTAT 3' | 60 |
| Unigene66147 | 5' GACAACATGTGATGGGCAAG 3' | 5' TACAAGCCCATCTTCTTTTCAG 3' | 56 |
| Unigene28241 | 5' CCTGCCCTGAGCAATGTTT 3' | 5' GCATCCCTTGCTCAAATCTTAA 3' | 60 |
| Unigene61739 | 5' AGATAAAGATGGGCACGGCTC 3' | 5' CGCTAATGATCTTGGCTGTCC 3' | 65 |
| Unigene54792 | 5' CTTTGCTCTCTGTCTGTCCTGTG 3' | 5' GTCTCTGAAAGGGCAGTGAACA 3' | 59 |
| Unigene86482 | 5' CGTGCCGCTCTTATGCTGTT 3' | 5' TTGACTGTGATGACCCCGACT 3' | 60 |
| Unigene15989 | 5' GCCATCGCTGTGGAAATCT 3' | 5' GAGCAAATATGCAAAGGAGGAG 3' | 57 |
| Unigene34891 | 5' GGCTGTTTCGTTCAGTTTTTTC 3' | 5' GTACTGCAAAGGCTTAAATATGGAG 3' | 60 |
| Unigene18154 | 5' ATCATCCAGTGAGGGTAGAGCA 3' | 5' GGATTGAAAAGTGGTGGAAGAA 3' | 60 |
| GAPDH-F | AGCTTGCCACCCTCTTGCT | AGCCATCAACGACCCCTTC | 58 |
