# Supplementary material for: Identification of olfactory receptor genes in the Japanese grenadier anchovy Coilia nasus
Source: Genes Genomics. 2017 Feb 23;39(5):521–32. doi: 10.1007/s13258-017-0517-8 (PMC5387026; doi:10.1007/s13258-017-0517-8)
Supplement: Supplementary file 3 — Nucleotide sequences of identified OR genes in Coilia nasus. (DOCX 125 KB) [file 13258_2017_517_MOESM3_ESM.docx]

>CL1061.Contig2_All CCTCAGTCAGACAGGTGACATTGAAGACTGGGCGCAGTAAGAAATATGAATTCTTTCAGA

TTATTTTTTATATTGAATGCAACGCCTGGACATTAACTTCACATGAGAGGAGACATTTTT

TAACAAGGACAACATCATCAACAGTTTTTTTATATTAAGGGCTATGGAAAATTCATCTTT

TCACAAGTTTTTTATTCTAAATGGACTTCAACAAGCAGGAAAAAATAAGTCTGTGTACTT

TTATTTGACATGTACACTGTATATTCTCATCATTGCCATGAATGTAACAATTATTATCAC

AGTCAGGGTAGATAAAGCCCTTCATGAACCGATGTACATATTTCTGTCAAATCTGTGTGT

TAATGGGTTGTATGGAACTGTTGGCTTCTACCCTAAATTTCTAATAGATCTGCTCTCTGA

GGTAGCAGTAATTTCATACCTACAATGTTTAACACAAACAATTGTAATTCATTCGTCTGC

CATATGTGAAATGACAACTTTAACAGCAATGGCTTTTGACAGGTATGTAGCGATATGCAG

ACCACTACAATACCACAGTATTTTGACGTCTCACATGGTCTTGAAGTTATTGTTACTCGC

TTGGTTTTATCCTGCATCAGTGTCGGTTGTGACTATTGTTCTTACTGTCAGAGTACCCAT

TTGTGGATCACTTATCAGTAAACTATTTTGTGACATTCCTTCAATTCTAAAGCAAGGCTG

TTATCCAACACCAGTGAATCGCATCTTTGGTATCTTTGTATTGATATTTCAAGTCATACA

GGTGCTGTTAATTTGTGTGTCATATGTTCAGATTGTTCGTGTTTGTTTACGTTCCAGGGC

CGGCAGGAGAAGGTTCACTCAAACCTGTTTGCCTCATATAATAGCCCTTTTTGTCTCCAT

CTTGTCTCTGTTGTTTGATCTTTCCTGTAGCTGGGCTGACACCACAAACCTTTCTCGCGA

TCTGAGAAATGTATTAGGCATGCAGTTTCTGATTCTCCCTCCAATCTGTAATCCACTTGT

GTATGGTCTGCAACTCCCACAGATACGTAGAGCAATTCTCAAAACATACTTAAAACCTAA

GGTCAGTAAAAATAAACGCAGTAATAGAAGATGTCATTAAAGAAATTAGTTAAATATACA

GTGCACATTTTTTTTCTGAGTAATGCAATGAATGTAAATATTTCCTGATGATCATTTATA

TTTTACCACAAAAATATTTCAAACAAAAGAGGAAGTCTCCCTCAGACTGTGAAAGGGCTG

AATATACAACACAAAAGATTCCACTAAAATGTATTTATATTTCATGAATGTATACTTTGT

TCTACATTTCCACAATTTACATAAATTTGCTTACAACTTTGTTAGTGATTTTATTTTCCA

GTGTGTAAACCATATTGTGTTTGTTACTTTTACAATCAACTGAAAAGCTGTATATCTGTA

GTCTTCATTACACTGTGTTTTGTGCCATATTATCATTTTTCCCCATGTTAAAATGACCAG

CAGGTAATCCTTAATTAAAAAAAGACCAGCACCTAAG

>CL10694.Contig1_All

CCTGTTGGTACTGTACAGTAGGTGGACATCAAACAAGTATGGAAAACAGCTCATCCGTCA

GTGACATCCTTGTACTGGAAGAGTTGGGGCACCCTGAGTCGACCACATACGCTGTGTTCT

TCACACTGCTCTTTGTCTACATTGCACTGCTAATAACTAACTTTGGTGTACTTATTATCA

TCATTGCAGAGAAGAGCTTGCACCAACCCATGTACTTGCTATTTTGTAACTTGCCAGTCA

ATGATATCCTTGGTAACACAATACTTCTACCCCATCTACTGTACGACATGGCTTCTAGAA

ACAGGTTAATGTTTTATAATAACTGTGTTGCACAAGTCTTTTTTACCCATGTTTACGCCT

CAGCATCACACAGCATACTAGTTGTTATGGCCATTGATAGGTATGTGGCTATATGCAAAC

CTCTGCGATACAATGCAATAATGACAGCTAAGGCAGTTCTGGTCTTGTCTGTGTCTGCCT

GGGCGTTTCCTGCTGTGTTTATAAGTGTACTAGTTGGCCTCAGTGTCAGGTTGTCTCGCT

GTAGATCTACTATTCCAAATTTCTACTGTGACAATGCATCTTTGTTCAAGTTATCCTGTG

AAAATGTGTCTATTAATAATATATATGGGCTGTTTTACAGTGTGCTTCTATTAGGCTCAT

CAATGGGTACAATTGCTGTCACTTATATTAGCATTGCAGTCACATGCTGGACTAAGAAAA

GTGCAGAACTTAACAGCAAAGCCATTCAAACCTGTGCAAGCCATTTAGTGTTGTACCTGA

TAATGCTACTGACTGGATATGTTGTGATTATCATGCATCGGTTCCCAGAACACCGTTTTT

TAAGAAAACTAATGGCTGTTCTTTTTCATGTTGTACCTGCTCATTTAAATCCAGTCATTT

ATGCTTTCCAGACTAAACATTTAAGGGTGAAAATATTGCAAATATTTGGTAGAAAAATTA

CACACAGAAATTTCTAGATATGTTTGGAGTCAGTTTTAGTCAATCATCTTTACACACACA

TTAAAA

>CL10694.Contig2_All ACACATATGGCTCAGCTTCACACACCATACTAATCGTAATGGCCATTGATAGGTATGTGG

CTATATGCAATCCATTGCGATACAGTGCAATAATGACCACAAAGGCTGTTTTGACTTTGT

CGTTTTTTGCATGGGCTGTTTCAGTTGTATTAGTGGGTGTGTTACTTAGCCTCACAATAA

GGCTATCGCGATGTAGGTCACACATTATGCATGCTTTCTGTGACAATGCATCCTTGTTCA

AGCTGTCTTGTGAGGATGTGTCCATTAATAATATATATGGGCTGTTTTACAGTGTGCTTC

TATTAGGCTCATCAATGGGTACAATTGCTGTCACTTATATTAGCATTGCAGTCACATGCT

GGACTAAGAAAAGTGCAGAACTTAACAGCAAAGCCATTCAAACCTGTGCAAGCCATTTAG

TGTTGTACCTGATAATGCTACTGACTGGATATGTTGTGATTATCATGCATCGGTTCCCAG

AACACCGTTTTTTAAGAAAACTAATGGCTGTTCTTTTTCATGTTGTACCTGCTCATTTAA

ATCCAGTCATTTATGCTTTCCAGACTAAACATTTAAGGGTGAAAATATTGCAAATATTTG

GTAGAAAAATTACACACAGAAATTTCTAGATATGTTTGGAGTCAGTTTTAGTCAATCATC

TTTACACACACATTAAAA

>CL10694.Contig3_All CCTGTTGGTACTGTACAGTAGGTGGACATCAAACAAGTATGGAAAACAGCTCATCCGTCA

GTGACATCCTTGTACTGGAAGAGTTGGGGCACCCTGAGTCGACCACATACGCTGTGTTCT

TCACACTGCTCTTTGTCTACATTGCACTGCTAATAACTAACTTTGGTGTACTTATTATCA

TCATTGCAGAGAAGAGCTTGCACCAACCCATGTACTTGCTATTTTGTAACTTGCCAGTCA

ATGATATCCTTGGTAACACAATACTTCTACCCCATCTACTGTACGACATGGCTTCTAGAA

ACAGGTTAATGTTTTATAATAACTGTGTTGCACAAGTCTTTTTTACCCATGTTTACGCCT

CAGCATCACACAGCATACTAGTTGTTATGGCCATTGATAGGTATGTGGCTATATGCAAAC

CTCTGCGATACAATGCAATAATGACAGCTAAGGCAGTTCTGGTCTTGTCTGTGTCTGCCT

GGGCGTTTCCTGCTGTGTTTATAAGTGTACTAGTTGGCCTCAGTGTCAGGTTGTCTCGCT

GTAGATCTACTATTCCAAATTTCTACTGTGACAATGCATCTTTGTTCAAGTTATCCTGTG

AAAATGTGTCTATTAATAATATATATGGGCTGTTTTACAGTGTGCTTCTATTAGGCTCAT

CAATGGGTACAATTGCTGTCACTTATATTAGCATTGCAGTCACATGCTGGACTAAGAAAA

GTGCAGAACTTAACAGCAAAGCCATTCAAACCTGTGCAAGTCACTTAGTCTTGTACCTGA

TATTGCTACTGATGGGCTACCTTATAATTATTATGCATCGGTTCCCAGAAGAACGATTTA

TAAGAAAACTAATGGCTGTTCTAATTCATATTGTACCTGGCCATTTTAATCCAATTATTT

ATGGACTCCAGACCAAACACTTAAAGCTAAAAATCTGGAAAATATTTAGGCCAAAAACCA

CAGAGACATAGAGGTTATTACAAGATATGTTTAAAAACAGCTTTAATTCAATGGAAACAG

AAAGTCCTTACATGAATTAAATATGTAATTGTAAAACTGCA

>CL10694.Contig4_All

ATTCCTACCCGTTTCTGGGATAGTTTATGAAGACATCAAACAAGGATGGAAAACATTTCA

TCCGTCAGTAACATCCTTGTACTGGAAGAGTTGGGGCACCCCGAGTCGACCACATACGCT

GTGTTCTTCACACTGCTCTTTGTCTACATTGCACTGCTAATAACTAACTTTGGTGTACTT

ATTATCATCATTGCAGAGAAGAGCTTGCACCAACCCATGTACTTGCTATTTTGTAACTTG

CCAGTCAATGATATCCTTGGTAACACAATACTTCTACCCC

>CL13258.Contig2_All

TATGACAGATTTGTCTCCATATTTAGGCCCCTGCAGTACCACACCATCATAACACCATGG

AAAGTAAAGCAGTTGATGATAGTTGCAAACGTGATCCCGACAGCTTTAATACTTGTTCAA

ATATTCATGACTTCCCAACTGCCTCAGTGCAGGTACGATGTTCATAGGACATATTGTGAT

AATTTAGCTGTTGTTAGTTTATCGTGTCTTGAGAGTCACACCATTCAAAGGCGAGT

>CL6601.Contig1_All

CGTCAAATCGGTAAGCCTGTATAATCTGCCCTCTTCAAGGGCCTGCTACTGTTGAGCCTC

CCCACTGCATATTCTTGTGGGAATGCCTCTCTAAGTCAAGTTGTAATTTGCTGTGTTAAT

CTGCACAAAAAATGTTTCCCAATGGTAAAAGGTATAAATGACTTGCATATCAAATGTCTG

TCTTCACAATGCAACAGATGGTAGCCAGTGTATATCTGTGTGACATCCAAACATGAAAAA

AACTTGGACAGAAAAGTACCAGTTTGAGATGGTAACATTGCACTGTGTAATTTGTATGTG

GATGTGTTTAAGTGCTAAACTATATATAATAGTCAATAGCATTTCAGTATATATAAAATG

TAATGATGGAAAACATGTCAGCTTTTAAATTGTTTGTTCTTTCTGGACTGCAGGAATCAG

GAGTTTATAAGCCTCTGTATTTCTTGTTGACATTAATTCTGTATGCCCTTATTATTGCAG

CCAATTTAACTCTGATCTTAAATGTGACTATGGAAAAAAGGCTCCATGAGCCAATGTATA

TTTTCCTGTCTAATCTTTGTGTGAATGGACTTTATGGGACTCTTGGTTTTTATCCCAAAT

TTTTGCTGGACCTGCAATCTGATGTTCACACAATAACCTATGGTTGGTGCATATTACAAG

TTTATGTGATATATACCTCTGTCATGTGTGAAATCACAATTTTAACAGTAATGTCTTATG

ACCGATATGTGGCAATCTGTAAACCTTTGCAGTATCACAGTATTTTGACTCCACATGCTA

TTCTGAAATTGCTTTTATTGGCCTGGGCCTATCCTTTATTGACATCAGTGGTGGCTATCT

TGTTAACTGTTAGAATACCCATTTGTGGCTCTCTCATCCATAAATTATTTTGTGACAATC

CTTCAGTTCTAAAGTTGGGGTGTTCTCAAAGCATAATAAACCAGATATGGAGTATAATAG

TAATAACTGGTCAACTTATGCAATATGTTTTTATTTTTATTTCCTATTGTCATATTATCC

ATGTTTGTATATCATCCAGTGAGGGCAGAGCAAAGTTCACAAAAACTTGTGTTCCTCATA

TACTGGTTATGTTAATCTTTGTCACAACAACACTGTTTGATGTGTTATACAGCTGGGGTG

AGTCCAAACATTTTCCAATTTGGATACGTAATGCATTGGCTGTACAATTTCTGATTCTTC

CCCCACTATTGAATCCAATAATATATGGTTTTCAACTCCCACAGATACGGAAAATACTGT

GCAGACAAAGTTGTAAACACAAAATGATGTGTTAACTTTAAGATTATTGTTTTACTGATT

AGGCATTATGCACCACTGCAAAACCTGTTTTTTGTCATATTTGTTGTGACAGCAATGTTT

GATACCATTCACAGCTGCAATGGCTCTATAGATTCAATTATATAATTTACTAATTTTTGT

TGTTTAGCCATCACTGATGGTCTCGAACTAATATATATATTTAACACTACAGGATGGAGA

CAACTTTAATCTTTACATTATATCATGAGATATAAGCAACAGTTTATAATGTTCTGGGTT

CGGTGAATATATCAGTGATTATATATCAAAATTATATGTTGTTGAAAATTAAGAACTCAT

CAAATTAGATAAAGGTGTTATGTCTTAGTTATAAAATGTATACAGAAATAGATATCTGTA

CTAAGAAGATGTGAATGCTAAATATCGTTACAGCGATAGCATACAGCATATAAAACATTG

TACATATGCTATCAATAACAGACTATATAAAACTTAATTTGCATGCATTACGTTCCATGA

AAAGGAAGCACATCACTTTTTGGTGTGAAACTTCTAATGTCACTGGAAAGTTATCAGCAA

AGAGGGGCGATAGTCGGGGCTTAGCTTCTCCATGTCTGTTGAGCAATGCATTCTGTAATT

TGCAGACATCTCATGTTCTTTTAAAGAATAATAAATATTTAAATAGAAAAATAATGAAAC

AGCATTACACCCTGTAACCAATAAACAACTATAATTAATACATCACAAAGTAGGTGTTGG

TCAACAACAAGAGCAGTCCTGTTATTATGCCATGCGTTTGTCTTTCAAATCAGCCTCCAC

GAAAGTTTGGACTGCAGTCTTTTACTGCTAAACCAGAGACTACAGACTATAGTACTCATC

TCATGAACAAATGTTAGATGGGGACAGGCACGGCTCATCAATATGGGCAGTGCCCTCCCA

AAAAACA

>CL6606.Contig4_All TCTGGATGTAACAACATGTAATATTGTGTACTTACTCTGTACATACCTAACACTAACTCT

AATCCCAACCCTTAAGTACAGTGTAAGTACACAATGTTATATATTGTTACACTATACCTA

TTTGATGTAACAGAAACAGTTAACTAAGTGTAACTGCAATTTCTTTTAAAAGACATTTAT

TTAATATAAATTAACATATTTTATTTTAGGTTGAAGATTCATGAGAATCCATCAGCAATG

TCTCTCAATTCATCAGGTGCAACAACTATGCTAACTTTACCTCCATTCTCCTTTCCTTTG

TCAGCCAGAGTTCCAGCTCTTGTTTTTTCAGCCCTTACATATCTGACCATTATTTGTTGC

AATCTGATGGTTGTTGTGACTATTGCTATTAACCATAACCTGCACAAGCCCATGTATATG

CTACTTATTAATTTACCTGTGTGTGATATGATGGGAGCGACAGCCTTTTTCCCTCAGATG

ATTTCCAGCATGCTGTATGACCCCAGAGTTATCCCATATTGGGCTTGTGTAACACAGGCT

TTATTTGTGCACACGTATGGTGCTGGATCACTTCTCATATTGACAGCCATGGCCCTTGAC

CGCTATGTAGCTATCTGCCAACCGCTTTCTTACAACACCATAATGACTAATAGTAATCTG

ATTAAGATTATAAGTGCTGTTTGGCTTACAGCCATGATTTTGATTTTTATCCTCATCGTG

TTGGCACTCCGTTTCAAGATTTGCAGGACCAATATCAGTGACATGTATTGTAACAACCCC

TCGTTGACAAACCTGGCCTGTGAATTGACATACGTGAATAACGTCTATGGTTTGCTTACT

ATTGCCTTTTTTCAGGGAATATCACTGTGTGCAATTCTGTTCACATATTTACAAATCTTA

ATAACTTGTGTTTTCAAGAAACAGTCAGATGCCAGAAGTAAAGCGATTCAGACATGTGGT

GC

>Unigene115271_All CGACGCGCTGCAGCTGAGCCTGGTGACGGCGCTGTACGTGGTGAGCTACGCCTTCTCGCG

CATCCACGCCTCGGTGTGCAGCGTGCTCATCATCACGGCCGTCACCACCACCCGCGCCAC

CCCGCTCATCCTGGCCGGCATGGCCGTGGAGCGCTACATCGCCATCTGCTTCCCGCTGCA

CTACGGCCACATGTGCACGCTGGCCCGCACGCTCTGGCTCATTCTGGGCATCCTGG

>Unigene16290_All CCATTAAAACCTGTGCAAGCCATTTAGTGTTGTACCTGATAATGCTACTGACTGGATATA

TTGTGATTGTCATGCATCGGTTCCCAGAACACACATTTTTGAGGAAACTTATTGCTGTTC

TTTTTCATGTTGTACCTGCCCATTTAAATCCTATTATTTATGGCCTACAGAATAAACAAT

TAAGGCTGAAAATTTTGCAAATATTTGGTCAAATCATACCTTGTTAAAAAGTTGTCGTTA

CACTTGTCTCCACAATGAAGATGTAAAGTGTGTGTTACTAAATATAAGGGGACATCCATT

TAGGTGATACCATGGAAAAGTTGGGCATTATATCCTTGTAATATTTGTCTTGACTCAGCT

TCTGCTGACATTATGCATCTTTACAATGAGTAAAAACAAAGGCAACAATATGTACATTCT

CTTCAAAAACATGTAGCGAATCCTGGAGCAAAAGTTATTTATTTTTCTTCTTTTAGACTG

TATGCTTTGGTTGTTCGTTTTTGTCTTCAACTGTGTAATGTCCTCTGTAATTGCAGGCCT

ACTTATAATTACAAATGTAAAATAAACTTATTAAAAATAAAATTAACCATACATTCACCA

TGCACCTTGGGTAAGCTACTTAGCTAAGGGCATGGCTTAAACTCATTCCCAAACCAAGAT

ACTAGAGAAAAAGATATAAC

>Unigene18154_All

TGGGCCTATCCTTTATTGACATCATTAATTGCTGTTATCCTTACTGTTAGAATACCTATT

TGTGGATCGCACATTCATAAACTATTTTGTGATAATCCTTCAATTCTAAAGCTGGGATGT

TTTCAAACCACAGTGAACAAAATATGGGGTATGATACTAATTACACTTCAGCTTAAGCAA

TTTGTTTTTATTTCAATTTCCTATTGTTATATTGTCCATGTTTGTGTATCATCCAGTGAG

GGTAGAGCAAAGTTCACTAAAACTTGTGTGCCCCATATTTTGGTTGTTGTAATCTTTATT

GCCACAACACTGTTTGATGTGTTGTATAGTTGGAATGGGTCTGTACATTTTCCAGTTATT

GTACGCAGTGCATTGGCTACACAGTTTTTGATTCTTCCACCACTTTTCAATCCAATAATC

TATGGGTTTCAGCTTCCACAGATCCGAAAAGTCTTGTGCAGACAAAGTTGTAATCACAAA

ATTATGTGCAATCGTTGAGTTAACCCTAATGGTTTTTCATCATCTTTGATTTGACCAAAA

TGTTGGAGCAGCTAGAATTATTATGAAGATCTTCCAGTTG

>Unigene21419_All

CACCCCCTGCACACTCTCAGAGGATCATATCGGAGCTGTCAGAAATGGTTTAGGATATAA

GCAGTTCTGCAGACAAGTCTCTGAGAAAACAGTGGTATTGTTACATGTTAAAGGTAGGAT

TTTATGTGTGATTCTCACACATAACTGATATAAAATGTGTTTTTACAAGTATTATAAGAG

AACTTTGCAGGATCTTTTTTTTTCACAGGTGAACAAGGACCAAAGATGAAAAACATTTCA

TCCATCAGTGACATTCTTGTGCTCGAAGAATTAGGGCATGCAGAATCATCCATATATCCT

ATATTTTTCACATTGCTCTTCCTCTACATCACATTGCTGATATCTAACTTTGGTGTTTTG

TCTATTATCATTGTGGAGAAAAGCTTACACCAACCCATGTACTTGCTGTTTTGCAATTTG

TCACTCAATGATGTCATTGGCAACACTATTCTGGTCCCCCATTTATTGTTTGACATGGTT

TCTAAAAACAGGCTGATTTCTTACAATAACTGTGTCACACAGGCATTTTTTGGCCACACA

TATGGCTCAGCTTCACACAGCATACTAATCATAATGGCTATTGACAGGTATGTGGCTATA

TGTAATCCACTGCGATACAGTGCAATAATGACAGCTAAGGCTGTTGTGACCTTGTCTGTG

TCTGCCTGGGCTGTTCCTGCTGTATTTGTCGGTGTACTAGTCAGCCTTAGTGCCAGGTTA

TCTCGCTGTAGATCACTAATTCAAAATTTCTACTGTGATAATGCATCTTTATTCAAGTTG

TCTTGTGAAGATGTAACCGTTAATAATATTTATGGGCTGTTTTACACTGTGGTGTTGTTA

TCCTCATCAATGGGTACAATTGCTGCCACATATATTAGAATTGCTGTCACATGCTGGACT

AAGAAAAATGCAGAGCTCAACAGCAAAGCCATTCAAACATGTGCAAGCCATTTAGTGTTG

TATCTGATAACACTGCTGACAGTGTACATTCTTGTCATTATGCAACGATTCCCAGACAAC

CCATTTTTAAGAAAATTCATCTCAATACTTTTTGTTGTTGTACCTGCCCATTTAAATCCA

GTTATTTACGGCCTACACACCAAACATTTAAGACTGAAAATTTTGCAACTAGTAGGTAGA

AAAATCACGCACAGTTAGTGGAGTTTGTGCTTTATGTTAAAGAAAACACATTATAATTGT

ATATTGTATATACACATGTATTGTTGTTTTGAAAATCAACCACTGAAAAGACTGCTGTAC

ATAAAATTCTGTACTAACACCATAATTTAGAAAAAGCAGTGTGATAACATCCCAAGGAGT

AGATTGCTGTTTAAAAGCGTAACATTTTTCAAGCTTGTAGCTAAGCTGAAGGAACAACGT

GTGTACTGTTACATCTTACACACTGCTGTAAAGTATACACTGTGTCAGCTAAACAATAAG

CCACGAAGGGTTGCTGTGGGTTTGGAACAGATTTAGAACAGCTAAGGAGTGTTGTTAGGC

ACAACGCAAGGTGGAGTGCACATAATACCGTTTCAACCAATTCTGAATCTGAACTATTAA

CTTATTTCTAATTTTTAGCAGAGTAGACACATGTTAGGATCATTGTAACATCAAACAATG

TTCTTTGAAAATGTTCTTTTCAGTTGTAATTATTATTGCAATATGATTACATTTACCATG

TCATTATAAAAGCAGGTTGATGCCAGAAGTACAAACAACCCCTGTAAAACAAGTAAATAA

ACATCAGTGGACAGTTGGATGGATATATTTCCACAAGAGAGATTTTGTGGGGAAAAAAAA

TAATGTGATCGTAACATGATGGCTATGGTGACATAAAATATTTAATTTTTCCACCTTAGA

TTAATATCTACCAGAGAGACCTGATATATCTCAAGAATGCCTTACGGCATGTACTTTACA

TTTAGTATGAACGTTTAGACAAAAAAGCAGAGCAACCTGACTTGAATTCCCACTGATTCC

CATTAGTCATTGTGATCACACAGCACACC

>Unigene21420_All

CACCCCCTGCACACTCTCAGAGGATCATATCGGAGCTGTCAGAAATGGTTTAGGATATAA

GCAGTTCTGCAGACAAGTCTCTGAGAAAACAGTGGTATTGTTACATGTTAAAGGTGAACA

AGGACCAAAGATGAAAAACATTTCATCCATCAGTGACATTCTTGTGCTCGAAGAATTAGG

GCATGCAGAATCATCCATATATCCTATATTTTTCACATTGCTCTTCCTCTACATCACATT

GCTGATATCTAACTTTGGTGTTTTGTCTATTATCATTGTGGAGAAAAGCTTACACCAACC

CATGTACTTGCTGTTTTGCAATTTGTCACTCAATGATGTCATTGGCAACACTATTCTGGT

CCCCCATTTATTGTTTGACATGGTTTCTAAAAACAGGCTGATTTCTTACAATAACTGTGT

CACACAGGCATTTTTTGGCCACACATATGGCTCAGCTTCACACAGCATACTAATCATAAT

GGCTATTGACAGGTATGTGGCTATATGTAATCCACTGCGATACAGTGCAATAATGACAGC

TAAGGCTGTTGTGACCTTGTCTGTGTCTGCCTGGGCTGTTCCTGCTGTATTTGTCGGTGT

ACTAGTCAGCCTTAGTGCCAGGTTATCTCGCTGTAGATCACTAATTCAAAATTTCTACTG

TGATAATGCATCTTTATTCAAGTTGTCTTGTGAAGATGTAACCGTTAATAATATTTATGG

GCTGTTTTACACTGTGGTGTTGTTATCCTCATCAATGGGTACAATTGCTGCCACATATAT

TAGAATTGCTGTCACATGCTGGACTAAGAAAAATGCAGAGCTCAACAGCAAAGCCATTCA

AACATGTGCAAGCCATTTAGTGTTGTATCTGATAACACTGCTGACAGTGTACATTCTTGT

CATTATGCAACGATTCCCAGACAACCCATTTTTAAGAAAATTCATCTCAATACTTTTTGT

TGTTGTACCTGCCCATTTAAATCCAGTTATTTACGGCCTACACACCAAACATTTAAGACT

GAAAATTTTGCAACTAGTAGGTAGAAAAATCACGCACAGTTAGTGGAGTTTGTGCTTTAT

GTTAAAGAAAACACATTATAATTGTATATTGTATATACACATGTATTGTTGTTTTGAAAA

TCAACCACTGAAAAGACTGCTGTACATAAAATTCTGTACTAACACCATAATTTAGAAAAA

GCAGTGTGATAACATCCCAAGGAGTAGATTGCTGTTTAAAAGCGTAACATTTTTCAAGCT

TGTAGCTAAGCTGAAGGAACAACGTGTGTACTGTTACATCTTACACACTGCTGTAAAGTA

TACACTGTGTCAGCTAAACAATAAGCCACGAAGGGTTGCTGTGGGTTTGGAACAGATTTA

GAACAGCTAAGGAGTGTTGTTAGGCACAACGCAAGGTGGAGTGCACATAATACCGTTTCA

ACCAATTCTGAATCTGAACTATTAACTTATTTCTAATTTTTAGCAGAGTAGACACATGTT

AGGATCATTGTAACATCAAACAATGTTCTTTGAAAATGTTCTTTTCAGTTGTAATTATTA

TTGCAATATGATTACATTTACCATGTCATTATAAAAGCAGGTTGATGCCAGAAGTACAAA

CAACCCCTGTAAAACAAGTAAATAAACATCAGTGGACAGTTGGATGGATATATTTCCACA

AGAGAGATTTTGTGGGGAAAAAAAATAATGTGATCGTAACATGATGGCTATGGTGACATA

AAATATTTAATTTTTCCACCTTAGATTAATATCTACCAGAGAGACCTGATATATCTCAAG

AATGCCTTACGGCATGTACTTTACATTTAGTATGAACGTTTAGACAAAAAAGCAGAGCAA

CCTGACTTGAATTCCCACTGATTCCCATTAGTCATTGTGATCACACAGCACACC

>Unigene23389_All TCTAACTCCTGCAAGTTCCTGTCCTCAGAAATAGAGGGCTATTTTTAAATGAGACTTTGT

CTAGTTGACAGCTGATTGCGACACTACTTCTCAGCAGGTAAAGGTTGCTCAGCAGAGAGG

AAAGGTTGATGATGGAAAACACATCCACTCTTCACTTTTTTATTCTTCATCGAATTCAGG

AATTGGGAAGAAACAAACTGTTGTATTTTTTCTTGACACTGGTTTTGTACATGGCAATTA

TTACACTTAATGTAACTCTAATTGTAACTATTGTGGTAGAAAAAAGTCTCCATGAGCCCA

TGTATATTTTCCTGTCTAATTTATGTGCCAATGGACTGTATGGCACTGTTGGTTTCTACC

CTAAATTTCTGATGGACTTGCTCTCTGACAATGCAGTGATTTCCTACGCAATGTGCGTCA

CTCAGACTTATATAATATATACATCAGCTATGTGTGAAATTGCTATTTTGACAGTGATGG

CTTATGACAGATATGTAGCCATATGCAGACCACTGCAGTATCACAGCATTCTTACTCCTA

AATCAATTTTGAGATTATTATTACTGGCTTGGTTTTATCCACTCTCAATGGCAGCTGTAT

CACTGATCCTCACGATACGATTACCTATCTGTGGCTCATACATTGGTAAGCTATTTTGTG

ACAATCCATCAATATTAAAGCAGGCATGTTATTCTGCTCATGCAAATCGCCTGTGGAGTA

TAATTATTATTGTTGGTCAAATTCTGCAGGCAGTTTTAGTTTTTGCGTCCTATGCTCAGA

TTGTACACATTTGTATGGGATCCAGTAAGGGCAGAACAAAGTTCACTCAAACCTGTTTAC

CACATATCCTGGCTATTTTCATATCCACCCTTGCTGTACTGTTTGATGTTTTATACAGTT

GGGATGGCTCAGCAGAGCTTCCTTTGCATGTACGTAATGCATTGGGGTTACAATTTTTAA

TTCTGCCTCCCTTCTGCAATCCACTCGTATACGGCCTGCAACTCCCTAGGATTCGAAAAA

CATTTTTTCAAAAACTCAGATGTTCCAGGACAAAAGTAGGGTGCAAAAGGCCTCCCTGAG

GGTAGATGTTTTGTATTTCTATTGAAATGTATGGTTACATTTTGAAATGTGTTTTAAAAC

AGCCTACTTGAAAGTAAAATGTTTAAATTGTGCTAAATTAAGGGGGTTCTCTTAGTAAAA

AATGAATATAGTATTCATTATCATGTTTTCATTGACTTACAAGATGCCCTTATATGCAGC

AGCTCATCTTCAGTAAAATCTACCTTACTGCGCCAGCATGTCTCTGTAGCAGCCAAGGCA

CACAGACGAAAACCCTGTCTGCCACCATCTTCCATGTCTGCTTACAATGCACCCTAAGCT

TCCCACTGCTAATACAGTTACACGTCCATCACTCATTCCAGGTGATTTAAACTGATACTT

TAAGAGCATCAAACAGCAAAACTGCGAAATGTCTAGCCTCAGTCTTGCCTCAACCTATCC

ACATTGGGATAAATGAGGACTACAAAACACAACATGCTTGTGTTTTCTTTTTACTTCATT

TTAGTAGTATTTTAGTAGAAGTATTCTTTCATTGATGCAGTCTTTCAAGTCAGAGGACCA

AGCATTTTACTACATATTGTACTTGTATTGTACCTGATGATTGCATGTGACAAATATAAT

TTAAATTTGCGTCTAAATCTGAATTTGAAATACCATTCACTACCCCGCAATATGTTTGAC

AGATGTTTTTAGGGACGAATGACATTGTCGTTGCCGATAAAATTCTTAAAAATACTTTAT

GCTTTATACTTTATACTAATACTTTACATTTGTCAATGACAGTTTTATTACAAATAACTA

GAGGTGGAGATCCTGGCTTTAAAAAAGTAATTAACTGATTCTAGTACACAGATCATCAGA

CAGGTAGGCCAGCTAATTAGTGTTAAGTGCACTGGAAGAAGGAATACATGGCAGGGTTTT

GGATTGACAACTCTGCCCCTGGTGGCCCTAGCAGCAGTGCCCCTGCTCATGGTAACTTGG

TGGCTGGAGTGTTAATGTAAGGTAATGAATCACTGAATATCTGTATTATGTATTATGAAG

GATGAAGTATGCTAGCACTCATACATTTATAGAGTGACAATTTAA

>Unigene52959_All ACACTGTATTGCTGCCCCGTTTGCTGGCTGACATGTTTGCGTCAAAGAAATTTACCACCT

ATGCTGAATGTGTCACACAGGCCTTTTGTAGTCACACATTTGGATCAGCTTCACACATGA

TACTAATCATAATGGCCTTTGACAGGTATATTGCTATATGTAACCCATTAAGATATGCGT

CAATAATGACAACAAAGACCATTGTAAAGTTGTCTGTGTCTGCCTGGGGCATTTCACTTG

TGCTGGTGGCTATTTTATTGGGTCTCACAATCAGGTTGTCTCGCTGTAGGTCGGTTATTC

TCAATGCGTACTGTGACAATGCATCTTTGTTTAAGTTATCTTGTGATGATGTGTCAATTA

ACAACCTCTATGGTCTAGTTTTCACCGCAGTACTTTTCGGCTCATCAATAGGAAGCATCC

TTGTGACATACTTGAGAATAGCAATAATATGCTGGACTAAAAAAAGCAAAGAGCTAAACG

GCAGAGCTTTACAAACGTGCACAAGTCACTTGTTAGTATACATGATTATGTTGTGGACAG

GGTTCCTTACTATAATTCTCCATCGTTTCCCAGATTACCCATATTTGAGGAAACTGGCAT

ACATACTGTTTCATGTTGTTCCTGCTAATTTGAATCCAATTATATATGGCATGCAAACAA

AAACTCTACGGAAGAAAATTCTGCAGACTTTTTTAAGAAAGGTGTCGTCTACATAGGCAC

TCCATTTTTTCAAAACTCTCCATCTGGTTAAAATTGTTGAATAACAGATTCTTCTAAAAC

CTGTGCTCACTGACTGACATAATTTGTGCACATAGAGACTAAATCATATTATATTTAAAT

GACACCAAACATTGTTTAGAAGATTTTCTAATCCTAACTGCTTACCAAGATTAACATTTT

TATTACTACTTATG

>Unigene59895_All GTTCCCAAAGACTGGCTGCATGTATGAGCTTTTTTTTAATGCTGATAGTCAGATCATATC

TAAACACAGGTTATCAGACTGCAAGGATGAGAAACATTTCATACATCAGTGATGTTCTTA

CCATGGAAGGATTAGAGATCGCTGAATCATCCACATATCCAGTGTTTTTCACATTACTCA

TTGTGTATATTGCACTGCTAATATCCAATATTGGTGTTCTTGCGGTCATCATTGCAGAGA

GAAAACTACACCAGCCTATGTACTTGCTGTTCTGCAATTTGTCTGTGAATGACATTTTAG

GCAATACAATCTTAATGCCCCGGGTACTGTTAGACATTGTTTCAAATGAGAAGCTGATTT

CTTATAGCGCATGTGTCACACAAGCATTTTTCAGCCACACATATGGCTCAGCTTCAC

>Unigene62329_All GCCAATGTACATATTTCTGTTTAATCTGACCTGTCTTGGACTGATCGGTAGCACTGCACT

TTGGCCCAAAGTGATGAGCAACCTTCTGTCTGACAAGCAGAGCAGCTCCTATGAGGCATG

TCTATGTCAGGTGTTCATAGTTAGTGTCTATGCCACTGGTACTTACTCTGTTCTAACTGT

GATGGCCTACGACCGCTATGTTTTTATAATCGACCCTTTGAGGTATCACACAATAATGAC

GCCACAGAAAGTGAAACAGCTGTTGACTGTGAGCAACCTTGTCCCACTATGCTCTGTGTC

TGGCCAGGTTTGGCTAACAGCACGAGTGCCTTTGTGTAGTCAAAACATCCATAAGTTTTA

CTGTGAAAACATTTCAGTTTCTAAACTATCATGTGTCACCAGTTCTCTGTATCGTGCAAG

TAGTCTTTACGGTGTTTCTGTTTTTGTGCTGTCAGTTGTGTTCATAGCATTTTTCGTGCT

TCTGTCATACATCAGAATAATTACTGTTAGTGTGAAAGCATCGAGTGATTCCCAGACGAA

GGCCATTTCTACCTGTGCTCCTCACCTGATCGTTTTCATTAATTTCTCCATGAGTTCTCT

CTTCTCCATCATTTACAACCAGTTTGCGTTTGTGTCGGAGGATATGCACATGTTCCTGTC

CTTCCAGTTCGCTCTGGGGCCCCCACTTTTACACCCAATCATTTACGGCATCAAGAACAC

AGACATCAGGAAGTGCATTGTTAAACATGTCAGACCAGTGGTGTCATATGTCACTTGTTA

CATCAAATCAAGCCACAAACTGTCACCAAACTTCGTGCAGTAATGGGTGATGAGCCGATG

TTAATCCCTTACTCTCTTTGTGTGTTGTAAAGTTTGCCAGTTGTGCTTGCATGAGTTTTG

ACGCTTGCTCAATGTAAGAATCTGGACGTTTTTGAAGAGGTTTTTGTTATTAAGCTTTTA

AAGTAAAATGTAATTCTGAATTCTTATAATAGGATTTATTCAGGATATTTA

>Unigene68575_All TCGCTGGAGCGCTACGTGGCCATCTTCTACCCGCTGCAGCGCCCCGCGGCCTGGCGCGCC

GACCGCATCTGGGTCGTCATCGTCTCCATGTGGGTGCTGAGCTGCGTGCAGCCCACGGCC

GAGTTCATCATGAGCAAGCCGCGCGGCGGCGCCCAAATGGACGTCCTCACCACGCCCGTG

CAGTGCAAGAGCGCCGTGCTGCACGTGGCGCCTGCAATCACGCTCTTCAAGGTGGTCCTG

AACGGGCTCTTCTTCGCCCTGGTGGCCGCCGTCATCCTCTTCACGTACGTGCGCATCCTG

CTGGGCACGCGCAG

>Unigene68576_All CACACTCCATGCCCCCCATCAATAGCACCTCTGTGGGCCTCTGTGCATTATGCAAAACCT

GACTAGCGCTGTCAACGCCACGTCAGACCGGAACCTGGCGGTCATCGTGAAAGTGTGCCT

GGTCATCCCCATCTTCAGCGTCTTCCTCTACTTCATCGTCCTCATGCTGCACACGTTCGC

CTCGCACCGCCACTTCCTGGAGAGCCCGCGCTACATCCTGTTCACCTACATGCTGGCCAA

CGACACGCTGCAGCTGCTCACCTCCGTGCTGCTCTTCCTGTTCGTCATGGCGCAGGTCAA

CTTCGCGCTGGTCTTCTGCGCGCCGCTGCTCTTCTTCTCCACAGCCACCTTCCTCAACAC

GCCGCTCGTCCTGG

>Unigene95217_All ACGCGAACTACACTGTAGTTTGTATGTGTGCTATATATATTACATTTAATCTGCATTTTG

TCATTAGCTTTAGTGTGGTTGTGCGTTGCCACATGTGAAGATTTTTTTCTGTGGTCATAC

ATTTCAGACTCTCTTTCATACAGATTATCATTTCATCATCCATGTTCATATTCTTTTTCA

GGTAAGAGGGAAAAATGAATCACTCGTTTGATATCACTGTCATTTTTACTGCTTATCAAT

CTATTGGGGTCCAGAAGTATGCCTATTTCACCATTATTGCAGCATTATACATTTCCTCTG

TATTTACCAACGTGTTCTTAATGCTTATTATCATCTGGGAGTCCAGACTTCATCAGCCAA

TGTACATATTTTTGTTTAATCTGACC

>Unigene96554_All GCCTCGGTGGCCAAGGCGCTGCACACGGTGCTCCTGCACGGCCTGCAGCTGCTGCTCAGC

ACCAGCGCCTTCACCATCCCCTTTACCGAGCACCTGATCGTGCTGCACGTGGGCTGGCTG

CGGGAGCACATGTCCTTCCTCAACTACGTGTCCTTCGTCATCGTGCCGCGCGTGCTCAGC

CCGCTCATCTACGGCCTGCGGGACGAGAGCCTGCGCAGGCACATGCAGCGCTCCCCACTC

TGCTGCGCCACCATCGCTGCCACCGCCAGGGGCC

>CL10461.Contig1_All CTTATTCTTACAGACACTTAAATACAACTTCACCCAACAATGTAATGTTAGTCGAGCCAT

TGCTTCTTGGAGTGTGCAACTAAGAGGAAGGTTTTCCTGCCCTTAACCATGGACAACACT

GTCTTAAATGAAACCTTTTCCTTTGAGCTAAAAATTGCCAGCTATGATATTCACCCCTCA

GCTGTCTACCCTATTTTCGCTATAGGCATGTTGATATACCTCATCTCTGTTGTCTCTAAT

CTGACCATCCTGCTGCTGATTGCTACTCAGAAGAGTCTCCACAAACCCATGTTTTACATC

CTCTTCAGCCTGCCACTGAATGACCTTGTTGGGATAACTGCACTGCTTCCTCGTGTGCTG

GTTGATATTGTAACCCAAGAACACACTGTTTATTACCCAACCTGTGTTATTCAGGCATTT

TTGATCCATATGTTTGGTGGTGGCACTCTTTTTGTATTGGCAGCTATGTCATTTGACCGG

TATCTTGCTATCTGCAAGCCACTAAGGTACCACTCCATCATGACTCCTTTAACGGTAGCA

GGGATATTGCTGTTGGCATGGGGTACAGACTTCATCTTAATACTTGTTCTTTTTCTCCTT

CAGAGTAGAATACGTAGATGCAGAAATGTCATACAGAATATGTACTGTAGTAATTTTGCA

TTATTGGGCCTGTCCTGTGGAGAGGACACTACAATCAATAATATATATGGGTTGTTCATA

ACAGCATTCATGCACATCATCACTATAACAATACAGTTGTTCTCATATGTCCAAATTCTA

CTAGCATGTGTCTTTCACAGACAATCTGATGCTAAAACAAAAGCTCTGAACACTTGCTTA

GCACAATTAATAGTATTCCTAATATTTGAATTTATTAGTTTCTTTACAATAACAACCTAT

CGGTTTCCAAATGTACCTTCAACTGTAAGAATGACCATTGGGATGATGACGTTTGTGATT

TCACCTTTTCTCAATCCTTTTATTTATGGAATGAAAACAAAAGATATTAGAATATCATTT

ATTCAGGTAGTGAGGAAAAGAAAGTTATCAACATGATACTGCCGACTATACAAGCTCTTT

GCATGGCCTAAGCAAGACAAGGCATACTACAGGTACCAAAGTATTCGGTATCCAAATGTA

TCTTCAACTGCAAGAATGCCCATTGCATGACATTGTTTATGCTTATGCCTTTTGTCAATC

CTATTATTTCTGGAATAAAAACAAAGGATATTAGAATCATATTTCTTCTCGTAGTAAGGA

TTTTTTTTTCACATAATACAGCCAGTCATACTTTACATGGGCCAAGCAAGACTTGCTGGA

TAAGAAAGGCAAGACAAGGGATGTTTATTTGTATCTGGTTGCTCGGTCTATTGTACATTT

CATACAGAGAGGCAGTTCAATGTGGGTTACAGAAATAAAAGCAAACAGTATGTAAAAGCA

TAAAAGATAAAAAAAAGTAGGGGAAACTGTGTACTCAGTGATCCAAATTGATCACGTTTT

TTTTTTTTTTTTTTTC

>CL10461.Contig2_All CTTATTCTTACAGACACTTAAATACAACTTCACCCAACAATGTAATGTTAGTCGAGCCAT

TGCTTCTTGGAGTGTGCAACTAAGAGGAAGGTAACACACTACTTTTACTGAAACATATGT

GTATATATTTTTTCTTGAAGAAGAAGACATTAAATGTTTTGAAATCTTGTGTATTGCAGG

TTTTCCTGCCCTTAACCATGGACAACACTGTCTTAAATGAAACCTTTTCCTTTGAGCTAA

AAATTGCCAGCTATGATATTCACCCCTCAGCTGTCTACCCTATTTTCGCTATAGGCATGT

TGATATACCTCATCTCTGTTGTCTCTAATCTGACCATCCTGCTGCTGATTGCTACTCAGA

AGAGTCTCCACAAACCCATGTTTTACATCCTCTTCAGCCTGCCACTGAATGACCTTGTTG

GGATAACTGCACTGCTTCCTCGTGTGCTGGTTGATATTGTAACCCAAGAACACACTGTTT

ATTACCCAACCTGTGTTATTCAGGCATTTTTGATCCATATGTTTGGTGGTGGCACTCTTT

TTGTATTGGCAGCTATGTCATTTGACCGGTATCTTGCTATCTGCAAGCCACTAAGGTACC

ACTCCATCATGACTCCTTTAACGGTAGCAGGGATATTGCTGTTGGCATGGGGTACAGACT

TCATCTTAATACTTGTTCTTTTTCTCCTTCAGAGTAGAATACGTAGATGCAGAAATGTCA

TACAGAATATGTACTGTAGTAATTTTGCATTATTGGGCCTGTCCTGTGGAGAGGACACTA

CAATCAATAATATATATGGGTTGTTCATAACAGCATTCATGCACATCATCACTATAACAA

TACAGTTGTTCTCATATGTCCAAATTCTACTAGCATGTGTCTTTCACAGACAATCTGATG

CTAAAACAAAAGCTCTGAACACTTGCTTAGCACAATTAATAGTATTCCTAATATTTGAAT

TTATTAGTTTCTTTACAATAACAACCTATCGGTTTCCAAATGTACCTTCAACTGTAAGAA

TGACCATTGGGATGATGACGTTTGTGATTTCACCTTTTCTCAATCCTTTTATTTATGGAA

TGAAAACAAAAGATATTAGAATATCATTTATTCAGGTAGTGAGGAAAAGAAAGTTATCAA

CATGATACTGCCGACTATACAAGCTCTTTGCATGGCCTAAGCAAGACAAGGCATACTACA

TGTCCCAAAGTATTCGGTATCCAAATGTATCTTCAACTGCAAGAATGGCCATTGTTTATG

CTTATGCCTTTTGTCAATCCTATTATTTCTGGAATAAAAACAAAGGATATTAGAATCATA

TTTCTTCTCGTAGTAAGGATTTTTTTTTCACATAATACAGCCAGTCATACTTTACATGGG

CCAAGCAAGACTTGCTGGATAAGAAAGGCAAGGCAAGGGATGTTTATTTGTATCTGGTTG

CTCGGTCTATTGTACATTTCATACAGAGAGGCAGTTCAATGTGGGTTACAGAAATAAAAG

CAAACAGTATGTAAAAGCATAAAATATAAAAAAGTAGGG

>CL1076.Contig1_All CACATGCTCTGCAACGACTCCATCATGATCCTCTTCACTTTCCTCATCGGCATCATCTCC

TATGTCCACAGGCCGACCAAAGCCATGTGCTCGCTCATAATGATCGTCACAACCTCCACT

TCCACCAACGCACCGCTCAACCTGGGTGTCATGTCACTGGAGCGCTACATTGCCATCTGC

TTTCCTCTGCACCACAGAGAACTTGCCACCACTAAGAGAACATATGTGGCAATCGCTGCC

ATTTGGTTCTTTGGGCTGGTGAACCCTGTGGTGGATTCATTTTATAACTCTGTGACTGAC

CGAGATTTTTTTACTGAAGAAATTTTATGTGGTAGTCAGACAATATTCAACACCTCACCA

TGGCAGGCATTGCTCTACCAGGCTCTCAATGGCCTGTACTATGTGACTGTGACACTGGTT

ATCCTCTTCAGCTACATCAACGTCATGCTCGTGGCTCGGTCTGTGTCCAGTGATGAGAAG

TCCACAAGGAAAGCACACAGGACTCTTCTGCTTCACCTGATCCAGCTGGTTCTCTGTCTC

AACATGTTGTTGTACATCAGTATAGTCCGTTTCCTTGCACTGGTTTTCAGTTTCGAAGTT

TACAAAGACGTTAGCTATGTCATCTTTCTGCTGGTCATCCTGCTGCCCAGATGTCTGAGT

CCCATCATCTACGGCTTAAGAGACAAGGCTGTTCGCAATTTGTTCATGTTTTACTTGAAA

TGTGGCTTTTGTAGAGCCAAGCCGAAAGTAAATATACAGTGTAATCACTAATATCCCTGA

ATTAAGGAAAGACATACAGACTTATATATAAAATATACTTACGTCTTAGATATTAGCTAA

AGCAGTCTAACCTTTGTACTTCTGCCATTAATTTATGTCAGTGATTACATGTGCTGGTTT

AGTCTGAGCACTGAATCCACAATAAAACATGCTAAATTGAATCTGATAAAGCATCAGTGG

CAACTGGTAATGAATGTGGTAAAGCGTAAAACTGCTGGAGTTTGTGATATGATGTCTGTC

TGGGCTCTGTCATACTGTACTAGCGCTTCTCAATCAGGGAGAACCCTAAGGCCTGTGCTG

CAGGTTTATAAGTCAGTGCCTCCTTACAGAACAGTAAGCGATAACATGTGTATTCACTAT

TAATACTGTATCTAAAACCTAAACAGATGTTTCTATACATCTCTGTGTTGTGAGCACCCC

GAATGACTTAATAAAGTGCATTTGCTCTAGACTATA

>CL1076.Contig2_All

CACATGCTCTGCAACGACTCCATCATGATCCTCTTCACTTTCCTCATCGGCATCATCTCC

TATGTCCACAGGCCGACCAAAGCCATGTGCTCGCTCATAATGATCGTCACAACCTCCACT

TCCACCAACGCACCGCTCAACCTGGGTGTCATGTCACTGGAGCGCTACATTGCCATCTGC

TTTCCTCTGCACCACAGAGAACTTGCCACCACTAAGAGAACATATGTGGCAATCGCTGCC

ATTTGGTTCTTTGGGCTGGTGAACCCTGTGGTGGATTCATTTTATAACTCTGTGACTGAC

CGAGATTTTTTTACTGAAGAAATTTTATGTGGTAGTCAGACAATATTCAACACCTCACCA

TGGCAGGCATTGCTCTACCAGGCTCTCAATGGCCTGTACTATGTGACTGTGACACTGGTT

ATCCTCTTCAGCTACATCAACGTCATGCTCGTGGCTCGGTCTGTGTCCAGTGATGAGAAG

TCCACAAGGAAAGCACACAGGACTCTTCTGCTTCACCTGATCCAGCTGGTTCTCTGTCTC

AACATGTTGTTGTACATCAGTATAGTCCGTTTCCTTGCACTGGTTTTCAGTTTCGAAGTT

TACAAAGACGTTAGCTATGTCATCTTTCTGCTGGTCATCCTGCTGCCCAGATGTCTGAGT

CCCATCATCTACGGCTTAAGAGACAAGGCTGTTCGCAATTTGTTCATGTTTTACTTGAAA

TGTGGCTTTTGTAGAGCCAAGCCGAAAGTAAATATACAGTGTAATCACTAATATCCCTGA

ATTAAGGAAAGACATACAGACTTATATATAAAATATACTTACGTCTCCCTGTGGTACCAA

TTGAAAATATCAGCTTGAGAAATGTGACAAGATTCACTGGTGATAATTATGGATGTACAT

AGAACATGGTGCTTTCCTTTACCCAAATGTAATCAATGTCTTAGATATTAGCTAAAGCAG

TCTAACCTTTGTACTTCTGCCATTAATTTATGTCAGTGATTACATGTGCTGGTTTAGTCT

GAGCACTGAATCCACAATAAAACATGCTAAATTGAATCTGATAAAGCATCAGTGGCAACT

GGTAATGAATGTGGTAAAGCGTAAAACTGCTGGAGTTTGTGATATGATGTCTGTCTGGGC

TCTGTCATACTGTACTAGCGCTTCTCAATCAGGGAGAACCCTAAGGCCTGTGCTGCAGGT

TTATAAGTCAGTGCCTCCTTACAGAACAGTAAGCGATAACATGTGTATTCACTATTAATA

CTGTATCTAAAACCTAAACAGATGTTTCTATACATCTCTGTGTTGTGAGCACCCCGAATG

ACTTAATAAAGTGCATTTGCTCTAGACTATA

>CL11354.Contig1_All GCCAGGGGTCTTTCTGCACTCAGACAAAACCATATGACCAAAGCTTCAAACCATGAAAGA

TAACCATGAAAGATATTACTTCCCAGGTGGTGAGGCTGAGCATTAGTGTATGAAATGGAC

AACTACAGACCAGGAAACAACACGGGCGATGTCTTTTACCTGACCGGATTCGCCAACCTC

AGGGAGCACCGGCGCCTGCTCTCCATTCCTTTCTTTTTTCTTTTGCTTTACATGATGGCA

GCGAATGCTGTTGTCACTTATGTCATTGCTAAACAGAGAAATCTGTACAAACCTATGTAT

GTGCTAATTGGTTCTCTTACACTTCTGAATTTCTTTTTCCCTCTATTATTTATCCCGAGG

ATGGTGGTTAGTTTTGCGTCAGGCAGAAATGACATCACGGCAGTAGAATGCCTCTTACAA

ATTTTTGTCATTCACCTGTGTGGTAGCTTCCAGACTAGTATTCTGCTTCAGATGGCCGTC

GATAGGTTTTTTGCCATTTGCTGGCCGTTACGTTACCACGACATTGTCAACCTGCGAAAC

TCCATACTGTTTACTGCTGCACTTGCCCTGCGCAACACGGTGACTATGGTCACAATGGTG

TGCTTGTTCATTCCGCTCACATTTTGCCAGTCAAATGTGATCTACCACTGCGCGTGTGTT

GTAACTCTGGCGTGTGGAACTTCGCTGCGTAGTTACATAGCAGGTGTCATGGCTTTCACT

ATCACCACCAGTGACTGCATTATCATTGCTGCGACTTACGTTGTTATTTTCATAGTCATA

TTTTGGAGTTCGTCAGGCGAATCCCGTCAGAAAGCCATACACACCTGTAGCACACACTTG

CTAGTGATTGGCATTTCATACCTGAGTGTTGTGTGTGCTTTTATTGGGTACAAAGTGAGT

GCTATCCCACCTGATATAAAGGTTGCATTGACCCTCTCATATCTCCTCATCCCAAGTTGT

TTTAGTCCAGTTATTTATGGCATTAGGACGAATGAGATTAAAGTTTATATGCTCAAAATG

CTGAAGTTAAATACAGTGGATTCTAAGAACACCTAGAAGGGGTACAGACACAACAAGGCA

AAGGTGCCATCAAAGATGCGCATTTAGTAGATGCATTCTATTCAAACTATTCAAATTCTG

TTGAAGTATTCAGAAAGCATTTTTTTTTAAGTCAGGGCTTCCTGACAAATTTATGGAAGC

CCTTTACACATTTATGACTTTTTTCCCCGTCTCCTGTATTTTAGAGATTCTTGGATGTTG

TCTGCTGTTTGTCTGGTTCAAAATGCCTGAATGTACACTGAAGTGTTTAAATACGCTCAT

GTAATAGCATTCAGTTATATTATCTTTTGAATCACTTATAGGTTAAGTACATTGCCACTG

TGTAACAGATATGTAACAATTCCTGGCAAACGTGATAAAACAAACACCTCATGCTTTGAC

ATAGGCTATGTGATCCCAAAATGACTATGGACCCAAAACAGAAATGTTAGTAACAAAATA

GAAACAAAATCAATTAAACCTTGAAAATCTTGTTTTTTTAATTACTTGACATGTATTCCT

TGTTTAGGCCTACCATGACTTCAGTAGGTGTGTTTATTTATCCCTGAAATTTTATCAGAC

ATTTTGGCATTTTATATGCATTCATAAGCTCAATAAAACATTGAATTAATGTAGTTTTTG

TATGGGGGTTTCAATACAACATCATCAACATATTTTGCATTATTTACAGAATCCACATTA

TATTGTATTTAATTATAACACCATATTCATATTGTACAGCTGCATTGTTCTGTATGTTGT

CTTAAGGTCCTATGACAAGTTAAAAAGTAATTTTA

>CL12962.Contig1_All GGAAGTTCCCCACACTACAAGAGGAATTTCTGATCTCTGAACACACACTGTCTCTGTCTC

ACTGGCACATAGTTGAGACATGCATGAAGTCCTACGAATCAGACTTGACACAGACTGGTG

TTAACTCTCTCAACACAAATGTACCAGGTGCAGCTGAATGAGGGCCCGCTGACTAAACTG

GTGGTGGCCATCATCATGTCGCTGCTGTTTATTTGGGTCAACTGCCTGCTGCTCTTCACG

CTGAGCCGCAAGCACATCTTCCGGGAGACGCCGCGCTACGTGCTCTTCTCCCACATGCTG

TGCAATGACTCCATCCAGCTGCTCTTCACCTCTCTGCTTTACATCTTTTCCATGTCCTAC

TCGCAAGTCCACCGCGGCGCCTGCTACCTCCTCCTCTTCGTCTCCAGCTCCACCTTCCTC

AACGCGCCGCTCAACCTGGCCCTCATGTCCCTCGAGCGCTATGCCGCCATCTGCTTCCCG

CTGCACCACGGCAACGTGGCCACACCCACAGCCACGGCCGTTGGCATCCTTACCATATGG

TTCCTGGGGGGCGTAAACGTAGTGATTGACATCTTGTACGCACTCACCAGAGATGGCACG

ACCCTCGGCAATCATATGTTCTGTACACGCGAGCGCATCTTCATGGCCGCGTGGCAGGCT

GACGCGTTCCAGGGATTTAACGGCTTCTACTTTGTGATCGTGACCCTGATCATCGTCTTC

ACCTACATCAGCGTGATGGTGGCTGCTCGCTCGGCCACTAGCAATAAAGAGTCAGCAAAG

AAAGCCCACCGGACGCTCCTGCTGCACCTCATCCAGCTGGGCCTGTGCCTGAACTCGTTT

CTGTATGGCATCATAGAGAGAGCACTAGCTATGACCAGCAGCAGTCGTTTG

>CL12962.Contig2_All GGAAGTTCCCCACACTACAAGAGGAATTTCTGATCTCTGAACACACACTGTCTCTGTCTC

ACTGGCACATAGTTGAGACATGCATGAAGTCCTACGAATCAGACTTGACACAGACTGGTG

TTAACTCTCTCAACACAAGTTAGTGTGTTTTTAGTGGGCTGAAGGGATCCTGAATTACCA

TAGTTTAAAGTGCGATGTGATAGCTCAGCTTAGAGTGCTCATTTATTGTGTATGCTGATG

TAAAGTTTGGATCTCCTCAGGCTGGAGTGTCTGTCAACCACCAGCACATTGTCTTTAATT

ATCTTTATTTGTACTGTATATTATTTAATGTTAGCTTGATGATGTACACCCTTATGCTTA

CTGGGAATCATTCCATTGTGATGACTTATTGTGATTAACCTGAGGTCACGCATGTATTGC

CTAATCTGTTATTTTTATTGATTTACTATCTTATTTGCCTTGATGTTTAACTTGATATTT

GCGTTGTATTAATTATACTGATACTTGTATTTGTATTAGTCTTAATATTCTATGGTCATG

GCCGACACAAACTCGTCCACTGAGCAGGTGCTCTTCGTCCACCAACAGATGTACCAGGTG

CAGCTGAATGAGGGCCCGCTGACTAAACTGGTGGTGGCCATCATCATGTCGCTGCTGTTT

ATTTGGGTCAACTGCCTGCTGCTCTTCACGCTGAGCCGCAAGCACATCTTCCGGGAGACG

CCGCGCTACGTGCTCTTCTCCCACATGCTGTGCAATGACTCCATCCAGCTGCTCTTCACC

TCTCTGCTTTACATCTTTTCCATGTCCTACTCGCAAGTCCACCGCGGCGCCTGCTACCTC

CTCCTCTTCGTCTCCAGCTCCACCTTCCTCAACGCGCCGCTCAACCTGGCCCTCATGTCC

CTCGAGCGCTATGCCGCCATCTGCTTCCCGCTGCACCACGGCAACGTGGCCACACCCACA

GCCACGGCCGTTGGCATCCTTACCATATGGTTCCTGGGGGGCGTAAACGTAGTGATTGAC

ATCTTGTACGCACTCACCAGAGATGGCACGACCCTCGGCAATCATATGTTCTGTACACGC

GAGCGCATCTTCATGGCCGCGTGGCAGGCTGACGCGTTCCAGGGATTTAACGGCTTCTAC

TTTGTGATCGTGACCCTGATCATCGTCTTCACCTACATCAGCGTGATGGTGGCTGCTCGC

TCGGCCACTAGCAATAAAGAGTCAGCAAAGAAAGCCCACCGGACGCTCCTGCTGCACCTC

ATCCAGCTGGGCCTGTGCCTGAACTCGTTTCTGTATGGCATCATAGAGAGAGCACTAGCT

ATGACCAGCAGCAGTCGTTTG

>CL13258.Contig1_All

GAAGAATGCATATTTTATTATCGTCTTCCTAGTTTACCTATCATCAATCATAGCCAATGT

TTTTCTTATGTGTTTGATTTATTTGGACTCTGCTCTACATAAGCCAATGTATATATTCCT

CTTCAGCCTCCTGGTAAATGGGTTGATCGGAAGCACAGCTATTTGGCCAAAGGTTATGAA

TATCCTTTCAACAAACAGCCCTCTTATTTCATGGGAAGGATGTATTGCTCAGGTTTTTTT

TGTATTAACATACGGAGCTTGCAACTACTCTCTGCTGGCAGTGATGGCTTACGACAGATT

TGTCTCCATATTTCAGCCTTTGCAATACCACACCATCATGACCCCACTGAAAGTAAAGCA

GCTAATGTTTGTTGCAAACTTAATGCCAACAGCTGTGATGCTTGGTCAAACATACATAGC

CTCACAGCTGCCTCTGTGCAGGTTTGAAGTGCATAAGATATTTTGTGACAATTTAGCTGT

TGTTAGCATGTCATGTAGTCGAAGTCAACTGAGCAACTTGTATGGTATAATTAATACTAT

TCTTTTTGTGGTTCTACCGGTGTTTTTTATTTTATTGTCATATGTGAAAATCATAGTATT

GAGCTTGAAAGCTTCAACAAATGCAAGAAAGAAAGTGTTTGAAACCTGCTCTCCACACAT

AATTACTTTTGTAAATTTCTTTTTGGTGTCCTTGTTCTCACTCGTCTATAACCGCTACTC

TTTCTTACCTGTAGAGGCAAACATAGTACAGTCAGTCAATTACATCCTTTTCCCTCCACT

GTTGCACCCAATTATATATGGAATAAAAACACAGGAAATTAGACAAAGTTTCTCAAAAGT

AATCAAAAGAATGCTTTTGCAGTGAAGTCAGTTTACTTCCAAGTTGGTTTTATCATCAGT

ATTGTTTTTCTGTCTGACAGTTTAACAATAGCACGTGAGCAGATTTCAGCTGTCTCAAAT

ATGTGTACTCCTTTCTAAAGCTGTGTTAAACTTTTGAACCGAGGATGTATTTTTTTTTTT

TTTTTTTT

>CL1434.Contig2_All TGTAACTATCCGGTTAAGCTACTAAGCCAATTTACTTATAATGTTTACCCCTACACATAT

GAATGTTTACGTAATGATCCAAATAATAGTGGTTGCATGCAGTTTGGCCTGTAAAGAATA

TGCCATAATGGTAAACTCTTTAGCACATTATGAAGCTTAACATAATAAATGTGAAACTTT

AATTTACATCCATGAAAATATAGAATGTCTTCTAATTTAACAACCAAACTGTTTGAGTTA

TGCTTCCTTTTGCCAGGAATTGAGCTGTGATGCAAAATCTGTCCAGAGTTTCAGTTTTTG

AACTTACTGGATTGAACTTTACATCAGAATACAAATATTTCCTCTTCTCTCTAACTTTAC

TGTGTTATCCTTTCATTTTGATGGTTAACATCACAGTAATCTATGTCATTGTGATAAATA

AGCATCTGTATGAACCAATGTACATATTCCTTTGCAATCTGTGTATGAATGCATTGTATG

GAACTCTTGGTTTCTATCCAAAGTTTTTGTATGATTTGTTGTCTGACCATCATGTCATAT

CCTATTTAGGATGCTTGGTCCAAGCATTTGTCATTTACTCATCAGCACTTTGTGAATTCT

CCACTTTGACTGTGATGGCTGTTGATAGATATTTGGCAATATGTAGACCGTTAGAGTATC

ACCGCATTATGACAAACAACATGGTTATTATGTGCATAGTATTTTCATGGTTTTCACCTT

TTATGTGTGTGTTAGTCGGTGTTTTGTTAGGCTCAAGTCTAACTCTGTGTGGTTCATACA

TCGATAAGCTTTATTGTGAAAACTGGTCTATTGTGAAACTTTCCTGCTATTCCACTATGC

TTAATAATGTGTATGGATATATTATAATTCTTGTGTATTTCATTCACTCACTCTTCATTA

TGTGCTCTTACATAAAACTGATTGACACAAGCATCAGATCAAAAGAAGAAAGAAGCAAAT

TCATGCAGACATGTTTGCCACACCTGCTCTCCTTGCTCAACGTGACAGTTGCTCTGTTGT

TTGATGTTATGTTTACAAGATATGGCACAACCAGGAATTTTTCCCAAGATCTTCGGAATT

TTTTGGCAATAGAATTTCTAGTCATACCACCGATTCTAAATCCACTCATCTATGGACTGA

AACTGAAAAAGATTCGGAATCAGCTGATGCGGTTTTATCACAGAAACATCATCAGGGACA

TAGGGGACATGCCCAGACCAATGTAACCAGAAACAGACCACCAATGTGTGGAGCATTTCT

AATGTGGTGTGAGCAAGATCTGCTGAAATGTAATAACTGACACTTTTGCAGAGAAATAAT

GACTGTTGTTTTAATCACATTGTAAATAATTCTCGTGTTTCTTTAGAAGGTAAGAGGGAT

AAGCATAGGGATAACAAATCACAAATTACTAATGACCACCAA

>CL14888.Contig1_All

ATGTGCTTAACATAACTATATTGTGTTTGCTGTGTTGGTGGAAAGGAATAGGACAATGCT

TGTGTAAAAGATTCACTTGCATGCCGAGAACTACAGCACAGATGAATGAGTCTTCAATCA

GCATAATTTTGGTTCTCACAGCTTATGAAGATATGGGTGTAGCCAAATATGTATTGCTCA

CACTAGTATTCAGTACTTATTTTGCTTCTGTCATAGCCAATGCTATTATGATGATTTTCA

TTTGCCTGGATACTTTCCTCCATAAGCCAATGTATATGTTTCTATTGAGCTTAATTCTGA

ATGGCCTTATGGGTAGTACAGCTATCTGGCCAAAGGTTATGAACATCCTCCTAACTGATG

ACAACACAATCTCGTATGTAGGATGTATATTACAGATCTCCCTCATGAGGCTATATGGAG

GCTGCAATTATACAATCCTGGCAGTAATGGCTTATGACAGATTTGTTGCTGTATTTAATC

CACTGAATTACCAAACCATTATGAGCCCAGAAAGTGTTGGGCTACTTGTGTTTGCTGCCT

GCTTTATTCCCTTTTGTCTCGTACTTTGTGAAGCATACCTTCTTTCACGAATGCATCTGT

GTAGATACACATTACAGAGGATTTTTTGCAATCTAGATATATCCCATCTCTCCTGTGACA

TCAAGGACAGTCAAGTTGCTCGATTATTTGGCACATTTGGTAATGTTATCATCTTGCTGT

CTGTAGTTGTTGTACTATTGTCCTATATTAAAATTTTCCTGTTAGTCAAGAAATTTTCAG

CAGATGTCAGAAAGAAAACATTAGCTACTTGCACTCCTCATTTGTTCATTTTTTTCACTT

TCACATTTGTTTCTGTTTTTCCTGCCGTTTTTAACCGTCTTCATCCTGTTGTGTCATCCA

AAGCACAAGTCATTAGACCCTTTTTAATGGCAACCAACTATATTCTAATACCACCTTTAC

TGCATCCAGTTGTGTATGGTTTCAATAATAAAGATATTAGAGCAGCTGTTAGTAGGATTA

GATTATTAACTGGTAGCAAGATTGTTCCCAATTTTAAAATGTCATCAGTAATGAATGTAA

AAACACTAACTAACCACAATGGAAATGTACAAAAACAGTGTGTCTGTGTGTGTCCTGCCA

TCTGCACTTTATGACCCAGTCTCTCTGGAAGGCTGATTTCTCTGTGGGGCAACAGTCATG

AAGTCTCAAATCATAACCATTCTTAAATTAAATTTTAAGTTTTACTGATCAAAA

>CL14951.Contig3_All ACAATGCACCTTTGAATCTGGCTGTGATGTCACTTGAACGCTATGTGGCCATTTGTTTTC

CATTAAGACACAGTGAGATTGTAAACCAGAAATCAGCTGGTGTTTCTATCGGTCTCATAT

GGATCTTTAGTTCAATAAATCTTGTAATAGATATAGTTTATTCACTGATAATAAATCCAG

TCTCTTTTAAAGATTCCATACACTGCTTTCGAGAGAGGGTTATTATATCACGATGGCAAG

AGGATATGTTTGAGGGCTTTAACATCTTTTTCTTTGTGACAGTAACAGGCATTTTAATTT

TCACTTATGTCAATGTTGTAGTTGTAGCTCGATCTGCCTCAACAGAAAAAGACTCTGCTA

AAAAGGCACATAAGACCATACTGCTGCATTTTGGTCAGTTAGTACTCTGTGTTAACTCGT

TTATTTATGGAACCATAGAGAGAGCCTTGGCTATGACTAGCAGTAGTCGTTTGTTCATGG

ACCTGCGTTATATGAACTTCCTCTTTGTTCTCCTTCTGCCTCGGTGCCTGAGCCCCCTGA

TCTACGGCCTCAGAGACGATGCTGTTCGACCTTTATTTGTGTACTATGTCAGGTGTGGAT

ACCAAAAGGTGAAGCCCTCTGGAACCATACACTGACGATGACATATTCAACTCATTTTCC

TGTCTGAGCCTATCGTCATCATCATCACCGCCCTCCCTGAAGATGGAGCTGTTTGAACTG

TTGTAAACAAACATCTAAATCTCAGTGATTTTTTTCCTAGGTACTTGCAATTAATATCAT

TAGAAACTTTAAAATCTACTTGTTCGAATATATCGATTGTAAAAATGAAATACATCTGTA

GCATTTTTTTTAACTGAATGAAAACACACATTAAAAATGTAGTATTTCAGACAGAAGCAG

CCTGTTAATGACCTTCCCATTAGCTATTCACATGCTAACTGCGTGGAAAGTCAGCACGGT

GACTGGCTCGTTCTGTAGCAAAAGGAGGAGACAACACCTGTTTCAACACCACATGTTGAC

ATTGCGACTGAGAAATATTTGTTTCACTAGCTGTAGTTGTCATGGTTTCAGTTAGCTTTT

AGGGAATTGTAGAACTAATAAATTGATATGCTATAGCATAGTACTGTATGTGTTATCTCC

ACAAGCTTGAGGGAGCTCCACACTTTCTTGCTAGTACAACAATGGGCCTGGCATTCAAAA

CGATGCACTAAAACTTTGTATTTTTTCACATCATTTATTAAAAAGAGTGTTTTTACAGGT

AGTACCTTGTCGCCGCCATGATGAAAGTCCAA

>CL15592.Contig1_All

GTAATTCAGTGCTGTGTGGCTATGTAAGGATTTGCCACCTCTACCTTAAATGCACTGCCA

CAGATGATGGATTCAGTGAACCAATCCTTCTCTTCTGTGCTCCGAATCGCCAGCTTTGAT

ATTCACCCCTCAGCTGTGTACCCTATTTTCCTCATGGGCCTTTTACTTCACTTTTTCTCT

GTTGTCTCTAATCTGGCCATCCTGCTGCTGATTGCTACTCAGAGGACTCTCCACAAACCC

ATGTTTTACATCCTCTTCAGCCTGCCACTGAATGACCTTATTGGAATAACTGCAATGCTT

CCTCGTGTGTTAGTGGACATTGTGATCATAAAAAACACGGTCAGCTACTCTGCCTGTGTC

CTTCAGGCATTTCTGCTCCATATGTATGGTGGTGCTATCCCTTTCATACTGGCAGCGATG

GCATTTGATCGCTATGTTGCCATATGCAAGCCTTTGCGATACAACACAATAATGACCCCT

CTCGCTGTGGCTGGCATCATATCTCTTGCCTGGGGAGCAGATTTTGCCTTAGTTCTAGTC

CTGTTTCTGCTTCAGGTTAGGATTAGAAAGTGCAAAAATTACATTATTAATGTTCTTTGT

GATAATTTCTCTTTGCTTAGTCTTAACTGCGGAGAGGACCTTACAATTAATAACATTTAT

GGGTTAGCCATAACAGCCTTTATGCACATCACCACTGTCTCAGTACAGCTGTTCTCATAC

ACTCACATCCTAGTAACATGCCTGAAAAACAAACAATCAGATGCCAAGTCCAAGGCAGTA

AACACCTGTTTGGCACAGATAATTGCATTTTTCCTCTTTGAGATTGTTGCGTTATTCACA

ATACTGTCTTACCGATTTCCAAACATATCCTCAAATGCAAGGAATGCCTGTGGAATGATG

ATATTTCTGATTCTGCCTGTTCTCAATCCCATTATCTATGGAATGAAAACTAAGGATATC

AGAATAGCATTCTTTCAACTTTTAAAAAAAGGAAAAGTTTCATGGTAATTGTAGATGTCT

CAATGTCTAATACACTTGCAATTTTAGTTTTGTTTTTAGTTAACAGTTCCATTAATGTTT

ACTCCATTTGTTAAAAATCTG

>CL15592.Contig2_All TGGTGGTGCTATCCCTTTCATACTGGCAGCGATGGCATTTGATCGCTATGTTGCCATATG

CAAGCCTTTGCGATACAACACAATAATGACCCCTCTCGCTGTGGCTGGCATCATATCTCT

TGCCTGGGGAGCAAATTTTGCCTTAGTTCTAGTCCTGTTTCTGCTTCAGGTTAGGATTAG

AAAGTGCAAAAATTACATTATTAATGTTTTTTGTGATAATTTCTCATTGCTTAGTCTTAA

CTGCGGAGAGGACCTTACAATTAATAACATTTATGGGTTAGCCATAACAGCTGTACTGAG

ACAGTGGTGATGTGCATAAAGGCTGTTATGGCTAAC

>CL15979.Contig1_All GCAAGTCAGTACATGCTATTGATCCCAGGGTGTATTCGAATGATAAGTCAGGGAAAATAC

TGTAAAATAACTAGTGATTAAACTAATGATGCACAATGATTATATTAAAACAGATTTATT

ATAGCTTTTGTTCTTTCAGGTGATTTTGTTCTTTAATGTAATTGCTGCTTTAAAAAGAGG

GAATATTAACTATGGAGGGCCTCTCAAATACTTCAAGGCAAGAAATTATGTTAGAAAATG

GTTTGCCAAATGTGTCCTCAATTTTAACACTGCAAGGTTTCAATCTGCCTCCTCAAAGTG

TTGTTCCAGCCTTTCTGTTTGCCAGTCTGAATTACATGATCATCCTGTTCTGTAACCTTG

TGCTTCTCCTTACTATCCTCCTAAACAAAAGCCTCCATCAGCCCATGTACCTGCTGCTGC

TCAGCCTACCCATCAACGACATCATCGGCTCTACCGGTCTGTTCCCACAAGTCATTAATG

AACTTCTGTTGGACTCTAGGAGAATACCGTACAATGCCTGTGTTACCCAAGCTTTTTTCA

TTCACATCTATGGAGCAGGCTCCGTGTTCATTTTGACTGCCATGGCTTATGACCGATATG

TCGCTATATGCTGTCCACTGCAGTACGGCACAATTATGACACATGCTCATGTTATGAGGA

TAATCACACTGGTGTGGCTGAGTAATTTTGCCGTGATAGGTGTTCTGTTTTTTCTGCTTT

TGCGATTGCCCCGCTGCAGGTCTGAGATGCCGCATTCCTACTGTGATAATCCTTCCCTTC

TTAGACTAACCTGTGCTGACACGACTATTAGCAACATCTACGGACTGGCACTGGTGGCTG

TAACACAGGTTGTGGCTCTTGGGATAATTTTCTGTACCTACATCCAGATCCTTGTTGCTT

GTTTCAGGAGCAAAAGAGCAGACACTAAAAGCAAAGCTCTGCAGACGTGTGCAACGCATC

TGATTGTGTTTCTGCTTCTGGAATGCTTAGGTCTTTTCACAATCATATCATACAGGATAC

CCAATCTCTCCGCTCAAACAAGACGCTTCATTGGGGTGTCCACTATGATCTTCCCTCCCA

CTGTAAATCCAATTATTTACGGCCTCAAGACAAAAGAAATCAAAGACAAAATCTTTACAT

TTTTCCGCTCAAAAATTCTGCCAGGTTAAAGCTGAAGTCTGATGAAGCTGCTCTGCTGAT

GTAAATGCATGACTGGTACAATGAATGATTTTAAAAGCGTCATTATTTAAAAGTCAGTAC

AGAAGTACTGATGGATAGTGTTTTTACTGTATTTTCAACTTCCCTGTTCAGATTTTCATT

ACTTTTACATGAGAGACACATACTGTAATGGCTTTCAGCAATATATACATATTGTCATTT

TCCTTTTAAGTAGTGGCTGTAAAACCAAATGGTTAGGGAGTAAGGATGGTGCTGTTTGTC

CCCCCTGGCATCATTGAACAGGTACAGGTAAAGTGATGCATTTCCTCTTGTTTCAACAGC

TGCTGGTAGTGAAACGCTATAATTAACTTTGCTGGCTATGCTCAGGTCTGTAAATAATTA

CAGGTTAAAACTAGGCCTCTGTAATTAAAGCTCTCAACACCGTCTGTCAGCTAGTGTTGG

CATATGTCCCTACCGTTTTTTTTTTTCTCCCATCACCAGTTTTGTTACCACCACTTGACA

ACCAGAAATAACATTTCACCACAACAATTAATTGATTGCCTTTCAACCAGAATGCTGATA

ATATAACAAAATTGATCAATAAAAAATGAAAGTTTAATGTTAGCCAATTATATATGTTAA

TGCCCCAATTTTAATGCTGTCACCATTGTCCCAGTGCATTATGTAAGCTGGCCCTCTAAA

ATGCATTCAGTGTTGTTAACTGAATGTTTTGCTGCACAGAAAAAAAGAAAATAAAAATGT

CATACTGTTTGGTATTTGGTGTTATGGCAATCCAGGCCAGGCTAATCTCAAACCACCATG

TGTATCAGCCATAATATAAACTGTATTTCACAGCTGAAATAATACGAATATAAAAGTGTG

ATAAGGGAAGCAGTGGTGCAGTACAGTGGAAAGATGTTCTTTTATTAGCCCCTCAGTGAG

GAAATTTACAAAAATTACCTTTTAACTGACCTTTTAATGAGAAAACAGTGATTACTTCCC

CTTTTATGACCTCCACTGTTAAATACAGTTTACCCACCTTTTACAGATACACCACTTAAG

ATGCTATCATTCAGTTTTGCTGTTTTTGGTCAGTTGGTATTGAAGTGTGCATTGCAGTTA

CAGTGCAGTTTCTCAAATGTCAGGGCCTCAGAGCTGAGATCCCAAAGCATGATACAAGAG

CCAAGCATGGAGCAAATCTGACACTCATTAAAACTAAGACATAAGGCCACAGGGTTCACA

AGGCAGGCAAACAAAGCAGATTAAGCATCTGAAGTGTAAATAACATGAGCAGTGTCAGTT

TTGAATGGAAGCTCATGGAAGTTTATGATATTTCCCTTCAGGTGAGGATTTCAAATTGGA

GTCTCA

>CL15979.Contig2_All

GCAAGTCAGTACATGCTATTGATCCCAGGGTGTATTCGAATGATAAGTCAGGGAAAATAC

TGTAAAATAACTAGTGATTAAACTAATGATGCACAATGATTATATTAAAACAGATTTATT

ATAGCTTTTGTTCTTTCAGGTGATTTTGTTCTTTAATGTAATTGCTGGTAAGTTGTCCTG

TTAGAATTGTTATTTCTGTGGTTATTGTGAAGTGAAAAAAACATTCACATACAGCACTTT

ACCCAGTGAAAATGTATGACTGTACATAATGTTTAAACATTTCAGTTCAGCTTGAACTTT

ATAATATTGACTTAATTAATTAATGATGATTAAAGTTAATTTATGTATCACTATTGTATA

GGTTTATACAAATGACACAATGAAAACCTTCAATGTCTGTTTTTTTACAGCTTTAAAAAG

AGGGAATATTAACTATGGAGGGCCTCTCAAATACTTCAAGGCAAGAAATTATGTTAGAAA

ATGGTTTGCCAAATGTGTCCTCAATTTTAACACTGCAAGGTTTCAATCTGCCTCCTCAAA

GTGTTGTTCCAGCCTTTCTGTTTGCCAGTCTGAATTACATGATCATCCTGTTCTGTAACC

TTGTGCTTCTCCTTACTATCCTCCTAAACAAAAGCCTCCATCAGCCCATGTACCTGCTGC

TGCTCAGCCTACCCATCAACGACATCATCGGCTCTACCGGTCTGTTCCCACAAGTCATTA

ATGAACTTCTGTTGGACTCTAGGAGAATACCGTACAATGCCTGTGTTACCCAAGCTTTTT

TCATTCACATCTATGGAGCAGGCTCCGTGTTCATTTTGACTGCCATGGCTTATGACCGAT

ATGTCGCTATATGCTGTCCACTGCAGTACGGCACAATTATGACACATGCTCATGTTATGA

GGATAATCACACTGGTGTGGCTGAGTAATTTTGCCGTGATAGGTGTTCTGTTTTTTCTGC

TTTTGCGATTGCCCCGCTGCAGGTCTGAGATGCCGCATTCCTACTGTGATAATCCTTCCC

TTCTTAGACTAACCTGTGCTGACACGACTATTAGCAACATCTACGGACTGGCACTGGTGG

CTGTAACACAGGTTGTGGCTCTTGGGATAATTTTCTGTACCTACATCCAGATCCTTGTTG

CTTGTTTCAGGAGCAAAAGAGCAGACACTAAAAGCAAAGCTCTGCAGACGTGTGCAACGC

ATCTGATTGTGTTTCTGCTTCTGGAATGCTTAGGTCTTTTCACAATCATATCATACAGGA

TACCCAATCTCTCCGCTCAAACAAGACGCTTCATTGGGGTGTCCACTATGATCTTCCCTC

CCACTGTAAATCCAATTATTTACGGCCTCAAGACAAAAGAAATCAAAGACAAAATCTTTA

CATTTTTCCGCTCAAAAATTCTGCCAGGTTAAAGCTGAAGTCTGATGAAGCTGCTCTGCT

GATGTAAATGCATGACTGGTACAATGAATGATTTTAAAAGCGTCATTATTTAAAAGTCAG

TACAGAAGTACTGATGGATAGTGTTTTTACTGTATTTTCAACTTCCCTGTTCAGATTTTC

ATTACTTTTACATGAGAGACACATACTGTAATGGCTTTCAGCAATATATACATATTGTCA

TTTTCCTTTTAAGTAGTGGCTGTAAAACCAAATGGTTAGGGAGTAAGGATGGTGCTGTTT

GTCCCCCCTGGCATCATTGAACAGGTACAGGTAAAGTGATGCATTTCCTCTTGTTTCAAC

AGCTGCTGGTAGTGAAACGCTATAATTAACTTTGCTGGCTATGCTCAGGTCTGTAAATAA

TTACAGGTTAAAACTAGGCCTCTGTAATTAAAGCTCTCAACACCGTCTGTCAGCTAGTGT

TGGCATATGTCCCTACCGTTTTTTTTTTTCTCCCATCACCAGTTTTGTTACCACCACTTG

ACAACCAGAAATAACATTTCACCACAACAATTAATTGATTGCCTTTCAACCAGAATGCTG

ATAATATAACAAAATTGATCAATAAAAAATGAAAGTTTAATGTTAGCCAATTATATATGT

TAATGCCCCAATTTTAATGCTGTCACCATTGTCCCAGTGCATTATGTAAGCTGGCCCTCT

AAAATGCATTCAGTGTTGTTAACTGAATGTTTTGCTGCACAGAAAAAAAGAAAATAAAAA

TGTCATACTGTTTGGTATTTGGTGTTATGGCAATCCAGGCCAGGCTAATCTCAAACCACC

ATGTGTATCAGCCATAATATAAACTGTATTTCACAGCTGAAATAATACGAATATAAAAGT

GTGATAAGGGAAGCAGTGGTGCAGTACAGTGGAAAGATGTTCTTTTATTAGCCCCTCAGT

GAGGAAATTTACAAAAATTACCTTTTAACTGACCTTTTAATGAGAAAACAGTGATTACTT

CCCCTTTTATGACCTCCACTGTTAAATACAGTTTACCCACCTTTTACAGATACACCACTT

AAGATGCTATCATTCAGTTTTGCTGTTTTTGGTCAGTTGGTATTGAAGTGTGCATTGCAG

TTACAGTGCAGTTTCTCAAATGTCAGGGCCTCAGAGCTGAGATCCCAAAGCATGATACAA

GAGCCAAGCATGGAGCAAATCTGACACTCATTAAAACTAAGACATAAGGCCACAGGGTTC

ACAAGGCAGGCAAACAAAGCAGATTAAGCATCTGAAGTGTAAATAACATGAGCAGTGTCA

GTTTTGAATGGAAGCTCATGGAAGTTTATGATATTTCCCTTCAGGTGAGGATTTCAAATT

GGAGTCTCAACAAAACACAACAGTTTACAATCTTTTTTTCTATAACACTGCTGATCTACT

GCCTCTCTCTCTCTCTCTGACCCCTTCCCCCATGCTGGTCCCCCTCAAC

>CL2746.Contig1_All AAATACAGCAGGTACACTGGGTGGCTCTGATTCAGAGAAAACTGGTGATAAACAAGTTTC

AAAGACATGTCTGGAAACAGCACTGAACAAAAGATTCTGAAGGGAAATGACCCAGTTCGG

CTGAACCTGACCACAGCCCTGGTGCAGGTCCTGGTATGGCCCTTCGTCTACATCAACCTC

TTTATGTATTTCACCTACCGGAAGAAGCCGGCTCTCCAGGCTGAACCTCGCTATGTGCTC

TTTGCTCAGACACTGCTCGCTGACTCGGCCCTCTTCATCATGACGGACTTTGTGGTCATC

GGCATCCACGTTCACCTCCTGTTGCCCCTGCCTGTTTGTATCCCTGTGGCGGTCTCCAAC

CAGGCCCTGGCACTGGTGTCGCCCACCCTCATCACGGCCATGTGTCTGGAGCGCTATGTG

GCCATCTGTTTCCCCCTGCGACACGTCGACGTCTTCACCCCGACCAGGACTATGTTTATC

TCAGCGGCTGTCTGGTTCCTGAGCTACCTCCGGCCATTTGTTGACCTGTTCATCCTTTTT

ACCACAATGCCCAAGGGCTACATGAACAGACTGAACTTCTGCTATTACGAAATTCTTTTG

GTTGCCAGGTGGCACATGGAAATGAGGGGTAATTTGTTTGCTTTGAACTACCTGGTGCTT

CTGTCCATTTTGTTTTTCTGTTATGTGGCGATCATCCGAGTAGCACGAAGAGCCTCTGGA

GCGGACAGGCAGGCAGCCTCCAAGGGCCAGAGAACACTACTGCTGCACCTGCTACAGCTC

TTCATGTGCACACTGGAGGTTTTCTGCCCATACATTGAATCCCAGGTCATCCTGATCGAC

ATAGACTTGTTTATTGTGGTGCGCTATTTCAATTTCCTGGCATTCACTATTCTGTCCAGG

GCCATTACCCCACTTATCTATGGCGTCAGAGACGAGAAGTTTCAAGCTGCTATGAAAGAG

TTTTTGCTACAGAAAAGGAGTAAAGTTGGTTTTGTTCACTAATTTTTGCGAATAAAATCT

TACATATGGCTACAAATTGCAAATAGGGACTCTGATGTAACTAAAGCAAGATTTAATGTT

TGAAAACACAGCTGTCTGAAAAGTTTGGTTATCAAATCTCCAGTCTAAATTTTTTACAAG

CTCAGGAGTCAATCACCAACACAGAGGTATGGCTCTCACGCAAAGTTTATTGCCAGACAG

CAGAGCATATAAAACCGGAGCTTGAAAACAAATGTTTTTGTTTGTTTCTTTTGCACTGTT

TGTTTACTGTCACTGGTTTTTACCACATTGTTTTTGTATGAAATATTTCTAATAATTGAT

CCAAGACTGTGTGACCATGACAAGTTTCTGCCCTCTCCAGCAGGTTTTGTGAAACGTGTT

TGAAAGAGCAAGGGCAAGTACAGCTAGAGCGAAGACAGATTTGCCAGCTGCCACAAACTA

TGGCACACACTGTCTTAGAAAGGGCTGGGCAGTGACGGTGCTCTGAACGTGGCTGAAAAT

ACATCAGCTATCACAGAGGTTCACAGTCCTGCCCCTCCCCAAACAGCTCTTTCTCCTGTA

CTGTCTGTTTCCCATATTATTTCCCTGCTCTGATTGGCACTGCTGCAGGAAGAGTTAGTA

ACCATTGTCTTCTACTCATTCACACACTGCCCACACCACACTGCCATTGTCAGCCTCTCT

AGACTATAACATCTTATTAACAATTATATTTGACATGAATTCCCCATCATTTTAATAGGC

CCAGCAACATAAGAACATGTAATAAACATGACATTGGCAATATGAACTGATCCACTTTTA

ATCCCTTTGCCTTTCCAAATAAAAAATAATGAATTAAAATTCATTATTCACCATTACTAT

TTTTTTCTGAGTAAACAACTATGTCAATCTATGAAGTAACCATAAATGGTGTGAGGTTGT

AAAAAAGAACTATTTTACACTTACAAAATATGCCAGTGATAGAGCCTGTCAGAAATATAA

AACATTTAATGTTTGAAATATGTTTATATGTTACCTTATTACACATACACAGATTACTAA

AATGCTAGAGGAGACCTTTGATGCATTAAACTGATAGAAATCTGTTAGTTAAACCCTGAT

CAATTGTGGCAAGGATGACTGAATAAAAATGAAATAAAATTACATCACCATAGGGAAAAC

AGTCAGCTCAGTGCTTACATAACCATGGTTATCCTCAGCACTAAACCAAAACCATGCATG

TTGGTGCAACATTTTTACACCCATAAAAAAT

>CL2771.Contig2_All

TTGACATAAGCAACTCAACAACTGCAGCCATTACATACCTTCTATAGATGCTCAGAAGGT

GCTGCAGAAATTATTTGTTTTGATTTGTCAAGAAAGAGTACACATGCCTGCTGTGTGTAT

CATGAAGAATGCATCTTCTTTTACAACCTTCATACTGATTAATTATGAGCCAATGGAACA

ACAGAGATATTTATATATCATCATTTTTTTACTACCATATATGTTAACTATTATTTTAAA

TACTTGCTTAATTTACATTATTTGTAAAGACAGAACCTTACATGAACCAATGTACATTTT

TATTTGCAATTTGTCATTTAATGGAATCTATGGCAGCACAGCTTTGTTACCACATATGCT

GAATAAGCTTGCCACAAAGTCATATAAGATCTCTTTACCAAATTGCCTTACTCAAGTATT

TTGTTTACACACGTTTGCCACTGTTGAACTGATGATACTTGCCATCATGGGATATGACAG

ATATGCTGCAATATGTACCCCCTTACACTATCATAACGAAATGTCTCCAAGAAAAGTTAA

AATTCTCATTGCAGTAGCTTGGCTTTTTCCTTTCTGTACATTTGCACTATTTTTATCACT

AACAATTCGACTATCTTTTTGTAGTAATGTCATTTACAAAACCCACTGTACAAATTATGA

TTTAGTTAAAATTTCCTGCAGTGACACTTCTGTGAATAACATTGTTGGGATCATTCTCAC

GGGTATGTATATGTTTCCGCAGTTCCTTGTCATTTTAATTTCATATGTTCAGATTCTCAG

GATCTGTTTACATGCATCCAAGGAGTGTCAGAAGAAAGCACTCCAGACCTGCATACCACA

TTTGTTGACAGTCATAAACTACGTTTTTGGTGTTTGTTTTGAACTGATCCAAGTCCGTAT

GAAGGCAACCCAAATTCCATATGGGATGGCTCTGTTTATGTCTGTGTATTTTTTGATTTT

GTCCACCCTGTTAAATCCTCTAATATATGGTTCTAATGCTCTAAAAATACATATTTATCG

TTATCTTCAAAAGAGGAAGTTAGCTTCAGTATCGTAATTCACAGGATTGTCATTTGAAAA

AAAAGGTCAGCTCCAACACAGAATGTCACTGCCTTCACAGCAGATATAACTGCCAAAAAA

AAACTTATGTTGAGAGCAAAGGAAATTGGTTGTTACATTTTCACTTTCATTCAACAGTAT

ATTACTCACATAATTTTTAAAAAAATTATAAAAATGGACATGGTAAC

>CL2791.Contig1_All

TCCAAGTCCCAGTGGTGACCTGTGTGTACCAAGTGGCATAAAAGCAGCCAACACAGCAAT

GCCACACCACAAAGGATGGACTACTTGTCTCAAGCAGCAGTACTGCTAGGATGAAATATG

GCCATTTTCTATTTGAACAATCCAACCATCTTTGAACGGATCATTTTCACTGTATTTTCA

AAAGGACATGGAAGGACAAAATAACACAGTTGTGAGGGTGTCTGAGTTTATTATAACAGG

CTTTGACCATCTCTCACATCAGAAACTTCTTGGGTCTATTATTTTTATTACTTATGTTCT

GATATTGTTTTTTTGGAGCACAAATTTGAGTATAATAGCTGCAAACAGAAGCTTGCATTC

ACCAATGTATATACTTATTTGCAACTTAGCTATTGTGGATATAGTGTTCAGCACCAGCTC

TAACGTAAGTATGATAGTCGTCCTTCTTGCAGAAATTAAAACTATTTCCTTCAATTCATG

CATCACTGGCATGTTCACCTATCATCTCGGCGACATCACCACGTGTCTGACAATTGCGCT

GATGGCCGTGGATCGCATGCTTGCAATTAGGTTTCCTCTAAGATCCATATACTATCGGTG

GGTTGATTTGGCAGTACGGTGGATATTTCACACTCATCTTGTTGACATATGTTGTCATTG

TCTGGACTGTTCTGAGACTCCCAGAAAAGGGAAGCAGGAAGAAAATGTTTACTACTTGTA

TTTCACATATCATTGTGGTTTCGGCTTATTACGCACCCAAATTGGTATCATATCTGCTTA

CTAGAATAGGGGTGAAACTTAACCTAACTGAGAGAAATGCTGTGCTTATTGTAGCTTCAA

CGCTGCCACATCTCATCAACCCTGTAACTTACTGTCTGACAACAAAAGAACTAAAAATAC

GGCTAATAAACTTGTTGACCAAAAATCGTGTGGGAATGAAGTGACTGAGTTAAAGGATAT

TGTTTTTGTACTCATGATGTAAATGTAACATAATGTTCATATCTATCTGCAACTCTACAC

TTAAATTTATGTCAACAGCATTTGCACTAATGCATACCAATGAATTTACTGAATTTACTA

AAGAAATGTTGATCACATCTCTGTCCCCCTGCCTCTTCTGAATGACTAGTAGCATAGTCT

GTCATTGTCCAGTCTGTTTAACTGTACCACTTATATGCTCTATACAAAATACTCACAATA

AATTAAATCAGATAATATATAATATACAATAAAATATTCCACTAAAC

>CL2791.Contig2_All TCCAAGTCCCAGTGGTGACCTGTGTGTACCAAGTGGCATAAAAGCAGCCAACACAGCAAT

GCCACACCACAAAGGATGGACTACTTGTCTCAAGCAGCAGTACTGCTAGGATGAAATATG

GCCATTTTCTATTTGAACAATCCAACCATCTTTGAACGGATCATTTTCACTGTATTTTCA

AAAGGACATGGAAGGACAAAATAACACAGTTGTGAGGGTGTCTGAGTTTATTATAACAGG

CTTTGACCATCTCTCACATCAGAAACTTCTTGGGTCTATTATTTTTATTACTTATGTTCT

GATATTGTTTTTTTGGAGCACAAATTTGAGTATAATAGCTGCAAACAGAAGCTTGCATTC

ACCAATGTATATACTTATTTGCAACTTAGCTATTGTGGATATAGTGTTCAGCACCAGCTC

TAACGTAAGTATGATAGTCGTCCTTCTTGCAGAAATTAAAACTATTTCCTTCAATTCATG

CATCACTGGCATGTTCACCTATCATCTCGGCGACATCACCACGTGTCTGACAATTGCGCT

GATGGCCGTGGATCGCATGCTTGCAATTAGGTTTCCTCTAAGGTACCACAGCATCCTCAC

AAACACACGCTGTTTCGTGCTCATTACAATGGTCTGGATAGTGGGTATGTTAACGTTGGG

TCCGCTTATGGCAGCAGCGCACAGTGTTCCATACTGCCAGCCCATAATAAGATATGTATT

CTGTGACTATGCTGCAATGATTCGAGCTGGATGTGTAAACCCGGAACCTTACTTTTCAGA

TCCATATACTATCGGTGGGTTGATTTGGCAGTACGGTGGATATTTCACACTCATCTTGTT

GACATATGTTGTCATTGTCTGGACTGTTCTGAGACTCCCAGAAAAGGGAAGCAGGAAGAA

AATGTTTACTACTTGTATTTCACATATCATTGTGGTTTCGGCTTATTACGCACCCAAATT

GGTATCATATCTGCTTACTAGAATAGGGGTGAAACTTAACCTAACTGAGAGAAATGCTGT

GCTTATTGTAGCTTCAACGCTGCCACATCTCATCAACCCTGTAACTTACTGTCTGACAAC

AAAAGAACTAAAAATACGGCTAATAAACTTGTTGACCAAAAATCGTGTGGGAATGAAGTG

ACTGAGTTAAAGGATATTGTTTTTGTACTCATGATGTAAATGTAACATAATGTTCATATC

TATCTGCAACTCTACACTTAAATTTATGTCAACAGCATTTGCACTAATGCATACCAATGA

ATTTACTGAATTTACTAAAGAAATGTTGATCACATCTCTGTCCCCCTGCCTCTTCTGAAT

GACTAGTAGCATAGTCTGTCATTGTCCAGTCTGTTTAACTGTACCACTTATATGCTCTAT

ACAAAATACTCACAATAAATTAAATCAGATAATATATAATATACAATAAAATATTCCACT

AAAC

>CL321.Contig1_All TTTTAAACTGTTGAGGCTGAAGAAACCCTTCAAAAGGAGAGTAAAATGGAAAATGAAACG

GTACCATCATACTTTTATTTCACATTATTTAAGGGTTATGAAAATGTAAGAATAATGTTT

TTTCTGTTAACGATAGTAACATACTTTTTCATCATAGTTTTCAATGTAACTATCCTCCTG

GTTGTATTCAAGGACAAGTCTCTTCACGAACCCATGTATCTTTTGGTGTCTTGCCTGTTA

TTCAACTCTCTGTATGGCAGCTCTGCACTTTTTCCGAGACTTTCTGCTGACCTTCTATCA

ACAACTCACACAATATCCCGGCCTGCATGTTTCACGCAAATATTTATTATTTACATATAT

GCAATTTCTGAATTCACTGTACTAAGTGTTATGGCATATGACAGATATGTAGCCATATGT

GAACCTTTGCAATATCATAACATCATGACACAAAGAAAGACATTTTTGCTTGTTTTATGT

GCTTTTTCCTACTCTGTCTTTGGTGTGGCTGTTGGAATATATTTATCTATAGGGCTACTT

CTGTGTGGCAATCAGATACACCGACTGTATTGCTCAAATTGGTCTGTAGTAAGACTTTCC

TGTGTGTCAACATTAATTAATAACATTTGGGGTTTTTTTCTTACTGTGACTACAGTCTTT

CTGCCTGCAGGTTTCATATTGTTTACATACATGAGAATTCTCCTTGTCTGCCGTAAAAGC

ACAGCAGAATTTAGAGGCAAAGCGCTGCAAACATGTCTACCTCACATTATAAATTTTGTT

ACCTATTCAATAGCAATATTTTCTGATATTGCACTCAGTCGGTATGAACCTGGCCAGATT

TTAAATGCAATAGTTTTGATCATTTCTCTTGAATTTATTGTGATTCCTCCAATACTAAAT

CCTCTGATATATGGCCTAAATTTACCAGGTATCCGAAGGAAAATTGGCAGTATGACGATT

TCATTAAAAGTTGCTTTGAGTTCTTAGAAATGTATTTACCAGAAACATATTACCAGAAAG

CATACTAAACCTATGAAATACATTTCTTATACTATGATGTATTTTTATGTATATAACCAG

GGCTGTACTGGAGGGTAAACGTTAGTAAACTGAGTTTACCCACCTCTAAAATTTCAGAGA

TAGAGTTTATAAATCTCTGAGGAAAGTTTATCCACATATTAATAGAGTGCATTTATATCA

TAATGAAGTTTATGCACCAAATACAACTTGTAATATTTCAAAGTTACACTACATAATTTA

TTTTTTACAATCTGTACACTAGGGGCCATTTACAATACCATTTGTACTGTTTAAACATAT

TTTGAATATCTTTTTTAAACATCGGATGCCATGCAGCGCATCAACCTCACTGTGATGACC

AAATTCATAGTTTGCCCACCTCTTACTGCACCACTGCAACCCTGTTTATAACACTGTGTT

ATATTGTAGGATATAATTGCTTGTTATGTGAAAGCTGATTGGTGTTTTATAGTGTAAAAT

GAATGGCTGACTATTGGGACAGTTATCTGATGGCCTGCTAATGTATCTATACAGTCAAGA

CACTGAAGTAGCTTTTTCAAAAATGACATAGAGTTCTGGGTTTTGTAAATAAAAAGGAAA

TTGATGAGTAATTGGACAGTTATCTGTTGGTCTGCTAATGTATGTGTATAGTGTAAAATC

CACTGAAGTGAATATTTAAAATATTACACAGGAAATCTTAAAATGTATGTGCACGTGTGC

CAAAATAAAAAGAAAA

>CL321.Contig2_All TTTTCTGATATTGCACTCAGTCGATTTGAACCTGGACAAATTATAAATGCGATAGCTTTG

ATCATTTCTCTTGAATTTATTGTGATTCCTCCAATACTAAATCCTCTGATATATGGCCTA

AACTTACCAGATATCCGAAGGAAAATTCTCAGTATGATGATTTCATTAAAAGTTGCTTTA

AGCTATTAGAAATGCATTTATCAAGAACATATTACCAGAAATCATACTAAACGTATGAAA

TATGTTTCTTATTTTATGATGTATTTTTATGTATATAACCAGGGTAGTACTGGAGGGTAA

ACGTTAGTAAACTGAGTTTACCCACCTCTGACATTTCAGAGATAGAGTTGATTCACCTCT

GAGGAGAGTTTGTCCACATATTAGTAGATTGCATTTACTTCATAATATAGTTTATGCACC

AAATAAAATGTGTAATATTCCAAAGTTACACCTCATCATTCATATTTACAACCTGTGCAC

CATGGACCATTTACAATACCACTTGTACTGTTTAAACATA

>CL3955.Contig1_All

CAGAATGTTATATCATGGATCCGTTTTGTCCCTGTGATTAATACATTCATATAAGATATG

TGCTATGTAATGATTTTATATAGATCTTTACTTATGTTTGTATAGAACCTAATGGCCATG

AATAATCAGTCTATTGAGGGCACTCTACTGGTGCATCAGCAGTTTTTTAAGGTGGAAATG

ACAGAGGGGCCAGTCTCTAAACTTATTGTGGCCATTCTCGTGGCCCTGGTATTCATCTTT

ATTAACAGCATCATGTTTCACACTCTTCTAAGTAAGCCTGTGTTTAGAGAGCTGCCTCGC

TATATCCTCTTTGCCCACATGCTCTGCAATGACTCAGTCCAGTTAATGCTGTCCATGTCA

TTGTATCTTATGATCGTGAGCTTACATCAGATTCCCAAAGCTCTGTGTGCATTACTTGTG

CTGCTTGCATCAACAACATTTCGCAATGCAGCTTTGAACCTGGCTGTGATGTCACTTGAG

CGCTATGTGGCCATTTGTTTTCCA

>CL3955.Contig2_All CCTCTTCTCTCTTTCAGATTTGTTGAATGAATCCAATACAATACATTTAAGTTTGTGGTC

GTGATTTGACAATATGTAGAAAAGTTCAACAGGTATGGACGCTTTCACCACACACTGTAC

ATATGTGGAATGATGACATCATTTCTGCCTATAAAAGTGAGCTCTAGGTGGTGTACACAA

CGTGCATGCCGCTTTTTCAAGCGTGTAGCAACAGTCATCCATTTAATCACTATAAATGTA

CACATGTTTGATTTTGATTTCTTGTGTGAAAAAAATTCACTTGGTGTTTAATCTGGTGTT

TAATTTGATGTTATCTCTGACCCCTTTTCTGATATTTGAAAGCTCTGTTGTACTGTGCTT

GGGAATGTGAAAAAGACTTTTGTGTTTATGTATATGATTTTTACAAGTCTTGCGAAATTA

CTGTGTATTGTGTTTTATACAGGTTTTTCTTTCTGCATCAGGACATTTCATTGACACAAA

ATATTTTTTGCAAAGGAAAGAAGCATTGTTGAACTGTTAAGAAAAAAGATCTTACTGTCA

AATATTTCATTTAAGCATAATCAGAATGTTATATCATGGTTCCGTTTTGTCCCTGTGATT

AATACATTCATATAAGATATGTGCTATGTAATGATTTTATATACATCTTTACTTATGTTT

GTATAGACCTAATGGCCATGAATAATGAGTCCATTGAGGGCACCCTACTGGTGCATCAGC

AGTTTTTTAAGGTGGAAATGACAGAGGGGCCAGCGTCTAAACTTATTGCGGCCATTCTCA

TGGCCCTGGTTTTCATCTTTATTAACAGCATCATGTTTCACACTCTTCTAAGTAAGCCTG

TGTTTAGAGAGCTGCCTCGCTATATCCTCTTTGCCCACATGCTCTGCAATGACTCAGTCC

AGTTGCTGGTATGTATGACATTGTATCTTATGACTGTAAACTTACAACAGATTCCTAAAG

CTCTGTGTGCATTATTTTTATTTTTTGCAACAACAACATTTAACAATGCACCTTTGAATC

TGGCTGTGATGTCACTTGAACGCTATGTGGCCATTTGTTTTCCATTAAGACACAGTGAGA

TTGTGAACCAGAAATCAGCAGGTGTTTCTATCGGTCTCATATGGATCTTTAGTTCAATAA

ATCTTGGAATAGATGTAATTTATTCACTGATGGTGAGTCCAGGCTCTTTTAAAGATTCCA

TGTACTGCAGTCGAGAGAGGATTATCATATCACCATGGCAAATAGATAAGTTTCAGGGCT

TTAATTACTTTTTCTTTGTGACTGTAACAGCCATTTTAATTTTCAGCTACATTAATGTTG

TAGTTGCAGCTCGATCTGCCTCATCACAAAAAGACTCTGCTAAAAAGGCACATAAGACCA

TACTGCTGCATCTTGGTCAGTTAGTACTCTGTGTTAACTCGTTTATTTTTGGAACCATAG

AGAGAGCCTTGGCTATGACCAGCAGTAGTCGTTTATTCATAGACCTGCATTATATGAACT

TCCTCTTTGTTCTCCTTCTGCCTCGGTGCCTGAGCCCCCTGATCTACGGCCTCAGAGATG

ATGCTGTTCGACCCTTATTTCTCTACTATGTCAGGTGTGGATACCAAAAGGTGAAGCCCT

CTGTAACCATACACTGATGATGTTAGGGTGTTATGAAAAACCCATTTAATTTCTTATACA

CAGCATGAATGTGCTATCAAACTACAACAACAGCAGATAATAGTTACATGTTTGTTTTTG

CTATACTTTTTGTCACTGTAAAGCTTTTATGTAACATTGCTAGCAACATGTGAATGCTAT

GGTCTAC

>CL4703.Contig1_All

CCCGGGTACAGATCATGGTGTTCTATGCATCTCAGACTATTTAATGTGAAAGTAGTACTT

ATGTGTCTGGCATCCTGGTTGTTTTTTCTCTTTCCTGTTTTTAACCTGGGCATATGTATT

TCATCCAAGTTCATACATGATGTGAAATACGATATGAGATTTTTATTTTCACTCCATTGA

GTCAAAGCTTTGTCACATGAGTAGGCATGGGTAGACTTCTGAAGTAATGATGCTTAATGG

TGCTTTTCAGCATCTAATATGTAAAGTATATGATTGTGAAGTGTTATATGATCTGTCAAA

AAGCATTGTTGATATTTTTTGTGTATCAATAATCTGATGATTTTTAAATTAGTTTTACAC

TTTCTGTCTTTTTAAACATGGTGTTATGCATTTAATAGATCTTGGGTAATTGTATAATTC

ATTAGTCATTGATAATAATGATATATCGTGTTTCTTTCAACTGACTTCATGATTTTGTGA

AGTTTTTGAGGAGGTCCATAGACGTAAATTAATTACAAATTTTGGGGATTAACATTTGGT

AATCTGTCAGAGACTGTCAGCAGTTCTCTAAAGCCATGCCGAACGTAACAGGATTCCCTG

CCCGCTTCCCCGCCGTCTTCTCTTCTCTTGAAGACTGGCTCATAGTGAAGGCCTTTTTTT

GTACGGCTCCCTGTGCCTTCTTCCTGTATGTGAATTTAGTCATGTTGTACACACTAAGGT

CTAAAGAAATCTTCACTGAAACCCCTCGCTACATACTGTTCAGTCACCTGCTCGTGTCTG

ACTCCCTGCAGCTGTGGTGCACCCTTGTGTACTATATACTGTACAACCAGACATTTGATC

AGGAGACTTTAAAAATAATAGGTGTTTACTGCTTTTTGAATTACCTTGTCTGTCGAATCA

CAAATAACTATCTTACCCCACTTTATCTAGGCCTTATGTCCCTGGAGCGTTATATTGCTA

TATGTTTTCCCCTTCGACATGCTGAGATAGCCAACAAAAGCAGGACCATGTTGGCCATAT

TATCTGTTTGGTTACTGGGCTTAATTCTCTGGACAGCTGACCTAGCTGCTGCCATGGTAT

TGCGGGGTAAGGAGGGGGGCTGTACTGACTACATAATCGCCCAAATGGTAGTTACCTATC

AGGTAAACACAGCATTAACAGGCCTTGTGTTTTCTTTGGTGAGTGTTGTTATAGTCTGTA

TATACATAGCCATTATGATAACAGCCAAGTCAAGTTCTACTGCTGACAAGTCCAAGGCGA

GTAAGGCCCACAAAACAGTACTCCTACACATGGTGCAGCTTTGCCTGTGTCTTTTGTCTC

TTCTATTCGGTGTGGTTCGCAGGGAGCTGGGTTTAAGCGGGCTGGACCCAGTGCTTGTTA

ATGAAGTGACGATTTTTTTGTTCTTAATGTTGAATATCTTGCCCAGGTGCCTCAGTCCTC

TTGTATATGGTCTGAGAGACAAGACATTCAAGGCCTATTTTAAAATCCATTTTCTTTTCT

GTATCAAAAGTAAAATACAGCCATCAGACCTTGAGATTTAGTATGGAACCTAGAGAGATT

TTCTGATATGGCCATGACTTTTCTGTAGGCTGCTCTGGCCCAAGTTCAATTACATTTAAT

TAAGTTGTATTAAGTCCATTACAAATGACAGCTTTTGTGTTTCATGTTCATGTTCACCTG

TTTGTTTAAACTATGTGTGTATTCTAAGAACCCATTGCTGTATTCTGTAAGCTCTTTCAG

TTTATTTTTTGCCTCTGCTAGTTATGAGAATAAATATGTGTATGTATGTGTATATGTGTA

ATTTGAACTTTTACTATGCATTGCCTGGCTCCATTGATCCACATTGAATTGGTTGTCAAA

TGAGCTGAGTTGTAAGCAACTGCACTTCTGGACTACCATGACCTGGATGAATGAGAATCT

ACACAGACATCTTAAAGACCTACTGTGTCAACATTTCTTCTATGTAGCGGTTAGGTTGTG

TATCGCAACGACCTGAGTAGTATTCCCTAGCCTGCACCCTTTCTAAGTGCACAGTTGCTT

CTATAGCAGGCGATATGTATCAACAAGTAAGATATTCTTCAAGTATTCTTTCAGCTGATC

AGAAATGGAAAAGGCGGAGGTCTATTTCCCTGGACTTGTGAGTTGTCCCCTGTTGGGACC

TATAATAGCAAGATGGCGGATGTACGTGGCAGTCATCATTAAGCCTATGAGCTGTACATA

TGTATACAGTACAGGGTATCCCAAAAGTCACTATACAGTTTTAATGAATTTCTAAAAAAT

ATACAAGAAACTGAGTTTATTCATGTTTTTAGCATCAACAGAGGAGTCATTGCATTCATT

GCATTACGTGCCATTTTGGGACACTCTGTATAAGTGAAAGAGCGTTGTGTTTGTGTGTGT

GTTTTGTTAGTGCTGGCATGAACATGATTGTACACGGTCGAGATTGTAAGTTTGTGTGTG

TGTGTGTGAGTTTGTGTGTGTGTGTGTGTGTGT

>CL4703.Contig2_All

CCCGGGTACAGATCATGGTGTTCTATGCATCTCAGACTATTTAATGTGAAAGTAGTACTT

ATGTGTCTGGCATCCTGGTTGTTTTTTCTCTTTCCTGTTTTTAACCTGGGCATATGTATT

TCATCCAAGTTCATACATGATGTGAAATACGATATGAGATTTTTATTTTCACTCCATTGA

GTCAAAGCTTTGTCACATGAGTAGGCATGGGTAGACTTCTGAAGTAATGATGCTTAATGG

TGCTTTTCAGCATCTAATATGTAAAGTATATGATTGTGAAGTGTTATATGATCTGTCAAA

AAGCATTGTTGATATTTTTTGTGTATCAATAATCTGATGATTTTTAAATTAGTTTTACAC

TTTCTGTCTTTTTAAACATGGTGTTATGCATTTAATAGATCTTGGGTAATTGTATAATTC

ATTAGTCATTGATAATAATGATATATCGTGTTTCTTTCAACTGACTTCATGATTTTGTGA

AGTTTTTGAGGAGGTCCATAGACGTAAATTAATTACAAATTTTGGGGATTAACATTTGGT

AATCTGTCAGAGACTGTCAGCAGTTCTCTAAAGCCATGCCGAACGTAACAGGATTCCCTG

CCCGCTTCCCCGCCGTCTTCTCTTCTCTTGAAGACTGGCTCATAGTGAAGGCCTTTTTTT

GTACGGCTCCCTGTGCCTTCTTCCTGTATGTGAATTTAGTCATGTTGTACACACTAAGGT

CTAAAGAAATCTTCACTGAAACCCCTCGCTACATACTGTTCAGTCACCTGCTCGTGTCTG

ACTCCCTGCAGCTGTGGTGCACCCTTGTGTACTATATACTGTACAACCAGACATTTGATC

AGGAGACTTTAAAAATAATAGGTGTTTACTGCTTTTTGAATTACCTTGTCTGTCGAATCA

CAAATAACTATCTTACCCCACTTTATCTAGGCCTTATGTCCCTGGAGCGTTATATTGCTA

TATGTTTTCCCCTTCGACATGCTGAGATAGCCAACAAAAGCAGGACCATGTTGGCCATAT

TATCTGTTTGGTTACTGGGCTTAATTCTCTGGACAGCTGACCTAGCTGCTGCCATGGTAT

TGCGGGGTAAGGAGGGGGGCTGTACTGACTACATAATCGCCCAAATGGTAGTTACCTATC

AGGTAAACACAGCATTAACAGGCCTTGTGTTTTCTTTGGTGAGTGTTGTTATAGTCTGTA

TATACATAGCCATTATGATAACAGCCAAGTCAAGTTCTACTGCTGACAAGTCCAAGGCGA

GTAAGGCCCACAAAACAGTACTCCTACACATGGTGCAGCTTTGCCTGTGTCTTTTGTCTC

TTCTATTCGGTGTGGTTCGCAGGGAGCTGGGTTTAAGCGGGCTGGACCCAGTGCTTGTTA

ATGAAGTGACGATTTTTTTGTTCTTAATGTTGAATATCTTGCCCAGGTGCCTCAGTCCTC

TTGTATATGGTCTGAGAGACAAGACATTCAAGGCCTATTTTAAAATCCATTTTCTTTTCT

GTATCAAAAGTAAAATACAGCCATCAGACCTTGAGATTTAGTATGGAACCTAGAGAGATT

TTCTGATATGGCCATGACTTTTCTGTAGGCTGCTCTGGCCCAAGTTCAATTACATTTAAT

TAAGTTGTATTAAGTCCATTACAAATGACAGCTTTTGTGTTTCATGTTCATGTTCACCTG

TTTGTTTAAACTATGTGTGTATTCTAAGAACCCATTGCTGTATTCTGTAAGCTCTTTCAG

TTTATTTTTTGCCTCTGCTAGTTATGAGAATAAATATGTGTATGTATGTGTATATGTGTA

ATTTGAACTTTTACTATGCATTGCCTGGCTCCATTGATCCACATTGAATTGGTTGTCAAA

TGAGCTGAGTTGTAAGCAACTGCACTTCTGGACTACCATGACCTGGATGAATGAGAATCT

ACATAGACATTTGAATGCTTAGTTTTGACTGTGGTGTGTATTGGATAGGTTTAACCAATG

CAGGTTTACGTTGTACATAGTTTGTCATCATTTGTAGGCTTAAAAACAATACATTGCACT

TTACTGCCGATCATTCCTCTTGGTACTGCCGTAATTCAGAAATAAACATGTGCTCGTGCC

TTTTAAGTTCCTGTTCCACTGTGACAACAGCCTGCTTTGCCCTCAGTGGACGCCCGCCCT

GCAGTGGATCTGAGCACGGCTGAGCACAGGTTACGTTATGTTAGGATGTTAGTCAAATGA

GCACAGGTTACATTATGTTAGGATGTTAGTCAAAGGTTTGATTTCAATTTTATTTTGTAA

CGGCCGCCTTTTTAATGTAGCTGTTTGCTTAAAAACACAAAATTCTGGTAACTTCGTTAG

GTGATTAGGTGAGATCAGCATGCAGTGCTGGCCAAGAGTGTTGGCGCCCCTGCAATCCTG

TCAGATGGTGCTCAATTTCTCCCAGAGAATGTTTGCAATTGCAGGTGCTTTGGTAGTAAT

GTCTTCATTTGTGTTGCTTGCAATGGAGGAACACAAAAGAGAATGAAAAAAAAAGTCACA

TCAAT

>CL4703.Contig3_All CCCGGGTACAGATCATGGTGTTCTATGCATCTCAGACTATTTAATGTGAAAGTAGTACTT

ATGTGTCTGGCATCCTGGTTGTTTTTTCTCTTTCCTGTTTTTAACCTGGGCATATGTATT

TCATCCAAGTTCATACATGATGTGAAATACGATATGAGATTTTTATTTTCACTCCATTGA

GTCAAAGCTTTGTCACATGAGTAGGCATGGGTAGACTTCTGAAGTAATGATGCTTAATGG

TGCTTTTCAGCATCTAATATGTAAAGTATATGATTGTGAAGTGTTATATGATCTGTCAAA

AAGCATTGTTGATATTTTTTGTGTATCAATAATCTGATGATTTTTAAATTAGTTTTACAC

TTTCTGTCTTTTTAAACATGGTGTTATGCATTTAATAGATCTTGGGTAATTGTATAATTC

ATTAGTCATTGATAATAATGATATATCGTGTTTCTTTCAACTGACTTCATGATTTTGTGA

AGTTTTTGAGGAGGTCCATAGACGTAAATTAATTACAAATTTTGGGGATTAACATTTGGT

AATCTGTCAGAGACTGTCAGCAGTTCTCTAAAGCCATGCCGAACGTAACAGGATTCCCTG

CCCGCTTCCCCGCCGTCTTCTCTTCTCTTGAAGACTGGCTCATAGTGAAGGCCTTTTTTT

GTACGGCTCCCTGTGCCTTCTTCCTGTATGTGAATTTAGTCATGTTGTACACACTAAGGT

CTAAAGAAATCTTCACTGAAACCCCTCGCTACATACTGTTCAGTCACCTGCTCGTGTCTG

ACTCCCTGCAGCTGTGGTGCACCCTTGTGTACTATATACTGTACAACCAGACATTTGATC

AGGAGACTTTAAAAATAATAGGTGTTTACTGCTTTTTGAATTACCTTGTCTGTCGAATCA

CAAATAACTATCTTACCCCACTTTATCTAGGCCTTATGTCCCTGGAGCGTTATATTGCTA

TATGTTTTCCCCTTCGACATGCTGAGATAGCCAACAAAAGCAGGACCATGTTGGCCATAT

TATCTGTTTGGTTACTGGGCTTAATTCTCTGGACAGCTGACCTAGCTGCTGCCATGGTAT

TGCGGGGTAAGGAGGGGGGCTGTACTGACTACATAATCGCCCAAATGGTAGTTACCTATC

AGGTAAACACAGCATTAACAGGCCTTGTGTTTTCTTTGGTGAGTGTTGTTATAGTCTGTA

TATACATAGCCATTATGATAACAGCCAAGTCAAGTTCTACTGCTGACAAGTCCAAGGCGA

GTAAGGCCCACAAAACAGTACTCCTACACATGGTGCAGCTTTGCCTGTGTCTTTTGTCTC

TTCTATTCGGTGTGGTTCGCAGGGAGCTGGGTTTAAGCGGGCTGGACCCAGTGCTTGTTA

ATGAAGTGACGATTTTTTTGTTCTTAATGTTGAATATCTTGCCCAGGTGCCTCAGTCCTC

TTGTATATGGTCTGAGAGACAAGACATTCAAGGCCTATTTTAAAATCCATTTTCTTTTCT

GTATCAAAAGTAAAATACAGCCATCAGACCTTGAGATTTAGTATGGAACCTAGAGAGATT

TTCTGATATGGCCATGACTTTTCTGTAGGCTGCTCTGGCCCAAGTTCAATTACATTTAAT

TAAGTTGTATTAAGTCCATTACAAATGACAGCTTTTGTGTTTCATGTTCATGTTCACCTG

TTTGTTTAAACTATGTGTGTATTCTAAGAACCCATTGCTGTATTCTGTAAGCTCTTTCAG

TTTATTTTTTGCCTCTGCTAGTTATGAGAATAAATATGTGTATGTATGTGTATATGTGTA

ATTTGAACTTTTACTATGCATTGCCTGGCTCCATTGATCCACATTGAATTGGTTGTCAAA

TGAGCTGAGTTGTAAGCAACTGCACTTCTGGACTACCATGACCTGGATGAATGAGAATCT

ACTTTGACATTGAATTGGTCATGATGTCTAAATCTTTGGTTTGAGATTATCATGACTCGT

TACAGGTTCTGAAATGATTTTTATCACCTGTGTTCACTGTCGTGTAATTTGTCCGACATT

AGAAGTGTATCCAAATCCAATTATGAAGCACAACAAATGTAAAATAATAAAGCTTGTTTC

ATATACCTGTTGTCTGACAACTGACAACCTGATGCATTGTTATGAATGCAACAAAATATA

TGGAAAACAATGCCAGTTGCAGAAGGAAACCATAATGCAGAGGTCAGCGCTTGCAGTGAC

CACCAGCAATTTAATCCCTTAACAAGTATCCTCTCAGCTCATACCTAAGTAGGCCGCAGT

TACCAGAACATAATGATTGACAAGGCACCTTTGCTTATTTACCCAATTGTGCGACTTTAA

TAATTGATGAGCTAGCTAATGAGGTGACAGGGGGACTGGATTTAGAGGGCTGATCACCAA

ATGCACCCAAATGTTTAGCCTAAAAGGACACTCTAACACCATGGGGAGACGGCTCCTTAT

TTTATAGATTTAGCACTAATCACTCATCATTATAATAAACTTAAACTGCTGTCAGTGTTG

CGTACTTAATTAAAACATTGGGACAACAACAACAAATTGGGCTCTTGGGTTTTGGCCTTG

TGTACACCTGTGTACATAACAGCTGTTGTGGGTTGATCTTGGCAGCCATGTTAACAGGCT

ACAGAGCTTGTATGAGATGCACTGCAGTTGCATATTCACTAGATGGCGTGAGGTCTATTT

TTCACATGTCACGAGCAACAAGCTGAATGCTGGTACTGGCAACCCAGCAAATATGCTTTA

AAATGCTTTGTATTGTGAACAACAACATGCAACACGGTGCAGCAGAAGCAAACACAACTG

CCATCGTAACACTGATGTGCCTTGGGCTGTAGTTTTACATGTTCACACACACACACACAC

>CL5232.Contig1_All CGACAGTCTGGTCAAAGAGACAGACAAGAACCTTTAGGTAGGACCGTCAGGACTGTAAAT

GTGCCATGATGGGGGAAGCCGAAGGAGCCAATATTTCACATGCTGTGTTCATTTTCATTG

GTTTCCCAGAGACCTATGATCACAGGGACTGGTACGCAGTGCCTGTCCTCCTGAGCTACC

TGCTTCTCCTGGCAGGCAACTCCCTCCTGCTCCACGTTATCCACAGCACGGCCAGCCTTC

ACAGCCCCATGTACGTCCTGGTCTCCGCTCTGGCCATCGTCAACATCGTGGTTCCCACGG

CCATCATCCCCAAGATGCTGCTGGCGGTCCTGTTCGACCTCAGGGAGATCACCCTGGCGG

GGTGCCTGGTGCAGATGTTCGTGACGCACTTCTTCTCGTCTGTGGAGTCCACCATCCTCC

TGGTCATGGCCCTTGACCGGTATGTGGCCATCTGCCATCCGCTGCGCTATGTGGAGATTG

TCAACAGCGCCCTGTTCGTAAAGCTGCTCGTCTTCACCCTCGTCCGCAGCGGGTCGATCA

TGCTGACGCTGGTTGGCCTGGTGGCGCCCCTCAGGTTTTGTGGATCGAATGTTATCAGCC

ACTGCTACTGCGACCACATGGCCCTCGTTAGCCTTGCGTGCAACAGCACGGACAAAAACA

GCGCCATGGGGGTGGCCGTTATAGTGTGCTTTGTCGGCATCGACATATCGCTCATATTCT

TCTCCTATGTCAACATTCTGTACGTTGTCCTAAGGGCAGCTGCTGGGGAGGACAGGTGGA

AAGCCTTTCACACCTGCGGTACTCACCTGATGGTCATGATGAGTTTTTACCTGGTGGGCA

GTGTGACATTCCTCTCCCACAACCTTAACCTACCCCTCCCTGTGGATGTGAACACCTGCT

TAGGCTTGCTCTACATCATCTTCCCTGCCAGTGTCAACCCTGTCATCTACGGGGTTCGGA

CTAAAGAAATAAGGCACGCCATACTGAAAATTTTCAAGGTCCAGGCCAATAAAGTGTTTG

TGGTACAA

>CL5232.Contig2_All CGACAGTCTGGTCAAAGAGACAGACAAGAACCTTTAGAGACCTATGATCACAGGGACTGG

TACGCAGTGCCTGTCCTCCTGAGCTACCTGCTTCTCCTGGCAGGCAACTCCCTCCTGCTC

CACGTTATCCACAGCACGGCCAGCCTTCACAGCCCCATGTACGTCCTGGTCTCCGCTCTG

GCCATCGTCAACATCGTGGTTCCCACGGCCATCATCCCCAAGATGCTGCTGGCGGTCCTG

TTCGACCTCAGGGAGATCACCCTGGCGGGGTGCCTGGTGCAGATGTTCGTGACGCACTTC

TTCTCGTCTGTGGAGTCCACCATCCTCCTGGTCATGGCCCTTGACCGGTATGTGGCCATC

TGCCATCCGCTGCGCTATGTGGAGATTGTCAACAGCGCCCTGTTCGTAAAGCTGCTCGTC

TTCACCCTCGTCCGCAGCGGGTCGATCATGCTGACGCTGGTTGGCCTGGTGGCGCCCCTC

AGGTTTTGTGGATCGAATGTTATCAGCCACTGCTACTGCGACCACATGGCCCTCGTTAGC

CTTGCGTGCAACAGCACGGACAAAAACAGCGCCATGGGGGTGGCCGTTATAGTGTGCTTT

GTCGGCATCGACATATCGCTCATATTCTTCTCCTATGTCAACATTCTGTACGTTGTCCTA

AGGGCAGCTGCTGGGGAGGACAGGTGGAAAGCCTTTCACACCTGCGGTACTCACCTGATG

GTCATGATGAGTTTTTACCTGGTGGGCAGTGTGACATTCCTCTCCCACAACCTTAACCTA

CCCCTCCCTGTGGATGTGAACACCTGCTTAGGCTTGCTCTACATCATCTTCCCTGCCAGT

GTCAACCCTGTCATCTACGGGGTTCGGACTAAAGAAATAAGGCACGCCATACTGAAAATT

TTCAAGGTCCAGGCCAATAAAGTGTTTGTGGTACAA

>CL5445.Contig1_All AATTACGTTTAGGAAATATTATGAATCTACTGCTGGTGTAGAGCTATACTATCCGTGTGA

ATTTTTGTCACACACAATTTCTCATCTGCCTAAATCAATTTGTGGACAAACTTTGTTTTG

CTGTGTTTGTAAATTCACATATGTTAAACATCAGTGAGACATGTGTTAAACATCACATCA

GTCTTTTTTCTATGTCTGTGTTTATTCTGTGCTTTTTATGTTTTTTGCTCTGTGTATGTT

TCACATGTACATGAATACATTCCTGTTAACTATTTACTGAAAAGGATTGTGTTCCTATTC

CTAAATTTACTGAATTGTTTGCAGTATATCTGTTAGGACTGACCGGCTACAATGAATGGA

TCAACTATCATTTTGACTTTTACAGCCTATGAGGAGATTGGTCCAGCCAAAAATGTATTC

TTTGCCATCATATTCTTGATCTATCTGGCCTCTGTATTCACCAATACTGGTCTGATGCTT

TTAATTTATTTGGACACGGCACTCCATAAACCGATGTACATATTTTTGTTTGGCTTAATG

CTGGAAGGTTTGATTGGCAGCACCACAGTTTGGCCAACAGTTATGGCCAACCTAGCAACT

AACATCCACTCAACATCATACGAAGCCTGCCTTGTTCAGAGTTACTTCATCACAGTCTAC

GGGGGTTGTATGTACACTATGCTCACTGTCATGGCGTATGACAGGTATGTTTGCATCTTC

CAGCCTCTACAGTACCACACCATAATGACCCCACACAAGGTGAGAGTGCTTCTGGTTGCG

GCTAACCTCTTCCCTGTCTTACTGGTGGTGGGTCAGATATGCCTGACGTCAGGGCTGCCT

CTGTGCCATCACGCAATCCATAAAATATTCTGTGATAATTTATCTGTGTCTAATCTGGGA

TGTATTAAGACAACTTACGCTACGGTGACCGATCTTTATGGTGTATGTGCTCTCTTTTTC

TTTGTCGTACTCCCAGTGTTCCTCATCCTACTGTCATACTTCCGACTCATCCTGCTGACC

TCTAAGATTTCAGCAGATGCACGAAGAAAAGCATTCGCCACATGTGCTCCACATATAATT

ATATTTGTAAATTTCTCCATGTCTGTATTGTTTGCTGTAAGTTACAATCGTATAGCTTAC

TATGTACCAATAGGAGTTAATATATTTTTGTCATCCCTTTATGTTCTCATTCCTCCGCTG

TTTCACCCTATTTTGTACGGTATGAAAAACCAGGAGATCAAAAGAAGTCTCTCAAAACTT

CTGCGATCTGCGTACCTGGTTCTTGGCCTGCCCAAGCCAAAGAAATCGTCTGTAGCTTTG

GCCTTCTGAGTCAGACATGTTAGAGATGAAATACTGAGTAGTGTGCCTGTCTACTCATTG

CCAAACAAGGGGGAGAATATGCCTCAGACCCTTCCCACAGTGTTAGGCTCCTAATGCTTG

CTGCTGGATAGCTTTTGAAAACATGGCTTCGCCTCAGCTGTAATGACAAACAGCAAAGTG

AGTTTTAGATCATCTTTTTATTGTTATTTTTTTAAAGACAAGTCCCTGGTTTGTTACCTG

AGATTGAATAGTTCTCTTTAACACTGAATATGTGATGTTGTGCCTGATATAAAAATTCTA

GTGTTGTATGACAGACTATTCCAAATGTACCTGCTTATGTTTTTATCTAATGTTTGTATC

TTATCTGAGTAGGTAAGGAGGATGGTTACATAGTTTTGATGCACAGTATGAACAGGCCAA

ATAGTCATTCGCCAACCTGTACAGGAATATGTTTATTAACTCTGTGGAAGGTGGGTGGAT

ATACTAATGCCATTACTTAAAGACACTTTTTCACATGACGTTTTCAGTTGCC

>CL5445.Contig2_All AATTACGTTTAGGAAATATTATGAATCTACTGCTGGTGTAGAGCTATACTGTAAGTAGAC

ATAATATATGAGTGAGATCATTCTCTTCTTTTTGGAAAATCTATGTGTTGATTATTCCTC

ATAAAAATGTGTTTTTATGGTTTTGTGTATGGTTGGTTGTATGATTCTCACAGTGCTATT

TTCTTTCAGATCCGTGTGAATTTTTGTCACACACAATTTCTCATCTGCCTAAATCAATTT

GTGGACAAACTTTGTTTTGCTGTGTTTGTAAATTCACATATGTTAAACATCAGTGAGACA

TGTGTTAAACATCACATCAGTCTTTTTTCTATGTCTGTGTTTATTCTGTGCTTTTTATGT

TTTTTGCTCTGTGTATGTTTCACATGTACATGAATACATTCCTGTTAACTATTTACTGAA

AAGGATTGTGTTCCTATTCCTAAATTTACTGAATTGTTTGCAGTATATCTGTTAGGACTG

ACCGGCTACAATGAATGGATCAACTATCATTTTGACTTTTACAGCCTATGAGGAGATTGG

TCCAGCCAAAAATGTATTCTTTGCCATCATATTCTTGATCTATCTGGCCTCTGTATTCAC

CAATACTGGTCTGATGCTTTTAATTTATTTGGACACGGCACTCCATAAACCGATGTACAT

ATTTTTGTTTGGCTTAATGCTGGAAGGTTTGATTGGCAGCACCACAGTTTGGCCAACAGT

TATGGCCAACCTAGCAACTAACATCCACTCAACATCATACGAAGCCTGCCTTGTTCAGAG

TTACTTCATCACAGTCTACGGGGGTTGTATGTACACTATGCTCACTGTCATGGCGTATGA

CAGGTATGTTTGCATCTTCCAGCCTCTACAGTACCACACCATAATGACCCCACACAAGGT

GAGAGTGCTTCTGGTTGCGGCTAACCTCTTCCCTGTCTTACTGGTGGTGGGTCAGATATG

CCTGACGTCAGGGCTGCCTCTGTGCCATCACGCAATCCATAAAATATTCTGTGATAATTT

ATCTGTGTCTAATCTGGGATGTATTAAGACAACTTACGCTACGGTGACCGATCTTTATGG

TGTATGTGCTCTCTTTTTCTTTGTCGTACTCCCAGTGTTCCTCATCCTACTGTCATACTT

CCGACTCATCCTGCTGACCTCTAAGATTTCAGCAGATGCACGAAGAAAAGCATTCGCCAC

ATGTGCTCCACATATAATTATATTTGTAAATTTCTCCATGTCTGTATTGTTTGCTGTAAG

TTACAATCGTATAGCTTACTATGTACCAATAGGAGTTAATATATTTTTGTCATCCCTTTA

TGTTCTCATTCCTCCGCTGTTTCACCCTATTTTGTACGGTATGAAAAACCAGGAGATCAA

AAGAAGTCTCTCAAAACTTCTGCGATCTGCGTACCTGGTTCTTGGCCTGCCCAAGCCAAA

GAAATCGTCTGTAGCTTTGGCCTTCTGAGTCAGACATGTTAGAGATGAAATACTGAGTAG

TGTGCCTGTCTACTCATTGCCAAACAAGGGGGAGAATATGCCTCAGACCCTTCCCACAGT

GTTAGGCTCCTAATGCTTGCTGCTGGATAGCTTTTGAAAACATGGCTTCGCCTCAGCTGT

AATGACAAACAGCAAAGTGAGTTTTAGATCATCTTTTTATTGTTATTTTTTTAAAGACAA

GTCCCTGGTTTGTTACCTGAGATTGAATAGTTCTCTTTAACACTGAATATGTGATGTTGT

GCCTGATATAAAAATTCTAGTGTTGTATGACAGACTATTCCAAATGTACCTGCTTATGTT

TTTATCTAATGTTTGTATCTTATCTGAGTAGGTAAGGAGGATGGTTACATAGTTTTGATG

CACAGTATGAACAGGCCAAATAGTCATTCGCCAACCTGTACAGGAATATGTTTATTAACT

CTGTGGAAGGTGGGTGGATATACTAATGCCATTACTTAAAGACACTTTTTCACATGACGT

TTTCAGTTGCC

>CL5918.Contig2_All AGCTCAACTCAATGCTGTTACTAATGGTGTGGCCAAACCACTCTTACAGGGAACATCCAT

ACGCTACTTTCAGTAGAGATTATTAGTGGCATGTTGAATGTGGTCATGAGACCATGTTCA

GGACCGCACCGTGGCAGACGCTGTTCTACCAGGCTCTTAATGGCCTGTACTATGTCACTG

TGACACTGGTTATCCTCTACAGCTACATCAAGGTCGTGCTCGTGGCCCGGTCTGTGTCCA

GTGACCAGAAGTCAGCTGGGAAGGCACACAGGACTCTTCTGCTACACTTCATCCAACTGC

TGCTCTGCCTGAACACGCTTCTGTATGGCCATATTATCGGCTTCATGGCTGTGATGCTGA

GCTATGAGGTTTACTATGATGTGCGATACACAATCTATCTGCTCGTGATTCTGCTGCCCA

GGTGTCTCAGTCCCGTGATCTACGGCCTGAGAGACGAGGCTCTCCGCTGTGTGTTTATGT

ACTATTTCAAATGTGCCCTGTCTAAAGTCAAGCCTAGTGTAAATATGCACTGACTACATT

TATGTTCCTGCATTAAAAGGCAAAAGGCTGCAGTGTATTCCAGACTTACTACAACTTGCA

GAGGTGTGTGTGTGTGTGTG

>CL6606.Contig1_All TGTATCTACATGTAACAACATGTAATATTATTGTATGTACTTTGTGTATGTACTGTATGT

ATTTACACTGTACGTAACAAACACTAACCCTAACCCTTTGGTACAGTGTAAGTACATAAT

GTTATATATTGTTACACTATACCTATTTGATGTAACAGAAACAGTTAACTAAGTGTAACT

GCAATTTCTTTTAAAAGACATTTATTTAATATAAATTAACATATTTTATTTTAGGTTGAA

GATTCATGAGAATCCATCAGCAATGTCTCTCAATTCATCAGGTGCAACAACTATGCTAAC

TTTACCTCCATTCTCCTTTCCTTTGTCAGCCAGAGTTCCAGCTCTTGTTTTTTCAGCCCT

TACATATCTGACCATTATTTGTTGCAATCTGATGGTTGTTGTGACTATTGCTATTAACCA

TAACCTGCACAAGCCCATGTATATGCTACTTATTAATTTACCTGTGTGTGATGTGATGGG

AGCGACTGCCTTTTTCCCTCAGATGATTTCCAGCATGCTGTATGATCCCAGAGTTATCCC

ATATTGGGCTTGTGTAACACAGGCTTTATTTGTGCACACGTATGGTGCTGGATCACTTCT

CATATTGACAGCCATGGCCCTTGACCGCTATGTAGCTATCTGCCAACCGCTTTCTTACAA

CACCATAATGACTAATAGTAATCTGATTAAGATGATAAGTGCTGTTTGGCTTACAACCAT

GATTTTGATGTTTATCCTCATCGTGTTGGCACTCCGTTTGAAGATTTGCAGGACCAATAT

CAGTGACATGTATTGTACCAACCCCTCGTTGACAAACCTGGCCTGTGAATTGACATACGT

GAATAACGTCTATGGTTTGCTTACTATTGCCTTTTTTTCAGGGAATATCACTGTGTGCAA

TTCTGTTCACATATTTACAAATCCTAATCACTTGTATTTTCAAGAAACAGTCAGATGCCA

GAAGTAAAGCGATTCAGACATGTGGTGCCCATTTAATAGTTTTTTTATTCCTGGAATGCA

ATGCATTTTTCACTTTACTTTCACATCGCTTTGAAAGTGCTCCCACTTTCTTACGCAGAG

CCTTGGGTGTGTCTGTGATGGTATTTCCTCCTTTACTGAATCCATTGATATATAGCTTTA

ACACAAAAGACATCAGAAGACACATATTGATATTCCTTAAAAGAAAAACATTTGCTTCCT

AATTTAAACTATGAAGGTGTTATTTGTTGATTATAGAAACAAACTATCTCCATTTGAAGA

ATTTAACTGTCTTACTGTCTGTATAAACCCATATCTGTCAGCACTGTACATGTAGCATTA

AACATTTCATTTAAGCAGATAACATTTAAATGACAAGAAGGTTCAATGTATATGGCAATG

GTTTTACGACATTTATTTTGTGCAGTGATGCAATAATTAAAAATCATGCTGTGGCCTAAA

AATGT

>CL6606.Contig2_All ATGATGGGAGCGACTGCCGTTTTCCCTCAGATGATTTCCACTATGTTGTATCATCCAAGA

GTTATCCCATATTGGGAGTGTGCTCTGCAGGCATTATTTGTGCACATGTATGGTGGTGGA

AATCTTATGATATTGACAGCCATGGCCTTTGACCGCTATATAGCTATCTGCCAACCACTG

TCTTATAATACCATAATGACCAATAGTAACCTAATTAAAATTATATGTGCTGTTTGGCTT

ACTGATTTCGTTTTGATATCTATCTCAATTTTGTTGCTATTACGACTGAAGAATTGCAGC

AGTGAAATCAATGATATGTTTTGTACTAACCCCTCATTAATGAAACTAGCTTGTGGAGAC

ACAAATGTGAATAACTTCTATGGTCTGTTTACTATTGCTTTCTTTCAGGGGGTATCATTG

TGTGCAGTTCTGTTTACATATTTACAAATCCTCATCACTTGTGTTTTTAAGAAACAGTCA

GAGGCCAGAAGTAAAGCGCTTCAGACGTGTGGTGCTCATTTAACAGTTTTTTTATTTTTT

GAATTTAATGTATTTGTTTTCTTCCTTGCACACCGTATTCAAACCGCTCCCCTTTTTGTA

CGCAGGGCATTGGGGGTTTCTGTAACGATATTTCCACCACTGTTAAATCCACTGATATAT

GGTATCAATACAAAAGATATAAGGAGAAATATTGTTGTAATACTTAAACGAAAAATAGTA

CCCTTTTAATTTAAATTTAAGATTTTTTGTTGATTACAGTCATCGTAATATCGCATATAT

GTAAAGAATTCTATTTATTATTGCTGTAATTATATATCTGTCAGCATTGTTCATAC

>CL6606.Contig3_All GCACACACACACACACACACGTGAAAAAAGTACTGTGCCTATGTAATGTAAACCGATGAG

GGAGCAATAATGGGTGTTACCTGGTTGACTTGGCTGTATATATTTAGCTCACACAGACCT

AGAGGACATTAAGTCACTGGAGGATGTAAAGAGAGGTATCTTTTAACACCGGCGTAATAC

TTCTGTATTCAAACACAGCACTGCTCATTGTATGTTGCAGGGTTATTTGGAAATAATATG

GACATAATGCAGTCAGTTATGTGTTTAAGAGGGTGATTAATATGAATTAAATGAATATTA

ATTATTTGTTGTTTTGCATTAATATGTTTTTGTTTACTTTTTAGGTTGGGCATTTAAAAG

GACCTAATACATCAGCCATGTCTCTTAACTCATCAGGCGCAATGACTTTGTTAACATTAC

CTCCATTCTCCTTTTCTTTGTCAGGCAGAGTTCCAGCTCTTGTTTTTTCAGCCCTTACAT

ATCTGACCATTATTTGTTGCAATCTGATGGTTGTTGTGACTATTGCTATTAACCATAACC

TGCACAAGCCCATGTATATGCTACTTATTAATTTACCTGTGTGTGATGTGATGGGAGCGA

CTGCCTTTTTCCCTCAGATGATTTCCAGCATGCTGTATGATCCCAGAGTTATCCCATATT

GGGCTTGTGCAACACAGGCTTTCTTTGTGCATACGTATGGTGGTGGGTCACTTTTCATAT

TGACAGCCATGGCCCTTGACCGCTATGTAGCTATCTGCCAACCGCTTTCTTACAACACCA

TAATGACTAATAGTAATCTGATTAAGATGATAAGTGCTGTTTGGCTTACAACCATGATTT

TGATGTTTATCCTCATCGTGTTGGCACTCCGTTTGAAGATTTGCAGGACCAATATCAGTG

ACATGTATTGTACCAACCCCTCGTTGACAAACCTGGCCTGTGAATTGACATACGTGAATA

ACGTCTATGGTTTGCTTACTATTGCCTTTTTTTCAGGGAATATCACTGTGTGCAATTCTG

TTCACATATTTACAAATCCTAATCACTTGTATTTTCAAGAAACAGTCAGATGCCAGAAGT

AAAGCGATTCAGACATGTGGTGCTCATATAATAGTTTTTTTATTCTTGGAATTGAATGCA

TTTATCGCTTTACTTTCACATCGTATTGCAAGTGTACCCACTTTCGTACGCAGGGTATTG

GGGGTTTCTGTAATGATATTTCCTCCTCTATTGAATCCACTGATATATGGCTTTAATACA

AAGGATATCAGAAGAAACATATTGGCTTTCTTTAAAAAGAAAACATCACCCTTTTAATGT

AACCTGCAAAAGTGTTCTTTGTTATTAATAAACAGACACCTTAATATAAAAAATATTAGT

GCTTGTTATTCTTGATGTGGACATGTACCTACCAACACTGTATGTGCAGCTTTATGCTTT

AAATGAAAATTATGACATTTAAACTACATACAGTGTATGGTTATTAAGTTATATCCACTG

ACTTTATGAAAAATATTTTTCACAGTGACGCCATGTTTTCTAAACCTGTGAACTTCCTTA

AACCTTTCACTAATAAAGCATAATTTAATGTTTAAA

>CL6688.Contig1_All CCAGCATTTCACTGTCACTCTCTCCTCAGACCTTGACATGTACCAACATGCAGAGCCAGT

GTCCAATTTCACTCTACTCTTCACTGGCTATGGTCCCCAGGGTCCAACGCAATATGGGGT

ATTGTTCATCACCCTGTTGCTTTACATTGGTACAATTGTATCTAATGTTGCCATACTGTT

GGTGATCTATTTTGATTCAAGTCTGCACAAACCTATGTACATATTTCTATTTAACTTGGC

TATCAATGGAGTACTCGGCAGCACTGCAGTGTGTCCAAAGATCATGGCTAATCTCCTGAA

AGACAATAACTACATATCTGTTGAGGGCTGCTTGACTCAGGTTCTTTCCATTAACATTTA

TGCCAGCTGTGCATATGCGATTTTTGCAGGGATGGCTTATGATCGCTATGTGTGTATATG

TAAACCACTCCAGTATCACAGTATAATGACCCCTTCTCGAGTGAAGGTGCTTCTGGCCTT

AATCTACATCCTTCCAGTATCCCTGTTGTCTGTCCAGGTGTATTTGACCTCTCGACTTCC

ACTGTGTAGGCATACCATCAATAAGCTCTTTTGTGACAATCTGGCTATTGTTAATCTGTC

CTGCGTAAAAGATGTCATTGGTAATTTGTATGGTGTTTGTCTGGTGTTTGTGCTTGTTGT

TCTTCCTCTGTTTCTAGTCATTCTGTCCTATGTTAAGATATTACATGTGAGCCTAAAGGC

ATCCGAGAATGCGCAACAGAAAGCACTGCAGACCTGTGCACCTCATTTGATTACATTTCT

TAATTTTTCTGTCGCCATCCTCTTCTCTGTTATCTACAACAGAATTAGTTACTACCTCCC

TCCGGAAGTTAATATATTAATCTCATTAGATTTTATCTTGTTACCCCCCCTTTTGCATCC

CCTGATTTATGGTTTCAAAACTCAGGATATTAGAAAAAGCCTGTATAAAATCTTCAGAAG

AAGAGTATCAATCAACTCTGAGTTCGACTCGTCAGATCAGAATGTGAGCAGGTGTAAAAC

AGTTTTCTTTTGAGTGATCATCAACGCACCTTGTCCAAGTCAAAGGATGCTGTGTAGAAA

GATTATTCGGTATGCTTAACAGAATGTCTGCAATTCCTTCTGCACTCCAGTGTGGGGTCA

ACCTGAGTGAAATGCATGTTATGTGAAATGTTCTGATGAAACAGAGAAAGGCTTTTGACA

GTTATTTGTTTTAACACATTTACTTGCACTTAATTTGATATGTCATCAATATCTCACCTG

GTATCAATCACAGATGATGACCCATGGTCATTGATGGAACATGCAAGTAGCCTGACAAAG

TGTCCTCCTGCTCACAAAAGTCTGTAACTACACATATGCATATATTTTAAAGAATCAAAA

TTGATTGTAAGTTAAGGCTGGGTGGCATGTCAAAAATGTGTTTATGATACATTTTCACAT

ATTGTTTAATCACCATATTTATAGCAGTGTTTTTTTTTGCTGATACTGCCAGTTCAGACA

GCATTCTGAGTAAAGCCTAATTTAAATCACTGCTTCAATCAGTCAAGCCATGAAATATGA

TTATATGAAAGTAAACAACGTTGTATATTCTGTTGGCCATAAGCCCAGGAGCTAAGGCCC

AGTGTGAGGGGAAACGCTCAGCAGCATTTCCACTGTGCCACGTGGAATTAACAGAGTTTG

CTGCCACAGAGACACAATGTAAACTAGGCACAATAGGTAACAACATTTACTTGGTAGATT

CCTTTGTTATAACATGCTCCTTAGCAACAACTAATACCTTTGGAAAGCTTCTATATTTCC

CATTTAAAACCATGTAGAATGTTTTATATAGGCTAAAATTAATGTACAGAAAACAACAAA

ACAAATGTTGACAGAACAAAGCATGCAATATTTTTGCCAGTGGTGGATTGTCTTTGCATT

GTCTTGAGTTTGCCATTATAAAAAATAAAAAGTAAGTCACATAAGGACTAAGGACAGTGC

CATCAGTATTGCAAATGGGGACAGAAAGAAAGAGAGACATTGAGAAAGACAGGTAAGGCA

AGTTTATTTATATAGTGCATTTCATACAAAAAGGTAGTTAAATGTACTTTACAAAAACAA

AGACAACAAACAGTAAGTTATAAAAAGTATGAAACATTAAGGATACAAAACAGCATACAT

TAAAAAAAGGACAGACAATAAGAAACTAGATGCACAGTGTGCTATGAAGTTCAGCACAAA

GGAAATTATAGTGCGCCCTATGGAATGAATGCCCATTGTGTAGTTATAAGCAAAAATGCT

ACTTTTTGAAATTAAACACCTTGCGGGGGCGCAG

>CL6688.Contig3_All CCAGCATTTCACTGTCACTCTCTCCTCAGACCTTGACATGTACCAACATGCAGAGCCAGT

GTCCAATTTCACTCTACTCTTCACTGGCTATGGTCCCCAGGGTCCAACGCAATATGGGGT

ATTGTTCATCACCCTGTTGCTTTACATTGGTACAATTGTATCTAATGTTGCCATACTGTT

GGTGATCTATTTTGATTCAAGTCTGCACAAACCTATGTACATATTTCTATTTAACTTGGC

TATCAATGGAGTACTCGGCAGCACTGCAGTGTGTCCAAAGATCATGGCTAATCTCCTGAA

AGACAATAACTACATATCTGTTGAGGGCTGCTTGACTCAGGTTCTTTCCATTAACATTTA

TGCCAGCTGTGCATATGCGATTTTTGCAGGGATGGCTTATGATCGCTATGTGTGTATATG

TAAACCACTCCAGTATCACAGTATAATGACCCCTTCTCGAGTGAAGGTGCTTCTGGCCTT

AATCTACATCCTTCCAGTATCCCTGTTGTCTGTCCAGGTGTATTTGACCTCTCGACTTCC

ACTGTGTAGGCATACCATCAATAAGCTCTTTTGTGACAATCTGGCTATTGTTAATCTGTC

CTGCGTAAAAGATGTCATTGGTAATTTGTATGGTGTTTGTCTGGTGTTTGTGCTTGTTGT

TCTTCCTCTGTTTCTAGTCATTCTGTCCTATGTTAAGATATTACATGTGAGCCTAAAGGC

ATCCGAGAATGCGCAACAGAAAGCACTGCAGACCTGTGCACCTCATTTGATTACATTTCT

TAATTTTTCTGTCGCCATCCTCTTCTCTGTTATCTACAACAGAATTAGTTACTACCTCCC

TCCGGAAGTTAATATATTAATCTCATTAGATTTTATCTTGTTACCCCCCCTTTTGCATCC

CCTGATTTATGGTTTCAAAACTCAGGATATTAGAAAAAGCCTGTATAAAATCTTCAGAAG

AAGAGTATCAATCAACTCTGAGTTCGACTCGTCAGATCAGAATGTGAGCAGGTGTAAAAC

AGTTTTCTTTTGAGTGATCATCAACGCACCTTGTCCAAGTCAAAGGATGCTGTGTAGAAA

GATTATTCGGTATGCTTAACAGAATGTCTGCAATTCCTTCTGCACTCCAGTGTGGGGTCA

ACCTGAGTGAAATGCATGTTATGTGAAATGTTCTGATGAAACAGAGAAAGGCTTTTGACA

GTTATTTGTTTTAACACATTTACTTGCACTTAATTTGATATGTCATCAATATCTCACCTG

GTATCAATCACAGATGATGACCCATGGTCATTGATGGAACATGCAAGTAGCCTGACAAAG

TGTCCTCCTGCTCACAAAAGTCTGTAACTACACATATGCATATATTTTAAAGAATCAAAA

TTGATTGTAAGTTAAGGCTGGGTGGCATGTCAAAAATGTGTTTATGATACATTTTCACAT

ATTGTTTAATCACCATATTTATAGCAGTGTTTTTTTTTGCTGATACTGCCAGTTCAGACA

GCATTCTGAGTAAAGCCTAATTTAAATCACTGCTTCAATCAGTCAAGCCATGAAATATGA

TTATATGAAAGTAAACAACGTTGTATATTCTGTTGGCCATAAGCCCAGGAGCTAAGGCCC

AGTGTGAGGGGAAACGCTCAGCAGCATTTCCACTGTGCCACGTGGAATTAACAGAGTTTG

CTGCCACAGAGACACAATGTAAACTAGGCACAATAGGTAACAACATTTACTTGGTAGATT

CCTTTGTTATAACATGCTCCTTAGCAACAACTAATACCTTTGGAAAGCTTCTATATTTCC

CATTTAAAACCATGTAGAATGTTTTACAACAAAACAAATGTTGACAGAACAAAGCATGCA

ATATTTTTGCCACTGGTGGATTTTCTTTGCACTGTCTTGAGTTTGCCATTATAAAAAATA

AAAAGTAAGTCACATAAGGACTAAGGACAGTGCCATCAGTATTGCAAATGGGGACAGAAA

GAAAGAGAGACATTGAGAAAGACAGGTAAGGCAAGTTTATTTATATAGTGCATTTCATAC

AAAAAGGTGTGCATTTCATACAAAAAGACAGTTAAATGTGCTTTACAAAAACAAAGACAA

CAAACAGTAAGTTATAAAAAGTATGAAACATTAAGGATACAAAACAGCATACATTAAAAA

AAGGACAGACAATAAGAAACTAGATGCACAGTGTGCTATGAAGTTCAGCACAAAGGAAAT

TATAGTGCGCCCTATGGAATGAATGCCCATTGTGTAGTTATAAGCAAAAATGCTACTTTT

TGAAATTAAACACCTTGCGGGGGCGCAG

>CL6688.Contig4_All CCAGCATTTCACTGTCACTCTCTCCTCAGACCTTGACATGTACCAACATGCAGAGCCAGT

GTCCAATTTCACTCTACTCTTCACTGGCTATGGTCCCCAGGGTCCAACGCAATATGGGGT

ATTGTTCATCACCCTGTTGCTTTACATTGGTACAATTGTATCTAATGTTGCCATACTGTT

GGTGATCTATTTTGATTCAAGTCTGCACAAACCTATGTACATATTTCTATTTAACTTGGC

TATCAATGGAGTACTCGGCAGCACTGCAGTGTGTCCAAAGATCATGGCTAATCTCCTGAA

AGACAATAACTACATATCTGTTGAGGGCTGCTTGACTCAGGTTCTTTCCATTAACATTTA

TGCCAGCTGTGCATATGCGATTTTTGCAGGGATGGCTTATGATCGCTATGTGTGTATATG

TAAACCACTCCAGTATCACAGTATAATGACCCCTTCTCGAGTGAAGGTGCTTCTGGCCTT

AATCTACATCCTTCCAGTATCCCTGTTGTCTGTCCAGGTGTATTTGACCTCTCGACTTCC

ACTGTGTAGGCATACCATCAATAAGCTCTTTTGTGACAATCTGGCTATTGTTAATCTGTC

CTGCGTAAAAGATGTCATTGGTAATTTGTATGGTGTTTGTCTGGTGTTTGTGCTTGTTGT

TCTTCCTCTGTTTCTAGTCATTCTGTCCTATGTTAAGATATTACATGTGAGCCTAAAGGC

ATCCGAGAATGCGCAACAGAAAGCACTGCAGACCTGTGCACCTCATTTGATTACATTTCT

TAATTTTTCTGTCGCCATCCTCTTCTCTGTTATCTACAACAGAATTAGTTACTACCTCCC

TCCGGAAGTTAATATATTAATCTCATTAGATTTTATCTTGTTACCCCCCCTTTTGCATCC

CCTGATTTATGGTTTCAAAACTCAGGATATTAGAAAAAGCCTGTATAAAATCTTCAGAAG

AAGAGTATCAATCAACTCTGAGTTCGACTCGTCAGATCAGAATGTGAGCAGGTGTAAAAC

AGTTTTCTTTTGAGTGATCATCAACGCACCTTGTCCAAGTCAAAGGATGCTGTGTAGAAA

GATTATTCGGTATGCTTAACAGAATGTCTGCAATTCCTTCTGCACTCCAGTGTGGGGTCA

ACCTGAGTGAAATGCATGTTATGTGAAATGTTCTGATGAAACAGAGAAAGGCTTTTGACA

GTTATTTGTTTTAACACATTTACTTGCACTTAATTTGATATGTCATCAATATCTCACCTG

GTATCAATCACAGATGATGACCCATGGTCATTGATGGAACATGCAAGTAGCCTGACAAAG

TGTCCTCCTGCTCACAAAAGTCTGTAACTACACATATGCATATATTTTAAAGAATCAAAA

TTGATTGTAAGTTAAGGCTGGGTGGCATGTCAAAAATGTGTTTATGATACATTTTCACAT

ATTGTTTAATCACCATATTTATAGCAGTGTTTTTTTTTGCTGATACTGCCAGTTCAGACA

GCATTCTGAGTAAAGCCTAATTTAAATCACTGCTTCAATCAGTCAAGCCATGAAATATGA

TTATATGAAAGTAAACAACGTTGTATATTCTGTTGGCCATAAGCCCAGGAGCTAAGGCCC

AGTGTGAGGGGAAACGCTCAGCAGCATTTCCACTGTGCCACGTGGAATTAACAGAGTTTG

CTGCCACAGAGACACAATGTAAACTAGGCACAATAGGTAACAACATTTACTTGGTAGATT

CCTTTGTTATAACATGCTCCTTAGCAACAACTAATACCTTTGGAAAGCTTCTATATTTCC

CATTTAAAACCATGTAGAATGTTTTACAACAAAACAAATGTTGACAGAACAAAGCATGCA

ATATTTTTGCCACTGGTGGATTTTCTTTGCACTGTCTTGAGTTTGCCATTATAAAAAATA

AAAAGTAAGTCACATAAGGACTAAGGACAGTGCCATCAGTATTGCAAATGGGGACAGAAA

GAAAGAGAGACATTGAGAAAGACAGGTAAGGCAAGTTTATTTATATAGTGCATTTCATAC

AAAAAGGTAGTTAAATGTACTTTACAAAAACAAAGACAACAAACAGTAAGTTATAAAAAG

TATGAAACATTAAGGATACAAAACAGCATACATTAAAAAAAGGACAGACAATAAGAAACT

AGATGCACAGTGTGCTATGAAGTTCAGCACAAAGGAAATTATAGTGCGCCCTATGGAATG

AATGCCCATTGTGTAGTTATAAGCAAAAATGCTACTTTTTGAAATTAAACACCTTGCGGG

GGCGCAG

>CL6914.Contig1_All

TAACAGTGCACAGCCGGGAGGAAGGCACCACACTGCTTTCATCAACATATGAGGACACAT

ACATCTTGTTTCTGAATAACATTTAACTGAAAACTTATTTTTAAGAATTTGAACCTGTTT

CTGTTGCAGGATTTTACATCCCTTACAGCACTGGTGATGGAGAGTAATGTATTGAATCAA

ACCTTTTCATATGAGCTTAAAATTGCCAGCTTTGACATTCCCCCCTCAGCTGTCTACCCT

ATTTTTATAATAGGCATGTTGATATACCTCTTCTCTGTTCTCTCTAATCTGACCATCCTG

CTGCTGATTGCTACTCAGAAGAGTCTCCACAAACCCATGTTTTACATCCTCTTCAGCCTG

CCACTGAATGACCTTGTTGGAATAACTGTAATGTTCCCTCGTATGCTGATTGATATTGTG

ACCAAGAGATACACTGTGTATTACCCAACCTGTGTTTTACAGGGATTTCTGCTCCATATG

TTTGCTGGTGGCACACTGTTTGTATTGGCGGCAATGGCATTTGACCGGTACATTGCTATT

TGCAAGCCACTGAGGTACCATGCCATCATGACTCCTGTAACTGTTGCAGGGATATTGGCA

TTGGCATGGGGTACGGACTTTGCCTTAATATTAGTCCTTTTTTTTCCTTCAGGCCAGAGT

GCGTAGGTGCAGAATTTTTATATTAAATGTGTTCTGTAGCAATGTCACATTGCTCAATCT

GTCCTGTGGAGAGGACACCACAATCAATAATATCTATGGATTGTTTATAACAGCATTCAT

GCAGATTGTCACTATAGCAGTACAGTTGTTCTCGTATATTCAAATCCTGTTAACGTGTGT

CTTTAACAAACAATCTAATGCCAAAACAAAAGCTGTGAACACCTGCCTGGCACAGATAAT

GGTGTTTCTGATTTTTGAATTTATTGGTATGTTCGGAATTCTGTCCTCCCGGTTTCCAAA

TGTACCTACAAATGCAAGAATGGTCATTGGCATTATGATTTATGTTGTCCTGCCTGTTAT

CAATCCTATTATTTATGGAATGAAAACTAACGATATTAGAATAGCATTCCTGCATGTGGT

AAAGATAAAGAAGTCTTCATTAACATAATATTGTCAATTGTAAAATCTACGTGTATGGCC

TGATTACGTTTGTTTTTGTAAAAGTTAAGATTCATGAGTCACTGTAGTTTTTCCCATAAA

TCCAAAATGGTCCCTGGTCGATCTTAGCATTTGACTGTACAGTTTTAGTGTGTCTGGTAT

GCCTGGTCTCACCTCACATGCAGGCCATGATGTAACAGCCATTAGCCGGCAACCCTCTTT

TGCTCCTCTGTCTTCTAATTGACACATACAACTTCACAGCTCACACTGATCAACTTAAAG

GGGCCATATTATGCGAAAAGTTTTTGCATGTTTTTGTATTGTCATTTGGGTCTCTACTGC

TAGTATAAACACTCCAAATGCCAAAAAAA

>CL6914.Contig2_All

GGATTTTACATCCCTTACAGCACTGGTGATGGAGAGTAATGTATTGAATCAAACCTTTTC

ATTTGAGCTTAAAATTGCCAGCTTTGACATTCCCCCCTCAGCTGTCTACCCTATTTTTAT

AATAGGCATGTTAATATACCTCTTCTCTGTTCTCTCTAATCTGACCATCCTGCTGCTGAT

TGCTACTCAGAAGAGTCTCCACAAACCCATGTTTTACATCCTCTTCAGCCTGCCACTGAA

TGACCTTGTTGGAATAACTGCAATGCTTCCTCGTGTGTTAGTGGACATTGTGATCATAAA

AAACACGGTCAGCTACCCTGCCTGTGTCCTTCAGGCATTTCTGCTCCATATGTATGGTGG

TGCTATCC

>CL6921.Contig1_All GCTTCTTTGCCCTATTACCTTAAGCCATAGTAGAATGGATTTAGCCTAAAATAGAAACTT

AATATAAGGTAGAAAATATGTCCATCCAGTTATAGTCACAGTTCAGTTAACTTTTGAAAA

CGTATTCCTCTTTAGCAAATGAATACATTTCTACCCACAAGGAATGAACACATCCATCCT

CTCTATGACTCTGATTTATGTAACCTATAAGGAAATGGGCTCAGCCAAGAATGTATTTCT

CATTTTAGTGCTAACCATTTACCTTGCCTCTGTAATAGCCAGTGGCACTGTTATGTTGTT

GATCTATATGGATACTAGCCTCCATAAGCCAGTGTACATATTCCTATTTAGTTTAATTGT

AAATGGCATCATAGGGAGCACTGCTGTTTGGCCCAAGGTAATGAACATTCTCTTAACTGA

CGACAATACTGTCTCATATGAAGGATGTCTTGTGCAAGTTTTCCTAACAGGAAGTTACGG

GGCCTGCAACTATACAATGCTGACTGTCATGGCTTATGACAGATTTGTGTTCATCTTTAA

GCCTCTGCACTATCACACTGTTATGAACCCTTACAGGGTGAAGCAACTTGTATTGATTGG

AAACTTAATACCTCTAACAGTTATGCTTACTCAGATATACCTTGCTACAAGGCTATCTCT

GTGCAAGTATACAATACAGAGGGCTTTTTGTGATAATGTATCTGTAATTGATCTATCCTG

TGACAATGACAACTTCAGTCTGGTATGCAGTGTGTTTGGTGTATGTGCTATTTTTTGTCT

TGGTGTCCTACCTATGTTTCTTGTTATACTGTCATATTTAAAAATCATATTAACCATTTT

GAAAATGTCCACAGATGCCAGAAAGAAAACATTTGCCACATGTTCTCCACATTTGATTGT

TTTCATACTTTTCTCATTTGTGTCTCTGTTTTCTATCATTTATAATCGTGTTTATCCTGA

TGTATCAGTAAGGGCTAAAGTTATCAGACCGTTTTTTCTCGCTACTAATTATATTCTCAT

CCCGCCTTTTGTTCAACCAGTTATATATGGTTTGAAAAGTCAAGAAATCAGGCAAAGCTT

TCTTAAGTTTAGAAAAAGGGCTATTTTAGGATTTCAGCTCAGTTTCAAATGATGAAAACC

ATTCATACTTTTATGTAACAGACAGTAACAATGTGCATTAAGTATGTACTTGCAGTGTGT

CTGTTCCCTGCCAACATGGAGGGACTGGACGTTTCACACTCTGACCTGAGAGCCGGAGTG

AATTTATCTGGCTGGAACTGATGGAATACAGTTAGTGTTTGTGGTCAGATCATCAAGCAT

CAAATTTGCCATTTAGCATGATAGTCAAAGGGTGGAGCTGTGTCATGTGTATACTAGCAC

AATCCTTGTGTGTGATATGTTTTAATAAAAAAGATAATTTAATGGTGTATAATGAGTTCA

CTTCACTATCTGTGATTTGTTTTCATGAATAATGAGAGTTTATCACAAACATGAATTAAT

TCACTATTAATTCACTTTAATGATATTGTTGCTAATCTGAGAACAAGCTTTTCTTTTATG

TCAAAAAAACAATCAGTGCTAGAACACGTGTATCCAACTATTAAGACCACATGCTGTGTC

AACCACCACTATGATGACATCAAAGTATACAATCAATGAGAATGTCTTTGTAATTGTACT

TGCAAATAAATAAAGAAAG

>CL6921.Contig3_All

GCTTCTTTGCCCTATTACCTTAAGCCATAGTAGAATGGATTTAGCCTAAAATAGAAACTT

AATATAAGGTAGAAAATATGTCCATCCAGTTATAGTCACAGTTCAGTTAACTTTTGAAAA

CGTATTCCTCTTTAGCAAATGAATACATTTCTACCCACAAGGAATGAACACATCCATCCT

CTCTATGACTCTGATTTATGTAACCTATAAGGAAATGGGCTCAGCCAAGAATGTATTTCT

CATTTTAGTGCTAACCATTTACCTTGCCTCTGTAATAGCCAGTGGCACTGTTATGTTGTT

GATCTATATGGATACTAGCCTCCATAAGCCAGTGTACATATTCCTATTTAGTTTAATTGT

AAATGGCATCATAGGGAGCACTGCTGTTTGGCCCAAGGTAATGAACATTCTCTTAACTGA

CGACAATACTGTCTCATATGAAGGATGTCTTGTGCAAGTTTTTCTAACAGCAAGTTACGG

GACCTGCAACTACACAATGCTGACCGTCATGGCTTATGACAGATTTGTATTCATCTTTAA

GCCTCTGCACTATCACACTGTTATGAACCCTTACAGGGTGAAGCAACTTGTATTGATTGG

AAACTTAATACCTCTAACAGTTATGCTTACTCAGATATACCTTGCTACAAGGCTATCTCT

GTGCAAGTATACAATACAGAGGGCTTTTTGTGATAATGTATCTGTAATTGATCTATCCTG

TGACAATGACAACTTCAGTCTGGTATGCAGTGTGTTTGGTGTATGTGCTATTTTTTGTCT

TGGTGTCCTACCTATGTTTCTTGTTATACTGTCATATTTAAAAATCATATTAACCATTTT

GAAAATGTCCACAGATGCCAGAAAGAAAACATTTGCCACATGTTCTCCACATTTGATTGT

TTTCATACTTTTCTCATTTGTGTCTCTGTTTTCTATCATTTATAATCGTGTTTATCCTGA

TGTATCAGTAAGGGCTAAAGTTCTTAGACCGTTTTTTCTCGCTACTAATTATATTCTCAT

CCCGCCTTTTATTCAACCGGTTATATATGGTTTGAAAAGTCAAGAAATCAGTAAAAGCTT

TCTTAAGTATAGAAAAAGGGCTGTTTTGGGATTTTAGCTCAGTTTCAAATGATGAAAACC

ATCCATACTTTTATGTAATAGACAATGTAAATGTGCATTAAGTGTGTACTTGCAGTGTGT

CTGTTCCCTGCCATATTAGCTATGTTATCCAGCTCTCCTTCAGCTCATGGGCAACACGGA

GGGACTGGACGTTTCACACTCTGACCTGAGAGCCGGCCGTGTACTTACTCTGATGACCTT

AGCGCGGCTGAGTTGAGTGAATTTATCTGGCTGGAACTGATGGAATACAGTCCTTCATGA

CTGCTTTAGGACCTCAACTACAAGGTAGTGTTTGTGGTCACATCATCAAGCATCAAATTT

GCCATTTAGCATGATAGTCAAAGGGTGGAGCTGTGTCATGTGTATACTAGCACAATCCTT

GTGTGTGATATGTTTTAATAAAAAAGATAATTTAATGGTGTATAATGAGTTCACTTCACT

ATCTGTGATTTGTTTTCATGAATAATGAGAGTTTATCACAAACATGAATTAATTCACTAT

TAATTCACTTTAATGATATTGTTGCTAATCTGAGAACAAGCTTTTCTTTTATGTCAAAAA

AACAATCAGTGCTAGAACACGTGTATCCAACTATTAAGACCACATGCTGTGTCAACCACC

ACTATGATGACATCAAAGTATACAATCAATGAGAATGTCTTTGTAATTGTACTTGCAAAT

AAATAAAGAAAG

>CL6972.Contig3_All

TGGAGATACTGGGTGATGAATCTCACTATTAAAGATGCCTTTGAGGCGGCTTTGGTGAAA

AACCTAGTCATTGTTGCCATGGGTATTGTCATCAATTGCATCAATGGGATCATAATTTTG

ACTTTCTTCAGGAACTCTGTTTTTCACTGTGAAACAAGATACATTCTGTACATGAACCTT

GTTGTGAATGACATGACGATGATTTTTGTTTCAGTGACACTGCACGTTT

>CL717.Contig2_All

CTTTTATTTTTGTTAAGCACATTAAACTGCCTTTGTGAATGAAATGCGCTAAACAAATAA

ACTTGTCTTGCCTTTCCTGAGTAAATAGAATGAAAAAATGAAAGAAATAAGCAAAAATGG

CTATATAATGATTTAAGCACACTGCCCAATATATGCTGAAATGGTAAATGTAATGTTTCT

ACTGTGTTTGACACTAGATGTCCAAATGGCAAACACCTCTAGGCCTCTCACTGTGACAGA

TGTGCAGGCTTTCTGGATGGCCATGACAGACGAGCGGATTTTTAAGATGTTTCTTATCAT

CTTTACCCACATTCTGTTCATCTACATCAACGTCGTGATGATGGTGACACTGCGTAGCAA

AGCCACGTTCTGCAACACGGCCCGCTACATTCTCTTTGGCCACATGCTCCTGATCGACAC

CCTACACCTAAGTGTAAGCCTGGTGCTGTACATGTTGAGTTCTTTCTTCATCACACTGGC

ACGGGCTGCCTGCTCCTTCCTGGTGCTGCTGTCGGCTACGACCTTCACCATTGCGCCACT

CAACCTGGCCGTCATGTCGCTGGAGCGCTACGTGGCCGTCTGCTTCCCCCTGCACCACTG

CCAGCTGGCAACCCCGGCCCGCACGCGCCTCGCCATCCCATTCATCTGGGCTGCCGGCTG

CGTCAACGTGCTGTCCGACGTATGTGTCCTGTTCCTCGCCAAGCCGCCCTTTTACCTGGC

ACACACGGTCTGCACCCACGAACAGCTGATGGTGGCGCCATGGCAGGGACTGAAGGCTCT

GATCCTCAACATCCTCCTCTTTGTCTCCGTGGCCCTTCTGCTGCTCTACACCTACATCGC

CATCCTCATTGAGGCCCGCTCAGCCTCCTCTGACAAGGCGTCCGCACACAAGGCCATGAA

GACTGTGCTCCTGCACGCGATCCAGCTGGGGCTCTCTCTCATGTCCTTCTGGTTTGTCTT

CCTAGAATCGCTGTTCTCCCGGCTGCCCTTTGACATCTACAAAGAGCTGCGCTTTGTAAA

CTACTTTTTGGTGGTGATTCTGCCCCGTTGCCTGAGTTCGCTGGTGTACGGTTTGAGGGA

CGAGGCCTTCAAGCCCCTGTTCAAGCGCCATTTTTTATTCTGTGGCAGCTGGGTGAAAGT

CAGACCTCCATAAGTCTCACTTGCCCGCAAATGTCCACACGTTTTATGTGTGTGTGTGCA

CGTTCGGTTTGTGTGCTGAAGACACACTGTGTGTGTATGTTAAGGTGTCTATGTGTATGT

TTCTGTCAGTTTGTGTGTGTGTGTGTGTGACTGAAACCTTGATGTATGTACCCTGTGAAT

AATACTAATGCTGACTTATTTGTATGATAATTTATTTGTATAGCACCTTTTTTAAATCCA

ACCTGTACACACAATGTTCATATATGTATAACAAATATTACTGTTACATTGGCAAAATTT

GTACTGGAGTTTATACGACTCTCCCCTTCCTCAATATGTCATGTTTGTGGTGTACTTATG

TTTACTTTGGCAACAATATGCAACAATAATGAAATGTTGAAACAGATAACAAATTCTCCA

TTTCTCTCCACCACCACTTATCTTAATCTGTCTGACCCCAATATCATTCCGGACCCAAAC

AAATGCTAGCCTAAAATCTAACTACAGGATTTCACCATGACTAAATGCACATTATACACT

AATCAATCAGTACATGCTGTTCAACATGAGCATCTAAAAACTATCTTAATGATCTTAATA

CCATGCCTGCGTGCACTTCACTAGGTAAATTATGTCCAACATCATTGCCTATCAGTCCCA

CAGGATTACACATAGTTTTGTTCAAGGCGGCGAGACATAAAGGCAAAACGGATTTGTCAT

TAGTTGTTAGTTTAAAGACATATAAAGAAAAACAGTTCGCTTCTTTACTTATGACAATCA

TGATCATAACTACCGGTTCAAGTTCAAGTTTATTGTCATTGTTACGACCATATGCCATAG

GCCAACAAAAACAGGGAGGCAAGAGGTTGAATTTCAAACTAAATATATTTAACTAAATTA

CAGACAAAACAAACACCATGAGATGGGTCAAAGTCAGTTATCAGTACTGCAAGCATGTTG

TTAATAGTGAGATGTCCTGAGTTATGTGTTGCTGTGTCAAAATCACAGAAGCATGGCCCC

AAAATGAGAGAGCAGTCTTTTAAAGGAGAACTTCGGTGTAAA

>CL717.Contig3_All CTTTTATTTTTGTTAAGCACATTAAACTGCCTTTGTGAATGAAATGCGCTAAACAAATAA

ACTTGTCTTGCCTTTCCTGAGTAAATAGAATGAAAAAATGAAAGAAATAAGCAAAAATGG

CTATATAATGATTTAAGCACACTGCCCAATATATGCTGAAATGGTAAATGTAATGTTTCT

ACTGTGTTTGACACTAGATGTCCAAATGGCAAACACCTCTAGGCCTCTCACTGTGACAGA

TGTGCAGGCTTTCTGGATGGCCATGACAGACGAGCGGATTTTTAAGATGTTTCTTATCAT

CTTTACCCACATTCTGTTCATCTACATCAACGTCGTGATGATGGTGACACTGCGTAGCAA

AGCCACGTTCTGCAACACGGCCCGCTACATTCTCTTTGGCCACATGCTCCTGATCGACAC

CCTACACCTAAGTGTAAGCCTGGTGCTGTACATGTTGAGTTCTTTCTTCATCACACTGGC

ACGGGCTGCCTGCTCCTTCCTGGTGCTGCTGTCGGCTACGACCTTCACCATTGCGCCACT

CAACCTGGCCGTCATGTCGCTGGAGCGCTACGTGGCCGTCTGCTTCCCCCTGCACCACTG

CCAGCTGGCAACCCCGGCCCGCACGCGCCTCGCCATCCCATTCATCTGGGCTGCCGGCTG

CGTCAACGTGCTGTCCGACGTATGTGTCCTGTTCCTCGCCAAGCCGCCCTTTTACCTGGC

ACACACGGTCTGCACCCACGAACAGCTGATGGTGGCGCCATGGCAGGGACTGAAGGCTCT

GATCCTCAACATCCTCCTCTTTGTCTCCGTGGCCCTTCTGCTGCTCTACACCTACATCGC

CATCCTCATTGAGGCCCGCTCAGCCTCCTCTGACAAGGCGTCCGCACACAAGGCCATGAA

GACTGTGCTCCTGCACGCGATCCAGCTGGGGCTCTCTCTCATGTCCTTCTGGTTTGTCTT

CCTAGAATCGCTGTTCTCCCGGCTGCCCTTTGACATCTACAAAGAGCTGCGCTTTGTAAA

CTACTTTTTGGTGGTGATTCTGCCCCGTTGCCTGAGTTCGCTGGTGTACGGTTTGAGGGA

CGAGGCCTTCAAGCCCCTGTTCAAGCGCCATTTTTTATTCTGTGGCAGCAGGGTGAAAGT

AGGACCTCCGTAAGTCTCATAGGCCCACAAATGTCCACATGTTTTTTGTGTGTGTGTGTA

TGTTTTGGTCAGTTGTGTGTGTGTGTGTAACTGAAACTTGTGAATAATACTAACACTGAT

TTATTTGTATGATAATTTATTTGTATAGCACCTTTCTGAAACCCAACCTGTATACACAAT

GTTCATATACATATAACAAATATTAAAACTGGGCCTGGGCCTGAACTCCCAAAAAAAA

>CL7175.Contig2_All CGCAGATGCTGTACATTGTTTCTATATTTTTATTGTAAATATTTTAAAATGTTTCTGTTT

TAACGTTGTCAGATTTTTCTGTCTTTACCTGTAGCTAGTAGTAGTAGTTAGTGATAAAGT

GGAAGAAGGGAGAATAACTTTCTTACTGGTTTCAGACATGCTGGATCTCAGAGCAGCTCT

GTCAAATGGCACCCTCATCCACCCTCCAGGCTTCTACATTGTTGGACTAAGCAGCATGGC

CCATGCTAACATCTACCTTATTTTCCTAGGTGTGGTTTATGTCCTCACCATTGTGTTTAA

CTGCTTTCTCCTGTCTCTTATCTGGC

>CL7212.Contig3_All

GGGCGTTTTGCCCTCCCACCAGTGTGTGTCCTTGACTTCAACATCAACTTGTATCAAATT

CCCTACATATATACATGCATGTACACTTGCTCTTGCTTCAGAAAACTGTACTGTCTGAAA

ATGAGATTTTATCATCTTAGAATGCGGAGCAAAAAAACGGTGTACAATTGTATGCGTATT

GTACTGTATTTTACACGACTTATCTATTTATCATATGTAATCTATTATATGTATTGTGTA

AAAGCTTAGACATGCAGAACTCAACTCAGATTTCATTTGTGATCTTAACTGCATACACCG

ATGTTGGACATGTTAAATACTTTTACTTCACTATTTTGTTAGCATTGTATGTTTGCATAA

TTTTTGCCAATGTACTTTTAATTACCATTATTTATATGGATAGGGCTCTGCATGAGCCTA

TGTACCTGTTTCTGTGCAGTCTGTGTGTGAATGAGTTATATGGCAGCATAGGTCTGTTCC

CTTGCCTTCTGACTAACCTGATCTCAAATAACCATGAAATTCATCTCACATATTGCTACC

TACAAATTTACTGTTTATATACATATGGAACAGTAGAAATCTGTAATTTAACAATCATGT

CTTATGACAGGTACTTGTCTATTTGTTATCCCTTACAGTACAACAACATCATGACGCTGA

ACAGAGTCTGTATTCTAATTGTGTTTGCATGGCTGTTTTCATTTGGCCAATTTACAGTTA

GCCTGGTGCTGAGTGTTCATTTGCGGTTGTGTGGTAACATTATGGAGAAGGTATGGTGTG

ACAACTTTTTCCTGATCAGACAATCCTGTACAGATACAACTGTAGTTAACATATATGGAC

TTTGCATGACTGTTGTTGTAATTGCTGTTCCACTTGTGCTTATTTTGCACTCATACATCA

GGATATTCAAAATTACTTTTAGATCCAGTAATGGCCCAAACAAGAAGTCTTTAAATACAT

GTGTTCCTCACATTGTTTCACTGTTAAATTTTTCCATAGGTGTTTCTTTTGAGATATTTC

AGGGCAGAGTATTGAAGTTAAATATGCCACCCACATTGCGCATAATTATTTCAGTGTATT

TCTTAATGATTCCACCACTTGTCAATCCAATTATGTTTGGACTAAAGTTAACTAAAATAC

GAGATGCATCTAAAAAATTCTTTCAACAGGTAATTCTTTTGAAATTCAGAGCATGTACTA

TTACAGATAACATGTATGCAAAATAGCAATATCACATCCTTTTGATTAATTTCAAAGTAC

AAATTCTATTTTATTTACATTCATTAAACATATAAGAAAATAATTGATTGAGATTCCACA

GACTAGTCATTGTTTTACTGCTCACATGCATAATTATTGTGGTAGGGAGTCATTATAAAA

TATGAGACTGTTGTTCTGAGAAAGCTGTGCTGTGTTTGGAAATCTCTTAAATACTGTTTA

ATATTTTCTGAATAAAAGGAAGTAGGTTTTAGGCTGCCTTTTATTCCCACTTCTGTAAGT

ATGGCAAAGGGCAAAAAATATTTTATTGTTTTCATGTGTTCATGTGACTAGTTCATGTTC

ATCATGTTGCATTACATTACACTAGGAAATAAAAATGGCATATTGTAAGGAAACATACAT

CTGCACATGGGTCTGCTCTAGAAAGGTTTACTTATGGTGTTTTATGTCTCTACACTAAAT

AATGAAGGCAGACTACCAAAACATTTGACAATAATTACACTTTCTACACTTTTATTATTA

TCCTGTGTTAACTTTATATGCCTGTGGAACATGCTGGCATACTGTGCATATGCCCTTAAA

GACCCCTTTCAGTAAATATCAACTGACACCCGTATGGTTCATTTTTAATATCTTTTTTTA

CGTATTCACTGTACATTATTACCGAAAATACCCTTTCTAAACGTATGGTCTTATTCTAGT

CTTATTATGTTAATCTACTTCTTTCACCTTTTGCACCATTTGAAACAAAGCCCCTCTTAA

AAGGAAAAACACAGCACCACCATGTTTTATGTCTTGTGAAATGGATGGTAACAGTTGATG

TAATACCTTTCTGG

>CL7212.Contig4_All

CCAGTAATGGCCCAAACAAGAAGTGTTTAAATACATGTGTTCCTCACATCGTTTCACTAT

TACATTTTTCCATGGGTTGTTCTTTTGACATATTTCAAAGCAGAGCGCCCAAGTTAAATA

TGCCTCCCACATTGCGCATAATTGTTTCAGTGTATTTTTTACTTATTCCACCACTTGTAA

ATCCAATTATGTTCGGACTAAAG

>CL8025.Contig1_All

CATGCTTCTAAGCATCACATTCTTCTATAACATGCATTTCTGAACATGTATGAGTGAATT

AATCAATGCATTTGGCTTTCTATTAATGCTGTAGAATGAAATCTGTGTAAAACACTTAAC

ATTGTTTTAGACTGCAGTCATGTTGAATGCAAGTATTCATTTGGAAGTAAGTCTGGATGC

ATTTTTTATTCCGGATGGGGCGAGGTATCCCATATGTTTATTTGGGCTTGTGATTTATCT

GTTTTGCATTTCCTGCAACATGACTCTGCTTGGACTTATCATTGCAAAGAGGAGCTTACA

CAAGCCCATGTATTTTATTTTGTTCAGTCTACCATTCAATGACCTTGTAGGGATTACTGT

CATGCTTCCCAAAGTCCTTTCAGATATCTTCACAGATACAAGCAGCACCTATTATCCCCT

GTGTGTTCTTCAGGGCTTTCTGCTTCACATGTACGGTGGTGGGATTCTTTTCATCCTGGC

TGCCATGGCCTTTGATCGATATGTTGCCATCTGTTTCCCATTAAGGTACAATTCCGTCAT

GACACCTGGAGTTGTAGTGGCAATCATTGCAATTATTTGGGGTCTTGACTTGGCTTTCAT

ATTGTCACTATTTTCTCTTCAGGCAGGCCTACCACTATGCAAAACCAAACTCATGAATGT

GTTCTGTGATAACCCCTCTCTTCTCAAGATGACATGTGGCAATACCTTTATCAACAACAT

TGTTGGCTTGGTTTACTTGGCACTTATGCAATTCATAAGTATTTCTATTCAAGTGTTCTC

TTATGTCAAGATACTGATTGCTTGTCTGGTTACAAGACAAAATGAAGCCAGGAGTAAGGC

TATTAACACATGTGTGGCACAACTGGTTATATTTTTCATATTTGAAATCATAGCGACTTT

CACCATTCTTTCACACAGATTTCAAAATGTTCACCCAGATTTACAGAAGATTATTGGTAT

GCTGATTTTTCTTGTGCCTCCTATCCTGAATCCTATTGTGTATGGACTGAACACAAATGA

AATTAGAAGGAACCTCTTTGGTTATCACCCAAAGTTGTCTGCATAAAACTACCCGTGTGT

AAACCAGTTACAGCTAAAATAAATCTAGAAATTTGACAATACATTCATGTCTACAATAGC

TGGGCTAACAATGTGCATTATCTCCCTTAACAATGGAA

>CL8025.Contig2_All

TTGACTTTTTTTTTATATTCACAATATTTATACTGCAGGCCAGGCTACCCAGATGCAAAT

CTAAACTTATGAATGTGTTCTGTGATAACCCCTCTCTCCTCAAGATGACATGTGGCAATA

CCTTTATCAACAACATTGTTGGCTTGGTTTACTTGGCACTTATGCAATTCATAAGTATTT

CTATTCAAGTGTTCTCTTATGTCAAGATACTGATTGCTTGTCTGGTTACAAGACAAAATG

AAGCCAGGAGTAAGGCTATTAACACATGTGTGGCACAACTGGTTATATTTTTCATATTTG

AAATCATAGCGACTTTCACCATTCTTTCACACAGATTTCAAAATGTTCACCCAGATTTAC

AGAAGATTATTGGTATGCTGATTTTTCTTGTGCCTCCTATCCTGAATCCTATTGTGTATG

GACTGAACACAAATGAAATTAGAAGGAACCTCTTTGGTTATCACCCAAAGTTGTCTGCAT

AAAACTACCCGTGTGTAAACCAGTTACAGCA

>CL8025.Contig3_All

CATGCTTCTAAGCATCACATTCTTCTATAACATGCATTTCTGAACATGTATGAGTGAATT

AATCAATGCATTTGGCTTTCTATTAATGCTGTAGAATGAAATCTGTGTAAAACACTTAAC

ATTGTTTTAGACTGCAGTCATGTTGAATGCAAGTATTCATTTGGAAGTAAGTCTGGATGC

ATTTTTTATTCCGGATGGGGCGAGGTATCCCATATGTTTATTTGGGCTTGTGATTTATCT

GTTTTGCATTTCCTGCAACATGACTCTGCTTGGACTTATCATTGCAAAGAGGAGCTTACA

CAAGCCCATGTATTTTATTTTGTTCAGTCTACCATTCAATGACCTTGTAGGGATTACTGT

CATGCTTCCCAAAGTCCTTTCAGATATCTTCACAGATACAAGCAGCACCTATTATCCCCT

GTGTGTTCTTCAGGGCTTTCTGCTTCACATGTACGGTGGTGGGATTCTTTTCATCCTGGC

TGCCATGGCCTTTGATCGATATGTTGCCATCTGCTCACCATTAAGGTACAATACCCTCAT

GACACCCAAAGCTGTAGCAGCAATTATTGCACTCGTTTGGGGTCTCGACTTTTTTTTTAT

ATTCACAATATTTATACTGCAGGCCAGGCTACCCAGATGCAAATCTAAACTTATGAATGT

GTTCTGTGATAACCCCTCTCTTCTCAAGATGACATGTGGCAATACCCTTATCAACAACAT

TGTTGGCTTAACTAACACTGCTCTTATGCAAATAATAAGTATATCTATTCAAGTGGTCTC

CTATGTCAAGATACTGATTGCATGTCTAGTAACAAGGCAAAATGAAACAAGAAGTAAAGC

AATTAACACCTGTGTGGCACAACTGGTTATATTCTTTTCATTTGAAATCACAAGTACTTT

TACCATTTTATCACATAGGTTTCAGCATGTTTCACCTGACTTACAGAAGATTATGGGCAT

GTTGATTTTTCTTGTTCCTCCTATCCTTAATCCGATTGTGTATGGATTGAACTCTCATGA

AATTCGAAGGAACCTCTTTGGCCATCATAATGAAGTGTCTACATAAAATGTCTTATGTAC

ATCACCGGCTCCCATGCAGTAGGCCTAAATGTTGTACACTGAGAAATGTGTATTTGATGA

TGAGAACTCCTACCGTGCTCAGACCCTGTTAAGGCCAAATGCCTCCTTTTAACCATGTTG

ATAAAAATGTAAATTTAAGATTTAAATTCTCCCAAACATTTCATGTTTTTTTTAAAGAAA

GCTTTTAGACCCCTGTGCCAGGTGTCATGTTTTAACAGCATGGCAGGTTTTGTGAAATTC

TGCAGAGAAAAGAGTCAAAAAAAGAGAAAGGAAACATAACAGCGTTGACAGAGGTGGTGT

GGTAATATGCATCCACTTACAGTAGGCTTATGTGCGGAAGAATTTTCTGTTTTGTTTTGT

GTGATATTAAAGTGCCATTGAAAGGTACTACACAGACAAAGATTTGTATTGTAGCTTAAG

TCATGTTTTATATACCAATTAGCAGTTACTGTACCAATCTGAATGTTTTTTCTTGTGGTT

CGGAAATGATTGTGAGCTAGTAGTTGTTTGACATTACATTGACATTTTACAACACATTTT

CTGAATTGTTTACCAGTTTGTTTACTTACTGTAATTTGTATTTGTTTACTGAGCATATTA

TACACTTGTAAAGAGTGCATTTGTCAAAGAGTGCACTTTTGTGCCTGTTCCCTTGGACTA

AGAGTTTTTGCCCTTTGGATTGTTTGCCTGTATGACTGAACATTGTCTCCTAATTACAAA

GTCTTATGTCCCTGGATTTGTTGCCTGAAACCTTTTGAGTAAAGTTTTCACTGTTTTGAA

AAAA

>CL9458.Contig1_All

ATGCTTTAAGGTATATGTATGAATGTTGGAGGGAAACAACAGAACAGAATTCAGAATAAA

AGAGTTTATTCTGGTGGGCTTTCCTGGACTGCATTCTGATTATCATGGCCTAGTGGCATT

CATACTCTGTCTTGTGTATGTTACCACAGTTGTAGGGAACTCTGTTCTAGTGGTGACGTT

TGTGCATGTGCCCAGCCTCCATAAACCTATGTACATTATCATGTTGAGTTTGGCTCTGTC

AGACATAGGATTCAGCACAGTTGCTCTGCCTAAGATCATATCTCGATACTGGTTTGATGA

CAAATTTATTGCTTTCGATGTATGTTTCTCTCAAATGTTCCTCATTCATTATTTTGGGAC

TGTGAACTCTTACATCATGGGGATCATGGCCATGGATCGTTACATAGCCATCTGTTTTCC

GTTTAGATATCCTGTGGTAATGAAAAATCGCACAATGTGCATCCTCAATATTTTCTTTTG

GCTTTTCTCTTTTGTTACCCCAGCTATCTTTTCTGTCTTTGACTACAGGCTTCCATATTG

TGGGCCCAATCAAATCTTTCAGTGTTATTGTGATCATTACTCCCTTGTTACACAAGCGTG

TGCTAGCCATGGTTTTGCAACACTGATTGCCTTTACAGTAGCCATGCTGGTGCTGCTGAT

ACCCCTGGCTTTCATCATCTACTCCTACATCCACATAATTGTCAGCGTAGCACGAATAGC

CAGCTCACAGAACCGGTGGAAAACGTTCTCCACCTGTACCACGCAACTGTGCATTATCAT

TCTTTACTATCTGCCACGGTGTATTGTGTACATTTTCAATGTTACTGGTCTCTATATGGA

CATAGATTTGCGCATCACCCTTATCCTTTTTTATAGTCTGTTTCCTCCCCTCGTCAACCC

CTTTATATACTGCTTTAGAACAAAAGAAATTAAACAGGCATTAGGTCATTGGTTCAGTGT

AGTGAAGCTGAACAAGACATCCTCTATAGCAGCTAATTCAAGATGACAGGCTATGTAGTT

TGTTAGTGTCAGTGTTAGTTGTATGTTATGATATGAATGTAAGATATTGTGGGGCATATT

AATTAAATGGAAAACTAGTTGTGACGTTGTAGCATTAAAATTATTTCCTGATCTTCAGAA

TTATAGTTTTTGATATTTTGATCCTTCAATACAAACTGAAGTTTTTGTGGGGGAAAAAGG

TGTATTTATTTAATAGTGTGTATCTCACTGCCCTAATAAAGCCTTCGGTAGTCTTGTTAA

TGTAGTTGGCAACACGATGATAGTTTTTATCAGAATTTTACAGTGAAATGTAAACGTAGC

TACACTTTAACAGGGTGACTACCATTTTTCCAGTAATGGTTTCTGTTTTAGGCTACTATA

TGATTTTGTAGTATGATATTTTCTCATTTTTTACAGTAATTCTCATTCGCTTTGGTACAT

TTCTCAGATCAGAATTGAAATTCTCAAAACTACTTGTTCAATCTTCACATCATTGTGTCA

CGTGCATCAAAAAGTTTCTCATTTTTTAACAAGTTGCAAATGCTTTGGTACATCCATGCA

AATGATTATGTACAATTTTCTGCCGTTTGCTACATTATCAATTACTTATGTCATGTTGAT

CAAAATGTATTATAGTGGGTCTCTGTTGAATAGTGTCACCCCCACAACATTAAGGGATTA

GTTCATGGCATAAGTCTTTACATGCAAAATGGTTGAACAAGTCATAATCTGTCAAGCATA

TTTTTATACATTTCCATTTCCACTTTTTTTCTAAATCTCTTCTAAATTGGTAAATTGCTC

CCAGGTGAATGTTGG

>CL9458.Contig2_All

GATAAATTCACCCCCTTTAATGTATGCATGACACAGGTGTTCCTGGTGCATTTCTTTGGG

ACTGTGAACTCTTACATCATGGGGATCATGGCCATGGATCGTTACATAGCCATCTGTTTC

CCTTTTAGATACCCAGTGGTTATGAGTTATCGCACCATGAGCAACCTTAATATTGTTGCT

TGGGCATTCTCCCTTACCACACCCGGCATCATGATAGTCTTGGATGCTATGTTGCCATAC

TGTGGGCCAAACCGTATCAACCAGTGTTACTGTGATCATATTTCTGTCATCTCACAGTCA

TGTGGTGACACCAGTATTGCAAAGCTGGTTGCTTTTACACAAGCCATGCTGGTGCTGCTG

ATACCCCTGGCTTTCATCATCTACTCCTACATCCACATCATTGTCAGCGTAGCACGAATA

GCCAGCTCACAGAACCGGTGGAAAACGTTCTCCACCTGTACCACGCAACTGTGCATTATC

ATTCTTTACTATCTGCCACGGTGTATTGTGTACATTTTCAATGTTACTGGTCTCTATATG

GACATAGATTTGCGCATCACCCTTATCCTTTTTTATAGTCTGTTTCCTCCCCTCGTCAAC

CCCTTTATATACTGCTTTAGAACAAAAGAAATTAAACAGGCATTAGGCCATTGGTTCAGT

GTAGTGAAGCTGAACAAGACATCCTCTATAGCAGCTAATTCAAGATGACAGGCTATGTAG

TTTGTTAGTGTCTTGTATGTTATGATATGAATGTAAGATATTGTGGGGCA

>CL9638.Contig1_All

AACCAGTGTTACTGTGATCATATTTCTGTCATCTCACAGTCATGTGGTGACACCAGTATT

GCAAAGCTGGTTGCTTTTACACAAGCCATGCTGGTGCTGCTGATACCCCTGGCTTTCATC

ATCTACTCTTACATACACATAATCGTCAGTGTTGTGAGAATAGCCAGTGCACAGGGCCGA

TGGAAAACCTTTTCTACCTGCATCACACAAATGTGCATAATTAGTCTTTACTATCTACCA

CGCTGTACTGTGTATATTTTCGATTTGACTGGTGTCTACCTGAATGTAGACCTCCGCATT

ACACTTGTACTCTTCTACAGTCTGTTTCCTCCTCTTGTGAACCCCTTTATCTATTGTCTC

AGAACACAAGAGATAAAACAAACGCTAGCTCGCTGGTTTAGTGTGAAGAGTCTTATAAGG

AAATCCACTGTTGTTGCTACTCTGTGACAGTCTGAAGCAAATAGATTATAGACTTAAGAA

GCTCCTTAAAGGTCTCATTGCATAATGTTTTCATTAATTGGTGGTCTCTAGTATTAATGA

AT

>CL9638.Contig2_All

CAACAGTTTGGCGAAGTTGGCTGCCTACATAATGTCCATGGTGGTGCTGCTGGTACCCCT

TGTCTTCATCGTATACTCCTATGTCCACATAATCGTCAGTGTTGTGAGAATAGCCAGTGC

ACAGGGCCGAAAGAAAACCTTCTCTACCTGCAGCACACAAATGTGCATAATAAGTCTGTA

CTATCTACCACGCTGCACTGTCTATGTTTTTGATGGGAGTGGTTTCTACATGAATTTAGA

CATCCGCATTACACTTGTCCTTTTCTACAGTCTGTTTCCCCCTCTTGCTAACCCCTTTAT

CTACTGCCTCAGAAC

>Unigene11975_All

TGCTGCACTTGCCCTGCGCAACACCTTGACCATTGTTGTAGCAGTGTGTTTGTTCATCCC

ACACACATTTTGCCACACAAACACCATCTACCACTGCCTCTGTGAACACACCTCGGTCGT

TAAACTGGTGTGTGGAAACATAGCACGCAATTTAGTAGCTGGAGCCATGACGTTCTCCCT

GACCACCAGTGACTGTATTCTTGTTCTTGCCACTTACATCATCATTTTCATAGTCATATT

CCAGAGCCCTTCAGGAGAGTCCCGACAGAAAGCCATCCACACCTGCAGTACACATTTAAC

AGTGATTTGCGTTGGATACGTCAGTGTTGTGTGTGCGTTTGTTGGGTACAGAGTCAGTGG

TATCCCCCCTGATACAAGGGTTCTGTTGACCTTATCATATCTGATAATCCCATGTGCTTT

TAATCCAGTCATTTATGGGATTAGGACAAAAGAGATTAAAGTGCATGTGCTTAAACTGTT

AAAGTTAAGTAAGGTGGATTCTGAGAAAACCTGATGTTACCTGTTCAGTCTAAGGCCATA

CACTGATTGATGGCCTCTGATAAGACTGTTGAAATTGACCCTGGCTATTTAGGAAACAGT

AATTGTATCTTGGTATGGCAATTTAACCTGCTCAACTTAGTGCATTATCATAACCATCGT

ATAAAAGAAAATTTTAATTCCTGACATATTTGATGGAAGAAATATTTGTTGGTGTGACAC

ATTTGAAACCAAACTGAAGGGGAAAAGTACTTTTTGTTATTTGATAAATTTAAGCATGTT

CACATTTACAATGGCCTTGTAAATGCTCCTTGAACAAAATGTTGAACAAGAAGATTGAAT

ATTAAGTTATTTTGTCCTTGAGTTTTTTGTTGTTGTTTTTGACGCCTTATCCCCTTCAGT

GGCAAGTGCTGTTACATTTGATGAATACATGTGACATACAGTATCATCATATATGATTTG

CCTGTTTGCCTGTTAAACTGATTTCTAAGTTTCTCTAAACATGCCATTTCAATATGCATA

TTATGTTTTCTTAACTGGGATGCTGGAAAGATAGTTTCTTTCTGTTCTAGCACATTCTTG

TAATGGTTCTGGTATTGTTGTACAGATGTATAAAGTCTTTATTGCTTTCAATCCCCTCAG

TTAGTGAACTACTTGTTTTTTGGCCATACATTTTTATAATAAACATCTTTGATAAATAAA

ACACCTAATGTTGATGGATTTCATACCCCTATTTCCAGCATTCAGTCTCAGTGATATAGG

CCTACTACCTCTTGCATCATCTGTGTGGTCAGCACACTTGTAATTGCCATTCTAATTGCA

ACTGTTGATGATGATAATTGGCCAGACTAATGTAGCCGTCCCTCAGTGCTAATTAAAAGG

GAGGGTAATTGGTCCCGGTGCAGTGGTGGTCATGCCTCAGTAGGACTCTCTGATCCTGCT

CCCTCCCAGGACTGTGACCTCACATGTTTGTCATGGACATGAACTCTTTGATGATTAAAT

TGTTTTTGCTGGTTGTGCTGGGCTTCCAACATCCACAAGTTGCCACATCAAAAGTCGCCA

GTCCAAAAAAAAAAATCCTGGAAAATGTTTGAAAAAGATGTAGAGTAAACCTGTGTCAG

>Unigene14513_All

CCCGGGTACAGATCATGGTGTTCTATGCATCTCAGACTATTTAATGTGAAAGTAGTACTT

ATGTGTCTGGCATCCTGGTTGTTTTTTCTCTTTCCTGTTTTTAACCTGGGCATATGTATT

TCATCCAAGTTCATACATGATGTGAAATACGATATGAGATTTTTATTTTCACTCCATTGA

GTCAAAGCTTTGTCACATGAGTAGGCATGGGTAGACTTCTGAAGTAATGATGCTTAATGG

TGCTTTTCAGCATCTAATATGTAAAGTATATGATTGTGAAGTGTTATATGATCTGTCAAA

AAGCATTGTTGATATTTTTTGTGTATCAATAATCTGATGATTTTTAAATTAGTTTTACAC

TTTCTGTCTTTTTAAACATGGTGTTATGCATTTAATAGATCTTGGGTAATTGTATAATTC

ATTAGTCATTGATAATAATGATATATCGTGTTTCTTTCAACTGACTTCATGATTTTGTGA

AGTTTTTGAGGAGGTCCATAGACGTAAATTAATTACAAATTTTGGGGATTAACATTTGGT

AATCTGTCAGAGACTGTCAGCAGTTCTCTAAAGCCATGCCGAACGTAACAGGATTCCCTG

CCCGCTTCCCCGCCGTCTTCTCTTCTCTTGAAGACTGGCTCATAGTGAAGGCCTTTTTTT

GTACGGCTCCCTGTGCCTTCTTCCTGTATGTGAATTTAGTCATGTTGTACACACTAAGGT

CTAAAGAAATCTTCACTGAAACCCCTCGCTACATACTGTTCAGTCACCTGCTCGTGTCTG

ACTCCCTGCAGCTGTGGTGCACCCTTGTGTACTATATACTGTACAACCAGACATTTGATC

AGGAGACTTTAAAAATAATAGGTGTTTACTGCTTTTTGAATTACCTTGTCTGTCGAATCA

CAAATAACTATCTTACCCCACTTTATCTAGGCCTTATGTCCCTGGAGCGTTATATTGCTA

TATGTTTTCCCCTTCGACATGCTGAGATAGCCAACAAAAGCAGGACCATGTTGGCCATAT

TATCTGTTTGGTTACTGGGCTTAATTCTCTGGACAGCTGACCTAGCTGCTGCCATGGTAT

TGCGGGGTAAGGAGGGGGGCTGTACTGACTACATAATCGCCCAAATGGTAGTTACCTATC

AGGTAAACACAGCATTAACAGGCCTTGTGTTTTCTTTGGTGAGTGTTGTTATAGTCTGTA

TATACATAGCCATTATGATAACAGCCAAGTCAAGTTCTACTGCTGACAAGTCCAAGGCGA

GTAAGGCCCACAAAACAGTACTCCTACACATGGTGCAGCTTTGCCTGTGTCTTTTGTCTC

TTCTATTCGGTGTGGTTCGCAGGGAGCTGGGTTTAAGCGGGCTGGACCCAGTGCTTGTTA

ATGAAGTGACGATTTTTTTGTTCTTAATGTTGAATATCTTGCCCAGGTGCCTCAGTCCTC

TTGTATATGGTCTGAGAGACAAGACATTCAAGGCCTATTTTAAAATCCATTTTCTTTTCT

GTATCAAAAGTAAAATACAGCCATCAGACCTTGAGATTTAGTATGGAACCTAGAGAGATT

TTCTGATATGGCCATGACTTTTCTGTAGGCTGCTCTGGCCCAAGTTCAATTACATTTAAT

TAAGTTGTATTAAGTCCATTACAAATGACAGCTTTTGTGTTTCATGTTCATGTTCACCTG

TTTGTTTAAACTATGTGTGTATTCTAAGAACCCATTGCTGTATTCTGTAAGCTCTTTCAG

TTTATTTTTTGCCTCTGCTAGTTATGAGAATAAATATGTGTATGTATGTGTATATGTGTA

ATTTGAACTTTTACTATGCATTGCCTGGCTCCATTGATCCACATTGAATTGGTTGTCAAA

TGAGCTGAGTTGTAAGCAACTGCACTTCTGGACTACCATGACCTGGATGAATGAGAATCT

ACACAGACATCTTGGAAGGGGGATGTCCATCTTTCACTAGCCTCCCAAAGTGCTGCTGAA

ACCATGCCCACACCACTGTGCTATCTATCCCACAGCAACAGCTAAAGAACAGCCTCAGGT

ATTCCTTTTATCTACTGTGAACACAAAAAAAGAAGCACAACACTCACTCCTGCTAAAACA

CCAAATCTAATGACATTCTTCTAAAAGCACTGGTGGCTATCTAACCCAGCGATGAAGCAT

GCATACTTTTGCTAATGAAAAATGCAACACACTAAAAGGCAGCGGGGAGGGTGGATTTTT

TTTTTTTGGAGTGGTTTTGACATATTATATATTATTTCACCTCTGTACAAATAGCTCATG

AAACTGGAATGTTATATTTTATTTTAATAGTATATAATTATTTTGGAAGTAGTCCTAAAT

ATTTAAAGATATTTTTGGAAGAAGTATTGTTTTCTGTGTTGTTCTTGGGCTCGTTAAAAC

ATGTATTTTCGAAGAGCAACTGTTACACTAGAACCACAGGGGGCCTTGAATATTTTTTAA

AAAGTGGTCTAGAAACACATCTCTCTGAGCTAGCGAGACCTTAGAGAGAATCCACATAAC

ATCCGCACAAAAGATGTCTCACCCACGGCAATTAGAGGAAGTCACCCTCTATCTATACAA

CCACTAGGCAAATCTTTTCACAATCTTGTGTCTTATTCCACACACCACACCTTAGTAGGA

CCTCTTCGTGACACAGCTCTGTGGGAGTAGTTGTGCCATCGCCGTGTTCTGTACTTCTCA

TTATTTATTTTACTTACTCATATCACATGTTACTCCACTTGGACATATCTCTGTAATATT

TTAAAGGAAGTGTGACTTTTGAGGTGGAGCCATTTTGTTGTCAGTGTTAGGCTATTCATC

ATTATCACACGAGTTACGTGTCATCTGATTATTTCATGTGTTCAGAGGTGTCAACCTACA

AATTAAAAAACCTGCAATATATGCCTTCTTGCCTGTGCACTTAACAAAGGTGACTTTACT

AATTAGCTGATCTACATGTCTGATGATTTGTGGGCTCGTTAGGATCAGCTGGGTTTGATC

AATAGAGAAATGGTTTGATCAATAATGTGGCAGGGTTTTTACTTTTTGAAGCCAAGATCT

CCACTTCTGCATGTTTTAATAAACACATCCATACCAGGAAGCCAGTGATCAGTCAAACTA

AAGGTAATGTCTTTTCTTCCCACCCTGATGCAAAGCCTCATGCTGTTTACACTACTGTAT

ATGTGAGCATTAGCAAAGTTAGCGACCTACTGCTACTGCTAACCAGATTGGTTGTAGTAA

AGGCATTCTGCCAGTATGTTTGTGTCATGCTAAAATATAAGGACAGCTTGCAGGGCAGGC

TACAAGTGAGCTCATAACCTTTCCAACAGTCTGGTCACTATGGTATATACTGCTCATGTT

AAGTTAAAAGGCTTGTGCAAATCTGTAGTCTTTGCTCTGTATTAAATTCTGAAGTTATTA

AACAGGTAAAGGAATGTAGACAGTCTTGCCAGTCTAAAGTTAGGTTTAACTGTAGAGGTG

CTAGCTTGCTGAACACTAACTTAATGGATACGTGCCAGTTTTGCAGTCAGAATTCAATTC

TACACCGTTTTCCAAACAACATGTACTGCCAAAATGATGTTTGTATTTTTCTAGTTTGAC

TAGTTTGTTTGCGCTTTGATGAAGTAAAATATATGCACTGATATTTTTATCAAATATGGA

TTTTTGTATTCAAATGTATTTTATTATTAAC

>Unigene15712_All

TGCTGAGTGAGGACAATGAACTCCACAACATCACAGTTTCTCGTGAGGGACACATTCTCG

GCGGCCTTTGTGAAAAACTTCATAGTGGTTCTCCTGCTGCTCGTGCTGAACTACATCAAT

GGCAGCCTGGTGGCCACCTTCCTGAGGAACCAGGTGTTCTACGAGGACCCGCGCTACATC

CTGTTCATCCACATGGTCATCAACGACGCCATCCAGCTGACGGTCACCATCACCCTGTTC

GTGCTCAGCTACATCTTCTACGCCATTAACGTCTCCTTTTGCTGCTTTTTCATCCTGGTG

GCGGTCTTCACCACCCGCAACACGCCCGTCAACCTGGCCGGCATGGCCATCGAGCGCTAC

ATCGCCATCTGCGACCCCCTACGCCATGCGCAGATCTGCACCATCCGCCGCACCTACATC

CTCATTGGCCTCATCTGGTTCGTGACCGTTGTGCCCGACATCACCGACCTCTTCGTCACA

CTCGCCCGCGAGCCAGTCAGCTTCTTCCACACGGCCGTCTTCTGCATCCGGTCCAACATC

TTCAAGGACCCCGTGCTGCTCTACAAGAGACAGGCCTTCGACGGGCTCTACTTCTCCCTG

GTCTTCTTCACGCTCGTCTTCACCTACCTGAGCATCCTGTGTGCGGCACGGGCCATGTCC

ACCGACATCAGGTCGGCCCAGCGCGCCCGCAACACCATCCTTCTCCACGGCGTGCAGCTG

CTCATGTGCATGCTCTCCTACATCTCGCCCAGCATGGACCTGATCATGATCCGGATCTTT

CCGAGTCAGATCCTGGAGATCCGCTATGCCAACTACCTGATCGTCTACATCTTCCCACGC

TTCCTCAGCCCCATCATATACGGGGTCAGGGACAAAAAGTTCCGCAGGTATCTGAAAAGG

TACTTTGTGTGCAAGCAGTGCAGTTCGGAAGTTCAGCACGAAGAGGGCGAGGACAAAATG

GTGTAGGAGCTTTCATTTTGTGTCCTGGGTGGGTTTTGCTGTTCAGATGTGCAGTTATTA

TTATCATTTGGTGCAAGAAACATGATATGTATCTTTTGTATGTAAATGGTGAAGACCTAT

GTCTGTCTATAATTGACTTTGTGTGTGTGTCTGTGTACATACCATCATTAAAAAATCCAG

GGAGATAAGTATATGAAGTAATTCTGCATTAAAAGAATATGAATGAAAATAACATAACAC

ATACAAAATGAAATAATGTGAGAAATGTAACTAAAGTCAAGATAAGGTTAATGTAATATA

AACCTATGATGTCATAAAGTATATTAGGATTAGGATGAGTATATTTTTTATTGTCTCTTG

ATATATTATTCTCTTTCCCTGTCTTTATTATGT

>Unigene16199_All CTTGTTTGTCTGTGTCAGGCCTGAGAGGTCTTGCTCACACGGGTGCATCATCCTTACTAT

TAACAGGATGAAGGCACACAAAAGTGGGATCTGGGTAAGGATGCATCTACTGGAAACAAT

GGAAACTGATTTGTGCAGTCAAGATGAAAAACCTGTTGGTGCATTCAAAAGAAGCTATTC

CTAATTTAGTAAATTATAAATGAACAGTATGTGATTATTCATAAATTAATTGTAGTACAA

TGTCTTTAACTTACAATGTTACAATATTACATGTCTTTTCATGTCCTCTTTCCCATGTGC

ATTTTGAGATGAGTAAGTGTACTTTATTTCTCCTGTAATATTTGGTTTACTGTGTAGCAC

ATAAGATATTATAGGCAAAATTACAGGGTATGGATATCTGTAATTCACATGCGACCATGT

TATTGTATTTTGTTTTTTATATTTGCTTCTGTTCACAGATCATTGCCAAAGATGAATGAC

TCTTCTTCCATTAGAGACGTTCTGATTTTCACTGTCTATAAAGAGTTGGGCTCAAACAAG

AGTGTGTTATTTACCACTGTTTTAATAACATATGTGTTATCTCTAGTAATTAATGTTTCC

CTACTGTTGTTATTCTACCTGGACCATTCGCTCCATAAGCCGATGTATATATTTTTATTT

TGCTTACTCTTGAATGGTTTGATTGGAAGCACAGCTGTTTGGCCACGAGTGATGTTCCTC

CTGTGGACAGACATCCATAGCACATCTTACGAGCTCTGTCTTGTTCAGGTTTTCCTGATG

GGAACCTATGGAGGATGTAATTTTACTATTTTAACAGTAATGGCTTATGATAGGTTTGTG

TCCATATTTAGACCCCTGCAGTACCACACTGTAATGACCCCACTGAGAGTAAAACTGCTG

TTGGTTGTAGCAAACCTTCCTGCAGCATTGGTACTCGGTCAGGTTATCCTGACCATTCAA

ATCCCCTTATGTAAATACAATCTACATCGGTTATTTTGTGATAATTTGTCTGTTTCTAAC

CTATCATGTGGTGAAACATTTCAAGCTAGGCTTAGTAATTTATATGGGTTATGTGCTATT

ATTATTTTTGTAGTATTTCCTATATGTTTAATTTTTCTGTCATATGTGAAAATTATAAAG

TTAAGCTTAAAAGCTTCAAGACATGCAAGAAGGAAAGCTATTGAGACCTGCTCTCCACAC

ATAATGGTTTTTATGAATTTTTCATTGGCCTCACTGTTTTCTGTTATTTATAATCGCAGA

AACCCTTATTTACCTGGAGAGGCCAACATATTGCTGGCCATAATTTACATTCTTCTTCCT

CCACTGTTACACCCAATTATATATGGCATAAAAAGTCAGGAAATTAGGTGCAGCATTTTC

AAGTTTTGGAAGAGAAAAAAATTGTTCATTTCAGGGTAATTTTGTCATATTCTGCCTTCA

GTATGTGTGACATCTGAGTGTGTTAAAGCAAAATGTAC

>Unigene18610_All AAAGGATCAAGGCACAGACAATAACCATGTCAGAGGAGAAGAACCTAACTACAACGATCA

CAGAGTTCATCTTGGTGGGCTTTCCCGGGCTGCATCCCAATTACCATAAGCTTATGGGAT

TCGTACTCTGTCTTCTCTACGTCAGCATTGTAGCAGCAAACACTACCTATGTGGTCATTT

TTTCACTTAAGCCTAGTCTCCATAAACCTATGTACATTATCCTAGTAAGTCTGGCCCTGT

CTGACATAGGATTCAGCACTGCTGCACTACCAAAGATAATTGCACGCTACTGGTTTGATG

ATAAAGTCATAGCCTTCCATGCATGCTTCATGCAAAGGTTATTCATCCATAGCTTTGGCT

CTCTTAACTCCTTCATTATGATGATCATGGCCGTAGATCGTTACTTAGCCATCTGCTTTC

CTCTCAGATATCCTGTGCTGGTAAAAAATCACACCATGGTCATAGTCAACTGCTGGGCCT

GGGTCTTCTCTTTGGTTCCCACAGGTGTTTCCTTTATATATCTTTATATGCTTCCATATT

GTGGGCCCAATATAATCTACCAGTGTTACTGTGACCAAAACTCCATCCTGAAACTGGCAT

GTGCTGGCCAAAGCTGGCCTACATTGATTGGCTTTTGTTTGGCCATGCTGGTGCTGCTGA

TACCCTTGGCTTTCATCGTGTACTCCTACTTGCACATCATAGTCAGTGTGGCGAAATTAA

AGAGTTCAAAGGGGCGATGGAAGACATTTTCCACCTGCAGCACGCAAATGTGCATCATCA

CCATTTACTACCTGCCACGCTGCACCGTCTACGCGTTAAACCTGGTTGGGATATCGATGA

ATATTAATTTGCACATCGGTGTCGCTCTCATCTACAGTTTGTTACCCCCACTTGTGAATC

CTCTTATCTACTGCTTCAGGACCAAGGAGATTCAAGAGACACTGGTTCGATTGATTGGTT

TTAAATGGGTGGGGAAGCGCTCAGTTGCTGCTGTTTCATGATAAAAATGTTTTTCTGTGA

TGTTAGATCACAACATTTTATATGTTGCCTGGTAAATCAGATGTGCTTTACATTACTTTT

GGCTGTTTTACTTTTATTTCAGCATTTCATATGTTTTGTCACTAATTATTTAATATGTGA

AAATATACCTAAAGTGCTGCAAAAATATAAGTCAACCAGCTACAGGCTATAAGTCAACCA

GCTACAGGCATTTCTAGCTGTCACTACAAAACAAACATCTCTGCTGTGAATTAGAATGTG

TGTTATTAGACTATATCACAAGCTAGCAATAATGATCTGGCATGTAAAGACTGTAAACAT

AAGTAATGTACCTGTAACGGTTATAATATTTCACAGTTGCACTAGCACCTATTACACCCA

ATTAGACACACAGAGAAAAACTGTACTTTTTCCTTACTTTTTAAACTGTTGAATGGGAGA

TGTGTTTTGACAGTTCCTTACTTTATAAGCAGCAGGACAATGATCATGAGGGCATTGTAA

ACAGAATATTAATTTCCAAAGCATAGCCTACATTATGTATCTTTAAAGTGTAATGTTGCA

AGCAACTGCTGAGACATATTGTGGAAATCCTCACAAATATAAAATAAGTTATAATTAGTA

AATCAAACGAATAACCGTGGTCTTTCTGAGACGTTCTCAAGACTAATTCTTCA

>Unigene21223_All TATACTCATATTGTTCAAAGTTATTGTAATTGCTCTAATGCAATATGATCACTTAATTTC

ATGTTTATGTTTGCTCAATTTCAACACAGTTTGTTTAACGAAAAGGAGTGAAGGGAGTAA

ATATTATCTGAAACAGACGGAGCCCAGTCCACGAAGATGGACAATGTTACAAATGTGTCC

TATGTTCTGATGCTACAAGGCTTCGATGTGCCCCAGGAAAGGGCTTTCCTGGCTTTTCTT

TTGGCCGTGCTGGGGTACATGGTTATAGTCTTCTGCAACCTTCTTCTGCTCCTTACTGTC

ATTCTCGACAGGGGCCTCCATCAGCCAATGTACATTCTTCTGATTAACATGCCGATCAAT

GATCTGATCGGCTCCACTGCTTTATTTTCTCACCTCTTCAGGGATTTTTTGTCCAACTCC

AAGGCCATAGAGTTTCCTGCCTGTGTCATTCAAGCCTTTTTCATTCACATCTATGCCGTG

GGAGTGGTGTTTAATTTAGCAGCGATGGCCTACGATAGATACATTGCTATATGCAGACCA

CTGCAGTATGCCTCGATAATGACAAATGCTCGCCTGATGACAATCATTGCACTCGTATGG

GGTGTTAATGTGCTTATCATTGGTGTTCTTTTCATCCTTCTCCTAAGGTTACCTCGCTGT

AGGAATAATGTGACCCACACTTACTGTGACAACCCATCACTACTGAGGCTGATGTGTGGA

GACACAACAATTAATAATATCTATGGTCTTCTGACTGTGACTTGTACACAGCCGATTACA

GTTGGTATAATTCTGTATACCTACTTGCAAATCCTGATCACTTGTTTTCGAAGCAAGAGT

ACTGACACCAGAGCCAAAGCCATGCAAACGTGTGCTACACATCTAGTTGTTTTTCTGCTG

TTTGAGTGTCTGGGGCTTTTCACTATCATCTCGTACAGAATAAAAAATCTTTCTCCTGTT

CTGACAAACTTCATCGGTGTTTCTGCACTAATATTCCCTCCTACCTTGAATCCAATTATT

TATGGAATCAATACAAAAGAAATTAGAAAAAGGGCCATCATGCTTTTTAAGAAATATGTA

TCTCCAATGTGAATGTAGAATGTAAGCTTTTCTACCTTTTGGTCTGAAGACACACTGTAA

TGATTTGTTTGTCAAGACGTGAACAGTACTACTAAATTATGAGTTGATGTCAGAACTGGT

CGCATAACCACATAACCTGTTTCTAGGAATCTACATTTACCCTGGCATTGCATCAACAAT

GACTGTGATTTACCTTTGCTTAAAACTGAGAATTAAGATCTCACAGAGAATTTAGAATCT

GTGTTTCTAAATCACTGTAAATGTTGTTACTTTGCCAATTTACACAATTGTAAAATAAGC

AGTGTATATTCTACATAGTTATTTGTATTATTGTCCTATTGGTATAAGTGTTTTGACCTT

GGTCCCAAAACAAAAAGTCATATCTAAATGTTTGTATACACAATGCTTATTTTCATTACT

GTGAGAATGCAGTCGATGTGTTTACATTTTTGTAAGATAAGGGTGTTGTGTAGCCTTACT

TTCATTTAGACAGGTCCAACCAACGGCCCTTTTTAAAGAATGACATTTTATTTGGATGTT

CTGGAACCCTGTATGTGTGTGTGTGTGTGT

>Unigene22779_All

CTTTTCACTGTTGCATCTACTTCACTGAAAAATGTCTTAAAGACTGATAAAGCTTATAAT

GGGAATGCAACACTGAACATTTTCAAGTCTCGTCAGTCTATTTATTTGTACATGGTCTGG

ACAAAATGTATTTTTAAGTATTTTATTGTGTTTTATTTTTAAACTCCTGTTGAATTCTAA

CTCGATCAAACTTGTTTTTTTTTTGTGCTGTTGTTGTTGTTGTTCTTTTGCACTGTTATC

AACTGTAATGGACAATTTTACCTTCAACTTTGACCTCACGCTGGACCCTTTTTCTATTCC

AGCTGGGGCAAGATATCCCATATTTTTCATCGGGCTTTTGATTTACCTCTTCTGTATTTC

CTGCAACATGACTCTGATCGGACTCATTATAACGAGGAGGAATCTCCACAAGCCCATGTA

TTTCATATTATTTAGCCTACCGCTGAACGACCTCATAGGGATCACTGCTATGCTGCCCAA

AGTCCTTTCTGACATTGTGACTGAGAGCAATCAGGTTTACTATCCTCTTTGTGTTCTTCA

AGGCTTTTTACTTCACATGTATGGTGGAGGAGTTCTTTTCATCCTTGCGGCAATGGCCTT

TGATCGATACGTTGCCATCTGTTTCCCACTGAGGTACAGTTCAATCATGACACCCAATGT

TGTGGTGACAATTATTGCAATTGTGTGGGGTCTGGATTTTGTCCTGATATTAGTACTGTT

TTCTCTCCAGGCCAGGCTACCTTTGTGCAGATCACAAATCATGAATGTGTTCTGTGACAA

CCCATCTCTTCTCAAGTTGACATGTGGGAATACCTTTATCAACAACATTATTGGCTTGTT

TAACACAGCTCTTATGCAAATAATAAGTATA

>Unigene22851_All GGCGACACGTTGCAAATGAAAAGAAGTATGTTGTACAACATATGTGGCTCTGCTATTATT

GTCATGTTCCCCATATTTTTGATTTTGGTGTCATATATGAAAATTATCACATTAATGTTA

AAATGTTCAAAATGTGCAAGACAGAAAATACTGGATACCTGTTTGCCACATGTTATGGTT

TTTGTCATTTTTACCCTATCCTCAGTGTTTCCTGTCATTTATACTCGCCTCAGTTCAGGC

TTACTCATGCAGGCCCACATATTCATGGCTATCATATACATCCTTGTCCCTCCACTTCTG

CACCCAATCATATATGGAATAAAAAATCAGGAAATCAGACACAGTTTCTCAAAAATGTGG

AAAAGAAAAGTTTTGTTCATCTGAGTGATATTCTGTCATCAGAATGTTTGACACCTCAGA

TGTTGGATGCAGAAGTATATGTATATGTATTTAAGCAGCATACCAC

>Unigene29409_All CACTGATGGTGAGTCCAGGCTCTTTTAAAGATTCCATGTACTGCAGTCGAGAGAGGATTA

TCATATCACCATGGCAAATAGATAAGTTTCAGGGCTTTAATTACTTTTTCTTTGTGACTG

TAACAGCCATTTTAATTTTCAGCTACATTAATGTTGTAGTTGCAGCTCGATCTGCCTCAT

CACAAAAAGACTCTGCTAAAAAGGCACATAAGACCATACTGCTGCATTTTGGTCAGTTAG

TACTCTGTGTTAACTCGTTTATTTATGGAACCATAGAGAGAGCCTTGGCTATGACTAGCA

GTAGTCGTTTGTTCATGGACCTGCGTTATATGAACTTCCTCTTTGTTCTCCTTCTGCCTC

GGTGCCTGAGCCCCCTGATCTACGGCCTCAGAGACGATGCTGTTCGACCCTTATTTCTCT

ACTATGTCAGGTGTGGATACCAAAAGGTGAAGCCCTCTGGAACCATGCACTGATGTTGAT

GAGATATCAAACTACAACAACAACAAATAATAGTTCAATTTTTATTTTTTTTAATATACT

TTTTCCACTGTAACGCGTTTGCTGTTTCTTAATGTTAATTTAAAATTGAGCAAGGATATT

TGGGAAACAAATGTTTTGCAAGCTTTGACACTCGGCTTAAATCTGGTAAATGTAACATAT

TGCATGGTAAGCTAGCAACATGTAAATGCTATGGTCTTCACATTTTA

>Unigene30140_All ATTCACTTATTTCCACAGTTCCTTGTGATTTTGTTTTCATATGTTCAAATTCTCAGGATC

TGTTTAAATGCATCCAAAGAATGTCAGAAGAAAGCACTTCAAACCTGCACACCGCATTTG

TTGGCAGTTATAATTTATTTTTTTGGTGCTATTTTTGAAGTAACCCAAGTGCGTATAAAG

AATAACCAAATTCCATACGGCATAGCAGTATTTATGTCTGTCTATTTTCAATGCACTAGC

ACCATATATCACAGGATTTAACAATG

>Unigene30141_All ATTCACTTATTTCCACAGTTCCTTGTGATTTTGTTTTCATATGTTCAAATTCTCAGGATC

TGTTTAAATGCATCCAAAGAATGTCAGAAGAAAGCACTTCAAACCTGCACACCGCATTTG

TTGGCAGTTATAATTTATTTTTTTGGTGCTATTTTTGAAGTAACCCAAGTGCGTATAAAG

AATAACCAAATTCCATACGGCATAGCAGTATTTATGTCTGTCTATTTTTTGATCATGCCA

CCATTGTTAAATCCTGTGATATATGG

>Unigene46725_All GAAGGGTTATGAAAATGTAAGAATACTTTATTTTTTGTTAACAATAACGGCATACTCTTG

CATCATACTTTTCAATGTAACTATCATACTGGCTGTATTCAAGGACAAGTCTCTTCACGA

ACCCATGTATATTTTAATGTCTTGCCTGTTTTTCAACTCTCTGTATGGCAGCTCTGCACT

TTTTCCGAGACTTTCTGCTGACCTTCTATCAACAACTCACACAATA

>Unigene48164_All

ACATAATTACTTTTGTAAATTTCTCTCTGGTGTCCTTGTTCTCTCTCTTCTATAACCGCT

ACAACTCATTCTTACCCGGAGGGGTCAACATTCTGATGTCAATTAATTACATTCTTGTTC

CTCCACTGTTGCATCCCATTGTGTATGGTATAAAAACAAAGGAAATTAGACAGAGATTTT

CAAAGCTTATCAGAAGTAAGTTTTGATTCATTTTTTGTGTGACTGTGACTATGCTATGTA

CGAAGATTGCAGCTGTTTGAAATTCATGCTTATGTGTAAAGGCTTGTTGGAGGCATTTTT

GAAAGCTTGG

>Unigene49175_All TGCCCAATAACTACTTTAAAATATTTTAAAATATGATGTTGAGGTATCTGCAGACATGAA

TGCTTTTTTGACCAACGACACTTTATTTTTTGCTGTTTATGCAGGCGTTGGCTCTATGGG

ATATGTATTACTTGCTGTTGTTCTCCTGGCATACCTTGCCACTGTATTAGCCAGTGCTAC

TGTAATGATGTTGATTTTTCTAGACACATCCCTACACAAGCCAATGTACATATTTTTATT

TAGTTTAATTTTAAATGGGTTGATCGGAAGCACAG

>Unigene54358_All

ACAACGTAAAGCTATTTCCACCTGTGCTCCCCACCTTATTATTTTCATCAATTTTTCTCT

GGTCGCTCTTTTTACCATTATTTATAATCAGTTCACAAGTGATGTATCTGTCGACATGCA

CATATTTTTTGCCATGCAGTTTAGTCTAATTCCTCCGCTCCTGCATCCCATAATTTATGG

TGTCAAAACCAAAGATATTCGGAAATGTATAGCAAGGCATGTAGAGAATGGTGTAGATGC

CACAAGTAGATTTAATACTCATCATAGCTTTTCACAAAACATGAATGGAGACAAACAGGC

TCAAGTCTTTGCTGTTAAGTTGTAGAAGACATGGAATGCACGCTCCCAGTATTTTTCATG

CTGGTACAGGAATTATTGTTGTTTGTACATCAAAAATAAAGCTAAGCTGCTTGTTTGTGG

GCTTTTTAATGTCTGTGTGACCACAGGTCGATCATAACTGAAAATACGTGTATTCTTCAG

ACCGCCAGCACTTCGCCAGATAAGAGGTGTGAACAGTTTTCTATACAGTCATTACCAGCC

CACAGACTTACAGGTCTTGTAAACTTTGGACAGCGG

>Unigene54574_All

TATGTGGTATATTTATCAGGAATAATGAACCAATCTGCTTTTGATGTCATTGTTGTTTTC

ACTGCTTATCACTCCATTGGCACCAACAAGTATGTATATATCGCTGTCATTCTCTCCGTG

TACATGGCCTCAGTGTTTGTTAATGTCTCACTCATGCTTCTGATCATCAGGCAGTCCACG

CTCCATCAGCCCATGTTCATATTCATGTTTAATCTGACCCTCGTCGGACTTATAGGGAGC

ACTGTAGTTTGG

>Unigene64615_All

TTTGCTCACATGCTCTGCAATGACACGGTTCAGATGGCCGTGACATCTATGCTCTTTATC

ATGGTGATGTACCTGCTTCAGCTTACTAAAGCCTTATGTGCTGTTATTGTGTACGTGTCG

GCGGTCACCTATCGTAACGCTGCTTTCAATGTTGCGGTGATGTCTCTGGAACGCTACGTT

GCCATCTGCTTCCCACTAAGGCACAGTCAAATGGTGACCCACAGTGCAACTGCTGTAGCG

ATTAGTGTCATGTGGATGCTATCCTCGATTTGCCCAGTGATAGACATATTTTACGGCATA

ATCATGGACCCCATGTTTTTCAGTGGACAGATTTTTTGCTTTCGAGAGATGTTAGTTAGA

ACTACCTGGCAAATTAAAATGTTTCAGACTGTTAATGGAATGTCCTTTGTGTCAGTAACG

TTAATTATTATCTTTACTTACATCGGTGTCATAATTGCAGCACGTGGTGTGTCTACAGAC

AAAAGCACAGCCCAGAAGGCAAAGAGGACAATCCTGCTGCACTTTATCCAGCTTATTTTT

TGTCTGACCAGTTTGCTGTATGGGTCTATTGAAGGTTTGTTAACTAACTTATCCAATGTT

GAGCTTTTCCGTAGTCTGCGGTTCGTAAACTTCCTGTTGATGCTCATTCTGCCTCGATGC

CTGAGCCCCCTGATCTATGGCCTGAGAGATGATGCTGTTCGACCCTTATTCCTGTACTAT

GTCAGGTGTGGATCCAAAAAGGTCAAACCCTCTGTAAATGCACATTAGCAGACTAGACTA

GGCCATAATCCTTCAGTGGTTGAAGTTTCAACACAGTAACATCTATGGCTGGAGGTAATA

CAGTCAGTGTATGTTTGCAATCCTTAAAGTAAATTTTTTTGTTTCGGGTGAAAAAATATA

TTATTCATTACATTGTTATGTATATCTGCTTCTAGTGATGAATGTTTTGTTTGCATTGGG

G

>Unigene64616_All

ACTCTAAACACTGTTGATGAATTGACAAAAATCTGTGACGTCACTCTAGCTACTGTTGGT

AAGCTGTCATATAGCTGTCACGTCACAGTTGATAATTTTTCATAAATTTGTGTTACTGTA

CTTGCTTGTCAGCCCCAATGACTAACAAGTCCAGTAGTGAGATTCAGTACCTAGAGCAGG

TGGCCTACACACTCCGGATTCGTGATGACACACTGATCAAACTCTCCGTGGTCGTCCTGG

CATCTCTGCTCTTCATCTACATTAATAGCATGATGTTCTACACTCTCCTAAGTAGGCCTG

TGTTTAGAGAGCTGCCCCGCTACGTCCTCTTTGCTCACATGCTCTGCA

>Unigene67012_All GATATGGTTAAAAATTTCTCTCACATCCCAAACATCACAGACAAGCCAACAGGTCTACTC

TTAAGGCCTTTAAATGAGAAGATTCTGCTTGTGCAGGTACTGGTGGGGATTTTTCTCTAT

GTCAACTCTCTCATGATCTTCACCTTCCTGAAGAAGGAGGCCTTCAGGACAGACACGCGC

TACATCCTCTTCGCTCAGACGCTCTTCATGGACTCCTCTCTGATGGTGCTAACGGATCTG

GCGGTGATCCAGAGCTACTTCCAGTACACCATACCGTGC

>Unigene7032_All GTGTGTGTGTGTGTGTGTAGGAGAAAGAGTGAGAGAGACGAGCGAGAAGCTGCCCCTTTC

TCTTCTGTTTCTCTCTGTGGGATGTCATGGCTCTGAATGGCACTGCCCAGGGGGTTCTGA

TTGCTCACCAGCAGATGTTCCAGGTGCAGCTGACGGCTCAGGGTCCCCTGACCAAGCTCC

TGGTGGCCATCCTCATGTCCGCCCTCTTCATCCTCATCAACACCATCATGTTCGTCACCC

TGCTCAGCAAGCCCGTGTTCAGGGACACGCCTCGCTACATCCTCTTCGCACACATGCTCT

GCAACGACTCCATCCAGCTGCTCTTCTCCTCCATGATGTATATCATCTCCCTGTGCTACG

TGCAGGTGGCCAAGGCCGTGTGCTCCATCCTAATCCTGGTCACGGCCTCCACCTCGCGCA

ACGCTCCCATCAACCTAGCGGTGATGTCGCTGGAGCGCTACGTGGCCATTTGTTTCCCCC

TGCGCCACGCCGACATCGCCACCAAGAGTGGGACGGGCGTGGCCATCTCCACCATCTGGT

TCTTCGGGGCAGTGAACCCCATCATAGACGTCCTCTACACCTCGGCCACCGACCCCTCCT

TCTTCAGCGAGCGCATGTTCTGCACGCGCGAGAGAATATTCATAGCGCCATGGCAACGGG

AGCTGTTCGAGGGGCTGAATGCATTTTACTTCGTAGCCGTGACTCTGATTATCGTCTTCA

CCTACATCAGCGTGATGGTGGCTGCTCGCTCGGCCACCAGCAATAAAGAGTCGGCGAAGA

AAGCCCACCGCACGCTCCTGCTGCACCTCGCCCAGCTGGGCCTGTGTCTGAACACGCTGG

TGTTCGGCAGCATAGAGAGGCTGCTGGCCATGACCAGCAGCAGCCGTCTGTTCATGGATC

TGCGCTACGTCAACTTCCTGTTTGTCCTCATTCTGCCCCGGTGCCTGAGCCCGCTGATCT

ACGGCCTGAGAGACGATGCTGTGCGGCCCTTGTTCTTGTTTTATCTGCTGTGTGGCACGC

GCAAGGTCAAGCCGAAGGTCAATGTACACTGACTAACAAGCATGACTTAATGCCAGGGCT

TGTATTACACACACACTCTCCTTCTGATGATTAATGTATAATCAGCCCACTGATAATGAA

ACTTTGGGGAAAACGTGTTAACAGTGGTAGCCTACATGATATAAGTTATATGTCACAATA

ATCAGCTGCCATGTTTGTTTTGTTTCATTTATTGTGTTCATATGTTTTTTAACACTGTGC

TGCTTGTTTAAATGGTTTTTGTAGCACTTGCGCTATGTGTTTCTTGTTTAGTCGTCTGAC

TGTAATAAACTGTTGTGTGTAGAAACATGCTTGTTTCTTTTTGTGGCTTATTTATTTTTT

CTAACTTGAATGTTTCCCTATGTTGCAGTTC

>Unigene74121_All GAAGAATGCATATTTTATTATCGTCTTCCTAGTTTACCTATCATCAATCATAGCCAATGT

TTTTCTTATGTGTTTGATTTATTTGGACTCTGCTCTACATAAGCCAATGTATATATTCCT

CTTCAGCCTCCTGGTAAATGGGTTGATCGGAAGCACAGCTGTGTGGCCTAAGGTTATGCT

TATCCTCATAACTGGTGATAACACCATCTCTGTTGCAGGGTGTTTTACTCAAACATACTT

CATGATAATATACGGGGCTTGCAACTTCACAATCCTAGCAGTAATGGCTTATGACAGGTT

GCTGTCCATATTTAATCCACTGGAGTACCACGCCTTAATGACACCCCAGAAAATCAGACA

GTTGCTGTTTGCTTCCAGTGTCATGCCCGCGGTGGTCATACTTGGTCACATATGTCTTAT

TTCACAGATGTCTCTCTGTCAATACAGAATTCCTAGATTGTTTTGTGATACTTTATCATT

TTTTGCATTGTCATGTGTTGAAAACATTCAGAGTCGAGTGAGCAACTTCTATGGATTATG

TGTGTTTATTTGTTTTGGAGCCCTCCCAATAGCGCTTGTTTTTCTGTCATATTTTAAGAT

TATAGTCTTCAGCTTAAAAGCTTCAGGAAATGCAAAAAGGAAAGCATTTCAAACATGCAC

TCCGCATTTGATTATTTTTACAAATTTTTCATTCACCTCTTGCTTTTTTGTCATCTATGT

ACGTTCCAATCCTAATTTACAAAACTTGTACCTGCCCATATTTTATAACCTTTCTCCCCC

TCTCTTGCACCCGCTTGTCTATGGCATAAGAAATAAGCAGACCAGACAGAGCTGGTCCAA

AATCAAAAAAAGAATTATTTTTACTCTCAATGAATATCACAGCACGTGCAAATGTTAATT

TGAATGTCGTTGTGTTGTTGTATGTATGGAGCTGAGTCAGACAGCATAGTTAAAAGCTTT

TGGATTGTATGAAATAGTTCATTTAAGTGTGCATGTGTGTGAGAGCATACATCTTCTCAG

GCGTTCTTTGGTCAAGGAGGTATACATTACATTACACTTAGCTGA

>Unigene76039_All CTTAGATTCGTTCCTCGGAGGCTGTAATGCTGGCAGTATCATATAAAGAGACGTATTTGG

GAGGATACTTAAAAACCCAGTGTATTACACACTGAACTTGTTTTTTAATTATAATTTTTT

TTTGCTTCTGGTTGCAAATACCACGTTTTTGAACAACATGCATTTTTAAATGTTTAATGT

ATTTGGCTGGATTCAAAGTGTGATTTAATGCTTTCTGCGGTAAAGGTTTGACATTGTTCT

GAGATGGCTGTTATGGAGAATGTTAGTATTAGTTTTGAACTAAGTTTGGATCCATTTTTT

ATTCCAGCTGGGGCGAGGTATCCCATATTTTTTGTTGGCCTTGGGATTTATCTCTTTAGC

GCCGCCTGCAATATGACTCTGCTTGGACTTATCATCGCAAAGAGGAGCTTACACAAGCCC

ATGTATTTTATTTTGTTCAGTCTACCATTCAATGACCTTGTAGGGATTACTG

>Unigene81937_All

GGGATATTGGCATTGGCATGGGGTCTGGACTTTGCCATGATTTTTGTTCTTTTTCTCCTT

CAGAGCAGAGTGCGTAGGTGCAGAAATTTCATCATGACTGTATACTGTAGCAATGTCTCC

TTGCTTAACCTGTCCTGTGGAGAGGATACCACTATCAATAATATCTATGGATTAGCTATC

ACAGCATTTATGCATATCATCACCATAACAATACAGCTGTTCTCATACATTCAAATCCTC

TTGACATGTTTCTTTAACAGACAATCAGATGCCAAAACAAAAGCTGTAAACACATGTTCA

GCCCAGATTATTGTGTTTGTAATTTTTGAGATGGTTAGTTTGTTTATTTTAATTGCATAT

CGGCTTCCAAATGTTTCGACAGATGCCAGGAATGCCTGTGGGATGATGATATTTCTGATT

CTACCTGTCGTCAACCCCATTATCTATGGAATGAAAACT

>Unigene81949_All TGGAGAGGGAGAGATCAGCAATGAAACAGACTCCATCACGTGTGTTCTTTGCCATCAAGA

ATTCTGGTCCAACAGACAAAGGGATAAATGTCTGAAAAAGGAAACCGAGTTTCTGTCATA

TAAAGAGATTATGGGTATTCTTCTGACTGTGATCTCAGTCATCGGGACCTGCTTGACATG

TGTGATTGCCATTATATTTTTCAAGTACAAGCGCACACCACTTGTTAAAGCTAACAACTC

TGAATTAAGCTTTTTGCTTTTGTTTTCACTGATGCTTTGCTTCCTCTGTTCTCTTACTTT

C

>Unigene84414_All

CGCACACATGCTCTGCAACGACTCTATTCAGCTGCTCTTCTCCTCAATCATGACCATTTT

CTCATTTGCCTACATCAGACAAACTAAAGCTGCATGCTCATTCTTACTCTTCGTCACCTC

CTCATCCTCCACAAATGCTCCCCTCAATCTGGCCGTCATGTCACTCGAGCGCTACACTGC

CATCTGCTTTCCTCTGCGCCACAGCGAATTTTCCACCACTAAGAGAACATATGTGG

>Unigene85149_All

TACGTGGCCATCTGCATGCCTCTGCGGCACGCCGACATCTCCAGCCCCAGGAACAGGCGA

CGAGGCCTTGCGATCATATGGGTGATCAGCACGCTGCTGCCAACGTTTACGGTTGTGTCG

TTTATCTCGCTGGTTCCACCCACCCTCCTCCTCACCTACGTGGTCTGCAGTATTGAAATG

ATGTATGTGCAGGCTTGGCTGAACCACGCACACAATGCAATCCTCCTTCTATACTTCTCC

GTCATGTTCATAATCATTGTCTTCACCTACATGTCAATCTTTAAGGCTGCGCAAGCCGCC

TCGTCTGACAACAAGAAGTCCACGCATAAGGGGCTGAGGACAATCATTCTACATGCTTTT

CAGCTGCTGCTCTGTCTTGCTCAGTTTGTGTGTCCTTTTGTGGAGATGGCCATACTTAAG

ATTGACTTCATGTTGTTCATCAACTTTAGATACTCACACTTTATTGTGTTCCTCATTGCC

CCTCGCTGTCTCAGTCCACTCATATATGGGCTGCGAGATGAGAAGTTTTATGTTGTGCTG

AGACACTATGCTTTTTTTGGTCTCGACACTGTTGTCCTCTCCCTTCTAAGCTCAGAAAAC

ATCAAATTCAGAGGGCGTGCCAAGACAGGGCCTTTGCAGTCTTAACCTGGTCTTAACGCT

ATGATTCCACATCATTCCCTGTTTAGCTTGCTCACTGTGTGTGTCAGGCTAAATGATGAC

ACAATGATCACAATGAAAGACTAATGCTATACTAACGAATGTGTAACATTTTGCACAGCA

CTGTACACACTGTCACCTGTGTGTATGCACAACAATAAAACTGCAATCAATCTTTTCTAC

ATCTTCCAGCTGAAAAACAGAAGATGGACATCCTAATGTGGTATTTTCAAACAGATGAAT

AATCATATTGAAATTATCACCCCAAAAAAC

>Unigene87980_All TTTTTAACTACCTTGCCAGTCGAATTACTAATAATTTTCTCACCCCAATCTATCTAACTC

TTATGTCCCTGGAGCGCTACGTAGCCATATGCTTTCCCCTCCGACATGTTCAGATAGCTA

ACAAGAGGAGAACCATGGTGGCAGTAATGGCCGTCTGGTCACTAGGGCTAGTAATATGGA

CAACGGACCTAACTGTTGCCATGGTGTTTCAGGCTGGCTCCTCCAAAAGGAGCTGTTCTG

ATTACATACTTTCCCAAATGGTAGTTTCCTATCAGGTAAGCACAGGTTCAATAGCCCTTG

TGTTCACATTGGCATGTGTTGTGATAATCTATGTATACATAGCCATTGTGGTAACTGCCA

GGTCAACTACTACCAGTGACAAGTCCTCTGCGAGTAAGGCCCATAAAACAGTACTCCTGC

ACATGGTGCAGCTTTGCTTGTGTCTCTCATCGCTTCTAATGGGAGCGGTTCGCAGGACAC

TAGTTATGAGCGGGCTGGACCGAGTGCGTTATGATGAAGTGTCGTACGTTTTGTTTTTAA

TGTTGAACATCTTGCCTCGGTGCCTCAGTCCCATCATATATGGTCTAAGAGACAAGACTT

TCTGTGCCTATTTTAAGGTCCATTTTCTTTTCTGTATCAAA

>Unigene88695_All TAGGTTATGGCTCTCATGACCAACAACACAAGAGAAGTATTTCAGGAGATGATCCAAATT

GAGATGGCACTTGATCGCAGAGTGTTCAAAGTCACTGTCACCATAATCATATCTTTCTTC

TTTGTTTACATTAACACTGTATTGTTTGTCATACTCCTGAGCAAACCAGTGTTCAGGGAC

ACACCTCGTTATGTGCTCTTCGCACACATGCTCTGCAAC

>Unigene9043_All CATGCTTCTAAGCATCACATTCTTCTATAACATGCATTTCTGAACATGTATGAGTGAATT

AATCAATGCATTTGGCTTTCTATTAATGCTGTAGAATGAAATCTGTGTAAAACACTTAAC

ATTGTTTTAGACTGCAGTCATGTTGAATGCAAGTATTCATTTGGAAGTAAGTCTGGATGC

ATTTTTTATTCCGGATGGGGCGAGGTATCCCATATGTTTATTTGGGCTTGTGATTTATCT

GTTTTGCATTTCCTGCAACATGACTCTGCTTGGACTTATCATTGCAAAGAGGAGCTTACA

CAAGCCCATGTATTTTATTTTGTTCAGTCTACCATTCAATGACCTTGTAGGGATTACTGT

CATGCTTCCCAAAGTCCTTTCAGATATCTTCACAGATACAAGCAGCACCTATTATCCCCT

GTGTGTTCTTCAGGGCTTTCTGCTTCACATGTACGGTGGTGGGATTCTTTTCATCCTGGC

TGCCATGGCCTTTGATCGATATGTTGCCATCTGCTCACCATTAAGGTTTCAGCATGTTTC

ACCTGACTTACAGAAGATTATGGGCATGTTGATTTTTCTTGTTCCTCCTATCCTTAATCC

GATTGTGTATGGATTGAACTCTCATGAAATTCGAAGGAACCTCTTTGGCCATCATAATGA

AGTGTCTACATAAAATGTCTTATGTACATCACCGGCTCCCATGCAGTAGGCCTAAATGTT

GTACACTGAGAAATGTGTATTTGATGATGAGAACTCCTACCGTGCTCAGACCCTGTTAAG

GCCAAATGCCTCCTTTTAACCATGTTGATAAAAATGTAAATTTAAGATTTAAATTCTCCC

AAACATTTCATGTTTTTTTTAAAGAAAGCTTTTAGACCCCTGTGCCAGGTGTCATGTTTT

AACAGCATGGCAGGTTTTGTGAAATTCTGCAGAGAAAAGAGTCAAAAAAAGAGAAAGGAA

ACATAACAGCGTTGACAGAGGTGGTGTGGTAATATGCATCCACTTACAGTAGGCTTATGT

GCGGAAGAATTTTCTGTTTTGTTTTGTGTGATATTAAAGTGCCATTGAAAGGTACTACAC

AGACAAAGATTTGTATTGTAGCTTAAGTCATGTTTTATATACCAATTAGCAGTTACTGTA

CCAATCTGAATGTTTTTTCTTGTGGTTCGGAAATGATTGTGAGCTAGTAGTTGTTTGACA

TTACATTGACATTTTACAACACATTTTCTGAATTGTTTACCAGTTTGTTTACTTACTGTA

ATTTGTATTTGTTTACTGAGCATATTATACACTTGTAAAGAGTGCATTTGTCAAAGAGTG

CACTTTTGTGCCTGTTCCCTTGGACTAAGAGTTTTTGCCCTTTGGATTGTTTGCCTGTAT

GACTGAACATTGTCTCCTAATTACAAAGTCTTATGTCCCTGGATTTGTTGCCTGAAACCT

TTTGAGTAAAGTTTTCACTGTTTTGAAAAAA

>Unigene91230_All TCTCCACGCCAGCAGGTGATCGGCGCAGCAGGCAACGGCATATGCATGGCCTTTGTGTGG

GTGACGCTGGTCTACACGTACGCACGGGTGCTCTGCGCAGCCCGCGCTGCCAGCTCGAAC

AAGGGCCAAATCCAGAAGGCGCAGAACACCATTCTGCTGCACGCCGCCCAGCTGCTACTT

TGCATGCTGTCGTATGTGGTTCCGGTGCTCGAAAACTACGTGCTCACGATG

>Unigene95547_All

AGAGCAGCCACGTGCGACGCGGCCTCTGCCAAAAAGGCGCAGAGTACCATTTTACTGCAC

GGAGCTCAGCTGCTTCTCTGCATGCTGTCCTACATCACTCCGTACATCGAGATGGCTTTA

GTACCTTTCTTCCCAGTCCATCGCTCCACAATAATGTTCCTGTGCTACCTGATCACCATG

ATCCTTCCAAGGTTGCTCAGTCCACTCATATATAGTATTAGGGATCAGAAATTTGCCAAA

TGTATGTCACAGTACTACTCCTGTAGGGTCAACAGGCCAAAGGAACATCGCAAGAGGAGG

GGAATGAGCTCTTTCAGCAAGAAAGTTTGTTCACTGAAAGGTTAATTCCATATTGCATTA

TGTCGTGAAACAAACATCATGGCATGGGACGATGCTGTAATTTATAAAAAAA

>Unigene99783_All

GAGAGATACTGCTCCCTGGCCGTGTATGGCACTAACATGCACCGCATAGAGGTATCTTTC

ACTTTTTTCTACTTCGTGAGCTCGTCTGTAATCATTACATATACGTACATCGCTATTGTC

AGAGCAGCCAAGTCATTAACCGATCAAGCAAAGAAATCAACCTCGAAGTCTAACGAGACT

GTGCTTCTTCACCTGGTTCAGCTCAGCCTGTGTCTCACCTCTCTGTTCTTCAACTTTGTC

ACCAAAGAAATCAGACCTAAGCTTGGAGAGGCGGCATTTCTGAATATACAGTATTTTCTG

TTTATGATATTCATCATCTGTCCCAGGTGTCTTAGCCCTCTCATATATGGCCTGAGAGAC

CAGGCTTTTGGCAGCTTGTTTAAATATTATTTTTTATTTGGCACAAAAGCTGTGGTTAAG

CCTACGTCAACCCAG

>Unigene14954_All

TTTTGATCATAAGAGTCTTGGATTAACACTTCATGTATTGTAGTATGGTGAGACTTGCTT

GTGTTCCCTACTATACTGTGAATCCTCTACTGATTTTACACAATGACTCTTGGTATGATG

ATGTGCAATTACTGACTTTTATTCATGTGATATCGATTTAATGCTGTTTTTCACTGGGGT

GATAAAGGGACATACACGAAAGACATGAACTTCTCCAGCAATGCAAGCATGGTCAGACCA

CTAAGGGACACTTTTCAGACGGCGGTGACCAAGAATGTGGTTGTGGTGGCCCTGTGCCTG

TCCATCAACTACATCAACGGCACACTGGTGCACACCTTCTTCAGGCACCGCATCTTCTCG

GAGAACCCGCGCTACATCCTCTTCATCCACATGGTCCTCAACGACATGATCCAGCTGACC

ATCGCCGTGCTGCTGCACGTCATCAGCTACGCCGTCTTCACCATCAACGTCTCCCTCTGC

TGCTTCCTGCTGATGATCGCCGTCTTCACCACGCTCAACACGCCGCTCAACCTGGCCAGC

ATGGCCATCGAGCGCTACATCGCCATCTGCAACCCGCTGCGGCACGCGCAGATCTGCACG

GCGCGGCGCACCTACAGCCTCATCGGCCTCATCTGGCTGATGGGCGCCATCCCCATCCTG

CCCGACCTTTTCATCCTCCTGGCCACCGAGCCGCTGGCCTTCTTCCACAGCCGCATCTTC

TGCCAGCGCGACTCTGTGTTTCGACACCCGTATCTGGTGGAGAAGAAGAACATCTCCCAC

ATGGTCTACCTGTCGTTCGTCTGGCTCACGCTGGTGTACACGTACTTCAGGATCATGTTC

GCGGCCAAGGCGGCCAAATCGGATGCCAGAAAGGCCCGGAACACGATCCTCCTCCACGGC

GTGCAGCTTTTGATGTGCATGTTTACATACATCGGCCCGCTGCTTGAGGGCTTGCTGGTT

TATCTCTTCCCCATGCTTTTGCTCGAGATGCGTTTCACAATCTATGTCATTGTGCACATT

TTGCCCAGACTAGTCAGTCCAGTAGTCTATGGACTGAGGGATCAGACATTTTGCAAGTAC

TTGAAAAACTACTACGTGTGTTGTAATTGTAGTAATTGTATGAGCCAAGCTCCTCAGAAT

GTGACCTCTTTAAAACCTTTGCCACAAGTTAAGCCCCTCTTTTAGAAGTCTCTTATGTTC

TGGTTTGATTCTGTTGTTTTTTTCCATTTCCAATTATATCAGACTGAAATCAAAACAAAA

TATGTAACAATAATCATGTACAGTCATACTGCAGTTCCAGTTCAAACTGCATAATATTGT

GGGTAAATCACCATTCAGTGAATATCTGTACAAAGTCATGGTTGTGTTATACATAATGCC

AGACAAAAAAAAACATGTCAAACATGCAAAAACACTGCTGAAAGCTACGTTTACTGACAT

GTTAATAACCAGCGGTGAGGAATTAATGGCTTACTTACTTAAACATTATTCAGGAAAGAA

AATGTTCACAGTGATCCCGGTCTTTAGAGAGAACTGTCATGTTTACCACTTAATATAGTC

ACACTTAGGAATACCAACTATTTGTAAATTATAATATATATTTGTTTTCTTTTCTCTGTC

TCCTCTCATCTCTATGTCTGCTCTTTGTTCATGTCAAGATTGTTAGTGTGTTTTGCACAG

CAGTATATAACTAGAATGGAACCTGTCTCTGAGCAGATATTCTTAGTAAAAATGGTTTTC

TATCAGAAACTGGCATGGATCTGTTGATTCGCTTATTCTTCCTCTACTCGGTCTTTTATT

GTATTTTATATTTTATATATGTATGTACATTTATTTGGCCAATTAGATTTGTTTTGTATA

ATGATACAGTGATACACCTCAAAGTATCAGACTTGGCCTGTACTGTAACTTCAGATGTGG

AGTCTTTCATTGATTTTCGGCAATGCAAATGCCTAAATATAAAATACAGAATTCTCTTAC

TTTTGCAAAACAACATCCATTTCTCAGTGAGGAGATTTTAATTATGTACATCTTCTTTAA

CAGTTGCCAGCAGTAATGCAAGGCAAATGAAAGCCTAGATAGTTTTTGCAACAATAGTCA

TTTATCCAGTGCTGATTCATATGTAGTATAAGTATGTATAATATGTAACATGTTTAAGTG

AGTTTTCACTGACCAAGAGAG

>CL1061.Contig1_All

TGTTGGTTTCTACCCTAAATTTCTAATAGATCTGCTCTCTGAGGTAGCAGTAATTTCATA

CCTACAATGTTTAACACAAACAATTGTAATTCATTCGTCTGCCATATGTGAAATGACAAC

TTTAACAGCAATGGCTTTTGACAGGTATGTAGCGATATGCAGACCACTACAATACCACAG

TATTCTTACGTCTCAAATGGTTTTGAAGTTATTGTCACTCGTTTGGTTTTACCCTCTGTC

AGTGACACTCACAGTTGTTCTTCTTTCTGTCAGAGTACCCATTTGTGGATCACTTATCAG

TAAACTATTCTGTGACATTCCTTCAATTCTAACGCAAGGTTGTTATCAAACCACAACAAA

CCGCATCTTTGGTATCTTTATAATGACAGGTCAAGTCATACAGGTACTGTTAATTTGTGT

GTCTTATGCTCAGATAGTTCGTGTTTGCTTACGGTCCAGAGAGGGCAGGAG

>CL10717.Contig1_All TAAATATTAAGCTATTCATTCATTCATGGTGACTAGTAATTTTAATGGTGATACAAACTA

ACCAGTTTTAAATATCTGAATTATTGGGGCCACACCCAGGCTAGGTATTTTGGAGCAAAT

TGTGAATATATTCAAAGATTTATTTTCTTTTCCTGCGCAGGATTTTAAACATCTTTTGAA

AACATGAATGTCTCTGATTCAGTCACATTTTTTGTCATAGAGGGACTTCAGGAGAAGAAA

ATGCTCATGTTTAGTATTTTCTTAATTGTGTATATTATGGTCTTATGTGGCAACAGCATG

ATTATATACCTGGTCAGAACAAACCCTAAGCTAAAGTCTCCTATGTATTTCTTCCTTTAT

AACCTGTCTTTCTCAGACATGGTGTATACAACAGTAACTATCCCTAACATGCTGTCAGGT

TTACTGAAGGAGGAACACACTATCTCAAGAAGTGGTTGCTTGTTGCAGATGTACTTTTTC

CTGTCTATGGCTGTGACCGGCCGCTACATCCTGACCGTCATGGCGTATGACCGCTATGTA

GCCGTGTGTAACCCACTACGATATGCTGCCATCATGACTAAGAAGGTGTGCATCCTGCTC

GTGGTGGCGGCATGGTTCTTTGGGTTCGTGACCCTCCTGCCGGCGCTTTCTCTGGCGGTC

CCACTGCCCTTCTGCGGACCCAATCGGGTCCAACACGTCTTCTGCGACCACTCGTCAGTG

GTGAGGCTAGCCTGCGGCAACACCACAGTCAATACTGTCGTGGCTCTCAGTGCTGCCATG

ATTGTCCTCATCGGCACGCTCTGTCTCATCCTGACCTCCTACATCAGCATAGGCAAGGCT

GTGCACGGCATGGGGAGGGCGGAGAAGATCAAGGCGTTCGCCACCTGTGCGTCGCACATG

ATAGTGGTCTGCATCTCATACGTGTCCGCCGCATGTGTGTACATCTCCTATCGTGTGGCC

ACGTTCTCTCCCGACGCACGCATGATCGTGGCAGTGCTGTACTCAGTCCTAACGCCTCTG

CTCAACCCCATCATCTACAGCTTAAGGAACAAGGAGCTTTGGGAGGCCCTCACTAGAGCC

TTATGTAAACATGCTGCAAAACCTACTTCTAACAGGAAAACAATCCCTTCTGTATCCTAA

CAGCGACATAATCTGATACAGGGTTTTTTTTTCTTGACTAGTTTCTTGACAAGTTTTATG

ACCAACTTATTGTTAAAATATATTTTGTAATTATTGTTTAAATGTAATTATCATGAAGCA

ATACATGTAGAACACCGCACAATGTTGACTGAAAATGAGATACTACACTTCTGTGTGTTT

ACAGCTGTCTCTGCCTGTACCTGTCTGTATTAGTTGTTACCTGTAGATGTGGAAATACCA

AGGCAAAAACTTAATGTGCTGGTAACGTGAACCCAGTCAAACTGTTAGAGGGGTAAAACA

TTGACTTAGCAAAAAACTAAATATTGCTACAGACAATACAGTACAAAGTCTTCTTCCTCT

TTCACAGTATTTATTAGTCAGATGTGTTACTAGGCAATTCTTAAATCTACAGAATTTCTA

AAATGCAATGTTAAAGCTTCAGCGCCTAATACATGTGGTCTAATCTATACACCAAGCTGC

AGCTATGAAAGTGATTGTAGATTCACATTTGCTCCCTGCCTTGTGTCTGTGTCATGTATT

ATCGCTACGGTAACGCATTGTGTGCTTTGGTCATGGAACACTACGCAGACCCTCCTCACC

TCACCCCTCTGACTTTGTGGAGAAAATACACACATTTTTACAGACTAATGTAATTCTGTC

CATGAATGTCCATAATTTTGCCAACACAATTTCCAATATATCACCAACTTTATGGGGGGC

AGGAAAACTCCTAAAGTTGTATGTATCTTCCTATTCCACAGGAATGAAATGCTGACACAC

GCTGAAGTTTTTTTTTTTTAAATCATTTGGATGTTACAGTATTATTTTACTGTATTATTT

TATATTATTTTAAATCTCTCATGCAAGGAGTGTAATGCACTGATGCAGCATTCATGTCGT

ATCTTTCTACACAGGGTGCACTCTCACC

>CL11354.Contig2_All

TGACTAAAGCTTCAGATCATAGCTGATAAATCTTAAATATCTCTTTCTCAGGTGGTGGGG

CTGAATATCACCACCTGAGATGGACTACAGACCAGGCAACAACTCCCACAAGGTCTTCTA

CCTGACAGGATTCAGCAGCCTCGGGGAGCACCGGCACCTGCTATCTATTCCTTTCTTCCT

CCTTTTGCTTTACGTGACGACAGCAAACGCTATTGTCACCTTTGTCATCGCAACACAGAA

AAAATTGTATGAACCCATGTATGTGTTAATAGGATCTCTTACACTTTTAAGCTTCTTTTA

CCCCATATTTTTTCTACCAAGGATGGTGATTAGTTTTGCGTCAGGCCGAAATGAGATCAC

TAAAGAGGAATGTCTCATACAGATGTTTCTAATACACTTTGGGGGCAGTTTTCAGAGTAG

TATTCTGCTTCAGATGGCAGTCGATAGATTTTTTGCCATTTGCTGGCCTTTGCGGTACCA

CAACATTGTCAACCTGCGAAACTCCA

>CL11354.Contig3_All CTTTGGGTAGGTTTTACCCAGCTACACCAACATTTTAGGTCTTTTATGGTCTTTGGCTAA

TCAGATAAAGACACTTGACTTTAAACTGTTTCTGCAGACATAATAGAAAGCTTCATCTGA

AATAAGGTAATGCTGTTTAAAAACAGAAACAATTTTGTGTTCATTACTTCTGGTGGTTAT

GAGAACCATAACCAGCATTTACATTTGTTCTTGTGAAATTGTGCTCTGAATTGGAAAATG

CAAAATGGCAGGCAATTCCCAAGTCTTTATTCTTTTTACTTTATTAGTAGACTGTCAGTG

CATATATTGTTGAACTCAGACTCAGATTATGATTTGTTCACTGCAGCTGAGGAAAATCTG

TCAGTCTTCTTTTTCACAACTGTGATATTGTACTAACAGCTGTTTTCCATCCTTCCACGG

TGTCCAAGTGTATTTACTGTATCTGTGCATGAGATGGACTACAGACCAGGCAACAACTCC

CACAAGGTCTTCTACCTGACAGGATTCAGCAGCCTCGGGGAGCACCGGCACCTGCTATCT

ATTCCTTTCTTCCTCCTTTTGCTTTACGTGACGACAGCAAACGCTATTGTCACCTTTGTC

ATCGCAACACAGAAAAAATTGTATGAACCCATGTATGTGTTAATAGGATCTCTTACACTT

TTAAGCTTCTTTTACCCCATATTTTTTCTACCAAGGATGGTGATTAGTTTTGCGTCAGGC

CGAAATGAGATCACTAAAGAGGAATGTCTCATACAGATGTTTCTAATACACTTTGGGGGC

AGTTTTCAGAGTAGTATTCTGCTTCAGATGGCAGTCGATAGATTTTTTGCCATTTGCTGG

CCTTTGCGGTACCACAACATTGTCAACCTGCGAAACTCTGTGTTATTCACTGCTGCACTT

GCCTTTCGCAACACCTTGACTATTGTCGTTAAAGTGGGCTTGTTCATCCCGCTCACGTTT

TGCCACACCAATGCCATCCATCACTGCCTGTGTGAACACTCCTCCGTTGTTAAACTGGCG

TGTGGAAACATAGCACGTAACTACATCGCCATCACTGTCGCTTTCTCCCTCACCACCGGT

GACTGCGTTTTCATCGCTGCCACCTACATCATCATTTTCATAGTCATCTTTAACAGTCCT

TCGGGAGAGTCCCGCCAGAAAGCCATTCACACCTGCAGCACACACTTAGCAGTGCTCTGT

GTTGCGTACCTGAGTGTTTTGTGTGCATTTGTTGGGTACAGAGTGAGCACTATACCCCCT

GATGTAAGGATTCTGTTGAGTTTAGCATATCTCCTCATTCCTAGTTGTTTTAATCCAGTT

ATTTACGGGATTAGGACAAAAGAGATTAAAGTGCATGTGCTTAAACTGTTTAAGTTAGAT

AAAGAGGATTATGGCAAAACCTGAAACGACTGCATGCAGAAGTAGGTAAAGTACTGAGTG

CCAGACTGTCAAATAATCATGAATTTAATTAATGAATTCTGCTGATACTGTTCAAATTCT

ATGCTATTGAGAATTCTATTTGCTATTTAGAAAGCCGTATGTTCAGTGTTTGTTTTCAAT

CTGGGTTTATAAACAAGTACATATAAATCTATTTATGCACTTTTTAACAATTGTTAAATA

ATTGTTGATAGCCTATTTATTTTTTCATTTGGAATTCTATTAGTCTAGACCCCTCTCATA

TTAAAGCAGACAGAAGTGTTTTATAAGTGACAAAAATATAACTTACGATGAGGAATAGCC

ATGCACACAAATGCCTACATGCTGTGTAAACAACATGACTTTGAAAGAGCTCTGTAGGCA

TGGAGACTTCCATTTTCCATTTTTAACAAATGCATCTGAGATGTATTGTAGAGCCCCATT

CGAACGAGTTCTGTAGACCATGAGAAAAATATTTCATTCAGGAACATTTAACCAGTGAAG

GAGTCAGAAGATAAGATATTACCTAATTTTGAGGTTTTAAATAGGTAACAGGTAATAGAA

AGTGTTTTAAGAGATATTTTTATACCATCCTCCTGTCAACCCTCTAAACAAGTGACTTAT

TGAATTACTAGACATGCCCCTGTTTCCAATGTCGGTATTCAAATTATGTATGCTTTTATT

TACGACTTTTATTGTCCAACTGAGCAGACTTGTATCACATACTTCTGTCCATTCATGATG

TATGAAGCTTTCATATGTCTGTATTTTCTGTGAGGTTGAGTGTGTGTGACAGGTGTCTGT

GAGAAAAGATGAACTTCCACATAAATGGACCACAGAGAGTCTAGACACTACTTGTCTATT

CAGACAAAGTTGTCAGTCTAGTTTCAGATTCAGTTTTACTATTGCTTCAGTGCCAAGCTT

TATTATTATCATGAACACATGTAACATTTAGAATTTCCTTATGCACATCTAATTTGGGGT

GTGTTAGACTGATCTTCGTTTTTTGTTACATGTTGCATTACAGCTTAGACTATCCATGTT

AATTTTTAAACTTTGCTTATCTTGCACATTTTTACCTTGTAAAGTTTATTTTAAAATTCA

ACAACACAGCATTGCTGTGGTTTTGGTCATTAACCTATAAAGAAGCAATTAACTACACCA

CATCCTAAAGGTGCATTTGTATTGTGCTGTAGACTACTGCCTTGAGGACCGTTTATCATT

AAAATATACATTTCTGCTTATGGATACAAGACAAATTATTTGTATGTCCATAAAAACACT

TTTAGTAATATTAAT

>CL11354.Contig4_All GTAAGGACCACTGTTGGTACCACTCAGTGTTATAGTGTGTTGATGCGTCATGGTCATTGT

ATACTTATGTTTCATGTACAGTATATGTGCACACTATGTAATAGACCTATGTCTCTTGTT

GACTAGTCGTTTGAGTGAAAAATATGAAATTATGTTCTGTGACTTTAAAATCACTTGTGA

AATTGAAAAATGCAGACCCAAATCATCTGTGATATATTAACACCTATTCTTCGACCACTG

CAAGGTGGTGGGGCTGAATATCACCACCTGAGATGGACTACAGACCAGGCAACAACTCCC

ACAAGGTCTTCTACCTGACAGGATTCAGCAGCCTCGGGGAGCACCGGCACCTGCTATCTA

TTCCTTTCTTCCTCCTTTTGCTTTACGTGACGACAGCAAACGCTATTGTCACCTTTGTCA

TCGCAACACAGAAAAAATTGTATGAACCCATGTATGTGTTAATAGGATCTCTTACACTTT

TAAGCTTCTTTTACCCCATATTTTTTCTACCAAGGATGGTGATTAGTTTTGCGTCAGGCC

GAAATGAGATCACTAAAGAGGAATGTCTCATACAGATGTTTCTAATACACTTTGGGGGCA

GTTTTCAGAGTAGTATTCTGCTTCAGATGGCAGTCGATAGATTTTTTGCCATTTGCTGGC

CTTTGCGGTACCACAACATTGTCAACCTGCGAAACTCTGTGTTATTCACTGCTGCACTTG

CCTTTCGCAACACCTTGACTATTGTCGTTAAAGTGGGCTTGTTCATCCCGCTCACGTTTT

GCCACACCAATGCCATCCATCACTGCCTGTGTGAACACTCCTCCGTTGTTAAACTGGCGT

GTGGAAACATAGCACGTAACTACATCGCCATCACTGTCGCTTTCTCCCTCACCACCGGTG

ACTGCGTTTTCATCGCTGCCACCTACATCATCATTTTCATAGTCATCTTTAACAGTCCTT

CGGGAGAGTCCCGCCAGAAAGCCATTCACACCTGCAGCACACACTTAGCAGTGCTCTGTG

TTGCGTACCTGAGTGTTTTGTGTGCATTTGTTGGGTACAGAGTGAGCACTATACCCCCTG

ATGTAAGGATTCTGTTGAGTTTAGCATATCTCCTCATTCCTAGTTGTTTTAATCCAGTTA

TTTACGGGATTAGGACAAAAGAGATTAAAGTGCATGTGCTTAAACTGTTTAAGTTAGATA

AAGAGGATTATGGCAAAACCTGAAACGACTGCATGCAGAAGTAGGTAAAGTACTGAGTGC

CAGACTGTCAAATAATCATGAATTTAATTAATGAATTCTGCTGATACTGTTCAAATTCTA

TGCTATTGAGAATTCTATTTGCTATTTAGAAAGCCGTATGTTCAGTGTTTGTTTTCAATC

TGGGTTTATAAACAAGTACATATAAATCTATTTATGCACTTTTTAACAATTGTTAAATAA

TTGTTGATAGCCTATTTATTTTTTCATTTGGAATTCTATTAGTCTAGACCCCTCTCATAT

TAAAGCAGACAGAAGTGTTTTATAAGTGACAAAAATATAACTTACGATGAGGAATAGCCA

TGCACACAAATGCCTACATGCTGTGTAAACAACATGACTTTGAAAGAGCTCTGTAGGCAT

GGAGACTTCCATTTTCCATTTTTAACAAATGCATCTGAGATGTATTGTAGAGCCCCATTC

GAACGAGTTCTGTAGACCATGAGAAAAATATTTCATTCAGGAACATTTAACCAGTGAAGG

AGTCAGAAGATAAGATATTACCTAATTTTGAGGTTTTAAATAGGTAACAGGTAATAGAAA

GTGTTTTAAGAGATATTTTTATACCATCCTCCTGTCAACCCTCTAAACAAGTGACTTATT

GAATTACTAGACATGCCCCTGTTTCCAATGTCGGTATTCAAATTATGTATGCTTTTATTT

ACGACTTTTATTGTCCAACTGAGCAGACTTGTATCACATACTTCTGTCCATTCATGATGT

ATGAAGCTTTCATATGTCTGTATTTTCTGTGAGGTTGAGTGTGTGTGACAGGTGTCTGTG

AGAAAAGATGAACTTCCACATAAATGGACCACAGAGAGTCTAGACACTACTTGTCTATTC

AGACAAAGTTGTCAGTCTAGTTTCAGATTCAGTTTTACTATTGCTTCAGTGCCAAGCTTT

ATTATTATCATGAACACATGTAACATTTAGAATTTCCTTATGCACATCTAATTTGGGGTG

TGTTAGACTGATCTTCGTTTTTTGTTACATGTTGCATTACAGCTTAGACTATCCATGTTA

ATTTTTAAACTTTGCTTATCTTGCACATTTTTACCTTGTAAAGTTTATTTTAAAATTCAA

CAACACAGCATTGCTGTGGTTTTGGTCATTAACCTATAAAGAAGCAATTAACTACACCAC

ATCCTAAAGGTGCATTTGTATTGTGCTGTAGACTACTGCCTTGAGGACCGTTTATCATTA

AAATATACATTTCTGCTTATGGATACAAGACAAATTATTTGTATGTCCATAAAAACACTT

TTAGTAATATTAAT

>CL11354.Contig5_All CTTTGGGTAGGTTTTACCCAGCTACACCAACATTTTAGGTCTTTTATGGTCTTTGGCTAA

TCAGATAAAGACACTTGACTTTAAACTGTTTCTGCAGACATAATAGAAAGCTTCATCTGA

AATAAGTGTATTTACTGTATCTGTGCATGAGATGGACTACAGACCAGGCAACAACTCCCA

CAAGGTCTTCTACCTGACAGGATTCAGCAGCCTCGGGGAGCACCGGCACCTGCTATCTAT

TCCTTTCTTCCTCCTTTTGCTTTACGTGACGACAGCAAACGCTATTGTCACCTTTGTCAT

CGCAACACAGAAAAAATTGTATGAACCCATGTATGTGTTAATAGGATCTCTTACACTTTT

AAGCTTCTTTTACCCCATATTTTTTCTACCAAGGATGGTGATTAGTTTTGCGTCAGGCCG

AAATGAGATCACTAAAGAGGAATGTCTCATACAGATGTTTCTAATACACTTTGGGGGCAG

TTTTCAGAGTAGTATTCTGCTTCAGATGGCAGTCGATAGATTTTTTGCCATTTGCTGGCC

TTTGCGGTACCACAACATTGTCAACCTGCGAAACTCTGTGTTATTCACTGCTGCACTTGC

CTTTCGCAACACCTTGACTATTGTCGTTAAAGTGGGCTTGTTCATCCCGCTCACGTTTTG

CCACACCAATGCCATCCATCACTGCCTGTGTGAACACTCCTCCGTTGTTAAACTGGCGTG

TGGAAACATAGCACGTAACTACATCGCCATCACTGTCGCTTTCTCCCTCACCACCGGTGA

CTGCGTTTTCATCGCTGCCACCTACATCATCATTTTCATAGTCATCTTTAACAGTCCTTC

GGGAGAGTCCCGCCAGAAAGCCATTCACACCTGCAGCACACACTTAGCAGTGCTCTGTGT

TGCGTACCTGAGTGTTTTGTGTGCATTTGTTGGGTACAGAGTGAGCACTATACCCCCTGA

TGTAAGGATTCTGTTGAGTTTAGCATATCTCCTCATTCCTAGTTGTTTTAATCCAGTTAT

TTACGGGATTAGGACAAAAGAGATTAAAGTGCATGTGCTTAAACTGTTTAAGTTAGATAA

AGAGGATTATGGCAAAACCTGAAACGACTGCATGCAGAAGTAGGTAAAGTACTGAGTGCC

AGACTGTCAAATAATCATGAATTTAATTAATGAATTCTGCTGATACTGTTCAAATTCTAT

GCTATTGAGAATTCTATTTGCTATTTAGAAAGCCGTATGTTCAGTGTTTGTTTTCAATCT

GGGTTTATAAACAAGTACATATAAATCTATTTATGCACTTTTTAACAATTGTTAAATAAT

TGTTGATAGCCTATTTATTTTTTCATTTGGAATTCTATTAGTCTAGACCCCTCTCATATT

AAAGCAGACAGAAGTGTTTTATAAGTGACAAAAATATAACTTACGATGAGGAATAGCCAT

GCACACAAATGCCTACATGCTGTGTAAACAACATGACTTTGAAAGAGCTCTGTAGGCATG

GAGACTTCCATTTTCCATTTTTAACAAATGCATCTGAGATGTATTGTAGAGCCCCATTCG

AACGAGTTCTGTAGACCATGAGAAAAATATTTCATTCAGGAACATTTAACCAGTGAAGGA

GTCAGAAGATAAGATATTACCTAATTTTGAGGTTTTAAATAGGTAACAGGTAATAGAAAG

TGTTTTAAGAGATATTTTTATACCATCCTCCTGTCAACCCTCTAAACAAGTGACTTATTG

AATTACTAGACATGCCCCTGTTTCCAATGTCGGTATTCAAATTATGTATGCTTTTATTTA

CGACTTTTATTGTCCAACTGAGCAGACTTGTATCACATACTTCTGTCCATTCATGATGTA

TGAAGCTTTCATATGTCTGTATTTTCTGTGAGGTTGAGTGTGTGTGACAGGTGTCTGTGA

GAAAAGATGAACTTCCACATAAATGGACCACAGAGAGTCTAGACACTACTTGTCTATTCA

GACAAAGTTGTCAGTCTAGTTTCAGATTCAGTTTTACTATTGCTTCAGTGCCAAGCTTTA

TTATTATCATGAACACATGTAACATTTAGAATTTCCTTATGCACATCTAATTTGGGGTGT

GTTAGACTGATCTTCGTTTTTTGTTACATGTTGCATTACAGCTTAGACTATCCATGTTAA

TTTTTAAACTTTGCTTATCTTGCACATTTTTACCTTGTAAAGTTTATTTTAAAATTCAAC

AACACAGCATTGCTGTGGTTTTGGTCATTAACCTATAAAGAAGCAATTAACTACACCACA

TCCTAAAGGTGCATTTGTATTGTGCTGTAGACTACTGCCTTGAGGACCGTTTATCATTAA

AATATACATTTCTGCTTATGGATACAAGACAAATTATTTGTATGTCCATAAAAACACTTT

TAGTAATATTAAT

>CL2771.Contig1_All

TTGACATAAGCAACTCAACAACTGCAGCCATTACATACCTTCTATAGATGCTCAGAAGGT

GCTGCAGAAATTATTTGTTTTGATTTGTCAAGAAAGAGTACACATGCCTGCTGTGTGTAT

CATGAAGAATGCATCTTCTTTTACAACCTTCATACTGATTAATTATGAGCCAATGGAACA

ACAGAGATATTTATATATCATCATTTTTTTACTACCATATATGTTAACTATTATTTTAAA

TACTTGCTTAATTTACATTATTTGTAAAGACAGAACCTTACATGAACCAATGTACATTTT

TATTTGCAATTTGTCATTTAATGGAATCTATGGAGGCACAGTTTTGCTGCCACATATTCT

GAGTAAGCTTGCCACAAAGTCATATAAGATGCCTTTAGCAAATTGCCTTATCCAAATATT

TTGTTTACACACTTTTGGTATTATTGAACTTATGATACTTGCTGTCATGGCATATGACAG

ATATGCTGCAATATGTATCCCCTTACACTATCATAACAAAATGTCTCCAAGAAATGTTAA

AATTCTCATTGCGTTCTCTTGGCTTTTTCCTTTATGTGCATTCCCAATGTGGATGTCATG

GACAACTCAATTATCCTTTTGTGGCAATATCATTTACAAAACTCACTGTACAAATTTTGA

TCTAATTAAACTTTCCTGTAATGATACTTCTCTGCAAAACATTGTTGGGATGCTTCTCAT

GGGTGTTTTTGTGTTACCACAGTTAACTGTCATTTTGTTTTCATATGTGCAGATTCTAAG

GATATGTTTACATGGACATAAGGACTCTAAAAAGAAAGCACTTCAGACCTGCATGCCACA

TTTGTTGACCGTCATTAACTATGTTTGTGGCCTTTCTTTTGAACTGATCCAAGTGCGCTT

AAAGACAACGCATACTCAATATGGAATGTCTCTTTTTATGTCTGTTTATTTTTTGATTAT

ACCTCCCCTGCTAAATCCTGTGGTATATGGTTCTACTGTCCTTAAAAAACATATTTATAC

ATTTGTTCAAAGGAAGAAGCTTTCACCAGTATCGTAATCCACATCACTGTCATGTGCACA

AAAGTCAGTGCATTTGTTACCCCTTATTTTCATCCATTGTTAATGTTTCTGTTTTCCTTC

AATACATTTAAATATTAGTTTTAAAACTGCACATGGTCATATTTCTTTGTGGCCTGTGTT

TCATGAAATGTAACTGTGAGTCAAGTTAACATACATAACAGGCCACTAATAACTAAAGTA

AATACATGTAGAATTCCTTTTCCATTTTGCTTAGGATTGATGTCTGGAACTCTTTTTAAA

GATATGTTGGGCAGGAGGTTAGGACTTGTGTTACATTATGGTTTTCAATATACATATCTG

TATTTATAGTTTCACTGAATTATCTTGAAATGTTATGGTTTAGCATTGGTGCCTTATTGC

ACAGGCATTGTAAAGGGCCTACTTGAACTCGTGTTTAAATGAAATCAAATAAAATATATT

GTTT

>CL5918.Contig1_All

CACATGCTCTGCAACGACTCCATCATGATCCTCTTCACTTTCCTCATCGGCATCATCTCC

TATGTCCACAGGCCGACCAAAGCCATGTGCTCGCTCATAATGATCGTCACAACCTCCACT

TCCACCAACGCACCGCTCAACCTGGGTGTCATGTCACTGGAGCGCTACATTGCCATCTGC

TTTCCTCTGCACCACAGAGAACTTGCCACCACTAAGAGAACATATGTGGCAATCGCTGCC

ATTTGGTTCTTTGGGCTGGTCAACCCTGTGACTGACTGGGTTTATAGCTCTATATTTGAC

CCTGATTTTTTCTTTGAACAGGTTGAATGTGGTCATGAGACCATGTTCAGGACCGCACCG

TGGCAGACGCTGTTCTACCAGGCTCTTAATGGCCTGTACTATGTCACTGTGACACTGGTT

ATCCTCTACAGCTACATCAAGGTCGTGCTCGTGGCCCGGTCTGTGTCCAGTGACCAGAAG

TCAGCTGGGAAGGCACACAGGACTCTTCTGCTACACTTCATCCAACTGCTGCTCTGCCTG

AACACGCTTCTGTATGGCCATATTATCGGCTTCATGGCTGTGATGCTGAGCTATGAGGTT

TACTATGATGTGCGATACACAATCTATCTGCTCGTGATTCTGCTGCCCAGGTGTCTCAGT

CCCGTGATCTACGGCCTGAGAGACGAGGCTCTCCGCTGTGTGTTTATGTACTATTTCAAA

TGTGCCCTGTCTAAAGTCAAGCCTAGTGTAAATATGCACTGACTACATTTATGTTCCTGC

ATTAAAAGGCAAAAGGCTGCAGTGTATTCCAGACTTACTACAACTTGCAGAGGTGTGTGT

GTGTGTGTG

>CL6972.Contig1_All

GCCAACCACAAGGTGATCACACACACAGGGTCTTTATGAACCAGCAGCTCTGCTGTGATC

TGCCACTGGTCCGACAAGGTAAGCTGCCCACCGCAAAATTCCTTTCATATTTCATAATTC

CACATCCCATGTCTACACATTTGCACATTTTCATATGGCATGCTGTTTTAAAATGCTTGT

TTTATGACCTGATGTTGCTGTTAATTGCAGTGCAGCTCTAGAGGCACTGGGTGATGAATC

TCACTGTCAGAGATGACTTTCAGGAGGCTTTGGTGAAAAATCTAGTTATTGTCGCCCTGG

CTATTGTCATCAACTGCATCAATGGGATCATAGTCATGACCTTCTGCAAGAACTCTGTTT

TTCACAGTAATTCAAGATATATTCTGTACATGAATCTCGTAGTGAACGACATGATCATGA

TCTACATTTCAGTGATAATGTATGTTTTGAGCTATGCTCACCCGTTTTTTAAAGCCTCAA

TGTGTTGTATTTTGGTTGTTGTCAGTTCCACTACGTACATGAACACTCCTATTATCCTGG

CCGGCATGGCTATTGAGCGCTACATTGCCATCTGCAAACCTCTCCATCACGCTCAGATCT

GCACGGTGCGCAGGACCTACGTACTCATCGGCCTGACCTGGGGTGTGGGCCTAATACCAG

CACTAGTGGACGTTGTTATTGTCCTTGCCACCAGGTCAGCAGGCTTCTTCTCCACAGTAG

TCTTTTGTTACTACCTCAGCCTCTACAACACCCGATACCATGAAGAGAAAGCGAGAGTGG

TCCAAGCCATGTATATGTTATTTGTGTGGCTGACACTGATCTACACTTACCTGAGGATAT

TCCTCACTGCTAAAGCAGCAACAGGTGACACTGCTTCAGCCAAAAAGGCCCAGAACACTA

TCTTACTGCACGGCGTGCAGCTCCTTCTGTGCATGCTGTCCTATATCACTCCCTTAATTG

ACTTAGCCTTAATTACATTTTTCCCAATGCATCGTTCAAAAATAACATTTCTGGGCTTCC

TGATCACAAACATTATTCCAAGGCTGCTAAGTCCTCTCATATATAGTATTAGAGATCAGA

AATTTGCGAAACACATGTCACAGTACTACTCCTGCTGTAGGGACATCAATTCAAAAAGGA

ACCGGAAAAGGAGAAAGCTGACCTTCTCTAGCAAGAGAGTTGGTTCGTTAAATTAATTTA

AATTATGTCATGAAATCTGTATCATAAGAGATATATGTATCGAGGTGTACTCTAATCTTG

TTGCTTCTCGTCATCCTTAACTCATACTTGCTATTATTATTTTTAAATGACAGCAGATGG

TTCAAACCCTGTCAGTTCCAGTAAAATATATTTTGTCATATACAATATATTTGTTTATAT

ATTATATAAGATATATATATATACTTTTT

>CL6972.Contig2_All

GCCAACCACAAGGTGATCACACACACAGGGTCTTTATGAACCAGCAGCTCTGCTGTGATC

TGCCACTGGTCCGACAAGTGCAGCTCTAGAGGCACTGGGTGATGAATCTCACTGTCAGAG

ATGACTTTCAGGAGGCTTTGGTGAAAAATCTAGTTATTGTCGCCCTGGCTATTGTCATCA

ACTGCATCAATGGGATCATAGTCATGACCTTCTGCAAGAACTCTGTTTTTCACAGTAATT

CAAGATATATTCTGTACATGAATCTCGTAGTGAACGACATGATCATGATCTACATTTCAG

TGATAATGTATGTTTTGAGCTATGCTCACCCGTTTTTTAAAGCCTCAATGTGTTGTATTT

TGGTTGTTGTCAGTTCCACTACGTACATGAACACTCCTATTATCCTGGCCGGCATGGCTA

TTGAGCGCTACATTGCCATCTGCAAACCTCTCCATCACGCTCAGATCTGCACGGTGCGCA

GGACCTACGTACTCATCGGCCTGACCTGGGGTGTGGGCCTAATACCAGCACTAGTGGACG

TTGTTATTGTCCTTGCCACCAGGTCAGCAGGCTTCTTCTCCACAGTAGTCTTTTGTTACT

ACCTCAGCCTCTACAACACCCGATACCATGAAGAGAAAGCGAGAGTGGTCCAAGCCATGT

ATATGTTATTTGTGTGGCTGACACTGATCTACACTTACCTGAGGATATTCCTCACTGCTA

AAGCAGCAACAGGTGACACTGCTTCAGCCAAAAAGGCCCAGAACACTATCTTACTGCACG

GCGTGCAGCTCCTTCTGTGCATGCTGTCCTATATCACTCCCTTAATTGACTTAGCCTTAA

TTACATTTTTCCCAATGCATCGTTCAAAAATAACATTTCTGGGCTTCCTGATCACAAACA

TTATTCCAAGGCTGCTAAGTCCTCTCATATATAGTATTAGAGATCAGAAATTTGCGAAAC

ACATGTCACAGTACTACTCCTGCTGTAGGGACATCAATTCAAAAAGGAACCGGAAAAGGA

GAAAGCTGACCTTCTCTAGCAAGAGAGTTGGTTCGTTAAATTAATTTAAATTATGTCATG

AAATCTGTATCATAAGAGATATATGTATCGAGGTGTACTCTAATCTTGTTGCTTCTCGTC

ATCCTTAACTCATACTTGCTATTATTATTTTTAAATGACAGCAGATGGTTCAAACCCTGT

CAGTTCCAGTAAAATATATTTTGTCATATACAATATATTTGTTTATATATTATATAAGAT

ATATATATATACTTTTT

>CL7175.Contig1_All

CTGGAAGGCTTGTGTCTTGGCAGGCTTAGAAAGCCTTGCCATTATACTTAATGGAGACAT

AGTTTATCTGTGCACCTGTATCCACAGGGACAGAAAAATATATTTAAGGTTTTGCTTGTT

TATTTTACAGAGTTATTGTTGCTGTTGTTTACTTTTCTTGTGATTTGAATATGATGTTTT

TTAACACTAATTCATAGTAAACTGTTTTCACTAGTTTACTGCTCTTCATCAGACAATGCC

TGCACTCAATGTGACCATTAACACATGTGTTATCTCTTACAATCTGTGACATGTATGCAT

TTAGTGTGATGATGAACACATGTGTGATCTCTTACAATCTGTGACACGTATGCATTTAGT

GTGATGATTAACAAGTGTGTTATCTCTTTCAATCTGTGACATGTACAGTATGCATTTTTT

GGACGTGTATTTATATGCGCAGATGCTGTACATTGTTTCTATAATTTTATTGTAAATATT

TTAAAATGTTTCTGTTTTAATGTTGTCAGATTGTTCTGTTTTTACCTGTAGCTAGTAGTA

GTAGTTAGTGATAAAGTGGTTTCAGACATGCTGGATCTCAGAGCAGCTCTGTCAAATGGC

ACCCTCATCCACCCTCCAGGCTTTTACATTGTTGGACTAAGCAGCATGGCCCATGCTAAC

GTCTACCTTATTTTCCTAGGTGTGGTTTATGTCCTCACCATTGTGTTTAACGGCTTTCTC

CTGTCTCTTATCTGGCTCAATCATAAGTTACACACTCCCAAGTTTCTGGCTGTGGCCAAT

CTGGCCGTGGTGGACACCTTAATCAGTTCCTGTATCATCCCCAGCATGCTAAAGTTCTTT

TTGTTTAGAGATTCATTTGTGCAATTTGATCTGTGCATCGTTCAGATGGGGGTCTACTAC

TGCTGTACGTCTCTCGAGTCCTTTTCTCTGGCTGTGCTGGCTTACGACAGGCTCATCGCG

ATCTGCTTCCCTCTGAGACAGCACACTATCAACACAAACACCAACATGCTGTGCATTCTG

GGAAGCATCTGGGCTCTGCTAATTGCATGCTTGCTGTTTGCTTGTTTGATAATGACAAGA

CTGTCTTTCTGTGACTCTGTTGAAGTGTTCAGCTTCTTCTGTGACTACACTCCTGTCTAC

AAACTGGCATGCAACGATTTTTCTCTGCAGTGGTCCACCGCTGTCAGTCTCAGTCTTACC

ATCATACTCGGTCCCCTCACCTTCATCATCATATCCTATGCCAGCATCCTAATTGCTGTT

TTCAAAATTAAGATTGCAGGGAACCGGTACAAAGCGCTGGCTACCTGCTCCGAGCATCTC

ATTCTCGTCGCCATTTTCTTCATTCCAAAACTGTCTTTGTATGTCCTTGGTTTCCTCTTT

TACCGGCTGGACATGGACATAAGGCTAGTGACTTTGTCCATGTCTACCTGCATGCCCCCC

TGCCTGAACCCTGTTGTGTACGCCCTGAAGACCAAGGAGATTCGCAGTAAAGCACAGATG

TTGTTTTGTAAGACCAAAGTGAGACAATGGGGGAAAATAGTAGCATTTCATAGCAAACAG

ATGGAATCAGTGCACACATAGGCTAGTAACCTCTATGTTTACTTATGTTGTTGTACAAAT

AAATAAGGAATTTATAACTAACTTTCCACACATAAATTGTCATGTATTAAACCTTACCTA

AGATAATAAATTGGCTGTGCCTCAATAATAAGAAGATGTAAATATAAGCTATTCCTTTTT

CCACATTGAAAAACCAAGTTATCCAAGAAAAAACGGAAGCCAACTGCCTTAAGAGTAAAG

TACTGTGCTTTGTGAAAAGGGTAATCAACTTAAAATGTTTTCCACTGTTTGTACAATAAA

AATAAATGAACATTTCAGGCAAAAAAAA

>Unigene109144_All

GTCTATGTCAGGTGTTTATAATTAATGTTTATGCGACTGGTACATACTCTATTCTCACTG

TAATGGCCTACGATCGCTATGTCTCCATAATCTACCCGTTACAATACCATGCGATAATGA

CGCCACAGAAAGTCAAACGGTTATTGGCGGTAAGCAACTTTGTTCCTGTAAGCTCAGCAT

TTGGTCAGGTATGGGTGACATCGCAAGTGCCTTTATGTAGTAGCACTATTCCTAAACTGT

ACTGTGAAAATATATCTGTGTCTAAGCTATCATGTGCATCTAGTAAATTGTATCGTGTGA

GTACGCTGTATGGTGTTTCTGTGTTTGTCATTTCAGTTGTTTTTATTGTGT

>Unigene1237_All

ACCTATTTGTCATATCAGTGTGTATACAGAGGAGAGTCATTCATTGGCCTACCTCTTGCA

AAGAGCAGTGGGAGATGCACATAACTCTAACTTAAAACTCAACCAGATTTAGTTATCAAT

CTAGGTCAAGTTAACAAATGCAGTTAAAATCTTCCTTAAATGTTTATGGATACTTTACAT

GTAAATATGACTATTATAAAACTTTGTGCTGTATTACTCTGACAGGCCTGGAAGATCTTA

TTGTGTTTTACATGCAGAGCTTTGTAAAAGCTGTTGAAGATGTCAAATGTCACTTTTACA

TTAGAAGCCTATCGCAGAATTCATGATCAAAGATTTATTTTTGCTGCTGTGTTGACCTTA

TTGTATCCTGTTATCATATTTGGTAACTTGTTAATTATATATGTTGTAAGTGTTGAGAGA

ACTTTACATAAGCCTATGTATATCCTCATTTGTAATTTGGCATGCATTAACTTGTATGGT

GGTTCCAGTCTTGCACCTTTTATTGTAGCGAATATTCTTTCAGGAACATTTCAGATATCC

TGGGTTGCTTGTTTTGTTCAAGTATTTTCCATTAACACATATGGAGGATGCGAAATGATG

AATTTAATGATGATGGCTTTTGATCGATACATTTCTATATGCTTTCCTCTGAACTACAGA

CAAATAATGTCCCCTTTAATTGTTATCATATGTGTCATTTTTATTTGGCTTATTCCTTTT

GGTCGGGTAACCATTACCCTCTCAATCACGGCAAGTCTAAAAATTTGTGGAAACATAATT

GAAAAGGTTTATTGTGACAACTACTCTGTAGTGAAACTTGCATGTTCAGAATCTTCAGCC

GTCAACATATACAGTGCAACTGTAACATTCATTTATGTCCTCTTGCCATTTTTTGTTATC

ATTTATTCATACATAAGGATTATTTTAATATGTGTTCAGTTGACAAGAAAAGGCCAAACA

AAAGTGATGAATACCTGCACGCCACACCTGGTATCGATATCCACCTTTTTTATCGGATGT

GCTTTTGAACTGTATCAGAGTAGATTTGACATGAGTCATGTTCCATATGCCGGTCGAGTC

GTTTTATCTCTCTATTTTCTAATCTTTTCCCCTGTATTAAATCCTGTGATTTATGGAGCC

CGGACAGAAAAGATAAATGAGGCAATTAAGATGAAAATACAGAATTTCTTTACCAAGCAA

AATATCACTGTGTAAGCTAGTATATGTATACATTGGTGATTATTTGCAAATGTACAGTTG

GTTAAATAAACCTTGACTTTATTAACTGGATCAAAGATAAACATGAGTGTGTAATCTTGT

ACATGGAATTCAATACGATCAGGTTCCCTCCTCTATTCTGTTTTATTGATCGATGATAGT

ATATTTTCTTTGAGAAGGTAAATACGAAAACCACACTTTGTATTATGATCTATTATTCAT

TTTTCCACAGAAGCTATTAACACTCAGCCTATGTTTTAGCCATCAATTAGGTCAGGACTA

CTAAAAAAAATCTATACTGACCAAGAGGGACTTACACACATGTAGAGGGATGACTAGTAG

CTCATTGTAGGGAGGATGGCATATCTACTTTAGAACATCTAGCAAGCTGCCTTGCTCTCA

TTTTATGATAGGAGATGTTGTTGCTGTTAACCCTATGGGCCCGAGGCTATTTTCAGGGTA

TTTTTA

>Unigene22006_All

AGCTCAACTCAATGCTGTTACTAATGGTGTGGCCAAACCACTCTTACAGGGAACATCCAT

ACGCTACTTTCAGTAGAGATTATTAGTGGCATGTCATGGCTCTAACACTCAACAGCACAA

AGGAGGAGCTCCTTCAACAGCTGATACTTCACCACTCGGCAATCGCTCGATCGCGGTCCA

AATTCAGTGTGATGATGATCGTGTCTTTTGTTTTCATCTATATTAACTCAATCATGTTCT

TCACTCTGCTCAGCAAGCCTGTGTTCAGGGACACGCCACGCTACGTCCTCTTCGCACACA

TGCTCTGCA

>Unigene23721_All

AAAGGGACAATTCAGCTTTCGGTACCTGCAATGAGCTAACAACATACTTCAAACACTTTT

GTCTCTGTATTTTATCATTTTTAACAATGACAAACTCAACTAAATTTTTGACTGTTATCC

TTGCAGGGTACATTGATACTGGTAATATTGCATACCTTTATTTTACCATATTATTACTGT

TTTATTTTTGTATAATATTTGCCAATGTTCTACTAATCACAGTTATTTGTATAAACAGGG

CTCTGCATGAGCCTATGTACTTGTTTGTCTGCAGCCTGTGTGTGAATGAGTTGTACGGCA

GCACCGGTCTGTTCCCTTGCCTTCTGGCTAATGTGATCTCAGACACACATGAAATCCCTC

TTGCATATTGCTACATGCAAATGTACTGTTTGTATACCTATGCAACTGCTGAGTTTTGTA

ATTTGTCTGTGATGTCTTACGACAGATACATGTCTATCTGTTATCCATTACAATACAGCA

GCATCATGACACAGAGGAAAGTGTGTATTTTACTTATGCTTGCATGGTCCTTTTCCTTCA

GCCAGTTTACCATCACCCTCTCCTTAAATCTGCAGTTGGAACTGTGTGGTAATGTCATAC

AGAAGGTGTGGTGCGACAACTTCTTGCTGGTGCGACAGGCCTGCTCAGATACTACAATGA

ATAACATATTTGGACTTTATGCGACTGCCCTGTGTGTAGGGCTTCCGTCCCTACTCATAC

TCTACTCGTACATCAGAATAATCATAATTACTTTCAGATCCAGTTATGAAGCGAAGAAAA

AGTGTCTCAATACTTGTGCTCCTCATGTCATCTCTGTGATTAATTATACCATTGGTTGTG

CTTTTGAAATGATTCAGGGTAGGCTAGGTAAAGCAAATATGCCAGATATGCTGCGTGTCT

TTATTTCATTATACTTTATAATAATTCCAGCACTTTTTAATCCAATTATGTATGGACTAA

AGATGTCTAAAATCAGGGAAGCGTGTGTAAAGTTAAAGAAAAAAATCATTTTTTAAGAAT

GGAAAAGACAACGTTTTTTTTTTTTAAGAGGAATGTGTTTTCAAACGTATAAAGTATAAT

GTGGTGTATCCTATTTGTAATTTGTGCTTCTTGGATTTTCAGCACTAGGAAATAAAAATG

GCATAT

>Unigene25551_All

GGCAGGCACTGTGGCATGCTGCTGTTTTGTGGCATCCAGCAAGGACTTACTGCCAGCTCA

ATGTGATGTGTTATTGTCTTTACCCTTTATATCACTGTTGTTCTGGATTTGCTCCAGTTT

TGCACCAGTTAACATGTCTCCTACTGAAATGGAAAATGCTACAGGGACGTTTATGTTTGT

CCTTATGGGGATCAATGAAACAGCTACTAACAAACACATCTACTTTGCCTTTGTACTGAC

TGCTTACCTCTTTACTGTGTTTGTTAATACCACTTTGATCATAACCATTGTCTTGGAAAA

GGCTCTGTACGAGCCGATGTATTTCTTTGTGTGTAACCTCTGTATCAATGCAATATGGGG

CGCAAACAGTTTTTATCCAAGGTTCCTTTTCGATCTGTCCAGCCAGGTGAATTTGATTTC

ATACTACGAGTGTTTGATCCAGATCTGTTTTATATTTTACTATGTGTTCTGTGAGTACAC

GTCGTTGGCGGTGATGGCTTACGACAGGTTTGTGGCGGTGTGCAGGCCACTGCAGTACCA

CTCCCTGATGACCCCCAGACGCGTGATGTACCTGCTGTTGTTTACCTGGGGGTTCACCCT

GACGGAGGTCGCGATCGGAGCCGTGCTGACGGTCCGGCTGCCCATGTGTGGGACGCACCT

GCACAAGATATACTGCTCCAACTGGCCCATAGTCAGGCTGTCGTGCGTGGACTTTACCGG

GAACAACATATATTCATATGTTCTGATAATAATTCATGTGTCTGAAGCTTTGCTTATCAT

TGTGTCGTATGTTCACATCATCAGGACATCGCTGCAGTCTAAGTCGGGCTGGGCTAAGTT

TATGCAGACATGCCTACCACATCTGATTACTCTGATCAACTTTAATGTGGCGCTGCTTCT

TGATGTGTTTGATGCGAGATATGGTTTATATCAGAAATCACAAGCCATTAGAAACTTCAT

GGCCATAGAATTTCTCATTGTTCCCCCATTGATAAATCCTCTCATATATGGAATATCTCT

TAAAAAAATCCGTACTAGAATAATGAAATTTTACAAACATCAAATTAAGCCTATAAGTTG

ACTATGGTTAACTGACTATTTAAGACAAGTGTACTGATTTATATTGTTGAAGGGGTTAAT

ATGTGTATTTATTAGTTATAACCCAATGTAAATGTGAATTGACATCAACACTTGTCGTGA

CCTATCATGATGCTGTTTGCAAACTTGTGATGACACTGTTTAGAAAGTTACCATGTGTGA

ATGTGGTGATATCACAGTGTATTAACAAGATATGTGAAATGATGACATAGTATTATTCTT

ATTTCAAAAAGAGATTATTTAAAATCAATACTCCAGTTTAAAATAAAAGTAAAAGTGACA

GTGGCATATTCAGTTTATCACAAGCGATAATATATCTGCATATGTTATAGTAAGGGAATG

AACTTTGGTCCTGGACCATAAAATCTAGTGACATTTTTGGGAGAAGTATTTTTTATTTAA

ACTGTCATGTATATTAACACAAATGATAGTTGTGGTAAGTGAGCAAAATATCATGGAGTA

AATCTGAAAGCACTCAGAAGATATAGAAGGTATAAACATAAAAGCTTTGAAACATATAAC

CTTAGGATCTGTACGGAGACATCTCAAAAATCACAACATCTCAGGTGACCTCATGCTGAC

TGCTCAGCTGAATTGGAGTAAAACTCAATATGACCTTTTTGGAGTCAAGCTGATGATAAA

AACGCAAACTTTCTAGACTGAAGTGTTGTTGTAGTTGACTTGGATTGGTGCTGGATCAAA

TGGAAAGGGAATATTTCAGCTTTCTCTTGGTTATTGCTGTATTACGGCAGAAAGAAAAAC

ATGTACGACTTCTATGCCTTTAGTAAAGAAATAAAATATAAATTCTGAAAAAAAA

>Unigene28245_All

CTGACACCGATATCTCATCTTGATACACTTGCTGATACAAAATCTTGTGTTGTCACATGA

CTTAACAAAGTTCTGTACTTGTAAACTACTAATTATTTTGTCACTTAGTATGTTGTGGTA

TTTATAACTTTTATCACTTGTATTTCATTATCTTCCTTCCTAGGCAAGAAAACATGTCAA

ATATGTCGATTATTTTGATTGCTTACAAGGGAATATATGACACAAGATTCATATTTGTTA

TAATGCTGACTTTCCTGTATCCTATTATGATCATTTCTAATGCTGCATTGATATTTATAA

TATGTAGTGAGAAGAGTTTGCACAAGCCTTTGTATATACTGATTTGTAATTTAGCATGCA

TAAACTTGTATGGGGGCTGTGGGTTAACCCCATTCGTGGTATTTAGGATCCTTTCAGGTA

ATTTTCAAATATCAAAAAGTGCTTGTCTTTGTCAGGTGTTTTCTGTTATGACGTTTGGGG

GTTGTGAGATAATGAACCTCATGGTTATGGCATACGACAGATATGTTTCTATATGCCTCC

CTCTTCATTACGAAAAAAATTATGTCTGCCTCAACACTCATGAAACTTATTGCCTTAGTT

TGGCTAGTCCCATTTATCAGGACTGGTATTGCTGTACTAATAAATGCAAATCTGGAAATG

TGTGGAAGGATTATTGAAAAGGTTTATTGTGATACCTATTCTGTGGTTAAATTAGCATGT

ACTGGCATCAGGGAGGTCAATATATATGGGTCAGTCATGACATTCTTTTCTGTGATCCCC

CCACTAATGATCATAATTTACTCATATGTAAAAATTCTAATGATTTGTACAAAACTAAAG

CAAAGTGGACAAGCTAAAGCACTGAGCACTTGCATTCCACACCTTGTAGCAATTGTTAAC

TTTTTTGTTGGCTGCACATTTGAAGTGTATCAAGGTAGATTTGATATGAACTACGTTCCA

TATCCTCTGCGTGTAGTGCTGTCATTGTATTTTTTTATTCTTTCACCTGTTCTCAATCCG

ATAATCTACGGAGTTAGGACACAAAAGATAAAAGACAGCCTGATTAAAAAAGTAATGCAT

GTATTCACAAAGGTACAATCCAGAAATAAAACATTTGTGTCAATAATGTGAATTGCTGAA

TTACACATCGACCTCTTTTTGTAAAGTTCTCTCTTTTTTTCTTCTTTTGTCTATGCAATA

GACATAGGTCTAAACTGAGGGTTGTTCACAATTCATCTTTATAGAACAACTGATCGCTAG

AAGGAATTTTTAATAAATATGCATTTCATCAATATTGTATTATTGTTAG

>Unigene34891_All

GTTGATTGGGAGCACAGCTGTTTGGCCGTACGTCATGGTCATCCTGTTGACAAACCTGAG

TATTATATCATACGAGGGCTGTTTCGTTCAGTTTTTTCTCCTAGGAAGCTATGGCAGTTG

TAATTTTACAATTTTAACAGCAATGGCTTATGACAGGCTTGTCTCCATATTTAAGCCTTT

GCAGTACCAAACCATCATGACCCCACAAAGAGTAAAACTGCTGTTGTTTATGGCCAGTTT

CATTCCAACAACTTTGCTAATCGGTCAGATAATCCA

>Unigene62103_All

TAATTGGCCTCAGCTGGAAGTAGTTCTCTTTGTGGTTATCTTGATCTTCTACCTGATGAC

ACTGACAGGAAACCTGTTCATCATCATCCTGTCATACGTGGACTCCCATCTCCACACACC

AATGTACTTCTTCCTTTCAAACCTCTCATTTCTGGATCTCTGCTACACCACCAGCTCTAT

CCCTCAGTTGCTGGTGAATCTC

>Unigene64046_All

CCTGGCTGGCACTGACTGAATACAGATTTTCACTGGATCAAAAACAGCTGTTTTTTCAAT

GTATTGCCGTTTGTTTAACTAGATTGTTTAACAGTATACCTGCTGGAACAGCACCACTTA

ACTGCTGCATTTAGTTATCCGTTTTTAGTTGGTGGGCAAAAAAAGAATATATTATGGCGG

ACAATGGAACTGTGAATGTTTTTGAGTCAGTTTTTACAATGGGTAGCTTTGACTTAAACC

CAGAAGCAGTCTATGTGACTCTTGGCCTTGGAATTGTCATTTATCTTTTTGCACTGTGCT

CCAACTTGCTTTTGCTTGGAATTATAGCAGTCCACATACACCTGCATCAGCCAATGTACA

TCTTACTGTTCAGTCTGTCTTTTAATGACATTATCGGTGCAACTGCCATCATTCCACCAG

TTCTCTACAACCTGATGCTAGAGGACAGGCAAACAAGTTTCCTGGCATGTCTGTTTCAGG

CCTTCGCTTTGCACTGGTATGGTGGGGCATCTTACCTCATTTTATCCGCAATGGCTCTTG

ACAGATACATAGCCATCTGTCACCCTCTTAGATATCACGCAATCATGTCCAAAAATGCTG

TGACATACCTCATAATGTTGTGCTGCATCATTAATGTCCTCCTGGTGGGATCTGTGTTTG

GGCTCACCCTGCGTTTC

>Unigene67013_All

ATGATCTTCACCTTCCTGAAGAAGGAGGCCTTCAGGACAGACACGCGCTACGTCCTCTTT

GCCCAGACACTCTTCATGGACTCGTTCCTAATGATCCTGACAGACTTGGCACTCATTGGA

AACTACTTCCAGTTCCCTATTCCCTGGATTCCATGCTGTGTATTTTGTTTTCTGATGAGT

TGGCTGAGCCTGGTCACCCCTTTGACACTTGCTGCCATGTGTCTGGAGCGCTACG

>Unigene78523_All

GAGATATTTCTTTAGAAAGAACAAAGTCATCGACGGGTTTTTTCATGTTAAGGACTATGG

AAAACTCATCTTTTCACAAGGTTTTTATTTTAACTGGACTTCAACAATCGGGAAGAAACA

AGTCTCTGTACTTTTACTTGACAAGTGTACTGTATATTCTCATCATTGCCATGAATGTAA

CAATTATTATCACAGTCAGGGTAGATAAAGCCCT

>Unigene82069_All

CTCTTAAGCACGCAGATATTACCTACTGCTGGCGAAGCTCAGTGTGATGAATTTCACAAG

AGATGACGTTGAGGAGGCTCTGGCGAAAAACCTTACCATCGTTGCTCTGGGCGTTGCTAT

CAACTGTATCAATGGGATGATAGTGTTCACTTACCTAAATAATGCTGTTTTTCACAATGA

CACACGATACATCCTGTACATACATCTTGTGATAAATGATATGATCATGATAACCCTGTC

TGTATCACTGTACGTTTTGACATATGCCTACCCAATCTATAATGTCTCTGTCTGCTGCAT

GTTGATTGTGATCGGTTCAGCGACTCACAAGAACACGCCACTTATCCTGGCCGGTATGGC

TATTGAGCGATACATTGCCATCTGCAGACCTCTCCATCATGCTCAGATCTGCACGGTACA

AAGGACCTATATTCTCATCAGCATCATTTGGGTTGTCGGCTTCATCCCTCCACTGGCTGA

TGTGATCATCCTCCTGATTGTCAAACCGTGGAGTTTTTTTTTCACCAACATCTTTTGTTA

TCCCTACACTTTGTACGGTTCCAAATATTACGATGAGAAGACAAAAGTGGTCCAAGCTCT

CTACCTGTCATTTGTGTGGTTAATACTGATATTCACTTACTTTAGGGTACTTGCTGCTGC

TAAAAAGGCTAAAGGAGATTCTCCTGCTAGCAAAGCCCACAACACCATTTTGCTTCATGG

AGTACAACTTCTTCTGTGTATGCTGTCTTACATCAGTCCTTTGCTCGACATCATTTTAGT

TCCGTTCTTTCCTGCCCATCGCTCAAAGATATCATTCTTCAGCTATCTAATTACAAACAT

AGTTCCGAGGCTGTTAAGCACCCTGATCTACGGAATCAGGGACAAGAACTTCGCCAAACA

TATGTCGGTCTATTGTTCTTGTAAGATGATCATTGTTAAAGTGAAGCCAAAAGTGCTTGT

TAATAAAGAGCAACTATGATGTAA

>Unigene86653_All

CAAAAACCCTTTGGCAATCACAGACACCAACCTATTTCAGCATAATGCTCATGACATGTG

ACGTGTTTATTTGCAAAGGCTGCTGACCTTTTTCCAAGTCAGGTATCGTAAGTCTTAGAC

CCTGCAACCATGAACACCACAATCATTTTGGATGACTTTAAAGATGCTCTTTCCAAAAAC

CTGACTGTGGTGCTGTGTGGCCTTTTCATCCTCTATGTCAATGGAGCTCTAATGCGAGTC

TACTTCACCGTGCCCTCTTTCACCCAGGAGGCACGCTACGTACTCTTCATCTTTCTGGTG

TTCAACGACATGATGATGGTGGCGCTCACTGTTGTGTTACACGTGCTGACCTACAAGCCT

CGTTTCATCAGTGTGCCTGTCTGCTACATTCTGGTGAGCACTAGTGACACCGCCTACCGA

A

>Unigene9188_All

GAGAGAGAGAGAGAGAGATGGATAGAGAGATATACAATGTATATTTGATTTGATTAGACT

TCACTAATTATCATTTGTGAAACAGAATAAAAATATGTTAGCCGTATGATTATGACATTG

TTATGACTGTATATATTTGAAGTTATAAATATTTAATCCGATCCTGTTGAAACTATTTGA

ATGTCCTGCTCCAGCCCTGCTCCAGTAAAAGACTCATACAAGGGCTGCCCTTGCATTGCA

GGTAGTCATGATGTCTTCTAATGTGTCTGTCTATCCATTCTTCTCATCTGCTTTGACGCT

GGAGTCACTGAAACTTTCACCACCAAGAAATTATCCAGCTTTTATCTTTGGGACACTGGT

CTATTTAGTCATAGTCTTTTTCAACACAGTGGTTCTCTCTGTCATCGTGCGAAGTAAAGA

TTTGCATAAGCCTATGTATATTATATTATTCAACATGCCACTGAATGATCTTATGGGTGC

AACAGCTTTTTTTCCCACAAATGGTCTTCAGTCTTGTGACCCAGAATATTTCCTTATCTC

ATATTGCATGCATCCTGCAAGGGGTTCTCATACACCTGTATGGAGCCGGAGCCCTTCTCA

TTTTAACTGCCATGGCTTATGATAGGTACGTTGCCATTTGCAGGCCTTTGAGGTACAACT

CCATTTTTACTAACGGTTACCTGATTAAACTGATCCTCCTTATGTGGGCTCTAGACCTGA

GCCTGATCCTAATTCTCTTCACTCTACTTACCCGACCTCAACTTTGCGGTACACAGATAC

AGGACCCCTTCTGTAACAACGCCGCTTTGGTTCGGTTGACGTGTGGTGACACACGGGTGA

ATAACTACTATGGTCTGTTTATAACAGCGTTTTTCCAAAGCCTGTCTCTTTCTGTCGTGA

TTTACACTTACTTGCAGATCCTGAGAGCCTGCATGTCTGGAAACAAAACAAACGCGAGGA

GCAAGGCGCTCCATACCTGTGCCACACACTTGGTGGTCTTCCTGATTTTTGAGGGCACGA

TTTTGTTCAATGTACTTTCATATCGCATGCAAGGTGTGTCGTCGAGTCTGAGCAAGTTTT

TTGCAGTGATGATGCTTGCCTTTCCACCTCTTGTCAACCCCTTGATTTATGGACTAAAGA

CTAAAGAAATTAAACAGAAAATTGTTGTCTTCTGTAAATAGATCTCATGTACAGTTATTA

AAGAACACTGAGTGTTTTGTATAGGGAATTTTAAATATTTGTATTTATCTGTATTTTGAT

TTATTTTTGTTTTATTTAGTATTCCAGTATAGAAATATATATGTTTTCAATAACAACAGA

AATCAAATAAATGTTTATCAGAACATGAAGTCTTGCTTTACAGAACAGGGAAATGGTATG

CATCATCAGTATTACAAAGTGTTACGAAGCCAAGACAGAGAAAATAAGGTTTTAAAATGA

CAGAACCTTTATATTCACTTTGCCAGGGAGGGTTAGATTGCAGTCAAATGCTATTTACAC

TCTGTTATGCACTCTGCTACAAAGGATCCGTTCAGTAGTGACTTTAAAGTAATCTTACTG

TTAGATTAAACATACTCTCCATGGCCAAAATGTGAGTGTGTCCATCTATAAACATCAGGG

TTGCTTTACATGTTAGCCTATAGTACTGCAGCACAGGTACCAGGACAAAGTCATGTGCCA

ATCTTGTGCATCATTTCCAACAATAAAATGAACAGAATACCACATGGTAACATTCCATAA

TAGGCCATAATACAGAGCAAATACAATTGTAAAACCAGGAATTACATGTAATCATTTATT

CATTACCACTTTTTCAACTTTACCTGTGCATAATCAATCATAGTTGCCTGTGCATTATCA

ATCATGTAACATTTAGTTTTTATAACTCACAAAGCAGTTATATATGAAATATACATGTTT

CCCATTAATATTGACACAACTAAGTGAATGTATT

>Unigene92818_All

CTGCACGGTGCGCAGGACCTACGTTCTCATCGGGCTCACCTGGGCAGTTGGATTCACACC

AACAATAGTTGACGTCTTCATTGTGTATGCCATCGAGCCCACGCGCTTTTTCTCCTCGGT

GGGCCTTTGCCACCCGCTCACCATATACATCAGCCAAATCTATGCTCAGAAAACGCAGGT

GGTGCAGGGCCTCTACATGTCGTT

>Unigene92982_All

CCAGGCTAGCTTTCAGAGATTTTTCCCCTTAGAATGGATGGTGATGAGGGGGCCTTGCCA

GAACCCCTTGCAGATGAAGCAGGAATTGGAGGCGAAGGAGAAAAAAGAAAAAGAAAAAGA

ATACCACAGTAAAGAAAAGAAAAAAGTAAGTTCCTAAGAGCTAGCGCATCAAAGAGGCTG

AGAGAGACAGGAGAGCAAATGAAGGGGAGAAACAGAAGAAGAAAGGAGAGGAAAAGCGAG

TGAAAAAGAGAACAGATGGCTAGTGAGGGAGCATTTCTTCTACTGACAGAGAGGACAAGG

ACAGACAGCACATAGACACAGACACACAGACAGAGGCGGAGGAGAGAAGTAGGGAGAGAG

GGAGAGAGAAAA

>Unigene96506_All

CCTCACCAGCAAAATCATCAGCCTCATTGCAGGCATTGCTGTCCTGAGGAGCCTGTACAT

GGTTGTTCCACTGGTGTTTCTCCTTCTGAGGCTGCCCTTCTGTGGGCATCGTATCATCCC

TCATACTTATTGTGAGCACATGGGCATTGCCCGTCTGGCCTGTGCCAGCATCAAAGTCAA

CATTAGGTTTGGCCTTGGCAACATATCTCTCTTGTTACTGGATGTTATCCTTATTAT

>Unigene69531_All

CTGCCTGACACGGATGTCTTTCTTGGTCCTTTTTGCATGTATAGAAGGCATGCTCCTGAC

TGTGATGGCCTATGACTGCTTTGTAGCCATCTGTCGCCCTCTGCACTACCCAGTCATCGT

GAATCCTCACCTCTGTGTCTTCTTCGTTTTGGTGTCCTTTTTCCTTAGCCTGTTGGATTC

CCAGCTGCACAGTTGGATTGTGTTACAATTCACCATCATCAAGAATGTGGAAATCTCTAA

TTTGGTCTGTGACCCCTCTCAACTTCTCAAACTTGCCTGTTCTGACAGCGTCATCAATAG

CATATTCATATATTTCGATAGTACTATGTTTGGTTTTCTTCCCATTTCAGGGATCCTTTT

GTCTTACTATAAAATTGTCCCCTCCATTCTAAGGATGTCATCGTCAGATGGG

>CL10990.Contig2_All

AGCTAATATTGCCTATATGCCTTGTCTGTACAGAGGAGGCTAATATGAAAAACGCCAGTC

AAGGAGGTCTTCCGCACAAACACTCGCTACATTCTCTTTGCGCACACTCTTATCTGTGAC

TGCGCCTTTCTCATCTTTACCAACATGCTACTGCTGCTCATTGTCCGCCGCATCATCATG

CCAACCGGGCTGTGTGTCGTCATCTGCGTCGTCCTGGTCGACCTCAATATCGCCACGCCG

CTGACGCTTACGGCTATGAGTGTGGAACGTTATGTGTCCATCTGTATGCCGCTGCGCCAC

GCCGAGCTCTCCACGCCGCAGCGCGCCGTGCACACCATTCTGGTCATCCACGCGCTCTGC

TCCGTGCATGTGCTGGCGATGCTGCTCAGCCTTTTTGCCGTCATGCCGCTCAGCTTCTAC

TCATCGTCGGTCATGTGCAGCCTGGTCATGCTACACACCTACCGCTGGCAGGTGTACCTG

AACGATACCATCTTACAGCTTTACTTTGTCTTCATGTCGCTGGTCATCTGCTTTAGCTAC

GTGAAAATAATGGGTGTTGCGCGCGC

>CL11013.Contig2_All

ATTACAGGTGAGAGCATGCGGGATGAGAATGCCAGCGAGAGTGGGAATAGCAGTGAGGAT

TCAAGGCTGCTGGAGGTGACAGATGTGATGGAATTGAACCTCTACACAGCTACGGTGCAG

CTTGTGGTGTGGCCTTTCCTCTGCTTGGAGCTGTTCATGCTTTTCATCTTAAAGAAGAGG

GAGGTTTTCCAAACAGAGGCGCGCTATGTGCTGTTTGCCCAGTCCTTGATTGCAGATTCC

TGCTTACTGGTGCTAACTGATTTTGTGGTGATCACTTTGCATGTTCGACTTTTACTGCCC

ATTGCTGCCTGTATACCAATAGTTATAATCATGGATGTTTTTTCCAATGTTTCCCCATTC

ATTATAGTAGTCATGTGTGTAGAGCGGTATGTCGCTATCTGCATGCCCCTAAGGCATGTC

AACATCTTCAGCCCGGATATGACTATTCTTTACAAAGCCGTGATCTGGTTATTGTTTTTT

TTTCATATCATTGGTTGACCTTTTCATTCTCCTTGGCTTAACCACAAAACACTACTTCGA

GGAGCTTAACTTCTGTTATTATGAGATACTGTTTGTAGAGTACTGGCATATGGAGATGAG

AGGGTTCTTTTACATCACTGACTGCTTAATTATTGTGTCTATCCTGCTCTTCTGTTACGG

CTCCATCATTGTGGTGGCCCGCAGAGCCTCCGGCGATGACAAGCTGGCTGCCTCCAAGGC

CCAGCGCACAGTACTGCTCCACTTTTTGCAGCTCCTGCTTTCCACTGTGGAGGTCTTTTT

CCCTTATGTGGAGTCACAGGTTATAGAGATCAGTTTAGACCTCTACCTCAGAATGCGCTT

TGTAAACTTCATAGTATTTTCTCTCATTGCAAAGATAATGAGCACTCTTGTCTATGGCTT

TAGGGATGAAAAGATCAGGGCATCCATGAAGTTTTATATAACATTTAATAGAAAAGTAGC

TCACGTCCCAAAGAATTAATGTTCACATGCTGTGTAATATATTTCTATGTGCACAACTGC

CTTCTGAGCGACTGTAAATGTTTTTGTATGGTTCTCAGATTTAATGTCAATGTGAATAAG

TGACAAGATGCTGTTCTTCAATACTTTTGGTGGTAAACATTCCGCCGTGTTTAAACTGAC

GTTGTATGAAAAAGGCAATGAATCAACATTCACACAGTTATACTGTCTATGATGTTATGT

TTTGTCCATGCATATTATGTACTGATCATGTTTATATTTCAATAAATGTTTTAATACTGC

TGTTTCTTAAAATGTTGGCACCCCCTCCCAACACACAACACTTTAAACTGTAAGTAGTTC

ATGATACAGAAGGCAATTAACACCCAAAGGGCAATGGACACACACTGAAAAAATGGGCTG

TTGAAATAACATTAAAAAATCTTGTACATCGGTTGCACATATAAAAATCATGTTC

>Unigene228_All

TAACAAATGACTTTTCATATGGTCCACAGATTATTCTTTATTCTCTGTATTTTTGCACTG

GTTTATCGCACATAGTACTTAGGCAATAATGTTAATACTGCTGACATTTTCTTTGTCAAA

ATGGTAGATGAGCCATGTCTGAGAATAGTTCCCAGACGGTGAACAGCAGTGAGCCCGGCC

TGTTATCTCTCCAGAGCTCCGTGCGAGTTACCGTCACCACCACCGTGATCCAGCTCCTCG

TGTGGCCTTTCATTGGCATCAACCTCTTCATGTTCCACGTGTACCGGCAGCGGGAGGCTC

TGTGGTCCGAGCCACGCTACGTGCTCTTCACCCAGACACTGCTGGCCGATGCCTTCTTCC

TAGCGCTCACCAACTTCGTGGTGTTGACCATCCACAGCAGACAGCTGCTGCCTGTGGCTT

TCTGCGTGCCGGTGTGCATGTTAATGGAGGGCCTCACGCACCTTTCGCCAACTGTCATTG

TGGCCATGTGTCTAGAGCGCTACGTTGCCATCTGTATGCCTCTGCAACACACCAACATCT

TCTCCCCCCACAGAACGAGGGTCCTCATTGCCATAGTCTGGTTTGTCAGCTTCTTAAAAC

CCCTCATAGATTTCAGCATCTTCCTGAGCCATGTCACCGAGAGTTATTTCATTCAGCCAA

CGTTTTGCAGCTATGAGATTATGTTGCTGAGGACTTGGCACATGGTGATGAGGGGGAATC

TGTATATACTAAATTATCTAGTTGTTCTTGTCATTCTGCTTTTCTGCTACATGTCAATAA

TTGTTGTCGCGCGCCGGGCTTCAGGCGACAACAAGCAAGCTGCATCTAAGGGCCAGCGTA

CGCTCTTTCTCCACCTGCTGCAGCTTCTCTTGTGCACGTTAGAAACAATATGCCCCTACA

TCGAGGCTCAGATCCTACAGACTGGCAATCTGTTTGTCTACATGGTTGTGCGCTCTTTCA

ACTTTATGGCCTTTTCTATTGTCTCCAGGGCAGTGAGCCCCCTCATCTATGGCTTCCGAG

ATGAGAAGTTTTATGCTGCAATTAAGAACTATGCCAGTCGTGCCGTTAATAAAGTTGCCT

CTGTAAAATGAGTTCACTGCTGAATTGATTTTGATACCTTTGAATGAGTAGGATGCAACA

AGTAGCATTCATTTGTCACTCTGTTAAAAAATGACCATTACAGGAGTTGTGATTCCTATT

TAATAAACGAAATGGCCTCCTACTAAAAAAA

>Unigene66561_All

TTCTCAAGGTGTCCAAGACACACGGAGCCACGGAATCTCACAGGTGTCTGAGAATTCCTC

CTCCTGGGACTCTCAGAGGATCCAGAACTGCAGCCGGTCCTCGCTTTGCTGTCCCTGTCC

CTGTCCATGTATCTGGTCACGGTGCTGAGGAACCTGCTCAGCATCCTGGCTGTCAGCTCT

GACTCCCACCTCCACACCCCCATGTACTTCTTCCTCTCCAACCTGTGCTGGGCTGACATC

GGTTTCACCTCGGCCATGGTTCCC

>Unigene97245_All

TGTACTTCTTCCTCTCCAACCTGTCCTTGCCTGACATCGGTTTCACCTCCACCACGGTCC

CCAAGATGATTGTGGACATCCAGTCTCACAGCAGAGTCATCTCCTATGCAGGCTGCCTGA

CTCAGATGTCTCTCTTTGCCATTTTTGGAGGCATGGAAGAGAGACATGCTCCTGAGTGTG

ATGGCCTATGACCGGTTTGTAGCCATCTGTCACCCTCTATATCATTCAGCCGTCATGAAC

CCCTGTTTCTGTGG

>Unigene14297_All

ATGGGGAGGTCTTGATTGGGGGACTGTTTCCTCTTCACTACATGGCCTCAAAGCCAGATC

AGAACTACACCTCCAGACCTCAGCACAGCCAGTGTAGTGGGTTTGATCTTCGTGCCTTTC

GCTGGGTCCAGACTATGGTATTTGCCATCGAGGAGATAAATAGAAACACCACCCTTTTGC

CAGGGCTTACCCTGGGCTACAGAATAATGGACAGTTGTGATCACGTCCACACAGGCCTGA

GGGGGGGTTTGGCACTGGTCAGCGGGTCCCTGCCTCCCCGTCAGAGTCCCACCGAAGGCT

CCCAGTGCACCCATAACGCCCCAGTGTCTGCTCTTATCGGTTTAGCGTCCTCCACACCCA

CCCGTGCCGTGGCACACACACTGGGACCCTTTGGGATACCCGTGGTTAGCTATTTTGCCA

CCTGCACGTGTCTTACAAACAAGCAAGTGTACCCTTCCTTCCTGCGCACAGTGCCTAGTG

ATATGTTTCAAGTGCAGGGGCTGGTTCAGCTGGTTGCCCACTTCAGCTGGCACTGGGTTG

GCACAGTGGGCACGGAGGACGACTACAGTCGCTATGGCATCCAGGCATTTTCTGAGCAGC

TGCAGGAATGGGGTGGCTGTTTGGCCTTCCATCAGACTATCCCAAAAGAACCAACAGTGG

CCAAAATCAGGGCCATCGCGGACACACTAGAGTCAACCACTGCCCGAGTAGTCATAGCTT

TTGCCACAGAGGGACAGCTGCTTGACCTCCTCACTGAAGTTGCCCGACGCAACCTGACTA

GGTTGCAGTGGGTGGCCAGCGAGGCATGGGTGACTGCCAGTCTACTCACCGCACCCGAGT

TCCAGCCTGTGCTCGCGGGCACGCTGGGTTTTTCGTTTCGAGGCACTGTGATCCCTGGGC

TGTCCGACTTTCTCCTGAAGGTGCGTCCGTCTCCAAGACCGCAGTCGGCCTTCACCAACA

TGTTCTGGGAGGAGTTGTTTGGGTGCAAGCTGGTGTTTGGGTCTCACAGTAGCAATACCT

CCTCCAAAACTCAGCCACAGTGTAGTGGTAATGAAGATCTGACCTCACGCAAAACCAGCT

ACAGTGATGTGTCACAGTTCAGAATATCTTACAATGTGTACAAAGCGGTGTATGCCATCG

CTCATGCTTTACATGAACTGCTGCAGTGTGCTACTCTGTGGGAAAACACCACACGTGTGG

GCTGTAAAACAACCACAGCATTTACACCAAGGCAGCTCCTAATGCACTTAAAAAGGGTTA

ACTTCATCAATCAGTTTGGTGAGAAAGTCTCCTTCGACTCTAATGGGGAGCCTGTGCCCC

TATATGATATCATAAATTGGCAGAGGGACCACAAGGGTGGCATCCTATTCAAGAAGGTGG

GAAGCTACGATGGCTCAGCTCCTAATGGCCAACAGTTATGGATGGATGAAGATGCCATAG

TGTGGACTGAGGGACATCAGGCTCCTGTGTCTGTCTGCAGTGTGGCCTGCCCTCCGGGGA

GTAGAAAAGCCTCACGCCCAGGAGAGGCTGTGTGCTGCTTCGACTGTCTGCCCTGCGCTG

AAGGACAGATCAGTAACACTACAGGAGCTACGGAGTGTCTCAGATGTCCTCTGTACTACT

GGTCTGACAGAGACAGAGTGGCTTGTATAGCAGGTGTCGAGGAGTTTCTTTCCTTCCAGG

AGACTCTGGGTATCATCCTTGTTGTTCTATCTCTACTGGGGGTTTCTGCAGCTGCAGTAA

TAACAGTGATCTTCCTCTGTCACAAGACCACACCCATTGTAAGAGCCAACAACTCCGAGA

TCAGCTTCCTGCTACTGCTGTCACTCAAACTCTGCTTCCTTTGCTCCCTGGTGTTCATGG

GCCGCCCCTCGCCGTGGTCCTGCCGCGCCCGACAGGCGGCGTTCGGCATCAGCTTTGTCC

TCTCGATCTCCTGCATCCTGGTCAAGACCATAGTGGTGCTGCTTGCCTTCCGCTCCACCT

TGCCTGGCTCCCGTAAGGGGCAGCTGTTTGGGCCTCCCCAGCAGAGGGCCTTCATCTTCT

GCTGCGCTGCCGTGCAGGTGGTCCTGTGTGCCAGCTGGCTGTCTGTGTCCCCCCCTGTGC

CCACTAAGAACACAGCCTACCAGGGAGGGAGGATTCTGCTGGAGTGCAAAGGCACGTGGC

CCCAGGGCTTCTACCTGGTTCTCGGGTACATCGGCCTGTTGTCCTGCATCTGCTTTGTGC

TCGCCTTTATGGGCCGCAAACTGCCGGACACTTTTAACGAAGCCAAGCTAATCACCTTCA

GCATGTTAATCTTCTTCGCTGTGTGGATCTCCTTCATACCCGCACACAATAGCTCCCCTG

GGAAATATACAGTGGCAGTGGAGATTTTTGCTATCCTGGCTTCCAGCTTTGGACTACTGT

TTTGCATCTTTGCACCAAAATGTTATATTATTCTTTTTAAACCTGAACAAAACACCAAGA

AAGGAATGACAAGGAAATACTCAAATTAATGACCTCCTGCTTTTGTGTGTATCATTCATG

TTTGTATTTTCTGTTCATGTGAACATACATTAAAATTCGAAATTCTGGTAAGATTGTTGA

TGCAAGTTTGTGCTTATGAATAATTTCTCAGCATTGTGTTATAGCTGTGAGGTATAGTTG

TAATTTTCCCATAACATCAATGAGTACAATACATTCACACAAAACTTTATCTTTTCCAG

>Unigene79724_All GGAGAGGGGAGGAGAGGAGAGGTCTGCAGTCCTGAGATGCTTTCTAGAGATGGCTGTTCT

CCACACCATGACTCTGTTCTTCGGCCTCCTTTACCCCAGCCTGAATGCCTCTGATGGGGG

ATGCCAGCGCTGGCACGACCTGGACTCCACCGTGGTGCACAAGGATGGCCAGGTGGTCCT

GGCAGGGATGTTTCCCATTCACTCTAAAGGAGTCGAACAAAAACTGACTTTCACAGAAAG

ACCAGGCAACAGGCGATGTCGAGGGTTTAATCTGCGGGTGTTTCGCTGGTCCCAGGCAAT

GGTGTATTTCATCGAGGAGATCAACAGAAACCCAGCTCTGCTGCCAAACATAACTCTGGG

GTACAGGCTGTACGACACGTGCGGAGTGGAGATGTTCTCTCTCAGGACGGCCCTGTCCGT

GATCTCACAGCCACTCAAGAGGAACGCCACGGGGGTTTGTGTGTCTCCCAGTGTGCCGCT

CATAGTGGGAGACTCTGGATCCTCTCTGTCAATGGCCATCTCCAGAGTGCTCAATCTCTT

TAAGGTCCCATTGGTCAGTTACTTTGCATCCTGTGCCTGTCTAAGTGACAAAAGCCAGTT

TCCATACTTTTTCCGCACCATCCCCAGTGATGTGAACCAGGCGAGAGCCCTTGCCTATCT

GGTCAGGCACTTTGGCTGGACCTGGGTGGGCACCATGGGAGCGGACGACGACTATGGGCG

GACAGGCATCGACATGTTCACAGCCGAAGTTACTCGCCTGGGCGTGTGTGTGGCATACCG

CATCACCATCCCCAAGCTCCCCTCACAGGAGCAGCTGCGGGAGATCGTGGCAACCATCAG

GGACTCCACAGCTGAAGTGGTAGTGGCGTTCGCCATTGAGGAGGACATCGAGCCTGTTGT

GAAAGAGATGGTGTCGCAAAACGTGACAGGAAAGCAGTGGGTGGCCAGCGAGGCCTGGGT

CACCTCCACTCTCATATCCACTCGAGAGAACTACGCCTCTCTCAGCGGCACCATCGGCTT

CGCTATCCGCCGGGCTGAGATGCCTACCCTGAAGCACTTCTTGCAGGACCTCAGACCCCT

GGAAAGCCCCTATAACCCCTTTATCCGAGAATTCTGGGAAACACAGTTCCAGTGCTCTTT

CAACACCACGCTTCCTGCAGCCTCTATGGCAGATCCCATGCATTACACCAACACCTGCAC

TGGCAGGGAAAGGGTCGAAGACACGGACAGCATTTACAATGATGTGTCACAGCTCAGAGT

GACTTATAACATGCACAAGGCTGTTTATGCAGTCGCTCATGCCCTGCATAACTTACTGAC

AGACCAGAACTCCACTGTCTCAATACATAATCTTCATCCATGGCAGGTGGTTAGATACTT

GCGCACAGTCAACTACACAAACATCTTCGGAGATGTGGTGCATTTTGATGAAAACGGGAA

CCCTGTGGGTGCCTATGACATTGTGAACTGGCAGAGAGGCTCCCCCGACGGGCCTGTGGA

GTATGTGACTGTTGGCAGATTTGACTCCAGCCTGCCTCCCACGCAGCAGCTGCTGGTGAA

CTCCGACACCATCATTTGGCACAGAGGGCAGAAAGAGGTTCCTAAATCAGTCTGCTCTGC

CAGTTGTCAGCATGGCTACAGAAAGGCCACGAGAGAGGGCCAGCCTGTGTGCTGTTATGA

CTGTGTCCCCTGTGCAGAGGGGACTATAACTAATGACACAGACCAGCCTGAATGCATTCC

ATGCCCTGATGACTACTGGTCCAACTCCCGGAGGGACGCCTGTGTGCGGAAGCAGATTGA

GTTCCTGTCCTACACCGAGGCCTTCGGCATGGCCCTGGCCGCCAGCGCCATCCTGGGTGC

CGTCTTCACATCTGCGGTGGCCGCCATCTTCCTGCAGCACCGAGGAACCCCCATCGTCCG

CGCCAACAACTCGGAGC

>CL1147.Contig5_All TGGACGTAGTCATGGCACAGACACCTCTGCTCCTACTGCTGCTGAGCCTCTGTGCCAGAG

CATCACTGCAGAGCTGCAGTCTACTGGGCCAGCCAGCACTGCCTCTCTTGTCTTCCGAGG

GCGACATCAATATCGGGGCAGTTTTTTCTTTACACAGAGAGCCTGTGGTGAAGGCTCATA

CTTTCACCTCAGAGCCAGAGCCTACCTCATGTATCAGACTGAATCTGCGTGAGTTTCAGT

TTGGCCAGACCCTTATTTTTGCAGTAGAGGAAATAAATAACAACACTAACCTACTACCAG

GCATAACTCTTGGATACAAGATCTATGACGCTTGCAGTTCCATTATTCTGGGAATTCGCT

CAGCTATGGCGTTGATGAACGGCTATGGACAGACACTGAGTGACACATCATGCCAGCGAC

CACCAGCTGTTCAAGCCATTGTTGGGGAGTCTGGCTCCACTCCCACTATTGGCATCGCAT

CTGCAGTGGGACCCTTCCGCATCCCTGTGGTCAGTCACTTTGCCACATGTGCCTGTCTCA

GCGACAAAGACAGGTTCCCAACTTTTTTCAGGACAATCCCCAGTGACTACTATCAGAGCA

GAGCACTTGCACAGCTGGTCAAACTATTTGGCTGGACTTGGATTGGAGCCGTTAGAAGTA

GGAATGATTACGGAAACAATGGAATGGCCACTTTTATTGAGGCCGCAGAGGAGCTTGGCG

TTTGTGTTGAATATTCTGAAGCCATCTTCAGAACAGATCCCAAAGAGGAAGTACTGAAGG

TCGTTGAGGTGATGAAAAGGGCTACTGCCAAAGTAGTAGTGGCATTTTTATCACTGGGGG

ACATTATCCCTCTTTTGAATGAGCTGGCACTGAATAACATTTCAGGGCTGCAGTGGGTGG

GCAGCGAGTCGTGGATTACTTCCAGAGGCCTATCGGAGACAAAATCCTTCAGTTTCTTAA

CAGGTGCCACTGGGTTTGTAATTGGAAACGTAAAGCTTAATGGCCTGAAAGAGTTTCTTG

TCAATGTGCATCCTTCTAAAGCACCACACAATCTCTTTCTCAGAGAGTTTTGGGAGACAG

CCTTTCAGTGCTCCTTTGAAAACAGCCCCGTGGAGGGTTCACGGTGTACGGGATCAGAGA

GCTTACTGACTCTGCAGAATCAGTACACTGATGTATCTGAGCTGCGCATCTCTAACAAAG

TCTACACAGCTGTATATGCCATTGCACAATCTCTTCATAATCTTATGATGGACGCTACTG

GCAATGACACCAAATGGAATGTATTTGTTCCTGAAAAGGTGCTTGAGTATTTAAAGAAGG

TGAATTTCACCTCCAAGACTGGGGAGCAGATATTCTTTGACTCCAGGGGTGACCCTGCTG

CAAGGTACGAGCTGGTCAACTGGCAGCCGGATGATGATGGAACTTTGCAGTTTAAGTCTG

TGGGAATCTATGATACATCTGTGCCCTCAGAGCAACGTTTTGTTCTGAACCAAGGAGGCC

TGGTATGGGCTGGTGGCCAAACACAGGTGCCGGTGTCTGTGTGCAGTGAGAGCTGTCCCC

CAGGCACCAGGAAGGCTGCACAGAAAGGGAAGCCTGTGTGCTGCTATGACTGTATACCAT

GTGGAGAGGGAGAGATCAGCAATGAAACAGGTAACTCAGAGACAAAGTATACACTGCATA

GAAAAGTGGCCTGTAGCGCCCTATGCAATTTCATAAAACTGTTCAAAATGCACTGTCTGA

GCTCTGGTTTACACAACCCGTACCAATACTGCTGGATTGCATCTCTAATCTCGGTGACAA

TAATTCTCTTACAGATTCTAATAACTGCGAACCTTGCCCAGGACAGTACTGGCCAAACCC

TGGCAGAGATAAATGCATATTAAAGGCTGTAGAGTTTCTCTTATTTACAGAAGTTATGGG

AATAGTGTTAGTATTTTTCTCCCTCTTTGGTGTCTTGATAACTGTAATCACTGCCATTAT

TTTTATAATAAAGAAAGATACTCCAATAGTGAAAGCTAACAACTCAGAGCTGAGCTTCCT

GCTGCTCTTCTCACTGACTCTGTGCTTCCTCTGTTCTCTTACTTTCATTGGTCGGCCCTC

TGAGTGGTCCTGTATGTTGCGTCACACAGCGTTTGGGATCACCTTTGTTCTCTGCATCTC

CTGTGTTCTGGGGAAAACAATAGTGGTGTTAATGGCCTTCAGAGCCACTCTTCCAGGCAG

TAATGTCATGAAGTGGTTTGGGCCTCTGCAACAGAGACTCAGTGTTCTTGGCTTCACTCT

CATACAAGTCCTTATATGTGTGCTGTGGTTAACTATATCCCCTCCTTTCCCTTACAGAAA

TATGCACCTTTACAATGATAAGATCATTCTAGAATGTGATGTGGGTTCAGCTATAGGATT

CTGGGCCATATTAGGTTACATCGGACTACTCGCCATCCTGTGTTTCTTTCTGGCTTTTCT

GGCTCGTAAACTACCTGATAACTTCAATGAGGCCAAATTCATCACATTCAGTATGCTCAT

ATTCTGTGCAGTTTGGATCACTTTTATCCCAGCTTATGTCAGCTCTCCTGGGAAATTTAC

TGTAGCTGTAGAGATATTTGCCATTTTAGCATCTAGTTTTGGATTACTTCTTTGCATTTT

CACCCCAAAATGTTACATTATTTTAATAAAGCCAGAGAAAAACACAAAGAAACAGTTAAT

GGGAAAGAAAGCACCAGGTTCCCTATAGGTGGCAATTAAGGAACTTCCTGCTCCCAAATA

TTTTAATCATTGTGAAATATTCATAATTAACTTCCATTCATGTGTATGTATACTTTGTAA

CAACATACCAAAAACAAGTTACATTTCATTAAAATACACATTGCTTTACTTCAACCCTCA

ATCGGATCACAAAATATGCACTCTTATGTGTTTATTAAACACTACTTTATCAAGCTACTT

TAAGTCTGATCACAATGGTATTACACAGGAGAGAATCCACAAAGGGCTCTGATCCACTAG

CTTTGCTCTCCACAGGCCCCATTCCAGGCTGTGGTGTCCTCCAGGTCAGGCCCAGACACA

CAGGACAGCCCCTCTTCACATCTTTCTCATGCATGCTGTGGATATGCATTGTGTACTACA

AAATAAAAAATAAGATTGCCTTTTCATATCTCAGTTTCCAAAATTGATTATGTTGCTGCA

TGTTATGAAAACATAAATATCTATGGAAGTTTGTATGAATGGCAGTGTAATGGGTTGTAT

GACTCTGTAAACATGATTTTCTAATATATATATTATTGTGAAATAAAGAGCTCCTTCCAG

A

>Unigene8764_All

TGGACGTAGTCATGGCACAGACACCTCTGCTCCTACTGCTGCTGAGCCTCTGTGCCAGAG

CATCACTGCAGAGCTGCAGTCTACTGGGCCAGCCAGCACTGCCTCTCTTGTCTTCCGAGG

GCGACATCAATATCGGGGCAGTTTTTTCTTTACACAGAGAGCCTGTGGTGAAGGCTCATA

CTTTCACCTCAGAGCCAGAGCCTACCTCATGTATCAGACTGAATCTGCGTGAGTTTCAGT

TTGGCCAGACCCTTATTTTTGCAGTAGAGGAAATAAATAACAACACTAACCTACTACCAG

GCATAACTCTTGGATACAAGATCTATGACGCTTGCAGTTCCATTATTCTGGGAATTCGCT

CAGCTATGGCGTTGATGAACGGCTATGGACAGACACTGAGTGACACATCATGCCAGCGAC

CACCAGCTGTTCAAGCCATTGTTGGGGAGTCTGGCTCCACTCCCACTATTGGCATCGCAT

CTGCAGTGGGACCCTTCCGCATCCCTGTGGTCAGTCACTTTGCCACATGTGCCTGTCTCA

GCGACAAAGACAGGTTCCCAACTTTTTTCAGGACAATCCCCAGTGACTACTATCAGAGCA

GAGCACTTGCACAGCTGGTCAAACTATTTGGCTGGACTTGGATTGGAGCCGTTAGAAGTA

GGAATGATTACGGAAACAATGGAATGGCCACTTTTATTGAGGCCGCAGAGGAGCTTGGCG

TTTGTGTTGAATATTCTGAAGCCATCTTCAGAACAGATCCCAAAGAGGAAGTACTGAAGG

TCGTTGAGGTGATGAAAAGGGCTACTGCCAAAGTAGTAGTGGCATTTTTATCACTGGGGG

ACATTATCCCTCTTTTGAATGAGCTGGCACTGAATAACATTTCAGGGCTGCAGTGGGTGG

GCAGCGAGTCGTGGATTACTTCCAGAGGCCTATCGGAGACAAAATCCTTCAGTTTCTTAA

CAGGTGCCACTGGGTTTGTAATTGGAAACGTAAAGCTTAATGGCCTGAAAGAGTTTCTTG

TCAATGTGCATCCTTCTAAAGCACCACACAATCTCTTTCTCAGAGAGTTTTGGGAGACAG

CCTTTCAGTGCTCCTTTGAAAACAGCCCCGTGGAGGGTTCACGGTGTACGGGATCAGAGA

GCTTACTGACTCTGCAGAATCAGTACACTGATGTATCTGAGCTGCGCATCTCTAACAAAG

TCTACACAGCTGTATATGCCATTGCACACTCTCTTCATAATCTTATGATGGACGCTACTG

GCAATGACACCAAATGGAATGTATTTGTTCCTGAAAAGGTGCTTGAGTATTTAAAGAAGG

TGAATTTCACCTCCAAGACTGGGGAGCAGATATTCTTTGACTCCAGGGGTGACCCTGCTG

CAAGGTACGAGCTGGTCAACTGGCAGCCGGATGATGATGGAACTTTGCAGTTTAAGTCTG

TGGGAATCTATGATACATCTGTGCCCTCAGAGCAACGTTTTGTTCTGAACCAAGGAGGCC

TGGTATGGGCTGGTGGCCAAACACAGGTGCCGGTGTCTGTGTGCAGTGAGAGCTGTCCCC

CAGGCACCAGGAAGGCTGCACAGAAAGGGAAGGCTGTGTGCTGCTATGACTGTATACCAT

GTGGAGAGGGAGAAATCAGCAATGAAACAGGCCCCATTCCAGGCTGTGGTGTCCTCCAGG

CCAGGCCCAGACACACAGGACAGCCCCTCTTCACATCTTTCTCATGCATGCTGTGGATAT

GCATTGTGTACTACAAAATAAAAAATAAGATTGCCTTTTCATATCTCAGTTTCCAAAATT

GATTATGTTGCTGCATGTTATGAAAACATAAATATCTATGGAAGTTTGTATGAATGGCAG

TGTAATGGGTTGTATGACTCTGTAAACATGATTTTCTAATATATATATTATTGTGAAATA

AAGAGCTCCTTCCAGA

>CL11881.Contig2_All

GTGATCATAGGAGGACTGTTTGAGGTCCACATGCTTGCTTTACAACCAGAGCTGTCTTAC

AGGAGTAAACCAAAACAGATCTGGTGTCAAGATTTTACCTTGTATGGCCTAAAAACAGTA

ATGGCGATGGCTTTTGCTGTTGAAGAGATCAACAGAAACCCAAACCTGCTGCCCACTGTA

AAGTTAGGCTATCGAATGTTTGACAATTGCATGAGGCTGGATGTGGCATTTCGAGCAGCC

ATGGCTCTTGTCAGCGGAACAAACAAGTATTCTTCAACGCAGAACTGCAGCGGTTTGCCT

CCCGTGCTAGGCATTGTGGGGGATCCTATCTCCTCCAATTCTATTGCCATATCAAGTGTT

CTGGGATTGTTCCGTGTTCCAATGGTAAGCTTCTACGCAACCTGTTCCTGCTTAAGTGAC

AGA

>Unigene26668_All GGCAGTGAGAAGGTCAGGCCAGAAGAGCGTCACCCATGGCCAGCTCTCCGTCAGCCCTCT

TACTGCAGGCTTCTGCCCCATTGCTGCTGCTGCTCTGGCTGCGTGGAGATGCAGTGGTGG

CGGACATGGCAGAGCAGGAGCAGAGCTGTGTGCGCTGGGACAAGCCTGAGGGGGTCGATG

ACCGTGGCCTCTCTCAGGACGGAGACGTGGTCATCGGAGGGCTCTTTGTGGTGCACAACC

AGCCGCCTAGTCCAGACCTAAGTTTTACTAGGAACCCTGGTCAAGACCCCTGTTTTAGTT

TTCAGAAGGAGCCATATCTCTGGGCCCATGCTATGGTATTTGCTGTTGAGGAGATAAACA

GGAATCCCCACCTGCTCCCAGGAGTACGGCTTGGCTACCAAATCTTAGACAGTTGCACTC

GTTACCCCTGGTCAGTGCGAGCAGCCATGTCTCTGATCAGTGGAGGGAACCACTCGTGTG

AGTCCACTGGACCTGTGCGAGTGATTGTAGGAGACGCGTCTTCTACCCAAACCATCATGC

TGTCGAGGATCCTCAGCCCTCTGCAGGTGCCACTGATCAGCTATCAAGCCAGCTGTGCCT

GTCTGAGTAACAGGCTTGAGTTCCCCAATGTGTTCCGCACGATTCCCAGTGATGTTTATC

AGGCGTGGACCATGGCCCACCTTGCTGAGCATTACAGGTGGACGTGGGTGGGAGCTGTGG

TTGTGAATAACGACTACGGTCTGCTGGCAGTGCAGGCCTTCAGGGAGAGGGCACAGGGCT

CAGGCATATGCCTGGCTTTCTTTGAGACGTTCGACCGCGAGACTTTGGCCCAGGATATGG

AGCGCATAGCAACTACTGTGCAGACGTCTTCAGCTCGAGTGGTGCTGGTGTTTGCCTGGT

ACACGGACGTGGGCGCTCTGCTGCTGGAGCTTGCCAGCAGGAATGTGACAGGCAGACAGT

TCCTGGCCAGCGAGGCCTGGAGCACCAGCTCATACCTGCTGAATGACCCTGCCCTGAGAA

CTATCACCAGTGGAGTGTTGGGAGTGGCCATTCGGAGCGCACCCATCCCAGGTCTGGAGG

CTCACTTGCGAAAGCTTCATCCTTCCCAACACCCCAGAGATGCCCTTTTGAGAGACCTGT

GGAGGGAGGAGTTTGGTTGTGAACCTGTCAGTGCCTCTAGCCCTGACTCACTGTCCTTAC

CCCCCTGCAGTGGGACCGAAAGTCTGGAGAGGGTGCAGAGCACCTTCACAGACACAGCTT

ATCTACGGGTGACCTATAATGTGTATTTAGCAGTGTATGCTGCTGCACACGCCTTCCACT

CTTTACTGGAATGCAGTCCAGTAGGGGACAGTGGGATGAACAGAAGCCAACCATGCTCGT

CCCAGTATAATATCAGCGCAGCTGAAGTTCTGCAACACCTGGGTCAAGTGAACTTCACCA

CTCAGATTGAAGAGAATTTCTTCTTCCAAGGTGGGGACATCCCAGCTGTGTATGACCTTG

TGAACTGGCAGGCGACACCTGGTGGTGTACTAAAGTACAGCACAATCGGACGTGTGGAGG

GCTCCAAGCTGCACATGGAAGAATCAGCCATCTGGTGGGCCACCGAATCACAAGTGCCAG

TGTCTGTGTGCAGTGAGCCCTGTCCTCCAGGGACTCGGAAAGCGAGACGAAAGGGGGAGC

CAATCTGCTGCTTTGACTGCCTTCCCTGTGCAGACGGAGAGATCAGCAACAAAACTGATT

CTCTAACTTGTTTACGCTGCCCCCTGGAGTTCTGGTCCAACGATCAGAAGAACATGTGTG

TTCCTCTGACTGTGGAGTTTCTCTCCTTTACTGATGCTATGGGCATCACACTCACAACCG

TGGCTGTGTCGGGTGCTGTGATGACAGCGGCCGTAGCAGTTGTCTTTGTGTACAACAGAC

ACACACCCATTGTGAAAGCCAACAACTCTGAGCTCAGTTTTATGCTCCTGATGTCACTGA

AGTTGTGCTTCCTGTGTTCTTTGTTGTTCATGGGGCAGCCAACAGACTGGTCGTGTCGCA

TTCAACAGGCAGCGTTTGGGATTAGTTTTGTTCTTAGCATCTCCTGCATCCTGGTCAAGA

CTATAGTGGTTCTAGCTGTCTTCCAGTCAGCAAGACCTGGTTCTGACTCTGTGATGAAGT

GGTTTGGACCGGGTCAACAAAGAGGCAGTGTGCTTCTCTTTACCTGTGTGCAGGTGGTTA

TATGTGCTGTATGGCTATCCATAACACCTCCTCAGCCTCACAGGAACGTTGGACTGAAGG

GGTCAAAGATCATTCTTGAATGCACACTGGGGTCAGTGGAAGGGTTTGCAACATTGCTGG

GGTACATTGGTCTACTGGCAGCAATCTGTTTCATGCTGGCCTTCTTGGCTAGAAAGCTAC

CGGATAACTTTAACGAGGCCAAGTTCATCACCTTCAGCATGCTCATCTTTTGTGCTGTGT

GGATCACATTTGTCCCTGCCTATGTGAGCTCACCAGGGAAGTATGCCATCGCTGTGGAGA

TCTTTGCGATCCTGGCGTCTAGTTTTGGCCTCCTTCTTTGCATATTTGCCCCAAAGTGCT

TCATAATCTTACTCAGGCCTGAGAAAAACACAAAGAAATTTCTTATGGGGAAGGCACAGG

GTCAGCAGTAATTTCGTTAGGAATACATCGTATTAAGCAAATAAAAA

>Unigene82853_All

CAACAACTCAGAGCTGAGCTTCCTCATCCTGGTCTCTCTGAGCCTGTGCTTCCTGTGTGC

CCTGCCCTTCATCGGCGAGCCCACCCCCTGGACCTGCATGCTGCGCCACACGGCGTTCAG

CATCAGTTTCTCCCTCTGCATCGCCTGCATTCTCAGCAAGACGGTGGTGGTCCTCATGGC

CTTTAAGGCCACGCTGCCAGGCTCCGTGGTCATGAAGTGGTTCGGCCCCGTCCAGCAGAG

GGCCATGATCTCGGTGGGGGCTGCCGTGCAGGTGGTGATCTGTGGGGTGTGGCTGACGGT

GGCGCCCCCTACGCCTCGGAAGCTTTCCAGCCGAGAGACGACGCAGATCATCCTGCTGTG

TGACGAAGGCTCCGCGCTGGCCTTCTCTCTGGTGCTGAGCTACATCGGGCTGCTGGCCAG

CCTGAGCCTGCTGCTGGCCTTTCTGGCCAGAAAGCTTCCTGGCAGCTTCAACGAGGCCAA

GCTCATCACTTTCAGCATGGTGATATTCTTCGCTGTCTGGGTGGCCTTTGTGCCGGCTTA

TGTCAGCTCTCCGGGGAAGTACTCTACAGCCACAGAGGTGTTCGCTATCTTAGCCTCCAG

CTATGCACTCCTGGTCTGCCTGTTTTTCCCCAAGTGCTATGTTATTTTGCTGAAGCCGGA

GAAAAACACTAGAAAACATTTGATGGCTAAAACTGCACAAGACAACAGATACTGAGATGA

CATGGCGGTGTTCATATAAAAAAAATAATAGATGTCCATATGTTGCATATCAACATATTA

GAATAGATGGGAAGATTTATGAATTATAAATCTACCCTTGTGGTGATCTTTACTCTTGTA

CATTTGCCACTAAGCTTGCCATTGAAATTTCTACATAAAGACTTTTCTCAGAAACACA

>Unigene86482_All

GATCATGTTTGGACATGGAATTCATATATGCCTGTGGCTGCATTCATGCTTTGCCTGTAA

TCTACTGGGTGACTTTGGAATGCCTAGTTCAACAGAGGATGGAGATATTGTGGTTGGCGG

CATTTTCCCTGTGTCTAATAAACAAAATCACTTAAGTGCCTCATTTGAGAAGGAACCTCC

AGATGCAACATGCAATGGGTTTGACATGCGTGCCTTTCGGTGGACTCAGGTGATGATCTT

TGCAATTAATGAAATCAACAGTGATAGCACTTTGCTTCCAAATATCTCTTTAGGGTATAG

GATCTTTGATTCATGCGCTTCCCCCACTAACACGTTACGTGCCGCTCTTATGCTGTTGAG

TGACCCAAATGGAGATAACTCTACTGTTCAGTGTCAGCCACCTGTGTCTGCATTAATAGC

AGAGTCGGGGTCATCACAGTCAATGGCTCTTGCTGGAACATTTGGACCTTTTAACTTGCC

AATGGT

>Unigene88383_All

TGATCCTGCTCCTTCTGGCCCCCCTCCTGTTGCAGCTGATAGCCATGTCGACAGCAAGCA

CATGCATCCTTCAGGGGGGGTTTGAGTTACCAGGATTTATGTCCGAGGGAAACTTCACCA

TCGGAGGCATATTCCCCTTGCATTATAGAGTAGAGCTTCCACAGACGGACTTCAAAACAC

CTCCTGTGTCAGCCCATTGTAGAGGGTTTGACCCGCGTGCCTTCCGC

>Unigene97945_All

CAAAAATGGTGATGCTTTGCCCATCTATGACATCCTGAACTGGCATGGGATGCCAGATGG

TTCAATGAAAGCCAAAACAGTGGGTGATGTGGATGAATCACAACCAAGTGACAAAGTTCT

AAGTCTAGAAGAGGACAAAATCTTCTGGAACTTTAAATCTAAAAAAGTAATTTAGCCACA

TATTTTGTTGAGCCTAAAATGATGAATTATTAGATGTGATCTAAATACGGTTGTAGTGTC

AATAAACAAGTAAAATATTTTTTCATACTAACACTGATGAAAT

>Unigene61719_All GCTTCTCCATGAAGGGGCGTGTCCTAACATGTCCACAATACAGCCATGGCAGCTTCTGGA

GTACCTGAAACAGGTGAAGTTCACTAATGACTTTGGCGAGGAAATGAAATTTGATGAGAA

TGGCGACCCAGCTGCCATGTATGATCTGATCAACTGGCAGCTGACTGCAGATGGTGATGT

GAAATATGTCACAGTTGGAAAATTTGATGAAACCACTCGCAACAAACTGGAGATTGAAGA

TGAAGCCATACTGTGGACAGGGACAACTAAAGAAGTCCCTCTCTCAGTCTGCAGTAAAAG

CTGCCCCCCAGGCACCAGGAGAGCCATCAAGCCCAATTTCCCTGTTTGCTGTTTTGACTG

TATATTTTGCGCAGCTGGGGAGATTAGCAATCAGACAGATGCCATAGAGTGTGAACAATG

TCTACCTGAGTTCTGGTCCAACACATGGAGAAACTCCTGTGTTCCCAAACAGGTGGAGTT

TCTGTCTTATAGCGACACCATGGGCATCACCCTGATGGTAGTTGCCATTGTGGGATTCTG

CTGTACCCTCATGGTCGTCCTGATTTTTGCCTGCAATAAAAACACTCCCATCATCAGAGC

CAACAACTCAGAGCTGAGC

>Unigene78794_All

AACAACTCGGAGCTGAGCTTCCTGCTGCTGCTGTCCCTGGGGCTGTGCTTCCTCTGCACG

CTCACCTTCCTGGGCCGGCCCACATCCTGGGCCTGCCCCCTCCGCCGCACCTCCTTCGGG

CTCAGCTTCGCCCTCTGCCTCTCCTGCCTGCTCTGCAAGACCCTGGTGGTGCTCCTGGCC

TTCAAGGCCAAGCTGCCCGGCGACAAGACGGCGCGCTGGTTTCATCGGCCGCAGCAGCGT

CTGAGCGTGCTCGTCTGCTCCTCACTGCAGGTGCTTCTGTGCGTGGTGTGGCTGGCCAGG

GCGCCACCGTACCCCATCAGGAACACATGGCTCTACCGGGACAGGGTCATCCTGGAGTGC

CACATGGGTTCGGTGGCGCTCTTCAGCTGCGTACTGGGTTACATCGGGTGTCTCGCTGCT

TTCTGCTTGCTACTGGCCTTTTTGGCTCGCAAGCTGCCTGATAACTTCAACGAGGCC

>CL10924.Contig1_All

CCAGGTCAGGTATTACAGTATTTACATGGAGTGAATTTTACAATGCCTTCTGGAGAGACT

GTTTACTTTGACCAAAATGGAGATCCAGCTGCGAGATACGAGCTGGTAAACTGGCAGAAA

AATGCAGCAGGAGACACTGTATTTGTGACAGTTGGGAAATACGATGCCTCGTTACCTGAA

GGCAACCAGTTTGCCATGAATAGTCTCGATATTGTTTGGGCTGGAGAAACCAAGTCAGTA

GAAACCAACATCTGTGTGTAGTGAGAGCTGCCTCCCAGGCTATCGTCAGGCAGTAATTCG

AGGAAGGCCTGTGTGCTGTTTCTCCTGTGTGCGGTGCGCTGAAGGAGAGGTCAGCAGTAA

AGTGGATTCTACAGAATGCAGAAAGTGTCCTCTGGAGTTCTGGTCAAATGAAGACCACAG

CCGCTGCATTCCAAAACAAACAGAGTTCCTCTCTTTCACAGAAAACATGGGAATCATCTT

GACTACATTTTCACTCGTAGGAGCATGCTTCACCATTGCTGTGGCAGTTGTGTTCTTTTG

TTACATCGACACACCCTTAGTCAAAGCCAGCAATTTAGAGCTCAGTTTTCTGCTGTTGTT

CTCTCTCACCCTGTGTTTTCTTTGTTCGCTCACTTTTATTGGCCAGCCCTCGGACTGGTC

CTGCAGACTTAGGCACACAGCGTTTGGGATCACCTTT

>CL10924.Contig2_All

CAAAATGTCAAATTGACAAAAACTATAGCCTTCAATTTGACTGTTGTTGTGTACCAGAAA

CCAACATCTGTGTGTAGTGAGAGCTGCCTCCCAGGCTATCGTCAGGCAGTAATTCGAGGA

AGGCCTGTGTGCTGTTTCTCCTGTGTGCGGTGCGCTGAAGGAGAGGTCAGCAGTAAAGTG

GATTCTACAGAATGCAGAAAGTGTCCTCTGGAGTTCTGGTCAAATGAAGACCACAGCCGC

TGCATTCCAAAACAAACAGAGTTCCTCTCTTTCACAGAAAACATGGGAATCATCTTGACT

ACATTTTCACTCGTAGGAGCATGCTTCACCATTGCTGTGGCAGTTGTGTTCTTTTGTTAC

ATCGACACACCCTTAGTCAAAGCCAGCAATTTAGAGCTCAGTTTTCTGCTGTTGTTCTCT

CTCACCCTGTGTTTTCTTTGTTCGCTCACTTTTATTGGCCAGCCCTCGGACTGGTCCTGC

AGACTTAGGCACACAGCGTTTGGGATCACCTTT

>CL1147.Contig10_All TTTTGCCACATGTGCTTGTCTGAGTAACAGAAAAGAGTTTCCCTCCTTCTTCAGAACTAT

TCCCAGTGACTACTACCAGAGCAGGGCTCTGGCCAAGCTGGTGAAACACTTTGGCTGGAC

CTGGGTCGGAGCCATCAGGAGCAGGAGTGATTACGGAAACGGTGGAATGGCAACATTTCT

CAGTGCAGCTGAGAAAGAGGGTGTCTGTGTTGAATACTCAGTGGCCATCTACAGGACCGA

CCCTCGTGAAAAGTTTCTTGAGGTGGTCAACATCATCAAAAGGTCCACCTCTCGCGTGAT

TGTGGCTTTTGCTGATGGAAACGACCTAGATATTCTCCTCAAAGAACTCTTCCTCCAAAA

TGTCACTGGCTTTCAGTGGGTTGGCAGTGAAGGGTGGATCACATACAGGTATGTGGCCAC

ACAGACGAACTACGCCGTGCTCGGAGGGGCAGTAGGCTTTGCTGTGCCCAATGCAGTCAT

CCCCGGACTGCAGGAGTTTATGCTGGCTGCCCGACCCTCCCTGCAGCCTGGGAACCGGGG

GCTGGTGGAGATGTGGGAGAGTGTGTTTGACTGCAGCTTCGACCCCAACAGCCAAGCTAG

AGCTTGCACAGGGGAGGAGTCCCTGGGAGACACAGACACTCGCTTCACAGACGTATCAGA

TGCCAGTCTGCTCAACAACATTTACAAAGCTGTGTACGCTGTGGCATATGCACTAGATGA

ACTCCTTGCGTGTGAGGATGGACGAGGACAATTTGCAAATCAGTCCTGTGCAAACAAAAG

CAAAATTGAACCATGGCAGGTGTTACACTATCTGACCCTGGTGAATTTTACCACAAAGCA

AAGGGACAGTGTATCTTTTGATCACCAGGGTGACCCAGCTGCACGATATTCCCTGGTGAA

CTGGCAGAGGAACATGGTCGGCACTATCACGTTTGAGTCTATAGGTATTTACGACGCATC

TCTACAGGAGGGGCAGCAGTTCATGATGAAAAATGATGTCAGTGCTGTCTGGGCTGGAGG

CAAACAGGAGGTCCCCAGGTCAATCTGCAGCGAGAGCTGCCTGCCAGGCACTCGGAGGGC

GTTTTTGAAAGGAAAGCCCATCTGTTGCTTTGACTGCATTCGTTGTGCAGATGGTGAATT

CAGTAACACCACAAATGCAGTCACATGCTTGTCCTGTCCACCTGAATTCAAGTCAAATGA

AGAGAGAACTCAGTGTGATTTGAAAAATACTGAATACTTAACTTTCAAAGAGCTAATGGG

GATACTGCTTGTGACATTCTCTGTGTTTGGTGGGTGTTTAACTATCACAATAGGCTTGGT

ATTCTTCCAGTTCAGACAAACACCTATAGTTAGAGCCAACAACTCAGAGCTGAGTTTCCT

GCTGCTCTTCTCACTGACTCTGTGCTTCCTCTGTTCTCTTACTTTCATTGGTCGGCCCTC

TGAGTGGTCCTGTATGTTGCGTCACACAGCGTTTGGGATCACCTTTGTTCTCTGCATCTC

CTGTGTTCTGGGGAAAACAATAGTGGTGTTAATGGCCTTCAGAGCCACTCTTCCAGGCAG

TAATGTCATGAAGTGGTTTGGGCCTCTGCAACAGAGACTCAGTGTTCTTGGCTTCACTCT

CATACAAGTCCTTATATGTGTGCTCTGGTTAACTATATCCCCTCCTTTCCCTTACAGAAA

TATGCACCTTTACAATGATAAGATCATTCTAGAATGTGATGTGGGTTCAGCTATAGGATT

CTGGGCCATATTAGGTTACATTGGACTACTTGCTATCTTATGTTTCATACTGGCTTTTCT

GGCTCGTAAACTACCTGATAACTTTAATGAGGCCAAATTCATCACATTCAGTATGCTGAT

ATTCTGTGCAGTCTGGATCACTTTTATCCCGGCGTATGTCAGCTCTCCTGGGAAGTTCAC

TGTAGCTGTGGAAATATTTGCCATTTTAGCATCTAGTTTTGGTGTCCTTTTTTGCATATT

TGTGCCTAAATGTTACATTATCATATTTCTGCCAGATCATAACACAAAGAAGTTCCTTAT

GTCACAGAAGGGGGGGCGATGAGACTGTTAATGTTCTTAGTAACCTTATTGAAATGTGTA

AATATAGATAAATGAGAATTTAACAACATTGGACAGTTAACATTTTTACGTATAATGTGT

TCATGTATGTCTCTTTATGTTCTTTTCATGTGCTATTTTAGGTGTCTTGCTTGTAAAACA

ATAAAGACATTGGTCTGAGCGATACAATGTAATGACATAGAGTAATGGTACTGTATCTTA

CCTACTGAAGTGTAGGTAAGCACTCTGACCACCAGAGAGCGCTAAAGCTCCAGTGAATTC

TTTTAAACAACATTACACGACACTTGGAGGATGTGTCTTTTGTGCTCAAAGAAGAGTTCT

GTAAACAGTCAGTCTGCTTTCTTAAAACAGTTCACCCCTATTAGCAGGGGTGGAAATGTC

AACTTCAGAAAGTACAATCCCTGTCTCATATTTGATCCAACCACCTGTTCCTAATGAGCA

CACACATACTGTATCATACAGACAGATCAGCTAATAAGTGACATCACCTGTGCACTGTAC

ACAGAATTTTTTTTTTTTACTTTCACTAGCCACAATCTCTACCTCTGCCTACTAGCATCT

GGTTTTTTCCTTTTGTATATTTGTCTTCAAATGCCACATTTCACATTTTGTTCTTCCAGA

ACAACAACAAAAAAATCATGTTGCTAAAAGGGCATTGTTGAGACTCTAATTCTGCTTGTA

ACCTTATTGAAAATATGTGAATATGTCCCTCTTGTCATCAAAACTCTGTGTAAAAATGGC

AACAATAATGATTCACAATAAAACCGCTG

>CL1147.Contig11_All

GATATACTGCCAAATATTACGCTGGGTTATGAAATACATGATTCATGTGCAGCTGTGCCG

GTGGCTGTGAAAGTTGCTTTTCAGTTCGCTAACGGGCTGACCCCTCATGTTTCAATGAGC

GCATCTTGTCCCAAAACAGCCAACATTGTAGCTATTGTAGGGGAGTCTGGATCAACACCG

TCAATTGGCATGTCCAGAATTCTGGGTCTTTTCGGAATTCCACAGGTTAGCCACTTCGCC

ACCTGTGCCTGCCTGAGCGATAAGACGCAGCATCCCGCATTCTTCAGGACTATACCAAGC

GATCACTACCAAGCAGCTGCACTGGCACGACTGATCAAACATTTCGGATGGACGTGGATT

GGAGCAGTTCGCAGTGATTCTGACTACGGTAACAACGGTATGGCCTCCTTCCTGACAGCT

GCACAGAAAGAAGGAATCTGTGTCGAATATTCTGTGGCTTTCTACAGGACAAATTCTCGC

AGCAAGTTAGAGAGAGTGGCAGATGTAATACGCAGCTCTACCGCACGAGTTATCGTGGCT

TTTCTTGCCTCAGGTGACATGCGAATCTTACTGGAGGAGTTGACTCGACGTCCGCCTCCA

GCTTTACAGTGGATTGGAAGTGAGTCCTGGGTGACAGATCCAGATATGCTGAGATTTGAA

CTGTGTGCTGGAGCCATTGGTTTCGGAATCGAGCGTTCAGTAATCCCTGGTTTACGACAC

TTCCTTCTTGACCTCTCCCCTGCTCAGGCGTCCAAATCCGCAGTTCTCACTGAGCTGTGG

GAGAGCGCATTTGGCTGCTCCCTGCACACTGCTGAAGCAGGCCAAATCAGCGGGACTGTT

GCGCCATGCAAAGGGAATGAAAAACTGGATGCCGTTCAAATTCCCTACACTGACACTACG

CAGCTGCGCGTCACTAACATGGCTTACAAAGCAGCTTATGCCATAGCTCATGCTATCCAT

GGCCTTGTATGCAAAGACAGGTCAGACTCTGTATCCAAATGCAACAAAAGCACAGTCCTT

GAGCCAGATCAGATCCTTCAACAACTCAAAAAGGTCAATTTCAGTGTAAATGGAAACCAT

GTTTCTTTTGACTCTAATGGGGACCCTGTTGCCACATATGAGCTTGTGAACTGGCAAGCC

ATGCAAGATGGAAGTGTGCAGTTTGTGACAGTGGGCCGCTATGATGCATCACAACCCAAT

GGTCAGGAGTTTAGTATAAGCAAAAGCATCACATGGATGAAAGGACAAACACAGGTGCCA

GTGTCTGTGTGCAGTGAGAGCTGTCCCCCAGGCACCAGGAAGGCTGCACAGAAGGGGAAG

CCTGTGTGCTGCTATGACTGTATACCATGTGGAGAGGGAGAGATCAGCAATGAGACAGAT

TCAGTCGACTGTGTGTCATGTCCTGTTGACTACTGGCCCAATGCCCAGAGGAATATATGC

CTGCCTAAACCAGTAGAGTTCCTGTCATGGGATGAGGTTCTTGGCATTGTACTAGCAGTT

TGCTCTATTGCAGGCGCTTGTATTGCTGTCACGGTTGCTACTGTATTCTACAAACACAGG

ATGTCTCCTATTGTCAGAGCAAATAATTCTGAACTGAGCTTCCTGCTGCTCTTCTCACTG

ACTCTGTGCTTCCTCTGTTCTCTTACTTTCATTGGTCGGCCCTCTGAGTGGTCCTGTATG

TTGCGTCACACAGCGTTTGGGATCACCTTTGTTCTCTGCATCTCCTGTGTTCTGGGGAAA

ACAATAGTGGTGTTAATGGCCTTCAGAGCCACTCTTCCAGGCAGTAATGTCATGAAGTGG

TTTGGGCCTCTGCAACAGAGACTCAGTGTTCTTGGCTTCACTCTCATACAAGTCCTTATA

TGTGTGCTCTGGTTAACTATATCCCCTCCTTTCCCTTACAGAAATATGCACCTTTACAAT

GATAAGATCATTCTAGAATGTGATGTGGGTTCAGCTATAGGATTCTGGGCCATATTAGGT

TACATTGGACTACTTGCTATCTTATGTTTCATACTGGCTTTTCTGGCTCGTAAACTACCT

GATAACTTTAATGAGGCCAAATTCATCACATTCAGTATGCTGATATTCTGTGCAGTCTGG

ATCACTTTTATCCCGGCGTATGTCAGCTCTCCTGGGAAGTTCACTGTAGCTGTGGAAATA

TTTGCCATTTTAGCATCTAGTTTTGGTGTCCTTTTTTGCATATTTGTGCCTAAATGTTAC

ATTATCATATTTCTGCCAGATCATAACACAAAGAAGTTCCTTATGTCACAGAAGGGGGGG

CGATGAGACTGTTAATGTTCTTAGTAACCTTATTGAAATGTGTAAATATAGATAAATGAG

AATTTAACAACATTGGACAGTTAACATTTTTACGTATAATGTGTTCATGTATGTCTCTTT

ATGTTCTTTTCATGTGCTATTTTAGGTGTCTTGCTTGTAAAACAATAAAGACATTGGTCT

GAGCGATACAATGTAATGACATAGAGTAATGGTACTGTATCTTACCTACTGAAGTGTAGG

TAAGCACTCTGACCACCAGAGAGCGCTAAAGCTCCAGTGAATTCTTTTAAACAACATTAC

ACGACACTTGGAGGATGTGTCTTTTGTGCTCAAAGAAGAGTTCTGTAAACAGTCAGTCTG

CTTTCTTAAAACAGTTCACCCCTATTAGCAGGGGTGGAAATGTCAACTTCAGAAAGTACA

ATCCCTGTCTCATATTTGATCCAACCACCTGTTCCTAATGAGCACACACATACTGTATCA

TACAGACAGATCAGCTAATAAGTGACATCACCTGTGCACTGTACACAGAATTTTTTTTTT

TTACTTTCACTAGCCACAATCTCTACCTCTGCCTACTAGCATCTGGTTTTTTCCTTTTGT

ATATTTGTCTTCAAATGCCACATTTCACATTTTGTTCTTCCAGAACAACAACAAAAAAAT

CATGTTGCTAAAAGGGCATTGTTGAGACTCTAATTCTGCTTGTAACCTTATTGAAAATAT

GTGAATATGTCCCTCTTGTCATCAAAACTCTGTGTAAAAATGGCAACAATAATGATTCAC

AATAAAACCGCTG

>CL1147.Contig12_All

GATATACTGCCAAATATTACGCTGGGTTATGAAATACATGATTCATGTGCAGCTGTGCCG

GTGGCTGTGAAAGTTGCTTTTCAGTTCGCTAACGGGCTGACCCCTCATGTTTCAATGAGC

GCATCTTGTCCCAAAACAGCCAACATTGTAGCTATTGTAGGGGAGTCTGGATCAACACCG

TCAATTGGCATGTCCAGAATTCTGGGTCTTTTCGGAATTCCACAGGTGCTTTCAATTTGT

TGTGTACTTTACATATGATGATGACCTAATTCCTTTTGAAAAACACAAATGCTTTACATT

GTACTGGCGATGTCACTGACGATTTTCACAATCCAGATGTTAATAGGCTATCTCCTTTTA

CCTTTTTATACAAACAATCAAAAGGTTAGCCACTTCGCCACCTGTGCCTGCCTGAGCGAT

AAGACGCAGCATCCCGCATTCTTCAGGACTATACCAAGCGATCACTACCAAGCAGCTGCA

CTGGCACGACTGATCAAACATTTCGGATGGACGTGGATTGGAGCAGTTCGCAGTGATTCT

GACTACGGTAACAACGGTATGGCCTCCTTCCTGACAGCTGCACAGAAAGAAGGAATCTGT

GTCGAATATTCTGTGGCTTTCTACAGGACAAATTCTCGCAGCAAGTTAGAGAGAGTGGCA

GATGTAATACGCAGCTCTACCGCACGAGTTATCGTGGCTTTTCTTGCCTCAGGTGACATG

CGAATCTTACTGGAGGAGTTGACTCGACGTCCGCCTCCAGCTTTACAGTGGATTGGAAGT

GAGTCCTGGGTGACAGATCCAGATATGCTGAGATTTGAACTGTGTGCTGGAGCCATTGGT

TTCGGAATCGAGCGTTCAGTAATCCCTGGTTTACGACACTTCCTTCTTGACCTCTCCCCT

GCTCAGGCGTCCAAATCCGCAGTTCTCACTGAGCTGTGGGAGAGCGCATTTGGCTGCTCC

CTGCACACTGCTGAAGCAGGCCAAATCAGCGGGACTGTTGCGCCATGCAAAGGGAATGAA

AAACTGGATGCCGTTCAAATTCCCTACACTGACACTACGCAGCTGCGCGTCACTAACATG

GCTTACAAAGCAGCTTATGCCATAGCTCATGCTATCCATGGCCTTGTATGCAAAGACAGG

TCAGACTCTGTATCCAAATGCAACAAAAGCACAGTCCTTGAGCCAGATCAGATCCTTCAA

CAACTCAAAAAGGTCAATTTCAGTGTAAATGGAAACCATGTTTCTTTTGACTCTAATGGG

GACCCTGTTGCCACATATGAGCTTGTGAACTGGCAAGCCATGCAAGATGGAAGTGTGCAG

TTTGTGACAGTGGGCCGCTATGATGCATCACAACCCAATGGTCAGGAGTTTAGTATAAGC

AAAAGCATCACATGGATGAAAGGACAAACACAGGTGCCAGTGTCTGTGTGCAGTGAGAGC

TGTCCCCCAGGCACCAGGAAGGCTGCACAGAAGGGGAAGCCTGTGTGCTGCTATGACTGT

ATACCATGTGGAGAGGGAGAGATCAGCAATGAGACAGATTCAGTCGACTGTGTGTCATGT

CCTGTTGACTACTGGCCCAATGCCCAGAGGAATATATGCCTGCCTAAACCAGTAGAGTTC

CTGTCATGGGATGAGGTTCTTGGCATTGTACTAGCAGTTTGCTCTATTGCAGGCGCTTGT

ATTGCTGTCACGGTTGCTACTGTATTCTACAAACACAGGATGTCTCCTATTGTCAGAGCA

AATAATTCTGAACTGAGCTTCCTGCTGCTCTTCTCACTGACTCTGTGCTTCCTCTGTTCT

CTTACTTTCATTGGTCGGCCCTCTGAGTGGTCCTGTATGTTGCGTCACACAGCGTTTGGG

ATCACCTTTGTTCTCTGCATCTCCTGTGTTCTGGGGAAAACAATAGTGGTGTTAATGGCC

TTCAGAGCCACTCTTCCAGGCAGTAATGTCATGAAGTGGTTTGGGCCTCTGCAACAGAGA

CTCAGTGTTCTTGGCTTCACTCTCATACAAGTCCTTATATGTGTGCTCTGGTTAACTATA

TCCCCTCCTTTCCCTTACAGAAATATGCACCTTTACAATGATAAGATCATTCTAGAATGT

GATGTGGGTTCAGCTATAGGATTCTGGGCCATATTAGGTTACATTGGACTACTTGCTATC

TTATGTTTCATACTGGCTTTTCTGGCTCGTAAACTACCTGATAACTTTAATGAGGCCAAA

TTCATCACATTCAGTATGCTGATATTCTGTGCAGTCTGGATCACTTTTATCCCGGCGTAT

GTCAGCTCTCCTGGGAAGTTCACTGTAGCTGTGGAAATATTTGCCATTTTAGCATCTAGT

TTTGGTGTCCTTTTTTGCATATTTGTGCCTAAATGTTACATTATCATATTTCTGCCAGAT

CATAACACAAAGAAGTTCCTTATGTCACAGAAGGGGGGGCGATGAGACTGTTAATGTTCT

TAGTAACCTTATTGAAATGTGTAAATATAGATAAATGAGAATTTAACAACATTGGACAGT

TAACATTTTTACGTATAATGTGTTCATGTATGTCTCTTTATGTTCTTTTCATGTGCTATT

TTAGGTGTCTTGCTTGTAAAACAATAAAGACATTGGTCTGAGCGATACAATGTAATGACA

TAGAGTAATGGTACTGTATCTTACCTACTGAAGTGTAGGTAAGCACTCTGACCACCAGAG

AGCGCTAAAGCTCCAGTGAATTCTTTTAAACAACATTACACGACACTTGGAGGATGTGTC

TTTTGTGCTCAAAGAAGAGTTCTGTAAACAGTCAGTCTGCTTTCTTAAAACAGTTCACCC

CTATTAGCAGGGGTGGAAATGTCAACTTCAGAAAGTACAATCCCTGTCTCATATTTGATC

CAACCACCTGTTCCTAATGAGCACACACATACTGTATCATACAGACAGATCAGCTAATAA

GTGACATCACCTGTGCACTGTACACAGAATTTTTTTTTTTTACTTTCACTAGCCACAATC

TCTACCTCTGCCTACTAGCATCTGGTTTTTTCCTTTTGTATATTTGTCTTCAAATGCCAC

ATTTCACATTTTGTTCTTCCAGAACAACAACAAAAAAATCATGTTGCTAAAAGGGCATTG

TTGAGACTCTAATTCTGCTTGTAACCTTATTGAAAATATGTGAATATGTCCCTCTTGTCA

TCAAAACTCTGTGTAAAAATGGCAACAATAATGATTCACAATAAAACCGCTG

>CL1147.Contig13_All

TTTTGCCACATGTGCTTGTCTGAGTAACAGAAAAGAGTTTCCCTCCTTCTTCAGAACTAT

TCCCAGTGACTACTACCAGAGCAGGGCTCTGGCCAAGCTGGTGAAACACTTTGGCTGGAC

CTGGGTCGGAGCCATCAGGAGCAGGAGTGATTACGGAAACGGTGGAATGGCAACATTTCT

CAGTGCAGCTGAGAAAGAGGGTGTCTGTGTTGAATACTCAGTGGCCATCTACAGGACCGA

CCCTCGTGAAAAGTTTCTTGAGGTGGTCAACATCATCAAAAGGTCCACCTCTCGCGTGAT

TGTGGCTTTTGCTGATGGAAACGACCTAGATATTCTCCTCAAAGAACTCTTCCTCCAAAA

TGTCACTGGCTTTCAGTGGGTTGGCAGTGAAGGGTGGATCACATACAGGTATGTGGCCAC

ACAGACGAACTACGCCGTGCTCGGAGGGGCAGTAGGCTTTGCTGTGCCCAATGCAGTCAT

CCCCGGACTGCAGGAGTTTATGCTGGCTGCCCGACCCTCCCTGCAGCCTGGGAACCGGGG

GCTGGTGGAGATGTGGGAGAGTGTGTTTGACTGCAGCTTCGACCCCAACAGCCAAGCTAG

AGCTTGCACAGGGGAGGAGTCCCTGGGAGACACAGACACTCGCTTCACAGACGTATCAGA

TGCCAGTCTGCTCAACAACATTTACAAAGCTGTGTACGCTGTGGCATATGCACTAGATGA

ACTCCTTGCGTGTGAGGATGGACGAGGACAATTTGCAAATCAGTCCTGTGCAAACAAAAG

CAAAATTGAACCATGGCAGGTGTTACACTATCTGACCCTGGTGAATTTTACCACAAAGCA

AAGGGACAGTGTATCTTTTGATCACCAGGGTGACCCAGCTGCACGATATTCCCTGGTGAA

CTGGCAGAGGAACATGGTCGGCACTATCACGTTTGAGTCTATAGGTATTTACGACGCATC

TCTACAGGAGGGGCAGCAGTTCATGATGAAAAATGATGTCAGTGCTGTCTGGGCTGGAGG

CAAACAGGAGGTCCCCAGGTCAATCTGCAGCGAGAGCTGCCTGCCAGGCACTCGGAGGGC

GTTTTTGAAAGGAAAGCCCATCTGTTGCTTTGACTGCATTCGTTGTGCAGATGGTGAATT

CAGTAACACCACAAATGCAGTCACATGCTTGTCCTGTCCACCTGAATTCAAGTCAAATGA

AGAGAGAACTCAGTGTGATTTGAAAAATACTGAATACTTAACTTTCAAAGAGCTAATGGG

GATACTGCTTGTGACATTCTCTGTGTTTGGTGGGTGTTTAACTATCACAATAGGCTTGGT

ATTCTTCCAGTTCAGACAAACACCTATAGTTAGAGCCAACAACTCAGAGCTGAGTTTCCT

GCTGCTCTTCTCACTGACTCTGTGCTTCCTCTGTTCTCTTACTTTCATTGGTCGGCCCTC

TGAGTGGTCCTGTATGTTGCGTCACACAGCGTTTGGGATCACCTTTGTTCTCTGCATCTC

CTGTGTTCTGGGGAAAACAATAGTGGTGTTAATGGCCTTCAGAGCCACTCTTCCAGGCAG

TAATGTCATGAAGTGGTTTGGGCCTCTGCAACAGAGACTCAGTGTTCTTGGCTTCACTCT

CATACAAGTCCTTATATGTGTGCTCTGGTTAACTATATCCCCTCCTTTCCCTTACAGAAA

TATGCACCTTTACAATGATAAGATCATTCTAGAATGTGATGTGGGTTCAGCTATAGGATT

CTGGGCCATATTAGGTTACATTGGACTACTTGCTATCTTATGTTTCATACTGGCTTTTCT

GGCTCGTAAACTACCTGATAACTTTAATGAGGCCAAATTCATCACATTCAGTATGCTGAT

ATTCTGTGCAGTCTGGATCACTTTTATCCCGGCGTATGTCAGCTCTCCTGGGAAGTTCAC

TGTAGCTGTGGAAATATTTGCCATTTTAGCATCTAGTTTTGGATTACTTCTTTGCATTTT

CACCCCAAAATGTTACATTATTTTAATAAAGCCAGAGAAAAACACAAAGAAACAGTTAAT

GGGAAAGAAAGCACCAGGTTCCCTATAGGTGGCAATTAAGGAACTTCCGGCTCCCAAATA

TTTTAATCATTGTGAAATATTCATAATTAACTTCCATTGATGTGTATGTATACTTTGTAA

CAACATACCAAAAACAAGTTACATTTCATTAAAATACACATTGCTTTACTTCAACCCTCA

ATCGGATCACAAAATATGCACTCTTATGTGTTTATTAAACACTACTTTATCAAGCTACTT

TAAGTCTGATCACAATGGTATTACACAGGAGAGAATCCACAAAGGGCTCTGATCCACTAG

CTTTGCTCTCCACAGGCCCCAT

>CL1147.Contig14_All

CGGGCCAAGTAATGTGTCCCTGTGCTTTGCTGTTGCTGTTCTGGACCTGGCTAGCAAAGG

CAGAGAAGCCTCTTTGCCAACTGAGTGGGATTCCAGTGTTGCCTCTGCTGTCAAACAAAG

GGGATGTGATCATCGGAGGAGCATTTTCAATTCACAGCAAAATCACACAACCACAGTTAA

CTTTTACAGAACAACCAAAACCTATTTCATGCTCTAGTGTGAACCTCAGAGAATTCCGTT

TTGCCCAGACTATGATTTTCACCATTGAGGAAATCAACAGAAGTGGAGCGCTGCTTCCGA

ATGTCACCATCGGCTACAGGATCTATGACAACTGCGGCTCAACTCTGTCGTCGATGAGAG

CTGTGATGGCTCTGATGAATGGTCATGAACTCACAGCAGAGGACCCATGTACAGGGCAAT

CAGCTGTTCATGCCATTATTGGAGAGTCAGAGTCCTCCTCTACCATTGTGCTTTCCAGAA

CAACAGGGCCCTTCAACATACCTGTGATCAGCCACTCTGCTACTTGTGAGTGCCTAAGTA

GCAGGAGAGAGTTCCCTTCCTTCTTCAGGACCATTGCGAGTGACTACTACCAGAGCAGGG

CACTGGCTCAGCTGGTCAAACACTTTGGATGGACCTGGGTGGGAGCAGTAAACAGTGACA

GTGACTATGGCAACAATGGAATGGCAATATTTCTGGAAGCTGCTGAAGAGGAGGGCATAT

GCGTGGAATACTCTGAGAAATTTCACAGAACTGAGCCTGACAAATTACTAAAGGTTGTGG

ACGTTATCAGAGAGGGTTCGGCCAAAGTGATAGTAGCATTTCTTGCCCACGTTGAAATGA

ACAACCTGCTGGAACAGTTCACTCTCCAGAACATAACTGGCTTGCAAATGATTGGCGTTG

AGGCCTGGATTACTGCAGACAGCTTGATTACCCCATCAAGCTTCAGTATCTTAGGTGGGT

CACTTGGCTTTGCAGTGGGAAAAGCCAACTTAAGTGGCTTTGATGAATTTGTGAAACAGT

TTTGGGAGAAAACCTTCCAGTGCTCAGAGAACATGTCACAAGACGAGAATCAGTGTAGCA

GATATCAAGATCTTCTTGAGTGGAGGCACTATAATGACGATGTACCAGAGCTCAGATATT

CAAGCAACATCTACAAAGCAATCTATGCTGTGGCTTATTCAATGCACAGTCTTCTGAATT

GCAGGGCAAATGTTGGATGTGACAAAGTAATGCAAACTAAGCCCCAAAAGGTGGTCGAGT

CTCTTAAAACAGTAAATTTCACCCTGAAGACCAAGGAGCAGGTGTGGTTTGACAGCACAG

GTGCGGCAGCAGCCAGGTACGAGGTGGTGAACTGGCAGCGAGGGCCAGACGGAACAGTGC

GGTTTAAACCGGTGGGCCACTATGATGTCTCGCTGCCGGTGGGGCATCGCTTTGTCCTCA

GTGAGGACACTATAGTATGGCCAGGGGGCTCTGCCCAGAGGCCGGTGTCTGTGTGCAGTG

AGAGCTGTCCCCCAGGCACCAGGAAGGCTGTACAGAAAGGGAAGCCTGTGTGCTGCTATG

ACTGTGTCCCGTGTGGGGAAGGAGAGATTAGCAAAGAGACAGATTCTAATGACTGCATTC

CTTGTGATGTGGAGTACTGGTCAAATGAGAGCCCCAT

>CL1147.Contig2_All CGGGCCAAGTAATGTGTCCCTGTGCTTTGCTGTTGCTGTTCTGGACCTGGCTAGCAAAGG

CAGAGAAGCCTCTTTGCCAACTGAGTGGGATTCCAGTGTTGCCTCTGCTGTCAAACAAAG

GGGATGTGATCATCGGAGGAGCATTTTCAATTCACAGCAAAATCACACAACCACAGTTAA

CTTTTACAGAACAACCAAAACCTATTTCATGCTCTAGTGTGAACCTCAGAGAATTCCGTT

TTGCCCAGACTATGATTTTCACCATTGAGGAAATCAACAGAAGTGGAGCGCTGCTTCCGA

ATGTCACCATCGGCTACAGGATCTATGACAACTGCGGCTCAACTCTGTCGTCGATGAGAG

CTGTGATGGCTCTGATGAATGGTCATGAACTCACAGCAGAGGACCCATGTACAGGGCAAT

CAGCTGTTCATGCCATTATTGGAGAGTCAGAGTCCTCCTCTACCATTGTGCTTTCCAGAA

CAACAGGGCCCTTCAACATACCTGTGATCAGCCACTCTGCTACTTGTGAGTGCCTAAGTA

GCAGGAGAGAGTTCCCTTCCTTCTTCAGGACCATTGCGAGTGACTACTACCAGAGCAGGG

CACTGGCTCAGCTGGTCAAACACTTTGGATGGACCTGGGTGGGAGCAGTAAACAGTGACA

GTGACTATGGCAACAATGGAATGGCAATATTTCTGGAAGCTGCTGAAGAGGAGGGCATAT

GCGTGGAATACTCTGAGAAATTTCACAGAACTGAGCCTGACAAATTACTAAAGGTTGTGG

ACGTTATCAGAGAGGGTTCGGCCAAAGTGATAGTAGCATTTCTTGCCCACGTTGAAATGA

ACAACCTGCTGGAACAGTTCACTCTCCAGAACATAACTGGCTTGCAAATGATTGGCGTTG

AGGCCTGGATTACTGCAGACAGCTTGATTACCCCATCAAGCTTCAGTATCTTAGGTGGGT

CACTTGGCTTTGCAGTGGGAAAAGCCAACTTAAGTGGCTTTGATGAATTTGTGAAACAGT

TTTGGGAGAAAACCTTCCAGTGCTCAGAGAACATGTCACAAGACGAGAATCAGTGTAGCA

GATATCAAGATCTTCTTGAGTGGAGGCACTATAATGACGATGTACCAGAGCTCAGATATT

CAAGCAACATCTACAAAGCAATCTATGCTGTGGCTTATTCAATGCACAGTCTTCTGAATT

GCAGGGCAAATGTTGGATGTGACAAAGTAATGCAAACTAAGCCCCAAAAGGTGGTCGAGT

CTCTTAAAACAGTAAATTTCACCCTGAAGACCAAGGAGCAGGTGTGGTTTGACAGCACAG

GTGCGGCAGCAGCCAGGTACGAGGTGGTGAACTGGCAGCGAGGGCCAGACGGAACAGTGC

GGTTTAAACCGGTGGGCCACTATGATGTCTCGCTGCCGGTGGGGCATCGCTTTGTCCTCA

GTGAGGACACTATAGTATGGCCAGGGGGCTCTGCCCAGAGGCCGGTGTCTGTGTGCAGTG

AGAGCTGTCCCCCAGGCACCAGGAAGGCTGTACAGAAAGGGAAGCCTGTGTGCTGCTACC

AGTGTGTACCATGTGGGGCAGGGGAGATAAGTAACACCACAGATGCAACTGATTGTCTGA

AGTGTCCTCCGACATACTGGTCCAACTGGAAGGGTGACCATTGTCTCTTAAAGACCGTCG

AATTTCTATCCTACACAGAACTCATGGGGATCCTTTTAACATTGTTTTCCTTGTTGGGTG

CCACTCTGACCATATTGATTGCTGCAGTTTTCTGCCATTTCAGACACACACCCATAGTCA

AAGCCAACAACTCAGAGCTGAGCTTCCTGCTGCTCTTCTCACTGACTCTGTGCTTCCTCT

GTTCTCTTACTTTCATTGGTCGGCCCTCTGAGTGGTCCTGTATGTTGCGTCACACAGCGT

TTGGGATCACCTTTGTTCTCTGCATCTCCTGTGTTCTGGGGAAAACAATAGTGGTGTTAA

TGGCCTTCAGAGCCACTCTTCCAGGCAGTAATGTCATGAAGTGGTTTGGGCCTCTGCAAC

AGAGACTCAGTGTTCTTGGCTTCACTCTCATACAAGTCCTTATATGTGTGCTCTGGTTAA

CTATATCCCCTCCTTTCCCTTACAGAAATATGCACCTTTACAATGATAAGATCATTCTAG

AATGTGATGTGGGTTCAGCTATAGGATTCTGGGCCATATTAGGTTACATTGGACTACTTG

CTATCTTATGTTTCATACTGGCTTTTCTGGCTCGTAAACTACCTGATAACTTTAATGAGG

CCAAATTCATCACATTCAGTATGCTGATATTCTGTGCAGTCTGGATCACTTTTATCCCGG

CGTATGTCAGCTCTCCTGGTAAATTTACTGTGGCTGTGGAGATATTTGCCATTTTAGCAT

CTAGTTTTGGATTACTTCTTTGCATTTTCACCCCAAAATGTTACATTATTTTAATAAAGC

CAGAGAAAAACACAAAGAAACAGTTAATGGGAAAGAAAGCACCAGGTTCCCTATAGGTGG

CAATTAAGGAACTTCCGGCTCCCAAATATTTTAATCATTGTGAAATATTCATAATTAACT

TCCATTGATGTGTATGTATACTTTGTAACAACATACCAAAAACAAGTTACATTTCATTAA

AATACACATTGCTTTACTTCAACCCTCAATCGGATCACAAAATATGCACTCTTATGTGTT

TATTAAACACTACTTTATCAAGCTACTTTAAGTCTGATCACAATGGTATTACACAGGAGA

GAATCCACAAAGGGCTCTGATCCACTAGCTTTGCTCTCCACAGGCCCCATTCCAGGCTGT

GGTGTCCTCCAGGCCAGGCCCAGACACACAGGACAGCCCCTCTTCACATCTTTCTCATGC

ATGCTGTGGATATGCATTGTGTACTACAAAATAAAAAATAAGATTGCCTTTTCATATCTC

AGTTTCCAAAATTGATTATGTTGCTGCATGTTATGAAAACATAAATATCTATGGAAGTTT

GTATGAATGGCAGTGTAATGGGTTGTATGACTCTGTAAACATGATTTTCTAATATATATA

TTATTGTGAAATAAAGAGCTCCTTCCAGA

>CL1147.Contig3_All

CGGGCCAAGTAATGTGTCCCTGTGCTTTGCTGTTGCTGTTCTGGACCTGGCTAGCAAAGG

CAGAGAAGCCTCTTTGCCAACTGAGTGGGATTCCAGTGTTGCCTCTGCTGTCAAACAAAG

GGGATGTGATCATCGGAGGAGCATTTTCAATTCACAGCAAAATCACACAACCACAGTTAA

CTTTTACAGAACAACCAAAACCTATTTCATGCTCTAGTGTGAACCTCAGAGAATTCCGTT

TTGCCCAGACTATGATTTTCACCATTGAGGAAATCAACAGAAGTGGAGCGCTGCTTCCGA

ATGTCACCATCGGCTACAGGATCTATGACAACTGCGGCTCAACTCTGTCGTCGATGAGAG

CTGTGATGGCTCTGATGAATGGTCATGAACTCACAGCAGAGGACCCATGTACAGGGCAAT

CAGCTGTTCATGCCATTATTGGAGAGTCAGAGTCCTCCTCTACCATTGTGCTTTCCAGAA

CAACAGGGCCCTTCAACATACCTGTGATCAGCCACTCTGCTACTTGTGAGTGCCTAAGTA

GCAGGAGAGAGTTCCCTTCCTTCTTCAGGACCATTGCGAGTGACTACTACCAGAGCAGGG

CACTGGCTCAGCTGGTCAAACACTTTGGATGGACCTGGGTGGGAGCAGTAAACAGTGACA

GTGACTATGGCAACAATGGAATGGCAATATTTCTGGAAGCTGCTGAAGAGGAGGGCATAT

GCGTGGAATACTCTGAGAAATTTCACAGAACTGAGCCTGACAAATTACTAAAGGTTGTGG

ACGTTATCAGAGAGGGTTCGGCCAAAGTGATAGTAGCATTTCTTGCCCACGTTGAAATGA

ACAACCTGCTGGAACAGTTCACTCTCCAGAACATAACTGGCTTGCAAATGATTGGCGTTG

AGGCCTGGATTACTGCAGACAGCTTGATTACCCCATCAAGCTTCAGTATCTTAGGTGGGT

CACTTGGCTTTGCAGTGGGAAAAGCCAACTTAAGTGGCTTTGATGAATTTGTGAAACAGT

TTTGGGAGAAAACCTTCCAGTGCTCAGAGAACATGTCACAAGACGAGAATCAGTGTAGCA

GATATCAAGATCTTCTTGAGTGGAGGCACTATAATGACGATGTACCAGAGCTCAGATATT

CAAGCAACATCTACAAAGCAATCTATGCTGTGGCTTATTCAATGCACAGTCTTCTGAATT

GCAGGGCAAATGTTGGATGTGACAAAGTAATGCAAACTAAGCCCCAAAAGGTGGTCGAGT

CTCTTAAAACAGTAAATTTCACCCTGAAGACCAAGGAGCAGGTGTGGTTTGACAGCACAG

GTGCGGCAGCAGCCAGGTACGAGGTGGTGAACTGGCAGCGAGGGCCAGACGGAACAGTGC

GGTTTAAACCGGTGGGCCACTATGATGTCTCGCTGCCGGTGGGGCATCGCTTTGTCCTCA

GTGAGGACACTATAGTATGGCCAGGGGGCTCTGCCCAGAGGCCGGTGTCTGTGTGCAGTG

AGAGCTGTCCCCCAGGCACCAGGAAGGCTGTACAGAAAGGGAAGCCTGTGTGCTGCTATG

ACTGTATACCATGTGGGGCAGGGGAGATAAGTAACACCACAGATGCAACTGATTGTCTGA

AGTGTCCTCCGACATACTGGTCCAACTGGAAGGGTGACCATTGTCTCTTAAAGACCGTCG

AATTTCTATCCTACACAGAACTCATGGGGATCCTTTTAACATTGTTTTCCTTGTTGGGTG

CCACTCTGACCATATTGATTGCTGCAGTTTTCTGCCATTTCAGACACACACCCATAGTCA

AAGCCAACAACTCAGAGCTGAGCTTCCTGCTGCTCTTCTCACTGACTCTGTGCTTCCTCT

GTTCTCTTACTTTCATTGGTCGGCCCTCTGAGTGGTCCTGTATGTTGCGTCACACAGCGT

TTGGGATCACCTTTGTTCTCTGCATCTCCTGTGTTCTGGGGAAAACAATAGTGGTGTTAA

TGGCCTTCAGAGCCACTCTTCCAGGCAGTAATGTCATGAAGTGGTTTGGGCCTCTGCAAC

AGAGACTCAGTGTTCTTGGCTTCACTCTCATACAAGTCCTTATATGTGTGCTCTGGTTAA

CTATATCCCCTCCTTTCCCTTACAGAAATATGCACCTTTACAATGATAAGATCATTCTAG

AATGTGATGTGGGTTCAGCTATAGGATTCTGGGCCATATTAGGTTACATTGGACTACTTG

CTATCTTATGTTTCATACTGGCTTTTCTGGCTCGTAAACTACCTGATAACTTTAATGAGG

CCAAATTCATCACATTCAGTATGCTGATATTCTGTGCAGTCTGGATCACTTTTATCCCGG

CGTATGTCAGCTCTCCTGGGAAGTTCACTGTAGCTGTGGAAATATTTGCCATTTTAGCAT

CTAGTTTTGGTGTCCTTTTTTGCATATTTGTGCCTAAATGTTACATTATCATATTTCTGC

CAGATCATAACACAAAGAAGTTCCTTATGTCACAGAAGGGGGGGCGATGAGACTGTTAAT

GTTCTTAGTAACCTTATTGAAATGTGTAAATATAGATAAATGAGAATTTAACAACATTGG

ACAGTTAACATTTTTACGTATAATGTGTTCATGTATGTCTCTTTATGTTCTTTTCATGTG

CTATTTTAGGTGTCTTGCTTGTAAAACAATAAAGACATTGGTCTGAGCGATACAATGTAA

TGACATAGAGTAATGGTACTGTATCTTACCTACTGAAGTGTAGGTAAGCACTCTGACCAC

CAGAGAGCGCTAAAGCTCCAGTGAATTCTTTTAAACAACATTACACGACACTTGGAGGAT

GTGTCTTTTGTGCTCAAAGAAGAGTTCTGTAAACAGTCAGTCTGCTTTCTTAAAACAGTT

CACCCCTATTAGCAGGGGTGGAAATGTCAACTTCAGAAAGTACAATCCCTGTCTCATATT

TGATCCAACCACCTGTTCCTAATGAGCACACACATACTGTATCATACAGACAGATCAGCT

AATAAGTGACATCACCTGTGCACTGTACACAGAATTTTTTTTTTTTACTTTCACTAGCCA

CAATCTCTACCTCTGCCTACTAGCATCTGGTTTTTTCCTTTTGTATATTTGTCTTCAAAT

GCCACATTTCACATTTTGTTCTTCCAGAACAACAACAAAAAAATCATGTTGCTAAAAGGG

CATTGTTGAGACTCTAATTCTGCTTGTAACCTTATTGAAAATATGTGAATATGTCCCTCT

TGTCATCAAAACTCTGTGTAAAAATGGCAACAATAATGATTCACAATAAAACCGCTG

>CL1147.Contig4_All

CGGGCCAAGTAATGTGTCCCTGTGCTTTGCTGTTGCTGTTCTGGACCTGGCTAGCAAAGG

CAGAGAAGCCTCTTTGCCAACTGAGTGGGATTCCAGTGTTGCCTCTGCTGTCAAACAAAG

GGGATGTGATCATCGGAGGAGCATTTTCAATTCACAGCAAAATCACACAACCACAGTTAA

CTTTTACAGAACAACCAAAACCTATTTCATGCTCTAGTGTGAACCTCAGAGAATTCCGTT

TTGCCCAGACTATGATTTTCACCATTGAGGAAATCAACAGAAGTGGAGCGCTGCTTCCGA

ATGTCACCATCGGCTACAGGATCTATGACAACTGCGGCTCAACTCTGTCGTCGATGAGAG

CTGTGATGGCTCTGATGAATGGTCATGAACTCACAGCAGAGGACCCATGTACAGGGCAAT

CAGCTGTTCATGCCATTATTGGAGAGTCAGAGTCCTCCTCTACCATTGTGCTTTCCAGAA

CAACAGGGCCCTTCAACATACCTGTGATCAGCCACTCTGCTACTTGTGAGTGCCTAAGTA

GCAGGAGAGAGTTCCCTTCCTTCTTCAGGACCATTGCGAGTGACTACTACCAGAGCAGGG

CACTGGCTCAGCTGGTCAAACACTTTGGATGGACCTGGGTGGGAGCAGTAAACAGTGACA

GTGACTATGGCAACAATGGAATGGCAATATTTCTGGAAGCTGCTGAAGAGGAGGGCATAT

GCGTGGAATACTCTGAGAAATTTCACAGAACTGAGCCTGACAAATTACTAAAGGTTGTGG

ACGTTATCAGAGAGGGTTCGGCCAAAGTGATAGTAGCATTTCTTGCCCACGTTGAAATGA

ACAACCTGCTGGAACAGTTCACTCTCCAGAACATAACTGGCTTGCAAATGATTGGCGTTG

AGGCCTGGATTACTGCAGACAGCTTGATTACCCCATCAAGCTTCAGTATCTTAGGTGGGT

CACTTGGCTTTGCAGTGGGAAAAGCCAACTTAAGTGGCTTTGATGAATTTGTGAAACAGT

TTTGGGAGAAAACCTTCCAGTGCTCAGAGAACATGTCACAAGACGAGAATCAGTGTAGCA

GATATCAAGATCTTCTTGAGTGGAGGCACTATAATGACGATGTACCAGAGCTCAGATATT

CAAGCAACATCTACAAAGCAATCTATGCTGTGGCTTATTCAATGCACAGTCTTCTGAATT

GCAGGGCAAATGTTGGATGTGACAAAGTAATGCAAACTAAGCCCCAAAAGGTGGTCGAGT

CTCTTAAAACAGTAAATTTCACCCTGAAGACCAAGGAGCAGGTGTGGTTTGACAGCACAG

GTGCGGCAGCAGCCAGGTACGAGGTGGTGAACTGGCAGCGAGGGCCAGACGGAACAGTGC

GGTTTAAACCGGTGGGCCACTATGATGTCTCGCTGCCGGTGGGGCATCGCTTTGTCCTCA

GTGAGGACACTATAGTATGGCCAGGGGGCTCTGCCCAGAGGCCGGTGTCTGTGTGCAGTG

AGAGCTGTCCCCCAGGCACCAGGAAGGCTGTACAGAAAGGGAAGCCTGTGTGCTGCTATG

ACTGTATACCATGTGGAGAGGGGGAAATCAGCAATGAGACAGGTAACTCAGAGACAAAGT

ATACACTGCATAGAAAAGTGGCCTGTAGCGCCCTATGCAATTTCATAAAACTGTTCAAAA

TGCACTGTCTGAGCTCTGGTTTACACAACCCGTACCAATACTGCTGGATTGCATCTCTAA

TCTCGGTGACAATAATTCTCTTACAGATTCTAATAACTGCGAACCTTGCCCAGGACAGTA

CTGGCCAAACCCTGGCAGAGATAAATGCATATTAAAGGCTGTAGAGTTTCTCTTATTTAC

AGAAGTTATGGGAATAGTGTTAGTATTTTTCTCCCTCTTTGGTGTCTTGATAACTGTAAT

CACTGCCATTATTTTTATAATAAAGAAAGATACTCCAATAGTGAAAGCTAACAACTCAGA

GCTGAGCTTCCTGCTGCTCTTCTCACTGACTCTGTGCTTCCTCTGTTCTCTTACTTTCAT

TGGTCGGCCCTCTGAGTGGTCCTGTATGTTGCGTCACACAGCGTTTGGGATCACCTTTGT

TCTCTGCATCTCCTGTGTTCTGGGGAAAACAATAGTGGTGTTAATGGCCTTCAGAGCCAC

TCTTCCAGGCAGTAATGTCATGAAGTGGTTTGGGCCTCTGCAACAGAGACTCAGTGTTCT

TGGCTTCACTCTCATACAAGTCCTTATATGTGTGCTCTGGTTAACTATATCCCCTCCTTT

CCCTTACAGAAATATGCACCTTTACAATGATAAGATCATTCTAGAATGTGATGTGGGTTC

AGCTATAGGATTCTGGGCCATATTAGGTTACATTGGACTACTTGCTATCTTATGTTTCAT

ACTGGCTTTTCTGGCTCGTAAACTACCTGATAACTTTAATGAGGCCAAATTCATCACATT

CAGTATGCTGATATTCTGTGCAGTCTGGATCACTTTTATCCCGGCGTATGTCAGCTCTCC

TGGGAAGTTCACTGTAGCTGTGGAAATATTTGCCATTTTAGCATCTAGTTTTGGTGTCCT

TTTTTGCATATTTGTGCCTAAATGTTACATTATCATATTTCTGCCAGATCATAACACAAA

GAAGTTCCTTATGTCACAGAAGGGGGGGCGATGAGACTGTTAATGTTCTTAGTAACCTTA

TTGAAATGTGTAAATATAGATAAATGAGAATTTAACAACATTGGACAGTTAACATTTTTA

CGTATAATGTGTTCATGTATGTCTCTTTATGTTCTTTTCATGTGCTATTTTAGGTGTCTT

GCTTGTAAAACAATAAAGACATTGGTCTGAGCGATACAATGTAATGACATAGAGTAATGG

TACTGTATCTTACCTACTGAAGTGTAGGTAAGCACTCTGACCACCAGAGAGCGCTAAAGC

TCCAGTGAATTCTTTTAAACAACATTACACGACACTTGGAGGATGTGTCTTTTGTTGTGC

TCAAAGAAGAGTTCTGTAAACAGTCAGTCTGCTTTCTTAAAACAGTTCACCCCTATTAGC

AGGGGTGGAAATGTCAACTTCAGAAAGTACAATCCCTGTCTCATATTTGATCCAACCACC

TGTTCCTAATGAGCACACACATACTGTATCAGACAGACAGATCAGCTAATAAGTGACATC

ACCTGTGCACTGTACACAGATTTTTTTTTTTTTACTTTCACTAGCCACAATCTCTACCTC

TGCCTACTAGCATCTGGTTTTTTCCTTTTGTATATTTGTCTTCAAATGCCACATTTCACA

TTTTGTTCTTCCAGAACAACAACAAAAAAATCATGTTGCTAAAAGGGCATTGTTGAGACT

CTAATTCTGCTTGTAACCTTATTGAAAATATGTGAATATGTCCCTCTTGTCATCAAAACT

CTGTGTAAAAATGGCAACAATAATGATTCACAATAAAACCGCT

>CL1147.Contig6_All

GATATACTGCCAAATATTACGCTGGGTTATGAAATACATGATTCATGTGCAGCTGTGCCG

GTGGCTGTGAAAGTTGCTTTTCAGTTCGCTAACGGGCTGACCCCTCATGTTTCAATGAGC

GCATCTTGTCCCAAAACAGCCAACATTGTAGCTATTGTAGGGGAGTCTGGATCAACACCG

TCAATTGGCATGTCCAGAATTCTGGGTCTTTTCGGAATTCCACAGGTTAGCCACTTCGCC

ACCTGTGCCTGCCTGAGCGATAAGACGCAGCATCCCGCATTCTTCAGGACTATACCAAGC

GATCACTACCAAGCAGCTGCACTGGCACGACTGATCAAACATTTCGGATGGACGTGGATT

GGAGCAGTTCGCAGTGATTCTGACTACGGTAACAACGGTATGGCCTCCTTCCTGACAGCT

GCACAGAAAGAAGGAATCTGTGTCGAATATTCTGTGGCTTTCTACAGGACAAATTCTCGC

AGCAAGTTAGAGAGAGTGGCAGATGTAATACGCAGCTCTACCGCACGAGTTATCGTGGCT

TTTCTTGCCTCAGGTGACATGCGAATCTTACTGGAGGAGTTGACTCGACGTCCGCCTCCA

GCTTTACAGTGGATTGGAAGTGAGTCCTGGGTGACAGATCCAGATATGCTGAGATTTGAA

CTGTGTGCTGGAGCCATTGGTTTCGGAATCGAGCGTTCAGTAATCCCTGGTTTACGACAC

TTCCTTCTTGACCTCTCCCCTGCTCAGGCGTCCAAATCCGCAGTTCTCACTGAGCTGTGG

GAGAGCGCATTTGGCTGCTCCCTGCACACTGCTGAAGCAGGCCAAATCAGCGGGACTGTT

GCGCCATGCAAAGGGAATGAAAAACTGGATGCCGTTCAAATTCCCTACACTGACACTACG

CAGCTGCGCGTCACTAACATGGCTTACAAAGCAGCTTATGCCATAGCTCATGCTATCCAT

GGCCTTGTATGCAAAGACAGGTCAGACTCTGTATCCAAATGCAACAAAAGCACAGTCCTT

GAGCCAGATCAGATCCTTCAACAACTCAAAAAGGTCAATTTCAGTGTAAATGGAAACCAT

GTTTCTTTTGACTCTAATGGGGACCCTGTTGCCACATATGAGCTTGTGAACTGGCAAGCC

ATGCAAGATGGAAGTGTGCAGTTTGTGACAGTGGGCCGCTATGATGCATCACAACCCAAT

GGTCAGGAGTTTAGTATAAGCAAAAGCATCACATGGATGAAAGGACAAACACAGGTGCCA

GTGTCTGTGTGCAGTGAGAGCTGTCCCCCAGGCACCAGGAAGGCTGCACAGAAGGGGAAG

CCTGTGTGCTGCTATGACTGTATACCATGTGGAGAGGGAGAGATCAGCAATGAGACAGAT

TCAGTCGACTGTGTGTCATGTCCTGTTGACTACTGGCCCAATGCCCAGAGGAATATATGC

CTGCCTAAACCAGTAGAGTTCCTGTCATGGGATGAGGTTCTTGGCATTGTACTAGCAGTT

TGCTCTATTGCAGGCGCTTGTATTGCTGTCACGGTTGCTACTGTATTCTACAAACACAGG

ATGTCTCCTATTGTCAGAGCAAATAATTCTGAACTGAGCTTCCTGCTGCTCTTCTCACTG

ACTCTGTGCTTCCTCTGTTCTCTTACTTTCATTGGTCGGCCCTCTGAGTGGTCCTGTATG

TTGCGTCACACAGCGTTTGGGATCACCTTTGTTCTCTGCATCTCCTGTGTTCTGGGGAAA

ACAATAGTGGTGTTAATGGCCTTCAGAGCCACTCTTCCAGGCAGTAATGTCATGAAGTGG

TTTGGGCCTCTGCAACAGAGACTCAGTGTTCTTGGCTTCACTCTCATACAAGTCCTTATA

TGTGTGCTCTGGTTAACTATATCCCCTCCTTTCCCTTACAGAAATATGCACCTTTACAAT

GATAAGATCATTCTAGAATGTGATGTGGGTTCAGCTATAGGATTCTGGGCCATATTAGGT

TACATTGGACTACTTGCTATCTTATGTTTCATACTGGCTTTTCTGGCTCGTAAACTACCT

GATAACTTTAATGAGGCCAAATTCATCACATTCAGTATGCTGATATTCTGTGCAGTCTGG

ATCACTTTTATCCCGGCGTATGTCAGCTCTCCTGGGAAGTTCACTGTAGCTGTGGAAATA

TTTGCCATTTTAGCATCTAGTTTTGGATTACTTCTTTGCATTTTCACCCCAAAATGTTAC

ATTATTTTAATAAAGCCAGAGAAAAACACAAAGAAACAGTTAATGGGAAAGAAAGCACCA

GGTTCCCTATAGGTGGCAATTAAGGAACTTCCGGCTCCCAAATATTTTAATCATTGTGAA

ATATTCATAATTAACTTCCATTGATGTGTATGTATACTTTGTAACAACATACCAAAAACA

AGTTACATTTCATTAAAATACACATTGCTTTACTTCAACCCTCAATCGGATCACAAAATA

TGCACTCTTATGTGTTTATTAAACACTACTTTATCAAGCTACTTTAAGTCTGATCACAAT

GGTATTACACAGGAGAGAATCCACAAAGGGCTCTGATCCACTAGCTTTGCTCTCCACAGG

CCCCATTCCAGGCTGTGGTGTCCTCCAGGCCAGGCCCAGACACACAGGACAGCCCCTCTT

CACATCTTTCTCATGCATGCTGTGGATATGCATTGTGTACTACAAAATAAAAAATAAGAT

TGCCTTTTCATATCTCAGTTTCCAAAATTGATTATGTTGCTGCATGTTATGAAAACATAA

ATATCTATGGAAGTTTGTATGAATGGCAGTGTAATGGGTTGTATGACTCTGTAAACATGA

TTTTCTAATATATATATTATTGTGAAATAAAGAGCTCCTTCCAGA

>CL1147.Contig7_All

GATATACTGCCAAATATTACGCTGGGTTATGAAATACATGATTCATGTGCAGCTGTGCCG

GTGGCTGTGAAAGTTGCTTTTCAGTTCGCTAACGGGCTGACCCCTCATGTTTCAATGAGC

GCATCTTGTCCCAAAACAGCCAACATTGTAGCTATTGTAGGGGAGTCTGGATCAACACCG

TCAATTGGCATGTCCAGAATTCTGGGTCTTTTCGGAATTCCACAGGTGCTTTCAATTTGT

TGTGTACTTTACATATGATGATGACCTAATTCCTTTTGAAAAACACAAATGCTTTACATT

GTACTGGCGATGTCACTGACGATTTTCACAATCCAGATGTTAATAGGCTATCTCCTTTTA

CCTTTTTATACAAACAATCAAAAGGTTAGCCACTTCGCCACCTGTGCCTGCCTGAGCGAT

AAGACGCAGCATCCCGCATTCTTCAGGACTATACCAAGCGATCACTACCAAGCAGCTGCA

CTGGCACGACTGATCAAACATTTCGGATGGACGTGGATTGGAGCAGTTCGCAGTGATTCT

GACTACGGTAACAACGGTATGGCCTCCTTCCTGACAGCTGCACAGAAAGAAGGAATCTGT

GTCGAATATTCTGTGGCTTTCTACAGGACAAATTCTCGCAGCAAGTTAGAGAGAGTGGCA

GATGTAATACGCAGCTCTACCGCACGAGTTATCGTGGCTTTTCTTGCCTCAGGTGACATG

CGAATCTTACTGGAGGAGTTGACTCGACGTCCGCCTCCAGCTTTACAGTGGATTGGAAGT

GAGTCCTGGGTGACAGATCCAGATATGCTGAGATTTGAACTGTGTGCTGGAGCCATTGGT

TTCGGAATCGAGCGTTCAGTAATCCCTGGTTTACGACACTTCCTTCTTGACCTCTCCCCT

GCTCAGGCGTCCAAATCCGCAGTTCTCACTGAGCTGTGGGAGAGCGCATTTGGCTGCTCC

CTGCACACTGCTGAAGCAGGCCAAATCAGCGGGACTGTTGCGCCATGCAAAGGGAATGAA

AAACTGGATGCCGTTCAAATTCCCTACACTGACACTACGCAGCTGCGCGTCACTAACATG

GCTTACAAAGCAGCTTATGCCATAGCTCATGCTATCCATGGCCTTGTATGCAAAGACAGG

TCAGACTCTGTATCCAAATGCAACAAAAGCACAGTCCTTGAGCCAGATCAGATCCTTCAA

CAACTCAAAAAGGTCAATTTCAGTGTAAATGGAAACCATGTTTCTTTTGACTCTAATGGG

GACCCTGTTGCCACATATGAGCTTGTGAACTGGCAAGCCATGCAAGATGGAAGTGTGCAG

TTTGTGACAGTGGGCCGCTATGATGCATCACAACCCAATGGTCAGGAGTTTAGTATAAGC

AAAAGCATCACATGGATGAAAGGACAAACACAGGTGCCAGTGTCTGTGTGCAGTGAGAGC

TGTCCCCCAGGCACCAGGAAGGCTGCACAGAAGGGGAAGCCTGTGTGCTGCTATGACTGT

ATACCATGTGGAGAGGGAGAGATCAGCAATGAGACAGATTCAGTCGACTGTGTGTCATGT

CCTGTTGACTACTGGCCCAATGCCCAGAGGAATATATGCCTGCCTAAACCAGTAGAGTTC

CTGTCATGGGATGAGGTTCTTGGCATTGTACTAGCAGTTTGCTCTATTGCAGGCGCTTGT

ATTGCTGTCACGGTTGCTACTGTATTCTACAAACACAGGATGTCTCCTATTGTCAGAGCA

AATAATTCTGAACTGAGCTTCCTGCTGCTCTTCTCACTGACTCTGTGCTTCCTCTGTTCT

CTTACTTTCATTGGTCGGCCCTCTGAGTGGTCCTGTATGTTGCGTCACACAGCGTTTGGG

ATCACCTTTGTTCTCTGCATCTCCTGTGTTCTGGGGAAAACAATAGTGGTGTTAATGGCC

TTCAGAGCCACTCTTCCAGGCAGTAATGTCATGAAGTGGTTTGGGCCTCTGCAACAGAGA

CTCAGTGTTCTTGGCTTCACTCTCATACAAGTCCTTATATGTGTGCTCTGGTTAACTATA

TCCCCTCCTTTCCCTTACAGAAATATGCACCTTTACAATGATAAGATCATTCTAGAATGT

GATGTGGGTTCAGCTATAGGATTCTGGGCCATATTAGGTTACATTGGACTACTTGCTATC

TTATGTTTCATACTGGCTTTTCTGGCTCGTAAACTACCTGATAACTTTAATGAGGCCAAA

TTCATCACATTCAGTATGCTGATATTCTGTGCAGTCTGGATCACTTTTATCCCGGCGTAT

GTCAGCTCTCCTGGGAAGTTCACTGTAGCTGTGGAAATATTTGCCATTTTAGCATCTAGT

TTTGGATTACTTCTTTGCATTTTCACCCCAAAATGTTACATTATTTTAATAAAGCCAGAG

AAAAACACAAAGAAACAGTTAATGGGAAAGAAAGCACCAGGTTCCCTATAGGTGGCAATT

AAGGAACTTCCGGCTCCCAAATATTTTAATCATTGTGAAATATTCATAATTAACTTCCAT

TGATGTGTATGTATACTTTGTAACAACATACCAAAAACAAGTTACATTTCATTAAAATAC

ACATTGCTTTACTTCAACCCTCAATCGGATCACAAAATATGCACTCTTATGTGTTTATTA

AACACTACTTTATCAAGCTACTTTAAGTCTGATCACAATGGTATTACACAGGAGAGAATC

CACAAAGGGCTCTGATCCACTAGCTTTGCTCTCCACAGGCCCCATTCCAGGCTGTGGTGT

CCTCCAGGCCAGGCCCAGACACACAGGACAGCCCCTCTTCACATCTTTCTCATGCATGCT

GTGGATATGCATTGTGTACTACAAAATAAAAAATAAGATTGCCTTTTCATATCTCAGTTT

CCAAAATTGATTATGTTGCTGCATGTTATGAAAACATAAATATCTATGGAAGTTTGTATG

AATGGCAGTGTAATGGGTTGTATGACTCTGTAAACATGATTTTCTAATATATATATTATT

GTGAAATAAAGAGCTCCTTCCAGA

>CL1147.Contig8_All

TTTTGCCACATGTGCTTGTCTGAGTAACAGAAAAGAGTTTCCCTCCTTCTTCAGAACTAT

TCCCAGTGACTACTACCAGAGCAGGGCTCTGGCCAAGCTGGTGAAACACTTTGGCTGGAC

CTGGGTCGGAGCCATCAGGAGCAGGAGTGATTACGGAAACGGTGGAATGGCAACATTTCT

CAGTGCAGCTGAGAAAGAGGGTGTCTGTGTTGAATACTCAGTGGCCATCTACAGGACCGA

CCCTCGTGAAAAGTTTCTTGAGGTGGTCAACATCATCAAAAGGTCCACCTCTCGCGTGAT

TGTGGCTTTTGCTGATGGAAACGACCTAGATATTCTCCTCAAAGAACTCTTCCTCCAAAA

TGTCACTGGCTTTCAGTGGGTTGGCAGTGAAGGGTGGATCACATACAGGTATGTGGCCAC

ACAGACGAACTACGCCGTGCTCGGAGGGGCAGTAGGCTTTGCTGTGCCCAATGCAGTCAT

CCCCGGACTGCAGGAGTTTATGCTGGCTGCCCGACCCTCCCTGCAGCCTGGGAACCGGGG

GCTGGTGGAGATGTGGGAGAGTGTGTTTGACTGCAGCTTCGACCCCAACAGCCAAGCTAG

AGCTTGCACAGGGGAGGAGTCCCTGGGAGACACAGACACTCGCTTCACAGACGTATCAGA

TGCCAGTCTGCTCAACAACATTTACAAAGCTGTGTACGCTGTGGCATATGCACTAGATGA

ACTCCTTGCGTGTGAGGATGGACGAGGACAATTTGCAAATCAGTCCTGTGCAAACAAAAG

CAAAATTGAACCATGGCAGGTGTTACACTATCTGACCCTGGTGAATTTTACCACAAAGCA

AAGGGACAGTGTATCTTTTGATCACCAGGGTGACCCAGCTGCACGATATTCCCTGGTGAA

CTGGCAGAGGAACATGGTCGGCACTATCACGTTTGAGTCTATAGGTATTTACGACGCATC

TCTACAGGAGGGGCAGCAGTTCATGATGAAAAATGATGTCAGTGCTGTCTGGGCTGGAGG

CAAACAGGAGGTCCCCAGGTCAATCTGCAGCGAGAGCTGCCTGCCAGGCACTCGGAGGGC

GTTTTTGAAAGGAAAGCCCATCTGTTGCTTTGACTGCATTCGTTGTGCAGATGGTGAATT

CAGTAACACCACAAGTGAGACAGCAAAGCTCCTGTCCTTTATTCTAATGATGTTAATGTT

ACTGTGTGGAAAAGTCACACTTAGCACACTTGTCAGATACGTAAAAATGGCCACCCTTCC

AACATTCAATGTCTTTGTCCTTTCAGATGCAGTCACATGCTTGTCCTGTCCACCTGAATT

CAAGTCAAATGAAGAGAGAACTCAGTGTGATTTGAAAAATACTGAATACTTAACTTTCAA

AGAGCTAATGGGGATACTGCTTGTGACATTCTCTGTGTTTGGTGGGTGTTTAACTATCAC

AATAGGCTTGGTATTCTTCCAGTTCAGACAAACACCTATAGTTAGAGCCAACAACTCAGA

GCTGAGTTTCCTGCTGCTCTTCTCACTGACTCTGTGCTTCCTCTGTTCTCTTACTTTCAT

TGGTCGGCCCTCTGAGTGGTCCTGTATGTTGCGTCACACAGCGTTTGGGATCACCTTTGT

TCTCTGCATCTCCTGTGTTCTGGGGAAAACAATAGTGGTGTTAATGGCCTTCAGAGCCAC

TCTTCCAGGCAGTAATGTCATGAAGTGGTTTGGGCCTCTGCAACAGAGACTCAGTGTTCT

TGGCTTCACTCTCATACAAGTCCTTATATGTGTGCTCTGGTTAACTATATCCCCTCCTTT

CCCTTACAGAAATATGCACCTTTACAATGATAAGATCATTCTAGAATGTGATGTGGGTTC

AGCTATAGGATTCTGGGCCATATTAGGTTACATTGGACTACTTGCTATCTTATGTTTCAT

ACTGGCTTTTCTGGCTCGTAAACTACCTGATAACTTTAATGAGGCCAAATTCATCACATT

CAGTATGCTGATATTCTGTGCAGTCTGGATCACTTTTATCCCGGCGTATGTCAGCTCTCC

TGGGAAGTTCACTGTAGCTGTGGAAATATTTGCCATTTTAGCATGTAGTTTTGGTGTCCT

TTTTTGCATATTTGTGCCTAAATGTTACATTATCATATTTCTGCCAGATCATAACACAAA

GAAGTTCCTTATGTCACAGAAGGGGGGGCGATGAGACTGTTAATGTTCTTAGTAACCTTA

TTGAAATGTGTAAATATAGATAAATGAGAATTTAACAACATTGGACAGTTAACATTTTTA

CGTATAATGTGTTCATGTATGTCTCTTTATGTTCTTTTCATGTGCTATTTTAGGTGTCTT

GCTTGTAAAACAATAAAGACATTGGTCTGAGCGATACAATGTAATGACATAGAGTAATGG

TACTGTATCTTACCTACTGAAGTGTAGGTAAGCACTCTGACCACCAGAGAGCGCTAAAGC

TCCAGTGAATTCTTTTAAACAACATTACACGACACTTGGAGGATGTGTCTTTTGTGCTCA

AAGAAGAGTTCTGTAAACAGTCAGTCTGCTTTCTTAAAACAGTTCACCCCTATTAGCAGG

GGTGGAAATGTCAACTTCAGAAAGTACAATCCCTGTCTCATATTTGATCCAACCACCTGT

TCCTAATGAGCACACACATACTGTATCATACAGACAGATCAGCTAATAAGTGACATCACC

TGTGCACTGTACACAGAATTTTTTTTTTTTACTTTCACTAGCCACAATCTCTACCTCTGC

CTACTAGCATCTGGTTTTTTCCTTTTGTATATTTGTCTTCAAATGCCACATTTCACATTT

TGTTCTTCCAGAACAACAACAAAAAAATCATGTTGCTAAAAGGGCATTGTTGAGACTCTA

ATTCTGCTTGTAACCTTATTGAAAATATGTGAATATGTCCCTCTTGTCATCAAAACTCTG

TGTAAAAATGGCAACAATAATGATTCACAATAAAACCGCTG

>CL1147.Contig9_All

TTTTGCCACATGTGCTTGTCTGAGTAACAGAAAAGAGTTTCCCTCCTTCTTCAGAACTAT

TCCCAGTGACTACTACCAGAGCAGGGCTCTGGCCAAGCTGGTGAAACACTTTGGCTGGAC

CTGGGTCGGAGCCATCAGGAGCAGGAGTGATTACGGAAACGGTGGAATGGCAACATTTCT

CAGTGCAGCTGAGAAAGAGGGTGTCTGTGTTGAATACTCAGTGGCCATCTACAGGACCGA

CCCTCGTGAAAAGTTTCTTGAGGTGGTCAACATCATCAAAAGGTCCACCTCTCGCGTGAT

TGTGGCTTTTGCTGATGGAAACGACCTAGATATTCTCCTCAAAGAACTCTTCCTCCAAAA

TGTCACTGGCTTTCAGTGGGTTGGCAGTGAAGGGTGGATCACATACAGGTATGTGGCCAC

ACAGACGAACTACGCCGTGCTCGGAGGGGCAGTAGGCTTTGCTGTGCCCAATGCAGTCAT

CCCCGGACTGCAGGAGTTTATGCTGGCTGCCCGACCCTCCCTGCAGCCTGGGAACCGGGG

GCTGGTGGAGATGTGGGAGAGTGTGTTTGACTGCAGCTTCGACCCCAACAGCCAAGCTAG

AGCTTGCACAGGGGAGGAGTCCCTGGGAGACACAGACACTCGCTTCACAGACGTATCAGA

TGCCAGTCTGCTCAACAACATTTACAAAGCTGTGTACGCTGTGGCATATGCACTAGATGA

ACTCCTTGCGTGTGAGGATGGACGAGGACAATTTGCAAATCAGTCCTGTGCAAACAAAAG

CAAAATTGAACCATGGCAGGTGTTACACTATCTGACCCTGGTGAATTTTACCACAAAGCA

AAGGGACAGTGTATCTTTTGATCACCAGGGTGACCCAGCTGCACGATATTCCCTGGTGAA

CTGGCAGAGGAACATGGTCGGCACTATCACGTTTGAGTCTATAGGTATTTACGACGCATC

TCTACAGGAGGGGCAGCAGTTCATGATGAAAAATGATGTCAGTGCTGTCTGGGCTGGAGG

CAAACAGGAGGTCCCCAGGTCAATCTGCAGCGAGAGCTGCCTGCCAGGCACTCGGAGGGC

GTTTTTGAAAGGAAAGCCCATCTGTTGCTTTGACTGCATTCGTTGTGCAGATGGTGAATT

CAGTAACACCACAAGTGAGACAGCAAAGCTCCTGTCCTTTATTCTAATGATGTTAATGTT

ACTGTGTGGAAAAGTCACACTTAGCACACTTGTCAGATACGTAAAAATGGCCACCCTTCC

AACATTCAATGTCTTTGTCCTTTCAGATGCAGTCACATGCTTGTCCTGTCCACCTGAATT

CAAGTCAAATGAAGAGAGAACTCAGTGTGATTTGAAAAATACTGAATACTTAACTTTCAA

AGAGCTAATGGGGATACTGCTTGTGACATTCTCTGTGTTTGGTGGGTGTTTAACTATCAC

AATAGGCTTGGTATTCTTCCAGTTCAGACAAACACCTATAGTTAGAGCCAACAACTCAGA

GCTGAGTTTCCTGCTGCTCTTCTCACTGACTCTGTGCTTCCTCTGTTCTCTTACTTTCAT

TGGTCGGCCCTCTGAGTGGTCCTGTATGTTGCGTCACACAGCGTTTGGGATCACCTTTGT

TCTCTGCATCTCCTGTGTTCTGGGGAAAACAATAGTGGTGTTAATGGCCTTCAGAGCCAC

TCTTCCAGGCAGTAATGTCATGAAGTGGTTTGGGCCTCTGCAACAGAGACTCAGTGTTCT

TGGCTTCACTCTCATACAAGTCCTTATATGTGTGCTCTGGTTAACTATATCCCCTCCTTT

CCCTTACAGAAATATGCACCTTTACAATGATAAGATCATTCTAGAATGTGATGTGGGTTC

AGCTATAGGATTCTGGGCCATATTAGGTTACATTGGACTACTTGCTATCTTATGTTTCAT

ACTGGCTTTTCTGGCTCGTAAACTACCTGATAACTTTAATGAGGCCAAATTCATCACATT

CAGTATGCTGATATTCTGTGCAGTCTGGATCACTTTTATCCCGGCGTATGTCAGCTCTCC

TGGGAAGTTCACTGTAGCTGTGGAAATATTTGCCATTTTAGCATCTAGTTTTGGATTACT

TCTTTGCATTTTCACCCCAAAATGTTACATTATTTTAATAAAGCCAGAGAAAAACACAAA

GAAACAGTTAATGGGAAAGAAAGCACCAGGTTCCCTATAGGTGGCAATTAAGGAACTTCC

GGCTCCCAAATATTTTAATCATTGTGAAATATTCATAATTAACTTCCATTGATGTGTATG

TATACTTTGTAACAACATACCAAAAACAAGTTACATTTCATTAAAATACACATTGCTTTA

CTTCAACCCTCAATCGGATCACAAAATATGCACTCTTATGTGTTTATTAAACACTACTTT

ATCAAGCTACTTTAAGTCTGATCACAATGGTATTACACAGGAGAGAATCCACAAAGGGCT

CTGATCCACTAGCTTTGCTCTCCACAGGCCCCATTCCAGGCTGTGGTGTCCTCCAGGCCA

GGCCCAGACACACAGGACAGCCCCTCTTCACATCTTTCTCATGCATGCTGTGGATATGCA

TTGTGTACTACAAAATAAAAAATAAGATTGCCTTTTCATATCTCAGTTTCCAAAATTGAT

TATGTTGCTGCATGTTATGAAAACATAAATATCTATGGAAGTTTGTATGAATGGCAGTGT

AATGGGTTGTATGACTCTGTAAACATGATTTTCTAATATATATATTATTGTGAAATAAAG

AGCTCCTTCCAGA

>CL5324.Contig1_All

AAAACTGCTTCCCTGTCCTCCTTCCTCTGTCATTGAAGGAGACTGTAGTGTTGTCAGAGG

ATGACTCCTCACCAACTTCTTCAATCCTTCGTACACCTTCTCTCTGCCATTCTCTCTCCT

GCCTTGGCATCAGCCACACCATGTGCTTTGCAATCACATTTTGAGGCAGGCTTCAAAGCC

GAGGGAGATTTTATTATCGGAGGCATATTTCCCCTGCACTACAATATGGAGATGCCAGAT

CTAAACTGCACCTACAAGCCTGCACCAGTGCAGTGTAATGGGTTTGACCCGCGTGCCTTC

CGCTGGGCCTTGACCATGAGACTGGCAGTGGAGGAGATCAACAGGAGCAAAGGCTTACTA

CCGAACCACACTCTGGGCTATAAGATTTTGGACTCCTGTGCGTACCCTTTAACGGGCCAG

CGGGCCGTGTTAGCCATACTAAATGGCCCCGGTGAGGTTGGCAGTCCGCTCTGCTCAGGT

GCTGGTCCCTTAGTAGCTGTTATTGGAGAGTCAGGCTCTGCCCAGTCAATAGTGGTATCC

AGGATTCTGCAACCTTTTAGAATACCAATGATCAGCTACTTCTCCTCATGTGCCTGTCTG

GGTGATAGAAAAGAGTTCCCTACTTTCTTCAGAGTCATCCCTAGTGATGACTACCAGGTA

AAAGCTATTGCCCAGCTACTGCAACACTTCAACTGGACGTGGGTGGGTGTGGTGCGCGGG

GATCATGAGTATGGCCGCTTTGCCCTGCAGGGGCTCCTGAGAGAGCTGGAAGGCACAGGT

GTGTGTGTGGCCTACCAGGAAATGATTCCTCTGCTGTATGACAGCCAGAGAGCACTGGAG

ATCATCCACGTCATGAGGACCTCCAGTGCACGGGTGGTGGTGGTCTTCTCCGCTGAGGGT

GAGCTCACCCCCTTCCTGAGAGACTACATTGAGCAGAATGTCACTGGCATCCAGTGGATC

GCCAGCGAAGCATGGGTGACATCCTCCGTGTTCACAGGAAAGGAGTATTATCCCTTTCTG

GGGGGCACCATCGGATTTGGCATACGACAAGGCCAGATCCCAGCACTCAGAGATTATCTG

AACACAGTGGACCCGTGGCGTTACCCCACTAATCGCCTCGTACGTGAGCTATGGGAGACC

CTTTATGGCTGCTCTCCCAATAACACAGCCAGAAATACGCAGTTCCCAGTCTGCGTGGGG

TACGAGTCTCTGCGTGAGCAGCACTCAGCTTACATGAACACCTCAAGCCCTCGGATTTCC

TACAATGTGTACAAAGGTGTATATGCCGTCGCTCATTCGCTGCACAACCTTATGCTCTGT

AAAAATGGACAGGGACCATTTAAAAACTCTTCATGTGCTGACCTCAACAACATCTATCCA

TGGCAGGAAGCTGGGAAAGAGCTACTCATAGAGGACAAAATGATCGTGTGGGCTGGTCAT

CGGAGCAAGGCAAGCTGCTCACAGTGTGTATTTGAATACATGAGGTGCCATCTGTTGAAG

TTGTTTAGGGTCAGGTGGAGGTGCTGGTGTCTGTGTGTAGTGACAGCTGTCCCCCAGGAT

CCCGGAAGGCTGTCCGTGCTGGGGAGCCTATTTGCTGCTTTGACTGTGTACCATGTGACA

GCGGCAAAATTAGCAATGAGACAGATTCAATAGACTGTATGGCCTGTCCTGAAGACTTCT

GGTCCAATACTGATGGAACACACTGTGTACCCAAGGTGGTTGAGTTTCTTTCCCATGATG

CCATGGGAGTGACTCTGACAGTGATAGCCATTGTAGGGGCCTGCCTCACGGTTTCTGTTC

TAGCAGTGTTCCTCTACCACAGAAGCACCCCCATAGTCCGTGTCAACAACTCAGAGCTGA

GTTTCTTCATCCTGCTGTCTCTGACTCTGTGTTTCCTGTGTGCGCTGGTGTTCATCGGAG

AGCCCACATCCTGGTCCTGCATGCTGCGCCACACGGCCTTCAGCATCACCTTCTCCCTCT

GCATCTCCTGCATCCTGGGAAAGACTTTGGTGGTGCTGGCAGCTTTCACAGCCACGCGGC

CTGGAAACAACATCATGAAGTGGCTGGGGCCCAGGCAGCAGAGGGTCATCATCTTCTCCT

GCACTCTGGTACAGGTGGTAATCTGTGCTGCCTGGCTGATATCTGCTCCCCCATTTCCCT

CTAGAAACACTCAGTACCAGCGCTCCAAAATCATTCTGGAGTGTAGTGTGGGGTCTGACT

TGGCCTTCTGGTGTGTTCTGGGATATATTGGGCTTCTTGCTTGCCTGTGTTTTGTGTTGG

CCTTTTTGGCCCGGAAACTTCCAGGAAATTTCAATGAGGCTAAATACATTACTTTTAGTA

TGCTGATTTTCTGTGCAGTCTGGTTAGCTTTCATCCCAGCCTATGTCAGCTCACCAGGAA

AGTTCACCGTAGCTGTGGAGATTTTTGCCATTTTGGCTTCCAGTTTTGGCCTGCTATTGT

GCTTATTTGCACCAAAGTGTTACATTATTTTGATAAGACCAGAGAAAAACACCAAAAATC

ACTTAATGGGAAAAGAAAAGTAAACAAATAAGTAATTAGATAACATGATCTAGAACTGAA

AAAAATGAGCAAACAAACTTTACTTTTAAGAATAAAATGTTTTACCAGTGTAGTTTTTTG

GTCTTTCCCCAATGATCAGAAGACAGGCAAAAAGACTACCCAGTCATCTGCCATAAAATT

AGCTTTAATAATTCTCTCTCTCTCTCTCTCTCT

>CL5324.Contig2_All

AAAACTGCTTCCCTGTCCTCCTTCCTCTGTCATTGAAGGAGACTGTAGTGTTGTCAGAGG

ATGACTCCTCACCAACTTCTTCAATCCTTCGTACACCTTCTCTCTGCCATTCTCTCTCCT

GCCTTGGCATCAGCCACACCATGTGCTTTGCAATCACATTTTGAGGCAGGCTTCAAAGCC

GAGGGAGATTTTATTATCGGAGGCATATTTCCCCTGCACTACAATATGGAGATGCCAGAT

CTAAACTGCACCTACAAGCCTGCACCAGTGCAGTGTAATGGGTTTGACCCGCGTGCCTTC

CGCTGGGCCTTGACCATGAGACTGGCAGTGGAGGAGATCAACAGGAGCAAAGGCTTACTA

CCGAACCACACTCTGGGCTATAAGATTTTGGACTCCTGTGCGTACCCTTTAACGGGCCAG

CGGGCCGTGTTAGCCATACTAAATGGCCCCGGTGAGGTTGGCAGTCCGCTCTGCTCAGGT

GCTGGTCCCTTAGTAGCTGTTATTGGAGAGTCAGGCTCTGCCCAGTCAATAGTGGTATCC

AGGATTCTGCAACCTTTTAGAATACCAATGATCAGCTACTTCTCCTCATGTGCCTGTCTG

GGTGATAGAAAAGAGTTCCCTACTTTCTTCAGAGTCATCCCTAGTGATGACTACCAGGTA

AAAGCTATTGCCCAGCTACTGCAACACTTCAACTGGACGTGGGTGGGTGTGGTGCGCGGG

GATCATGAGTATGGCCGCTTTGCCCTGCAGGGGCTCCTGAGAGAGCTGGAAGGCACAGGT

GTGTGTGTGGCCTACCAGGAAATGATTCCTCTGCTGTATGACAGCCAGAGAGCACTGGAG

ATCATCCACGTCATGAGGACCTCCAGTGCACGGGTGGTGGTGGTCTTCTCCGCTGAGGGT

GAGCTCACCCCCTTCCTGAGAGACTACATTGAGCAGAATGTCACTGGCATCCAGTGGATC

GCCAGCGAAGCATGGGTGACATCCTCCGTGTTCACAGGAAAGGAGTATTATCCCTTTCTG

GGGGGCACCATCGGATTTGGCATACGACAAGGCCAGATCCCAGCACTCAGAGATTATCTG

AACACAGTGGACCCGTGGCGTTACCCCACTAATCGCCTCGTACGTGAGCTATGGGAGACC

CTTTATGGCTGCTCTCCCAATAACACAGCCAGAAATACGCAGTTCCCAGTCTGCGTGGGG

TACGAGTCTCTGCGTGAGCAGCACTCAGCTTACATGAACACCTCAAGCCCTCGGATTTCC

TACAATGTGTACAAAGGTGTATATGCCGTCGCTCATTCGCTGCACAACCTTATGCTCTGT

AAAAATGGACAGGGACCATTTAAAAACTCTTCATGTGCTGACCTCAACAACATCTATCCA

TGGCAGCTCCAGCACTACCTTCAAGAGGTTTCCTTCACTATATCAGGAGAGGAAGTTAAC

TTTGATGACAAAGGGGACTCAATTCCATCGTATGACCTTATTAACTGGCAAAGAGGCCAC

GAGGGCAATATTGAGTTCATTAATGTTGGGCTGTTTGATGCCGCACAGGAAGCTGGGAAA

GAGCTACTCATAGAGGACAAAATGATCGTGTGGGCTGGTCATCGGAGCAAGGCAAGCTGC

TCACAGTGTGTATTTGAATACATGAGGTGCCATCTGTTGAAGTTGTTTAGGGTCAGGTGG

AGGTGCTGGTGTCTGTGTGTAGTGACAGCTGTCCCCCAGGATCCCGGAAGGCTGTCCGTG

CTGGGGAGCCTATTTGCTGCTTTGACTGTGTACCATGTGACAGCGGCAAAATTAGCAATG

AGACAGATTCAATAGACTGTATGGCCTGTCCTGAAGACTTCTGGTCCAATACTGATGGAA

CACACTGTGTACCCAAGGTGGTTGAGTTTCTTTCCCATGATGCCATGGGAGTGACTCTGA

CAGTGATAGCCATTGTAGGGGCCTGCCTCACGGTTTCTGTTCTAGCAGTGTTCCTCTACC

ACAGAAGCACCCCCATAGTCCGTGTCAACAACTCAGAGCTGAGTTTCTTCATCCTGCTGT

CTCTGACTCTGTGTTTCCTGTGTGCGCTGGTGTTCATCGGAGAGCCCACATCCTGGTCCT

GCATGCTGCGCCACACGGCCTTCAGCATCACCTTCTCCCTCTGCATCTCCTGCATCCTGG

GAAAGACTTTGGTGGTGCTGGCAGCTTTCACAGCCACGCGGCCTGGAAACAACATCATGA

AGTGGCTGGGGCCCAGGCAGCAGAGGGTCATCATCTTCTCCTGCACTCTGGTACAGGTGG

TAATCTGTGCTGCCTGGCTGATATCTGCTCCCCCATTTCCCTCTAGAAACACTCAGTACC

AGCGCTCCAAAATCATTCTGGAGTGTAGTGTGGGGTCTGACTTGGCCTTCTGGTGTGTTC

TGGGATATATTGGGCTTCTTGCTTGCCTGTGTTTTGTGTTGGCCTTTTTGGCCCGGAAAC

TTCCAGGAAATTTCAATGAGGCTAAATACATTACTTTTAGTATGCTGATTTTCTGTGCAG

TCTGGTTAGCTTTCATCCCAGCCTATGTCAGCTCACCAGGAAAGTTCACCGTAGCTGTGG

AGATTTTTGCCATTTTGGCTTCCAGTTTTGGCCTGCTATTGTGCTTATTTGCACCAAAGT

GTTACATTATTTTGATAAGACCAGAGAAAAACACCAAAAATCACTTAATGGGAAAAGAAA

AGTAAACAAATAAGTAATTAGATAACATGATCTAGAACTGAAAAAAATGAGCAAACAAAC

TTTACTTTTAAGAATAAAATGTTTTACCAGTGTAGTTTTTTGGTCTTTCCCCAATGATCA

GAAGACAGGCAAAAAGACTACCCAGTCATCTGCCATAAAATTAGCTTTAATAATTCTCTC

TCTCTCTCTCTCTCT

>CL6778.Contig1_All

TCCTCCTCCTCCTCCTCCAGGGCAGAGAGGCCCCTGTGCACAGGAGCAGAGGACATCCAG

GAGAAGGCGAATGCGTATTCAGATGTGACTCAGCTGGGAGTCAGCTATAATGTGTACAAG

GCTGTGTATGCTGTTGCCCATGCCATTCAAGACATGCTCGCCTGCCAACCAGGGCAGGGG

CCATTTGAAAATGGAAAGTGTCCAAGCATCAATAACATTATACCTAAACAACTACTTCAC

TACCTGGAGAGAGTGAATTTCACAACCCCTCTGGGTGACATTGTGAACTTTGATATGAAT

GGAGACCCTCCTGCATCGTACGATCTGATTAACTGGCACATCGGTGACAAAGGCATGGCT

GAATTTGTGAAGGTGGGGCAATATGACTCAAACATTGGCCCAGATCAGAGACTCCAACTG

GACCTCAATAAAGTTGTGTGGGGCGGAGGCTGGACCGACAAGGTACCTGTGTCTGTGTGC

AGCATTCCTTGTGCCCCAGGCACCTGGAAAGCTCTGCAAAAGGGGAAACCTGTCTGTTGT

TTTGACTGTATACCTTGCCCAGATGGAGAAATCAGTAATAAAACAGGTGCAACTGAATGC

ACGAGATGTCCAGAGCAGTTCTGGTCCAACAACCTGAGGACCCAGTGCATCCTGAAGCGA

GAGGAATTCCTGTCTTTCTATGAGCCCCTGGGCATCATCCTGACGGTGCTGTCGATATCC

GGGGCAATGCTCACGACCGTTGTCCTGGTGACATTCATCCTCCACAGAGACACGCCTCTC

GTCCGCGCCAACAACTCGGAGCTGAGCTTCCTGTTGCTGCTGTCCCTCATCCTGTGCTTC

CTGTGTGCCATGTCCTTCATGGGTCGGCCCCTCGCCTGGTCCTGCATGTTGAGACACACC

CTCTTCGGGATAAGTTTTGTGGTGTGCATCTCCTGCATCCTCAGCAAGACGGTGGTGGTC

CTGGTGGCCTTCCGCGCCACTCTGCCGGGCAGTAAAGTGATGCGCTACTTCGGGCCACTG

CAGCAAAGGCTGGGCATCTGCCTGTGCACCCTGGTGCAGGTGCTGGTGTGTGTGCTGTGG

CTCACACTGGACCCGCCACTGCCCTCTCAAAACTCGGCCACCGTACGCAGTGCCACCGTC

GTCCTGGAGTGCGCGTCCCGCTCCCTGGCCGGGTTTGCTGCCTTGTTGAGCTACATCGGC

CTGCTGGCAACTGTTTGCTTCTTGCTGGCTTTCTTTGCCAGGAGACTGCCAGATAATTTT

AACGAGGCGAAGTTCATCACCTTCAGCATGCTGATCTTCTGCGCTGTGTGGATTGCGTTT

GTCCCTGCCTATGTGAGCTCGCCAGGAAAGTACACGGTAGCTGTGGAGATCTTCGCCATC

CTGGCCTCAAGCTATGGACTGCTCTTATGCATTTTTGCACCAAAATGTTACATCATTCTG

TTTCAGTCGCAAAAAAATACCAAGAAAAATATGATGGCCAAATAACAGTGTACTCCAAGT

TGTACTTTTGAGTGATTGTATGACTCCTGTCATTCTTAATTCTAATGTCATTAATCTAAT

TCCGAAGACATACTTGTTTTGTAATATGTTTGTCAGAATTTCTTGTTCTTATAATGGTAC

AGTAAAATAAAATGATTTGTGAGAATGTCAAAAAAAA

>CL6778.Contig2_All

TCCTCCTCCTCCTCCTCCAGGGCAGAGAGGCCCCTGTGCACAGGAGCAGAGGACATCCAG

GAGAAGGCGAATGCGTATTCAGATGTGACTCAGCTGGGAGTCAGCTATAATGTGTACAAG

GCTGTGTATGCTGTTGCCCATGCCATTCAAGACATGCTCGCCTGCCAACCAGGGCAGGGG

CCATTTGAAAATGGAAAGTGTCCAAGCATCAATAACATTATACCTAAACAACTACTTCAC

TACCTGGAGAGAGTGAATTTCACAACCCCTCTGGGTGACATTGTGAACTTTGATATGAAT

GGAGACCCTCCTGCATCGTACGATCTGATTAACTGGCACATCGGTGACAAAGGCATGGCT

GAATTTGTGAAGGTGGGGCAATATGACTCAAACATTGGCCCAGATCAGAGACTCCAACTG

GACCTCAATAAAGTTGTGTGGGGCGGAGGCTGGACCGACAAGGTGAAATACCAGATAATG

AATAAAATGACACTATTTCTTTAGTTATGTGCAGGGTGTGTACAGGTACATAGCCAATTG

TACTGGTTTTCTTGTCTACATGTTTATATATGTTGAATGTATGTAAAAATTAATGTCGAA

TGGACGCCATATTCCTTGTGTATGTAAATATACTTGTCAGATAAATCTGATTGTGATTTC

TGTAGGTACCTGTGTCTGTGTGCAGCATTCCTTGTGCCCCAGGCACCTGGAAAGCTCTGC

AAAAGGGGAAACCTGTCTGTTGTTTTGACTGTATACCTTGCCCAGATGGAGAAATCAGTA

ATAAAACAGGTGCAACTGAATGCACGAGATGTCCAGAGCAGTTCTGGTCCAACAACCTGA

GGACCCAGTGCATCCTGAAGCGAGAGGAATTCCTGTCTTTCTATGAGCCCCTGGGCATCA

TCCTGACGGTGCTGTCGATATCCGGGGCAATGCTCACGACCGTTGTCCTGGTGACATTCA

TCCTCCACAGAGACACGCCTCTCGTCCGCGCCAACAACTCGGAGCTGAGCTTCCTGTTGC

TGCTGTCCCTCATCCTGTGCTTCCTGTGTGCCATGTCCTTCATGGGTCGGCCCCTCGCCT

GGTCCTGCATGTTGAGACACACCCTCTTCGGGATAAGTTTTGTGGTGTGCATCTCCTGCA

TCCTCAGCAAGACGGTGGTGGTCCTGGTGGCCTTCCGCGCCACTCTGCCGGGCAGTAAAG

TGATGCGCTACTTCGGGCCACTGCAGCAAAGGCTGGGCATCTGCCTGTGCACCCTGGTGC

AGGTGCTGGTGTGTGTGCTGTGGCTCACACTGGACCCGCCACTGCCCTCTCAAAACTCGG

CCACCGTACGCAGTGCCACCGTCGTCCTGGAGTGCGCGTCCCGCTCCCTGGCCGGGTTTG

CTGCCTTGTTGAGCTACATCGGCCTGCTGGCAACTGTTTGCTTCTTGCTGGCTTTCTTTG

CCAGGAGACTGCCAGATAATTTTAACGAGGCGAAGTTCATCACCTTCAGCATGCTGATCT

TCTGCGCTGTGTGGATTGCGTTTGTCCCTGCCTATGTGAGCTCGCCAGGAAAGTACACGG

TAGCTGTGGAGATCTTCGCCATCCTGGCCTCAAGCTATGGACTGCTCTTATGCATTTTTG

CACCAAAATGTTACATCATTCTGTTTCAGTCGCAAAAAAATACCAAGAAAAATATGATGG

CCAAATAACAGTGTACTCCAAGTTGTACTTTTGAGTGATTGTATGACTCCTGTCATTCTT

AATTCTAATGTCATTAATCTAATTCCGAAGACATACTTGTTTTGTAATATGTTTGTCAGA

ATTTCTTGTTCTTATAATGGTACAGTAAAATAAAATGATTTGTGAGAATGTCAAAAAAAA

>CL6778.Contig3_All TCCTCCTCCTCCTCCTCCAGGGCAGAGAGGCCCCTGTGCACAGGAGCAGAGGACATCCAG

GAGAAGGCGAATGCGTATTCAGATGTGACTCAGCTGGGAGTCAGCTATAATGTGTACAAG

GCTGTGTATGCTGTTGCCCATGCCATTCAAGACATGCTCGCCTGCCAACCAGGGCAGGGG

CCATTTGAAAATGGAAAGTGTCCAAGCATCAATAACATTATACCTAAACAACTACTTCAC

TACCTGGAGAGAGTGAATTTCACAACCCCTCTGGGTGACATTGTGAACTTTGATATGAAT

GGAGACCCTCCTGCATCGTACGATCTGATTAACTGGCACATCGGTGACAAAGGCATGGCT

GAATTTGTGAAGGTGGGGCAATATGACTCAAACATTGGCCCAGATCAGAGACTCCAACTG

GACCTCAATAAAGTTGTGTGGGGCGGAGGCTGGACCGACAAGGTGAAATACCAGATAATG

AATAAAATGACACTATTTCTTTAGTTATGTGCAGGGTGTGTACAGGTACATAGCCAATTG

TACTGGTTTTCTTGTCTACATGTTTATATATGTTGAATGTATGTAAAAATTAATGTCGAA

TGGACGCCATATTCCTTGTGTATGTAAATATACTTGTCAGATAAATCTGATTGTGATTTC

TGTAGGTACCTGTGTCTGTGTGCAGCATTCCTTGTGCCCCAGGCACCTGGAAAGCTCTGC

AAAAGGGGAAACCTGTCTGTTGTTTTGACTGTATACCTTGCCCAGATGGAGAAATCAGTA

ATAAAACAGGTACACTTCTTTATTCACTTTAATATTTTCCACTTTTACTCCCAATTTGTT

CCATTAGGATCGGTGTTATACTGCTTAGACAATATGCACTGTTTCGCAAGAAGTGTCAAT

CCCAGCTTCAGGAAATAATAACCCTGCCATGTCTTCCCTCTGCCTGCCTGTGTACTTATC

ACAGGTGTTTGCACAGATCAGCTGAGCTGCATATCTGATGAT

>CL6778.Contig4_All

TCCTCCTCCTCCTCCTCCAGGGCAGAGAGGCCCCTGTGCACAGGAGCAGAGGACATCCAG

GAGAAGGCGAATGCGTATTCAGATGTGACTCAGCTGGGAGTCAGCTATAATGTGTACAAG

GCTGTGTATGCTGTTGCCCATGCCATTCAAGACATGCTCGCCTGCCAACCAGGGCAGGGG

CCATTTGAAAATGGAAAGTGTCCAAGCATCAATAACATTATACCTAAACAACTACTTCAC

TACCTGGAGAGAGTGAATTTCACAACCCCTCTGGGTGACATTGTGAACTTTGATATGAAT

GGAGACCCTCCTGCATCGTACGATCTGATTAACTGGCACATCGGTGACAAAGGCATGGCT

GAATTTGTGAAGGTGGGGCAATATGACTCAAACATTGGCCCAGATCAGAGACTCCAACTG

GACCTCAATAAAGTTGTGTGGGGCGGAGGCTGGACCGACAAGGTACCTGTGTCTGTGTGC

AGCATTCCTTGTGCCCCAGGCACCTGGAAAGCTCTGCAAAAGGGGAAACCTGTCTGTTGT

TTTGACTGTATACCTTGCCCAGATGGAGAAATCAGTAATAAAACAGGTACACTTCTTTAT

TCACTTTAATATTTTCCACTTTTACTCCCAATTTGTTCCATTAGGATCGGTGTTATACTG

CTTAGACAATATGCACTGTTTCGCAAGAAGTGTCAATCCCAGCTTCAGGAAATAATAACC

CTGCCATGTCTTCCCTCTGCCTGCCTGTGTACTTATCACAGGTGTTTGCACAGATCAGCT

GAGCTGCATATCTGATGAT

>Unigene15989_All

ATTTGGCTGGCCACCCGCCCCCCCTACGCAGTGCACAACACAAGGTTCCTGAGCGCCAAG

ATCATCCTGGAGTGTGTGGTGGGGTCGGAGGTGGGCTTCTGGTGTGTGCTGGGCTACATC

GGTTTACTAGCATGCATGTGCTTTTTAATGGCCTTTTTGGCCAGAAAATTGCCAGACAAT

TTCAACGAAGCCAAGTTCATCACTTTCAGCATGCTGATTTTTTTCGCGGTTTGGATCACT

TTTATCCCTGTGTATGTTAGCACGTCAGGAAAGTACACGGTGGCTGTTCATGTGTTCGCC

ATTTTAGCCTCCACTTTTGGCCTCCTGTTTTGTATTTTCATGCCCAAATGCTACATCATC

TTGCTCAAGCCAGAGAAAAACAGCAAACAACATATGATGCGAAAATAAAGAAATGGTGCA

AAGGGCTGCATGATTGGACACCAAACACCATACAGATGTTGAAAATGCAATTGATTGTTA

AAATGTTTGTTTAAGAGTAAATGTTTTGTTTATCAGTCACTGTTCATGTTTAATTTTATT

TAAATCGTTTTTTTTTAGTAACATCAACCCAATGGTTGAGAACTGAGAGTGTTTTCAAGT

GTAGAGATTATATGAAAGACATGGCAGAGTTATTGTTTAATGTCCCTAACAAC

>Unigene54792_All

CACAGCGTTTGGGATCACCTTTGCTCTCTGTCTGTCCTGTGTCTTGTCTAAGACTGTGAC

AGTCGTCATGGCTTTTAAAGCCTCAGTGCCAGGTGCCAGGGTTCCTCAGTGTTCACTGCC

CTTTCAGAGACTAAGCGTGATGTGCTGTACACTTCTGCAGGTAATCATATGTGCCTTATG

GATTGCACTCGCTCCTCCAGTTCCACAGAAAAATATGGCCTTCTTTAGTAATAAAATCAT

ACTGGAATGTGGTCTTGGATCAGCCATCGGTTTCTGGGCTGTGCTGGGATACATTGGAAT

CCTTGCTATCTTGTGTTTCATATTG

>Unigene61739_All

TCCCAGTGACTACTACCAGAGCAGAGCCCTGGCCAAGCTGGTCAAACATTTTGGCTGGAC

TTGGGTAGGGGCCGTGCGAAGTGACAACGACTATGGAAACAATGGCATGGCAACGTTTGT

GGAGGCAGCACAGAGAGAGGGAGTGTGCATAGAGTACTCGGAGGCCATTCTGAGGACCAA

CTCTCGCGAGAGGATTGCTAGCGTGGTCGAGCAGATAAAGATGGGCACGGCTCGGGTCCT

GGTGGCCTTCTTGGCGCAGAGTGAGATGGAGGTACTGTTGGAGGAGGCCCTCCTTCAGAA

TGTCACCGGCTTGCAGTGGATAGCGAGCGAATCCTGGATCACTGCTAGGTACCTCGCCAC

GCACAGGACAGCCAAGATCATTAGCGGAGCCCTTGGCTTTTCCATAAGCAGGTCAAACAT

TCCCGGGCTAAAGGACTTCCTCTTGAAAGTGAACCCATCAGAAAACAGCTCCATTCTGAG

GGAATTCTGGGAGACGGCGTTCCAGTGCCAATTGCCTGGACAAAGTGAGACAAAAAACAT

AAGGCGATGTTCAGGTTCAGAAAACCTGGGCGAGCTTAAAAATCCTTTCACTGATGTGTC

AGAGCTTAGGATCTCCAATAATGTGTACAAGGCAGTATACACAGTGGCCCACGCGGTGCA

AAACTTTTTGGGCTGTACAGATGATGAGCACACTTCAGATAACAAAGCAGAGAACAGAGA

GTGTATTCTGAGAGAAAATATTATGCCAGGTCAGGTAAGCTT

>Unigene62553_All

AACAGATTCAGTGAATTGTTTGAGGTGTTCAGAAGACACATGGCCAAACCAAGCACAGGA

CCAATGTATTCCAAAAAACATAGAGTTTTTATCTTATCATGAAACCATGGGGATAGTTCT

GTGGGTGGTCTCTGCTTTTGGGGCCTTCACTACAGTAGCAGTGTTGGGCGTGTTTGTGGC

TTATAGAAAGACGCCCATGGTTCGGGCAAACAACATGGAGTTGAGCTTCTTACTCCTCCT

GTTCCTCTGTGCCTGCTTCCTGATTGGCCTGACTTTTATTGGGGAGCCAACTGATTGGTT

CTGTCAGTTCCGGTACACCGCCTTTGGCATCAGCTTTGCTCTCTGCATCTCATGCATTCT

GGCTAAAACGGTTGTAGTGATGATGGCATTCAGGGCCACTCTTCCTGGAAGTAATGTCAT

GAAGTGGTTTGGCCCGGCCAAACAGAGAGCTAGCGTGATTGTGTGCACTTCCATACAGGT

GCTCATATGTGTTATTTGGC

>Unigene78793_All

TAGACAAAGACATGTTAGTTTATTTAACTGCACTGCTTACATTGCTAAATGCAACACATG

CATGCCGAGCCTATGGATCCCAAGAACTGCTTCATTTTTCTAAAAAAGGTGACGTTAATA

TAGGAGGCATTTTTTCATTTCACCAAAACCCGGCAGGTGTGACTCCATCACTTCAGGTCA

ACCCAGGACATGCAAAATGTGAAGGACTTGATCTTGGAGAACTACAATATGCTATGACTA

TGATCTTTGCTATTGAGGAGATTAACAATAGCACGGAGCTCTTGCCTGGCTTCACACTGG

GGTACAAGATATTCGGCTCCTGTCCCAGCATTCCCCTCTCTGTGGACGCATCACTTGCCC

TGATGAATGGTCCTGTGTCAGAGAACTGTGTCAGACCTGACACTGTCCATGCAGTCATAG

GGGAAACCACTTCCACTGCCACTATTGGCATTGCAAGGACTATGGGACCATTTCACATTC

CAGTGCTCAGTCACTCAGCCACATGTGCTTGTCTGAGCGACAGGAAAGAGTTCCCATCCT

TCTTCAGAACCATTCCCAGT

>CL14841.Contig2_All

GTTTGCCCTGGTCTCCCCGCTCTCAGGAGCTGAGCCTGTCTGCAAGCTACTGGGAGAGGC

GTTAGTCCCTGAGTTAGAGGGACAAGGAGATCTAGTCATTGGTGGTATCTTCACATTTCG

CACTGGTTACGATGGGGAGATACCTACCTTTGAAAGCTTGCCGAATGAACCCAAATGCAA

AAAACTAGGGTACAGAATCTACAATGCCTGTGGATATTCCAACATAATGAGGTCTGCAAT

TGCATTGGCAAGTGGTGTGGCAAAGGTGATTGATGGGAAGAATTGCTCAAACACCTTCAG

AGCTGAAGCCATCATAGGCCACTCAGCATCTGCTCCAACTGTGGGTTTCGCACGGGTATT

AGGGCTGTTTCAACTTCCAGTGATAAGTCATTTTGCCACATGTCCGTGTCTGAGTAACAG

GAAAGAGTTCCCCTCCTTC

>Unigene24070_All

TCATACTGGCTTTTCTGGCTCGTAGATTGCCTGATAATTTTAATGAAGCCAAATTCATAA

CATTCAGTATGCTGATATTCTGTTCAGTCTGGATTGCTTTCATCCCAGCTTATGTAAGCT

CCCCTGGAAAATTTACTGTAGCTGTGGAGATATTTGCCATTCTGGCATCTAGTTTTGGGG

TGCTCTTTTGCATTTTTATACCAAAATGTTACATTATTCTATATAAACCAGACAAAAACA

CCAAAAGACACCTTATGGGAAAAATGCCATCAAAAGCTCTTTGAGTTTTATTGTAACATC

TGTATCACACATGTATGCACATGGTTTAGTGTATATAATATGTATCTGTTATTGATAGTG

TGTTCTACATTATGTAATAGTATTAGCTCTTCATTTAAGTTGTAAACAATAAATAAATAA

ATATTAAAATGTACTGTTTCCCAGATTTACCCAAAGTCACATAAAAGTCACGAAGTGACA

GCGTAAAACTCATATAAGCTTTTTCCATTAGCATTGGAGGTCATTACCTTAAAACCCCTT

TGTTAAGAGCATGTCCTTATAGTTAAGCCACTGGAACACAGATCCCTATATGAAAGTATT

TACTGCTATGAACACAAGTAGTTATTTTGTGTGACCCGTGTGACAATGTCAGTATAACCA

ATTATTGTTTCTTAACTCGTAAACATTGCAGTGGTAGTTATATACATTGGTAGTGATTCT

TCAACCATTTAAATTGTTATAAATAAGTGTTTTATAAGATAT

>Unigene28241_All

TTCACTGTAGCTGTGGAAATATTTGCTATTTTAGCATCTAGTTATGGAATGCTTTTCTGT

ATATTTCTCCCAAAATGTTATATAATTGTATTTCAACCAGAGAAAAACACAAAGAGGCAT

TTAATGGGGAAAGTGACAACAAAAGCTCTATGAATACACAATCTAGTTTGCTGTACATCA

TGCAGCCTGCCCTGAGCAATGTTTAGCTGTAGTTTACTTATATGAAAAAAAGTTCAAATA

TTTGTCTACATTCACCTATGTACTGGAGTGAAATAAATGCCAAGATTGTCTGTACTTTCA

TGTACAGCAATTCATATCTTTATTTAAGGCAATATAATATTTAAGATTTGAGCAAGGGAT

GCAATATCTGTTGATGGTAGGTTGACCCAAAAGTTGAAAGCATTGTATTTCCCCCATGTT

ATTTTATATATTTGTTGATATATATTTGCTGATAAATCACACATTGAGAA

>Unigene65091_All

TGGAGGAGATCAACAGGAGCAAAGACTTGTTACCGAACCACACTCTTGGTTATAAGATTT

TGGACTCCTGCGCCACACCGGTGACGGCACAGAGAGCTGTTCTTGCTGTACTAAATGGGG

AGGACAGTAGAGAGAAAACCATGTGTTCTGCTTCCAGCCCTCTCCTAGCAGTTATTGGGG

AATCTGGGTCTTCACAGTCAATTGTAGTGTCCAGAACACTACAACCTTTCAGAATTCCAA

TGATCAGTTATTTCTCCACATGTGCTTGTCTGAGTGATAGGACACAGTTCCCAACATTTT

TCCGAGTAGTGCCGAGTGATGATTATCAGGTGAAGGCAGTCGCTCAGCTGCTGAGGCACT

TTGGTTGGACGTGGATCGGAGTAGTGACAGAAGATCACGATTATGGCAGATTCGCACTGC

AGGGCTTAAAAAAGGAAATCGAAAACACAGACATCTGCCTGGCCTACCACGAAATGATCC

CCAAAGATTACAGCACTGAGAAAGTGCTTAAAATACTCGAGGTGATGACAATGTCCACTG

CCCGAGTGGTGGTGGTGTTTTCGGGCGAGGGGGAGTTTTATCCCTTTCTGAAAGAGCTCA

GCAAACAAAACATAACGGGGATCCAGTGGATTGCAAGTGAGGCGTGGGTCTCGGCCTCCG

TGCTCGCAGAGACATATCCTTTCCTGGACGGCACCATTGGTTTTGCCGTCCGTAAAGGAT

ATGTCCCCAATCTCAGGGATTATGTGACAACAGTGAATCCTTGGAATTACCCCTCGAATG

TACTAGTGCAGGAGCTGTGGGAGACTTTGTATGCTTGCTCTCCCCCCAGTGCCACCACCA

CAAACACCCAGTTGTCACCCTGCACTGGTCATGAGACTGTACAGGAGCAGCACTCAGCCT

ACATGAACACCACTAGCCCCCGCGTTGCATATAATGTGTACAAAGGTGTTTATGCTATTG

CCCATTCCCTGCACAACCTCATACAGTGTACCCCTGGTCATGGCCCATTCCTCAATGGTT

CATGTGCAGATGTCAGTACTATCCAACCGTGGCAGCTACAGCATTATCTTCAGGATGTCT

CCTTCTTCATCTCGGGAGAAAAAGTCAATTTTGATATGAAAGGGGATTCTATTCCATCAT

ATGATCTGATAAACTGGCAGAGGGATACTTATGGACAAATTCAGTTTGTGACAGTGGGTC

TGTATGATGGAGCAAAAGTTGCTGGGAAAGAGCTTGTTATTGATAAGGACACAATCAAGT

GGACAGGACAGCGGAAAGAGGCAAGATGCACCTTTTTGGAATTAAAATGTCACCTTTTGA

AGTACTATAGACAGCACATGCAGTGCTGGTGTCTGTGTGTAGTGAC

>Unigene77993_All

ATTTGTCCCTGCCTATGTGAGCTCACCAGGAAAATACGCCATCGCTGTGGAAATCTTCGC

CATTCTGTCATCTAGCTTTGGCCTCCTCCTTTGCATATTTGCTCCAAAATGCTTCATTAT

ATTAATACAACCTCATAAAAACACCAAGAAATTCCTAATGGGAAAGACTGAGAAAACATA

GAAATGATTACATTCCTCGTAAGAAGACATTTTTGTCCATGGAATGTTGTAACTCTCATG

TGACCATTTTTCAACATATATATAATACACACATATATAATTATTATACAGTCAATGTTC

AAAAATGCAATGTTACACAGTAATACAGTTATGTCTTTG

>CL10326.Contig1_All

CAAAAACAGAGAGAGATGAAACAAGACAGAGGGACGACATGCAGGTCTCTCCTAGCCACT

TCTTGCTGGCCTGGACAGTATCAGCAGCCACACTGCTCTCTCCCGCTCTCACCTCCAGCT

CTGACACCCCTAACCTCTGCCAGCTGCGGGAAGAGTTTAACCTAACTGGGATGCACCAGG

ATGGAGATGTGATCATAGGAGGACTGTTTGAGATCAACTTGTTCACTGTATTTCCAATGC

TATCTTTCAGGACTAAACCAGACCAGCCCTGGTGTTATAAGTCTGATCTAGAAGGTTTTA

AGATGGCGCAGACAATGGCGTTTGCCATAAATGAGATAAACCAGAACCCAAATCTGCTTT

CAAACATTTCTCTGGGATACAGCATCTATGACAACTGTGTAAAACTTGGAGTGGCCTTCC

GTGCTGCCTTAGCCCTTATCAGCGGAACACAAAAAACCCTCTCTTCATCTGACTGCAGGG

AAGGCTTGCCACCTGTCCTAGGAATTGTGGGGGATTCCGTGTCGACTCACTCGATTGCCA

TTTCTAGTGTGGCGGGTCTGTTTCATGTCCCAATGGTAAGCTACTTTGCTACATGTTCCT

GCTTAAGTGACAGACAGCGGTACCCATCTTTTTTCAGGACTATCCCCAGTGATGCTTTTC

AAGTGAGAGCTATGATTCAGATACTGAGGCACTTCAAATGGACATGGGTGGGTCTCATCT

ACAGTAATGATGACTATGGCCGACATGCTGCCCAGTCTTTCCACAAGGACATCACTACAT

TTGGTGGCTGTGTGGCATTCTCAGAAATGCTGCCCAAAGATAATGATATTGTGGAGATCA

GAAAGATCATGCAGGTGATAAGGGAATCTACATCACGTGTGATTGTGGTCTTCTCCACTA

AGTCTTACCTACTGCCACTCATGGACGAGGTAGCTCAACAGAACGTGATTCGCCAGTGGA

TTGCCAGTGAGGCGTGGTCCTCCTCCCCACTGTTCCTCACCCCACGCATGCTGCCCTTCC

TTAAAGGCACTCTGGGCATCACTATTCGCAGAGGAGAGATCGATGGACTGAGAGAATTTT

TGCTGAGCGTCCGCCCTGACAATGATTCTAAAAACAATATGGTGAGAAGGTTCTGGCAAG

AGACGTTCAGCTGCAGGTTTGACCCAAAGGAGGCCTTGGCAGTGGCTGAGGGAAAGGTCT

GTACAGGACAGGAGGACCTGAGCAAGGCTGACATGGCGTACAGCGATGTGTCTGACCTCA

GGCCTTCCTACAATGTCTATAAAGCAGTCTATGCACTGGCACATTCTCTCCACAACCTTC

TGAGCTGTGTCCCAGGACAAGGCCCCTTTAAGGGGAATAGCTGTGCCAGTCTCCATGACA

TGCAGCCCTGGCAGCCTCCTAGGTCAGTGTGTAGTGAGCCCTGCCCCCCTGGAACCAGAA

AAGCCACAAGGAAGGGACTACCTGTCTGCTGCTTTGATTGCCTCCATTGTGCAGATGGAG

AGATCAGTAACACCACAGATGCCATTGAGTGTGTTAAGTGCCCTGTGGAATTCTGGTCAA

GCTTGAAGAAAGATCGATGTGTCCCTAAAGAGGTTGAGTTTCTCTCCTATGAGGAGGCAT

TGGGCATCTCTTTAACAACAGTGTCCATATTTGGTGCATGCGTCTCTGCTGTGGTCCTTG

CAATTTTTGTTCACCACCGTCATACACCTGTGGTACGAGCCAACAACTCAGAACTGAGCT

TCCTCATTCTGCTTTCACTAAAACTCTGTTTCCTGTGTGCACTGCTATTCATTGGTCAGC

CCAGGACGTGGACCTGTCAGCTCCGCCACGCAGCATTTGGTATCAGCTTTGTGCTCTGTG

TCTCGAGCATTCTGGTGAAGACGATGGTGGTCATTGCCGTTTTTAAGGCCTCAAGACCTG

AAGGTCAAGGGGCCATGAAATGGTTCGGAGCAGCCCAACAAAGAGGCACAATTTTTATCC

TAACCTCTCTCCAGGTGGCCATCTGTGTGATCTGGCTCTCCACTGCCTCTCCTACTCCCC

ACAAAAACACTCACTACCAGAGCTCCAAGATTGTGTTTGAATGTGCAGTTGGCTCAGTGG

CAGGGTTTGCTACATTGTTGGGCTACATAGGCCTTCTGGCGGCCATTAGCTTTCTCTTAG

CTTTCTTAGCAAGAAATCTTCCAGATAATTTTAATGAGGCCAAGTTTATTACCTTTAGTA

TGCTCATCTTCTGTGCTGTGTGGATTGCTTTTGTTCCTGCTTATGTTAGTTCCCCTGGTA

AGTATGCAGATGTGGTTGAAATTTTTGCCATCCTGGCTTCAAGCTTTGGCCTGCTCATTG

CTATATTTGCCCCCAAATGCTACATAATTCTCCTGCATCCAGAGCGAAACACCAAGAAAG

CACTAATGAGTAGAGGTTCTTAAAAGTAATAACAGACTTATTTAGATATATTATGTCACT

GAAATCATAACAAAGTGTACAACAGCGCTTCCTCTTAAAATATTACAAATTAAGAAAATG

CAGATTGATGCAGATAGATCCCTGCCAAATCTAAATTTAGTTTAGCAATTTTATAACAAG

CCAACAATGTGCAAGTCCCACTGGTGTTTATGTAAATGTCATGCTTGCATATTCTATGGC

TAGGCTACTGCCCAAATTGACAGTGTAACACACCACGCAAAGTCATATCCTACTTTTACA

GATTTTAAGAGTTTAAAATGAACCTATTTGAGTCTGCCTTTTGAACAACAGTAGAGTAGG

TAGATTTGTGTTGTCCTACGTAAATATTTAGAACTGTTGTCCCTATATTATCAGTGGGTT

AATGTGGCCACTGCAAATGACACAGGCAGACCAGACAGTAGTGGCGCACTATAAAGACTT

ACCTTAAATGTTAGAATTGAGAGGCATCCTAAAAGTTCCCCCAAAACTTGCTAATGTTAT

GACTGAAAATATGTTATTAAAAACACATCACAAGTCTATCAAATATATCTTTACTCACAA

TAAAAGAAAGTTTAAGAATAAAA

>CL10326.Contig2_All

CAAAAACAGAGAGAGATGAAACAAGACAGAGGGACGACATGCAGGTCTCTCCTAGCCACT

TCTTGCTGGCCTGGACAGTATCAGCAGCCACACTGCTCTCTCCCGCTCTCACCTCCAGCT

CTGACACCCCTAACCTCTGCCAGCTGCGGGAAGAGTTTAACCTAACTGGGATGCACCAGG

ATGGAGATGTGATCATAGGAGGACTGTTTGAGATCAACTTGTTCACTGTATTTCCAATGC

TATCTTTCAGGACTAAACCAGACCAGCCCTGGTGTTATAAGTCTGATCTAGAAGGTTTTA

AGATGGCGCAGACAATGGCGTTTGCCATAAATGAGATAAACCAGAACCCAAATCTGCTTT

CAAACATTTCTCTGGGATACAGCATCTATGACAACTGTGTAAAACTTGGAGTGGCCTTCC

GTGCTGCCTTAGCCCTTATCAGCGGAACACAAAAAACCCTCTCTTCATCTGACTGCAGGG

AAGGCTTGCCACCTGTCCTAGGAATTGTGGGGGATTCCGTGTCGACTCACTCGATTGCCA

TTTCTAGTGTGGCGGGTCTGTTTCATGTCCCAATGGTAAGCTACTTTGCTACATGTTCCT

GCTTAAGTGACAGACAGCGGTACCCATCTTTTTTCAGGACTATCCCCAGTGATGCTTTTC

AAGTGAGAGCTATGATTCAGATACTGAGGCACTTCAAATGGACATGGGTGGGTCTCATCT

ACAGTAATGATGACTATGGCCGACATGCTGCCCAGTCTTTCCACAAGGACATCACTACAT

TTGGTGGCTGTGTGGCATTCTCAGAAATGCTGCCCAAAGATAATGATATTGTGGAGATCA

GAAAGATCATGCAGGTGATAAGGGAATCTACATCACGTGTGATTGTGGTCTTCTCCACTA

AGTCTTACCTACTGCCACTCATGGACGAGGTAGCTCAACAGAACGTGATTCGCCAGTGGA

TTGCCAGTGAGGCGTGGTCCTCCTCCCCACTGTTCCTCACCCCACGCATGCTGCCCTTCC

TTAAAGGCACTCTGGGCATCACTATTCGCAGAGGAGAGATCGATGGACTGAGAGAATTTT

TGCTGAGCGTCCGCCCTGACAATGATTCTAAAAACAATATGGTGAGAAGGTTCTGGCAAG

AGACGTTCAGCTGCAGGTTTGACCCAAAGGAGGCCTTGGCAGTGGCTGAGGGAAAGGTCT

GTACAGGACAGGAGGACCTGAGCAAGGCTGACATGGCGTACAGCGATGTGTCTGACCTCA

GGCCTTCCTACAATGTCTATAAAGCAGTCTATGCACTGGCACATTCTCTCCACAACCTTC

TGAGCTGTGTCCCAGGACAAGGCCCCTTTAAGGGGAATAGCTGTGCCAGTCTCCATGACA

TGCAGCCCTGGCAGCTAACTCACTACCTACAGGATGTGAATTTTAGCACAGGTTTTGGGG

ACCAGGTGTCTTTTGACAAAACTGGCGATGCCTTGGCCATCTATGACATCTTCAACTGGC

ATGGGATGCCAGATGGCACAATGAAAGCCAGAACAGTGGGTGTTGTTGATGAATCACAAC

CAACTGAGAATGTGTTTCATCTGGATGAAGGCAATCTCTCCTGGAACTTTGAATTTAACA

AACCTCCTAGGTCAGTGTGTAGTGAGCCCTGCCCCCCTGGAACCAGAAAAGCCACAAGGA

AGGGACTACCTGTCTGCTGCTTTGATTGCCTCCATTGTGCAGATGGAGAGATCAGTAACA

CCACAGATGCCATTGAGTGTGTTAAGTGCCCTGTGGAATTCTGGTCAAGCTTGAAGAAAG

ATCGATGTGTCCCTAAAGAGGTTGAGTTTCTCTCCTATGAGGAGGCATTGGGCATCTCTT

TAACAACAGTGTCCATATTTGGTGCATGCGTCTCTGCTGTGGTCCTTGCAATTTTTGTTC

ACCACCGTCATACACCTGTGGTACGAGCCAACAACTCAGAACTGAGCTTCCTCATTCTGC

TTTCACTAAAACTCTGTTTCCTGTGTGCACTGCTATTCATTGGTCAGCCCAGGACGTGGA

CCTGTCAGCTCCGCCACGCAGCATTTGGTATCAGCTTTGTGCTCTGTGTCTCGAGCATTC

TGGTGAAGACGATGGTGGTCATTGCCGTTTTTAAGGCCTCAAGACCTGAAGGTCAAGGGG

CCATGAAATGGTTCGGAGCAGCCCAACAAAGAGGCACAATTTTTATCCTAACCTCTCTCC

AGGTGGCCATCTGTGTGATCTGGCTCTCCACTGCCTCTCCTACTCCCCACAAAAACACTC

ACTACCAGAGCTCCAAGATTGTGTTTGAATGTGCAGTTGGCTCAGTGGCAGGGTTTGCTA

CATTGTTGGGCTACATAGGCCTTCTGGCGGCCATTAGCTTTCTCTTAGCTTTCTTAGCAA

GAAATCTTCCAGATAATTTTAATGAGGCCAAGTTTATTACCTTTAGTATGCTCATCTTCT

GTGCTGTGTGGATTGCTTTTGTTCCTGCTTATGTTAGTTCCCCTGGTAAGTATGCAGATG

TGGTTGAAATTTTTGCCATCCTGGCTTCAAGCTTTGGCCTGCTCATTGCTATATTTGCCC

CCAAATGCTACATAATTCTCCTGCATCCAGAGCGAAACACCAAGAAAGCACTAATGAGTA

GAGGTTCTTAAAAGTAATAACAGACTTATTTAGATATATTATGTCACTGAAATCATAACA

AAGTGTACAACAGCGCTTCCTCTTAAAATATTACAAATTAAGAAAATGCAGATTGATGCA

GATAGATCCCTGCCAAATCTAAATTTAGTTTAGCAATTTTATAACAAGCCAACAATGTGC

AAGTCCCACTGGTGTTTATGTAAATGTCATGCTTGCATATTCTATGGCTAGGCTACTGCC

CAAATTGACAGTGTAACACACCACGCAAAGTCATATCCTACTTTTACAGATTTTAAGAGT

TTAAAATGAACCTATTTGAGTCTGCCTTTTGAACAACAGTAGAGTAGGTAGATTTGTGTT

GTCCTACGTAAATATTTAGAACTGTTGTCCCTATATTATCAGTGGGTTAATGTGGCCACT

GCAAATGACACAGGCAGACCAGACAGTAGTGGCGCACTATAAAGACTTACCTTAAATGTT

AGAATTGAGAGGCATCCTAAAAGTTCCCCCAAAACTTGCTAATGTTATGACTGAAAATAT

GTTATTAAAAACACATCACAAGTCTATCAAATATATCTTTACTCACAATAAAAGAAAGTT

TAAGAATAAAA

>CL10326.Contig3_All

CCCCCCTGGAACCAGAAAAGCCACAAGGAAGGGACTACCTGTCTGCTGCTTTGATTGCCT

CCCTTGTGCAGATGGAGAGATCAGTAACACTACTGATTCTCTTGAATGCTTTAGATGTCC

AAAGGAGTTCTGGTCAAGCCCAGAGAAAGATCGATGTGTCCCCAAGGAGGTCGAGTTTCT

CTCCTATGAAGAAGCATTGGGCATTTCCTTGACAACAGTGTCCATATTTGGGGCATGTAT

AGCTGCTGTGGTTCTTGGAATATTTGTTCACCACCGTCACAC

>CL14841.Contig1_All

GTTTGCCCTGGTCTCCCCGCTCTCAGGAGCTGAGCCTGTCTGCAAGCTACTGGGAGAGGC

GTTAGTCCCTGAGTTAGAGGGACAAGGAGATCTAGTCATTGGTGGTATCTTCACATTTCG

CACTGGTTACGATGGGGAGATACCTACCTTTGAAAGCTTGCCGAATGAACCCAAATGCAA

AAACCTTAACTACAGAGAATTTAAATTTGCACACACAGTTATATTTGCAGTTGAAGAGAT

AAATAAAAATCCCAAAATTCTTCCACAACATAGACTAGGGTACAGAATCTACAATGCCTG

TGGATATTCCAACATAATGAGGTCTGCAATTGCATTGGCAAGTGGTGTGGCAAAGGTGAT

TGATGGGAAGAATTGCTCAAACACCTTCAGAGCTGAAGCCATCATAGGCCACTCAGCATC

TGCTCCAACTGTGGGTTTCGCACGGGTATTAGGGCTGTTTCAACTTCCAGTGATAAGTCA

TTTTGCCACATGTCCGTGTCTGAGTAACAGGAAAGAGTTCCCCTCCTTC

>Unigene10816_All

TGAAGTGGTTTGGGCCTCTGCAACAGAGACTCAGTGTTCTTGGCTTCACTCTCATACAAG

TCCTTATATGTGTGCTGTGGTTAACTATATCCCCTCCTTTCCCTTACAGAAATATGCACC

TTTACAATGATAAGATCATTCTAGAATGTGATGTGGGTTCAGCTATAGGATTCTGGGCCA

TATTAGGTTACATCGGACTACTCGCCATCCTGTGTTTCTTTCTGGCTTTTCTGGCTCGTA

AACTACCTGATAACTTCAATGAGGCCAAATTCATCACATTCAGTATGCTCATATTCTGTG

CAGTTTGGATCACTTTTATCCCAGCTTATGTCAGCTCTCCTGGGAAATTTACTGTAACTG

TAGAGATATTTGCTATTTTAGCCTCAAGTTCTGGTTTGTTATTTTGCATTTTCTTACCAA

AGTGCTACGTTATTTTAGTAAGGCCAGACTTGAACACTAAAAAGCATGTAATGGGAAAAA

CATCCACTGACATTCATTATTGAATACTGCCAACTTTTAAATCCTGAATGGTAACAGTAC

AGCATAATTTGAAATAACATTTCTAAAACCACAGCCTGTTTGAGTCATCTTTTAAAAACT

ATAAGCAGTTGAATAAGTGAACTGAAAAAGACTACTCTATTACACACTGTTAC

>Unigene1953_All

TGAAGTGGTTTGGGCCTCTGCAACAGAGACTCAGTGTTCTTGGCTTCACTCTCATACAAG

TCCTTATATGTGTGCTGTGGTTAACTATATCCCCTCCTTTCCCTTACAGAAATATGCACC

TTTACAATGATAAGATCATTCTAGAATGTGATGTGGGTTCAGCTATAGGATTCTGGGCCA

TATTAGGTTACATTGGACTACTTGCTATCTTATGTTTCATACTGGCTTTTCTGGCTCGTA

AACTACCTGATAACTTCAATGAGGCCAAATTCATCACATTCAGTATGCTCATATTCTGTG

CAGTTTGGATCACTTTTATCCCGGCTTATGTCAGCTCTCCTGGAAAATTTACTGTGGCTG

TGGAGATATTTGCCATTTTAGCCTCCAGTTTTGGATTAGTATTTTGTATATTTATTCCCA

AATGCTATGTTATTATTTGTAGGCCACATGAAAATACAAGGAAACACATCATGCAAAAGA

ATCCTCAGTAAATGTTGACTTTCCATGGTCAGACAAATGTACATCTTTGCTTCCATAATT

TTTATAATGCTAAAATAATAATTAAAGAAAAAAACATGACATAC

>Unigene51872_All

GATGGATGCAGACTATGATATTTGCAATCGAAGAGATCAACAAAAATCCAAATCTTCTGC

CAAGCATCACACTTGGCTATCAGATCTATGATTCCTGCAGCACGCCTGTCCAGGCACTTC

GCACAGCCCTGGCTGTGACAGGGGGCCAGTTTGAAGAGGAGAGTGGCAACTTGAAGTGCA

GCGGAACAGTGCCTGTGGTTATTGGGGACGGCGGGTCAACACTCTCTCTGGTAGTGGCAA

GATTCCTGGGTGTTTTTCATGTTCCACAGGTAAGCTACTTCTCCAGCTGTGCCTGTCTAA

GTAACAAAGTTGAGTTTCCTGCTTTCTTGAGAACCATGCCTAGTGACTTTTTTCAAGTGG

ACGCTCTGGCTCAGCTGGTGAAACACTTTGGCTGGA

>Unigene66147_All

TGCATGCTGCGCCACACGGCCTTCGCTGTGACCTTCGCCCTCTGCATCTCCTGTGTTCTG

GGCAAAACGCTGGTGGTGATCACAGCCTTCCGTGCCACACTGCCAGGCGCAACGAAGTTC

GGGCCCGTGCAGCAGAGGGTCATTGTGTATTCCTGCACTGTTGTACAAGTTTTTATCTGC

ATACTCTGGCTGACTATCACACCCCCGTATCCTCAGAAAAGGCATAACAATAGAAAGATT

ATTGTAGAGTGCAATACAGGGTCAGACACAGCCTTTTATGCTGTGTTGGGTTACATTGGC

TTTTTGGCGGGTATATGTCTAGTTTTAGCTTTCTTAGCAAGAAAATTACCAGATAATTTT

AACGAAGCCAAATTTATTACTTTTAGCATGCTTGTGTTCTGTGCTGTGTGGATTACATTC

ATCCCTGCATACGTGAGCTCACCAGGAAAATATACTGTTGCCGTGGAAATATTTGCTATT

TTGTCCTCAGCTTTTGGCTTGCTTGTTTGTATTTTTGCCCCAAAATGTTATATTATTATA

TTTCATTCTGAGAAAAACACTAGACAACATGTGATGGGCAAGGTACCTAAAACTGGTCTT

TGATAACATTGTACATGATTTTGTTCAGTGTGTCCTGTTTATCTGAAAAGAAGATGGGCT

TGTAAATTATGTACGGACCGTTATATATGCAGTCTATGGTACGGACCAGGTGTTCTTAAA

AATAATTTCTGTATATATTTTGTATATTTTGTGTATTTTGTATATTCTGTATAAGTCTAC

AAGTATTTATAATAGTGTAGTTATAGTAACCAGGCTTAAGACTAATGGCAATAAAACAAA

AACTAATTTGTCTACTGTATGTATTATGAAGGGTGTAACTGCACTGTAAACAAAAGAACT

GTAAATAAATAACATGGCTACTTCTTTTAA

>Unigene71950_All

GGGGATGTCAGGCCTTTTTTCTGTTTACCATATGCCATGTGTCTTTCTCTGTCTTCTGTG

TAGGTGATGGGCATCCGCATCAGCTTCTGGCTGCTGTTGGTCCTCTGTGGTCAGGCCTGG

GTAGGGGCCGAAGAGGAGAAGGGCGTCTGTCGTCTGCAGTGGAGGACTGTGTCTAATAGT

CTCTACAGGGAGGGAGATGTGATCATCGGGGGTCTGTTTCCCCTCCATGTTGTGGCTCCT

GAGCTGGATCTCAGTTTCAAAGGGAAAGTGACTACCGCTGTATGTCAGAGGTTCATCTTG

TGGCACTATCAGTGGATGCAGACTATGGTGTTCGCCATTGAGGAGATAAACCAAAGCCCA

AGCTTGCTCCCAAACCTCACTCTGGGGTACCTGGCCTTTGACTCCTGCCTGGCGGAACAC

ACCACGGTCGGGGCAGCACTGGCCATGGTGGCGGGACAGGAGGATGCCGTATCTGGCTCC

AGCTGCACAGGTGGCCCTCAAGTGCCCGTCATCATCGGGGACCCCAGGTCGTCCGCCTCT

ATTGCAGTCGCCCGGACTCTGGGCATTTTTGACATTCCCTTGGTCAGCTACTTTGCCTCC

TGTGCTTGTCTAAGTGACAGACAGAAGTACCCAACCTTCTTCCGCACTGTCCCCAGCGAC

GCTTTCCAGGCCAAGGAGCTGGCCCGCCTGCTGCAGCTGCTGGGCTGGGTTTGGATCGGC

GTGGCGTTCGGAGACGACGACTACGGCCGCTACGGGGTACAGCTGCTGCTAAAGGAGCTG

CAGGGCTCCGACGTGTGTGTGGCTTTTAGCCAAGTCATCCCCAAAGCCCACAGCCCCAGG

AGGATAAGACACATCGTGGAGACCATCCGTCTGTCTACGGCGAAGGTGGTGGTGGTCTTC

GCCATCTCCCAGGATGCCCAACCGCTCCTGGAGGAGGCGGTGAGGCAGAACGTCAGCGAC

AGGCAGTGGATTGGCTCAGAGGGCTGGGTCACCTCTTCCGACATCTCCACCCCCCAAAAC

CTGCCCTCGCTGGTGGGCACACTGGGCTTTGCCCTGAGGAAGGCCCAGATCCCCGGCCTG

GGCCCCTTCCTCATCCGCATCAGGCCCGCGGGGTCTAAGACAGAGCCGTTTGTGAGGAAG

TTCTGGGAGACGTTGTTTGAGTGCTCGCTGGAGGGTAA

>Unigene55404_All

CTCTATCTCTCATCCTCCTTGTTGCCCTATGGGGTGTGGGGCTAACACCTGTGGAGTCTG

GAACTAATTTAGTCACCTGCAGGCTGCAAGGATCTACCAAGTTTCCTGCATTTTCCAAAG

ATGGAGATTTTGTGATTGGGGGAATATTTTCAATCCACTATTACATGCATACTGTACAGC

ACAACTATACAAGAGTGCCAGAACCTCTAAAATGCACTGGGAGCATGGACACTCGGGAGC

TCCGATTTGCTCGCGCTAT

>CL16219.Contig1_All TTCATGGTTGGGGGATACATGGAGGAATGGTTAACACTACTTTTGTGCACAAGATTTATT

TTCCATGACATATTAGGACTAAATGGCTTTTACAGCAATGGGGGATCACACAGTAGTTAA

CTACTGTTTTCAAACGCTAAACAACTCCTGCACAAAGGATATCAGAGCACAAGGGGAGTA

CATAATTCTGTACATACTCTTGTTCCTGATATCGGCATCAACTGTGTTTCTGAATCTTTT

AGTGGTCATTGCTGTTTCTCACTTCAAGCAGCTTCATACTCCAACCAACCTGCTCCTTCT

CTCTCTGGCTGTGGCAGACTTGCTTGTGGGCTTCCTGGTAATGCCTGTGGAGGGCATGAG

ATTAATTGAAAGGTGTTGGTATTTTGGAGACACATTCTGCTACATATTTCCACTAATTTT

GTTTGTTGTTATTTCAGCCTCTTTGGGAAATCTGGTTTTTATATCTGTTGATCGTTACAT

TGCTGTTTCCAATGCTTTGAACTATTATTCATACATAACACTTAACAAGGCAGTGCTTTG

CATATTTTTTAGCTGGTTTGGTTCTTTTGTTTATTCGGTGCTGATACTGTCCAATCATCT

TCTTCAACCTGAGCCACACAGGACTTGCCATGGAGAGTGTCTGCTTGTAATCAATTTTTC

ATGGATTATTGCTGACCTTTTTGTTTCATTTACAGTGCCATGTTCCATTGTAATATGTCT

ATACCTTAAAATATTTAGTATGGCTAAACACCAGACATTAGCAATCAACTCTGTTAGAAA

TCCTGGCATGACCACAAATGAAAATGTCAAAATACGTAAATCTGTCAACAAAGCAGCAAG

AACATTAGGAATTCTTGTGGCTGTTTATTTGCTCTGTTGGATTCCTTACTATGTAAGCAT

TCTTGCTAACGGTAGCATTTCATCATCATCTTTTATAGTGACTTTCCTCAGCTGGACTAT

GTACATGAACTCTTGCATGAACCCACTGATTTATGCACTGTTTTATCCATGGTTTAGAGT

GTCAATTAAGCGCATTTTAACACTGGGCATACTCGACCCAACTTCACCATATTACAGTGT

CTATCCAGATGATGTATAACTTTGAAAAGAATCACAGTTATACTTTGGATGTATTTATAT

GTTCCCTTCACTGTTTACCAGTACTAGTTCTGTAGTAACATTAAATGGCAGTCTCGCTAT

CATTTCATGGTTTCAACTATCAGCTAAACATAATTTAACACTGGCAATACTTGAAC

>CL16219.Contig2_All CTTCAAGCAGCTTCACACTCCGACAAATCTGCTCATCCTCTCTCTAGCTATGGCTGACTT

ACATGTCGGACTACTGGTGATGCCTGTGGAAGGCATGAGATTGATTGAAACGTGTTGGTA

TTTTGGAGACGCATTTTGTTACGTATTTCCTGTTATTATGTTTGTTGTTGTTTCAGCCTC

TTTGGGCAATTTAGTTTTCATATCGGTTGATCGCTACATCGCTGTTAACAATCCTTTGAA

ATATTATTCATGCGTAACACCTAACAAAGCAATGCTGTGCATATCTCTTAGCTGGTTTGG

ATCTTTTGTGTATGCAATGCTAATGCTGTCCGATCATCTTCAGTCAAAGCCTCAGAGGGC

TTGCCATGGAGAGTGTCTGATTGTATTCAGCGTTCCGCGAGTCATTTCTGATGTTGTGTT

TTCATTTCTAGCTCCTTGTTCAATTGTCATATGCTTATACGTGAAAATATTTTTTATGGC

TAAATACCAAACACGAGTTATGAACTCTGTCAAAAATGCCAACATGACAACTGAACATGT

TAAATTTAGGAAATCAGAGAGCAAAGCAGCAAAAACATTAGGTATTGTTGTAGCCATTTA

TTTGCTCTGTTGGATTCCTTACTATATCTGCACACTTGCTTATGGTAGTGCTTCATCAAC

ATCTGTTGTATTGACTTTTCTCATTTGGTTTATGTATATTAACTCCTGCATGAATCCACT

TATTTATGCACTATTTTATCCATGGTTTAGAGTCTCTGCTAAACATATATTAACACTGGC

AATACTTGACCCAGGCTCACAATATTACAGTCTGAATCCAGATGATGTATAACATTTGGG

AAACCTA

>CL337.Contig1_All TTACCAGCCACTGGGGCATGTGATCCTGGATTCATTGTGCAAACTTGAGGACATCGTAGA

CACTTGGCATGATCTCCAACACAACCTCTCTTTCTATGACCACTAAAGGAAGTAATTTTA

CTGCTTACTGTTCTGCTTCCACTGAACTACTCAGTTCGATGAGACCGTCAGAAACTGCAG

TGCGTGTGATATTGTACTTGATTTTGACTGCTGTTATCCTTTTAACTGTGTGTGGGAATC

TGCTGGTTATTATCTCGGTGTGTCACTTCAAGGAGCTGCACACACCAACTAATGTGCTCA

TCCTCTCTTTGGCCTTTTCTGATATCCTCACAGGGCTCACAGTAATGCCATTTCATTTTG

TTTGGATGATTGAATTGTGCTGGATTTTTGGAGCAGCAATGTGGATACTTTACAGCTTTG

CAACTTGTCTACTATCTTCTCAGTCTGTGTACAATGTTACTCTCCTTGCTTTAGATCGAT

ATGTGGCTTTGAATTATCCATTTTTCTACTTGGACAAAGTCACATTAAAACGGAGTGTCA

TAATAGCATCCCTGTCCTGGCTGTTTTCAGTATTTTATAACTATATTCTTCTGTATTTTA

ATGGATTTTTCACTGTTTCCCCCACTGAAGAGGAAGGTCTCAACAGCTCGAATGAAATAT

TGTTTTTTGCTGACGTTATGGTGGTCTTTGTTTTTCCATGTTGTGCAATGATCTTTTTGT

ATTTTAGAATTTTTGCCATTGCCCAAAGACACACGAGAGAAATCAAAAAGGTTAACAATG

CCATGAGACTTAAAGTCGTAGACTGTGAGATTGAGCTTGATCAGAAATCTGAGAGAAAAG

CTGCTAAAATTATTGGAATTGTGGTGTTTGTTTTTCTTTTGTCTATGGTGCCATATTACA

TCTCTACTTTATTGTCGGGAGCTGTTAATGATAAATTGCTAAATATTATTGTGATCTACA

CATCATCATTCTTTTTTCTAAATTCTTTGTTCAATCCATTCATTTATGCTTTGTTTTATC

CATG

>TAAR-CL337.Contig3_All

CTCTACCAGCCACTGGGCATGTGATCCTGGATTTATTGTGCAATCTTGAGGACATCTTAG

ACATTTGTCATGACCGAGAACAAAATCCTTGTTTCTATGCGCACTAAAAGAAGTAATTTT

ACTGTTCATTGTTCTGCTTCCCTTGGATTACTCAGACCATCAGAAACTGCAGTGCATGTG

ATACTGTACTTGATTTTGACTGCTGTTATCCTTTTAACTGTGTGTGGGAATCTGCTGGTT

ATTATTGCTGTGTGTCACTTCAAGGAGCTGCACACACCAACTAATGTGCTCATCCTCTCT

TTGGCCTTTTCTGATATCCTCACAGGGCTCACAGTAATGCCATTTCAGTTTGTTTGGTTG

ATTGAATCATGCTGGATTTTTGGAGCGGCAATGTGGACACTTTACAGCTTTGCAACTTTT

GTA

>CL6517.Contig1_All CTGCCTAAACTGGATCGACATAATCTTCAGGACAGGGAGCTTTATTACCAGAACTGGGCA

CAATACAATGGAGGACCTCTGGAAAGCTAATTACTGTTATCCATCCTTGAATTCTTCATG

TGCAAAGTTGAATAGGTCACCAGCGATTTATATAATGTTGTACATACTTCTATCATCAGT

ATCTCTGTCTACCGTGGTTTTGAACTTGTTGGTGATCATCTCCATCTCTCACTTCAAGCA

GCTTCACACTCCGACCAACCTTCTCATCCTCTCCATGGCTGTGGCCGATTTATTGATTGG

ACTGATTGCCATGCCTGTGGAAGGATCACAAACAATTGAAACATGCTGGTATTTTGGGGA

CACATTGTGTAGTATATACCCGCTCATTTGCTCCGTGGCTCTGTCAGCATCTCTCTGCAG

TCTTGTCTTAATCTCCACCGATCGCTTCATTGCAATAACTGACCCTTTGAGGTATTCTGT

GAATGTCACACTTAACAAAACAAATGCTGTTGTTGTTCTTGGCTGGTCTTCTTGTGTAGT

TTATTATTTTTTCTTCTTGAATGACCACCTTATGCAAAGGGAACCACACAGAATATGCCA

CGGGCACTGTCTGCCCACAATTAAATTCCCATGGATTGTTTGTGATATTATTGTGTCGTT

CATTGTACCTTGTTCCACTGTAATAGTTTTGAACCTGAAAATATTTTGTACTGCTCACCA

TCAGGCACAAGCAATCAGCTCTGTTACAGAAGGTGCAAATGCTGCTCAAGAAAACAAAAT

GACTAAAACATCCAACAGAAAAGCAGCAAAAAACATAGGCACACTTGTTACAGTTTATTT

ATTGTGCTACATGCCATATTTTATAAGTGTTTTTGCATACATTCATCCTTCCCTGGTGAC

CGGTCTGATATGGATTATGTATATGAATTCTTGTATAAATCCAATAATATATGCACTTTT

CTACCCATGGTTTAAACTATCAGCTAAGCATATCATTACCCTTGCAATATTTCACAAGGA

TTCCTCTTACCTAAATGTTCTGAAGTGAAATAGCTTTTTATACTTGACTTATTTTTTATG

TGGTCACTTGTTGCATTTTCGTTCTCTAATGTAAATCTTATTTGGGCCAATACTGGTCCT

GGACGGTTCTTGAAAAGGGATAACTAAGATTTTAAATCAATGTGATAGTTAACTACT

>CL6517.Contig2_All CAGACATGTAGTAATACCCTGTCATGATTTCTTATTCATATGCCACATGCTGTTAAATAC

ACTGCATTTTCATAGTGAACTGTGTTTTGCTGGCATTTTGTATTTATGGAAATAAGGGGC

GTACAATCTGTGGAATACAATGTTGCTCAATATTGCTTTCCGTCAAACAACTTATCCTGT

CTTAGGGAGATGCGGACTAGATCTGAAACGGTCATCATGTACATCTTCTTTTCAGCACTT

TCAGGCTGTACTGTGTTTCTTAACCTGCTGGTGATCATCTCCATCTCTCACTTCAAGCAG

CTTCACACTCCGACCAACCTTCTCATCCTCTCCATGGCTGTGGCCGATTTATTGATTGGA

CTGATTGCCATGCCTGTGGAAGGATCACAAACAATTGAAACATGCTGGTATTTTGGGGAC

ACATTGTGTAGTATATACCCGCTCATTTGCTCCGTGGCTCTGTCAGCATCTCTCTGCAGT

CTTGTCTTAATCTCCACCGATCGCTTCATTGCAATAACTGACCCTTTGAGGTATTCTGTG

AATGTCACACTTAACAAAACAAATGCTGTTGTTGTTCTTGGCTGGTCTTCTTGTGTAGTT

TATTATTTTTTCTTCTTGAATGACCACCTTATGCAAAGGGAACCACACAGAATATGCCAC

GGGCACTGTCTGCCCACAATTAAATTCCCATGGATTGTTTGTGATATTATTGTGTCGTTC

ATTGTACCTTGTTCCACTGTAATAGTTTTGAACCTGAAAATATTTTGTACTGCTCACCAT

CAGGCACAAGCAATCAGCTCTGTTACAGAAGGTGCAAATGCTGCTCAAGAAAACAAAATG

ACTAAAACATCCAACAGAAAAGCAGCAAAAAACATAGGCACACTTGTTACAGTTTATTTA

TTGTGCTACATGCCATATTTTATAAGTGTTTTTGCATACATTCATCCTTCCCTGGTGACC

GGTCTGATATGGATTATGTATATGAATTCTTGTATAAATCCAATAATATATGCACTTTTC

TACCCATGGTTTAAACTATCAGCTAAGCATATCATTACCCTTGCAATATTTCACAAGGAT

TCCTCTTACCTAAATGTTCTGAAGTGAAATAGCTTTTTATACTTGACTTATTTTTTATGT

GGTCACTTGTTGCATTTTCGTTCTCTAATGTAAATCTTATTTGGGCCAATACTGGTCCTG

GACGGTTCTTGAAAAGGGATAACTAAGATTTTAAATCAATGTGATAGTTAACTACT

>CL6517.Contig3_All GTTTATTATTTCTTCTTCTTCAATGACCACCTTATGCAAAGGGAACCACACAGAATATGC

CATGGGGACTGTCTGCTCACAATTGAATTCCCAATGATTGTTTGTGATATTATTTTGTCA

TTCGTCGTACCTTGTTCTACTGTAATAAGTTTGAACCTGAAAATATTTTGTACTGCTCAC

CATCAGGCACAAGCAATCAGCTCTGTTACAGAAGGTGCAAATGCTGCTCAAGAGAACACA

ATGACTAAAACATCCAACAGAAAAGCAGCAAAAAACATAGGCACACTTGTTACAGTTTAT

TTATTGTGCTACATGCCATTTTATATGACTGCTTTTGCATACATTCA

>Unigene103024_All CTAGACCACTCCATCCCTGAGCTTTTGTATGAGAGTTTAATGTGGGTTGCGTACATAAAC

TCATTGTTTAACCCCATCATCTATACCTTCTCATATTCTTGGTTTAGAAAAAAGTTTGCA

ACCCTATTTAAAAGACAATTTTAAATTGGGTACTGTGTGTTCTATTCTATTATATTCTGT

TATATGATATAATATAAGGAAATGCATGGGGGCATTACAGCGTTAGTGAAAACCATGGCT

TGATGGGTGGACTCATGGAACACAATAGATAACGTTATCATTATCGCCTTGCC

>Unigene121087_All GTATTACTGAGGGGTTAAGTTGATACATGTTTATGTGTTTTGTGCTTTTAGCAGATATGG

ACGAATCACTAGCTTTACAGTATTGTTATCCTTGGAATAATGCATCATGTATCAAAACTG

TGAGGCCCACGTATGAGTATGTTGTAATGTTTTTCATTTTTTCTGTGATATCGGCAACCA

CTGTGGTAGTGAATCTGCTGGTGGTCATTGCGATTGCTCACTTCAGGCAGCTGCACACTC

CCACCAACCTGCTCATTCTCTCTCTGTCTGTG

>Unigene1931_All CAGACATGTAGTAATACCCTGTCATGATTTCTTATTCATATGCCACATGCTGTTAAATAC

ACTGCATTTTCATAGTGAACTGTGTTTTGCTGGCATTTTGTATTTATGGAAATAAGGGGC

GTACAATCTGTGGAATACAATGTTGCTCAATATTGCTTTCCGTCAAACAACTTATCCTGT

CTTAGGGAGATGCGGACTAGATCTGAAACGGTCATCATGTACATCTTCTTTTCAGCACTT

TCAGGCTGTACTGTGTTTCTTAACCTGCTGGTGATCATCTCCATCTCTCACTTCAAGCAG

CTCCACACCCCCACCAACCTGCTGATTCTCTCGCTGGCTGTGGCAGACTTTGTTGTTGGG

CTTATTGTCATGCCCATTGAAAGCATTCGAATGATTGAAAACTGCTGGTATTTTGGTGTG

ATCTTTTGCTCGCTGTTTCCATTTATTCTTTACTTAGTTGTCTCTGCTTCACTTGGTAAT

TTGGTTTTTATATCAATTGACAGGTTCATAGCAGTAAACGATCCACTGAGGTATAATTCC

AAGGTGACAATGCGAAAAACAATGTTCTGTATATTCCTTAGTTGGTTTTGCTCTATTGTA

TATGCTGTGATTCTTTTATATGATCATTTATGCAACCCAGTTGTTCACAGTTCATGTGTT

GGGCAGTGCACAGTTGTTTCAGACTACGCAGTAGCGATGACTGACCTCATAGTCACTTTT

CTCAGCCCGTGCATGGCCATGATTTCTTTGTATGTCAGGATCTTTCACACAGCTAGAAAG

CAAATTAAACTCATCACTGCTGCAGCGTCTAATGTCTGTCAGCTGCACTCTAAAGCTGTG

GTAGCAAAGAAATCTGAGCACAAGGCTGCAAAAACATTAGGGGTTGTAGTGTTGATGTAT

TTAATTTGCTGGATTCCATATTACATTTGTATCCTCACAATGAGCACTATCTCAACATCG

TCACTCACGATAAACATATTTAGCTGGTTCATGTACAGTAATTCTTGTATAAATCCCATC

ATTTATGCTTTCTTCTATCCATGGTTCAAAACGTCTGCACAACATATCTTAAGCCGTAAA

ATACTAGACAAATCCTCGTCTTTTAGGAACTTGTTTCCAGAAAATTCATAGTTTACACAT

GTATTTGGAATTGAAACTCCTGCCTGAAATTGGTGTATTGTGTATACTCCACTGTTTAAT

ACTGAGCCTGCCTTGCAGACTGTGCAAAAGGCCTTGCTCATTAAAAGATGAAAAAGATGA

AAAAATGTGTGTGACAAGCAATTCACTGATTTGGAAAAAAATGTATTGGCTACGAATCTA

ATGGATATTGCTGTGGATATACAGTACTGTATATTTACTCTAGAACATGCTGCTACTCTA

CTGCTGTACATGGCTAAAATGAGCATAAGATGATGTACTGTAACTGTATTGAGCAGGTGA

CTGTGTTGGTCAGGATTCTGTGCTGGTAAGCTCTTGTGACTGAGACAGTGAACTTTCATC

TCTCTCTTAACATTTCAGTCATCCAATTCTGCTTTCCTCATGGAAGTGCACTACCTTAAA

AAGAGT

>Unigene38592_All GAGTAAAGCCATAGTGCCGCAGAAGTCTGAGACCAAGGCTGCAAAAACACTGGGTGGACT

CATAGCAATTTACTTCCTCTGCTGGACACCTTATTTTGTTGTGAGCCTGACTGCAGAAAC

TCTACCATATAACCCAACTGTTTTACATGTTACATACTGGTTTTTGTACATACATTCCTG

TATAAACCCTCTGATGTATGCACTGTTTTATCCCTGGTTTAGAATATCAGTGAAGCACAT

TTTAACCTTGTCAATACTTCATTCATCATCTTCATACT

>Unigene48722_All GTGCAACAGTCGTTCTTTTAAAGAAGTGCAGAAGCCTGACATGTCACTGAAAGGCATCTT

GAACAAGTCAGAGGCTGCAGCCAACGTTGCTCTCTGCTATGACGCTGTGAACAGCTCCTG

TGTGAAGTTCACATATCCGCTTGCTATTCGTGTTCCTCTCTATGTGTTCCTTATGACTAC

AGTGGCACTAACAGTGGTTGGCAACCTGCTGGTCATCATTACTGTGCTCCACTTCAAACA

GCTCCACACACCTACAAACTACCTGATCCTGTCTCTGGCTGTGGCAGACCTGCTTGTAGG

CAGTGTTGTGATGCCCCCTAGTGTTGTGAGGGTGGTGGAGTCATGCTGGTATTGGGGAGA

ACTGTTTTGCAAAGTTCACACAAGTACATCTATGATGTGCTGCGCTGCATCAATTGTAAA

TCTTACAATGATATCTGTTGATCGCTATTATGCCATAAGTCAACCACTGCTGTATAAGAC

CAAGATAACTATTTCAGTTGTGTTTATAATGAATTCAATTACATGGATTATCTCTGCCTT

ATTTGGTTTTGGGGTGGTCTTTCTAGAGGTAAATCTTTTAGGCATTAGGGACTTGTATAA

CAGCATTGTTTGCAAAGGTAGCTGCATTTTTTTACACAGTTTGCTTTCAAGTACTTTGTC

ATCTCTACTGTCTTTTTACCTGCCAGGTATCATAATGGCCTGTATATATCTTAAAATATT

TGTGCTTGCGCAGGAACAATTAAAATCAATTCACACGATAAATACAAATGTTAGTGGGAG

GCCATCAATGACTAAAACTGAAAGAAAAGCCACCAAAACTCTAGCTATTATCATGGGTGT

TTTTATATCATCGTGGATTCCTTTTTTTCTTGTTTTTACTATTAATCCGTACTTTGGCTA

TGGAAGTCCAAAAATTTTATTAGAAATTCTTGGTTGGGTCGGTTACCTAAACTCCACAGT

TAATCCTTTTGTATATGCCTACTTTTACCGGTGGTTTAGGCAAGCATTTCGAATCATTAT

GTCCGGCAAAATCTTCCGACAGCATTCATCCAGAACTATCCTGATCTCGAACTAAACAAC

TTTGTGTGTCACAGAAGAACCATCTTATATACCATCTTTATGCAGTTATACCATCTTTAT

GCAGTTCACTGTTTAAAACACATGACTGATATCCATGGCC

>Unigene75350_All GCGGACAGTAACACAAAGCGTGTGGAGATTTCACTAGACTTCACACTGGAGAATGAAGTG

GCCTTCATCGCAATGGGAATGCACACAGAAGTCATTTACTGTTTTCCAGCACTGAATGGT

TCTTGCAAAAAAGAAGGCAGGACACAAGGGGGGTACATAATAATGTACATACTGTTGTTC

ATCATATCAGCATCAACAGTGTTTTTGAATCTACTGGTGATTGTTTCTGTGTCCCACTTC

AAGCAGCTTCACACTCCG

>Unigene91808_All CTGATAGTCTCCATGTCTGTGGCTGATTTCCTGTTGGGTGTAATTGTAATGCCTCCGGCC

ATGATTGAGTTTTTGGAAAAATGCTGGTACTTTGGTGACGTACTGTGTAAATTTCACACT

GCTGTTGACATCACCCTGTGCAATGCCTCAGTTTTACATCTCACCTGTATATCCATTGAC

CGCTTCTGTGCGGTGAGTCAGCCGCTTCAGTACAAAAAGAGGATGACAATGTGTGTGGCA

TTCATCATGATCTCTGTCAGCTGGGTTTTGTCTGCCATGTTTGGATTTATAGTGATATTC

CCTCCTGCTAACACCACACATCAGGACACTGAAATAACTCAAGAAAACTGCATAGGAGGT

TGCTCAGGCCTACATGAAAAAGAAGCAAGCAACGTGTCATACTTCTTTATTTTCTATTTT

ATCCCACTGACTGTAATGTTGAGCCTTTACCTCAGGATTTTTGCCATTGCAATAAGACAA

GCTCACACTATCCACGTCAACAAAGAATCAAACAGAGATGGCAAGCACAATATGACCTTA

GCAGATTACAAGGCCACAAAGACACTTGGAATTGTCATTGGTGTGTTTCTGTGTTGTTGG

ACTCCATTTTTTATATGCAATGTCATAGACCCAGTAC

>Unigene92362_All CTTATTGTCCTTTTACATCCCTGGTGTGATAATGCTGTGCATCTATCTGAAAATATATCT

CATTGCACAACAACAAGTGCGATCTATTCACAACAAACATATGCAAAGCAAGTCTTCGGC

AGCTGCGAGTAAAATGGAAAGGAAGGCCACCAAAACCCTGGCAATTATTATGGGAGTGTT

TCTGTCTTTCTGGCTGCCATTTTTCCTTTGTAACATCATCGACCCTCTTACTGGCTACTC

CATTCCTCCGCTGTGGTTCGACATGGTTGTGTGGATTGGGTATTTCAACTCTACATGTAA

CCCTCTTGTATATGCTTTGTTTTACAGCTGGTTTAGGAAAGCATTCAGAATCATTT

>Unigene93314_All

GGAAAATCCAATTAACTTTACTGGCAAAGTATACCCAGAGGCTGCTAAAAATGAACATCT

CTCTATCTGCAAATGTGGAATTCTGCTATGAAGCACTTAACGTCTCATGCACCCGGATGA

CGTACCCTTTATCCCTACGCCTGACATTTTACTTTTTTTTTGTGTCAACGATCATTGCCA

TTGCGGCGGGAAATCTTCTTGTTATGTGTACTATTGTGCACTTTGAGCAGCTGCAAACCC

C

>Unigene95434_All

GGGGCATAGGTCTGGCTGGTACAGCAGAGAGCATCCCCCTTTGCCATGAATTCCTGAACG

GTTCCTGTCCAAAGTTCACCTACCCAACAGCATTTCGTGTTCCTCTCTATGTGTTCTTTG

GGACCTCCGTGGTCTTAACAGTGGTTGGCAACCTGCTAGTCATCATCACTGTCGTTCATT

TTAAACAGCTCCACACCCCAACCAACTACCTTATTCTCTCTCTGGCTGTGTCTGACCTAT

TGCTGGGAGGCTTTGTCATGCCCCCTAG

>CL5101.Contig1_All GTGAAGACAGCAATGGCATCGGTTTAATTTTATAGATGAAGTGGTGTAAAGCCATATGTT

GAATATTCTTTTTCTGATATTATATATCAACTTAACGGTCAAACATCAGGATCAATGATT

CTTAATGAAGTCAACATAACAGGTGATTGCCTGGTGTCCATCAACTCAACCTGTTTAAAG

ATTTTTTCTTCCATGGCAACTTATGTGTCACTGTATGCATTTGCTGCAGCTATTGTGATA

TTTACTGTGTGTGGAAACCTTCTGGTAATAATTTCTGTGTATCACTTCAAGCAACTTCAT

ACGCCAACCAATGTCCTCATTCTGTCTCTGGCTATTTCTGACTGTCTCGTAGGTTTATTT

GTGATGCCAGTACATTCAATGATTTTGATTGAATCATGTTCACTGTTTGGGTCAAACTTT

TGTGCCTTATACAACATGATTAATTTTCAGCTAACGTTTGTGTCTGTATACAATATTTCT

CTTATTGCCGTGGACCGTTACTTTGCCTTAAGCAACCCTTTTGTCTACTCTAAGAAGGTT

TCACTCTTTATATCTTTTATTATAGTAATACATGTCTGGATCGTGTCACTGTTTTATAAC

TGTGCACTTCTTTACTTTAATGGAAACTTCAAGAGCAAATGTCCAGATAAATGTGTTCCT

TATGTGAATGAGGTGTGGTCTACTGTTGATCTGATGGTTGTTTTTATGTTTCCATGTGCA

ACCATTATTATTCTGTACCTTAAAGTCTTCTTCATTGCAAGAAGGCATGCAGTCCTCATT

AGAGCAGCACAGAAGGACAGAAAAAGACTGGATGTCCAGAACAGTGGCGATTCAATGAGA

CTGGAGAGGAAAGCAGCCAGAGTCCTTGGGATTCTTGTATCAGTCTTTCTGATGTGTTTA

ATACCCTATTACATTTGTATGATGTTAATGGACGCGTTAGCCCAATCATATGATCGTGTG

GTAAATACCATGTTGACACTATTCTTTCTTAACTCCACTGTCAACCCCATAATTTATGCC

CTATTCTATCCTTGGTTTCAAAAAAGTATGAAACTAATTTTCACATTTAAAATATGCAGA

ACTGACTCTTCTTTGATAAATGTACTCTCAAGTGACTCTTGATTTTTGTCTTTCGTCATG

CATTTGCAGATGTTATTTAGTCGCCATAGTTTCATTCACTGTTTTATATGATTTGAAATA

CCCACCCAACAGGCCCCCATAAAAATACCTATTCTTTTTACGTATGTTAAGTAACTGTAT

CTGAAATCATCAGTTACATTATCCTGAACCAAAGTGTGAGCAATATGTAAAACTTGGCCA

TATGTGAATGATTTTTGCTGTATCTCTGTTGAATTGAAAATTAAAGCAAGTGCTTTTTCA

TTAAAATAGTC

>CL5101.Contig2_All TATATATCAACTTAACGGTCAAACATCAGGATCAATGATTCTTAATGAAGTCAACATAAC

AGTCTTCTTCATTGCAAGAAGGCATGCAGTCCTCATTAGAGCAGCACAGAAGGACAGAAA

AAGACTGGATGTCCAGAACAGTGGCGATTCAATGAGACTGGAGAGGAAAGCAGCCAGAGT

CCTTGGGATTCTTGTATCAGTCTTTCTGATGTGTTTAATACCCTATTACATTTGTATGAT

GTTAATGGACGCGTTAGCCCAATCATATGATCGTGTGGTAAATACCATGTTGACACTATT

CTTTCTTAACTCCACTGTCAACCCCATAATTTATGC

>CL6297.Contig1_All CATGAGTCACATAAATGGTGAACATCTCTAATCCTGACTTCACTTTTTTTGCATTACATA

CCTCATTGGTTAGGCAATATAATGATAGAGGCAACTGCCTGATGTCCCTTAACTCTTCCT

GTTCAATGGCATATGATTCTGTAACAGCTTACGTGTTATTGTATGCATTTGCAGCCGTTG

TGGTGTTGTTTACAGTGTGTGGAAACCTTTTTGTAATCATCTCTGTCTGTCATTTTAAAC

AACTCCATACGCCAACCAATATCCTCATTCTCTCGCTGGCTTTGTCAGATTTTCTGGTTG

GTATATCTCTGATGCCTGTACAGTCAAGGTTTTGGATTGAGCCGTGTTCACTGTTCGAGC

CATCACTTTGTACCTTTTTTTACATGATTGGTTCCCATTTAACTTTCGTCTCCGTTTATA

ACATTTCTTTTATTGCAGTAGACCGCTATTTTGCCTTAAGCAACCCTTTTCTTTACTCCA

AGAAGGTTTCACTGTGTTTAGCATGTATCATAACAATTAATGTCTGGATTGTGTCACTGT

TTTACAACAGTGCACTTCTTTACTTCAACGGGAACTTCAACCATAAATGTCCAAGTAGAT

GTCTTGCCTACATGAATGAGCTGTGGTCAACTGCTGACCTAATAGCTGTTTTCATCCTGC

CATGTGTTATTATTACAACCCTGTACATGAAAGTTTTCTTCATAGCTAGAAGGCATGCTG

TTGCAATCAGGGCCACACTGAAAGACAGAAAAAAAATAGGCGATCAGAACAACGGTGACA

CAATGAGACTGGAGACTAAAGCTGCCAAAGCTCTTGGAACTGTTGTGTCAGTCTTTCTGA

TATGTTTAATACCCTATTATATTTGTACATTGCTGTTTGATGTTTTAGGTCAGTCATACC

ATCGTGTTGTGAACATCATGCTGTTCCTCTTTTTTGTGAACTCTTCCATCAATCCCATTA

TTTATGCGCTGTTCTATCCTTGGTTTCAGAAAAGTGTAAAACTAATTTTCATATGTAGAA

TTTGCAGGACAAGCTCATCACTGATAAATGTGCTCTGAAATGGCCGTTGACTTTTTAATG

AAACAGTTATCACTATGGCATAATAATGTACTAGTGTAATTGTAACTGTACTGTAATTTT

TTGTTTTACTTAAAAAAGCGATGCCTTCCAATTACTATACAAGTACACACAAGCAGAAAT

TTAATTTATGACCAATACAACTATCACAATGAGATTTTAATGGTAAACCAAAAAAATGTC

ACATGCATGTAATGCATAATGTAATGTGTAATGAGGTCTGTGTACTAGTTGTGCCGCAAA

CATTTTAAAACAAGGCAAACAAAATTCTATGTAATGTTAGCTAGGAATGTATGTTTAAGT

GGACTAGCTGTGGGTAGCGAGTTGGTCTTGGGGTTGGAAGGTTACAGGTTTGATTCCTGT

ACCAGTAAAAAAACATTGATGGGGACTGAATAACCAGCACTGTCCTGCACCCTCATCCTT

GGCTAAAATGCTGTTGAGCAAGGCATCTCAACCCCATGTTGCTCCAGGGTGACTGTCACT

CCCAGTAATAGTCAGGTAGTTCATGTTATATTTCTGATAATACAACCATAGATAGATATA

AGATCATTCAAAATATAAAATCTAATTCAGGATTATATGTACTGCATGTAAACATTATCT

AAATAATCATGTATTCCCTGACAATAAGTGTTTTTATTTCTGATACTGTGGCCGTGACTT

TGTATGTGATATGAAAGTAGCATACTGATTGATAATAAAGTGAGGTACTTGGTGTTTTAT

CTCAGAACAAAACCATAATAGGTGCAAAAGCATAAAGAATTTACAATGTAACTCAGATTT

ATAGATACGGTATGTAACCTATACATCTAAATAAAC

>CL6297.Contig3_All

CATGAGTCACATAAATGGTGAACATCTCTAATCCTGACTTCACTTTTTTTGCATTACATA

CCTCATTGGTTAGGCAATATAATGATAGAGGCAACTGCCTGATGTCCCTTAACTCTTCCT

GTTCAATGGCATATGATTCTGTAACAGCTTACGTGTTATTGTATGCATTTGCAGCCGTTG

TGGTGTTGTTTACAGTGTGTGGAAACCTTTTTGTAATCATCTCTGTCTGTCATTTTAAAC

AACTCCATACGCCAACCAATATCCTCATTCTCTCGCTGGCTTTGTCAGATTTTCTGGTTG

GTATATCTCTGATGCCTGTACAGTCAAGGTTTTGGATTGAGCCGTGTTCACTGTTCGAGC

CATCACTTTGTACCTTTTTTTACATGATTGGTTCCCATTTAACTTTCGTCTCCGTTTATA

ACATTTCTTTTATTGCAGTAGACCGCTATTTTGCCTTAAGCAACCCTTTTCTTTACTCCA

AGAAGGTTTCACTGTGTTTAGCATGTATCATAACAATTAATGTCTGGATTGTGTCACTGT

TTTACAACAGTGCACTTCTTTACTTCAACGGGAACTTCAACCATAAATGTCCAAGTAGAT

GTCTTGCCTACATGAATGAGCTGTGGTCAACTGCTGACCTAATAGCTGTTTTCATCCTGC

CATGTGTTATTATTACAACCCTGTACATGAAAGTTTTCTTCATAGCTAGAAGGCATGCTG

TTGCAATCAGGGCCACACTGAAAGACAGAAAAAAAATAGGCGATCAGAACAACGGTGACA

CAATGAGACTGGAGACTAAAGCTGCCAAAGCTCTTGGAACTGTTGTGTCAGTCTTTCTGA

TATGTTTAATACCCTATTATATTTGTACATTGCTGTTTGATGTTTTAGGTCAGTCATACC

ATCGTGTTGTGAACATCATGCTGTTCCTCTTTTTTGTGAACTCTTCCATCAATCCCATTA

TTTATGCGCTGTTCTATCCTTGGTTTCAGAAAAGTGTAAAACTAATTTTCATATGTAGAA

TTTGCAGGACAAGCTCATCACTGATAAATGTGCTCTGAAATGGCCGTTGACTTTTTAATG

AAACAGTTATCACTATGGCATAATAATGTACTAGTGTAATTGTAACTGTACTGTAATTTT

TTGTTTTACTTAAAAAAGCGATGCCTTCCAATTACTATACAAGTACACACAAGCAGAAAT

TTAATTTATGTCAAACCTAAAGTAGGATGTGAGTGACTAATACAACTATCACAATGACAT

TTTAATGGTAAACCAAAAAAAT

>Unigene14363_All

TGTACATTTAACAACTTTAATCGAATCATGCTCACTGTTTGAGTCAACATTTTGTGCTTG

GTACAGTACAATTACGTTGCTGTTATCTTTTGTCTCTATATACAATATTTTATGCATTGC

TGTTGATCGTTATTTTGCTTTGAGTATGCCATTTGAATATTCCAAGAGGATGTCTGTCAA

TGTAGCTGTCATTTTGACAGCAAATGTCTGGATTTTGTCCCTACTTTATAACTGGGCACT

TCTGTACTTTAATGGTAACTACATCCGCAAGTGTCCAGGTAGATGCCTGCCTTCTGTAAG

TGAGACGTGGTCTCAAATCGATCTTGTTCTTTTCTTTATTTTCCC

>Unigene42548_All

ACAAAAAAAAGAATGCATGACCAGAATAATGCTGACTCAATGAAATCAGAGAGAAAAGCT

GCCAGAGTTCTTGGTATTCTTGTGTCAGTTTTCCTAGTATGTTTAATACCCTTAAATATC

TGCACTTTTCTCATAGATGAATTGGGTAAATCATTTGATTATGTAATGAATAACATGTTA

ACCCTCCTTTTCCTGAATTCCACCG

>Unigene78742_All

TTCTTATTCAGTTTCTAGGCAGCTCATCACTGAGCCAACATACCTAGGAACAAACACAAA

TTTTGGATTCAAGAATCGAAGCCTTAAGGATAATTGTGTTTCATGCCTTGGTGAAGCAAT

GGTTACTCCAGAATATGTCACTTTCTATGTATGTGCAATACTTGTTGTTATAATTACTGT

GTTTGGCAACCTGCTTGTCATAATGTCTGTGTGTCACTTCAGGCAGCTGCATACGCCGAC

TAATATGCTCATCCTCTCCTTGGCTGTGGCAGATTTTATGGTGGGACTGTTTGTGATGCC

ACTGGGATTCATCTGGATGATAGAAGCATGTTGGTTTTTTGGGGTTTCAATGTGTGCTTT

TCTAAACTTTTTTGCATTTCAGTTGACAACTGCCTCTGTACATAATGTGGCTCTTATTGC

AGTGGACAGGTATCTGGCGCTGAGCAATCCCTTTCTGTATACCAAACAAATAACTGTGAG

ACTTACCATCATTGTGGCCTTGTTGACCTGGGTGTTTTCTGTCGGTTACAACTTTTCTCT

TCTGTACTGCAATGGGTTTTTCACCACCTCACTGACACTCTGTCCAAATGAGTGTCCTGT

TGCTGTGAGTGAAGCGTGGTCATGGGTTGATTTTGTTGTGGTATTTGCATTACCATGCTC

CATCATGTTTGTATTGTACCTGAAAATCTTTGCCATTGCAAGGAGACATGCCAATGCAAT

CAGGGCAGCTAATAGTCAGGGTAATCCAAATGCCAAACAGAATGACAATGTACCCAAACG

ATCTGAGAGAAAAGCTGCTAAGGTGCTTGGAATACTCGTGTTTGTCTTCTTGTTGTGTGT

TGTGCCATATTACATCTCTAGTGTTATGGCTGAGTTTATCCATAGTCATATATTTGAAGA

TATTTTAAATTACACCTCACTCATACTTTACCTGAATTCTTTATTTA

>Unigene84000_All

GGACCACAGAGTGTCTCTTGCTTCCATCACAGGAGGCAAAGAAAAATGGACACGGACGTG

TCAGCTGATCAATATTGCTATCCGTCCGTGAATGGCTCTTGCACGAAAGGCGTATACAAC

CAGGCGACGCAAGTGGTTCTGTACATCATGTTCTGGACCAGCATGGCAGTCACTGTTATG

GGCAACCTGGTCGTGATCATCTCCATAGTGCACTTTAAACAACTGCACACACCGACAAAC

ATGTTGGTGATGTCCCTGGCACTCGCAGACCTGCTGCTGGGACTGACTGTGATGCCTTTC

AGCATTATTAGATCTGTAGATGGGTGCTGGTACTATGGTGATGCTTTTTGTTTGCTTCAT

TCCAGCTTTGACATGTTTTTAACGTCTGCCTCTATTTTTCACCTGATATTTATAGCCATT

GATCGACACCAGGCTGTTTGTAACCCTTTGTTGTATCATACTAGAATAACTCTACCTGTG

GCCTGGCTCATGATAGCTGTGAGTTGGTCAATTGCTGCTGCTTATTCCTTCAGTCTTCTC

TATAGCAAAGCAAATGTTAAAGGGTTCGATGACTTCCTCAGGTCCATATACTGTGTTGGT

AGTTGTAACCTTTTATTTAATGCACTATGGGGGGCACTGGATACTATGATAGCGTTTTTC

CTGCCTTGCTCTGTCATGGTTGGTTTGTATGCAAAAATATTCTCTGTGGCCAAAGGTCAT

GCTAAAAAGATTGAAGATGTGAGCCGAGGCCTTAATATGAATGGTGAACACGTAATCAAG

TCAAACAGCACAGAGCATAAAGCTGCAAAGACTCTTGGCATTGTTGTTGGTGCTTTCATT

CTTTGCTGGTTGCCTTTCTTTGTCAATTCAATTGTTGATCCTTACACAAATTTTTCTACA

CCAGTTATACTTTTTGAGATTTTTACTTGGCTTG

>CL337.Contig2_All

TACAATGTTACTCTCCTTGCTTTAGATCGATTTGTGGCTTTAAACAGTCCATTTTTTTAT

TTGGACAAAGTAACAGTGTATTTAAGCTTCATAATAGCATTCCTGTCCTGGTTGTTTTCA

ATATTTTATAACTTTATCCTTCTTTATTTTAATGGATTTTTCACTGTTTCCCCCACTGAA

GAGGAAGGTCTCAACAGCTCGAATGAAATATTGTTTTTTGCTGACGTTATGGTGGTCTTT

GTTTTTCCATGTTGTGCAATGATCTTTCTGTATTTTAGAATTTTTGCCA

>Unigene14007_All

GGCATCTCTACTGCCTCCTGCATTCCTCTCACCAAAACAACTTGCAACTTTTCAGTCTCT

ATGCGGATGTATATGGAATGACTTTACTTTCAGTGGAAGATCTGCACTCATATAACAGGA

GCTCAGTTAATTTGAGTTTAGTGACTGAACAACATTTTTGCTCGAATCACACGTGTGACA

TTTTTGAGGTGACACCTGTTGATATACTTACCTATGTATGTAAGCTGGCAGTGATAATAA

TGGCGGTGTGTGGAAACTTGCTTGTGATCATCTCCATCTGTCACTTCAAGCAGCTCCACA

CACCAACAAACTACATCATTCTCTCTCTTGCAGTGGTGGACATGTTGGTTGGAATTACTG

TTCTGCCTGGACAGCTTATATTATTAAATTCATGCTTGAAAACAGGCATGATTTTTTGCT

ATTTTACTCTGATATGTACTTACTGCCTCAGTTTCCTCTCTATTTACAATGTGTCTTTAA

TTTCACTGGATCGATTGTACGCTTTATGGAAGCCATTGCTTTATGCAAGTAGAGTGTCTG

TCAAAGTCATGTCTAAGGTCATCTTTGTTACTTGGATAATTTCTTTGGTTTACAATCTAG

GTCTTGTGTATCTAGTTACCACGGGCCGTGATGATGTGTTATGTATTGGGGATTGCAGTG

TAGCACTAAATGAAATTTGGTCAACCGTAGATATTTTTATTGTGTTTGTTTTGCCATGTT

CTATTATAATTGTTTCATATTTGAAAATATTTACTATTGCTAGAAAGCATGCAAGAAGCA

TCTCTGTCAGTAAAAAACAAACCATCCCAAGGATAAGATCTAAAGGCATTACTCTCAAAG

CCTCAGAGAGGAAAGCAGCCTTCACTCTCGGAAAGCTGGTGCTTGTGTTTGTTTTGTGTT

TGCTGCCCCCATTCTTAACCTTAGTATTTAGTGATAGTATCCCGGATGCATTGCTACAGG

AAGTGATGGAAGGAGCTATGCTTGTTCACTACCTGCACTCTGCAATTAACCCATTAGTAT

ATGCTTTCTTCTACCCTTGGTTTCGTAAATGTGCAAAGCTCATTCTCATGCTCAAGACCT

TAAATCCTGACACAGCGCTGCTGGTTGTGCTCTCAAACAATGGATAATTAAATCATACTC

CCCTTATTTCACTCATGCAAGTCATTTTGGTCTAAAATGCAATTTATGTATTTCAGTATG

TATGGATTTGGATGAATATGTATGTATGGATATGTATGGATATGTGTTTTCTGTTAGTCA

TGTACATAAAACGGTAATACCTTTTGACTTATAATGTACTCA

>Unigene22708_All

CTGGATTCACACTTCATTCACAGGGAGCTCTTCGCTGACCCCACAACACTCAGAGGAACA

AACACACTTGGATCCAGCAATCTCAGCCTGCTGGACAGCTGTGTGTCTTGCTTAGGGAAA

ACACCAGTGACTACATCGTATGTGGCTCTGTATGTGTCTGCAGTAATTGCTGTTGTAGTG

ACTGTCTGTGGCAACCTGCTTGTCATCACATCAGTGTGCCACTTCAAACAGCTTCACACA

CCAACTAATGTTCTTCTCGTCTTCTTGGCTGTGACAGATTTTTTTGTGGGGATATTTGTG

ATGCCGCTTCAATTCGTCTCGCTGATAGAATCATGTTGGTTTTTTGGAGCAGTTGCCTGT

GCTTTTTTTAACTTTGTGTCATTTCATTTGCCAACTACCTCGGTCCATATTGTGGCTCTT

ATCGCAGTGGACAGATGCATGGCTCTGAGTGATCCGTTTTTCTATTTTAAGAAAATTACT

GTGAACCTCATCATCATTATAGCCACACTAGACTGGGTGTTCTCTTTGGTTTACTGTTTT

ACCCTTCTATACTGTACTGGTTTCTTTACTCGTGCACTGACACTCTGCCCATACACCTGT

CTCATTGCTGTGGTTGATGAAATATGGTCTTGGGTTGATTTTGTTTTGGTATTTGCATTA

CCATGCTCTATCATGTTTGTATTGTACCTGATCATCTTTGCTATTGCAAGGAGACATGCC

AATGCCATCAGGGCAGCTAATAGTCAGGTTAATCCAAACGCCAGACAGAATGACAATGTA

CCCAAACGATCTGAGAGAAAAGCGGCCAAAGTGCTTGGTATACTCGTGTCAGTCTTTTTG

TTATGTATTGTTCCATATTACATCGCAAACCTTATTCCAGGGAGAATTAATGCTGAAATC

TTTGAGTTAGTGTTAAATATCACATCGGCCCTATTTTACCTGAACTCTTTGTTTAATCCA

ATTATTTATGCTTTGTTCTATCCATGGTTTCGAAAGTGCATGAAAATAATTTTGAGTTGT

CAAATATGCAGCCCCTCATCCTCTGTAATGCATGTAATGTGAGATTGTTTATTTTATGAG

CCCTCAAAATGAAAATAAGACATTGAAACAAAATGCTTGTCTGAGCATGCACGGATTGTT

GCTCTGATATGTGAAATGCGTAATGTGCTATGTTCACTAATGATATTTGTACATGTTTAG

TAGAGAATTAAGAAAAATGCTTTTCCAGTTGCTGGCACATTATGAAATCTGAAGAAGGCC

ACAGGTTTCCTAAAATTAAATATGGAGCCACAGCATGCGCCATCTTTATTGTGTTTTAAG

TAGAGAATTAAAAACATCTCCAATGTGTTTCCAATTTTAATTGCACCCTTATTCATCAAC

ATACTATTACATTATGATTATTATGATTAGGCTACATTTGTTCACATAGATAATTTCATT

TTAATTTGTCATTATTTGCATTACTCTTATTTTCATTTCCTTTTGTGTTTTTATCAACTA

TTCAATTTTTAGTGGTACAGTAGTTATTTTATCAGTGTGAGATGATTGAAAAAAAATCTG

TCATTAAAATATGAGTTGTAAAGTCGGGTTTTGATAAAAGATTGATTGACGGTGTATAAC

TGACTGGTAATAAAACACTGGATAGCATTTTATTTATTTTCTTTTTGCCTTAACTTGAGT

TTTATTACACAAAGTTGCAGTATGCATACATTGGCATCTAAGTAAATGTATGCATATCAC

AGTGAAATACAATATTGTGAAATGGTTCATTTTTTTCATTGATTCAATTTAAAAAAGTGA

ACCTCATATATTATATTGATTCTTCACACACAAAGTGAAAAATGTAAAAGCTTGTTTGCT

TTCATGATTACAGCTTACAGCCAATGAAAACCTCAAAACA

>Unigene2345_All

GAATTCTTTATTTAATCCAATTATTTATGCACTGTTTTATCCATGGTTTCAAAAAAGCAT

GAAATTGATTTTAACGTGTCGTATTTGTACCACAGATTCTTCTCTCATGCAAGTGAAGTA

ATTTTTTCTAGTTATGTCTTTATACAAAAATAATGAATTGAGACAAAGTTGTCTAGTCTG

TAAGCTTTGTTTTCCAGGTATGTTGAAAATGTAAGCAATATATCTTACATATACTGTATA

TACTGTATGTAAATATCAGAAAGAAGGAAAGAAAACATATTATAAAGAGAGTGTTAAATT

AAATTAATTGTAAAGACTGTGAAGGTCCACAGAATTTGCATTGTCTTAATAAATACTGAA

GTCACTATAAGGAAGGATTGTAGGATTAGTAAGGTGGAATTGTGCAGTGGTAATGCAGAC

TAATGCAGACTAACACAGAATATCTAAATATTACTTTAATAAAGTTCTGCGGTGCAATTA

TGTATCAGAATAACATCAAGTTTTTTGCCTGCTTTATATGTTGGACAGCAATGCACCCGT

GTGAATTTAAATGAATTATACAGAGTGTGAAGGTCTACAGAATTTGCATTGTCTAAATGA

GTATTGAAGTCCAAGCATTAGTACAGTATAGGCTTACAAACAGCAAGTTTACTGTATGTC

TTAGTTTACCCAGTATCTCTGTGCATGCACGTGACAATAAAATATCCATGTAAATAAACA

TTAGACAAATAAATGTTTG

>Unigene25026_All

CGAGAGAAATCAGAAAGGTAAACGTCACGAGACCTAAAGCCACATTCCGTGGGCATGATC

AGAAATCTGAGAGAAAAGCTGCTAAAATTATAGGAATTGTGGTGTTTGTTTTTCTTTTGT

CTATGGTGCCATATTACATCTCTACATTACTGTCAGGAGCCATTGATGCAAAATTAGTAA

ATATTATTATGAACTACACGTCATCATTCTTTTATCTAAATTCTATGTTCAATCCATTCA

TTTATGTTTTGTTTTATCCATCGTTTCAGAAGAGCATGAAACTCATATTTACAGTAAACA

GGAACTCATAATGTGAAATGTAATGTCTGGGGTCTTCAGCTGTGTGCCAAATTAAATTAA

CAGTTGTACATATGTGTTGTTTGTGGTAAATAATTAAGTGTGCAAGACAGCCTGACAGTG

ATAAGTGGCCCACAGGACCACATTGGCACCCAAAGATTCTAGAGTGGAG

>Unigene3763_All

CCCCTTTAATGCCATTGTGATCTTGTACTTCAAAGTATTCCTAATTGCTAGAAGGCATGC

AGTTGTAATCAGAGCAACATTCAAAAACAGGAAAAGACTGGAAGACCAGAATAATGCTGA

CTCTATGGCATCTGAGGGAAAAGCTGCCAGAGTGCTTGGGATTCTTGTTTCAGTTTTTCT

AATATGTTTAATACCTTACAATGTCTCCATGCTCCTGACAAATACACTGGATGAATCACA

CGGTCATGTGGTAGACAACACAGTGACCCTTCTTCTGGCTAACTCTACTAT

>Unigene73382_All

GACGCTGTCACCTGCGAAGGTAGCTGTGCTCTGCTATTTAGTTTGTTGTCAAGCACTTTA

GCCTCTGTGCTCACCTTCTACCTGCCTGGCACAATAGTGATAAGCATCTATTTAAAAATC

CTCCTTATTGCATTAGAGCAGGAACAAACAAGTCGGGACAGGGGTCTAAATGTCAGCATG

AAGCCTCCAACCAATAAAATAGAGGCGAAAGCCACCAAAACCCTAGCTATTGTTGTGGGG

GTATTTTTGTCATTCTGGATACCATATTTCCTAATAATTTCATTTGATCCGCATTTTGGT

TACATGATACCACCATTACTGTATGACATCTTTGCCTGGGTTGGTTATCTAAATTCCACA

TTCAATCCAATAATCTATGCATACTTCTACAGCTGGTTCAGGAAAGCATTTCAAATAATT

GTTTCGGGTAAAATATTTCAGAGGGGTTCTTCTAGAATTCTGTTGTTCTAAGATGTCACC

AAAGCAACTTACATTCTAATGTCAATCTCTGAAGAGCACCCTCCTGTGTACTTCATCAGT

ACTACTCCAGGCCCATACATAAAGCTGTCTAGCACTACACATTTAAGCACATTGTGTCGC

GAATATTCAACAGAAAAACAAAGAGTGAAGCAGGCTTTACTCAGTCATTGAGAAACCAAA

GAGTGTGTGCAAAAAATGTTTTCCTTGTTCCCTTGTTCTCATAAATATTGAACCATGTTA

TAATTCACCACATGATGAGGTTTAAGCCTGGCTAATGTTGTTTTATATTATGTTATGTTA

TGTTATG

>Unigene101623_All TACTGCAGCGTATTCCTTCTGGTCATCGTCAGTATAGACCGCTGCTTATGTGTCATCGCT

CCTGTGTGGTCTCAGAACCACCGTACTTTGAGAGGGTCCTCTGTCGTCATTGCTGTCGCC

TGGACTCTTTCAGCACTGATATCTCTTCCCACCCTTGTCTTTTCTACCACACTGGAGCAA

CCTGGCAGGACCATCTGTGTTTATAATGACATCGTTGCTAAAGTGTCGCTGCAAACGTTG

ATTGTGACTGGTTTTGTAGTAGGACTCGCTGTCCCTTTGGTAGTTATAGTCGTTTGTTAT

GCTATTATTATGCAGAAGCTGAGAGCCAATCGTATGTCAAAATCCTCCAGGCCATTTAGG

GTCATGACGGCTGTTATAATAACATTTG

>Unigene66923_All

CAAGAACCAAGCAGTGAAGGTGGGCCTGACTCTGGCCAAGGGCATCGCCTACTTCAACAG

CTGCGTCAACCCAGTGCTCTACTTCTGCATGGGCCTCAACTTGCGCCATCGACTGAACCA

GACGCTGTCGAGCGTATACCGACGTGCTCTAACCGAAGATGGGGATGGGCCGACGACACA

GTCGCAGGAACAAGGCGTAGACGATAGCTCGGCCTCCCTCCCTAGACATGCCCATGCACA

GGTGCAGGGCAAGACATCACCTGGTGTAGTGCCCAATGCTTAATTGGATGGTCCGCAAAG

AAGTATCTTGGTTTTTACTCTCGTCAAAGACCC
