# Supplementary material for: Identification of olfactory receptor genes in the Japanese grenadier anchovy Coilia nasus
Source: Genes Genomics. 2017 Feb 23;39(5):521–32. doi: 10.1007/s13258-017-0517-8 (PMC5387026; doi:10.1007/s13258-017-0517-8)
Supplement: Supplementary file 4 — Amino acid sequences of identified OR genes in Coilia nasus. (DOCX 49 KB) [file 13258_2017_517_MOESM4_ESM.docx]

>CL1061.Contig2_All MENSSFHKFFILNGLQQAGKNKSVYFYLTCTLYILIIAMNVTIIITVRVDKALHEPMYIF

LSNLCVNGLYGTVGFYPKFLIDLLSEVAVISYLQCLTQTIVIHSSAICEMTTLTAMAFDR

YVAICRPLQYHSILTSHMVLKLLLLAWFYPASVSVVTIVLTVRVPICGSLISKLFCDIPS

ILKQGCYPTPVNRIFGIFVLIFQVIQVLLICVSYVQIVRVCLRSRAGRRRFTQTCLPHII

ALFVSILSLLFDLSCSWADTTNLSRDLRNVLGMQFLILPPICNPLVYGLQLPQIRRAILK

TYLKPKVSKNKR

>CL10694.Contig1

ENSSSVSDILVLEELGHPESTTYAVFFTLLFVYIALLITNFGVLIIIIAEKSLHQPMYLL

FCNLPVNDILGNTILLPHLLYDMASRNRLMFYNNCVAQVFFTHVYASASHSILVVMAIDR

YVAICKPLRYNAIMTAKAVLVLSVSAWAFPAVFISVLVGLSVRLSRCRSTIPNFYCDNAS

LFKLSCENVSINNIYGLFYSVLLLGSSMGTIAVTYISIAVTCWTKKSAELNSKAIQTCAS

HLVLYLIMLLTGYVVIIMHRFPEHRFLRKLMAVLFHVVPAHLNPVIYAFQTKHLRVKILQ

IFGRKIT

>CL10694.Contig2_All

YGSASHTILIVMAIDRYVAICNPLRYSAIMTTKAVLTLSFFAWAVSVVLVGVLLSLTIRL

SRCRSHIMHAFCDNASLFKLSCEDVSINNIYGLFYSVLLLGSSMGTIAVTYISIAVTCWT

KKSAELNSKAIQTCASHLVLYLIMLLTGYVVIIMHRFPEHRFLRKLMAVLFHVVPAHLNP

VIYAFQTKHLRVKILQIFGRKIT

>CL10694.Contig3_All ENSSSVSDILVLEELGHPESTTYAVFFTLLFVYIALLITNFGVLIIIIAEKSLHQPMYLL

FCNLPVNDILGNTILLPHLLYDMASRNRLMFYNNCVAQVFFTHVYASASHSILVVMAIDR

YVAICKPLRYNAIMTAKAVLVLSVSAWAFPAVFISVLVGLSVRLSRCRSTIPNFYCDNAS

LFKLSCENVSINNIYGLFYSVLLLGSSMGTIAVTYISIAVTCWTKKSAELNSKAIQTCAS

HLVLYLILLLMGYLIIIMHRFPEERFIRKLMAVLIHIVPGHFNPIIYGLQTKHLKLKIWK

IFRPKTTET

>CL10694.Contig4_All ENISSVSNILVLEELGHPESTTYAVFFTLLFVYIALLITNFGVLIIIIAEKSLHQPMYLL

FCNLPVNDILGNTILLP

>CL13258.Contig2_All YDRFVSIFRPLQYHTIITPWKVKQLMIVANVIPTALILVQIFMTSQLPQCRYDVHRTYCD

NLAVVSLSCLES

>CL6601.Contig1_All MENMSAFKLFVLSGLQESGVYKPLYFLLTLILYALIIAANLTLILNVTMEKRLHEPMYIF

LSNLCVNGLYGTLGFYPKFLLDLQSDVHTITYGWCILQVYVIYTSVMCEITILTVMSYDR

YVAICKPLQYHSILTPHAILKLLLLAWAYPLLTSVVAILLTVRIPICGSLIHKLFCDNPS

VLKLGCSQSIINQIWSIIVITGQLMQYVFIFISYCHIIHVCISSSEGRAKFTKTCVPHIL

VMLIFVTTTLFDVLYSWGESKHFPIWIRNALAVQFLILPPLLNPIIYGFQLPQIRKILCR

QSCKHKM

>CL6606.Contig4_All SGATTMLTLPPFSFPLSARVPALVFSALTYLTIICCNLMVVVTIAINHNLHKPMYMLLIN

LPVCDMMGATAFFPQMISSMLYDPRVIPYWACVTQALFVHTYGAGSLLILTAMALDRYVA

ICQPLSYNTIMTNSNLIKIISAVWLTAMILIFILIVLALRFKICRTNISDMYCNNPSLTN

LACELTYVNNVYGLLTIAFFQGISLCAILFTYLQILITCVFKKQSDARSKAIQTCG

>Unigene115271_All DALQLSLVTALYVVSYAFSRIHASVCSVLIITAVTTTRATPLILAGMAVERYIAICFPLH

YGHMCTLARTLWLILGIL

>Unigene16290_All IKTCASHLVLYLIMLLTGYIVIVMHRFPEHTFLRKLIAVLFHVVPAHLNPIIYGLQNKQL

RLKILQIFGQ

>Unigene18154_All

WAYPLLTSLIAVILTVRIPICGSHIHKLFCDNPSILKLGCFQTTVNKIWGMILITLQLKQ

FVFISISYCYIVHVCVSSSEGRAKFTKTCVPHILVVVIFIATTLFDVLYSWNGSVHFPVI

VRSALATQFLILPPLFNPIIYGFQLPQIRKVLCRQSCNHKI

>Unigene21419_All NISSISDILVLEELGHAESSIYPIFFTLLFLYITLLISNFGVLSIIIVEKSLHQPMYLLF

CNLSLNDVIGNTILVPHLLFDMVSKNRLISYNNCVTQAFFGHTYGSASHSILIIMAIDRY

VAICNPLRYSAIMTAKAVVTLSVSAWAVPAVFVGVLVSLSARLSRCRSLIQNFYCDNASL

FKLSCEDVTVNNIYGLFYTVVLLSSSMGTIAATYIRIAVTCWTKKNAELNSKAIQTCASH

LVLYLITLLTVYILVIMQRFPDNPFLRKFISILFVVVPAHLNPVIYGLHTKHLRLKILQL

VGRKITHS

>Unigene21420_All NISSISDILVLEELGHAESSIYPIFFTLLFLYITLLISNFGVLSIIIVEKSLHQPMYLLF

CNLSLNDVIGNTILVPHLLFDMVSKNRLISYNNCVTQAFFGHTYGSASHSILIIMAIDRY

VAICNPLRYSAIMTAKAVVTLSVSAWAVPAVFVGVLVSLSARLSRCRSLIQNFYCDNASL

FKLSCEDVTVNNIYGLFYTVVLLSSSMGTIAATYIRIAVTCWTKKNAELNSKAIQTCASH

LVLYLITLLTVYILVIMQRFPDNPFLRKFISILFVVVPAHLNPVIYGLHTKHLRLKILQL

VGRKITHS

>Unigene23389_All MENTSTLHFFILHRIQELGRNKLLYFFLTLVLYMAIITLNVTLIVTIVVEKSLHEPMYIF

LSNLCANGLYGTVGFYPKFLMDLLSDNAVISYAMCVTQTYIIYTSAMCEIAILTVMAYDR

YVAICRPLQYHSILTPKSILRLLLLAWFYPLSMAAVSLILTIRLPICGSYIGKLFCDNPS

ILKQACYSAHANRLWSIIIIVGQILQAVLVFASYAQIVHICMGSSKGRTKFTQTCLPHIL

AIFISTLAVLFDVLYSWDGSAELPLHVRNALGLQFLILPPFCNPLVYGLQLPRIRKTFFQ

KLRCSRTKV

>Unigene52959_All TVLLPRLLADMFASKKFTTYAECVTQAFCSHTFGSASHMILIIMAFDRYIAICNPLRYAS

IMTTKTIVKLSVSAWGISLVLVAILLGLTIRLSRCRSVILNAYCDNASLFKLSCDDVSIN

NLYGLVFTAVLFGSSIGSILVTYLRIAIICWTKKSKELNGRALQTCTSHLLVYMIMLWTG

FLTIILHRFPDYPYLRKLAYILFHVVPANLNPIIYGMQTKTLRKKILQTFLRKVSST

>Unigene59895_All

NISYISDVLTMEGLEIAESSTYPVFFTLLIVYIALLISNIGVLAVIIAERKLHQPMYLLF

CNLSVNDILGNTILMPRVLLDIVSNEKLISYSACVTQAFFSHTYGSAS

>Unigene62329_All PMYIFLFNLTCLGLIGSTALWPKVMSNLLSDKQSSSYEACLCQVFIVSVYATGTYSVLTV

MAYDRYVFIIDPLRYHTIMTPQKVKQLLTVSNLVPLCSVSGQVWLTARVPLCSQNIHKFY

CENISVSKLSCVTSSLYRASSLYGVSVFVLSVVFIAFFVLLSYIRIITVSVKASSDSQTK

AISTCAPHLIVFINFSMSSLFSIIYNQFAFVSEDMHMFLSFQFALGPPLLHPIIYGIKNT

DIRKCIVKHVRPVVSYVTCYIKSSHKLSP

>Unigene68575_All SLERYVAIFYPLQRPAAWRADRIWVVIVSMWVLSCVQPTAEFIMSKPRGGAQMDVLTTPV

QCKSAVLHVAPAITLFKVVLNGLFFALVAAVILFTYVRILLGTR

>Unigene68576_All MQNLTSAVNATSDRNLAVIVKVCLVIPIFSVFLYFIVLMLHTFASHRHFLESPRYILFTY

MLANDTLQLLTSVLLFLFVMAQVNFALVFCAPLLFFSTATFLNTPLVL

>Unigene95217_All TVIFTAYQSIGVQKYAYFTIIAALYISSVFTNVFLMLIIIWESRLHQPMYIFLFNL

>Unigene96554_All ASVAKALHTVLLHGLQLLLSTSAFTIPFTEHLIVLHVGWLREHMSFLNYVSFVIVPRVLS

PLIYGLRDESLRRHMQRSPLCCA

>CL10461.Contig1_All MDNTVLNETFSFELKIASYDIHPSAVYPIFAIGMLIYLISVVSNLTILLLIATQKSLHKP

MFYILFSLPLNDLVGITALLPRVLVDIVTQEHTVYYPTCVIQAFLIHMFGGGTLFVLAAM

SFDRYLAICKPLRYHSIMTPLTVAGILLLAWGTDFILILVLFLLQSRIRRCRNVIQNMYC

SNFALLGLSCGEDTTINNIYGLFITAFMHIITITIQLFSYVQILLACVFHRQSDAKTKAL

NTCLAQLIVFLIFEFISFFTITTYRFPNVPSTVRMTIGMMTFVISPFLNPFIYGMKTKDI

RISFIQVVRKRKL

>CL10461.Contig2_All MDNTVLNETFSFELKIASYDIHPSAVYPIFAIGMLIYLISVVSNLTILLLIATQKSLHKP

MFYILFSLPLNDLVGITALLPRVLVDIVTQEHTVYYPTCVIQAFLIHMFGGGTLFVLAAM

SFDRYLAICKPLRYHSIMTPLTVAGILLLAWGTDFILILVLFLLQSRIRRCRNVIQNMYC

SNFALLGLSCGEDTTINNIYGLFITAFMHIITITIQLFSYVQILLACVFHRQSDAKTKAL

NTCLAQLIVFLIFEFISFFTITTYRFPNVPSTVRMTIGMMTFVISPFLNPFIYGMKTKDI

RISFIQVVRKRKL

>CL1076.Contig1_All HMLCNDSIMILFTFLIGIISYVHRPTKAMCSLIMIVTTSTSTNAPLNLGVMSLERYIAIC

FPLHHRELATTKRTYVAIAAIWFFGLVNPVVDSFYNSVTDRDFFTEEILCGSQTIFNTSP

WQALLYQALNGLYYVTVTLVILFSYINVMLVARSVSSDEKSTRKAHRTLLLHLIQLVLCL

NMLLYISIVRFLALVFSFEVYKDVSYVIFLLVILLPRCLSPIIYGLRDKAVRNLFMFYLK

CGFCRAKPKVNI

>CL1076.Contig2_All HMLCNDSIMILFTFLIGIISYVHRPTKAMCSLIMIVTTSTSTNAPLNLGVMSLERYIAIC

FPLHHRELATTKRTYVAIAAIWFFGLVNPVVDSFYNSVTDRDFFTEEILCGSQTIFNTSP

WQALLYQALNGLYYVTVTLVILFSYINVMLVARSVSSDEKSTRKAHRTLLLHLIQLVLCL

NMLLYISIVRFLALVFSFEVYKDVSYVIFLLVILLPRCLSPIIYGLRDKAVRNLFMFYLK

CGFCRAKPKVNI

>CL11354.Contig1_All FYLTGFANLREHRRLLSIPFFFLLLYMMAANAVVTYVIAKQRNLYKPMYVLIGSLTLLNF

FFPLLFIPRMVVSFASGRNDITAVECLLQIFVIHLCGSFQTSILLQMAVDRFFAICWPLR

YHDIVNLRNSILFTAALALRNTVTMVTMVCLFIPLTFCQSNVIYHCACVVTLACGTSLRS

YIAGVMAFTITTSDCIIIAATYVVIFIVIFWSSSGESRQKAIHTCSTHLLVIGISYLSVV

CAFIGYKVSAIPPDIKVALTLSYLLIPSCFSPVIYGIRTNEIKVYMLKMLKLNTVDS

>CL12962.Contig1_All

LSTQMYQVQLNEGPLTKLVVAIIMSLLFIWVNCLLLFTLSRKHIFRETPRYVLFSHMLCN

DSIQLLFTSLLYIFSMSYSQVHRGACYLLLFVSSSTFLNAPLNLALMSLERYAAICFPLH

HGNVATPTATAVGILTIWFLGGVNVVIDILYALTRDGTTLGNHMFCTRERIFMAAWQADA

FQGFNGFYFVIVTLIIVFTYISVMVAARSATSNKESAKKAHRTLLLHLIQLGLCLNSFLY

GIIERAL

>CL12962.Contig2_All DTNSSTEQVLFVHQQMYQVQLNEGPLTKLVVAIIMSLLFIWVNCLLLFTLSRKHIFRETP

RYVLFSHMLCNDSIQLLFTSLLYIFSMSYSQVHRGACYLLLFVSSSTFLNAPLNLALMSL

ERYAAICFPLHHGNVATPTATAVGILTIWFLGGVNVVIDILYALTRDGTTLGNHMFCTRE

RIFMAAWQADAFQGFNGFYFVIVTLIIVFTYISVMVAARSATSNKESAKKAHRTLLLHLI

QLGLCLNSFLYGIIERAL

>CL13258.Contig1_All FIIVFLVYLSSIIANVFLMCLIYLDSALHKPMYIFLFSLLVNGLIGSTAIWPKVMNILST

NSPLISWEGCIAQVFFVLTYGACNYSLLAVMAYDRFVSIFQPLQYHTIMTPLKVKQLMFV

ANLMPTAVMLGQTYIASQLPLCRFEVHKIFCDNLAVVSMSCSRSQLSNLYGIINTILFVV

LPVFFILLSYVKIIVLSLKASTNARKKVFETCSPHIITFVNFFLVSLFSLVYNRYSFLPV

EANIVQSVNYILFPPLLHPIIYGIKTQEIRQSFSKVIKRML

>CL1434.Contig2_All MQNLSRVSVFELTGLNFTSEYKYFLFSLTLLCYPFILMVNITVIYVIVINKHLYEPMYIF

LCNLCMNALYGTLGFYPKFLYDLLSDHHVISYLGCLVQAFVIYSSALCEFSTLTVMAVDR

YLAICRPLEYHRIMTNNMVIMCIVFSWFSPFMCVLVGVLLGSSLTLCGSYIDKLYCENWS

IVKLSCYSTMLNNVYGYIIILVYFIHSLFIMCSYIKLIDTSIRSKEERSKFMQTCLPHLL

SLLNVTVALLFDVMFTRYGTTRNFSQDLRNFLAIEFLVIPPILNPLIYGLKLKKIRNQLM

RFYHRNI

>CL14888.Contig1_All

MNESSISIILVLTAYEDMGVAKYVLLTLVFSTYFASVIANAIMMIFICLDTFLHKPMYMF

LLSLILNGLMGSTAIWPKVMNILLTDDNTISYVGCILQISLMRLYGGCNYTILAVMAYDR

FVAVFNPLNYQTIMSPESVGLLVFAACFIPFCLVLCEAYLLSRMHLCRYTLQRIFCNLDI

SHLSCDIKDSQVARLFGTFGNVIILLSVVVVLLSYIKIFLLVKKFSADVRKKTLATCTPH

LFIFFTFTFVSVFPAVFNRLHPVVSSKAQVIRPFLMATNYILIPPLLHPVVYGFNNKDIR

AAVSRI

>CL14951.Contig3_All NAPLNLAVMSLERYVAICFPLRHSEIVNQKSAGVSIGLIWIFSSINLVIDIVYSLIINPV

SFKDSIHCFRERVIISRWQEDMFEGFNIFFFVTVTGILIFTYVNVVVVARSASTEKDSAK

KAHKTILLHFGQLVLCVNSFIYGTIERALAMTSSSRLFMDLRYMNFLFVLLLPRCLSPLI

YGLRDDAVRPLFVYYVRCGYQKVKPSGTIH

>CL15592.Contig1_All NQSFSSVLRIASFDIHPSAVYPIFLMGLLLHFFSVVSNLAILLLIATQRTLHKPMFYILF

SLPLNDLIGITAMLPRVLVDIVIIKNTVSYSACVLQAFLLHMYGGAIPFILAAMAFDRYV

AICKPLRYNTIMTPLAVAGIISLAWGADFALVLVLFLLQVRIRKCKNYIINVLCDNFSLL

SLNCGEDLTINNIYGLAITAFMHITTVSVQLFSYTHILVTCLKNKQSDAKSKAVNTCLAQ

IIAFFLFEIVALFTILSYRFPNISSNARNACGMMIFLILPVLNPIIYGMKTKDIRIAFFQ

LLKKGKV

>CL15592.Contig2_All

GGAIPFILAAMAFDRYVAICKPLRYNTIMTPLAVAGIISLAWGANFALVLVLFLLQVRIR

KCKNYIINVFCDNFSLLSLNCGEDLTINNIYGLAITAVLR

>CL15979.Contig1_All NGLPNVSSILTLQGFNLPPQSVVPAFLFASLNYMIILFCNLVLLLTILLNKSLHQPMYLL

LLSLPINDIIGSTGLFPQVINELLLDSRRIPYNACVTQAFFIHIYGAGSVFILTAMAYDR

YVAICCPLQYGTIMTHAHVMRIITLVWLSNFAVIGVLFFLLLRLPRCRSEMPHSYCDNPS

LLRLTCADTTISNIYGLALVAVTQVVALGIIFCTYIQILVACFRSKRADTKSKALQTCAT

HLIVFLLLECLGLFTIISYRIPNLSAQTRRFIGVSTMIFPPTVNPIIYGLKTKEIKDKIF

TFFRSK

>CL15979.Contig2_All NGLPNVSSILTLQGFNLPPQSVVPAFLFASLNYMIILFCNLVLLLTILLNKSLHQPMYLL

LLSLPINDIIGSTGLFPQVINELLLDSRRIPYNACVTQAFFIHIYGAGSVFILTAMAYDR

YVAICCPLQYGTIMTHAHVMRIITLVWLSNFAVIGVLFFLLLRLPRCRSEMPHSYCDNPS

LLRLTCADTTISNIYGLALVAVTQVVALGIIFCTYIQILVACFRSKRADTKSKALQTCAT

HLIVFLLLECLGLFTIISYRIPNLSAQTRRFIGVSTMIFPPTVNPIIYGLKTKEIKDKIF

TFFRSK

>CL2746.Contig1_All SGNSTEQKILKGNDPVRLNLTTALVQVLVWPFVYINLFMYFTYRKKPALQAEPRYVLFAQ

TLLADSALFIMTDFVVIGIHVHLLLPLPVCIPVAVSNQALALVSPTLITAMCLERYVAIC

FPLRHVDVFTPTRTMFISAAVWFLSYLRPFVDLFILFTTMPKGYMNRLNFCYYEILLVAR

WHMEMRGNLFALNYLVLLSILFFCYVAIIRVARRASGADRQAASKGQRTLLLHLLQLFMC

TLEVFCPYIESQVILIDIDLFIVVRYFNFLAFTILSRAITPLIYGVRDEKFQAAMKEFLL

QKRSKVGFVH

>CL2771.Contig2_All MKNASSFTTFILINYEPMEQQRYLYIIIFLLPYMLTIILNTCLIYIICKDRTLHEPMYIF

ICNLSFNGIYGSTALLPHMLNKLATKSYKISLPNCLTQVFCLHTFATVELMILAIMGYDR

YAAICTPLHYHNEMSPRKVKILIAVAWLFPFCTFALFLSLTIRLSFCSNVIYKTHCTNYD

LVKISCSDTSVNNIVGIILTGMYMFPQFLVILISYVQILRICLHASKECQKKALQTCIPH

LLTVINYVFGVCFELIQVRMKATQIPYGMALFMSVYFLILSTLLNPLIYGSNALKIHIYR

YLQKRKLA

>CL2791.Contig1_All

IWQYGGYFTLILLTYVVIVWTVLRLPEKGSRKKMFTTCISHIIVVSAYYAPKLVSYLLTR

IGVKLNLTERNAVLIVASTLPHLINPVTYCLTTKELKIRLINLLTKNRV

>CL2791.Contig2_All

NNTVVRVSEFIITGFDHLSHQKLLGSIIFITYVLILFFWSTNLSIIAANRSLHSPMYILI

CNLAIVDIVFSTSSNVSMIVVLLAEIKTISFNSCITGMFTYHLGDITTCLTIALMAVDRM

LAIRFPLRYHSILTNTRCFVLITMVWIVGMLTLGPLMAAAHSVPYCQPIIRYVFCDYAAM

IRAGCVNPEPYFSDPYTIGGLIWQYGGYFTLILLTYVVIVWTVLRLPEKGSRKKMFTTCI

SHIIVVSAYYAPKLVSYLLTRIGVKLNLTERNAVLIVASTLPHLINPVTYCLTTKELKIR

LINLLTKNRV

>CL321.Contig1_All MENETVPSYFYFTLFKGYENVRIMFFLLTIVTYFFIIVFNVTILLVVFKDKSLHEPMYLL

VSCLLFNSLYGSSALFPRLSADLLSTTHTISRPACFTQIFIIYIYAISEFTVLSVMAYDR

YVAICEPLQYHNIMTQRKTFLLVLCAFSYSVFGVAVGIYLSIGLLLCGNQIHRLYCSNWS

VVRLSCVSTLINNIWGFFLTVTTVFLPAGFILFTYMRILLVCRKSTAEFRGKALQTCLPH

IINFVTYSIAIFSDIALSRYEPGQILNAIVLIISLEFIVIPPILNPLIYGLNLPGIRRKI

GSM

>CL321.Contig2_All FSDIALSRFEPGQIINAIALIISLEFIVIPPILNPLIYGLNLPDIRRKILSMM

>CL3955.Contig1_All MAMNNQSIEGTLLVHQQFFKVEMTEGPVSKLIVAILVALVFIFINSIMFHTLLSKPVFRE

LPRYILFAHMLCNDSVQLMLSMSLYLMIVSLHQIPKALCALLVLLASTTFRNAALNLAVM

SLERYVAICFP

>CL3955.Contig2_All MAMNNESIEGTLLVHQQFFKVEMTEGPASKLIAAILMALVFIFINSIMFHTLLSKPVFRE

LPRYILFAHMLCNDSVQLLVCMTLYLMTVNLQQIPKALCALFLFFATTTFNNAPLNLAVM

SLERYVAICFPLRHSEIVNQKSAGVSIGLIWIFSSINLGIDVIYSLMVSPGSFKDSMYCS

RERIIISPWQIDKFQGFNYFFFVTVTAILIFSYINVVVAARSASSQKDSAKKAHKTILLH

LGQLVLCVNSFIFGTIERALAMTSSSRLFIDLHYMNFLFVLLLPRCLSPLIYGLRDDAVR

PLFLYYVRCGYQKVKPSVTIH

>CL4703.Contig1_All KAFFCTAPCAFFLYVNLVMLYTLRSKEIFTETPRYILFSHLLVSDSLQLWCTLVYYILYN

QTFDQETLKIIGVYCFLNYLVCRITNNYLTPLYLGLMSLERYIAICFPLRHAEIANKSRT

MLAILSVWLLGLILWTADLAAAMVLRGKEGGCTDYIIAQMVVTYQVNTALTGLVFSLVSV

VIVCIYIAIMITAKSSSTADKSKASKAHKTVLLHMVQLCLCLLSLLFGVVRRELGLSGLD

PVLVNEVTIFLFLMLNILPRCLSPLVYGLRDKTFKAYFKIHFLFCIKSKIQPSDLEI

>CL4703.Contig2_All KAFFCTAPCAFFLYVNLVMLYTLRSKEIFTETPRYILFSHLLVSDSLQLWCTLVYYILYN

QTFDQETLKIIGVYCFLNYLVCRITNNYLTPLYLGLMSLERYIAICFPLRHAEIANKSRT

MLAILSVWLLGLILWTADLAAAMVLRGKEGGCTDYIIAQMVVTYQVNTALTGLVFSLVSV

VIVCIYIAIMITAKSSSTADKSKASKAHKTVLLHMVQLCLCLLSLLFGVVRRELGLSGLD

PVLVNEVTIFLFLMLNILPRCLSPLVYGLRDKTFKAYFKIHFLFCIKSKIQPSDLEI

>CL4703.Contig3_All KAFFCTAPCAFFLYVNLVMLYTLRSKEIFTETPRYILFSHLLVSDSLQLWCTLVYYILYN

QTFDQETLKIIGVYCFLNYLVCRITNNYLTPLYLGLMSLERYIAICFPLRHAEIANKSRT

MLAILSVWLLGLILWTADLAAAMVLRGKEGGCTDYIIAQMVVTYQVNTALTGLVFSLVSV

VIVCIYIAIMITAKSSSTADKSKASKAHKTVLLHMVQLCLCLLSLLFGVVRRELGLSGLD

PVLVNEVTIFLFLMLNILPRCLSPLVYGLRDKTFKAYFKIHFLFCIKSKIQPSDLEI

>CL5232.Contig1_All MMGEAEGANISHAVFIFIGFPETYDHRDWYAVPVLLSYLLLLAGNSLLLHVIHSTASLHS

PMYVLVSALAIVNIVVPTAIIPKMLLAVLFDLREITLAGCLVQMFVTHFFSSVESTILLV

MALDRYVAICHPLRYVEIVNSALFVKLLVFTLVRSGSIMLTLVGLVAPLRFCGSNVISHC

YCDHMALVSLACNSTDKNSAMGVAVIVCFVGIDISLIFFSYVNILYVVLRAAAGEDRWKA

FHTCGTHLMVMMSFYLVGSVTFLSHNLNLPLPVDVNTCLGLLYIIFPASVNPVIYGVRTK

EIRHAILKIFKVQANKVFVVQ

>CL5232.Contig2_All ETYDHRDWYAVPVLLSYLLLLAGNSLLLHVIHSTASLHSPMYVLVSALAIVNIVVPTAII

PKMLLAVLFDLREITLAGCLVQMFVTHFFSSVESTILLVMALDRYVAICHPLRYVEIVNS

ALFVKLLVFTLVRSGSIMLTLVGLVAPLRFCGSNVISHCYCDHMALVSLACNSTDKNSAM

GVAVIVCFVGIDISLIFFSYVNILYVVLRAAAGEDRWKAFHTCGTHLMVMMSFYLVGSVT

FLSHNLNLPLPVDVNTCLGLLYIIFPASVNPVIYGVRTKEIRHAILKIFKVQANKVFVVQ

>CL5445.Contig1_All TMNGSTIILTFTAYEEIGPAKNVFFAIIFLIYLASVFTNTGLMLLIYLDTALHKPMYIFL

FGLMLEGLIGSTTVWPTVMANLATNIHSTSYEACLVQSYFITVYGGCMYTMLTVMAYDRY

VCIFQPLQYHTIMTPHKVRVLLVAANLFPVLLVVGQICLTSGLPLCHHAIHKIFCDNLSV

SNLGCIKTTYATVTDLYGVCALFFFVVLPVFLILLSYFRLILLTSKISADARRKAFATCA

PHIIIFVNFSMSVLFAVSYNRIAYYVPIGVNIFLSSLYVLIPPLFHPILYGMKNQEIKRS

LSKLLRSAYLVLG

>CL5445.Contig2_All

TMNGSTIILTFTAYEEIGPAKNVFFAIIFLIYLASVFTNTGLMLLIYLDTALHKPMYIFL

FGLMLEGLIGSTTVWPTVMANLATNIHSTSYEACLVQSYFITVYGGCMYTMLTVMAYDRY

VCIFQPLQYHTIMTPHKVRVLLVAANLFPVLLVVGQICLTSGLPLCHHAIHKIFCDNLSV

SNLGCIKTTYATVTDLYGVCALFFFVVLPVFLILLSYFRLILLTSKISADARRKAFATCA

PHIIIFVNFSMSVLFAVSYNRIAYYVPIGVNIFLSSLYVLIPPLFHPILYGMKNQEIKRS

LSKLLRSAYLVLG

>CL5918.Contig2_All APWQTLFYQALNGLYYVTVTLVILYSYIKVVLVARSVSSDQKSAGKAHRTLLLHFIQLLL

CLNTLLYGHIIGFMAVMLSYEVYYDVRYTIYLLVILLPRCLSPVIYGLRDEALRCVFMYY

FKCALSKVKPSVNMH

>CL6606.Contig1_All LLPFFQGISLCAILFTYLQILITCIFKKQSDARSKAIQTCGAHLIVFLFLECNAFFTLLS

HRFESAPTFLRRALGVSVMVFPPLLNPLIYSFNTKDIRRHILIFLKRK

>CL6606.Contig2_All MGATAVFPQMISTMLYHPRVIPYWECALQALFVHMYGGGNLMILTAMAFDRYIAICQPLS

YNTIMTNSNLIKIICAVWLTDFVLISISILLLLRLKNCSSEINDMFCTNPSLMKLACGDT

NVNNFYGLFTIAFFQGVSLCAVLFTYLQILITCVFKKQSEARSKALQTCGAHLTVFLFFE

FNVFVFFLAHRIQTAPLFVRRALGVSVTIFPPLLNPLIYGINTKDIRRNIVVILKRKIV

>CL6606.Contig3_All LLPFFQGISLCAILFTYLQILITCIFKKQSDARSKAIQTCGAHIIVFLFLELNAFIALLS

HRIASVPTFVRRVLGVSVMIFPPLLNPLIYGFNTKDIRRNILAFFKKK

>CL6688.Contig1_All NFTLLFTGYGPQGPTQYGVLFITLLLYIGTIVSNVAILLVIYFDSSLHKPMYIFLFNLAI

NGVLGSTAVCPKIMANLLKDNNYISVEGCLTQVLSINIYASCAYAIFAGMAYDRYVCICK

PLQYHSIMTPSRVKVLLALIYILPVSLLSVQVYLTSRLPLCRHTINKLFCDNLAIVNLSC

VKDVIGNLYGVCLVFVLVVLPLFLVILSYVKILHVSLKASENAQQKALQTCAPHLITFLN

FSVAILFSVIYNRISYYLPPEVNILISLDFILLPPLLHPLIYGFKTQDIRKSLYKIFRRR

V

>CL6688.Contig3_All NFTLLFTGYGPQGPTQYGVLFITLLLYIGTIVSNVAILLVIYFDSSLHKPMYIFLFNLAI

NGVLGSTAVCPKIMANLLKDNNYISVEGCLTQVLSINIYASCAYAIFAGMAYDRYVCICK

PLQYHSIMTPSRVKVLLALIYILPVSLLSVQVYLTSRLPLCRHTINKLFCDNLAIVNLSC

VKDVIGNLYGVCLVFVLVVLPLFLVILSYVKILHVSLKASENAQQKALQTCAPHLITFLN

FSVAILFSVIYNRISYYLPPEVNILISLDFILLPPLLHPLIYGFKTQDIRKSLYKIFRRR

V

>CL6688.Contig4_All NFTLLFTGYGPQGPTQYGVLFITLLLYIGTIVSNVAILLVIYFDSSLHKPMYIFLFNLAI

NGVLGSTAVCPKIMANLLKDNNYISVEGCLTQVLSINIYASCAYAIFAGMAYDRYVCICK

PLQYHSIMTPSRVKVLLALIYILPVSLLSVQVYLTSRLPLCRHTINKLFCDNLAIVNLSC

VKDVIGNLYGVCLVFVLVVLPLFLVILSYVKILHVSLKASENAQQKALQTCAPHLITFLN

FSVAILFSVIYNRISYYLPPEVNILISLDFILLPPLLHPLIYGFKTQDIRKSLYKIFRRR

V

>CL6914.Contig1_All FFLQARVRRCRIFILNVFCSNVTLLNLSCGEDTTINNIYGLFITAFMQIVTIAVQLFSYI

QILLTCVFNKQSNAKTKAVNTCLAQIMVFLIFEFIGMFGILSSRFPNVPTNARMVIGIMI

YVVLPVINPIIYGMKTNDIRIAFLHVVKIKK

>CL6914.Contig2_All MESNVLNQTFSFELKIASFDIPPSAVYPIFIIGMLIYLFSVLSNLTILLLIATQKSLHKP

MFYILFSLPLNDLVGITAMLPRVLVDIVIIKNTVSYPACVLQAFLLHMYGG

>CL6921.Contig1_All

MNTSILSMTLIYVTYKEMGSAKNVFLILVLTIYLASVIASGTVMLLIYMDTSLHKPVYIF

LFSLIVNGIIGSTAVWPKVMNILLTDDNTVSYEGCLVQVFLTGSYGACNYTMLTVMAYDR

FVFIFKPLHYHTVMNPYRVKQLVLIGNLIPLTVMLTQIYLATRLSLCKYTIQRAFCDNVS

VIDLSCDNDNFSLVCSVFGVCAIFCLGVLPMFLVILSYLKIILTILKMSTDARKKTFATC

SPHLIVFILFSFVSLFSIIYNRVYPDVSVRAKVIRPFFLATNYILIPPFVQPVIYGLKSQ

EIRQSFLKFRKRAILGFQL

>CL6921.Contig3_All MNTSILSMTLIYVTYKEMGSAKNVFLILVLTIYLASVIASGTVMLLIYMDTSLHKPVYIF

LFSLIVNGIIGSTAVWPKVMNILLTDDNTVSYEGCLVQVFLTASYGTCNYTMLTVMAYDR

FVFIFKPLHYHTVMNPYRVKQLVLIGNLIPLTVMLTQIYLATRLSLCKYTIQRAFCDNVS

VIDLSCDNDNFSLVCSVFGVCAIFCLGVLPMFLVILSYLKIILTILKMSTDARKKTFATC

SPHLIVFILFSFVSLFSIIYNRVYPDVSVRAKVLRPFFLATNYILIPPFIQPVIYGLKSQ

EISKSFLKYRKRAVLGF

>CL6972.Contig3_All VMNLTIKDAFEAALVKNLVIVAMGIVINCINGIIILTFFRNSVFHCETRYILYMNLVVND

MTMIFVSVTLHV

>CL717.Contig2_All TSRPLTVTDVQAFWMAMTDERIFKMFLIIFTHILFIYINVVMMVTLRSKATFCNTARYIL

FGHMLLIDTLHLSVSLVLYMLSSFFITLARAACSFLVLLSATTFTIAPLNLAVMSLERYV

AVCFPLHHCQLATPARTRLAIPFIWAAGCVNVLSDVCVLFLAKPPFYLAHTVCTHEQLMV

APWQGLKALILNILLFVSVALLLLYTYIAILIEARSASSDKASAHKAMKTVLLHAIQLGL

SLMSFWFVFLESLFSRLPFDIYKELRFVNYFLVVILPRCLSSLVYGLRDEAFKPLFKRHF

LFCGSWVK

>CL717.Contig3_All TSRPLTVTDVQAFWMAMTDERIFKMFLIIFTHILFIYINVVMMVTLRSKATFCNTARYIL

FGHMLLIDTLHLSVSLVLYMLSSFFITLARAACSFLVLLSATTFTIAPLNLAVMSLERYV

AVCFPLHHCQLATPARTRLAIPFIWAAGCVNVLSDVCVLFLAKPPFYLAHTVCTHEQLMV

APWQGLKALILNILLFVSVALLLLYTYIAILIEARSASSDKASAHKAMKTVLLHAIQLGL

SLMSFWFVFLESLFSRLPFDIYKELRFVNYFLVVILPRCLSSLVYGLRDEAFKPLFKRHF

LFCGSRVK

>CL7175.Contig2_All

ALSNGTLIHPPGFYIVGLSSMAHANIYLIFLGVVYVLTIVFNCFLLSLI

>CL7212.Contig3_All MQNSTQISFVILTAYTDVGHVKYFYFTILLALYVCIIFANVLLITIIYMDRALHEPMYLF

LCSLCVNELYGSIGLFPCLLTNLISNNHEIHLTYCYLQIYCLYTYGTVEICNLTIMSYDR

YLSICYPLQYNNIMTLNRVCILIVFAWLFSFGQFTVSLVLSVHLRLCGNIMEKVWCDNFF

LIRQSCTDTTVVNIYGLCMTVVVIAVPLVLILHSYIRIFKITFRSSNGPNKKSLNTCVPH

IVSLLNFSIGVSFEIFQGRVLKLNMPPTLRIIISVYFLMIPPLVNPIMFGLKLTKIRDAS

KKF

>CL7212.Contig4_All KKCLNTCVPHIVSLLHFSMGCSFDIFQSRAPKLNMPPTLRIIVSVYFLLIPPLVNPIMFG

LK

>CL8025.Contig1_All

EVSLDAFFIPDGARYPICLFGLVIYLFCISCNMTLLGLIIAKRSLHKPMYFILFSLPFND

LVGITVMLPKVLSDIFTDTSSTYYPLCVLQGFLLHMYGGGILFILAAMAFDRYVAICFPL

RYNSVMTPGVVVAIIAIIWGLDLAFILSLFSLQAGLPLCKTKLMNVFCDNPSLLKMTCGN

TFINNIVGLVYLALMQFISISIQVFSYVKILIACLVTRQNEARSKAINTCVAQLVIFFIF

EIIATFTILSHRFQNVHPDLQKIIGMLIFLVPPILNPIVYGLNTNEIRRNL

>CL8025.Contig2_All

DFFFIFTIFILQARLPRCKSKLMNVFCDNPSLLKMTCGNTFINNIVGLVYLALMQFISIS

IQVFSYVKILIACLVTRQNEARSKAINTCVAQLVIFFIFEIIATFTILSHRFQNVHPDLQ

KIIGMLIFLVPPILNPIVYGLNTNEIRRNL

>CL8025.Contig3_All LNASIHLEVSLDAFFIPDGARYPICLFGLVIYLFCISCNMTLLGLIIAKRSLHKPMYFIL

FSLPFNDLVGITVMLPKVLSDIFTDTSSTYYPLCVLQGFLLHMYGGGILFILAAMAFDRY

VAICSPLRYNTLMTPKAVAAIIALVWGLDFFFIFTIFILQARLPRCKSKLMNVFCDNPSL

LKMTCGNTLINNIVGLTNTALMQIISISIQVVSYVKILIACLVTRQNETRSKAINTCVAQ

LVIFFSFEITSTFTILSHRFQHVSPDLQKIMGMLIFLVPPILNPIVYGLNSHEIRRNL

>CL9458.Contig1_All IKEFILVGFPGLHSDYHGLVAFILCLVYVTTVVGNSVLVVTFVHVPSLHKPMYIIMLSLA

LSDIGFSTVALPKIISRYWFDDKFIAFDVCFSQMFLIHYFGTVNSYIMGIMAMDRYIAIC

FPFRYPVVMKNRTMCILNIFFWLFSFVTPAIFSVFDYRLPYCGPNQIFQCYCDHYSLVTQ

ACASHGFATLIAFTVAMLVLLIPLAFIIYSYIHIIVSVARIASSQNRWKTFSTCTTQLCI

IILYYLPRCIVYIFNVTGLYMDIDLRITLILFYSLFPPLVNPFIYCFRTKEIKQALGHWF

S

>CL9458.Contig2_All

TPFNVCMTQVFLVHFFGTVNSYIMGIMAMDRYIAICFPFRYPVVMSYRTMSNLNIVAWAF

SLTTPGIMIVLDAMLPYCGPNRINQCYCDHISVISQSCGDTSIAKLVAFTQAMLVLLIPL

AFIIYSYIHIIVSVARIASSQNRWKTFSTCTTQLCIIILYYLPRCIVYIFNVTGLYMDID

LRITLILFYSLFPPLVNPFIYCFRTKEIKQALGHWFS

>CL9638.Contig1_All QCYCDHISVISQSCGDTSIAKLVAFTQAMLVLLIPLAFIIYSYIHIIVSVVRIASAQGRW

KTFSTCITQMCIISLYYLPRCTVYIFDLTGVYLNVDLRITLVLFYSLFPPLVNPFIYCLR

TQEIKQTLARWFSVKSLIR

>CL9638.Contig2_All 17 313 minus strand odorant receptor [Danio rerio]

LAAYIMSMVVLLVPLVFIVYSYVHIIVSVVRIASAQGRKKTFSTCSTQMCIISLYYLPRC

TVYVFDGSGFYMNLDIRITLVLFYSLFPPLANPFIYCLR

>Unigene11975_All ALALRNTLTIVVAVCLFIPHTFCHTNTIYHCLCEHTSVVKLVCGNIARNLVAGAMTFSLT

TSDCILVLATYIIIFIVIFQSPSGESRQKAIHTCSTHLTVICVGYVSVVCAFVGYRVSGI

PPDTRVLLTLSYLIIPCAFNPVIYGIRTKEIKVHVLKLLKLSKVDS

>Unigene14513_All KAFFCTAPCAFFLYVNLVMLYTLRSKEIFTETPRYILFSHLLVSDSLQLWCTLVYYILYN

QTFDQETLKIIGVYCFLNYLVCRITNNYLTPLYLGLMSLERYIAICFPLRHAEIANKSRT

MLAILSVWLLGLILWTADLAAAMVLRGKEGGCTDYIIAQMVVTYQVNTALTGLVFSLVSV

VIVCIYIAIMITAKSSSTADKSKASKAHKTVLLHMVQLCLCLLSLLFGVVRRELGLSGLD

PVLVNEVTIFLFLMLNILPRCLSPLVYGLRDKTFKAYFKIHFLFCIKSKIQPSDLEI

>Unigene15712_All MNSTTSQFLVRDTFSAAFVKNFIVVLLLLVLNYINGSLVATFLRNQVFYEDPRYILFIHM

VINDAIQLTVTITLFVLSYIFYAINVSFCCFFILVAVFTTRNTPVNLAGMAIERYIAICD

PLRHAQICTIRRTYILIGLIWFVTVVPDITDLFVTLAREPVSFFHTAVFCIRSNIFKDPV

LLYKRQAFDGLYFSLVFFTLVFTYLSILCAARAMSTDIRSAQRARNTILLHGVQLLMCML

SYISPSMDLIMIRIFPSQILEIRYANYLIVYIFPRFLSPIIYGVRDKKFRRYLKRYFVCK

QCSSEVQHE

>Unigene16199_All

SSIRDVLIFTVYKELGSNKSVLFTTVLITYVLSLVINVSLLLLFYLDHSLHKPMYIFLFC

LLLNGLIGSTAVWPRVMFLLWTDIHSTSYELCLVQVFLMGTYGGCNFTILTVMAYDRFVS

IFRPLQYHTVMTPLRVKLLLVVANLPAALVLGQVILTIQIPLCKYNLHRLFCDNLSVSNL

SCGETFQARLSNLYGLCAIIIFVVFPICLIFLSYVKIIKLSLKASRHARRKAIETCSPHI

MVFMNFSLASLFSVIYNRRNPYLPGEANILLAIIYILLPPLLHPIIYGIKSQEIRCSIFK

FWKRK

>Unigene18610_All TTITEFILVGFPGLHPNYHKLMGFVLCLLYVSIVAANTTYVVIFSLKPSLHKPMYIILVS

LALSDIGFSTAALPKIIARYWFDDKVIAFHACFMQRLFIHSFGSLNSFIMMIMAVDRYLA

ICFPLRYPVLVKNHTMVIVNCWAWVFSLVPTGVSFIYLYMLPYCGPNIIYQCYCDQNSIL

KLACAGQSWPTLIGFCLAMLVLLIPLAFIVYSYLHIIVSVAKLKSSKGRWKTFSTCSTQM

CIITIYYLPRCTVYALNLVGISMNINLHIGVALIYSLLPPLVNPLIYCFRTKEIQETLVR

LIGFKWVGKRSVAAVS

>Unigene21223_All NVTNVSYVLMLQGFDVPQERAFLAFLLAVLGYMVIVFCNLLLLLTVILDRGLHQPMYILL

INMPINDLIGSTALFSHLFRDFLSNSKAIEFPACVIQAFFIHIYAVGVVFNLAAMAYDRY

IAICRPLQYASIMTNARLMTIIALVWGVNVLIIGVLFILLLRLPRCRNNVTHTYCDNPSL

LRLMCGDTTINNIYGLLTVTCTQPITVGIILYTYLQILITCFRSKSTDTRAKAMQTCATH

LVVFLLFECLGLFTIISYRIKNLSPVLTNFIGVSALIFPPTLNPIIYGINTKEIRKRAIM

LFKKYV

>Unigene22779_All NFTFNFDLTLDPFSIPAGARYPIFFIGLLIYLFCISCNMTLIGLIITRRNLHKPMYFILF

SLPLNDLIGITAMLPKVLSDIVTESNQVYYPLCVLQGFLLHMYGGGVLFILAAMAFDRYV

AICFPLRYSSIMTPNVVVTIIAIVWGLDFVLILVLFSLQARLPLCRSQIMNVFCDNPSLL

KLTCGNTFINNIIGLFNTALMQIISI

>Unigene22851_All LYNICGSAIIVMFPIFLILVSYMKIITLMLKCSKCARQKILDTCLPHVMVFVIFTLSSVF

PVIYTRLSSGLLMQAHIFMAIIYILVPPLLHPIIYGIKNQEIRHSFSKMWKRKV

>Unigene29409_All LMVSPGSFKDSMYCSRERIIISPWQIDKFQGFNYFFFVTVTAILIFSYINVVVAARSASS

QKDSAKKAHKTILLHFGQLVLCVNSFIYGTIERALAMTSSSRLFMDLRYMNFLFVLLLPR

CLSPLIYGLRDDAVRPLFLYYVRCGYQKVKPSGTMH

>Unigene30140_All LFPQFLVILFSYVQILRICLNASKECQKKALQTCTPHLLAVIIYFFGAIFEVTQVRIKNN

QIPYGIAVFMSVYFQCTSTIYHRI

>Unigene30141_All LFPQFLVILFSYVQILRICLNASKECQKKALQTCTPHLLAVIIYFFGAIFEVTQVRIKNN

QIPYGIAVFMSVYFLIMPPLLNPVIY

>Unigene46725_All YENVRILYFLLTITAYSCIILFNVTIILAVFKDKSLHEPMYILMSCLFFNSLYGSSALFP

RLSADLLSTTHTI

>Unigene48164_All

IITFVNFSLVSLFSLFYNRYNSFLPGGVNILMSINYILVPPLLHPIVYGIKTKEIRQRFS

KLIR

>Unigene49175_All

NDTLFFAVYAGVGSMGYVLLAVVLLAYLATVLASATVMMLIFLDTSLHKPMYIFLFSLIL

NGLIGST

>Unigene54358_All

QRKAISTCAPHLIIFINFSLVALFTIIYNQFTSDVSVDMHIFFAMQFSLIPPLLHPIIYG

VKTKDIRKCIAR

>Unigene54574_All

MNQSAFDVIVVFTAYHSIGTNKYVYIAVILSVYMASVFVNVSLMLLIIRQSTLHQPMFIF

MFNLTLVGLIGSTVV

>Unigene64615_All

FAHMLCNDTVQMAVTSMLFIMVMYLLQLTKALCAVIVYVSAVTYRNAAFNVAVMSLERYV

AICFPLRHSQMVTHSATAVAISVMWMLSSICPVIDIFYGIIMDPMFFSGQIFCFREMLVR

TTWQIKMFQTVNGMSFVSVTLIIIFTYIGVIIAARGVSTDKSTAQKAKRTILLHFIQLIF

CLTSLLYGSIEGLLTNLSNVELFRSLRFVNFLLMLILPRCLSPLIYGLRDDAVRPLFLYY

VRCGSKKVKPSVNAH

>Unigene64616_All

TNKSSSEIQYLEQVAYTLRIRDDTLIKLSVVVLASLLFIYINSMMFYTLLSRPVFRELPR

YVLFAHML

>Unigene67012_All LLRPLNEKILLVQVLVGIFLYVNSLMIFTFLKKEAFRTDTRYILFAQTLFMDSSLMVLTD

LAVIQSYFQYTI

>Unigene7032_All NGTAQGVLIAHQQMFQVQLTAQGPLTKLLVAILMSALFILINTIMFVTLLSKPVFRDTPR

YILFAHMLCNDSIQLLFSSMMYIISLCYVQVAKAVCSILILVTASTSRNAPINLAVMSLE

RYVAICFPLRHADIATKSGTGVAISTIWFFGAVNPIIDVLYTSATDPSFFSERMFCTRER

IFIAPWQRELFEGLNAFYFVAVTLIIVFTYISVMVAARSATSNKESAKKAHRTLLLHLAQ

LGLCLNTLVFGSIERLLAMTSSSRLFMDLRYVNFLFVLILPRCLSPLIYGLRDDAVRPLF

LFYLLCGTRKVKPKVNVH

>Unigene74121_All FIIVFLVYLSSIIANVFLMCLIYLDSALHKPMYIFLFSLLVNGLIGSTAVWPKVMLILIT

GDNTISVAGCFTQTYFMIIYGACNFTILAVMAYDRLLSIFNPLEYHALMTPQKIRQLLFA

SSVMPAVVILGHICLISQMSLCQYRIPRLFCDTLSFFALSCVENIQSRVSNFYGLCVFIC

FGALPIALVFLSYFKIIVFSLKASGNAKRKAFQTCTPHLIIFTNFSFTSCFFVIYVRSNP

NLQNLYLPIFYNLSPPLLHPLVYGIRNKQTRQSWSKIKKRIIFTL

>Unigene76039_All MENVSISFELSLDPFFIPAGARYPIFFVGLGIYLFSAACNMTLLGLIIAKRSLHKPMYFI

LFSLPFNDLVGIT

>Unigene81937_All

GILALAWGLDFAMIFVLFLLQSRVRRCRNFIMTVYCSNVSLLNLSCGEDTTINNIYGLAI

TAFMHIITITIQLFSYIQILLTCFFNRQSDAKTKAVNTCSAQIIVFVIFEMVSLFILIAY

RLPNVSTDARNACGMMIFLILPVVNPIIYGMK

>Unigene81949_All EGEISNETDSITCVLCHQEFWSNRQRDKCLKKETEFLSYKEIMGILLTVISVIGTCLTCV

IAIIFFKYKRTPLVKANNSELSFLLLFSLMLCFLCSLTF

>Unigene84414_All

HMLCNDSIQLLFSSIMTIFSFAYIRQTKAACSFLLFVTSSSSTNAPLNLAVMSLERYTAI

CFPLRHSEFSTTKRT

>Unigene85149_All YVAICMPLRHADISSPRNRRRGLAIIWVISTLLPTFTVVSFISLVPPTLLLTYVVCSIEM

MYVQAWLNHAHNAILLLYFSVMFIIIVFTYMSIFKAAQAASSDNKKSTHKGLRTIILHAF

QLLLCLAQFVCPFVEMAILKIDFMLFINFRYSHFIVFLIAPRCLSPLIYGLRDEKFYVVL

RHYAFFGLDTV

>Unigene87980_All

PIYLTLMSLERYVAICFPLRHVQIANKRRTMVAVMAVWSLGLVIWTTDLTVAMVFQAGSS

KRSCSDYILSQMVVSYQVSTGSIALVFTLACVVIIYVYIAIVVTARSTTTSDKSSASKAH

KTVLLHMVQLCLCLSSLLMGAVRRTLVMSGLDRVRYDEVSYVLFLMLNILPRCLSPIIYG

LRDKTFCAYFKVHFLF

>Unigene88695_All

TNNTREVFQEMIQIEMALDRRVFKVTVTIIISFFFVYINTVLFVILLSKPVFRDTPRYVL

FAHMLCN

>Unigene9043_All

RFQHVSPDLQKIMGMLIFLVPPILNPIVYGLNSHEIRRNL

>Unigene91230_All

NGICMAFVWVTLVYTYARVLCAARAASSNKGQIQKAQNTILLHAAQLLLCMLSYVVPVLE

>Unigene95547_All

SAKKAQSTILLHGAQLLLCMLSYITPYIEMALVPFFPVHRSTIMFLCYLITMILPRLLSP

LIYSIRDQKFAKCMSQYYSCRV

>Unigene99783_All IEVSFTFFYFVSSSVIITYTYIAIVRAAKSLTDQAKKSTSKSNETVLLHLVQLSLCLTSL

FFNFVTKEIRPKLGEAAFLNIQYFLFMIFIICPRCLSPLIYGLRDQAFGSLFKYYFLFGT

K

>Unigene14954_All

SSNASMVRPLRDTFQTAVTKNVVVVALCLSINYINGTLVHTFFRHRIFSENPRYILFIHM

VLNDMIQLTIAVLLHVISYAVFTINVSLCCFLLMIAVFTTLNTPLNLASMAIERYIAICN

PLRHAQICTARRTYSLIGLIWLMGAIPILPDLFILLATEPLAFFHSRIFCQRDSVFRHPY

LVEKKNISHMVYLSFVWLTLVYTYFRIMFAAKAAKSDARKARNTILLHGVQLLMCMFTYI

GPLLEGLLVYLFPMLLLEMRFTIYVIVHILPRLVSPVVYGLRDQTFCKYLKNYYVC

>CL1061.Contig1_All

GFYPKFLIDLLSEVAVISYLQCLTQTIVIHSSAICEMTTLTAMAFDRYVAICRPLQYHSI

LTSQMVLKLLSLVWFYPLSVTLTVVLLSVRVPICGSLISKLFCDIPSILTQGCYQTTTNR

IFGIFIMTGQVIQVLLICVSYAQIVRVCLRSREGR

>CL10717.Contig1_All MNVSDSVTFFVIEGLQEKKMLMFSIFLIVYIMVLCGNSMIIYLVRTNPKLKSPMYFFLYN

LSFSDMVYTTVTIPNMLSGLLKEEHTISRSGCLLQMYFFLSMAVTGRYILTVMAYDRYVA

VCNPLRYAAIMTKKVCILLVVAAWFFGFVTLLPALSLAVPLPFCGPNRVQHVFCDHSSVV

RLACGNTTVNTVVALSAAMIVLIGTLCLILTSYISIGKAVHGMGRAEKIKAFATCASHMI

VVCISYVSAACVYISYRVATFSPDARMIVAVLYSVLTPLLNPIIYSLRNKELWEALTRAL

CKHAAKPTSNRKTIPSVS

>CL11354.Contig2_All

FYLTGFSSLGEHRHLLSIPFFLLLLYVTTANAIVTFVIATQKKLYEPMYVLIGSLTLLSF

FYPIFFLPRMVISFASGRNEITKEECLIQMFLIHFGGSFQSSILLQMAVDRFFAICWPLR

YHNIVNLRNS

>CL11354.Contig3_All FYLTGFSSLGEHRHLLSIPFFLLLLYVTTANAIVTFVIATQKKLYEPMYVLIGSLTLLSF

FYPIFFLPRMVISFASGRNEITKEECLIQMFLIHFGGSFQSSILLQMAVDRFFAICWPLR

YHNIVNLRNSVLFTAALAFRNTLTIVVKVGLFIPLTFCHTNAIHHCLCEHSSVVKLACGN

IARNYIAITVAFSLTTGDCVFIAATYIIIFIVIFNSPSGESRQKAIHTCSTHLAVLCVAY

LSVLCAFVGYRVSTIPPDVRILLSLAYLLIPSCFNPVIYGIRTKEIKVHVLKLFK

>CL11354.Contig4_All FYLTGFSSLGEHRHLLSIPFFLLLLYVTTANAIVTFVIATQKKLYEPMYVLIGSLTLLSF

FYPIFFLPRMVISFASGRNEITKEECLIQMFLIHFGGSFQSSILLQMAVDRFFAICWPLR

YHNIVNLRNSVLFTAALAFRNTLTIVVKVGLFIPLTFCHTNAIHHCLCEHSSVVKLACGN

IARNYIAITVAFSLTTGDCVFIAATYIIIFIVIFNSPSGESRQKAIHTCSTHLAVLCVAY

LSVLCAFVGYRVSTIPPDVRILLSLAYLLIPSCFNPVIYGIRTKEIKVHVLKLFK

>CL11354.Contig5_All FYLTGFSSLGEHRHLLSIPFFLLLLYVTTANAIVTFVIATQKKLYEPMYVLIGSLTLLSF

FYPIFFLPRMVISFASGRNEITKEECLIQMFLIHFGGSFQSSILLQMAVDRFFAICWPLR

YHNIVNLRNSVLFTAALAFRNTLTIVVKVGLFIPLTFCHTNAIHHCLCEHSSVVKLACGN

IARNYIAITVAFSLTTGDCVFIAATYIIIFIVIFNSPSGESRQKAIHTCSTHLAVLCVAY

LSVLCAFVGYRVSTIPPDVRILLSLAYLLIPSCFNPVIYGIRTKEIKVHVLKLFK

>CL2771.Contig1_All

MKNASSFTTFILINYEPMEQQRYLYIIIFLLPYMLTIILNTCLIYIICKDRTLHEPMYIF

ICNLSFNGIYGGTVLLPHILSKLATKSYKMPLANCLIQIFCLHTFGIIELMILAVMAYDR

YAAICIPLHYHNKMSPRNVKILIAFSWLFPLCAFPMWMSWTTQLSFCGNIIYKTHCTNFD

LIKLSCNDTSLQNIVGMLLMGVFVLPQLTVILFSYVQILRICLHGHKDSKKKALQTCMPH

LLTVINYVCGLSFELIQVRLKTTHTQYGMSLFMSVYFLIIPPLLNPVVYGSTVLKKHI

>CL5918.Contig1_All HMLCNDSIMILFTFLIGIISYVHRPTKAMCSLIMIVTTSTSTNAPLNLGVMSLERYIAIC

FPLHHRELATTKRTYVAIAAIWFFGLVNPVTDWVYSSIFDPDFFFEQVECGHETMFRTAP

WQTLFYQALNGLYYVTVTLVILYSYIKVVLVARSVSSDQKSAGKAHRTLLLHFIQLLLCL

NTLLYGHIIGFMAVMLSYEVYYDVRYTIYLLVILLPRCLSPVIYGLRDEALRCVFMYYFK

CALSKVKPSVNMH

>CL6972.Contig1_All LIAVQLURHWVMNLTVRDDFQEALVKNLVIVALAIVINCINGIIVMTFCKNSVFHSNSRY

ILYMNLVVNDMIMIYISVIMYVLSYAHPFFKASMCCILVVVSSTTYMNTPIILAGMAIER

YIAICKPLHHAQICTVRRTYVLIGLTWGVGLIPALVDVVIVLATRSAGFFSTVVFCYYLS

LYNTRYHEEKARVVQAMYMLFVWLTLIYTYLRIFLTAKAATGDTASAKKAQNTILLHGVQ

LLLCMLSYITPLIDLALITFFPMHRSKITFLGFLITNIIPRLLSPLIYSIRDQKFAKHMS

QYYSC

>CL6972.Contig2_All VRQVQLURHWVMNLTVRDDFQEALVKNLVIVALAIVINCINGIIVMTFCKNSVFHSNSRY

ILYMNLVVNDMIMIYISVIMYVLSYAHPFFKASMCCILVVVSSTTYMNTPIILAGMAIER

YIAICKPLHHAQICTVRRTYVLIGLTWGVGLIPALVDVVIVLATRSAGFFSTVVFCYYLS

LYNTRYHEEKARVVQAMYMLFVWLTLIYTYLRIFLTAKAATGDTASAKKAQNTILLHGVQ

LLLCMLSYITPLIDLALITFFPMHRSKITFLGFLITNIIPRLLSPLIYSIRDQKFAKHMS

QYYSC

>CL7175.Contig1_All LDLRAALSNGTLIHPPGFYIVGLSSMAHANVYLIFLGVVYVLTIVFNGFLLSLIWLNHKL

HTPKFLAVANLAVVDTLISSCIIPSMLKFFLFRDSFVQFDLCIVQMGVYYCCTSLESFSL

AVLAYDRLIAICFPLRQHTINTNTNMLCILGSIWALLIACLLFACLIMTRLSFCDSVEVF

SFFCDYTPVYKLACNDFSLQWSTAVSLSLTIILGPLTFIIISYASILIAVFKIKIAGNRY

KALATCSEHLILVAIFFIPKLSLYVLGFLFYRLDMDIRLVTLSMSTCMPPCLNPVVYALK

TKEIRSKAQMLFCKTKVR

>Unigene109144_All

LCQVFIINVYATGTYSILTVMAYDRYVSIIYPLQYHAIMTPQKVKRLLAVSNFVPVSSAF

GQVWVTSQVPLCSSTIPKLYCENISVSKLSCASSKLYRVSTLYGVSVFVISVV

>Unigene1237_All FTLEAYRRIHDQRFIFAAVLTLLYPVIIFGNLLIIYVVSVERTLHKPMYILICNLACINL

YGGSSLAPFIVANILSGTFQISWVACFVQVFSINTYGGCEMMNLMMMAFDRYISICFPLN

YRQIMSPLIVIICVIFIWLIPFGRVTITLSITASLKICGNIIEKVYCDNYSVVKLACSES

SAVNIYSATVTFIYVLLPFFVIIYSYIRIILICVQLTRKGQTKVMNTCTPHLVSISTFFI

GCAFELYQSRFDMSHVPYAGRVVLSLYFLIFSPVLNPVIYGARTEKINEAIK

>Unigene22006_All

IVSFVFIYINSIMFFTLLSKPVFRDTPRYVLFAHML

>Unigene23721_All ILAGYIDTGNIAYLYFTILLLFYFCIIFANVLLITVICINRALHEPMYLFVCSLCVNELY

GSTGLFPCLLANVISDTHEIPLAYCYMQMYCLYTYATAEFCNLSVMSYDRYMSICYPLQY

SSIMTQRKVCILLMLAWSFSFSQFTITLSLNLQLELCGNVIQKVWCDNFLLVRQACSDTT

MNNIFGLYATALCVGLPSLLILYSYIRIIIITFRSSYEAKKKCLNTCAPHVISVINYTIG

CAFEMIQGRLGKANMPDMLRVFISLYFIIIPALFNPIMYGLKMSKIREAC

>Unigene25551_All

MFVLMGINETATNKHIYFAFVLTAYLFTVFVNTTLIITIVLEKALYEPMYFFVCNLCINA

IWGANSFYPRFLFDLSSQVNLISYYECLIQICFIFYYVFCEYTSLAVMAYDRFVAVCRPL

QYHSLMTPRRVMYLLLFTWGFTLTEVAIGAVLTVRLPMCGTHLHKIYCSNWPIVRLSCVD

FTGNNIYSYVLIIIHVSEALLIIVSYVHIIRTSLQSKSGWAKFMQTCLPHLITLINFNVA

LLLDVFDARYGLYQKSQAIRNFMAIEFLIVPPLINPLIYGISLKKIRTRIMKFYKHQ

>Unigene28245_All

RFIFVIMLTFLYPIMIISNAALIFIICSEKSLHKPLYILICNLACINLYGGCGLTPFVVF

RILSGNFQISKSACLCQVFSVMTFGGCEIMNLMVMAYDRYVSICLPLHYEKNYVCLNT

>Unigene34891_All

LIGSTAVWPYVMVILLTNLSIISYEGCFVQFFLLGSYGSCNFTILTAMAYDRLVSIFKPL

QYQTIMTPQRVKLLLFMASFIP

>Unigene62103_All NWPQLEVVLFVVILIFYLMTLTGNLFIIILSYVDSHLHTPMYFFLSNLSFLDLCYTTSSI

PQLLVNL

>Unigene64046_All

FESVFTMGSFDLNPEAVYVTLGLGIVIYLFALCSNLLLLGIIAVHIHLHQPMYILLFSLS

FNDIIGATAIIPPVLYNLMLEDRQTSFLACLFQAFALHWYGGASYLILSAMALDRYIAIC

HPLRYHAIMSKNAVTYLIMLCCIINVLLVGSVFGLTLR

>Unigene67013_All

MIFTFLKKEAFRTDTRYVLFAQTLFMDSFLMILTDLALIGNYFQFPIPWIPCCVFCFLMS

WLSLVTPLTLAAMCLERY

>Unigene78523_All

MENSSFHKVFILTGLQQSGRNKSLYFYLTSVLYILIIAMNVTIIITVRVDK

>Unigene82069_All

DDVEEALAKNLTIVALGVAINCINGMIVFTYLNNAVFHNDTRYILYIHLVINDMIMITLS

VSLYVLTYAYPIYNVSVCCMLIVIGSATHKNTPLILAGMAIERYIAICRPLHHAQICTVQ

RTYILISIIWVVGFIPPLADVIILLIVKPWSFFFTNIFCYPYTLYGSKYYDEKTKVVQAL

YLSFVWLILIFTYFRVLAAAKKAKGDSPASKAHNTILLHGVQLLLCMLSYISPLLDIILV

PFFPAHRSKISFFSYLITNIVPRLLSTLIYGIRDKNFAKHMSVYCSCKMIIVKVKPKVL

>Unigene86653_All MNTTIILDDFKDALSKNLTVVLCGLFILYVNGALMRVYFTVPSFTQEARYVLFIFLVFND

MMMVALTVVLHVLTYKPRFISVPVCYILV

>Unigene9188_All SSALTLESLKLSPPRNYPAFIFGTLVYLVIVFFNTVVLSVIVRSKDLHKPMYIILFNMPL

NDLMGATAFFP

>Unigene92818_All

CTVRRTYVLIGLTWAVGFTPTIVDVFIVYAIEPTRFFSSVGLCHPLTIYISQIYAQKTQV

VQGLYMS

>Unigene92982_All SRNWRRRRKKKKKKNTTVKKRKKUVPKSURIKEAERDRRANEGEKQKKKGEEKRVKKR

>Unigene96506_All LTSKIISLIAGIAVLRSLYMVVPLVFLLLRLPFCGHRIIPHTYCEHMGIARLACASIKVN

IRFGLGNISLLLLDVILI

>Unigene69531_All CLTRMSFLVLFACIEGMLLTVMAYDCFVAICRPLHYPVIVNPHLCVFFVLVSFFLSLLDS

QLHSWIVLQFTIIKNVEISNLVCDPSQLLKLACSDSVINSIFIYFDSTMFGFLPISGILL

SYYKIVPSILRMSSSDG

>CL10990.Contig2_All VKEVFRTNTRYILFAHTLICDCAFLIFTNMLLLLIVRRIIMPTGLCVVICVVLVDLNIAT

PLTLTAMSVERYVSICMPLRHAELSTPQRAVHTILVIHALCSVHVLAMLLSLFAVMPLSF

YSSSVMCSLVMLHTYRWQVYLNDTILQLYFVFMSLVICFSYVKIMGVAR

>CL11013.Contig2_All ASESGNSSEDSRLLEVTDVMELNLYTATVQLVVWPFLCLELFMLFILKKREVFQTEARYV

LFAQSLIADSCLLVLTDFVVITLHVRLLLPIAACIPIVIIMDVFSNVSPFIIVVMCVERY

VAICMPLRHVNIFSPDMTILYKAVIWLL

>Unigene228_All

VRVTVTTTVIQLLVWPFIGINLFMFHVYRQREALWSEPRYVLFTQTLLADAFFLALTNFV

VLTIHSRQLLPVAFCVPVCMLMEGLTHLSPTVIVAMCLERYVAICMPLQHTNIFSPHRTR

VLIAIVWFVSFLKPLIDFSIFLSHVTESYFIQPTFCSYEIMLLRTWHMVMRGNLYILNYL

VVLVILLFCYMSIIVVARRASGDNKQAASKGQRTLFLHLLQLLLCTLETICPYIEAQILQ

TGNLFVYMVVRSFNFMAFSIVSRAVSPLIYGFRDEKFYAAIKNYASRAVNKVAS

>Unigene66561_All

LKVSKTHGATESHRCLRIPPPGTLRGSRTAA

>Unigene97245_All

DMLLSVMAYDRFVAICHPLYHSAVMNPCFC

>Unigene14297_All 3 2483 vomeronasal 2 receptor, x1 precursor [Danio rerio] >gi|94733469|emb|CAK05327.1| novel protein similar to vertebrate phermone receptor protein [Danio rerio] >gi|126632607|emb|CAM56435.1| novel protein similar to vertebrate phermone receptor protein [Danio rerio]

GEVLIGGLFPLHYMASKPDQNYTSRPQHSQCSGFDLRAFRWVQTMVFAIEEINRNTTLLP

GLTLGYRIMDSCDHVHTGLRGGLALVSGSLPPRQSPTEGSQCTHNAPVSALIGLASSTPT

RAVAHTLGPFGIPVVSYFATCTCLTNKQVYPSFLRTVPSDMFQVQGLVQLVAHFSWHWVG

TVGTEDDYSRYGIQAFSEQLQEWGGCLAFHQTIPKEPTVAKIRAIADTLESTTARVVIAF

ATEGQLLDLLTEVARRNLTRLQWVASEAWVTASLLTAPEFQPVLAGTLGFSFRGTVIPGL

SDFLLKVRPSPRPQSAFTNMFWEELFGCKLVFGSHSSNTSSKTQPQCSGNEDLTSRKTSY

SDVSQFRISYNVYKAVYAIAHALHELLQCATLWENTTRVGCKTTTAFTPRQLLMHLKRVN

FINQFGEKVSFDSNGEPVPLYDIINWQRDHKGGILFKKVGSYDGSAPNGQQLWMDEDAIV

WTEGHQAPVSVCSVACPPGSRKASRPGEAVCCFDCLPCAEGQISNTTGATECLRCPLYYW

SDRDRVACIAGVEEFLSFQETLGIILVVLSLLGVSAAAVITVIFLCHKTTPIVRANNSEI

SFLLLLSLKLCFLCSLVFMGRPSPWSCRARQAAFGISFVLSISCILVKTIVVLLAFRSTL

PGSRKGQLFGPPQQRAFIFCCAAVQVVLCASWLSVSPPVPTKNTAYQGGRILLECKGTWP

QGFYLVLGYIGLLSCICFVLAFMGRKLPDTFNEAKLITFSMLIFFAVWISFIPAHNSSPG

KYTVAVEIFAILASSFGLLFCIFAPKCYIILFKPEQNTKKGMTRKYS

>Unigene79724_All 188 1936 olfactory receptor C family, w1 [Danio rerio] >gi|63102050|gb|AAH95823.1| Vomeronasal 2 receptor, d1 [Danio rerio]

MFPIHSKGVEQKLTFTERPGNRRCRGFNLRVFRWSQAMVYFIEEINRNPALLPNITLGYR

LYDTCGVEMFSLRTALSVISQPLKRNATGVCVSPSVPLIVGDSGSSLSMAISRVLNLFKV

PLVSYFASCACLSDKSQFPYFFRTIPSDVNQARALAYLVRHFGWTWVGTMGADDDYGRTG

IDMFTAEVTRLGVCVAYRITIPKLPSQEQLREIVATIRDSTAEVVVAFAIEEDIEPVVKE

MVSQNVTGKQWVASEAWVTSTLISTRENYASLSGTIGFAIRRAEMPTLKHFLQDLRPLES

PYNPFIREFWETQFQCSFNTTLPAASMADPMHYTNTCTGRERVEDTDSIYNDVSQLRVTY

NMHKAVYAVAHALHNLLTDQNSTVSIHNLHPWQVVRYLRTVNYTNIFGDVVHFDENGNPV

GAYDIVNWQRGSPDGPVEYVTVGRFDSSLPPTQQLLVNSDTIIWHRGQKEVPKSVCSASC

QHGYRKATREGQPVCCYDCVPCAEGTITNDTDQPECIPCPDDYWSNSRRDACVRKQIEFL

SYTEAFGMALAASAILGAVFTSAVAAIFLQHRGTPIVRANNSE

>CL1147.Contig5_All 1775 2707 olfactory receptor C family, d2 precursor [Danio rerio] >gi|118406874|gb|ABK81645.1| C-family odorant receptor OLFCD2 [Danio rerio]

DCISNLGDNNSLTDSNNCEPCPGQYWPNPGRDKCILKAVEFLLFTEVMGIVLVFFSLFGV

LITVITAIIFIIKKDTPIVKANNSELSFLLLFSLTLCFLCSLTFIGRPSEWSCMLRHTAF

GITFVLCISCVLGKTIVVLMAFRATLPGSNVMKWFGPLQQRLSVLGFTLIQVLICVLWLT

ISPPFPYRNMHLYNDKIILECDVGSAIGFWAILGYIGLLAILCFFLAFLARKLPDNFNEA

KFITFSMLIFCAVWITFIPAYVSSPGKFTVAVEIFAILASSFGLLLCIFTPKCYIILIKP

EKNTKKQLMGK

>Unigene8764_All 12 1649 olfactory receptor C family, d2 precursor [Danio rerio] >gi|118406874|gb|ABK81645.1| C-family odorant receptor OLFCD2 [Danio rerio]

MAQTPLLLLLLSLCARASLQSCSLLGQPALPLLSSEGDINIGAVFSLHREPVVKAHTFTS

EPEPTSCIRLNLREFQFGQTLIFAVEEINNNTNLLPGITLGYKIYDACSSIILGIRSAMA

LMNGYGQTLSDTSCQRPPAVQAIVGESGSTPTIGIASAVGPFRIPVVSHFATCACLSDKD

RFPTFFRTIPSDYYQSRALAQLVKLFGWTWIGAVRSRNDYGNNGMATFIEAAEELGVCVE

YSEAIFRTDPKEEVLKVVEVMKRATAKVVVAFLSLGDIIPLLNELALNNISGLQWVGSES

WITSRGLSETKSFSFLTGATGFVIGNVKLNGLKEFLVNVHPSKAPHNLFLREFWETAFQC

SFENSPVEGSRCTGSESLLTLQNQYTDVSELRISNKVYTAVYAIAHSLHNLMMDATGNDT

KWNVFVPEKVLEYLKKVNFTSKTGEQIFFDSRGDPAARYELVNWQPDDDGTLQFKSVGIY

DTSVPSEQRFVLNQGGLVWAGGQTQVPVSVCSESCPPGTRKAAQKGKAVCCYDCIPCGEG

EISNET

>CL11881.Contig2_All 1 423 olfactory receptor family C subfamily 16 member 3 [Salmo salar]

VIIGGLFEVHMLALQPELSYRSKPKQIWCQDFTLYGLKTVMAMAFAVEEINRNPNLLPTV

KLGYRMFDNCMRLDVAFRAAMALVSGTNKYSSTQNCSGLPPVLGIVGDPISSNSIAISSV

LGLFRVPMVSFYATCSCLSDR

>Unigene26668_All 45 2633 olfactory receptor family C subfamily 15 member 1 [Salmo salar]

SPSALLLQASAPLLLLLWLRGDAVVADMAEQEQSCVRWDKPEGVDDRGLSQDGDVVIGGL

FVVHNQPPSPDLSFTRNPGQDPCFSFQKEPYLWAHAMVFAVEEINRNPHLLPGVRLGYQI

LDSCTRYPWSVRAAMSLISGGNHSCESTGPVRVIVGDASSTQTIMLSRILSPLQVPLISY

QASCACLSNRLEFPNVFRTIPSDVYQAWTMAHLAEHYRWTWVGAVVVNNDYGLLAVQAFR

ERAQGSGICLAFFETFDRETLAQDMERIATTVQTSSARVVLVFAWYTDVGALLLELASRN

VTGRQFLASEAWSTSSYLLNDPALRTITSGVLGVAIRSAPIPGLEAHLRKLHPSQHPRDA

LLRDLWREEFGCEPVSASSPDSLSLPPCSGTESLERVQSTFTDTAYLRVTYNVYLAVYAA

AHAFHSLLECSPVGDSGMNRSQPCSSQYNISAAEVLQHLGQVNFTTQIEENFFFQGGDIP

AVYDLVNWQATPGGVLKYSTIGRVEGSKLHMEESAIWWATESQVPVSVCSEPCPPGTRKA

RRKGEPICCFDCLPCADGEISNKTDSLTCLRCPLEFWSNDQKNMCVPLTVEFLSFTDAMG

ITLTTVAVSGAVMTAAVAVVFVYNRHTPIVKANNSELSFMLLMSLKLCFLCSLLFMGQPT

DWSCRIQQAAFGISFVLSISCILVKTIVVLAVFQSARPGSDSVMKWFGPGQQRGSVLLFT

CVQVVICAVWLSITPPQPHRNVGLKGSKIILECTLGSVEGFATLLGYIGLLAAICFMLAF

LARKLPDNFNEAKFITFSMLIFCAVWITFVPAYVSSPGKYAIAVEIFAILASSFGLLLCI

FAPKCFIILLRPEKNTKKFLMGK

>Unigene82853_All 2 712 minus strand olfactory receptor family C subfamily 12 member 1 [Salmo salar]

NNSELSFLILVSLSLCFLCALPFIGEPTPWTCMLRHTAFSISFSLCIACILSKTVVVLMA

FKATLPGSVVMKWFGPVQQRAMISVGAAVQVVICGVWLTVAPPTPRKLSSRETTQIILLC

DEGSALAFSLVLSYIGLLASLSLLLAFLARKLPGSFNEAKLITFSMVIFFAVWVAFVPAY

VSSPGKYSTATEVFAILASSYALLVCLFFPKCYVILLKPEKNTRKHLMAKTAQDNRY

>Unigene86482_All 110 484 olfactory receptor family C subfamily 3 member 1 [Salmo salar]

VVGGIFPVSNKQNHLSASFEKEPPDATCNGFDMRAFRWTQVMIFAINEINSDSTLLPNIS

LGYRIFDSCASPTNTLRAALMLLSDPNGDNSTVQCQPPVSALIAESGSSQSMALAGTFGP

FNLPM

>Unigene88383_All 15 227 minus strand olfactory receptor family C subfamily 2 member 2 [Salmo salar]

LAPLLLQLIAMSTASTCILQGGFELPGFMSEGNFTIGGIFPLHYRVELPQTDFKTPPVSA

HCRGFDPRAFR

>Unigene97945_All 5 166 minus strand olfactory receptor family C subfamily 16 member 1 [Salmo salar]

NGDALPIYDILNWHGMPDGSMKAKTVGDVDESQPSDKVLSLEEDKIFWNFKSKK

>Unigene61719_All 14 619 C-family odorant receptor OLFCT1 [Danio rerio]

GACPNMSTIQPWQLLEYLKQVKFTNDFGEEMKFDENGDPAAMYDLINWQLTADGDVKYVT

VGKFDETTRNKLEIEDEAILWTGTTKEVPLSVCSKSCPPGTRRAIKPNFPVCCFDCIFCA

AGEISNQTDAIECEQCLPEFWSNTWRNSCVPKQVEFLSYSDTMGITLMVVAIVGFCCTLM

VVLIFACNKNTPIIRANNSELS

>Unigene78794_All 1 477 novel protein similar to vertebrate pheromone receptor [Danio rerio]

NNSELSFLLLLSLGLCFLCTLTFLGRPTSWACPLRRTSFGLSFALCLSCLLCKTLVVLLA

FKAKLPGDKTARWFHRPQQRLSVLVCSSLQVLLCVVWLARAPPYPIRNTWLYRDRVILEC

HMGSVALFSCVLGYIGCLAAFCLLLAFLARKLPDNFNEA

>CL10924.Contig1_All 7 255 minus strand uncharacterized protein LOC100004933 precursor [Danio rerio] >gi|157888737|emb|CAP09591.1| novel 7 transmembrane receptor (metabotropic glutamate family) protein [Danio rerio]

QVLQYLHGVNFTMPSGETVYFDQNGDPAARYELVNWQKNAAGDTVFVTVGKYDASLPEGN

QFAMNSLDIVWAGETKSVETNIC

>CL10924.Contig2_All 58 513 minus strand uncharacterized protein LOC100004933 precursor [Danio rerio] >gi|157888737|emb|CAP09591.1| novel 7 transmembrane receptor (metabotropic glutamate family) protein [Danio rerio]

KPTSVCSESCLPGYRQAVIRGRPVCCFSCVRCAEGEVSSKVDSTECRKCPLEFWSNEDHS

RCIPKQTEFLSFTENMGIILTTFSLVGACFTIAVAVVFFCYIDTPLVKASNLELSFLLLF

SLTLCFLCSLTFIGQPSDWSCRLRHTAFGITF

>CL1147.Contig10_All 5 2059 uncharacterized protein LOC100005028 precursor [Danio rerio] >gi|157888736|emb|CAP09590.1| novel 7 transmembrane receptor (metabotropic glutamate family) protein [Danio rerio]

ATCACLSNRKEFPSFFRTIPSDYYQSRALAKLVKHFGWTWVGAIRSRSDYGNGGMATFLS

AAEKEGVCVEYSVAIYRTDPREKFLEVVNIIKRSTSRVIVAFADGNDLDILLKELFLQNV

TGFQWVGSEGWITYRYVATQTNYAVLGGAVGFAVPNAVIPGLQEFMLAARPSLQPGNRGL

VEMWESVFDCSFDPNSQARACTGEESLGDTDTRFTDVSDASLLNNIYKAVYAVAYALDEL

LACEDGRGQFANQSCANKSKIEPWQVLHYLTLVNFTTKQRDSVSFDHQGDPAARYSLVNW

QRNMVGTITFESIGIYDASLQEGQQFMMKNDVSAVWAGGKQEVPRSICSESCLPGTRRAF

LKGKPICCFDCIRCADGEFSNTTNAVTCLSCPPEFKSNEERTQCDLKNTEYLTFKELMGI

LLVTFSVFGGCLTITIGLVFFQFRQTPIVRANNSELSFLLLFSLTLCFLCSLTFIGRPSE

WSCMLRHTAFGITFVLCISCVLGKTIVVLMAFRATLPGSNVMKWFGPLQQRLSVLGFTLI

QVLICVLWLTISPPFPYRNMHLYNDKIILECDVGSAIGFWAILGYIGLLAILCFILAFLA

RKLPDNFNEAKFITFSMLIFCAVWITFIPAYVSSPGKFTVAVEIFAILASSFGVLFCIFV

PKCYIIIFLPDHNTKKFLMSQKGGR

>CL1147.Contig11_All 1 2271 PREDICTED: extracellular calcium-sensing receptor-like [Danio rerio]

DILPNITLGYEIHDSCAAVPVAVKVAFQFANGLTPHVSMSASCPKTANIVAIVGESGSTP

SIGMSRILGLFGIPQVSHFATCACLSDKTQHPAFFRTIPSDHYQAAALARLIKHFGWTWI

GAVRSDSDYGNNGMASFLTAAQKEGICVEYSVAFYRTNSRSKLERVADVIRSSTARVIVA

FLASGDMRILLEELTRRPPPALQWIGSESWVTDPDMLRFELCAGAIGFGIERSVIPGLRH

FLLDLSPAQASKSAVLTELWESAFGCSLHTAEAGQISGTVAPCKGNEKLDAVQIPYTDTT

QLRVTNMAYKAAYAIAHAIHGLVCKDRSDSVSKCNKSTVLEPDQILQQLKKVNFSVNGNH

VSFDSNGDPVATYELVNWQAMQDGSVQFVTVGRYDASQPNGQEFSISKSITWMKGQTQVP

VSVCSESCPPGTRKAAQKGKPVCCYDCIPCGEGEISNETDSVDCVSCPVDYWPNAQRNIC

LPKPVEFLSWDEVLGIVLAVCSIAGACIAVTVATVFYKHRMSPIVRANNSELSFLLLFSL

TLCFLCSLTFIGRPSEWSCMLRHTAFGITFVLCISCVLGKTIVVLMAFRATLPGSNVMKW

FGPLQQRLSVLGFTLIQVLICVLWLTISPPFPYRNMHLYNDKIILECDVGSAIGFWAILG

YIGLLAILCFILAFLARKLPDNFNEAKFITFSMLIFCAVWITFIPAYVSSPGKFTVAVEI

FAILASSFGVLFCIFVPKCYIIIFLPDHNTKKFLMSQ

>CL1147.Contig12_All 1 2430 PREDICTED: extracellular calcium-sensing receptor-like [Danio rerio]

DILPNITLGYEIHDSCAAVPVAVKVAFQFANGLTPHVSMSASCPKTANIVAIVGESGSTP

SIGMSRILGLFGIPQVLSICCVLYIUUUPNSFUKTQMLYIVLAMSLTIFTIQMLIGYLLL

PFYTNNQKVSHFATCACLSDKTQHPAFFRTIPSDHYQAAALARLIKHFGWTWIGAVRSDS

DYGNNGMASFLTAAQKEGICVEYSVAFYRTNSRSKLERVADVIRSSTARVIVAFLASGDM

RILLEELTRRPPPALQWIGSESWVTDPDMLRFELCAGAIGFGIERSVIPGLRHFLLDLSP

AQASKSAVLTELWESAFGCSLHTAEAGQISGTVAPCKGNEKLDAVQIPYTDTTQLRVTNM

AYKAAYAIAHAIHGLVCKDRSDSVSKCNKSTVLEPDQILQQLKKVNFSVNGNHVSFDSNG

DPVATYELVNWQAMQDGSVQFVTVGRYDASQPNGQEFSISKSITWMKGQTQVPVSVCSES

CPPGTRKAAQKGKPVCCYDCIPCGEGEISNETDSVDCVSCPVDYWPNAQRNICLPKPVEF

LSWDEVLGIVLAVCSIAGACIAVTVATVFYKHRMSPIVRANNSELSFLLLFSLTLCFLCS

LTFIGRPSEWSCMLRHTAFGITFVLCISCVLGKTIVVLMAFRATLPGSNVMKWFGPLQQR

LSVLGFTLIQVLICVLWLTISPPFPYRNMHLYNDKIILECDVGSAIGFWAILGYIGLLAI

LCFILAFLARKLPDNFNEAKFITFSMLIFCAVWITFIPAYVSSPGKFTVAVEIFAILASS

FGVLFCIFVPKCYIIIFLPDHNTKKFLMSQ

>CL1147.Contig13_All 5 2047 uncharacterized protein LOC100005028 precursor [Danio rerio] >gi|157888736|emb|CAP09590.1| novel 7 transmembrane receptor (metabotropic glutamate family) protein [Danio rerio]

ATCACLSNRKEFPSFFRTIPSDYYQSRALAKLVKHFGWTWVGAIRSRSDYGNGGMATFLS

AAEKEGVCVEYSVAIYRTDPREKFLEVVNIIKRSTSRVIVAFADGNDLDILLKELFLQNV

TGFQWVGSEGWITYRYVATQTNYAVLGGAVGFAVPNAVIPGLQEFMLAARPSLQPGNRGL

VEMWESVFDCSFDPNSQARACTGEESLGDTDTRFTDVSDASLLNNIYKAVYAVAYALDEL

LACEDGRGQFANQSCANKSKIEPWQVLHYLTLVNFTTKQRDSVSFDHQGDPAARYSLVNW

QRNMVGTITFESIGIYDASLQEGQQFMMKNDVSAVWAGGKQEVPRSICSESCLPGTRRAF

LKGKPICCFDCIRCADGEFSNTTNAVTCLSCPPEFKSNEERTQCDLKNTEYLTFKELMGI

LLVTFSVFGGCLTITIGLVFFQFRQTPIVRANNSELSFLLLFSLTLCFLCSLTFIGRPSE

WSCMLRHTAFGITFVLCISCVLGKTIVVLMAFRATLPGSNVMKWFGPLQQRLSVLGFTLI

QVLICVLWLTISPPFPYRNMHLYNDKIILECDVGSAIGFWAILGYIGLLAILCFILAFLA

RKLPDNFNEAKFITFSMLIFCAVWITFIPAYVSSPGKFTVAVEIFAILASSFGLLLCIFT

PKCYIILIKPEKNTKKQLMGK

>CL1147.Contig9_All 5 2179 uncharacterized protein LOC100005028 precursor [Danio rerio] >gi|157888736|emb|CAP09590.1| novel 7 transmembrane receptor (metabotropic glutamate family) protein [Danio rerio]

ATCACLSNRKEFPSFFRTIPSDYYQSRALAKLVKHFGWTWVGAIRSRSDYGNGGMATFLS

AAEKEGVCVEYSVAIYRTDPREKFLEVVNIIKRSTSRVIVAFADGNDLDILLKELFLQNV

TGFQWVGSEGWITYRYVATQTNYAVLGGAVGFAVPNAVIPGLQEFMLAARPSLQPGNRGL

VEMWESVFDCSFDPNSQARACTGEESLGDTDTRFTDVSDASLLNNIYKAVYAVAYALDEL

LACEDGRGQFANQSCANKSKIEPWQVLHYLTLVNFTTKQRDSVSFDHQGDPAARYSLVNW

QRNMVGTITFESIGIYDASLQEGQQFMMKNDVSAVWAGGKQEVPRSICSESCLPGTRRAF

LKGKPICCFDCIRCADGEFSNTTSETAKLLSFILMMLMLLCGKVTLSTLVRYVKMATLPT

FNVFVLSDAVTCLSCPPEFKSNEERTQCDLKNTEYLTFKELMGILLVTFSVFGGCLTITI

GLVFFQFRQTPIVRANNSELSFLLLFSLTLCFLCSLTFIGRPSEWSCMLRHTAFGITFVL

CISCVLGKTIVVLMAFRATLPGSNVMKWFGPLQQRLSVLGFTLIQVLICVLWLTISPPFP

YRNMHLYNDKIILECDVGSAIGFWAILGYIGLLAILCFILAFLARKLPDNFNEAKFITFS

MLIFCAVWITFIPAYVSSPGKFTVAVEIFAILASSFGLLLCIFTPKCYIILIKPEKNTKK

QLMGK

>CL1147.Contig2_All 63 2495 PREDICTED: extracellular calcium-sensing receptor-like, partial [Oreochromis niloticus]

EKPLCQLSGIPVLPLLSNKGDVIIGGAFSIHSKITQPQLTFTEQPKPISCSSVNLREFRF

AQTMIFTIEEINRSGALLPNVTIGYRIYDNCGSTLSSMRAVMALMNGHELTAEDPCTGQS

AVHAIIGESESSSTIVLSRTTGPFNIPVISHSATCECLSSRREFPSFFRTIASDYYQSRA

LAQLVKHFGWTWVGAVNSDSDYGNNGMAIFLEAAEEEGICVEYSEKFHRTEPDKLLKVVD

VIREGSAKVIVAFLAHVEMNNLLEQFTLQNITGLQMIGVEAWITADSLITPSSFSILGGS

LGFAVGKANLSGFDEFVKQFWEKTFQCSENMSQDENQCSRYQDLLEWRHYNDDVPELRYS

SNIYKAIYAVAYSMHSLLNCRANVGCDKVMQTKPQKVVESLKTVNFTLKTKEQVWFDSTG

AAAARYEVVNWQRGPDGTVRFKPVGHYDVSLPVGHRFVLSEDTIVWPGGSAQRPVSVCSE

SCPPGTRKAVQKGKPVCCYQCVPCGAGEISNTTDATDCLKCPPTYWSNWKGDHCLLKTVE

FLSYTELMGILLTLFSLLGATLTILIAAVFCHFRHTPIVKANNSELSFLLLFSLTLCFLC

SLTFIGRPSEWSCMLRHTAFGITFVLCISCVLGKTIVVLMAFRATLPGSNVMKWFGPLQQ

RLSVLGFTLIQVLICVLWLTISPPFPYRNMHLYNDKIILECDVGSAIGFWAILGYIGLLA

ILCFILAFLARKLPDNFNEAKFITFSMLIFCAVWITFIPAYVSSPGKFTVAVEIFAILAS

SFGLLLCIFTPKCYIILIKPEKNTKKQLMGK

>CL1147.Contig3_All 63 2495 PREDICTED: extracellular calcium-sensing receptor-like, partial [Oreochromis niloticus]

EKPLCQLSGIPVLPLLSNKGDVIIGGAFSIHSKITQPQLTFTEQPKPISCSSVNLREFRF

AQTMIFTIEEINRSGALLPNVTIGYRIYDNCGSTLSSMRAVMALMNGHELTAEDPCTGQS

AVHAIIGESESSSTIVLSRTTGPFNIPVISHSATCECLSSRREFPSFFRTIASDYYQSRA

LAQLVKHFGWTWVGAVNSDSDYGNNGMAIFLEAAEEEGICVEYSEKFHRTEPDKLLKVVD

VIREGSAKVIVAFLAHVEMNNLLEQFTLQNITGLQMIGVEAWITADSLITPSSFSILGGS

LGFAVGKANLSGFDEFVKQFWEKTFQCSENMSQDENQCSRYQDLLEWRHYNDDVPELRYS

SNIYKAIYAVAYSMHSLLNCRANVGCDKVMQTKPQKVVESLKTVNFTLKTKEQVWFDSTG

AAAARYEVVNWQRGPDGTVRFKPVGHYDVSLPVGHRFVLSEDTIVWPGGSAQRPVSVCSE

SCPPGTRKAVQKGKPVCCYDCIPCGAGEISNTTDATDCLKCPPTYWSNWKGDHCLLKTVE

FLSYTELMGILLTLFSLLGATLTILIAAVFCHFRHTPIVKANNSELSFLLLFSLTLCFLC

SLTFIGRPSEWSCMLRHTAFGITFVLCISCVLGKTIVVLMAFRATLPGSNVMKWFGPLQQ

RLSVLGFTLIQVLICVLWLTISPPFPYRNMHLYNDKIILECDVGSAIGFWAILGYIGLLA

ILCFILAFLARKLPDNFNEAKFITFSMLIFCAVWITFIPAYVSSPGKFTVAVEIFAILAS

SFGVLFCIFVPKCYIIIFLPDHNTKKFLMSQ

>CL1147.Contig4_All 1745 2659 PREDICTED: extracellular calcium-sensing receptor-like, partial [Oreochromis niloticus]

GDNNSLTDSNNCEPCPGQYWPNPGRDKCILKAVEFLLFTEVMGIVLVFFSLFGVLITVIT

AIIFIIKKDTPIVKANNSELSFLLLFSLTLCFLCSLTFIGRPSEWSCMLRHTAFGITFVL

CISCVLGKTIVVLMAFRATLPGSNVMKWFGPLQQRLSVLGFTLIQVLICVLWLTISPPFP

YRNMHLYNDKIILECDVGSAIGFWAILGYIGLLAILCFILAFLARKLPDNFNEAKFITFS

MLIFCAVWITFIPAYVSSPGKFTVAVEIFAILASSFGVLFCIFVPKCYIIIFLPDHNTKK

FLMSQ

>CL1147.Contig6_All 1 2271 PREDICTED: extracellular calcium-sensing receptor-like [Danio rerio]

DILPNITLGYEIHDSCAAVPVAVKVAFQFANGLTPHVSMSASCPKTANIVAIVGESGSTP

SIGMSRILGLFGIPQVSHFATCACLSDKTQHPAFFRTIPSDHYQAAALARLIKHFGWTWI

GAVRSDSDYGNNGMASFLTAAQKEGICVEYSVAFYRTNSRSKLERVADVIRSSTARVIVA

FLASGDMRILLEELTRRPPPALQWIGSESWVTDPDMLRFELCAGAIGFGIERSVIPGLRH

FLLDLSPAQASKSAVLTELWESAFGCSLHTAEAGQISGTVAPCKGNEKLDAVQIPYTDTT

QLRVTNMAYKAAYAIAHAIHGLVCKDRSDSVSKCNKSTVLEPDQILQQLKKVNFSVNGNH

VSFDSNGDPVATYELVNWQAMQDGSVQFVTVGRYDASQPNGQEFSISKSITWMKGQTQVP

VSVCSESCPPGTRKAAQKGKPVCCYDCIPCGEGEISNETDSVDCVSCPVDYWPNAQRNIC

LPKPVEFLSWDEVLGIVLAVCSIAGACIAVTVATVFYKHRMSPIVRANNSELSFLLLFSL

TLCFLCSLTFIGRPSEWSCMLRHTAFGITFVLCISCVLGKTIVVLMAFRATLPGSNVMKW

FGPLQQRLSVLGFTLIQVLICVLWLTISPPFPYRNMHLYNDKIILECDVGSAIGFWAILG

YIGLLAILCFILAFLARKLPDNFNEAKFITFSMLIFCAVWITFIPAYVSSPGKFTVAVEI

FAILASSFGLLLCIFTPKCYIILIKPEKNTKKQLMGK

>CL1147.Contig7_All 1 2430 PREDICTED: extracellular calcium-sensing receptor-like [Danio rerio]

DILPNITLGYEIHDSCAAVPVAVKVAFQFANGLTPHVSMSASCPKTANIVAIVGESGSTP

SIGMSRILGLFGIPQVLSICCVLYIUUUPNSFUKTQMLYIVLAMSLTIFTIQMLIGYLLL

PFYTNNQKVSHFATCACLSDKTQHPAFFRTIPSDHYQAAALARLIKHFGWTWIGAVRSDS

DYGNNGMASFLTAAQKEGICVEYSVAFYRTNSRSKLERVADVIRSSTARVIVAFLASGDM

RILLEELTRRPPPALQWIGSESWVTDPDMLRFELCAGAIGFGIERSVIPGLRHFLLDLSP

AQASKSAVLTELWESAFGCSLHTAEAGQISGTVAPCKGNEKLDAVQIPYTDTTQLRVTNM

AYKAAYAIAHAIHGLVCKDRSDSVSKCNKSTVLEPDQILQQLKKVNFSVNGNHVSFDSNG

DPVATYELVNWQAMQDGSVQFVTVGRYDASQPNGQEFSISKSITWMKGQTQVPVSVCSES

CPPGTRKAAQKGKPVCCYDCIPCGEGEISNETDSVDCVSCPVDYWPNAQRNICLPKPVEF

LSWDEVLGIVLAVCSIAGACIAVTVATVFYKHRMSPIVRANNSELSFLLLFSLTLCFLCS

LTFIGRPSEWSCMLRHTAFGITFVLCISCVLGKTIVVLMAFRATLPGSNVMKWFGPLQQR

LSVLGFTLIQVLICVLWLTISPPFPYRNMHLYNDKIILECDVGSAIGFWAILGYIGLLAI

LCFILAFLARKLPDNFNEAKFITFSMLIFCAVWITFIPAYVSSPGKFTVAVEIFAILASS

FGLLLCIFTPKCYIILIKPEKNTKKQLMGK

>CL1147.Contig8_All 5 2191 uncharacterized protein LOC100005028 precursor [Danio rerio] >gi|157888736|emb|CAP09590.1| novel 7 transmembrane receptor (metabotropic glutamate family) protein [Danio rerio]

ATCACLSNRKEFPSFFRTIPSDYYQSRALAKLVKHFGWTWVGAIRSRSDYGNGGMATFLS

AAEKEGVCVEYSVAIYRTDPREKFLEVVNIIKRSTSRVIVAFADGNDLDILLKELFLQNV

TGFQWVGSEGWITYRYVATQTNYAVLGGAVGFAVPNAVIPGLQEFMLAARPSLQPGNRGL

VEMWESVFDCSFDPNSQARACTGEESLGDTDTRFTDVSDASLLNNIYKAVYAVAYALDEL

LACEDGRGQFANQSCANKSKIEPWQVLHYLTLVNFTTKQRDSVSFDHQGDPAARYSLVNW

QRNMVGTITFESIGIYDASLQEGQQFMMKNDVSAVWAGGKQEVPRSICSESCLPGTRRAF

LKGKPICCFDCIRCADGEFSNTTSETAKLLSFILMMLMLLCGKVTLSTLVRYVKMATLPT

FNVFVLSDAVTCLSCPPEFKSNEERTQCDLKNTEYLTFKELMGILLVTFSVFGGCLTITI

GLVFFQFRQTPIVRANNSELSFLLLFSLTLCFLCSLTFIGRPSEWSCMLRHTAFGITFVL

CISCVLGKTIVVLMAFRATLPGSNVMKWFGPLQQRLSVLGFTLIQVLICVLWLTISPPFP

YRNMHLYNDKIILECDVGSAIGFWAILGYIGLLAILCFILAFLARKLPDNFNEAKFITFS

MLIFCAVWITFIPAYVSSPGKFTVAVEIFAILACSFGVLFCIFVPKCYIIIFLPDHNTKK

FLMSQKGGR

>CL1147.Contig9_All 5 2179 uncharacterized protein LOC100005028 precursor [Danio rerio] >gi|157888736|emb|CAP09590.1| novel 7 transmembrane receptor (metabotropic glutamate family) protein [Danio rerio]

ATCACLSNRKEFPSFFRTIPSDYYQSRALAKLVKHFGWTWVGAIRSRSDYGNGGMATFLS

AAEKEGVCVEYSVAIYRTDPREKFLEVVNIIKRSTSRVIVAFADGNDLDILLKELFLQNV

TGFQWVGSEGWITYRYVATQTNYAVLGGAVGFAVPNAVIPGLQEFMLAARPSLQPGNRGL

VEMWESVFDCSFDPNSQARACTGEESLGDTDTRFTDVSDASLLNNIYKAVYAVAYALDEL

LACEDGRGQFANQSCANKSKIEPWQVLHYLTLVNFTTKQRDSVSFDHQGDPAARYSLVNW

QRNMVGTITFESIGIYDASLQEGQQFMMKNDVSAVWAGGKQEVPRSICSESCLPGTRRAF

LKGKPICCFDCIRCADGEFSNTTSETAKLLSFILMMLMLLCGKVTLSTLVRYVKMATLPT

FNVFVLSDAVTCLSCPPEFKSNEERTQCDLKNTEYLTFKELMGILLVTFSVFGGCLTITI

GLVFFQFRQTPIVRANNSELSFLLLFSLTLCFLCSLTFIGRPSEWSCMLRHTAFGITFVL

CISCVLGKTIVVLMAFRATLPGSNVMKWFGPLQQRLSVLGFTLIQVLICVLWLTISPPFP

YRNMHLYNDKIILECDVGSAIGFWAILGYIGLLAILCFILAFLARKLPDNFNEAKFITFS

MLIFCAVWITFIPAYVSSPGKFTVAVEIFAILASSFGLLLCIFTPKCYIILIKPEKNTKK

QLMGK

>CL5324.Contig1_All 328 1467 unnamed protein product [Tetraodon nigroviridis]

VEEINRSKGLLPNHTLGYKILDSCAYPLTGQRAVLAILNGPGEVGSPLCSGAGPLVAVIG

ESGSAQSIVVSRILQPFRIPMISYFSSCACLGDRKEFPTFFRVIPSDDYQVKAIAQLLQH

FNWTWVGVVRGDHEYGRFALQGLLRELEGTGVCVAYQEMIPLLYDSQRALEIIHVMRTSS

ARVVVVFSAEGELTPFLRDYIEQNVTGIQWIASEAWVTSSVFTGKEYYPFLGGTIGFGIR

QGQIPALRDYLNTVDPWRYPTNRLVRELWETLYGCSPNNTARNTQFPVCVGYESLREQHS

AYMNTSSPRISYNVYKGVYAVAHSLHNLMLCKNGQGPFKNSSCADLNNIYPWQEAGKELL

IEDKMIVWAGHRSKASCSQC

>CL5324.Contig2_All 328 1629 minus strand unnamed protein product [Tetraodon nigroviridis]

VEEINRSKGLLPNHTLGYKILDSCAYPLTGQRAVLAILNGPGEVGSPLCSGAGPLVAVIG

ESGSAQSIVVSRILQPFRIPMISYFSSCACLGDRKEFPTFFRVIPSDDYQVKAIAQLLQH

FNWTWVGVVRGDHEYGRFALQGLLRELEGTGVCVAYQEMIPLLYDSQRALEIIHVMRTSS

ARVVVVFSAEGELTPFLRDYIEQNVTGIQWIASEAWVTSSVFTGKEYYPFLGGTIGFGIR

QGQIPALRDYLNTVDPWRYPTNRLVRELWETLYGCSPNNTARNTQFPVCVGYESLREQHS

AYMNTSSPRISYNVYKGVYAVAHSLHNLMLCKNGQGPFKNSSCADLNNIYPWQLQHYLQE

VSFTISGEEVNFDDKGDSIPSYDLINWQRGHEGNIEFINVGLFDAAQEAGKELLIEDKMI

VWAGHRSKASCSQC

>CL6778.Contig1_All 25 1482 pheromone receptor [Danio rerio] >gi|126632712|emb|CAM56593.1| novel pheromone receptor protein [Danio rerio]

ERPLCTGAEDIQEKANAYSDVTQLGVSYNVYKAVYAVAHAIQDMLACQPGQGPFENGKCP

SINNIIPKQLLHYLERVNFTTPLGDIVNFDMNGDPPASYDLINWHIGDKGMAEFVKVGQY

DSNIGPDQRLQLDLNKVVWGGGWTDKVPVSVCSIPCAPGTWKALQKGKPVCCFDCIPCPD

GEISNKTGATECTRCPEQFWSNNLRTQCILKREEFLSFYEPLGIILTVLSISGAMLTTVV

LVTFILHRDTPLVRANNSELSFLLLLSLILCFLCAMSFMGRPLAWSCMLRHTLFGISFVV

CISCILSKTVVVLVAFRATLPGSKVMRYFGPLQQRLGICLCTLVQVLVCVLWLTLDPPLP

SQNSATVRSATVVLECASRSLAGFAALLSYIGLLATVCFLLAFFARRLPDNFNEAKFITF

SMLIFCAVWIAFVPAYVSSPGKYTVAVEIFAILASSYGLLLCIFAPKCYIILFQSQKNTK

KNMMAK

>CL6778.Contig2_All 25 477 pheromone receptor [Danio rerio] >gi|126632712|emb|CAM56593.1| novel pheromone receptor protein [Danio rerio]

ERPLCTGAEDIQEKANAYSDVTQLGVSYNVYKAVYAVAHAIQDMLACQPGQGPFENGKCP

SINNIIPKQLLHYLERVNFTTPLGDIVNFDMNGDPPASYDLINWHIGDKGMAEFVKVGQY

DSNIGPDQRLQLDLNKVVWGGGWTDKVKYQI

>CL6778.Contig3_All 666 788 pheromone receptor [Danio rerio] >gi|126632712|emb|CAM56593.1| novel pheromone receptor protein [Danio rerio]

VPVSVCSIPCAPGTWKALQKGKPVCCFDCIPCPDGEISNKT

>CL6778.Contig4_All 25 585 pheromone receptor [Danio rerio] >gi|126632712|emb|CAM56593.1| novel pheromone receptor protein [Danio rerio]

ERPLCTGAEDIQEKANAYSDVTQLGVSYNVYKAVYAVAHAIQDMLACQPGQGPFENGKCP

SINNIIPKQLLHYLERVNFTTPLGDIVNFDMNGDPPASYDLINWHIGDKGMAEFVKVGQY

DSNIGPDQRLQLDLNKVVWGGGWTDKVPVSVCSIPCAPGTWKALQKGKPVCCFDCIPCPD

GEISNKT

>Unigene15989_All 1 405 minus strand PREDICTED: extracellular calcium-sensing receptor [Danio rerio]

IWLATRPPYAVHNTRFLSAKIILECVVGSEVGFWCVLGYIGLLACMCFLMAFLARKLPDN

FNEAKFITFSMLIFFAVWITFIPVYVSTSGKYTVAVHVFAILASTFGLLFCIFMPKCYII

LLKPEKNSKQHMMRK

>Unigene54792_All 2 325 minus strand uncharacterized protein LOC100004933 precursor [Danio rerio] >gi|157888737|emb|CAP09591.1| novel 7 transmembrane receptor (metabotropic glutamate family) protein [Danio rerio]

TAFGITFALCLSCVLSKTVTVVMAFKASVPGARVPQCSLPFQRLSVMCCTLLQVIICALW

IALAPPVPQKNMAFFSNKIILECGLGSAIGFWAVLGYIGILAILCFIL

>Unigene61739_All 2 757 minus strand PREDICTED: extracellular calcium-sensing receptor-like [Oreochromis niloticus]

PSDYYQSRALAKLVKHFGWTWVGAVRSDNDYGNNGMATFVEAAQREGVCIEYSEAILRTN

SRERIASVVEQIKMGTARVLVAFLAQSEMEVLLEEALLQNVTGLQWIASESWITARYLAT

HRTAKIISGALGFSISRSNIPGLKDFLLKVNPSENSSILREFWETAFQCQLPGQSETKNI

RRCSGSENLGELKNPFTDVSELRISNNVYKAVYTVAHAVQNFLGCTDDEHTSDNKAENRE

CILRENIMPGQV

>Unigene62553_All 2 499 minus strand PREDICTED: extracellular calcium-sensing receptor [Danio rerio]

TDSVNCLRCSEDTWPNQAQDQCIPKNIEFLSYHETMGIVLWVVSAFGAFTTVAVLGVFVA

YRKTPMVRANNMELSFLLLLFLCACFLIGLTFIGEPTDWFCQFRYTAFGISFALCISCIL

AKTVVVMMAFRATLPGSNVMKWFGPAKQRASVIVCTSIQVLICVIW

>Unigene78793_All 18 560 minus strand uncharacterized protein LOC100005028 precursor [Danio rerio] >gi|157888736|emb|CAP09590.1| novel 7 transmembrane receptor (metabotropic glutamate family) protein [Danio rerio]

VYLTALLTLLNATHACRAYGSQELLHFSKKGDVNIGGIFSFHQNPAGVTPSLQVNPGHAK

CEGLDLGELQYAMTMIFAIEEINNSTELLPGFTLGYKIFGSCPSIPLSVDASLALMNGPV

SENCVRPDTVHAVIGETTSTATIGIARTMGPFHIPVLSHSATCACLSDRKEFPSFFRTIP

S

>CL14841.Contig2_All 182 439 minus strand PREDICTED: extracellular calcium-sensing receptor-like [Oreochromis niloticus]

KLGYRIYNACGYSNIMRSAIALASGVAKVIDGKNCSNTFRAEAIIGHSASAPTVGFARVL

GLFQLPVISHFATCPCLSNRKEFPSF

>Unigene24070_All 3 272 minus strand PREDICTED: extracellular calcium-sensing receptor-like [Danio rerio]

ILAFLARRLPDNFNEAKFITFSMLIFCSVWIAFIPAYVSSPGKFTVAVEIFAILASSFGV

LFCIFIPKCYIILYKPDKNTKRHLMGKMPS

>Unigene28241_All 1 144 PREDICTED: extracellular calcium-sensing receptor-like, partial [Oreochromis niloticus]

FTVAVEIFAILASSYGMLFCIFLPKCYIIVFQPEKNTKRHLMGKVTTK

>Unigene65091_All 3 1283 minus strand novel protein similar to vertebrate phermone receptor protein [Danio rerio]

EEINRSKDLLPNHTLGYKILDSCATPVTAQRAVLAVLNGEDSREKTMCSASSPLLAVIGE

SGSSQSIVVSRTLQPFRIPMISYFSTCACLSDRTQFPTFFRVVPSDDYQVKAVAQLLRHF

GWTWIGVVTEDHDYGRFALQGLKKEIENTDICLAYHEMIPKDYSTEKVLKILEVMTMSTA

RVVVVFSGEGEFYPFLKELSKQNITGIQWIASEAWVSASVLAETYPFLDGTIGFAVRKGY

VPNLRDYVTTVNPWNYPSNVLVQELWETLYACSPPSATTTNTQLSPCTGHETVQEQHSAY

MNTTSPRVAYNVYKGVYAIAHSLHNLIQCTPGHGPFLNGSCADVSTIQPWQLQHYLQDVS

FFISGEKVNFDMKGDSIPSYDLINWQRDTYGQIQFVTVGLYDGAKVAGKELVIDKDTIKW

TGQRKEA

>Unigene77993_All 2 166 PREDICTED: extracellular calcium-sensing receptor-like [Oreochromis niloticus]

FVPAYVSSPGKYAIAVEIFAILSSSFGLLLCIFAPKCFIILIQPHKNTKKFLMGK

>CL10326.Contig1_All 138 2420 minus strand PREDICTED: extracellular calcium-sensing receptor-like [Danio rerio]

CQLREEFNLTGMHQDGDVIIGGLFEINLFTVFPMLSFRTKPDQPWCYKSDLEGFKMAQTM

AFAINEINQNPNLLSNISLGYSIYDNCVKLGVAFRAALALISGTQKTLSSSDCREGLPPV

LGIVGDSVSTHSIAISSVAGLFHVPMVSYFATCSCLSDRQRYPSFFRTIPSDAFQVRAMI

QILRHFKWTWVGLIYSNDDYGRHAAQSFHKDITTFGGCVAFSEMLPKDNDIVEIRKIMQV

IRESTSRVIVVFSTKSYLLPLMDEVAQQNVIRQWIASEAWSSSPLFLTPRMLPFLKGTLG

ITIRRGEIDGLREFLLSVRPDNDSKNNMVRRFWQETFSCRFDPKEALAVAEGKVCTGQED

LSKADMAYSDVSDLRPSYNVYKAVYALAHSLHNLLSCVPGQGPFKGNSCASLHDMQPWQP

PRSVCSEPCPPGTRKATRKGLPVCCFDCLHCADGEISNTTDAIECVKCPVEFWSSLKKDR

CVPKEVEFLSYEEALGISLTTVSIFGACVSAVVLAIFVHHRHTPVVRANNSELSFLILLS

LKLCFLCALLFIGQPRTWTCQLRHAAFGISFVLCVSSILVKTMVVIAVFKASRPEGQGAM

KWFGAAQQRGTIFILTSLQVAICVIWLSTASPTPHKNTHYQSSKIVFECAVGSVAGFATL

LGYIGLLAAISFLLAFLARNLPDNFNEAKFITFSMLIFCAVWIAFVPAYVSSPGKYADVV

EIFAILASSFGLLIAIFAPKCYIILLHPERNTKKALMSRGS

>CL10326.Contig2_All 72 2642 minus strand novel protein similar to vertebrate pheromone receptor protein [Danio rerio]

WTVSAATLLSPALTSSSDTPNLCQLREEFNLTGMHQDGDVIIGGLFEINLFTVFPMLSFR

TKPDQPWCYKSDLEGFKMAQTMAFAINEINQNPNLLSNISLGYSIYDNCVKLGVAFRAAL

ALISGTQKTLSSSDCREGLPPVLGIVGDSVSTHSIAISSVAGLFHVPMVSYFATCSCLSD

RQRYPSFFRTIPSDAFQVRAMIQILRHFKWTWVGLIYSNDDYGRHAAQSFHKDITTFGGC

VAFSEMLPKDNDIVEIRKIMQVIRESTSRVIVVFSTKSYLLPLMDEVAQQNVIRQWIASE

AWSSSPLFLTPRMLPFLKGTLGITIRRGEIDGLREFLLSVRPDNDSKNNMVRRFWQETFS

CRFDPKEALAVAEGKVCTGQEDLSKADMAYSDVSDLRPSYNVYKAVYALAHSLHNLLSCV

PGQGPFKGNSCASLHDMQPWQLTHYLQDVNFSTGFGDQVSFDKTGDALAIYDIFNWHGMP

DGTMKARTVGVVDESQPTENVFHLDEGNLSWNFEFNKPPRSVCSEPCPPGTRKATRKGLP

VCCFDCLHCADGEISNTTDAIECVKCPVEFWSSLKKDRCVPKEVEFLSYEEALGISLTTV

SIFGACVSAVVLAIFVHHRHTPVVRANNSELSFLILLSLKLCFLCALLFIGQPRTWTCQL

RHAAFGISFVLCVSSILVKTMVVIAVFKASRPEGQGAMKWFGAAQQRGTIFILTSLQVAI

CVIWLSTASPTPHKNTHYQSSKIVFECAVGSVAGFATLLGYIGLLAAISFLLAFLARNLP

DNFNEAKFITFSMLIFCAVWIAFVPAYVSSPGKYADVVEIFAILASSFGLLIAIFAPKCY

IILLHPERNTKKALMSR

>CL10326.Contig3_All 2 280 minus strand vomeronasal 2 receptor, h32 precursor [Danio rerio] >gi|94733466|emb|CAK05324.1| novel protein similar to vertebrate phermone receptor protein [Danio rerio]

PPGTRKATRKGLPVCCFDCLPCADGEISNTTDSLECFRCPKEFWSSPEKDRCVPKEVEFL

SYEEALGISLTTVSIFGACIAAVVLGIFVHHRH

>CL14841.Contig1_All 2 529 minus strand pheromone receptor [Takifugu rubripes]

FALVSPLSGAEPVCKLLGEALVPELEGQGDLVIGGIFTFRTGYDGEIPTFESLPNEPKCK

NLNYREFKFAHTVIFAVEEINKNPKILPQHRLGYRIYNACGYSNIMRSAIALASGVAKVI

DGKNCSNTFRAEAIIGHSASAPTVGFARVLGLFQLPVISHFATCPCLSNRKEFPSF

>Unigene10816_All 3 500 minus strand olfactory receptor family C subfamily 17 member 1 [Salmo salar]

KWFGPLQQRLSVLGFTLIQVLICVLWLTISPPFPYRNMHLYNDKIILECDVGSAIGFWAI

LGYIGLLAILCFFLAFLARKLPDNFNEAKFITFSMLIFCAVWITFIPAYVSSPGKFTVTV

EIFAILASSSGLLFCIFLPKCYVILVRPDLNTKKHVMGKTSTDIHY

>Unigene1953_All 3 479 minus strand olfactory receptor family C subfamily 17 member 2 [Salmo salar]

KWFGPLQQRLSVLGFTLIQVLICVLWLTISPPFPYRNMHLYNDKIILECDVGSAIGFWAI

LGYIGLLAILCFILAFLARKLPDNFNEAKFITFSMLIFCAVWITFIPAYVSSPGKFTVAV

EIFAILASSFGLVFCIFIPKCYVIICRPHENTRKHIMQK

>Unigene51872_All 3 395 olfactory receptor family C subfamily 12 member 1 [Salmo salar]

WMQTMIFAIEEINKNPNLLPSITLGYQIYDSCSTPVQALRTALAVTGGQFEEESGNLKCS

GTVPVVIGDGGSTLSLVVARFLGVFHVPQVSYFSSCACLSNKVEFPAFLRTMPSDFFQVD

ALAQLVKHFGW

>Unigene66147_All 1 582 olfactory receptor family C subfamily 3 member 1 [Salmo salar]

CMLRHTAFAVTFALCISCVLGKTLVVITAFRATLPGATKFGPVQQRVIVYSCTVVQVFIC

ILWLTITPPYPQKRHNNRKIIVECNTGSDTAFYAVLGYIGFLAGICLVLAFLARKLPDNF

NEAKFITFSMLVFCAVWITFIPAYVSSPGKYTVAVEIFAILSSAFGLLVCIFAPKCYIII

FHSEKNTRQHVMGK

>Unigene71950_All 88 1170 olfactory receptor family C subfamily 11 member 4 [Salmo salar]

WLLLVLCGQAWVGAEEEKGVCRLQWRTVSNSLYREGDVIIGGLFPLHVVAPELDLSFKGK

VTTAVCQRFILWHYQWMQTMVFAIEEINQSPSLLPNLTLGYLAFDSCLAEHTTVGAALAM

VAGQEDAVSGSSCTGGPQVPVIIGDPRSSASIAVARTLGIFDIPLVSYFASCACLSDRQK

YPTFFRTVPSDAFQAKELARLLQLLGWVWIGVAFGDDDYGRYGVQLLLKELQGSDVCVAF

SQVIPKAHSPRRIRHIVETIRLSTAKVVVVFAISQDAQPLLEEAVRQNVSDRQWIGSEGW

VTSSDISTPQNLPSLVGTLGFALRKAQIPGLGPFLIRIRPAGSKTEPFVRKFWETLFECS

L

>CL10326.Contig3_All 2 280 minus strand vomeronasal 2 receptor, h32 precursor [Danio rerio] >gi|94733466|emb|CAK05324.1| novel protein similar to vertebrate phermone receptor protein [Danio rerio]

PPGTRKATRKGLPVCCFDCLPCADGEISNTTDSLECFRCPKEFWSSPEKDRCVPKEVEFL

SYEEALGISLTTVSIFGACIAAVVLGIFVHHRH

>Unigene55404_All 18 257 minus strand olfactory receptor family C subfamily 4 member 6 [Salmo salar]

LVALWGVGLTPVESGTNLVTCRLQGSTKFPAFSKDGDFVIGGIFSIHYYMHTVQHNYTRV

PEPLKCTGSMDTRELRFARA

>CL16219.Contig1_All 104 1093 PREDICTED: trace amine-associated receptor 7g-like [Danio rerio]

DHTVVNYCFQTLNNSCTKDIRAQGEYIILYILLFLISASTVFLNLLVVIAVSHFKQLHTP

TNLLLLSLAVADLLVGFLVMPVEGMRLIERCWYFGDTFCYIFPLILFVVISASLGNLVFI

SVDRYIAVSNALNYYSYITLNKAVLCIFFSWFGSFVYSVLILSNHLLQPEPHRTCHGECL

LVINFSWIIADLFVSFTVPCSIVICLYLKIFSMAKHQTLAINSVRNPGMTTNENVKIRKS

VNKAARTLGILVAVYLLCWIPYYVSILANGSISSSSFIVTFLSWTMYMNSCMNPLIYALF

YPWFRVSIKRILTLGILDPTSPYYSVYPDD

>CL16219.Contig2_All 2 826 PREDICTED: trace amine-associated receptor 7c-like [Danio rerio]

FKQLHTPTNLLILSLAMADLHVGLLVMPVEGMRLIETCWYFGDAFCYVFPVIMFVVVSAS

LGNLVFISVDRYIAVNNPLKYYSCVTPNKAMLCISLSWFGSFVYAMLMLSDHLQSKPQRA

CHGECLIVFSVPRVISDVVFSFLAPCSIVICLYVKIFFMAKYQTRVMNSVKNANMTTEHV

KFRKSESKAAKTLGIVVAIYLLCWIPYYICTLAYGSASSTSVVLTFLIWFMYINSCMNPL

IYALFYPWFRVSAKHILTLAILDPGSQYYSLNPDD

>CL337.Contig1_All 162 1022 minus strand PREDICTED: trace amine-associated receptor 7a-like [Danio rerio]

RPSETAVRVILYLILTAVILLTVCGNLLVIISVCHFKELHTPTNVLILSLAFSDILTGLT

VMPFHFVWMIELCWIFGAAMWILYSFATCLLSSQSVYNVTLLALDRYVALNYPFFYLDKV

TLKRSVIIASLSWLFSVFYNYILLYFNGFFTVSPTEEEGLNSSNEILFFADVMVVFVFPC

CAMIFLYFRIFAIAQRHTREIKKVNNAMRLKVVDCEIELDQKSERKAAKIIGIVVFVFLL

SMVPYYISTLLSGAVNDKLLNIIVIYTSSFFFLNSLFNPFIYALFYP

>CL337.Contig3_All

RPSETAVHVILYLILTAVILLTVCGNLLVIIAVCHFKELHTPTNVLILSLAFSDILTGLT

VMPFQFVWLIESCWIFGAAMWTLYSFATF

>CL6517.Contig1_All 83 1054 minus strand PREDICTED: trace amine-associated receptor 7a-like [Danio rerio]

KANYCYPSLNSSCAKLNRSPAIYIMLYILLSSVSLSTVVLNLLVIISISHFKQLHTPTNL

LILSMAVADLLIGLIAMPVEGSQTIETCWYFGDTLCSIYPLICSVALSASLCSLVLISTD

RFIAITDPLRYSVNVTLNKTNAVVVLGWSSCVVYYFFFLNDHLMQREPHRICHGHCLPTI

KFPWIVCDIIVSFIVPCSTVIVLNLKIFCTAHHQAQAISSVTEGANAAQENKMTKTSNRK

AAKNIGTLVTVYLLCYMPYFISVFAYIHPSLVTGLIWIMYMNSCINPIIYALFYPWFKLS

AKHIITLAIFHKDSSYLNVLKUNS

>CL6517.Contig2_All 148 1113 minus strand PREDICTED: trace amine-associated receptor 7a-like [Danio rerio]

QYCFPSNNLSCLREMRTRSETVIMYIFFSALSGCTVFLNLLVIISISHFKQLHTPTNLLI

LSMAVADLLIGLIAMPVEGSQTIETCWYFGDTLCSIYPLICSVALSASLCSLVLISTDRF

IAITDPLRYSVNVTLNKTNAVVVLGWSSCVVYYFFFLNDHLMQREPHRICHGHCLPTIKF

PWIVCDIIVSFIVPCSTVIVLNLKIFCTAHHQAQAISSVTEGANAAQENKMTKTSNRKAA

KNIGTLVTVYLLCYMPYFISVFAYIHPSLVTGLIWIMYMNSCINPIIYALFYPWFKLSAK

HIITLAIFHKDSSYLNVLKUNS

>CL6517.Contig3_All 10 327 PREDICTED: trace amine-associated receptor 7a-like [Danio rerio]

FFFFNDHLMQREPHRICHGDCLLTIEFPMIVCDIILSFVVPCSTVISLNLKIFCTAHHQA

QAISSVTEGANAAQENTMTKTSNRKAAKNIGTLVTVYLLCYMPFYM

>Unigene103024_All 1 141 minus strand trace amine-associated receptor 11 [Danio rerio] >gi|56311450|emb|CAI29429.1| novel protein with transmembrane receptor (rhodopsin family) domain [Danio rerio] >gi|190339660|gb|AAI63664.1| Trace amine-associated receptor 1a [Danio rerio] >gi|190340270|gb|AAI63651.1| Trace amine-associated receptor 1a [Danio rerio]

LDHSIPELLYESLMWVAYINSLFNPIIYTFSYSWFRKKFATLFKRQF

>Unigene121087_All 60 272 PREDICTED: trace amine-associated receptor 7c-like [Danio rerio]

DESLALQYCYPWNNASCIKTVRPTYEYVVMFFIFSVISATTVVVNLLVVIAIAHFRQLHT

PTNLLILSLSV

>Unigene1931_All 148 1125 minus strand PREDICTED: trace amine-associated receptor 7c-like [Danio rerio]

QYCFPSNNLSCLREMRTRSETVIMYIFFSALSGCTVFLNLLVIISISHFKQLHTPTNLLI

LSLAVADFVVGLIVMPIESIRMIENCWYFGVIFCSLFPFILYLVVSASLGNLVFISIDRF

IAVNDPLRYNSKVTMRKTMFCIFLSWFCSIVYAVILLYDHLCNPVVHSSCVGQCTVVSDY

AVAMTDLIVTFLSPCMAMISLYVRIFHTARKQIKLITAAASNVCQLHSKAVVAKKSEHKA

AKTLGVVVLMYLICWIPYYICILTMSTISTSSLTINIFSWFMYSNSCINPIIYAFFYPWF

KTSAQHILSRKILDKSSSFRNLFPEN

>Unigene38592_All 23 277 PREDICTED: trace amine-associated receptor 7c-like [Danio rerio]

KSETKAAKTLGGLIAIYFLCWTPYFVVSLTAETLPYNPTVLHVTYWFLYIHSCINPLMYA

LFYPWFRISVKHILTLSILHSSSSY

>Unigene48722_All 92 1066 minus strand trace amine-associated receptor 10b [Danio rerio] >gi|56311449|emb|CAI29428.1| novel protein with transmembrane receptor (rhodopsin family) domain [Danio rerio]

LCYDAVNSSCVKFTYPLAIRVPLYVFLMTTVALTVVGNLLVIITVLHFKQLHTPTNYLIL

SLAVADLLVGSVVMPPSVVRVVESCWYWGELFCKVHTSTSMMCCAASIVNLTMISVDRYY

AISQPLLYKTKITISVVFIMNSITWIISALFGFGVVFLEVNLLGIRDLYNSIVCKGSCIF

LHSLLSSTLSSLLSFYLPGIIMACIYLKIFVLAQEQLKSIHTINTNVSGRPSMTKTERKA

TKTLAIIMGVFISSWIPFFLVFTINPYFGYGSPKILLEILGWVGYLNSTVNPFVYAYFYR

WFRQAFRIIMSGKIFRQHSSRTILI

>Unigene75350_All 82 258 PREDICTED: trace amine-associated receptor 6-like [Danio rerio]

HTEVIYCFPALNGSCKKEGRTQGGYIIMYILLFIISASTVFLNLLVIVSVSHFKQLHTP

>Unigene91808_All 1 636 minus strand trace amine-associated receptor 11 [Danio rerio] >gi|56311450|emb|CAI29429.1| novel protein with transmembrane receptor (rhodopsin family) domain [Danio rerio] >gi|190339660|gb|AAI63664.1| Trace amine-associated receptor 1a [Danio rerio] >gi|190340270|gb|AAI63651.1| Trace amine-associated receptor 1a [Danio rerio]

LIVSMSVADFLLGVIVMPPAMIEFLEKCWYFGDVLCKFHTAVDITLCNASVLHLTCISID

RFCAVSQPLQYKKRMTMCVAFIMISVSWVLSAMFGFIVIFPPANTTHQDTEITQENCIGG

CSGLHEKEASNVSYFFIFYFIPLTVMLSLYLRIFAIAIRQAHTIHVNKESNRDGKHNMTL

ADYKATKTLGIVIGVFLCCWTPFFICNVIDPV

>Unigene92362_All 2 355 minus strand trace amine-associated receptor 10b [Danio rerio] >gi|56311449|emb|CAI29428.1| novel protein with transmembrane receptor (rhodopsin family) domain [Danio rerio]

LLSFYIPGVIMLCIYLKIYLIAQQQVRSIHNKHMQSKSSAAASKMERKATKTLAIIMGVF

LSFWLPFFLCNIIDPLTGYSIPPLWFDMVVWIGYFNSTCNPLVYALFYSWFRKAFRII

>Unigene93314_All 54 239 minus strand PREDICTED: trace amine-associated receptor 6-like [Sarcophilus harrisii]

NISLSANVEFCYEALNVSCTRMTYPLSLRLTFYFFFVSTIIAIAAGNLLVMCTIVHFEQL

QT

>Unigene95434_All 39 266 trace amine-associated receptor 10b [Danio rerio] >gi|56311449|emb|CAI29428.1| novel protein with transmembrane receptor (rhodopsin family) domain [Danio rerio]

LCHEFLNGSCPKFTYPTAFRVPLYVFFGTSVVLTVVGNLLVIITVVHFKQLHTPTNYLIL

SLAVSDLLLGGFVMPP

>CL5101.Contig1_All 115 1107 minus strand trace amine associated receptor 14e [Danio rerio]

MILNEVNITGDCLVSINSTCLKIFSSMATYVSLYAFAAAIVIFTVCGNLLVIISVYHFKQ

LHTPTNVLILSLAISDCLVGLFVMPVHSMILIESCSLFGSNFCALYNMINFQLTFVSVYN

ISLIAVDRYFALSNPFVYSKKVSLFISFIIVIHVWIVSLFYNCALLYFNGNFKSKCPDKC

VPYVNEVWSTVDLMVVFMFPCATIIILYLKVFFIARRHAVLIRAAQKDRKRLDVQNSGDS

MRLERKAARVLGILVSVFLMCLIPYYICMMLMDALAQSYDRVVNTMLTLFFLNSTVNPII

YALFYPWFQKSMKLIFTFKICRTDSSLINVL

>CL5101.Contig2_All 62 334 minus strand trace amine associated receptor 14f [Danio rerio]

VFFIARRHAVLIRAAQKDRKRLDVQNSGDSMRLERKAARVLGILVSVFLMCLIPYYICMM

LMDALAQSYDRVVNTMLTLFFLNSTVNPIIY

>CL6297.Contig1_All 117 1055 minus strand trace amine associated receptor 14a [Danio rerio] >gi|56208554|emb|CAI21052.1| novel protein with neurotransmitter receptor domain [Danio rerio]

SCSMAYDSVTAYVLLYAFAAVVVLFTVCGNLFVIISVCHFKQLHTPTNILILSLALSDFL

VGISLMPVQSRFWIEPCSLFEPSLCTFFYMIGSHLTFVSVYNISFIAVDRYFALSNPFLY

SKKVSLCLACIITINVWIVSLFYNSALLYFNGNFNHKCPSRCLAYMNELWSTADLIAVFI

LPCVIITTLYMKVFFIARRHAVAIRATLKDRKKIGDQNNGDTMRLETKAAKALGTVVSVF

LICLIPYYICTLLFDVLGQSYHRVVNIMLFLFFVNSSINPIIYALFYPWFQKSVKLIFIC

RICRTSSSLINVL

>CL6297.Contig3_All 117 1055 minus strand trace amine associated receptor 14a [Danio rerio] >gi|56208554|emb|CAI21052.1| novel protein with neurotransmitter receptor domain [Danio rerio]

SCSMAYDSVTAYVLLYAFAAVVVLFTVCGNLFVIISVCHFKQLHTPTNILILSLALSDFL

VGISLMPVQSRFWIEPCSLFEPSLCTFFYMIGSHLTFVSVYNISFIAVDRYFALSNPFLY

SKKVSLCLACIITINVWIVSLFYNSALLYFNGNFNHKCPSRCLAYMNELWSTADLIAVFI

LPCVIITTLYMKVFFIARRHAVAIRATLKDRKKIGDQNNGDTMRLETKAAKALGTVVSVF

LICLIPYYICTLLFDVLGQSYHRVVNIMLFLFFVNSSINPIIYALFYPWFQKSVKLIFIC

RICRTSSSLINVL

>Unigene14363_All 2 340 minus strand trace amine associated receptor 14g [Danio rerio]

VHLTTLIESCSLFESTFCAWYSTITLLLSFVSIYNILCIAVDRYFALSMPFEYSKRMSVN

VAVILTANVWILSLLYNWALLYFNGNYIRKCPGRCLPSVSETWSQIDLVLFFI

>Unigene42548_All 28 204 trace amine associated receptor 14a [Danio rerio] >gi|56208554|emb|CAI21052.1| novel protein with neurotransmitter receptor domain [Danio rerio]

NADSMKSERKAARVLGILVSVFLVCLIPLNICTFLIDELGKSFDYVMNNMLTLLFLNST

>Unigene78742_All 101 940 minus strand trace amine associated receptor 14a [Danio rerio] >gi|56208554|emb|CAI21052.1| novel protein with neurotransmitter receptor domain [Danio rerio]

SCLGEAMVTPEYVTFYVCAILVVIITVFGNLLVIMSVCHFRQLHTPTNMLILSLAVADFM

VGLFVMPLGFIWMIEACWFFGVSMCAFLNFFAFQLTTASVHNVALIAVDRYLALSNPFLY

TKQITVRLTIIVALLTWVFSVGYNFSLLYCNGFFTTSLTLCPNECPVAVSEAWSWVDFVV

VFALPCSIMFVLYLKIFAIARRHANAIRAANSQGNPNAKQNDNVPKRSERKAAKVLGILV

FVFLLCVVPYYISSVMAEFIHSHIFEDILNYTSLILYLNS

>Unigene84000_All 55 933 trace amine associated receptor 13e [Danio rerio] >gi|56311444|emb|CAI29423.1| novel protein with transmembrane receptor (rhodopsin family) domain [Danio rerio]

DVSADQYCYPSVNGSCTKGVYNQATQVVLYIMFWTSMAVTVMGNLVVIISIVHFKQLHTP

TNMLVMSLALADLLLGLTVMPFSIIRSVDGCWYYGDAFCLLHSSFDMFLTSASIFHLIFI

AIDRHQAVCNPLLYHTRITLPVAWLMIAVSWSIAAAYSFSLLYSKANVKGFDDFLRSIYC

VGSCNLLFNALWGALDTMIAFFLPCSVMVGLYAKIFSVAKGHAKKIEDVSRGLNMNGEHV

IKSNSTEHKAAKTLGIVVGAFILCWLPFFVNSIVDPYTNFSTPVILFEIFTWL

>CL337.Contig2_All 1 285 trace-amine-associated receptor family member [Danio rerio]

YNVTLLALDRFVALNSPFFYLDKVTVYLSFIIAFLSWLFSIFYNFILLYFNGFFTVSPTE

EEGLNSSNEILFFADVMVVFVFPCCAMIFLYFRIF

>Unigene14007_All 126 1112 Si:ch211-119b12.1 protein [Danio rerio]

VNLSLVTEQHFCSNHTCDIFEVTPVDILTYVCKLAVIIMAVCGNLLVIISICHFKQLHTP

TNYIILSLAVVDMLVGITVLPGQLILLNSCLKTGMIFCYFTLICTYCLSFLSIYNVSLIS

LDRLYALWKPLLYASRVSVKVMSKVIFVTWIISLVYNLGLVYLVTTGRDDVLCIGDCSVA

LNEIWSTVDIFIVFVLPCSIIIVSYLKIFTIARKHARSISVSKKQTIPRIRSKGITLKAS

ERKAAFTLGKLVLVFVLCLLPPFLTLVFSDSIPDALLQEVMEGAMLVHYLHSAINPLVYA

FFYPWFRKCAKLILMLKTLNPDTALLVVL

>Unigene22708_All 82 1059 minus strand Si:ch211-119b12.1 protein [Danio rerio]

LSLLDSCVSCLGKTPVTTSYVALYVSAVIAVVVTVCGNLLVITSVCHFKQLHTPTNVLLV

FLAVTDFFVGIFVMPLQFVSLIESCWFFGAVACAFFNFVSFHLPTTSVHIVALIAVDRCM

ALSDPFFYFKKITVNLIIIIATLDWVFSLVYCFTLLYCTGFFTRALTLCPYTCLIAVVDE

IWSWVDFVLVFALPCSIMFVLYLIIFAIARRHANAIRAANSQVNPNARQNDNVPKRSERK

AAKVLGILVSVFLLCIVPYYIANLIPGRINAEIFELVLNITSALFYLNSLFNPIIYALFY

PWFRKCMKIILSCQICSPSSSVMHVM

>Unigene2345_All 2 115 Si:ch211-119b12.1 protein [Danio rerio]

NSLFNPIIYALFYPWFQKSMKLILTCRICTTDSSLMQV

>Unigene25026_All 63 296 minus strand Si:ch211-119b12.1 protein [Danio rerio]

KSERKAAKIIGIVVFVFLLSMVPYYISTLLSGAIDAKLVNIIMNYTSSFFYLNSMFNPFI

YVLFYPSFQKSMKLIFTV

>Unigene3763_All 2 289 minus strand Si:ch211-119b12.1 protein [Danio rerio]

PFNAIVILYFKVFLIARRHAVVIRATFKNRKRLEDQNNADSMASEGKAARVLGILVSVFL

ICLIPYNVSMLLTNTLDESHGHVVDNTVTLLLANST

>Unigene73382_All 7 474 unnamed protein product [Tetraodon nigroviridis]

VTCEGSCALLFSLLSSTLASVLTFYLPGTIVISIYLKILLIALEQEQTSRDRGLNVSMKP

PTNKIEAKATKTLAIVVGVFLSFWIPYFLIISFDPHFGYMIPPLLYDIFAWVGYLNSTFN

PIIYAYFYSWFRKAFQIIVSGKIFQRGSSRILLFUD

**>Unigene101623_All 1 387 PREDICTED: chemokine-like receptor 1-like [Oreochromis niloticus]**

YCSVFLLVIVSIDRCLCVIAPVWSQNHRTLRGSSVVIAVAWTLSALISLPTLVFSTTLEQ

PGRTICVYNDIVAKVSLQTLIVTGFVVGLAVPLVVIVVCYAIIMQKLRANRMSKSSRPFR

VMTAVIITF

**>Unigene66923_All 2 226 minus strand fMet-Leu-Phe receptor [Osmerus mordax]**

KNQAVKVGLTLAKGIAYFNSCVNPVLYFCMGLNLRHRLNQTLSSVYRRALTEDGDGPTTQ

SQEQGVDDSSASLPR
