# Supplementary material for: Identification of olfactory receptor genes in the Japanese grenadier anchovy Coilia nasus
Source: Genes Genomics. 2017 Feb 23;39(5):521–32. doi: 10.1007/s13258-017-0517-8 (PMC5387026; doi:10.1007/s13258-017-0517-8)
Supplement: Supplementary file 5 — Putative identified OlfC genes in Coilia nasus. (DOCX 20 KB) [file 13258_2017_517_MOESM5_ESM.docx]

**Appendix SVI. Putative identified OlfC genes in *Coilia nasus*.**

| **Unigene ID** | **Length** | **CDS (aa)** | **Blast annotation/Organism** | **E-value** | **TM(No)** | **Signal peptide** |
| --- | --- | --- | --- | --- | --- | --- |
| Unigene14297_All | 2699 | 827 | vomeronasal 2 receptor, x1 precursor (Danio rerio) | 0 | 7 | No |
| Unigene79724_All | 1937 | 583 | Vomeronasal 2 receptor, d1 (Danio rerio) | 0 | 1 | No |
| CL1147.Contig5_All | 3301 | 311 | olfactory receptor family C subfamily 17 member 2 gene (Salmo salar) | 1E-159 | 7 | Yes |
| Unigene8764_All | 1936 | 546 | olfactory receptor C family, d2 precursor (Danio rerio) | 0 | 0 | Yes |
| CL11881.Contig2_All | 423 | 141 | olfactory receptor family C subfamily 16 member 3 (Salmo salar) | 7E-48 | 0 | No |
| Unigene26668_All | 2687 | 863 | olfactory receptor family C subfamily 15 member 1 (Salmo salar) | 0 | 7 | No |
| Unigene82853_All | 898 | 237 | olfactory receptor family C subfamily 12 member 1 (Salmo salar) | 5E-101 | 6 | Yes |
| Unigene86482_All | 486 | 125 | olfactory receptor family C subfamily 3 member 1 (Salmo salar) | 1E-42 | 0 | No |
| Unigene88383_All | 227 | 71 | olfactory receptor family C subfamily 2 member 2 (Salmo salar) | 9.00E-14 | 0 | Yes |
| Unigene97945_All | 283 | 54 | olfactory receptor family C subfamily 16 member 1 (Salmo salar) | 3.00E-13 | 0 | No |
| Unigene61719_All | 619 | 202 | C-family odorant receptor OLFCT1 (Danio rerio) | 1E-83 | 1 | No |
| Unigene78794_All | 477 | 159 | Tanakia lanceolata V2R gene for vomeronasal receptor, partial cds, note: 5-35 | 9.00E-06 | 4 | Yes |
| CL10924.Contig1_All | 697 | 83 | Salmo salar olfactory receptor family C subfamily 17 member p1 pseudogene | 1.00E-08 | 0 | No |
| CL10924.Contig2_All | 513 | 152 | Salmo salar olfactory receptor family C subfamily 17 member p1 pseudogene | 1.00E-08 | 2 | Yes |
| CL1147.Contig10_All | 2849 | 685 | Salmo salar olfactory receptor family C subfamily 17 member 2 gene | 1E-163 | 7 | No |
| CL1147.Contig11_All | 3073 | 757 | Salmo salar olfactory receptor family C subfamily 17 member 2 gene | 1E-160 | 7 | No |
| CL1147.Contig12_All | 3232 | 810 | Salmo salar olfactory receptor family C subfamily 17 member 2 gene | 1E-160 | 9 | No |
| CL1147.Contig13_All | 2362 | 681 | Salmo salar olfactory receptor family C subfamily 17 member 2 gene | 1E-159 | 7 | No |
| CL1147.Contig14_All | 1657 | 725 | vomeronasal 2 receptor | 6E-158 | 0 | No |
| CL1147.Contig2_All | 3089 | 811 | Salmo salar olfactory receptor family C subfamily 17 member 2 gene | 1E-154 | 7 | No |
| CL1147.Contig3_All | 3297 | 811 | Salmo salar olfactory receptor family C subfamily 17 member 2 gene | 1E-166 | 7 | No |
| CL1147.Contig4_All | 3463 | 305 | Salmo salar olfactory receptor family C subfamily 17 member 2 gene | 1E-163 | 7 | No |
| CL1147.Contig6_All | 2865 | 757 | Salmo salar olfactory receptor family C subfamily 17 member 2 gene | 1E-156 | 7 | No |
| CL1147.Contig7_All | 3024 | 810 | Salmo salar olfactory receptor family C subfamily 17 member 2 gene | 1E-156 | 9 | No |
| CL1147.Contig8_All | 2981 | 729 | Salmo salar olfactory receptor family C subfamily 17 member 2 gene | 1E-160 | 8 | No |
| CL1147.Contig9_All | 2773 | 725 | Salmo salar olfactory receptor family C subfamily 17 member 2 gene | 1E-159 | 8 | No |
| CL5324.Contig1_All | 2733 | 380 | Salmo salar olfactory receptor family C subfamily 2 member 2 gene | 1E-119 | 0 | Yes |
| CL5324.Contig2_All | 2895 | 434 | Salmo salar olfactory receptor family C subfamily 2 member 2 gene | 1E-119 | 0 | Yes |
| CL6778.Contig1_All | 1657 | 486 | Salmo salar olfactory receptor family C subfamily 2 member 1 gene | 3E-33 | 7 | Yes |
| CL6778.Contig2_All | 1860 | 151 | Salmo salar olfactory receptor family C subfamily 2 member 1 gene | 3E-33 | 0 | Yes |
| CL6778.Contig3_All | 1002 | 41 | Salmo salar olfactory receptor family C subfamily 11 member 5 gene | 1.00E-6 | 0 | Yes |
| CL6778.Contig4_All | 799 | 187 | Salmo salar olfactory receptor family C subfamily 11 member 5 gene | 1.00E-6 | 0 | Yes |
| Unigene15989_All | 653 | 135 | Salmo salar olfactory receptor family C subfamily 13 member 1 gene | 1E-30 | 3 | Yes |
| Unigene54792_All | 325 | 108 | Salmo salar olfactory receptor family C subfamily 17 member p1 pseudogene | 6.00E-09 | 3 | No |
| Unigene61739_All | 762 | 252 | Salmo salar olfactory receptor family C subfamily 17 member p1 pseudogene | 7.00E-17 | 0 | Yes |
| Unigene62553_All | 500 | 166 | Salmo salar olfactory receptor family C subfamily 13 member 1 gene | 2.00E-10 | 3 | No |
| Unigene78793_All | 560 | 181 | Salmo salar olfactory receptor family C subfamily 17 member p1 pseudogene | 2.00E-10 | 0 | No |
| CL14841.Contig2_All | 439 | 86 | Vomeronasal type-2 receptor 1 OS=Mus musculus | 1.00E-06 | 0 | Yes |
| Unigene24070_All | 762 | 90 | Vomeronasal type-2 receptor 26 OS=Mus musculus | 3E-27 | 2 | No |
| Unigene28241_All | 470 | 48 | Vomeronasal type-2 receptor 1 OS=Mus musculus | 3.00E-11 | 1 | No |
| Unigene65091_All | 1366 | 427 | Vomeronasal type-2 receptor 1 OS=Mus musculus | 3E-87 | 0 | No |
| Unigene77993_All | 339 | 55 | Vomeronasal type-2 receptor 1 OS=Mus musculus GN=Vmn2r1 PE=1 SV=1 | 1.00E-14 | 1 | Yes |
| CL10326.Contig1_All | 3023 | 761 | vomeronasal 2 receptor | 0 | 7 | No |
| CL10326.Contig2_All | 3251 | 857 | vomeronasal 2 receptor | 0 | 7 | No |
| CL10326.Contig3_All | 282 | 93 | vomeronasal 2 receptor, h32 precursor (Danio rerio) | 5E-38 | 1 | Yes |
| CL14841.Contig1_All | 529 | 176 | vomeronasal 2 receptor | 3E-34 | 0 | Yes |
| Unigene10816_All | 653 | 166 | olfactory receptor family C subfamily 17 member 1 (Salmo salar) | 3E-81 | 4 | No |
| Unigene1953_All | 584 | 159 | olfactory receptor family C subfamily 17 member 2 (Salmo salar) | 1E-78 | 4 | No |
| Unigene51872_All | 396 | 131 | olfactory receptor family C subfamily 12 member 1 (Salmo salar) | 5E-52 | 0 | No |
| Unigene66147_All | 930 | 194 | olfactory receptor family C subfamily 3 member 1 (Salmo salar) | 3E-90 | 5 | Yes |
| Unigene71950_All | 1178 | 361 | olfactory receptor family C subfamily 11 member 4 (Salmo salar) | 1E-121 | 0 | No |
| Unigene55404_All | 259 | 80 | olfactory receptor family C subfamily 4 member 6 (Salmo salar) | 1E-22 | 0 | Yes |
