# Supplementary material for: Identification of olfactory receptor genes in the Japanese grenadier anchovy Coilia nasus
Source: Genes Genomics. 2017 Feb 23;39(5):521–32. doi: 10.1007/s13258-017-0517-8 (PMC5387026; doi:10.1007/s13258-017-0517-8)
Supplement: Supplementary file 6 — Putative identified MOR genes in Coilia nasus. (DOCX 35 KB) [file 13258_2017_517_MOESM6_ESM.docx]

**Table. Unigenes of putative main olfactory receptors (MORs).**

| **Unigene reference** | **Length (bp)** | **CDS (aa)** | **BLASTx best hit** | **E value** | **Full length** | **TM(No)** | **Signal peptide** |
| --- | --- | --- | --- | --- | --- | --- | --- |
| **CL1061.Contig2_All** | 1537 | 312 | main olfactory receptor family E subfamily 500 member 1, partial [Salmo salar] | 2E-96 | Yes | 7 | No |
| **CL10694.Contig1_All** | 1026 | 307 | main olfactory receptor family F subfamily 115 member 1, partial [Salmo salar] | 7E-113 | Yes | 7 | No |
| **CL10694.Contig2_All** | 678 | 203 | main olfactory receptor family F subfamily 115 member 1, partial [Salmo salar] | 4E-78 | No | 4 | Yes |
| **CL10694.Contig3_All** | 1061 | 309 | main olfactory receptor family F subfamily 115 member 1, partial [Salmo salar] | 1E-108 | Yes | 7 | No |
| **CL10694.Contig4_All** | 280 | 77 | main olfactory receptor family F subfamily 115 member 1, partial [Salmo salar] | 1E-19 | No | 1 | No |
| **CL13258.Contig2_All** | 236 | 72 | main olfactory receptor family E subfamily 120 member 1, partial [Salmo salar] | 3.00E-18 | NO | 1 | No |
| **CL6601.Contig1_All** | 2227 | 307 | main olfactory receptor family E subfamily 500 member 1, partial [Salmo salar] | 8E-104 | Yes | 7 | Yes |
| **CL6606.Contig4_All** | 962 | 236 | main olfactory receptor-like protein [Salmo salar] | 3E-72 | No | 5 | No |
| **Unigene115271_All** | 236 | 78 | main olfactory receptor family H subfamily 134 member 1 [Salmo salar] | 1E-27 | No | 2 | No |
| **Unigene16290_All** | 680 | 70 | main olfactory receptor family F subfamily 115 member 1, partial [Salmo salar] | 3E-20 | No | 2 | Yes |
| **Unigene18154_All** | 580 | 161 | main olfactory receptor family E subfamily 500 member 1, partial [Salmo salar] | 1E-48 | No | 3 | No |
| **Unigene21419_All** | 2009 | 308 | main olfactory receptor family F subfamily 115 member 1, partial [Salmo salar] | 1E-110 | Yes | 7 | Yes |
| **Unigene21420_All** | 1914 | 308 | main olfactory receptor family F subfamily 115 member 1, partial [Salmo salar] | 9E-111 | Yes | 7 | Yes |
| **Unigene23389_All** | 2145 | 309 | main olfactory receptor family E subfamily 500 member 1, partial [Salmo salar] | 3E-97 | Yes | 7 | Yes |
| **Unigene52959_All** | 914 | 237 | main olfactory receptor family F subfamily 115 member 1, partial [Salmo salar] | 5E-106 | No | 5 | No |
| **Unigene59895_All** | 417 | 108 | main olfactory receptor family F subfamily 115 member 1, partial [Salmo salar] | 2E-35 | No | 1 | No |
| **Unigene62329_All** | 1011 | 269 | main olfactory receptor family E subfamily 120 member 2, partial [Salmo salar] | 3E-70 | No | 5 | No |
| **Unigene68575_All** | 314 | 104 | main olfactory receptor family H subfamily 129 member 1 [Salmo salar] | 8E-33 | No | 2 | Yes |
| **Unigene68576_All** | 374 | 108 | main olfactory receptor family H subfamily 129 member 1 [Salmo salar] | 1E-37 | No | 3 | Yes |
| **Unigene95217_All** | 386 | 56 | main olfactory receptor family E subfamily 120 member 2, partial [Salmo salar] | 6E-9 | No | 1 | No |
| **Unigene96554_All** | 274 | 83 | main olfactory receptor family H subfamily 129 member 1 [Salmo salar] | 1E-24 | No | 2 | Yes |
| **CL10461.Contig1_All** | 1516 | 313 | odorant receptor, family F, subfamily 117, member 1 [Danio rerio] | 1E-103 | Yes | 7 | Yes |
| **CL10461.Contig2_All** | 1539 | 313 | odorant receptor, family F, subfamily 117, member 1 [Danio rerio] | 1E-103 | Yes | 7 | Yes |
| **CL1076.Contig1_All** | 1236 | 252 | odorant receptor, family H, subfamily 132, member 1 [Danio rerio] | 3E-51 | No | 6 | Yes |
| **CL1076.Contig2_All** | 1351 | 252 | odorant receptor, family H, subfamily 132, member 1 [Danio rerio] | 4E-51 | No | 6 | Yes |
| **CL11354.Contig1_All** | 1835 | 297 | odorant receptor, family A, subfamily 114, member 1 [Danio rerio] | 5E-87 | Yes | 8 | Yes |
| **CL12962.Contig1_All** | 891 | 247 | odorant receptor, family H, subfamily 132, member 2 [Danio rerio] | 7E-71 | No | 6 | Yes |
| **CL12962.Contig2_All** | 1341 | 258 | odorant receptor, family H, subfamily 132, member 2 [Danio rerio] | 7E-74 | Yes | 6 | Yes |
| **CL13258.Contig1_All** | 1028 | 281 | odorant receptor [Danio rerio] | 9E-87 | Yes | 7 | No |
| **CL1434.Contig2_All** | 1422 | 307 | putative odorant receptor [Carassius auratus] | 2E-114 | Yes | 7 | Yes |
| **CL14888.Contig1_All** | 1254 | 306 | odorant receptor [Danio rerio] | 6E-64 | Yes | 7 | No |
| **CL14951.Contig3_All** | 1292 | 210 | odorant receptor, family H, subfamily 132, member 1 [Danio rerio] | 4E-61 | No | 4 | No |
| **CL15592.Contig1_All** | 1101 | 307 | odorant receptor, family F, subfamily 117, member 1 [Danio rerio] | 1E-103 | Yes | 7 | No |
| **CL15592.Contig2_All** | 336 | 100 | odorant receptor, family F, subfamily 117, member 1 [Danio rerio] | 5E-30 | No | 1 | Yes |
| **CL15979.Contig1_All** | 2526 | 306 | odorant receptor [Tetraodon nigroviridis] | 4E-125 | Yes | 7 | No |
| **CL15979.Contig2_All** | 2869 | 306 | odorant receptor [Tetraodon nigroviridis] | 5E-125 | Yes | 7 | No |
| **CL2746.Contig1_All** | 2251 | 310 | odorant receptor, family H, subfamily 130, member 1 [Danio rerio] | 5E-76 | Yes | 7 | No |
| **CL2771.Contig2_All** | 1247 | 308 | odorant receptor [Tetraodon nigroviridis] | 6E-73 | Yes | 7 | Yes |
| **CL2791.Contig1_All** | 1247 | 109 | odorant receptor, family G, subfamily 106, member 1 [Danio rerio] | 1E-25 | No | 1 | No |
| **CL2791.Contig2_All** | 1444 | 310 | odorant receptor, family G, subfamily 106, member 1 [Danio rerio] | 7E-100 | Yes | 7 | No |
| **CL321.Contig1_All** | 1756 | 303 | odorant receptor, family E, subfamily 127, member 1 [Danio rerio] | 3E-102 | Yes | 7 | No |
| **CL321.Contig2_All** | 520 | 53 | odorant receptor [Larimichthys crocea] | 4E-10 | No | 1 | No |
| **CL3955.Contig1_All** | 504 | 131 | odorant receptor [Danio rerio] | 1E-29 | No | 2 | No |
| **CL3955.Contig2_All** | 1807 | 321 | odorant receptor [Danio rerio] | 3E-88 | Yes | 7 | No |
| **CL4703.Contig1_All** | 2493 | 297 | odorant receptor [Danio rerio] | 1E-47 | No | 6 | No |
| **CL4703.Contig2_All** | 2525 | 297 | odorant receptor [Danio rerio] | 1E-47 | No | 6 | No |
| **CL4703.Contig3_All** | 2880 | 297 | odorant receptor [Danio rerio] | 1E-47 | No | 6 | No |
| **CL5232.Contig1_All** | 1028 | 321 | odorant receptor ZOR6A [Danio rerio] | 6E-131 | Yes | 7 | No |
| **CL5232.Contig2_All** | 936 | 300 | odorant receptor ZOR6A [Danio rerio] | 4E-124 | Yes | 7 | Yes |
| **CL5445.Contig1_All** | 1852 | 313 | odorant receptor [Danio rerio] | 6E-80 | Yes | 7 | No |
| **CL5445.Contig2_All** | 1991 | 313 | odorant receptor [Danio rerio] | 7E-80 | Yes | 7 | No |
| **CL5918.Contig2_All** | 620 | 135 | odorant receptor, family H, subfamily 132, member 3 [Danio rerio] | 3E-28 | No | 3 | No |
| **CL6606.Contig1_All** | 1445 | 108 | odorant receptor, family F, subfamily 118, member 3 [Danio rerio] | 4E-83 | No | 3 | No |
| **CL6606.Contig2_All** | 836 | 239 | odorant receptor, family F, subfamily 118, member 3 [Danio rerio] | 8E-66 | No | 5 | No |
| **CL6606.Contig3_All** | 1596 | 108 | odorant receptor, family F, subfamily 118, member 3 [Danio rerio] | 4E-83 | No | 3 | Yes |
| **CL6688.Contig1_All** | 2314 | 301 | odorant receptor [Danio rerio] | 3E-108 | Yes | 7 | Yes |
| **CL6688.Contig3_All** | 2308 | 301 | odorant receptor [Danio rerio] | 3E-108 | Yes | 7 | Yes |
| **CL6688.Contig4_All** | 2287 | 301 | odorant receptor [Danio rerio] | 3E-108 | Yes | 7 | Yes |
| **CL6914.Contig1_All** | 1469 | 151 | odorant receptor, family F, subfamily 117, member 1 [Danio rerio] | 3E-100 | No | 4 | No |
| **CL6914.Contig2_All** | 368 | 111 | odorant receptor, family F, subfamily 117, member 1 [Danio rerio] | 4E-37 | No | 2 | Yes |
| **CL6921.Contig1_All** | 1699 | 319 | odorant receptor [Danio rerio] | 1E-74 | Yes | 7 | Yes |
| **CL6921.Contig3_All** | 1812 | 317 | odorant receptor [Danio rerio] | 2E-74 | Yes | 7 | Yes |
| **CL6972.Contig3_All** | 229 | 72 | odorant receptor, family H, subfamily 137, member 5 [Danio rerio] | 1E-16 | No | 2 | No |
| **CL717.Contig2_All** | 2202 | 308 | odorant receptor, family H, subfamily 132, member 2 [Danio rerio] | 9E-70 | Yes | 7 | No |
| **CL717.Contig3_All** | 1378 | 308 | odorant receptor, family H, subfamily 132, member 2 [Danio rerio] | 3E-70 | Yes | 7 | No |
| **CL7175.Contig2_All** | 326 | 49 | odorant receptor [Danio rerio] | 2E-9 | No | 1 | No |
| **CL7212.Contig3_All** | 2054 | 303 | odorant receptor, family E, subfamily 121, member 1 [Danio rerio] | 5E-91 | Yes | 7 | Yes |
| **CL7212.Contig4_All** | 203 | 62 | odorant receptor, family E, subfamily 122, member 1 [Danio rerio] | 1E-14 | No | 1 | Yes |
| **CL8025.Contig1_All** | 1178 | 291 | odorant receptor [Larimichthys crocea] | 1E-120 | No | 7 | Yes |
| **CL8025.Contig2_All** | 511 | 150 | odorant receptor [Larimichthys crocea] | 4E-57 | No | 3 | No |
| **CL8025.Contig3_All** | 1864 | 298 | odorant receptor, family F, subfamily 116, member 2 [Danio rerio] | 2E-122 | No | 7 | Yes |
| **CL9458.Contig1_All** | 1815 | 301 | odorant receptor, family C, subfamily 105, member 1 [Danio rerio] | 2E-96 | Yes | 7 | No |
| **CL9458.Contig2_All** | 770 | 217 | odorant receptor, family C, subfamily 105, member 1 [Danio rerio] | 7E-63 | No | 5 | Yes |
| **CL9638.Contig1_All** | 542 | 139 | odorant receptor, family C, subfamily 105, member 1 [Danio rerio] | 5E-42 | No | 3 | No |
| **CL9638.Contig2_All** | 315 | 99 | odorant receptor [Danio rerio] | 1E-27 | No | 3 | No |
| **Unigene11975_All** | 1619 | 166 | odorant receptor, family A, subfamily 114, member 1 [Danio rerio] | 3E-51 | No | 4 | Yes |
| **Unigene14513_All** | 3691 | 297 | odorant receptor [Danio rerio] | 1E-47 | No | 6 | No |
| **Unigene15712_All** | 1353 | 309 | odorant receptor, family H, subfamily 134, member 1 [Danio rerio] | 2E-130 | No | 6 | Yes |
| **Unigene16199_All** | 1478 | 305 | odorant receptor [Danio rerio] | 8E-82 | Yes | 7 | No |
| **Unigene18610_All** | 1673 | 316 | odorant receptor, family C, subfamily 105, member 1 [Danio rerio] | 1E-92 | Yes | 7 | Yes |
| **Unigene21223_All** | 1650 | 306 | odorant receptor [Danio rerio] | 1E-107 | Yes | 7 | No |
| **Unigene22779_All** | 871 | 206 | odorant receptor [Larimichthys crocea] | 1E-92 | No | 4 | No |
| **Unigene22851_All** | 466 | 114 | odorant receptor [Danio rerio] | 3E-24 | No | 3 | No |
| **Unigene29409_All** | 707 | 156 | odorant receptor, family H, subfamily 132, member 1 [Danio rerio] | 9E-41 | No | 3 | No |
| **Unigene30140_All** | 266 | 84 | odorant receptor [Tetraodon nigroviridis] | 4E-14 | No | 0 | No |
| **Unigene30141_All** | 266 | 86 | odorant receptor [Tetraodon nigroviridis] | 9E-19 | No | 3 | No |
| **Unigene46725_All** | 226 | 73 | odorant receptor, family E, subfamily 128, member 10 [Danio rerio] | 4E-16 | No | 2 | No |
| **Unigene48164_All** | 310 | 64 | odorant receptor [Danio rerio] | 3E-16 | No | 1 | No |
| **Unigene49175_All** | 275 | 67 | odorant receptor [Danio rerio] | 4E-13 | No | 2 | Yes |
| **Unigene54358_All** | 576 | 72 | odorant receptor [Danio rerio] | 4E-19 | No | 2 | Yes |
| **Unigene54574_All** | 252 | 75 | odorant receptor [Danio rerio] | 1E-09 | No | 2 | No |
| **Unigene64615_All** | 961 | 255 | odorant receptor [Danio rerio] | 1E-63 | No | 5 | Yes |
| **Unigene64616_All** | 348 | 68 | odorant receptor, family H, subfamily 132, member 4 [Danio rerio] | 5E-08 | No | 1 | No |
| **Unigene67012_All** | 279 | 72 | odorant receptor, family H, subfamily 131, member 1 [Danio rerio] | 2E-18 | No | 2 | Yes |
| **Unigene7032_All** | 1411 | 318 | odorant receptor, family H, subfamily 132, member 1 [Danio rerio] | 1E-90 | No | 6 | No |
| **Unigene74121_All** | 1065 | 285 | odorant receptor [Danio rerio] | 5E-69 | No | 6 | No |
| **Unigene76039_All** | 472 | 73 | odorant receptor, family F, subfamily 116, member 1 [Danio rerio] | 1E-23 | No | 1 | No |
| **Unigene81937_All** | 459 | 152 | odorant receptor, family F, subfamily 117, member 1 [Danio rerio] | 6E-44 | No | 4 | No |
| **Unigene81949_All** | 301 | 99 | putative odorant receptor [Carassius auratus] | 1E-40 | No | 2 | Yes |
| **Unigene84414_All** | 236 | 75 | odorant receptor [Danio rerio] | 1E-14 | No | 1 | Yes |
| **Unigene85149_All** | 930 | 191 | odorant receptor, family H, subfamily 131, member 1 [Danio rerio] | 6E-52 | No | 4 | Yes |
| **Unigene87980_All** | 641 | 196 | odorant receptor [Danio rerio] | 3E-34 | No | 4 | Yes |
| **Unigene88695_All** | 219 | 67 | odorant receptor [Danio rerio] | 8E-07 | No | 1 | Yes |
| **Unigene9043_All** | 1471 | 40 | odorant receptor, family F, subfamily 116, member 1 [Danio rerio] | 5E-10 | No | 1 | Yes |
| **Unigene91230_All** | 231 | 60 | odorant receptor, family H, subfamily 137, member 1 [Danio rerio] | 4E-15 | No | 2 | Yes |
| **Unigene95547_All** | 412 | 82 | odorant receptor [Danio rerio] | 5E-24 | No | 2 | Yes |
| **Unigene99783_All** | 435 | 121 | odorant receptor [Danio rerio] | 2E-26 | No | 3 | No |
| **Unigene14954_All** | 2181 | 296 | Takifugu rubripes odorant receptor (OR4208-1) gene, partial cds | 8E-19 | No | 6 | No |
| **CL1061.Contig1_All** | 471 | 155 | olfactory receptor family E subfamily 500 member 1 [Oncorhynchus tshawytscha] | 3E-45 | No | 3 | Yes |
| **CL10717.Contig1_All** | 2068 | 318 | PREDICTED: olfactory receptor 6C75-like [Danio rerio] | 1E-104 | Yes | 7 | No |
| **CL11354.Contig2_All** | 506 | 130 | PREDICTED: olfactory receptor 52A5-like, partial [Danio rerio] | 1E-36 | No | 3 | No |
| **CL11354.Contig3_All** | 2715 | 295 | PREDICTED: olfactory receptor 52A5-like, partial [Danio rerio] | 7E-99 | Yes | 7 | No |
| **CL11354.Contig4_All** | 2534 | 295 | PREDICTED: olfactory receptor 52A5-like, partial [Danio rerio] | 7E-99 | Yes | 7 | No |
| **CL11354.Contig5_All** | 2413 | 295 | PREDICTED: olfactory receptor 52A5-like, partial [Danio rerio] | 6E-99 | Yes | 7 | No |
| **CL2771.Contig1_All** | 1504 | 298 | PREDICTED: olfactory receptor 52D1-like [Oreochromis niloticus] | 5E-69 | Yes | 7 | Yes |
| **CL5918.Contig1_All** | 849 | 253 | PREDICTED: olfactory receptor 2M5 [Danio rerio] | 3E-54 | No | 6 | Yes |
| **CL6972.Contig1_All** | 1409 | 305 | PREDICTED: olfactory receptor 4F6-like [Danio rerio] | 6E-88 | Yes | 7 | Yes |
| **CL6972.Contig2_All** | 1277 | 305 | PREDICTED: olfactory receptor 4F6-like [Danio rerio] | 8E-88 | Yes | 8 | Yes |
| **CL7175.Contig1_All** | 1888 | 318 | olfactory receptor 1-1 [Takifugu rubripes rubripes] | 8E-94 | Yes | 7 | No |
| **Unigene109144_All** | 351 | 113 | PREDICTED: olfactory receptor 52D1-like [Danio rerio] | 1E-25 | No | 0 | No |
| **Unigene1237_All** | 1686 | 292 | PREDICTED: olfactory receptor 11A1-like [Oreochromis niloticus] | 7E-70 | Yes | 7 | No |
| **Unigene22006_All** | 309 | 36 | PREDICTED: olfactory receptor 2M3-like [Oreochromis niloticus] | 5E-06 | No | 1 | No |
| **Unigene23721_All** | 1146 | 290 | PREDICTED: olfactory receptor 11A1-like [Oreochromis niloticus] | 7E-94 | Yes | 7 | No |
| **Unigene25551_All** | 1915 | 297 | PREDICTED: olfactory receptor 51E1-like [Oreochromis niloticus] | 1E-90 | Yes | 7 | No |
| **Unigene28245_All** | 1309 | 118 | PREDICTED: olfactory receptor 11A1-like [Oreochromis niloticus] | 6E-65 | No | 3 | Yes |
| **Unigene34891_All** | 276 | 82 | PREDICTED: olfactory receptor 52D1-like [Danio rerio] | 3E-17 | No | 2 | No |
| **Unigene62103_All** | 202 | 67 | PREDICTED: olfactory receptor 2J2-like [Pan paniscus] | 1E-31 | No | 1 | No |
| **Unigene64046_All** | 677 | 158 | PREDICTED: olfactory receptor 52B2-like [Oreochromis niloticus] | 2E-31 | No | 4 | No |
| **Unigene67013_All** | 235 | 78 | PREDICTED: olfactory receptor 4F6 [Danio rerio] | 7E-21 | No | 2 | Yes |
| **Unigene78523_All** | 214 | 51 | olfactory receptor family E subfamily 500 member 1 [Oncorhynchus kisutch] | 1E-06 | No | 1 | No |
| **Unigene82069_All** | 984 | 299 | PREDICTED: olfactory receptor 4F6-like [Danio rerio] | 9E-94 | Yes | 7 | Yes |
| **Unigene86653_All** | 421 | 89 | PREDICTED: olfactory receptor 4K2-like [Danio rerio] | 1E-17 | No | 2 | Yes |
| **Unigene9188_All** | 1954 | 71 | PREDICTED: olfactory receptor 52R1-like [Oreochromis niloticus] | 7E-85 | No | 1 | No |
| **Unigene92818_All** | 204 | 67 | PREDICTED: olfactory receptor 4D9-like [Oreochromis niloticus] | 1E-07 | No | 1 | No |
| **Unigene92982_All** | 372 | 58 | Olfactory receptor 4F15 [Cricetulus griseus] | 5E-06 | No | 0 | No |
| **Unigene96506_All** | 237 | 78 | olfactory receptor, family 52, subfamily E, member 8 [Homo sapiens] | 1E-36 | No | 1 | Yes |
| **Unigene69531_All** | 412 | 137 | Homo sapiens olfactory receptor, family 7, subfamily E, member 105 pseudogene (OR7E105P) on chromosome 14 | 0 | No | 3 | No |
| **CL10990.Contig2_All** | 566 | 169 | Olfactory receptor 51Q1 OS=Homo sapiens GN=OR51Q1 PE=2 SV=2 | 8E-07 | No | 4 | No |
| **CL11013.Contig2_All** | 1435 | 148 | Olfactory receptor 4K17 OS=Homo sapiens GN=OR4K17 PE=2 SV=3 | 8E-08 | No | 3 | No |
| **Unigene228_All** | 1231 | 294 | Olfactory receptor 4F6 OS=Homo sapiens GN=OR4F6 PE=2 SV=1 | 5E-10 | No | 6 | No |
| **Unigene66561_All** | 264 | 31 | Olfactory receptor 7E24 OS=Homo sapiens GN=OR7E24 PE=2 SV=1 | 3E-32 | No | 0 | Yes |
| **Unigene97245_All** | 254 | 30 | Homo sapiens olfactory receptor, family 7, subfamily E, member 37 pseudogene (OR7E37P) | 1E-135 | No | 0 | Yes |
