# Supplementary material for: Identification of olfactory receptor genes in the Japanese grenadier anchovy Coilia nasus
Source: Genes Genomics. 2017 Feb 23;39(5):521–32. doi: 10.1007/s13258-017-0517-8 (PMC5387026; doi:10.1007/s13258-017-0517-8)
Supplement: Supplementary file 7 — Putative identified TAAR genes in Coilia nasus. (DOCX 18 KB) [file 13258_2017_517_MOESM7_ESM.docx]

**Table. Unigenes of putative trace amine associated receptors (TAARs).**

| **Unigene reference** | **Length (bp)** | **CDS (aa)** | **BLASTx best hit** | **E value** | **Full length** | **TMD(No)** | **Signal peptide** |
| --- | --- | --- | --- | --- | --- | --- | --- |
| **CL16219.Contig1_All** | 1256 | 338 | PREDICTED: trace amine-associated receptor 7g-like [Danio rerio] | 4E-95 | Yes | 7 | No |
| **CL16219.Contig2_All** | 847 | 276 | PREDICTED: trace amine-associated receptor 7c-like [Danio rerio] | 5E-81 | No | 6 | Yes |
| **CL337.Contig1_All** | 1024 | 318 | PREDICTED: trace amine-associated receptor 7a-like [Danio rerio] | 5E-69 | Yes | 7 | No |
| **CL337.Contig3_All** | 423 | 118 | PREDICTED: trace amine-associated receptor 7a-like [Danio rerio] | 8E-26 | No | 2 | Yes |
| **CL6517.Contig1_All** | 1197 | 327 | PREDICTED: trace amine-associated receptor 7a-like [Danio rerio] | 1E-84 | Yes | 7 | Yes |
| **CL6517.Contig2_All** | 1256 | 333 | PREDICTED: trace amine-associated receptor 7a-like [Danio rerio] | 1E-87 | Yes | 7 | No |
| **CL6517.Contig3_All** | 347 | 106 | PREDICTED: trace amine-associated receptor 7a-like [Danio rerio] | 1E-18 | No | 2 | No |
| **Unigene103024_All** | 293 | 47 | trace amine-associated receptor 11 [Danio rerio] | 4E-10 | No | 1 | Yes |
| **Unigene121087_All** | 272 | 71 | PREDICTED: trace amine-associated receptor 7c-like [Danio rerio] | 1E-17 | No | 1 | No |
| **Unigene1931_All** | 1566 | 326 | PREDICTED: trace amine-associated receptor 7c-like [Danio rerio] | 1E-95 | Yes | 7 | No |
| **Unigene38592_All** | 278 | 85 | PREDICTED: trace amine-associated receptor 7c-like [Danio rerio] | 1E-21 | No | 2 | No |
| **Unigene48722_All** | 1180 | 325 | trace amine-associated receptor 10b [Danio rerio] | 1E-101 | Yes | 7 | No |
| **Unigene75350_All** | 258 | 59 | PREDICTED: trace amine-associated receptor 6-like [Danio rerio] | 2E-15 | No | 1 | No |
| **Unigene91808_All** | 637 | 212 | trace amine-associated receptor 11 [Danio rerio] | 1E-57 | No | 4 | No |
| **Unigene92362_All** | 356 | 118 | trace amine-associated receptor 10b [Danio rerio] | 1E-38 | No | 3 | No |
| **Unigene93314_All** | 241 | 62 | PREDICTED: trace amine-associated receptor 6-like [Sarcophilus harrisii] | 9E-08 | No | 1 | Yes |
| **Unigene95434_All** | 268 | 76 | trace amine-associated receptor 10b [Danio rerio] | 5E-25 | No | 2 | No |
| **CL5101.Contig1_All** | 1391 | 331 | trace amine associated receptor 14e [Danio rerio] | 1E-88 | Yes | 7 | Yes |
| **CL5101.Contig2_All** | 336 | 91 | trace amine associated receptor 14f [Danio rerio] | 1E-14 | No | 2 | Yes |
| **CL6297.Contig1_All** | 1896 | 313 | trace amine associated receptor 14a [Danio rerio] | 8E-78 | Yes | 7 | Yes |
| **CL6297.Contig3_All** | 1282 | 313 | trace amine associated receptor 14a [Danio rerio] | 4E-78 | Yes | 7 | Yes |
| **Unigene14363_All** | 345 | 113 | trace amine associated receptor 14g [Danio rerio] | 1E-18 | No | 2 | Yes |
| **Unigene42548_All** | 205 | 59 | trace amine associated receptor 14a [Danio rerio] | 5E-06 | No | 1 | Yes |
| **Unigene78742_All** | 947 | 280 | trace amine associated receptor 14a [Danio rerio] | 6E-74 | No | 6 | No |
| **Unigene84000_All** | 934 | 293 | trace amine associated receptor 13e [Danio rerio] | 1E-117 | Yes | 7 | No |
| **CL337.Contig2_All** | 289 | 95 | trace-amine-associated receptor family member [Danio rerio] | 6E-17 | No | 2 | No |
| **Unigene14007_All** | 1302 | 329 | PREDICTED: Danio rerio trace amine-associated receptor 7c-like (LOC100002324), mRNA | 4E-07 | Yes | 7 | Yes |
| **Unigene22708_All** | 1900 | 326 | Trace amine-associated receptor 7b OS=Rattus norvegicus GN=Taar7b PE=3 SV=2 | 4E-53 | Yes | 7 | Yes |
| **Unigene2345_All** | 739 | 38 | Trace amine-associated receptor 4 OS=Rattus norvegicus GN=Taar4 PE=3 SV=1 | 6E-06 | No | 0 | No |
| **Unigene25026_All** | 469 | 78 | Trace amine-associated receptor 7a OS=Rattus norvegicus GN=Taar7a PE=3 SV=1 | 3E-12 | No | 2 | Yes |
| **Unigene3763_All** | 291 | 96 | Trace amine-associated receptor 3 OS=Mus musculus GN=Taar3 PE=2 SV=1 | 3E-06 | No | 2 | No |
| **Unigene73382_All** | 787 | 156 | Trace amine-associated receptor 1 OS=Macaca mulatta GN=TAAR1 PE=3 SV=1 | 3E-38 | No | 3 | No |
