# Supplementary material for: Identification of olfactory receptor genes in the Japanese grenadier anchovy Coilia nasus
Source: Genes Genomics. 2017 Feb 23;39(5):521–32. doi: 10.1007/s13258-017-0517-8 (PMC5387026; doi:10.1007/s13258-017-0517-8)
Supplement: Supplementary file 8 — Putative identified FPR genes in Coilia nasus. (DOCX 14 KB) [file 13258_2017_517_MOESM8_ESM.docx]

**Table. Unigenes of putative formyl peptide receptors (FPRs)**

| **Gene name** | **Unigene reference** | **Length (bp)** | **CDS (aa)** | **BLASTx best hit** | **E value** | **Full length** | **TM(No)** | **Signal peptide** |
| --- | --- | --- | --- | --- | --- | --- | --- | --- |
| **Unigene101623_All** | 388 | 129 |  | N-formyl peptide receptor 3 (Fragment) OS=Macaca mulatta GN=FPR3 PE=3 SV=1 | 3E-25 | No | 3 | No |
| **Unigene66923_All** | 333 | 75 |  | N-formyl peptide receptor 3 OS=Homo sapiens GN=FPR3 PE=2 SV=2 | 1E-07 | No | 1 | No |
